# Comprehensive Review on the Structural Diversity and Versatility of Multi-Resonance Fluorescence Emitters: Advance, Challenges, and Prospects toward OLEDs

**DOI:** 10.1021/acs.chemrev.5c00021

**Published:** 2025-05-09

**Authors:** Xiugang Wu, Songqian Ni, Chih-Hsing Wang, Weiguo Zhu, Pi-Tai Chou

**Affiliations:** † School of Materials Science and Engineering, Jiangsu Engineering Laboratory of Light-Electricity-Heat Energy-Converting Materials and Applications, 12412 Changzhou University, Changzhou 213164, China; ‡ 33561 National Taiwan University, Department of Chemistry, Taipei 10617, Taiwan

## Abstract

Fluorescence emitters with a multiple-resonant (MR) effect have become a research hotspot. These MR emitters mainly consist of polycyclic aromatic hydrocarbons with boron/nitrogen, nitrogen/carbonyl, and indolocarbazole frameworks. The staggered arrangement of the highest occupied molecular orbital and the lowest unoccupied molecular orbital facilitates MR, resulting in smaller internal reorganization energy and a narrower emission bandwidth. Optimal charge separation suppresses the energy gap between singlet and triplet excited states, favoring thermally activated delayed fluorescence (TADF). These MR-TADF materials, due to color purity and high emission efficiency, are excellent candidates for organic light-emitting diodes. Nevertheless, significant challenges remain; in particular, the limitation imposed by the alternated core configuration hinders their diversity and versatility. Most existing MR-TADF materials are concentrated in the blue-green range, with only a few in red and near-infrared spectra. This review provides a timely and comprehensive screening of MR emitters from their pioneering work to the present. Our goal is to gain understandings of the MR-TADF structure–performance relationship from both basic and advanced perspectives. Special emphasis is placed on exploring the correlations between chemical structure, photophysical properties and electroluminescent performance in both depth and breadth with an aim to promote the future development of MR emitters.

## Graphical abstract

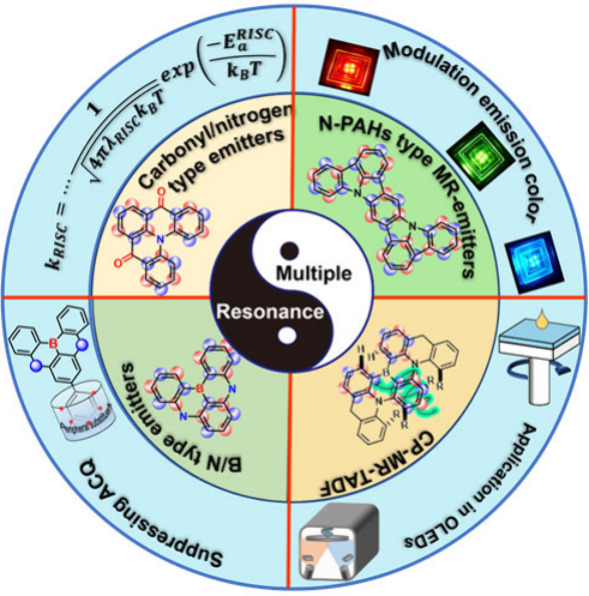

## 1 Introduction

Emitters that achieve 100% internal quantum efficiency of excitons for electron-to-photon conversion in OLEDs have been actively studied and applied to next-generation displays and solid-state lighting.^1^ Significant progress has been achieved with noble-metal emitters exhibiting phosphorescence and organic materials with characteristics of thermally activated delayed fluorescence (TADF). Both have made outstanding exciton utilization efficiency (EUE), contributing to the development of state-of-the-art OLEDs.^2–6^ However, due to their inherent charge transfer properties, both types of emitters exhibit wide emission bands and hence relatively poor color gamut, making them unable to meet the more stringent color purity and brightness requirement for next-generation displays. Developing organic luminophores with strong and narrowband emission capabilities is thus crucial for the further advancement of OLEDs, which yet is much more challenging. It was not until 2016 that Hatakeyama et al.^7^ proposed a seminal concept dubbed “multiple resonance” (MR), which was put into practice using compound DABNA-1 (1) (Scheme 1) to showcase the MR-TADF behavior. Note that some emitters are named differently in different papers, so numerical numbers are also used throughout the text to make it easier to find the structure. Currently, studies of MR-TADF-related materials and their application to OLEDs have become one of the research frontiers in chemistry and materials science, thanks to their unique emission spectral feature, which is characterized by a narrow full width at half maximum (FWHM).^8^ The FWHM of MR emitters can compete with the well-defined light emitting diode (LED) based on semiconducting materials such as gallium nitrides (micro-LEDs) and CdS/ZnS or CdSe/ZnS quantum dots (QD-LEDs).^7,9^ Today, high-definition displays have heightened the demand for luminescent materials. For instance, BT.2020 (Broadcast Service Television 2020) specifies the Commission Internationale de l’Éclairage (CIE) chromaticity coordinates for the three RGB hues (red, green, blue) as (0.708, 0.292), (0.170, 0.797), and (0.131, 0.046), respectively. In contrast, the corresponding standards for NTSC (National Television System Committee) are­(0.67, 0.33), (0.21, 0.71), and (0.14, 0.08). In practical RGB OLED displays, each subpixel emits a distinct spectrum for red, green, and blue, with each color appearing as a relatively narrow peak on a spectrum graph, which ensures high color purity and clear separation of primary colors, resulting in vibrant and saturated color reproduction. To meet these stringent requirements, the peak position of MR emitters should typically be around 630 nm for red, 530 nm for green, and 460 nm for blue. If the emission peak deviates significantly from these primary color windows, its practical application may be limited, regardless of how narrow the FWHM is. Only in this way can MR emitters, with their inherently narrow bandwidth, effectively minimize efficiency losses associated with color filters or optical microcavities in display applications.

**Scheme 1:**
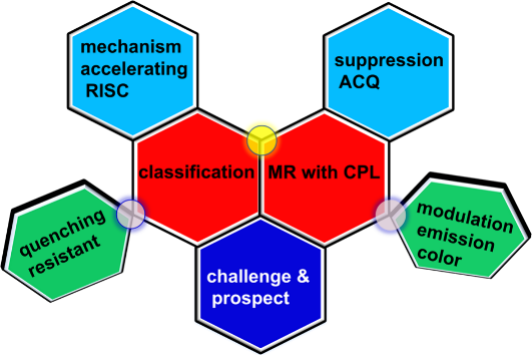
Key Themes of This Review

The underlying mechanism for MR-TADF molecules relies on their unique chemical structure. These molecules typically feature a fused planar polycyclic aromatic framework, which promotes a horizontal dipole orientation of MR-TADF emitters in vacuum-deposited OLEDs. This orientation enhances light outcoupling efficiency, thereby contributing to high external quantum efficiency (EQE).^10,11^ In these molecules, electron-donating, and electron-accepting units (see Figure 1), typically associated with nitrogen and boron, respectively, are arranged in a *para*-position. As a result, HOMO and LUMO are mainly ascribed to nitrogen and boron sites and accordingly to electron donor (D) and acceptor (A), respectively. Consequently, the electron density distributions between HOMO and LUMO orbitals are in a mutually staggered arrangement, separated proximally by one atom or so. Such small spatially separated HOMO and LUMO on the same planar moiety is denoted as a short-range charge transfer (SRCT). For MR molecules, this staggered type of charge separation manifests its distinction from conventional TADF emitters that possess a linear type of D-A charge separation and undergo a relatively long-range charge transfer (LRCT). Furthermore, the alternating distributions of HOMO and LUMO by one atom-inducing nonbonding character significantly lower the corresponding vibration frequencies in MR molecules, facilitating a reduction in the reorganization energy (λ) for the f S_0_ and S_1_ states, which to a great extent prohibits the high-frequency vibrational quenching and facilitate the narrow FWHM and high emission efficiency (as illustrated in Figure 1b).^12,13^ In principle, the decent separation between electron density distribution in an MR configuration leads to a decrease in the electron correlation energy. The net result is a reduction of the energy gap between the lowest-lying singlet (S_1_) and triplet (T_1_) states, defined as Δ*E*_ST_, and hence boosts the rate of reverse intersystem crossing (RISC) *k*_RISC_, as expressed in eq 1,

1 $$k_{RISC}=\frac{2\pi }{\hbar }{\left|S\left|{Ĥ}_{SOC}\right|T\right|}^{2}\frac{1}{\sqrt{4\pi \lambda_{RISC}k_{B}T}}\exp\left(\frac{-E_{a}^{RISC}}{k_{B}T}\right)$$

where *k*_*RISC*_ represents the rate constant of RISC, while |⟨*S*|*Ḧ*_*SOC*_|*T*⟩| denotes a spin–orbit coupling (SOC) matrix element. *E*_*a*_^*RISCM*^ (≈Δ*E*_ST_) is the activation energy of RISC. λ specifies the reorganization energy. Additionally, ℏ indicates the reduced Planck constant, *k*_B_ is the Boltzmann constant, and T signifies the absolute temperature.

**Figure 1:**
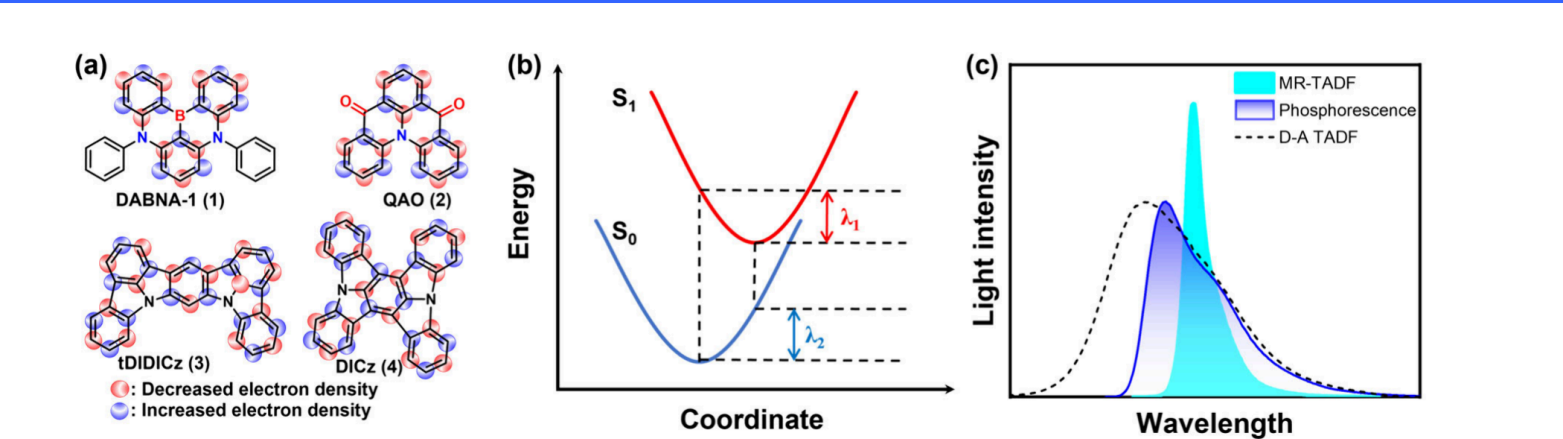
(a) Electron density distribution of representative MR emitters. (b) The multiple resonance effect facilitates a reduction in the reorganization energy (λ) for the S_0_ and S_1_ states. (c) MR emitters require lower energy peak emission to meet the same CIE_*y*_, owing to their superior efficiency and narrower bandwidth compared to phosphorescence and TADF counterparts.

Moreover, SRCT’s smaller spatially separated CT renders MR molecules larger S_1_→S_0_ transition moment. That is the larger radiative decay rate constant (*k*_r_) than that of the conventional TADF molecules. Larger *k*_RISC_ and *k*_r_, in theory, not only improve TADF efficiency, thereby increasing the emission quantum yield but also benefit the operation lifespan of OLEDs. The multiple resonant effect also elongates the π-electron delocalization. As a result, the corresponding vibration displacement due to the electronic excitation can be partitioned among the multiple resonance modes. This is effectively equivalent to lowering the internal reorganization energy (λ), which has been observed experimentally in many MR-TADF molecules, where the overlap between the lowest-lying absorption and emission bands is significant. Consequently, the emission Franck–Condon 0–0 vibronic transition is greatly enhanced, while other 0–*n* (*n* ≥ 1) transitions are largely suppressed. The resulting emission thus possesses a small FWHM. The same emission peak, combined with a narrower emission band, leads to a smaller CIE_*y*_ value. This explains why MR emitters with ultranarrow-band can effectively address the challenges in the practical realization of deep-blue OLEDs, even if their emission wavelength is slightly longer (∼460 nm) (Figure 1). In brief, MR-TADF materials offer several advantages, including small Δ*E*_ST_, narrow FWHM, large oscillator strength (*f*_osc_), horizontal dipole orientation, and high radiative decay rates (*k*_r_ ≈ 10^8^–10^9^ s^–1^). The latter, together with the fused, rigid planar structure that reduces nonradiative deactivation pathways, results in high photoluminescence quantum yields (Φ_PL_). These advantages have promoted the rapid development of MR-TADF in recent OLED-related research.

Materials can never be perfect, there are always pros and cons. Despite the above advantages, the strict structural configuration has also encountered obstacles in the practical applications of MR-TADF emitters. Major hurdles foreseen can be summarized from five aspects: (i) The rather stringent limitation on the core moiety, which requires alternated D/A configuration and hence their special frontier molecular orbitals (FMOs) distribution, restricts the chemical diversity of MR-type molecules. (ii) Similar to conventional TADF materials, MR-TADF materials also suffer a relatively long decay lifetime (τ) for the delayed fluorescence. (iii) MR-TADF property is strongly affected by functionalization, limiting its full derivatization. Especially, it remains challenging to harness emission colors, at least in the current stage, to deep-red and near-infrared (NIR) regions. (iv) Factors that improve spectral shape and FWHM are still under investigation, in particular how to eliminate the vibronic shoulder peaks that commonly appear in MR emitter spectra. (v) MR-TADF molecules are commonly associated with undesired aggregation upon film formation, causing quenching of the emission and/or unwanted emission, e.g., the broad excimer emission.

Several reviews on MR emitters have been published, each addressing specific aspects of this field. For example, Wai-Yeung Wong^14^ and Jang Hyuk Kwon^15^ et al. presented the recent progress on B/N-type MR-TADF emitters with fast *k*_RIS_ rate (*>*10^–5^ s^–1^). While Prof. Yue Wang^16^ and Lian Duan^17^ et al. reviewed research achievements in developing narrowband B/N-type MR-TADF materials employing the FMOs engineering strategy. Hatakeyama^14^ et al. provided a general introduction to organoboron-based MR emitters, mainly focusing on the synthetic strategies and clarifying structure–photophysical property correlations. These previous reviews are elegant in terms of academic major. However, it seems that each review provides fragments focusing on specific areas, which have a deficiency in the provision of omnidirectional scope and perspectives. Although Hyung Jong Kim and Takuma Yasuda reviewed recent progress in narrowband emissive MR-TADF systems (such as B/N- and N/-CO-based OLEDs) up to October 2022, their work covered only 131 papers, leaving room for a broader and more comprehensive perspective.^15^ Meanwhile, the boom of many novel varieties of MR emitters has received much attention, yet their properties, correlation with previous progress, and future advances need to be discussed in a more general manner. Therefore, it is timely to conduct a comprehensive review of recent MR emitters relevant works, providing an overarching perspective on various aspects such as their molecular structures, synthetic pathways, chemical advances, photophysical properties, and applications in OLEDs. One of the features and important results of this comprehensive review is the listing of approximately 683 MR-related compounds as of early 2025. We hope that readers with chemistry background will understand how chemical structure affects lighting performance, and how to systematically classify these materials based on their core structures and functionalities.

For clarity, this review is divided into six topics, including **1**. classification of MR-type molecules; **2**. emission color modulation; **3**. suppression of aggregation; **4**. acceleration of spin-flipping RISC; **5**. MR-TADF with circularly polarized emission; **6**. application in OLEDs. Each section provides essential background information, highlighting the specific advancements in MR-TADF and guiding future research directions (see Scheme 1). As a result, each section allows readers to seek the differences in the sameness and vice versa the sameness in differences, in the hope of offering informative references and constructive assistance to chemists interested in optoelectronics. The associated insights of fundamentals and applications would stimulate the readership to gain an understanding of the development of MR emitters in both depth and breadth.

## 2 Classification of MR-Type Molecules

MR emitters represent an ingenious modulation of D-A units embedded in rigid polycyclic aromatic hydrocarbons (PAHs) framework, which are known for their ultrapure emissions ideal for OLEDs. Such strategic design remains an exigent task to enrich structural diversity. Ever since the seminal MR concept was proposed and put into practice, making a ground-breaking work,^7^ a wide range of emitters based on heteroatom-embedded PAHs core have been explored.^16^ Their functionalization primarily involves boron, carbonyl, sulfone, and indolocarbazole fragments. This mainstream approach has created a myriad of MR libraries, where relevant progress has been receiving great attention from both academic research and the commercialization of OLEDs.^17^

### 2.1 Synthesis of MR Emitters

Elaborated below are the MR emitters integrated into OLEDs up to date, systematically categorized into three primary frameworks: boron (B)/ nitrogen (N)-type, N/carbonyl (-CO-)-type, and N embedded PAHs (N-PAHs-type). Thanks to the efforts of researchers, significant advances in synthetic strategies have driven the diversification of MR emitters. We first comprehensively detail the pivotal routes to successfully synthesize these target molecules, offering extensive information to aid researchers in similar synthetic endeavors.

#### 2.1.1 Synthesis of B/N-Type MR Emitters

Regarding B/N-type MR emitters, the boron atom’s vacant p-orbital and the nitrogen or chalcogens’ lone pair electrons in a *para*-positioned boron atom contribute to multiple resonant effects and facilitate short-range charge transfer (SRCT).^14^ The effects of the position and number of the boron and heteroatoms on the resulting photophysical properties have also been explored. Compound DABNA-1 (1) with B/N atoms in *para*-substitution was first reported by Hatakeyama and co-workers.^7^ This pioneering molecular architecture and its upgrades have been applied in state-of-the-art emitters and corresponding OLEDs. Chemically, borylation methods make a key contribution to the diversity of boron-based MR emitters, though suitable synthetic protocols are limited.^18^ Comprehensive reviews on the synthesis and structure–property correlations of organoboron-based MR emitters before 2023 have been extensively reported in the literature. Readers interested in earlier advancements in synthesis are encouraged to peruse the cited references.^14,17^ The synthetic pathway is synoptically depicted in Scheme 2 and described in general as follows from more than 400 reported B/N MR emitters.^15,16,18–25^

**Scheme 2:**
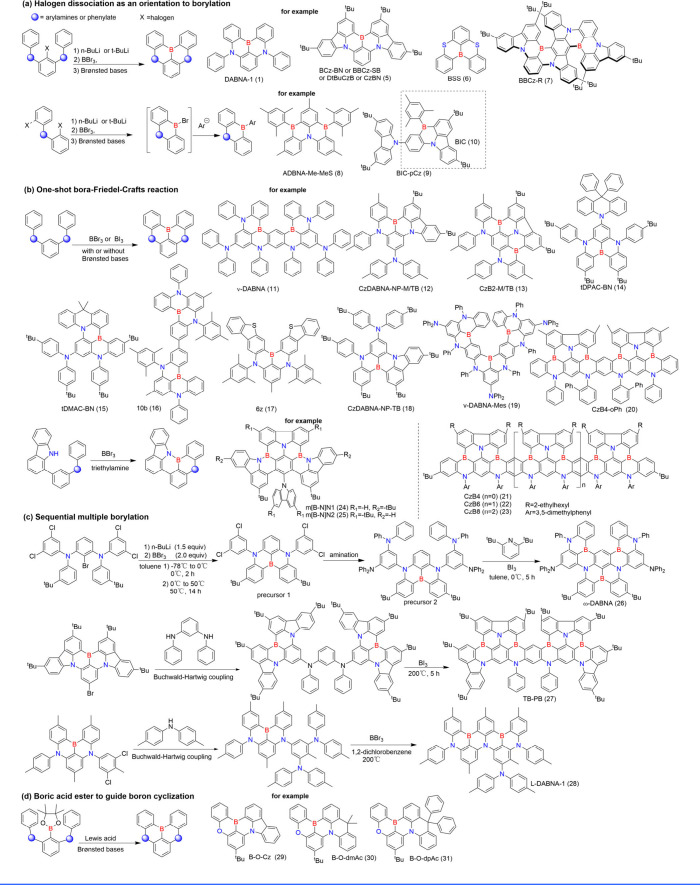
Borylation Methods of MR Emitters and Illustrative Examples

The synthetic method of DABNA-1 (1), which utilizes halogen dissociation as an orientation to borylation, has become a common practice for obtaining MR emitters, including but not limited to boron/sulfur-based BSS (6),^26^ multiple B/N-centered BBCz-R (7),^27^ and peripheral B/central N-type ADBNA-Me-MeS (8)^28^ (Scheme 2). The boron atom is introduced in one pot through a lithium-halogen exchange reaction with an organolithium reagent, followed by electrophilic trapping with boron tribromide, and tandem electrophilic arene borylation-annulation in the presence of suitable Brønsted bases, yielding the desired borylation compounds.^7^ This synthetic method employs initial lithiation using lithium alkylide to introduce the boron atom, but it significantly limits the choice of precursors and often results in low product yields.

Alternatively, a suitable borylation approach for triarylamines or phenylate eliminates the need for initial lithiation and instead employs sole borylating reagents, such as BBr_3_ or BI_3_, in the presence of appropriate Brønsted bases, significantly improving efficiency and yield (Scheme 2).^29,30^ This one-shot bora-Friedel–Crafts reaction is facile to construct boron-based PAHs even for multiple borylation reactions, such as the cascade synthesis of ν-DABNA (11).^9^ It is also worth noting that this method enables regioselective borylation, selectively targeting the ortho position of the HOMO-localized precursor while minimizing steric hindrance effects. For instance, by strategically introducing substituents such as methyl, t-butyl, phenyl, chloride, or steric diphenylamine at the C5 position of 1,3-benzenediamine substrates, carbazole derivatives (CzDABNA-NP-M/TB (12) and CzB2-M/TB (13))^31^ or acridan (tDPAC-BN (14) and tDMAC-BN (15))^32^ or methyl group (10b (16) and 6z (17))^33^ based DABNA-1 (1) analogs were achieved with remarkable regioselective borylation. The combination of DFT calculations and experimental investigation revealed that electronegativity and steric hindrance play critical roles in achieving efficient regioselective borylation. Moreover, the deprotonation process during the initial C–H borylation step was identified as the key rate-determining step in the reaction. Theoretical and XRD analyses determined that the reported structure TBN-TPA (18)^34^ via one-shot borylation is incorrect, which should be CzDABNA-NP-TB (18).^31^ Notably, the borylation yield was enhanced by employing excessive boron tribromide in an autoclave compared to standard reflux conditions in a flask, as demonstrated in the synthesis of V-DABNA-Mes (19), particularly for multiboron-centered PAHs.^35^ Additionally, CzB4-oPh (20), CzB4 (21), CzB6 (22), and CzB8 (23), featuring B/N-embedded multiacene frameworks, were synthesized via one-shot borylation reactions with nearly 100% yield. This remarkable efficiency is credited to the careful selection of borylation reagents and the incorporation of long-chain alkyl-substituted carbazolyl groups, which effectively mitigate HOMO energy reduction and prevent in-solubilization during the borylation process.^36^ Furthermore, the amine-directed formation of B-N covalent bonds enabled the construction of MR emitters with *para*-positioned nitrogen atoms, such as m­[B-N]­N1 (24) and m­[B-N]­N2 (25), to induce the MR effect (Scheme 2).^37^

Interestingly, the tandem reaction of lithium-halogen exchange reaction, amination, and one-shot borylation, called “sequential multiple borylations”, was reported for the synthesis of ω-DABNA (26) (Scheme 2).^38^ Notably, a significantly reduced yield (4%) was observed when 2,6-di*tert*-butylpyridine was omitted, highlighting its role as a sterically hindered base that selectively captures in situ generated hydrogen iodide to promote borylation and suppress deborylation. Similarly, the use of BBr_3_ at 180 °C resulted in a 23% yield of ω-DABNA (26) due to its lower reactivity, compared to a 50% yield obtained with BI_3_. A similar procedure was also employed in the synthesis of TB-PB (27), where a one-step Bora-Friedel–Crafts-type reaction was efficiently conducted in the presence of BI_3_ (bath temperature: 200 °C), yielding the target tetraborate compound TB-PB (27) with a 36% yield. However, alternative boron reagents, BCl_3_ and BBr_3_, did not produce any TB-PB (27).^39^ L-DABNA-1 (28), which could not be synthesized via conventional methods such as one-shot borylation, was successfully prepared through stepwise one-shot borylations.^40^ As shown in Scheme 2c, precursors bearing halogen atoms, particularly chlorine-based precursors, play an important role in subsequent reactions. On one hand, they are used in sequential amination, C–C coupling, and borylation—e.g., precursor 1 in the synthesis of ω-DABNA (26)—leveraging their lower reactivity compared to bromine.^33,38,41^ On the other hand, they are well-suited for constructing other functional compounds, such as cyano-modified emitters, as exemplified by ν-DABNA-CN-Me (267).^42^

Additionally, a relatively mild method utilizing boric acid ester to direct boron cyclization under the influence of a Lewis acid has been developed, resulting in asymmetrically structured B-O-Cz (29), B-O-dmAc (30) and B-O-dpAc (31) emitters (Scheme 2).^43^

To sum up briefly, the above-mentioned advancements in boronization methods have enabled the synthesis of B/N-type MR emitters to flourish, which has made a significant contribution to the development of high-performance OLEDs in recent years.

#### 2.1.2 Synthesis of Nitrogen/Carbonyl-Type MR Emitters

Unlike boron-based MR-TADF compounds, these nitrogen/carbonyl-type MR emitters with electron-donating N atom and electron-withdrawing carbonyl groups inducing resonance effect, are relatively easy to synthesize with higher yields. The classic synthetic route for the nitrogen/carbonyl-type MR core follows the procedure below: (a) The precursors, containing two ester groups in homo- or heteroaromatic rings (Scheme 3a), are obtained via Ullmann or Buchwald-Hartwig coupling reaction. After esters hydrolysis, benzoyl chloride derivates are yielded in the presence of chlorinated reagents like thionyl chloride or oxalyl chloride. Under the influence of a Lewis acid, the target MR emitters are ultimately synthesized through intramolecular Friedel–Crafts acylation, similar to the process used for accessing QAO (2), 3-PhQAD (32), and 7-PhQAD (33).^44,45^ (b) Another type of intramolecular cyclization is through the cyano group. Fluorobenzonitrile and diphenylamine derivatives undergo a nucleophilic substitution reaction in the presence of an inorganic base to yield a cyclization precursor. Subsequently, the cyano group, under the action of triflic acid, facilitates intramolecular annulation (Scheme 3).^46^ (c) Notably, spiro amines cannot react with dimethyl 2-iodoisophthalate in the Ullmann coupling reaction due to the significant steric hindrance between the reactants. To resolve this issue, the carbonyl group is temporarily reduced using a reducing reagent and then regenerated by an oxidant after the spiro structure is formed (Scheme 3). This feasible measure has been applied in the construction of SFQ (35), SOQ (36), SSQ (37), and SSeQ (38).^47^ Furthermore, to prevent side reactions involving the carbonyl group, the strategy of temporarily removing the carbonyl group and regenerating it in the final step is also applied in the spiro-functionalization of SS-DAO (39).^48^

**Scheme 3:**
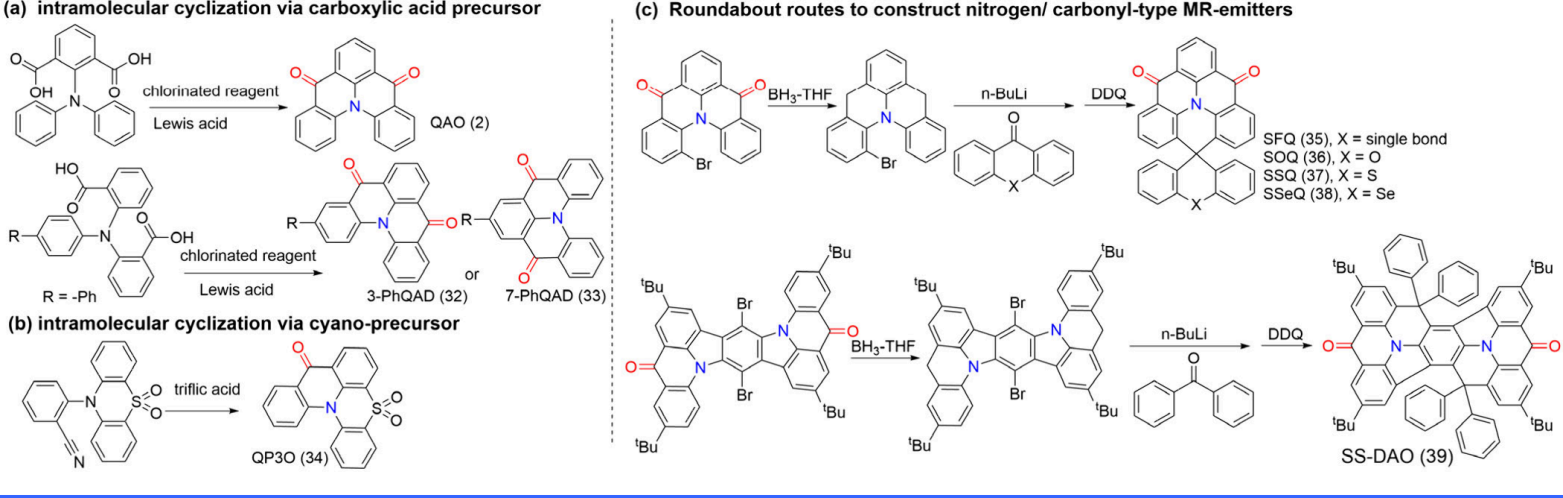
Intramolecular Cyclization of Nitrogen/Carbonyl-Type MR Emitters and Illustrative Examples

#### 2.1.3 Synthesis of N-PAHs-Type MR Emitters

The aforementioned MR emitters, including boron/nitrogen and carbonyl/nitrogen, all require an electron acceptor to exhibit SRCT properties. However, a new MR structure is emerging that does not include acceptor atoms. These MR emitters are represented by nitrogen (N)-atom embedded PAHs, commonly referred to as N-PAHs-type MR emitters.

Small molecules with a single N-PAH core, such as carbazole-derived indolo­[3,2,1-jk]-carbazole (ICz (342)), initially did not explore their potential for narrow bandwidth emission.^44^ The pure violet organic emitter, tDIDCz (3), which displays an impressively narrow bandwidth of up to 14 nm (105 meV), has reignited interest in research on this type of emitter.^49^ The key routes to cyclization of N-PAHs-type MR emitters proceed through either C–C coupling or C–N coupling, as demonstrated in the construction of tDIDCz (3) and t3IDCz (40), respectively (Scheme 4). The synthetic procedures seem to be routine compared to the harsh borylation required for B/N counterparts.

**Scheme 4:**
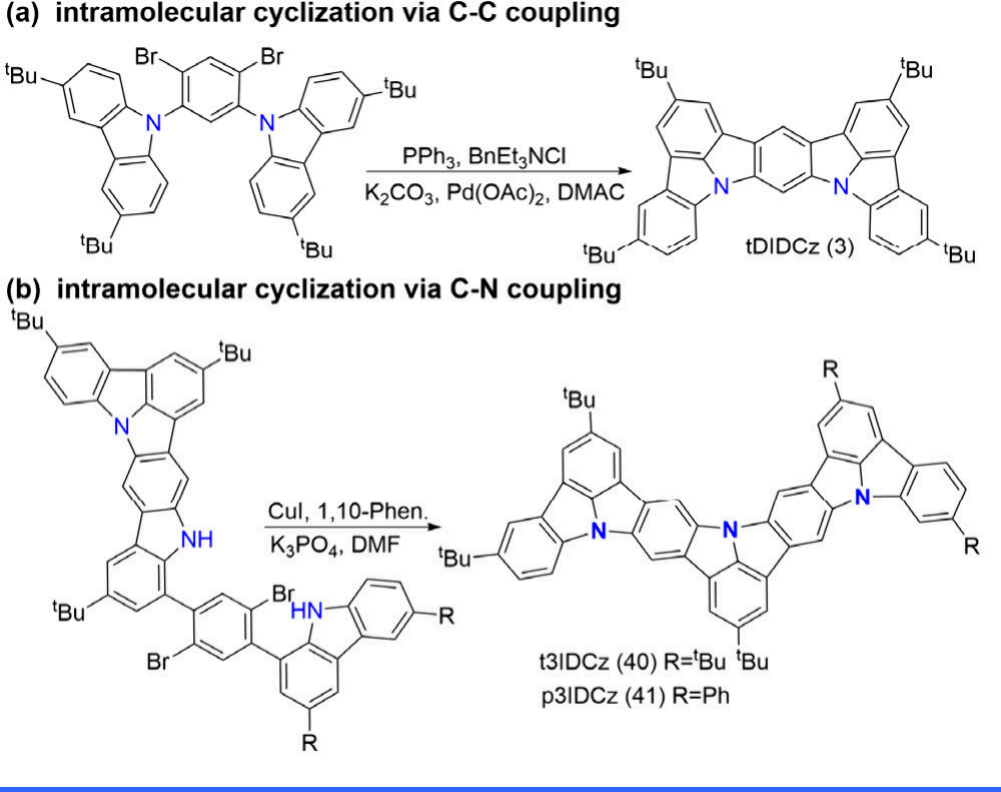
Intramolecular Cyclization of N-PAHs-Type MR Emitters and Illustrative Examples

In addition to a brief summary of the synthetic procedures (boration, carbonyl cyclization, and N-PAH cyclization), key intermediates such as DtCzB-Bpin (186) (see Figure 12) are also noteworthy as versatile building blocks for novel MR emitters. The detailed synthetic route can be referred to in specific reviews or source documents.

### 2.2 Diversity of B/N-Type MR Emitters

Boron-based emitters likely account for around 80% of B/N-MR emitters, significantly promoting the flourishment of MR emitters. Most performance parameters such as FWHM, color gamut, champion *k*_RISC_, and near-unity PLQY, are set by B/N-based MR emitters. In this section, we highlight B/N-type MR emitters and explore their diversity.

#### 2.2.1 One Boron-Centered MR Emitters

Single boron-centered MR emitters are easier to synthesize than their multiboron analogs. Using the pioneering compound DABNA-1 (1) as a prototype, a triangulene configuration featuring a boron center constrained by peripheral nitrogen atoms has become a hallmark design for single-boron MR emitters (Figure 2). In the quest for improved performance, novel donors have been incorporated into the triangulene framework. For instance, unlike the TADF counterparts B-N-S-1 (42) and B-N-S-2 (43), the tetrahydroquinoline-based donor compound B-N-S-3 (44) exhibits prompt fluorescence without TADF properties. However, in deep-blue OLEDs with an anthracene-based host, B-N-S-3 (44) extends the operating life of OLEDs by mitigating intersystem crossing of excitons.^50^ Additionally, a unique series of compounds—BN1 (45), TCz-BN1 (46), BN2 (47), TCz-BN2 (48), and BN3 (49)—featuring a rare tetracoordinate boron configuration with C^N^C- and N^N^N-chelating ligands, demonstrated how the B-N covalent bond influences optoelectronic properties.^51^ Notably, these emitters displayed broad FWHM values exceeding 82 nm, contrasting sharply with three-dimensional B/N counterparts that generally exhibit narrow FWHM values below 40 nm.^20,52^

**Figure 2:**
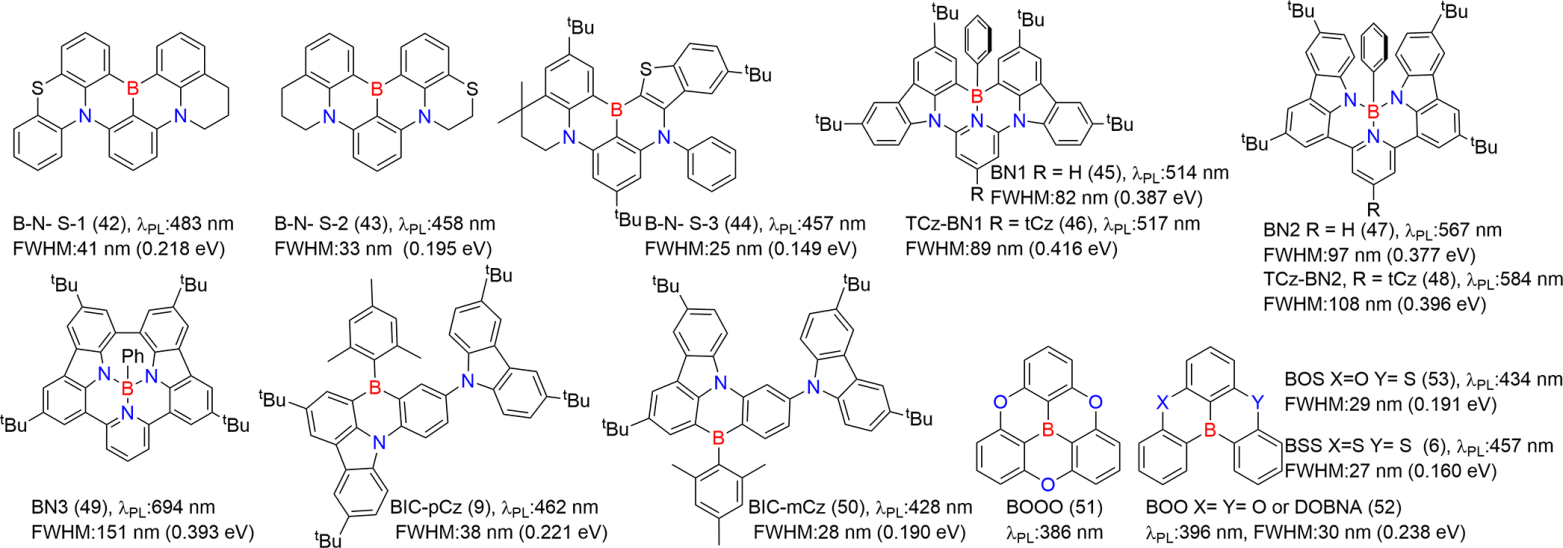
Chemical structures and photophysical properties of unconventional MR emitters based on one-boron in a toluene solution.

Expanding beyond triangulene-doped B/N emitters, Duan and collaborators introduced BIC-pCz (9) and BIC-mCz (50). These emitters are designed using mesityl-boron as the pendant acceptor and t-butylcarbazole (tCz) as the donor. This configuration reduces the charge transfer (CT) effect, inducing a hypsochromic shift compared to the classic ternary-doped BCz-BN (5) framework. Such a diversification highlights the continued development and optimization of one-boron-centered MR emitters.^53^

In addition to nitrogen-based electron-donating units, spacers such as carbon (C), oxygen (O), and sulfur (S) incorporated into MR frameworks are gaining increasing attention.^54–58^ Notably, replacing oxygen with sulfur (*Z* = 16), a heavier atom, enhances the heavy-atom effect, which strengthens SOC, reduces the singlet–triplet energy gap (Δ*E*_ST_), and facilitates faster reverse intersystem crossing (*k*_RISC_). Among these emitters, BOS (53) and BSS (6) stand out, combining electron-deficient boron atoms with electron-rich sulfur atoms within PAHs. These molecules exhibit strong TADF characteristics, attributed to the interplay between the electron-donating and electron-accepting centers.^26^ Further details regarding the mechanisms and factors influencing *k*_RISC_ rates in this class of emitters will be discussed in later sections, providing insights into the design strategies for enhancing device performance.

#### 2.2.2 Multiple-Boron MR Emitters

In theory, large molecular frameworks are more versatile for structural modifications and exhibit enhanced carrier recombination, exciton transfer, and energy transfer. These attributes lead to high PLQY.^59^ As a result, multiple boron-centered MR emitters hold great potential for superior lighting performance (Figure 3). One notable example is ν-DABNA (11), which features a robust framework comprising five benzene rings linked by two N-B-N resonant segments and two dangling diphenylamine groups. This design enables and enhances multiple boron/nitrogen resonance channels, which minimizes the displacement (*K*_*j*_) between S_0_ and S_1_, while also reducing the Δ*E*_ST_. These characteristics facilitate efficient *k*_RISC_, ultimately improving device performance.^9^

**Figure 3:**
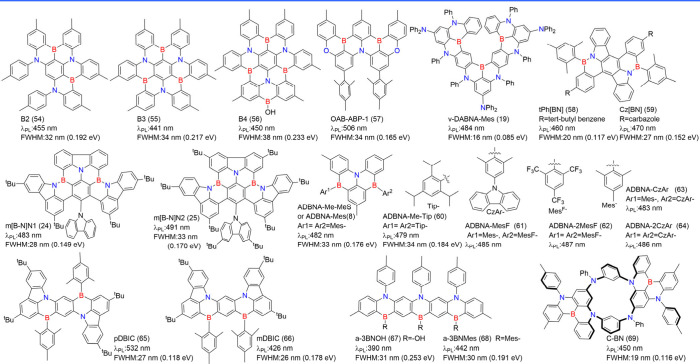
Chemical structures and photophysical properties of multiple boron-based MR-emitters.

Thanks to one-shot multiple borylation without initial lithiation (vide supra), various MR emitters with *meta*-positioned borons with different PAHs shapes have sprung up; these include clock-hand-like structures on the disk of B2 (54)-B4 (56),^60^ the linear layout green emitter of OAB-ABP-1 (57),^61^ and the helical V-DABNA-Mes (19)^35^ (see Figure 3). Notably, a new paradigm of emitters based on easy-to-access B-N covalent bonds through amine-directed borylation, e.g., tPh­[BN] (58) and Cz­[BN] (59), m­[B-N]­N1 (24), and m­[B-N]­N2 (25), exhibited narrowband sky-blue emission with high Φ_PL_, benefiting from B-N covalent effect incorporating the B/N multiresonance.^37,62^

At the current stage, a predominant configuration among multiple-boron-based MR emitters involves boron atoms positioned on the periphery and nitrogen atoms at the center, arranged in an alternating and staggered relationship to sustain the MR effect. This design capitalizes on the electron-deficient boron atoms and the electron-donating nitrogen centers to achieve unique photophysical properties. However, the peripheral boron atom with an empty *p*_*z*_ orbital is highly reactive and prone to instability in the presence of water, oxygen, or other nucleophiles. To address this issue, steric protection groups such as mesitylene (Me-MeS) are introduced to stabilize the *sp* ^*2*^-hybridized boron and constrain its structure.^63^ A landmark contribution in this domain comes from Hatakeyama and co-workers, who introduced this innovative B/N layout in ADBNA-Me-MeS (8) and ADBNA-Me-Tip (60), featuring two boron atoms and one nitrogen atom (Figure 3). These emitters were synthesized via nucleophilic substitution and electrophilic C-H borylation reactions. In doped films, ADBNA-Me-MeS (8) and ADBNA-Me-Tip (60) demonstrated sky-blue emission bands centered at 482 and 479 nm, respectively, with narrow FWHM values of 33 and 34 nm.^28^ To expand the structural diversity of such emitters, Wang and colleagues leveraged boronic acid-functionalized 1,4-B,N-anthracene as a versatile precursor. This approach facilitated the preparation of symmetrically and unsymmetrically functionalized derivatives of ADBNA-Me-Mes (8) (see Figure 3).^64^ Moreover, by fusing the structural framework of single-boron-based emitters such as BIC (10), binary B/N-based periphery emitters, namely pDBIC (65) and mDBIC (66), were synthesized. These emitters utilized N-π-N and B-π-B configurations in *para*- and *meta*-positions, respectively, to enhance π-delocalization. The π-delocalization consequently reduces the CT nature of the excited states, improving color tuning and emission properties, and paving the way for highly efficient narrowband emitters.^53^

Zysman-Colman and co-workers explored the use of boron moieties as peripheral components to create a linear, nontriangulated MR-TADF emitter, namely α-3BNOH (67). This compound displayed deep UV emission with a peak at 390 nm and a narrow FWHM of 31 nm in a THF solution.^65^ However, the hydroxyl functionalities in α-3BNOH (67) exhibited a propensity to form dehydrated dimers, similar to what was observed in studies with B4 (56) (see Figure 3).^60^ To overcome this limitation, hydroxyl groups in α-3BNOH (67) were replaced with sterically bulky mesityl substituents, leading to the development of α-3BNMes (68). This modification not only stabilized the molecule but also achieved the desired red shift of emission, resulting in an ideal blue emission with a peak at 442 nm and an FWHM of 30 nm.^66^ In another innovative approach, researchers synthesized a B/N-doped calix[4]­arene macrocycle, named C-BN (69). This macrocycle incorporated centrosymmetric double-DABNA fragments bridged by tertiary amine groups. The unique, strained structure of C-BN (69) induced large intermolecular distances between adjacent MR-emitting cores, effectively mitigating spectral broadening and the aggregation-caused quenching (ACQ) effect. Consequently, C-BN (69) exhibited exceptionally narrowband emission, positioning it as a promising candidate for advanced optoelectronic applications.^67^

### 2.3 Diversity of Carbonyl/Nitrogen-Type MR Emitters

Although the popularity of MR emitters is mainly attributed to the B/N doping type, there is a strong demand for boron-free MR emitters to diversify molecular design strategies. Among these, N/-CO-MR emitters (see Figure 4) have emerged as promising candidates, showcasing narrowband emission through the contrasting resonance effects between carbonyl and nitrogen atoms.^68^ The classic parent compound, QAO (2), was revitalized by Jiang et al.,^44^ who demonstrated its MR characteristics that were initially overlooked when it was categorized as a conventional fluorescent emitter.^69^ Its delocalized frontier molecular orbitals (FMOs) and photophysical properties are comparable to those of B/N-doped MR emitters.^44^ Around the same time, Zhang and co-workers introduced 3-PhQAD (32) and 7-PhQAD (33), which feature pure nitrogen/carbonyl frameworks and exhibit excellent device performance, particularly in FWHM and EQE, due to the MR effect.^45^ To further enhance the performance of nitrogen/carbonyl MR emitters, the strategy of bay-area fusing has been adopted to increase molecular rigidity and suppress structural distortions such as bending and rocking. For example, CZ2CO (70), which incorporates an additional five-membered aromatic ring compared to QAO (2), exhibited an ultranarrow FWHM of 16 nm (0.10 eV) with a peak wavelength of 440 nm.^70^ DQAO (71), OQAO (72), and SQAO (73), with carbon, oxygen, and sulfur atoms interlocking the bay area, respectively, displayed a red-shifted emission as the electron-donating strength of the amino substituents increased.^71^ Further advancements involve spiro-conjugation functionalization, which has proven effective in enhancing molecular rigidity, inducing a blue shift in emission, narrowing FWHM, increasing Φ_PL_, and suppressing intermolecular interactions. Examples include SFQ (35), SOQ (36), SSQ (37), and SSeQ (38), which demonstrated significant advantages over the parent QAO (2).^47^ TSFQ-TRZ (643) and TSFQ-Ph (644) (see Figure 4) incorporate a fused N/-CO-skeleton with varying adjacent segments—2,4,6-triphenyl-1,3,5-triazine (TPTRZ) and a phenyl group, respectively—linked through a rigid spiro spacer. TSFQ-TRZ (643) exhibited narrower emissions than TSFQ-Ph due to the TPTRZ segment, which introduces steric hindrance while simultaneously suppressing molecular vibrations through intramolecular interactions.^72^

**Figure 4:**
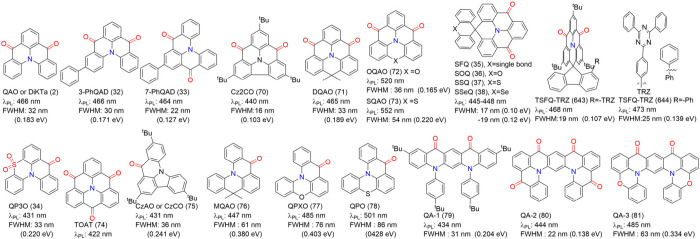
Chemical structures and photophysical properties of nitrogen/carbonyl-type MR emitters.

Another notable development is QP3O (34), a sulfone-incorporated nitrogen/carbonyl MR emitter first reported by Wu et al. QP3O (34), along with benchmark emitters such as DABNA-1 (1) and QAO (2), highlighted the critical role of host–guest interactions in enhancing TADF.^73^ A fascinating example supporting this perspective is trioxoazatriangulene (TOAT, 74), which features a completely flat structure with three bridging positions on the triphenylamine backbone locked by electron-withdrawing carbonyl groups. Despite its enhanced planarity induced by π-stacking and the presence of multiple resonance effects, TOAT (74) exhibited only modest room-temperature phosphorescence efficiency and no TADF.^74^

Asymmetric monocarbonyl locking in triphenylamine derivatives also induces the MR effect. For example, CzAO (75) (also referred to as CzCO^70^) achieved a narrow FWHM of 36 nm. By fixing the acetophenone moiety and varying the donor units, such as in CzAO (75), MQAO (76), QPXO (77), and QPO (78), it was revealed that the emission bandwidth is influenced by the extent of CT effects. A stronger CT effect generally correlates with a broader emission bandwidth.^75^

Finally, configurations involving multiple nitrogen/carbonyl skeletons were demonstrated in cis-quinacridone (cis-QA) derivatives, such as QA-1 (79), QA-2 (80), and QA-3 (81) (Figure 4). These compounds exhibit narrowband blue-to-green emission and are recognized as innovative MR emitters. Unlike widely studied trans-isomers, which lack TADF properties, cis-QA derivatives reduce Δ*E*_ST_, enhance SOC, and display prominent TADF characteristics.^76^

The structural diversity of N/-CO-MR materials, in terms of color modulation and device performance, is further elaborated in the following sections.

### 2.4 Diversity of N-PAHs-Type MR Emitters

N-PAHs-type MR emitters, despite lacking electron-withdrawing groups in their frameworks, exhibit SRCT properties. The incorporation and isomerization of indolocarbazole blocks play a critical role in these emitters (Figure 5). For instance, fusing fluorene with indolocarbazole creates the rigid chromophore IDCz (82), which demonstrated deep-blue emission.^77^ Emitters CNICCz (83) and CNICtCz (84), incorporating cyano-group (-CN)-modified indolocarbazole moieties as acceptors and carbazole (Cz) or t-butyl carbazole (tCz) as donors, exhibited deep-blue emission with peak wavelengths at 449 and 456 nm and narrow FWHMs of 56 and 60 nm, respectively.^78^ The rigid tripod structure of tDIDCz (3), centered around nitrogen and driven by multiresonance through alternating carbon and nitrogen atoms, displayed pure violet emission. Notably, this emitter avoided excimer emission in both solid and film states, thanks to peripheral t-butyl decorations that prevent intermolecular aggregation and packing. Additionally, tDIDCz (3) exhibited a fluorescence lifetime of 11.4 ns without a delayed fluorescence component in transient PL decay measurements.^49^

**Figure 5:**
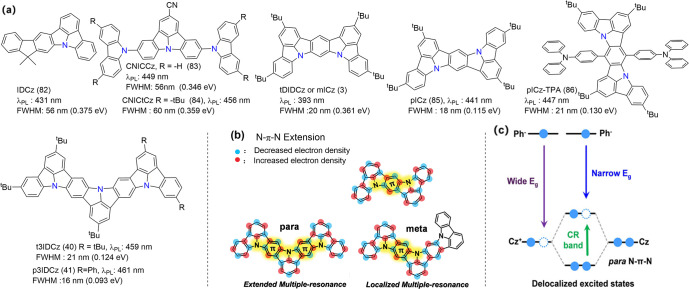
(a) The molecule structures of ICz-PAHs. (b) The design concept of 3IDCz with an N-π-N extended molecular structures. (c) The diagram of the formation of delocalized excited states.^79,80^ Reproduced with permission from ref 79 (copyright, 2021, John Wiley and Sons) and ref 80 (copyright, 2022, John Wiley and Sons).

To further optimize MR fluorophores, a strategy was proposed to incorporate indolocarbazole subunits, leveraging the synergistic effect of *para*-positioned nitrogen atoms to enhance electronic coupling and reduce the energy gap. As a result, deep-blue emitters pICz (85) and pICz-TPA (86) achieved emission peak wavelengths at 441 and 447 nm with remarkably narrow FWHMs of only 18 and 21 nm, respectively.^79^ Meanwhile, t3IDCz (40) and p3IDCz (41) were designed by fusing a trifused skeleton (3IDCz) via *para*-oriented nitrogen atoms to further extend along the *para*-N-π-N direction, whereas the MR extension was discontinued in the *meta*-positioned N-π-N direction.^80^

With the growing understanding of MR emitters, the incorporation of spacers such as carbon, oxygen, sulfur, and phosphorus has expanded the diversity of MR emitter libraries. For example, a promising approach involves integrating asymmetric O-B-N or carbonyl units into traditional B-N PAHs MR frameworks, forming rigid and extended π-skeletons (Figure 6). Regioselective one-shot electrophilic C-H borylation at different positions of the same precursor yielded compounds OBN (87), NBN (88), and ODBN (89), all of which exhibited deep-blue emission with CIE_*y*_ coordinates below 0.1.^82^ The incorporation of carbonyl groups into these frameworks enhanced intramolecular charge transfer and SOC, resulting in bathochromic-shifted narrowband emission and a faster *k*_RISC_ rate. This was exemplified by DOBDiKTa (91), synthesized by fusing tBuDOBNA (90) and DiKTa (2), which demonstrated desirable pure blue emission with efficient TADF characteristics.^84^ Both the proof-of-concept compound TCZBAO (93)^81^ and h-BNCO-1 (94)^83^ achieved green emission, narrow FWHM, and a *k*_RISC_ rate of magnitude10^5^ s^–1^. Sym-OBOICz (645) and asym-OBOICz (646) (see Figure 6b), synthesized via a one-pot method by merging boron/oxygen (B/O)-embedded MR triangulene and indolo­[3,2,1-jk]­carbazole units, exhibited significantly narrowed spectral bandwidths accompanied by red-shifted emission, owing to their fully resonating extended helical skeleton.^85^

**Figure 6:**
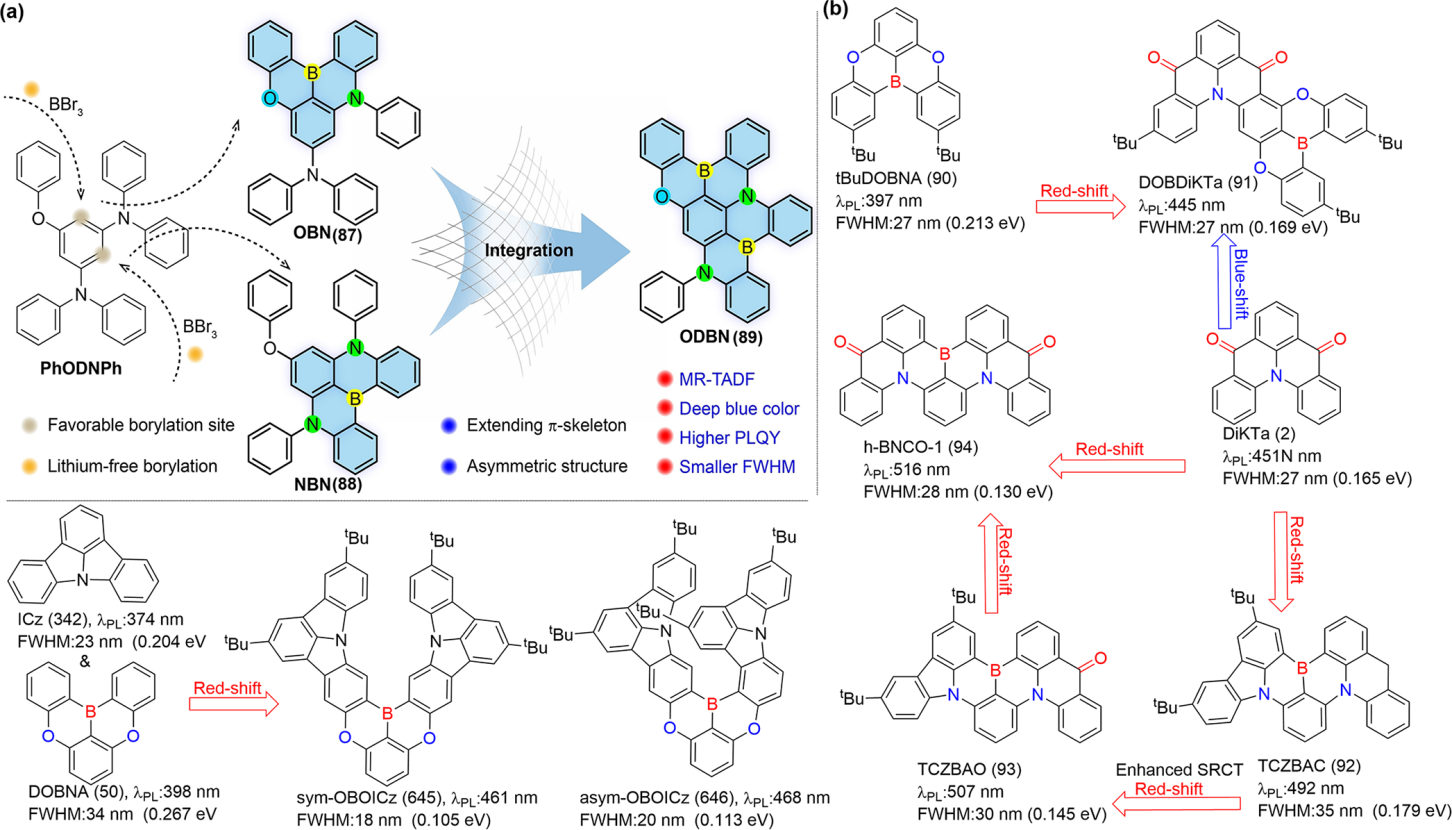
(a) The design strategy proposed for deep blue MR-TADF emitters. (b) The design strategy of carbonyl-fused organoboron PAHs.^82^ Reproduced with permission from ref 82. Copyright, 2023, John Wiley and Sons.

Recently, some sulfone-embedded PAHs such as tP (95), tCPD (96), 2tCPD (97), tPD (98), and tPT (99) have been emerging.^86^ Additionally, distinct from the p-π conjugation-induced MR-TADF in B/N systems, azaphosphinines compounds like CzP2PO (100) and tBCzP2PO (also named 2PO^87^) (101), C3PO (648) (Figure 7c), which feature σ*-π hyperconjugation with carbazole-phosphine oxide (P=O) fused aromatics, achieved narrowband emission with peak wavelengths below 430 nm (see Figure 7a).^88^ These collective endeavors not only highlight the innovative chemical design but also broaden the scope for creating more complex MR systems beyond the mainstream B/N MR frameworks.

**Figure 7:**
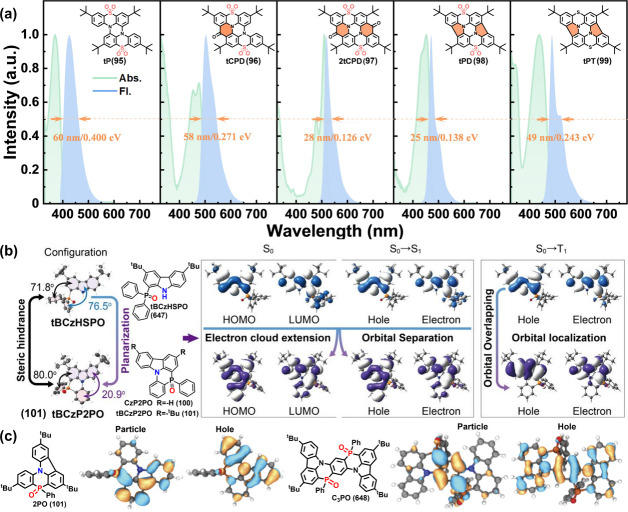
(a) UV–vis, absorption spectra (green line) and PL spectra (blue line) of MR molecules in dilute *n*-hexane solution at room temperature (inset: the corresponding molecular structures).^86^ (b) Single crystal structures, contours of HOMO and LUMO, and nature transition orbitals (“hole” and “electron”) of tBCzHSPO (647) and tBCzP2PO (101).^88^ (c) NTO orbitals of azaphosphinines 2PO (101) and C3PO (648) in S_1_ state.^87^ Reproduced with permission from refs 86, 88 (copyright, 2023, John Wiley and Sons) and ref 87 (copyright, 2025, American Chemical Society).

## 3 Modulation Emission Colors

The MR effect induced by heteroatoms like N/B, O/B, S/B, Se/B, or N/CO in rigid PAHs adheres to the “poly heteroaromatic omni-delocalization (PHOD)” principle, providing a robust framework for designing efficient pure blue fluorophores.^89^ Analyzing FMOs (see Figure 1) reveals that the electron density distribution of the SRCT characteristics in MR emitters indicates peripheral donors have minimal influence on emission color. In contrast, sacrificing color purity converts the system into a conventional D-A-type TADF emitter. Furthermore, MR-TADF emitters typically lack extensive conjugation; otherwise, the vibronic shoulder would increase, significantly broadening the emission bandwidth. Consequently, achieving full-color emission from MR emitters, particularly in the red and near-infrared regions, presents a fundamental challenge. To address this, strategies such as adjusting the configuration of electron-withdrawing and electron-donating units and modifying peripheral substituents have been explored to tune emission from deep blue to deep red, as discussed in the following sections.

### 3.1 Modulation Emission Colors of B/N-Type MR Emitters

For the B/N-type MR emitters, the nonbonding characteristics of heteroatoms within the B/N-embedded PAH framework theoretically disrupt conjugation and inhibit the extension of planar structures. Additionally, peripheral motifs have minimal impact on the distribution of FMOs. As a result, achieving a color redshift in B/N-type MR emitters is a challenging endeavor. It requires precise modulation of emission colors while simultaneously maintaining high luminescence efficiency and excellent color purity.

#### 3.1.1 Modulation Emission Colors of One Boron-Centered MR Emitters

The most effective strategy for tuning emission colors without compromising the FWHM is to balance the strengths of do nor and acceptor groups. This can be accomplished by functionalizing nitrogen (N), boron (B), or both, which are typically located in the *para*-position of the molecular framework (Figure 8).

**Figure 8:**
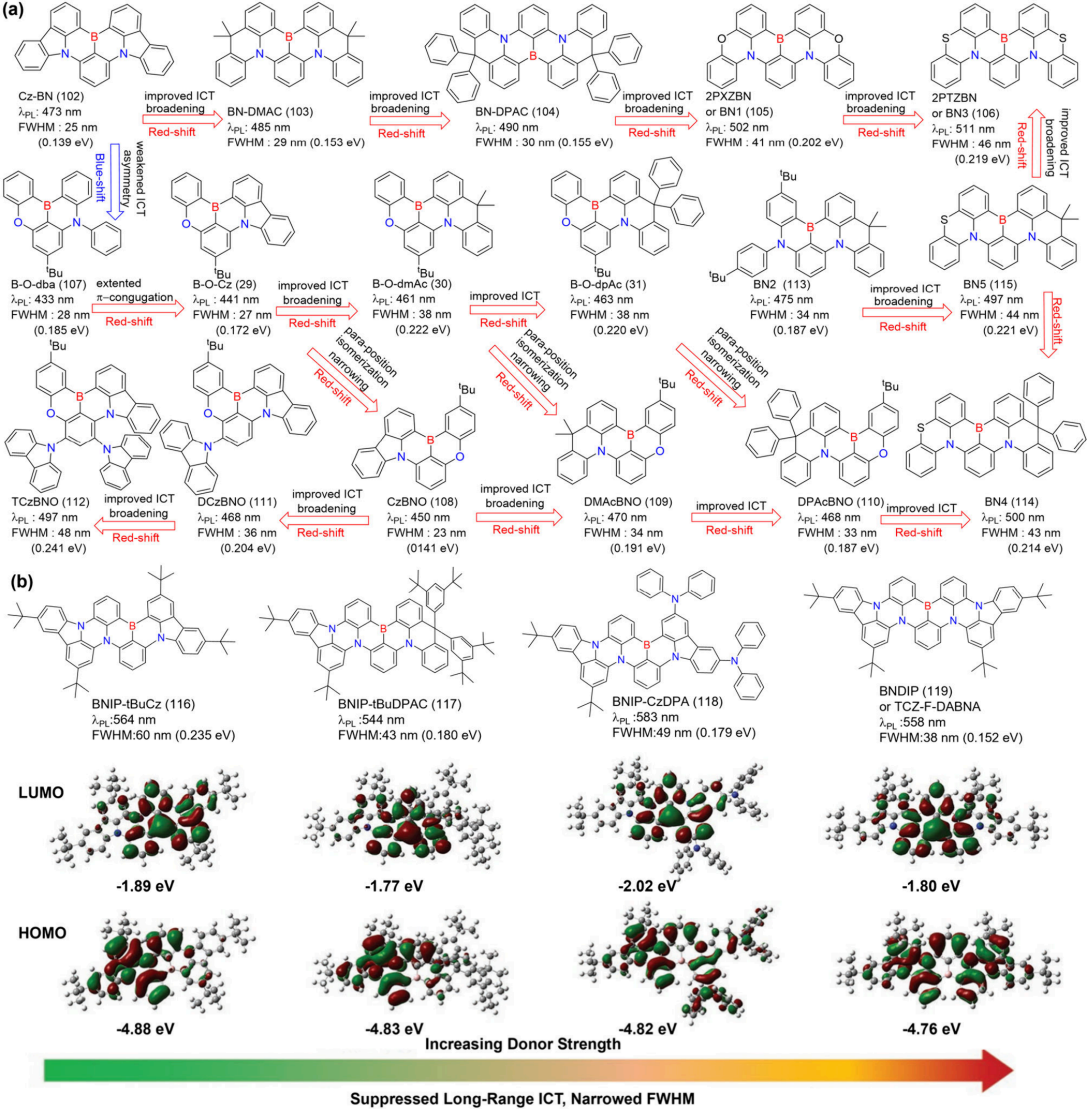
(a) Molecules based on ICT strength modulating emission colors and FWHM. (b) The optimized configurations, HOMO and LUMO energies, and distributions of BNIP-tBuCz (116), BNIP-tBuDPAC (117), BNIP-CzDPA (118), and BNDIP (119). Adapted from ref 90. Reproduced with permission from ref 90. Copyright, 2023, John Wiley and Sons.

Decorating the *para*-N position with electron-donating units, denoted as *para*-positioned D-π-N, enhances the donating capacity while introducing electron-withdrawing units to the *para*-B site of the central ring, denoted as *para*-positioned A-π-N, increases the electron-withdrawing ability of the boron atom. This configuration intensifies the intramolecular charge transfer (ICT) effect, leading to a bathochromic shift. Conversely, a hypsochromic shift can be achieved by attenuating the ICT effect, either by attaching electron-donating units to *para*-positioned D-π-B sites or electron-withdrawing units to *para*-positioned A-π-N sites.

Achieving both red-shifted emission and a narrow FWHM requires a delicate balance between ICT strength and the rigidity of the molecular framework, making the design process particularly challenging. In MR emitters, varying donor groups such as diphenylamine (DPA), carbazole (Cz), acridan (DMAc or DPAc), phenoxazine (PXZ), and phenothiazine (PTZ) demonstrate that stronger donor groups typically result in a bathochromic shift accompanied by an increase in FWHM. For instance, a series of Cz-BN-based emitters showed emission and FWHM trends as follows: Cz-BN (102) (λ_PL_ = 473 nm, FWHM = 25 nm),^91^ BN-DMAC (103) (λ_PL_ = 485 nm, FWHM = 29 nm),^92^ BN-DPAC (104) (λ_PL_ = 490 nm, FWHM = 30 nm),^92^ 2PXZBN (105) (λ_PL_ = 504 nm, FWHM = 34 nm),^93,94^ 2PTZBN (106, also named BN3) (λ_PL_ = 510 nm, FWHM = 39 nm).^94^

Further research underscores how modifying donor moieties in MR emitters, such as phenyl-borane (BN1 (105)-BN5 (115)) or acetophenone frameworks, influences ICT strength and, consequently, the emission bandwidth.^75^ Similarly, Lee and co-workers investigated asymmetric molecular structures by fixing weak electron-withdrawing oxygen atoms and varying donor units (e.g., DPA, Cz, DMAc, and DPAc). Their findings demonstrated that B-O-dba (107) exhibited blue-shifted emission (λ_PL_ = 433 nm, FWHM = 28 nm), surpassing the prototypical DABNA-1 (1) in color purity. In contrast, B-O-Cz (29), B-O-dmAc (30), and B-O-dpAc (31) extended π-conjugation and ICT effects, resulting in progressively red-shifted emissions with broader FWHMs.^43^ Additionally, modifications such as adding a tBu group at the *para*-B or *para*-O position further highlighted how fine-tuning electronic effects can effectively shift emission spectra. This is reflected by the bathochromic shift of 9 nm for CzBNO (108), 9 nm for DMAcBNO (109), and 5 nm for DPAcBNO (110) compared to B-O-Cz (29), B-O-dmAc (30), and B-O-dpAc (31), respectively.^95^ DCzBNO (111) and TCzBNO (112) exhibited a bathochromic shift and broadening FWHM due to the incremental addition of carbazole units compared to CzBNO (108).^96^ It should be noted that symmetrical donor configurations help maintain narrower FWHMs compared to their asymmetrical counterparts due to the balanced donor strength. For instance, asymmetrical indolophenazine-based MR-TADF emitters such as BNIP-tBuCz (116), BNIP-tBuDPAC (117), and BNIP-CzDPA (118) exhibited LRCT effects, resulting in larger FWHMs. In contrast, symmetrical designs like BNDIP (also known as TCZ-F-DABNA,^97^ 119) achieved narrower emissions with FWHM of 38 nm while effectively minimizing ACQ (Figure 8).^90^

Bathochromic shifts also can be achieved by incorporating PAHs segments, such as phenanthrene, triphenylene, and pyrene, into the MR core (Figure 9). These PAHs contribute stable, rigid skeletons with Clar π-sextets, thereby enhancing conjugations. For instance, DtBuPhCzB (120) displayed bluish-green emission (λ_PL_ = 496 nm, FWHM = 21 nm),^99^ while BP-2DPA (121) and DBP-4DPA (122) advanced into the red emission region emission (λ_PL_ = 599 nm and FWHM = 34 nm, λ_PL_ = 605 nm and FWHM = 34 nm, respectively).^100^ AN-BN (128)^101^ exhibited a more pronounced redshift effect compared to analogs such as BN-TP (123, λ_PL_ = 523 nm and FWHM = 34 nm),^102^ AZA-BN (130)^103^ and BN-TP-Nx (124–127) with aza-aromatics,^98^ and BN-Py (129),^104^ even though AN-BN (128) contains fewer π-sextets, highlighting the importance of precise conjugation.

**Figure 9:**
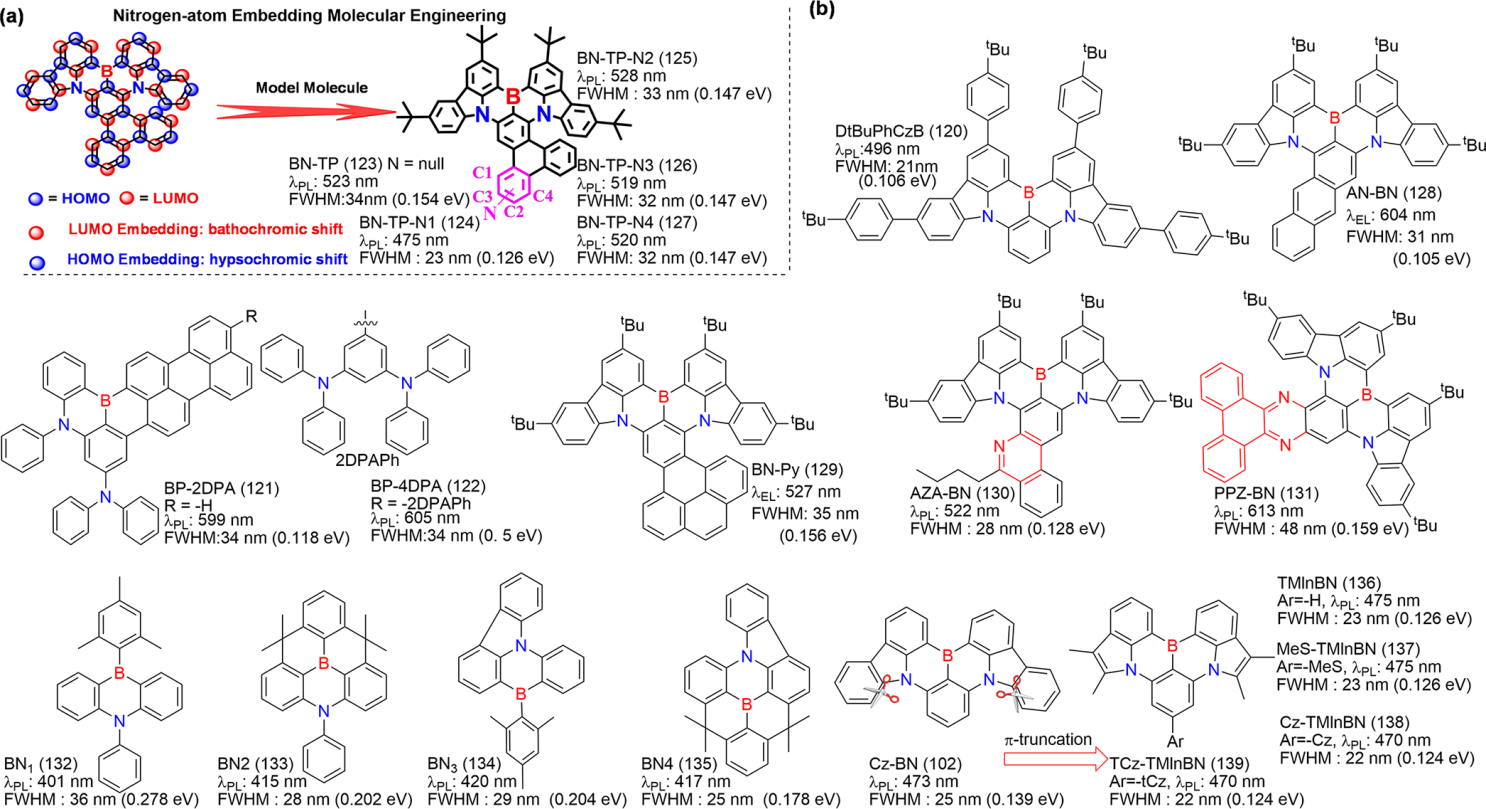
(a) Paradigm in polycyclization of B-N-containing MR parent core, frontier molecular orbitals population, and model molecule BN-TP (123), and photophysical properties of compound BN-TP-N*x* (*x* = 1, 2, 3, 4); adapted from ref 98. (b) MR emitters based on conjugation modulating emission colors and FWHM. Photophysical properties measured in toluene. Reproduced with permission from ref 98. Copyright, 2023, John Wiley and Sons.

The synergistic effect of increased π-conjugation and enhanced charge-transfer properties represents an effective strategy for tuning long-range emission colors. For example, PPZ-BN (131), which incorporates phenanthro­[9,10-b]­pyrazine, demonstrated pure-red emission with a significant redshift of over 128 nm compared to its parent compound, BCz-BN (5).^105^

Reduced π-conjugation is facile to design blue emitters. For instance, planarization of the triaryl-borane and/or triarylamine framework of the simple scaffold BN1 (132) improved photophysical properties in derivatives such as BN2 (133), BN3 (134), and BN4 (135) by enhancing the MR effect and SOC. Among these, the most planar structure, BN4 (135), exhibited superior TADF characteristics (see Table S2).^106^ Conversely, π-truncation can induce the emission blue-shifted, for example, a series of novel indole-fused MR-TADF emitters, denoted as TMlnBN (136), MeS-TMlnBN (137), Cz-TMlnBN (138), and TCz-TMlnBN (139) were developed via π-truncation of Cz-BN (102) to achieve narrowband blue emission.^107^

Figure 10 illustrates the color modification achieved through various parent nuclei using the aforementioned strategies. The pioneering DABNA-1 (1) exhibited a maximum emission at 462 nm with an FWHM of 33 nm.^7^ Based on DABNA-1 (1), TABNA (140) incorporated an additional aniline group on the *para*-positioned boron atom, creating another channel for the resonance effect. The decreased electron-withdrawing capacity of the boron unit resulted in an emission peak at 399 nm with an FWHM of 29 nm in the PMMA film.^108^ Phenylene-bridged MR-TADF emitters, OP-BN (141), Cz-OP-BN (142), and 2Cz-OP-BN (143), exhibited sky-blue emission at approximately 480 nm with a near-unity PLQY, a small FWHM of 26–31 nm in the solid state by tuning numbers of carbazole units.^109^ In contrast, PAB (144), featuring diphenylamine decorated on the *para*-positioned B-centered phenyl ring, exhibited a hypsochromic shift to 449 nm and an FWHM of 23 nm.^110^ 2FPAB (145), MePAB (146), and MePABF (147), incorporating fluorine and methyl groups based on PAB (144), exhibited ultrapure deep-blue emission peaks at 431, 446, and 463 nm, with identical FWHMs of 22 nm in a toluene solution.^111^ Similarly, A-BN (148) displayed deep-blue emission (CIE_*y*_ = 0.08) with near-unity PLQY and horizontal dipole orientation ratio (*Θ_∥_*) up to 90%.^112^ Two blue MR-TADF emitters, namely Me-PABO (149) and Me-PABS (150), by introducing dibenzofuran and dibenzothiophene to extend π-conjugate skeletons, showed large *k*_RISC_ values, slight bathochromic-shifted narrowband emission with a small FWHM value of 21 nm.^113^ t-DABNA (151), a derivative of DABNA-1 (1) featuring t-butyl groups surrounding the *para*-positioned noncentral nitrogen units, exhibited emission at 458 nm with an FWHM of 26 nm.^114^ By substituting the *para*-H of the central boron-located phenyl ring with di-t-butyl benzene to enlarge the conjugation structure, t-DABNA-dtB (152) achieved a red-shifted emission peak at 465 nm with an FWHM of 22 nm.^115^ Peripherally cladding with weak donor moieties, such as acridine and diphenylamine in a *para*-positioned boron atom, results in a B-π-N layout that facilitates a hypsochromic shift and a narrow FWHM. Examples include tDPAC-BN (14), 2TPAB (153), and 3TPAB (154) (also known as t-DAB-DPA^116^ or DABNA-NP-TB^117^), which exhibited emission peaks at 454–456 nm with FWHM values between 19 and 26 nm.^32,110^ These steric modifications also minimized undesired ACQ.

**Figure 10:**
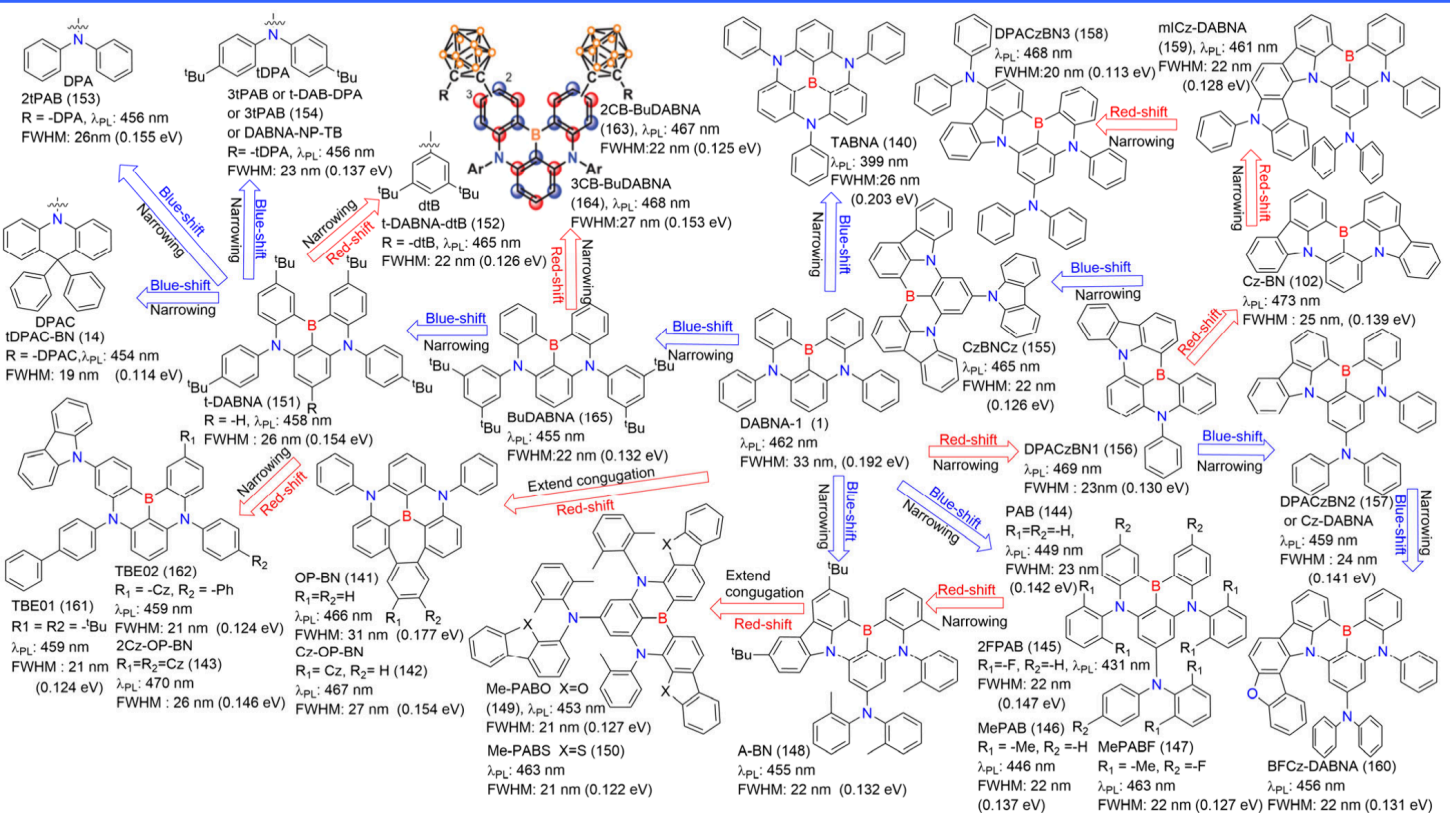
Modulating colors based on analogues of DABNA-1 (1).

The rotated benzene rings in the diphenylamine moiety enable a larger *K*_*j*_ compared to the interlocked bridging rings. Carbazole (Cz, HOMO = −5.44 eV) is a weaker donor moiety than diphenylamine (HOMO = −5.08 eV),^118^ while the interlinked benzene rings endow Cz with advantageous features such as larger conjugation, and more rigid planarity, leading to red-shifted emission and enhanced PLQYs. Among Cz-based DABNA analogs, Cz-BN (102) showed λ_PL_ at 473 nm with an FWHM of 25 nm, while CzBNCz (155) decorated at the *para*-position of the boron-centered phenyl ring, displayed emission at 465 nm with an FWHM of 22 nm, in line with the “*para*-positioned D-π-B” principle.^91^ Asymmetrically modified DPACzBN1 (156) and its diphenylamine-decorated derivative, DPACzBN2 (157) (also denoted as Cz-DABNA^119^), exhibited emission peaks of 469 and 459 nm, respectively, with FWHM values of 23 and 24 nm. The enhanced donor strength in DPACzBN3 (158) (λ_PL_ = 468 nm, FWHM = 20 nm) resulted in a bathochromic shift compared to DPACzBN2 (157).^120^

Further modifications highlight the interplay between donor strength and rigidity. mICz-DABNA (159) and BFCz-DABNA (160), with the introduction of electron-donating/-withdrawing properties of substituents, exhibited the bathochromic/hypsochromic shifted emission and narrower FWHM, respectively, compared to the parent Cz-DABNA (157).^119^ In TBE01 (161) and TBE02 (162) emitters, peripheral electron-donating Cz moieties, and benzene ring improved radiative recombination by increased *f*_osc_ as well as reduced electron exchange energy, giving smaller Δ*E*_ST_ due to more extended π-conjugation.^121^ Incorporating o-carborane units into the MR core of 2CB-BuDABNA (163) and 3CB-BuDABNA (164) led to red-shifted emissions relative to the parent BuDABNA (165).^122^

The above-mentioned strategy of “*para*-positioned D-π-N” or “*para*-positioned A-π-N” has further proven highly effective for modulating emission characteristics (see Figure 11). Yasuda et al. demonstrated that by employing imine and amine as donor (D) and acceptor (A) units to decorate carbazole moieties in Cz-BN (102), narrowband emissions could be systematically shifted from deep blue to yellow (461–571 nm). This modulation was observed in the series of compounds γ-Cb-B (166), Cz-BN (102), TCz-B (167), DACz-B (168), and DG7 (169).^123,124^ Similarly, Yang and co-workers synthesized a series of Cz-BN (102) derivatives featuring end-capped carbazole and diphenylamine groups. By varying the electron-donating ability and the number of peripheral groups, they achieved systematic color tuning of narrowband emissions. The resulting emitters, BN1 (170) to BN3 (172), displayed a range of colors from bluish-green (BN1 (170)) to green (BN2 (171)) and yellow (BN3 (172)).^125^ BN1 (170) exhibited a blue-shifted emission with a larger FWHM compared to BN2 (171) due to the higher donor strength and reduced rigidity of diphenylamine relative to carbazole. Additionally, the comparison between BN2 (171) and BN3 (172) confirmed that asymmetrical peripheral-donating groups generally result in a larger FWHM. Relative to the parent molecule Cz-BN (102), emitters CzBN-tDPA (173) and CzBN-mCP (174), decorated with different diarylamino moieties, exhibited blue emission peaks at 465 and 464 nm with narrow FWHM values of 23 and 21 nm in a toluene solution, respectively. These modifications also enhanced the *k*_RISC_ and the Φ_PL_.^126^ Additionally, N-π-N fragments simultaneously enhance donor ability and expand the π-conjugation. They further fine-tune the incorporation of auxiliary donor and acceptor moieties into the HOMO and/or LUMO positions of the MR skeletons. Consequently, emitters BN-Y (175) and BN-R (176) exhibited bright yellow and red emission at 567 and 624 nm, respectively.^127^ Symmetrical emitters such as BpIC-DPA (177) and BpIC-Cz (178), showed narrow FWHM below 25 nm and high Φ_PL_ in pure green emission, which is beneficial from the synergistic effect of curvilinear indolocarbazole (pIC) donors enhancing the rigidity and *para*-positioned boron donors regulating the FMOs.^128^

**Figure 11:**
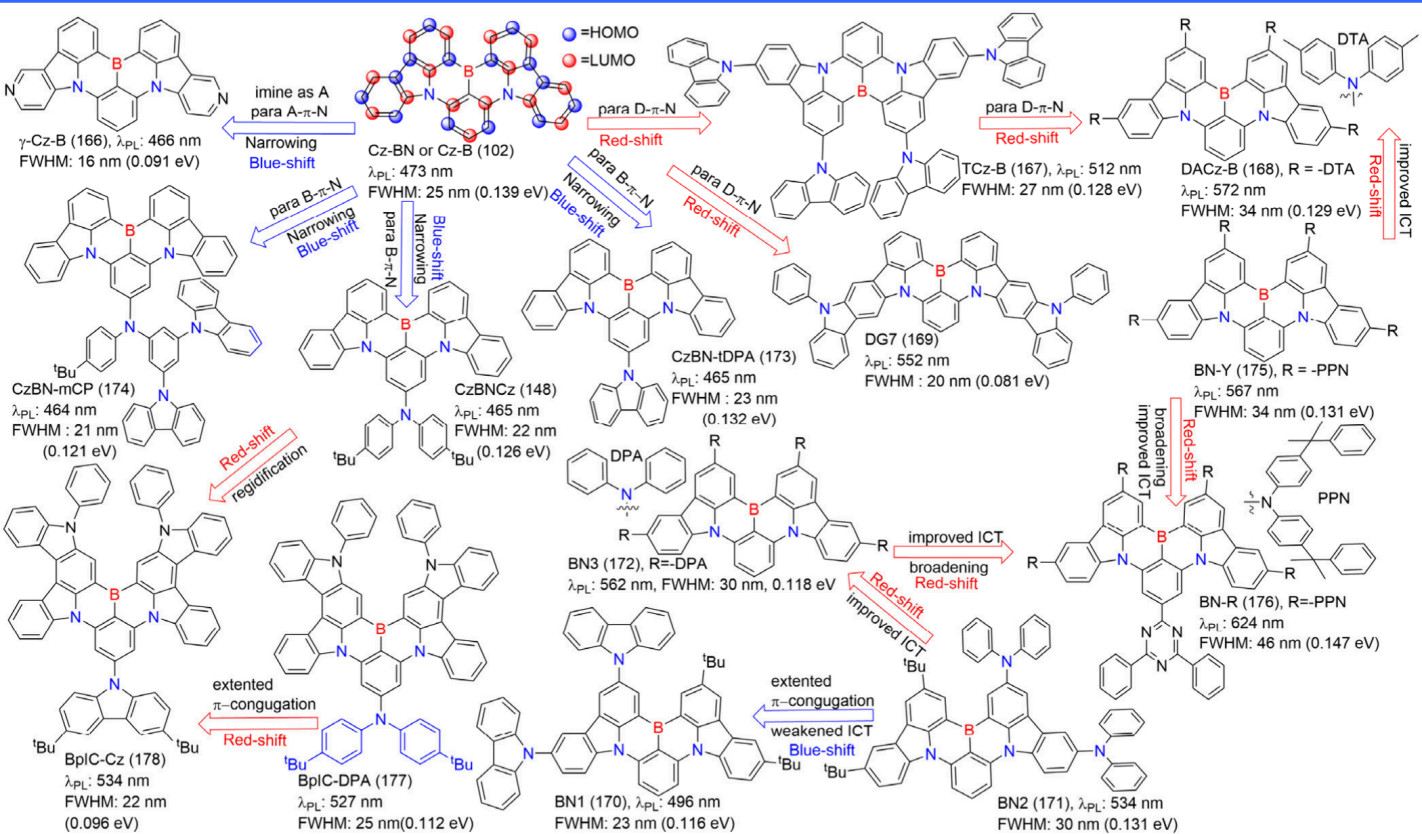
Modulating colors based on analogues of Cz-BN (102).

Duan and co-workers proposed a concept of “decoration strategy at *para*-B position” based on DtBuCzB (5) to tune the emission color of MR emitters.^129^ The derivatives 2F-BN (179), and 3F-BN (180), 4F-BN (181) (see Figure 12), which incorporate peripheral D or A substituents at the *para*-positioned B-substituted phenyl ring in the MR-core, exemplified this approach. This strategy highlights the versatility of MR emitters for color tuning while maintaining narrow emission bandwidths, which are crucial for high-performance optoelectronic applications.^130^ Fluorobenzene, with electron-deficit properties, enhances the conjugation of the skeleton while maintaining the desirable narrow emission bandwidths. Attachment of 1,3,5-triazine and pyrimidine derivatives as acceptors to the *para*-positioned boron atom in DtCzB strengthens the acceptor capacity. The resulting emitters, DtCzB-DPTRZ (185), DtCzB-TPTRZ (184), DtCzB-PPm (182) and DtCzB-CNPm (183) achieved simultaneous bathochromic shifts and narrowband emissions with λ_PL_ values of 521 nm (FWHM = 24 nm), 501 nm (FWHM = 27 nm), 499 nm (FWHM = 25 nm), and 515 nm (FWHM = 36 nm), respectively.^131^ Notably, the key intermediate DtCzB-Bpin (186) provides a versatile platform for constructing a variety of MR emitters via a simple one-step Suzuki-coupling reaction, overcoming the challenges of borylation at *para*-carbon atoms hindered by electron-withdrawing groups. Furthermore, leveraging the exceptional electron-withdrawing capacity of a cyano (CN) group at the LUMO position of BCz-BN (5) induces red-shifted emission. CN-BCz-BN (187) exhibited emission at 496 nm with a narrow FWHM of 21 nm.^132^ By introducing electron-donating groups on both sides of the CN group in CN-BCz-BN (187), CNCz-BNCz (188) demonstrated a red-shifted emission at 581 nm with a relatively small FWHM of 42 nm, attributed to the electron-withdrawing effect of the cyan group, which restricts structural relaxation into a coplanar conformation.^132^

**Figure 12:**
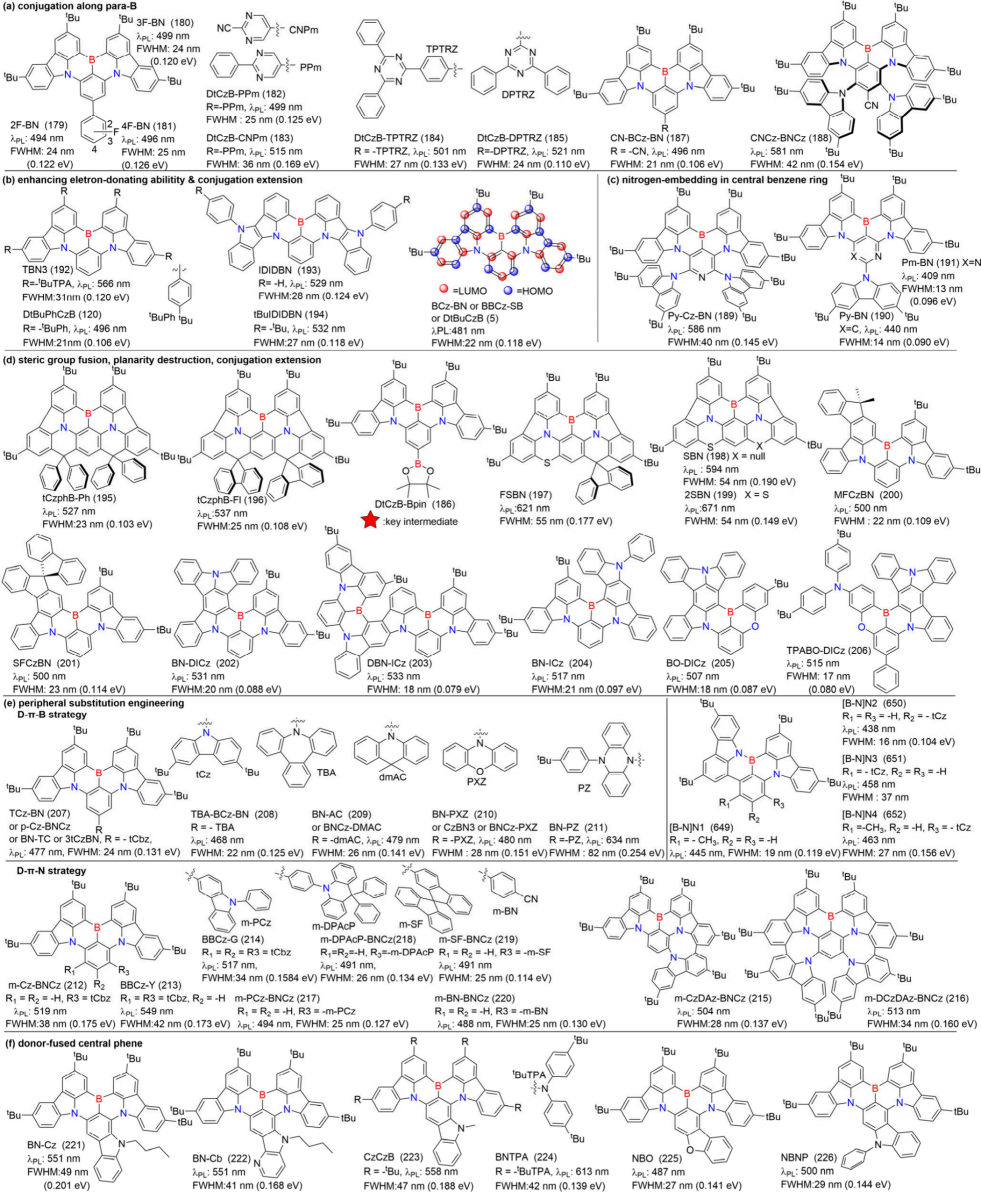
Modulating colors based on analogues of BCz-BN (5).

Nitrogen embedding in the central benzene imparts unique characteristics to MR emitters, distinct from conventional benzene-centered MR emitters, due to the formation of intramolecular hydrogen bonds. For instance, compared to benzene-centered BCz-BN (5), Py-Cz-BN (189), with a central pyridine ring acting as a co-acceptor through steric effects, exhibited a significant spectral red shift, a narrower spectrum, and improved Φ_PL_ due to intramolecular hydrogen bonding.^133^ Two heterocyclic MR-TADF molecules, Py-BN (190) and *Pm*-BN (191), exhibited deep-blue emissions with high Φ_PL_ values of 93% and 94%, and exceptionally narrow FWHM of 14 and 13 nm, respectively. This enhanced performance stems from the stabilization of HOMO energy levels by the nitrogen atoms in the central benzene ring and the formation of intramolecular hydrogen bonds, inducing hypsochromic shifts and spectral narrowing.^134^

Another effective approach involves substituting the *para*-position of the N-located periphery in Cz-BN (102) with t-butyl groups or larger π-conjugated t-butylbenzene units, resulting in red-shifted emissions with narrow FWHMs and concentration-independent spectral features. As shown in Figure 12, DtBuCzB (5) (λ_PL_ = 481 nm, FWHM = 22 nm) and DtBuPhCzB (120) (λ_PL_ = 496 nm, FWHM = 21 nm) demonstrate the utility of peripheral modifications for fine-tuning emission properties while maintaining narrow emission bandwidths.^99^ Benefited from a rigid π-conjugated framework and sterically hindered structure, DtBuCzB (5)^99,135^ (also named BN-Cz,^136,137^ or BBCz-SB^27^) has served as a prototype for cutting-edge emitters. For instance, IDIDBN (193) and tBuIDIDBN (194), which replace the carbazole subunits in the bluish-green BCz-BN (5) skeleton with 5-phenyl-5,10-dihydroindolo­[3,2-*b*]­indole (IDID) and 5-(4-(*tert*-butyl)­phenyl)-5,10-dihydroindolo­[3,2-*b*]­indole (tBuIDID), demonstrated pure green emission at 529 and 532 nm, with CIE coordinates of (0.25, 0.71) and (0.28, 0.70), respectively.^138^

Fusing steric groups onto MR emitters not only extends π-conjugation but also mitigates intermolecular interactions to some extent. For instance, the rigidification of emitters such as tCzphB-Ph (195) and tCzphB-Fl (196) was accomplished by introducing external phenyl groups into the DtBuCzB (5) molecule via bonding with a spirocarbon bridge. These molecules exhibited green emission with a very narrow FWHM of 14 nm and a CIE_*y*_ value of 0.77 in cyclohexane, attributed to the suppression of high-frequency vibration under the synergistic effect of the MR effect and the multiple interlocking strategy^139^ FSBN (197), employing a spiro-carbon-locking and sulfur-embedding strategy to enhance the ICT excited state and the π-conjugation, exhibited saturated red emission with a peak wavelength of 621 nm and a relatively broad FWHM of 55 nm (0.18 eV) in dilute toluene solution.^140^ By modifying the spiro-carbon-locking in FSBN (197), the resulting S-BN (198) and 2S-BN (199) exhibited emission maxima at 594 and 671 nm, respectively. Notably, 2S-BN (199) represents the first example of a single boron deep-red MR emitter.^141^ Emitters such as MFCzBN (200) (λ_PL_ = 500 nm and FWHM = 22 nm) and SFCzBN (201) (λ_PL_ = 500 nm and FWHM = 23 nm) exhibited green emission with narrowband, which were endowed by the fusion of steric groups.^142^ Similarly, BN-DICz (202) and DBN-ICz (203) exhibited emissions in dilute toluene solutions with peak wavelengths ranging from 533 to 542 nm and exceptionally narrow FWHMs of ≤ 20 nm compared to the parent BN-ICz (204). These enhancements are ascribed to the extension of the π-conjugation length, simultaneously increasing the structural rigidity and decreasing the vibrational frequencies associated with transition.^143^ Furthermore, combining the B/O-MR fragment with ICz-MR units facilitated precise tuning of the MR distribution regions, which became localized within the narrowband ICz-MR segments. This approach yielded ultrapure green emissions in BO-DICz (205) and TPABO-DICz (206), both of which exhibited narrow FWHMs of 17 nm.^144^

The implementation of the *para*-positioned D-π-B strategy allows modulation of the balance between locally excited (LE) and charge transfer (CT) states by incorporating peripheral donor (D) units into the BCz-BN (5) backbone. Moderate donor units can reduce the electron-withdrawing effect of the boron atom, resulting in a blue shift of the emission. For example, TCz-BN (207) (also named p-Cz-BNCz,^145^ BN-TC,^146^ 3tCzBN^134^), introduces a carbazole unit at the *para*-position of the boron-substituted phenyl ring, reducing ICT characteristic and exhibiting blue-shifted emission at λ_PL_ = 477 nm with an FWHM of 24 nm.^130^ Additionally, a medium-ring donor, heptagonal tribenzo­[b,d,f]­azepine (TBA), attached to BCz-BN (5) with a unique perpendicular geometry, forms TBA-BCz-BN (208), which showed significantly blue-shifted emission at 468 nm and a decreased Δ*E*_ST_ of 0.14 eV.^147^ TADF emitters such as BN-TC (207), BN-AC (209) (also named BNCz-DMAC^148^), and BN-PXZ (210) (also named CzBN3,^149^ BNCz-PXZ^148^), exhibited narrow emission with predominant LE characteristics, while BN-PZ (211) displayed a broad, red-shifted emission centered at 634 nm with pronounced CT property due to the stronger electron-donating nature of the phenazine unit.^146^

Introducing electron-donating group at the *meta*-carbon position of BCz-BN (5) results in HOMO delocalization across the BCz-BN (5) core and peripheral donor unit. This usually raises the HOMO energy level, resulting in a bathochromic shift in emission compared to the parent BCz-BN (5). For example, m-Cz-BNCz (212) exhibited red-shifted emission at 519 nm with an FWHM of 38 nm. BBCz-Y (213), featuring two tCz units at the *meta*-positions of the boron center, further extends HOMO delocalization and enhances ICT characteristics, significantly red-shifting the emission to 549 nm with a broader FWHM of 42 nm. Compared to m-Cz-BNCz (212) and BBCz-Y (213), m-CzDAz-BNCz (215) and m-DCzDAz-BNCz (216), which incorporate intramolecular covalent bond-locked octagonal rings, exhibited bright light-green and green fluorescence in toluene with maxima at 504 and 513 nm and FWHMs of 28 and 34 nm, respectively.^150^ Building on BBCz-Y (213), BBCz-G (214) incorporated an extra tCz to the *para*-position of the BCz-BN (5) core, exhibiting a hypochromic shifted emission to 517 nm with a narrow FWHM of 34 nm.^27^ Notably, *meta*-positioned tCz has the opposite effect compared to the *para*-positioned tCz, as observed in p-Cz-BNCz (207) (vide supra).^145^ The introduction of peripheral groups via a benzene ring bridge at the *meta*-carbon position of BCz-BN (5) (Figure 12) minimally affects the FMOs distribution, allowing only partial HOMO delocalization onto the benzene ring. This results in modest bathochromic shifts, as exemplified by m-PCz-BNCz (217), m-DPAcP-BNCz (218), m-SF-BNCz (219), and m-BN-BNCz (220).^151^ The [B-N]N covalent system, featuring a B-N embedded bond, induces a blue shift in emission compared to its counterpart BCz-BN (5). By adopting a peripheral substitution engineering strategy, the emission color can be further precisely modulated. For example, [B-N]­N1 (649) to [B-N]­N4 (652), incorporating tCz units at different positions, exhibit blue emissions ranging from 445 to 463 nm with narrow FWHMs.^152^

Fusing donor units into the core ring not only extends conjugation but also tunes the electron-donating strength, thereby effectively modulating the emission color. N-Cz (221) and BN-Cb (222), synthesized via direct polycyclization of the donor group attached to the MR core, resulting in a significant red-shifted emission with bright yellow fluorescence and narrow bandwidth.^153^ Extending the MR core with *para*-positioned N-π-N conjugation, as in CzCzB (223), resulted in bright yellow photoluminescence and electroluminescence with emissions maxima around 560 nm.^154^ In striking contrast, extending π-conjugation with a *para*-positioned N-π-B configuration, as in NBO (225) and NBNP (226), resulted in bathochromic-shifted emissions, though the shifts were less pronounced compared to CzCzBN (223) (Figure 12).^155^ BNTPA (224), designed by integrating secondary electron-donating units and extending the π-skeleton within MR cores, achieved redshift narrowband emission along with an accelerated *k*_RISC_ compared to CzCzB (223) and TBN3 (192).^156^

#### 3.1.2 Modulation Emission Colors of Multiple-Boron Centered MR Emitters

The above section elaborates on the strategy of manipulating ICT strength or π-conjugation to modulate emission colors in one boron-centered MR emitters. The design principle of *para*-positioned D-π-N (*para*-positioned A-π-N) or *para*-positioned A-π-B (*para*-positioned D-π-B) configurations has been widely applied to larger fused PAHs incorporating multiple boron-centered MR emitters. D-π-N-based heterocyclic aromatic hydrocarbons extend conjugation, and minimize ground-to-excited state structural displacement and spectral redshift, while the MR fragment primarily restricts low-frequency vibronic coupling and facilitates narrow-band emission.^157^ These synergistic approaches have broadened the spectrum of emission colors, with ν-DABNA (11)^9^ serving as a milestone prototype for blue and BBCz-R (7)^27^ for red emitters. However, achieving further redshifts, particularly beyond 620 nm, remains a great challenge from viewpoints of design and synthesis, which have been drawing substantial attention. In the synthetic strategy, Takuma Yasuda et al.^27^ proposed an epoch-design principle by substituting traditional D and A moieties in *para*-positioned configurations with B and N atoms, forming *para*-positioned B-π-B and N-π-N moieties. This substitution facilitates the development of larger fused polycyclic π-systems, enhancing D-A strengths and enabling red-shifted MR-TADF emitters (Figure 13). The criss-cross B-π-B/N-π-N configuration improves the electronic coupling between *para*-positioned atoms, constricting the π-bonds on the phenyl-core, narrowing the energy gap, and inducing red-shifted emission. Additionally, the mutually *para*-positioned B- and N atoms also induce a multiresonance effect on the peripheral skeleton, creating the nonbonding orbitals with shallow potential energy surfaces that suppress high-frequency vibrational quenching. For instance, the rigid BBCz-R (7) with its X-shaped configuration of *para*-positioned B-π-B and N-π-N units exhibited an emission peak at 615 nm with an FWHM of 21 nm.^27^ Similarly, Duan and co-workers incorporated congested Cz units as the donors, leading to highly distorted central benzene rings in R-BN (227) and R-TBN (228). This exquisite layout gives rise to shallow potential energy surfaces to bypass the emission quenching governed by exciton-vibration, i.e., the coupling “energy gap law”, enabling efficient deep-red emission at 662 and 692 nm for R-BN (227) and R-TBN (228).^12^ Further tuning of electron-donating strength and π-conjugation extension in *para*-boron-fused PAHs, such as PXZ-R-BN (229) and BCz-R-BN (230), pushed the emission into the NIR region with FWHMs of 49 and 43 nm, respectively.^158^

**Figure 13:**
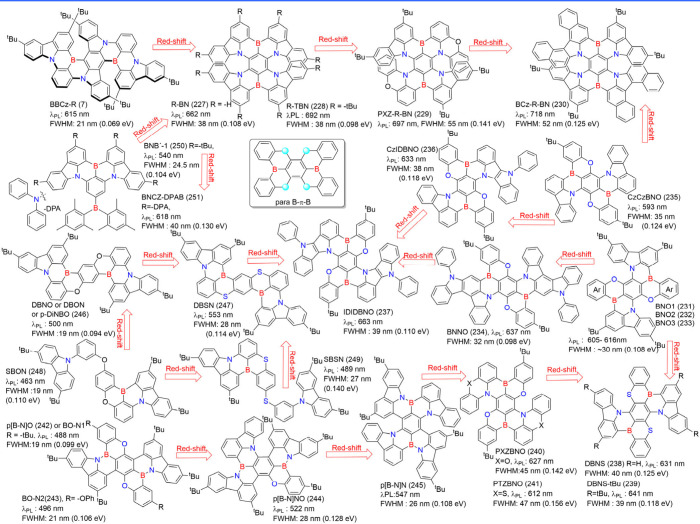
Modulating colors based on the principle of *para*-positioned B-π-B and D-π-D.

Notably, replacing the nitrogen atom with less electron-rich oxygen atoms can lead to the blueshift of the emission peak wavelength. Incorporating *para*-positioned N-π-N, O-π-O, and B-π-B pairs into benzene cores yielded red emitters, namely BNO1 (231), BNO2 (232), and BNO3 (233), with emission peak wavelengths ranging from 605 to 616 nm, which are significantly blue-shifted compared to their prototype R-TBN (228).^136^ However, by leveraging extended π-conjugation, precise red-shift tuning can be achieved. A prime example is BNNO (234), which met BT.2020 red coordinates of (0.708, 0.292) with high efficiency and an ultralong lifetime in the device by fusing indolocarbazole segments into a B/O-embedded skeleton.^159^ CzCzBNO (235), CzIDBNO (236), and IDIDBNO (237), showcased emissions from orange-red to deep red by simultaneously regulating the π-conjugation and electron-donating strengths.^160^ Substitution of oxygen in DBNS (238) and DBNS-tBu (239) with sulfur enhanced *k*_RISC_ due to the heavy atom effect, but resulted in a larger FWHM.^161^ PXZBNO (240) and PTZBNO (241), incorporating stronger electron-donating like phenoxazine and phenothiazine, demonstrated pure-red emission with emission maxima reaching up to 627 nm and a small FWHM of 45 nm.^162^

Building on the *para*-positioned B-π-B structures, stepwise modifications to the resonance interactions between heteroatoms and boron have yielded a wide range of emission colors, spanning from deep blue to yellow-green. Emitters like p­[B-N]O (242) (also named BO-N1 ^163^), p­[B-N]­NO (244), p­[B-N]N (245), and BO-N2 (243) demonstrate this versatility, exhibiting impressive narrow FWHMs of 19–28 nm.^164^ DBON (246)^165^ (or DBNO^166^ or p-DiNBO^167^) and DBSN (247),^165^ featuring a boron-chalcogen-nitrogen-embedded MR-skeleton similar to the configuration of BBCz-R (7), exhibited a red shift in photoluminescence compared to their single boron center counterparts SBON (248) and SBSN (249), respectively.

Furthermore, the heavier S-based DBSN (247) showed a red-shifted emission compared to the O-based DBON (246) due to the stronger electron-donating effect.^165,166^

A *para*-positioned B-π-B configuration by a flexible boron atom and an embedded boron atom formed also demonstrates an enhanced acceptor ability. For instance, BNB′-1 (250) exhibited remarkable electroluminescence with a peak at 540 nm in both solution-processed and vacuum-processed OLEDs.^168^ Further structural modifications to BNB′-1 (250) by introducing flexible aromatic amino moieties at the periphery led to the development of BNCZ-DPAB (251), a dramatically red-shifted narrowband emitter, which achieved pure-red electroluminescence in solution-processed OLEDs, with CIE coordinates of (0.697, 0.306), closely matching the BT.2020 red color standard.^169^

In 2018, Takuji Hatakeyama et al. employed the *meta*-positioned B-π-B/N-π-N framework to design a series of MR emitters, including B2 (54) to B4 (56). B2 (54), featuring three N-units as peripheral donors, exhibited blue-shifted emission at 455 nm in PMMA film compared to ν-DABNA (11). B3 (55), which incorporated an additional B moiety at the *para*-position of the N-center, showed a further hypsochromic shifted emission to 441 nm due to the reduced donating strength. B4 (56), with quadruple borylation and a hydroxy synergistic effect that enhanced electron-withdrawing strength, revealed a red shift compared to B3 (55) (see Figure 14).^60^ DPA2MN2B (252), SAC2MN2B (253), and Cz2MN2B (254), which incorporated a “locking-ring” to confine electronic and structural distortion based on the peripheral rotor SAC2MN1B (255), effectively narrowed the FWHM while retaining blue emission (see Figure 14).

**Figure 14:**
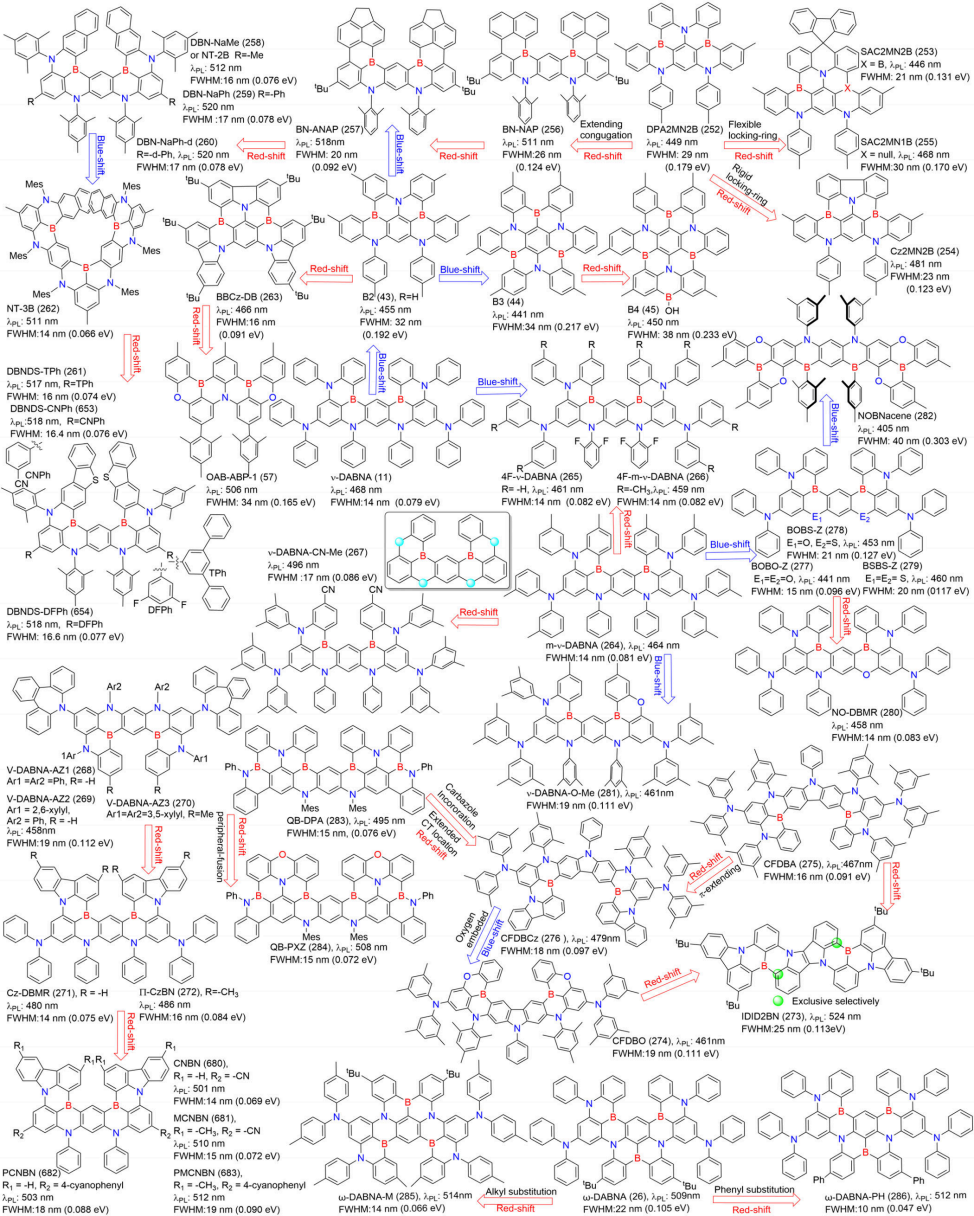
MR emitters based on the *meta*-positioned boron framework.

Notably, these MR emitters with naphthalene units exhibit nearly negligible or no potential for the RISC process of triplet excitons due to the low T_1_ energy level of naphthalene, leading to increased Δ*E*_ST_. SAC2MN2B (253) achieved state-of-the-art device performance in a sensitizer-free OLED.^170^ BN-NAP (256) and BN-ANAP (257) demonstrated a simple yet effective strategy to red-shift emissions while maintaining a narrow FWHM by fusing the *meta*-positioned double boron framework with two naphthalene moieties.^171^ Compounds DBN-NaMe (also named NT-2B^172^) (258), DBN-NaPh (259), and DBN-NaPh-d (260), designed by coordinating boron with naphthalene units and incorporating methyl, phenyl, or perdeuterated phenyl groups at the *para*-position of boron, exhibited green emission with peaks at 512–521 nm and narrow FWHMs of 16–17 nm in a toluene solution. Notably, devices based on the partially deuterated DBN-NaPh-d (260) achieved an EQ_max_ of 35.2% with a lifetime (LT_50_) exceeding 3000 h at an initial luminance of 1000 cd m^–2^.^173^ NT-2B (258) and NT-3B (262), which incorporated two or three boron/nitrogen-embedded [4]­helicene subunits with naphthalene, emitted at 510 and 511 nm, respectively, in dilute toluene solution, with exceptionally narrow FWHM values of 15 and 14 nm.^172^ DBNDS-TPh (261), DBNDS-CNPh (653), and DBNDS-DFPh (654) (Figure 14) feature a dibenzo­[b,d]­thiophene unit, which simultaneously reduces the bandgap and elevates the triplet state energy, while different *para*-positioned boron substituents further deepen both HOMO and LUMO levels. As a result, the CIE coordinates of DBNDS-TPh (261), DBNDS-DFPh (654), and DBNDS-CNPh (653) first achieved a CIE_*y*_ value of 0.77 in a dilute toluene solution.^41^

BBCz-DB (263), which adopted a similar B-N layout to B3 (55) but incorporated larger conjugation with tCz at the periphery, exhibited a red-shifted emission at 466 nm and a narrow FWHM of 16 nm.^27^ OAB-ABP-1 (57), featuring an extended π-skeleton consisting of ADBNA-Me-MeS (8) and DOBNA (52) substructures, demonstrated attractive photophysical properties with an emission peak at 506 nm and an FWHM of 34 nm in the PMMA film. This emission was attributed to π-resonance elongation induced by the interplay of boron, nitrogen, and oxygen atoms. Notably, OAB-ABP-1 (57) represents the first solution-processed OLED to combine high color purity and efficiency, with an EQE_max_ of up to 21.8% and an FWHM of 33 nm.^61^

ν-DABNA (11) consisting of two fused DABNA-1 (1) units in a *para*-positioned B-π-N and *meta*-positioned B-π-B/N-π-N framework revealed an ultranarrow FWHM comparable to well-defined LEDs such as gallium nitrides (micro-LEDs) and CdS/ZnS or CdSe/ZnS quantum dots (QD-LEDs).^9^ The reported ν-DABNA (11) demonstrated blue electroluminescence (EL) emission at 469 nm with a CIE_*y*_ coordinate of 0.1161—slightly deviating from the NTSC standard for blue color (CIE_(*x*,*y*)_ of (0.14, 0.08)). To achieve a hypsochromic shift and meet the NTSC requirement (Figure 14), researchers introduced weak electron-donating methyl groups at boron *para*-positions to raise the LUMO energy level while adding electronegative fluorine atoms at the nitrogen *ortho*-positions to lower the HOMO energy level.^174^ These synergistic effects enlarged the optical bandgap, resulting in hypsochromic shifts in derivatives such as m-ν-DABNA (264), 4F-ν-DABNA (265), and 4F-m-ν-DABNA (266), with an emission peak wavelength at 464, 461, and 459 nm, respectively.^175^ Introducing an electron-withdrawing cyano group into the *para*-boron LUMO distribution induces a bathochromic shift without compromising the color purity. As a result, ν-DABNA-CN-Me (267) gave pure green emission with an FWHM of 17 nm.^42^ Given the substantial molecular weight of ν-DABNA (11), further added substituents pose challenges for vacuum evaporation. Thus, ν-DABNA-Az1 (268), ν-DABNA-Az2 (269), and ν-DABNA-Az3 (270) incorporated a stronger donor unit (azepine) instead of diphenylamine, confirmed enhanced stabilization, and achieved ultrapure deep-blue emission by altering LUMO distributions.^176^

Additionally, symmetrical Cz-DBMR (271) and Π-CzBN (272), which replaced diphenylamine units in the ν-DABNA (11) core with carbazole homologues, enhanced molecular rigidity and charge-transfer localization. This modification resulted in a bathochromic shifted emission to approximately 480 nm, further improving the MR effects.^177,178^ CNBN (680) and MCNBN (681) (Figure 14) exhibited sharp green emission with extremely narrow FWHMs of only 14 nm/0.066 eV and 15 nm/0.071 eV by synergistic rigid π-extension and cyano-substitution. While PCNBN (682) and PMCNBN (683), replacing cyano units with 4-cyanophenyl groups, displayed emission maximum similar to CNBN (680) and MCNBN (681) but increased FWHMs.^179^ IDID2BN (273), featuring an indolo­[3,2-*b*]­indole (32bID) segment as a multinitrogen π-extended bridge, displayed high-efficiency green emission.^180^ CFDBO (274), CFDBA (275), and CFDBCz (276), incorporating a carbazole-fused dual-boron MR-TADF framework, displayed ultranarrowband blue emission with peaks ranging from 452 to 479 nm and slender FWHM of only 16–18 nm in dilute toluene solutions.^181^

The systematic implementation of chalcogen (oxygen and sulfur) atoms at the *meta*-positioned B sites fine-tuned the resonant effect and restricted HOMO π-conjugation rather than that of the LUMO, thereby inducing a hypsochromic shift compared to the parent nucleus. For example, the exquisite combination of boron, nitrogen, oxygen, and sulfur heteroatoms in fused PAHs induced a multiple-resonant effect among B-N, B-O, and B-S. Consequently, BOBO-Z (277), BOBS-Z (278), and BSBS-Z (279) achieved ultrapure blue emissions at 441, 453, and 460 nm, respectively.^182^ It is worth noting that chalcogen atoms not only finely modulate the emission color while maintaining a narrow bandwidth, but also facilitate the spin-flipping rates between the lower-lying excited singlet and triplet states by strengthening SOC (vide infra). The unsymmetrical NO-DBMR (280), which replaces a centered nitrogen (N) atom in the ν-DABNA (11) core with an oxygen atom, exhibited a hypsochromic-shifted emission at 447 nm without disturbing the FWHM of 14 nm.^177^ Implementing a weak electron-donating oxygen (O) atom instead of an outside nitrogen (N) atom in the ν-DABNA (11) core induces a partial B-O resonance effect. This modification is evident in ν-DABNA-O-Me (281), which shows a slight hypsochromic shift (∼3 nm) in its emission peak compared to m-ν-DABNA (264). Furthermore, the introduction of oxygen at the *meta*-positioned B atom suppressed efficiency roll-off and prolonged device lifetime compared to that of ν-DABNA (11), underscoring the critical role of oxygen incorporation in design strategy.^183^ NOBNacene (282), which combines DOBNA (52) and α-3BNMes (68) (cf. Figure 3) subunits into a framework of nine annulated six-membered rings with a linearly extended ladder-type configuration, exhibited efficiently a narrow deep blue emission with a peak at 410 nm and an FWHM of 38 nm.^184^ Quadruple borylated MR-TADF emitters, QB-PXZ (284), developed by combining π-extension and peripheral locking approaches, exhibited red-shifted pure-green emission with a narrow FWHM of 15 nm, compared to its π-extension-only counterpart QB-DPA (283).^185^

In addition to the linear-type molecular configuration, a special scaffold based on *meta*-positioned B-π-B/N-π-N delocalization has been also explored. For instance, ω-DABNA (26), featuring an omega-shaped MR skeleton with three boron atoms and four nitrogen atoms, demonstrated narrowband green emission with an FWHM of 22 nm.^38^ Furthermore, through π-conjugation extension or enhanced donor strength relative to ω-DABNA (26), ω-DABNA-M (285), and ω-DABNA-PH (286) showed significantly red-shifted emission, aligning closely with NTSC and BT.2020 standards (Figure 14).^186^

Extending the π-skeletons of MR emitters provides a practical way to modulate their optoelectronic properties (see Figure 15). Theoretical calculations show that the extended conjugation lowers the FMOs and decreases the energy gap, causing an emission red shift. For instance, B4N6-Me (287), which combines the hybrid *para*-positioned B-π-N and B-π-B/N-π-N patterns, simultaneously realized long-wavelength emission with ultranarrow FWHM in dilute toluene (see Figure 15a).^187^ Notably, m-DiNBO (289) and p-DiNBO (246), as dimerized derivatives of NBO (288) with *meta*- and *para*-positioned B-π-B patterns, respectively, exhibited red-shifted emission with narrower bandwidth and enhanced horizontal molecular orientation due to their larger planar molecular structure.^167^ Among these, p-DiNBO (246) demonstrates a larger redshift and higher oscillator strength compared to m-DiNBO (289). This difference arises from their FMOs configuration, i.e., the central π-core in p-DiNBO (246) versus a nonbonding character in m-DiNBO (289). Similarly, two isomeric compounds, p-CzB (290) and m-CzB (291), employing a dimerization strategy with enlarged π-extension through different linking positions, preserved intrinsic MR characteristics while achieving noticeable bathochromic emission-band shifts without compromising color purity (Figure 15b).^188^ Furthermore, sky-blue emitters such as Cz-DBCz (292), Cz-DBTPA (293), and PhO-DBCz (294) utilize B-N/B-O fusion lockers to extend the π-conjugation and alleviate the steric hindrance. This structural enhancement improves the planarity of their skeletons, suppressing high-frequency stretching and scissoring vibrations, which results in ultranarrowband emissions with small FWHM values of 17–18 nm—significantly narrower than their merely conjugated aromatic counterparts.^189^ Na-dBN (296), compared to Na-sBN (295), exhibited a red-shifted emission and narrow bandwidth due to the distinct conjugated bonding characteristics of naphthalene and fused B/N-doped MR units (Figure 15c).^190^

**Figure 15:**
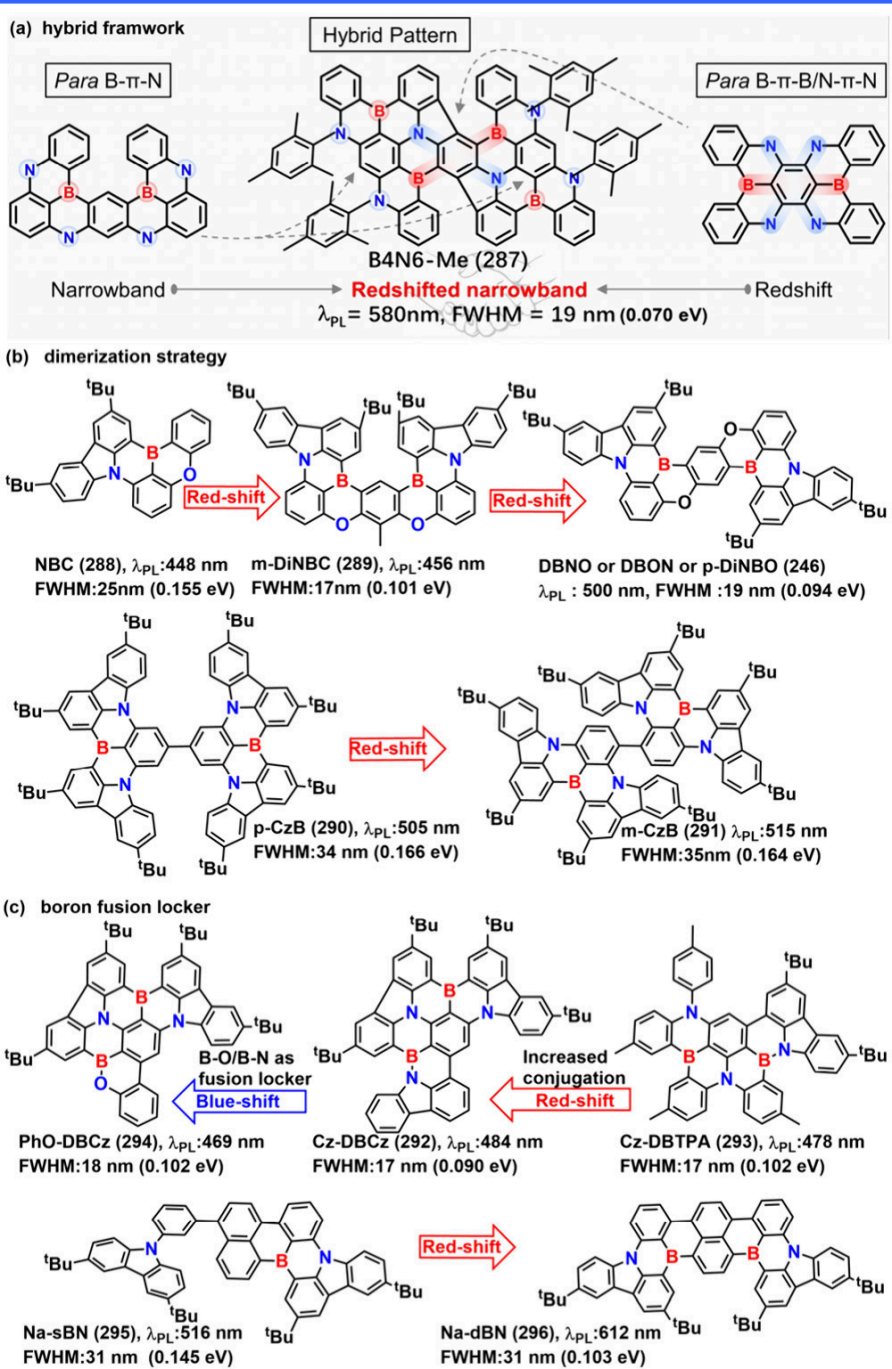
MR emitters based on (a) the hybrid double boron framework, (b) dimerization strategy, and (c) boron fusion locker.^187^ Reproduced with permission from ref 187. Copyright, 2023, John Wiley and Sons.

Overall, three mainstream strategies have been identified for red-shifting MR emissions while preserving a narrow FWHM in B/N-type MR-TADF emitters: (i) Peripheral decoration: Substituting bulky D/A moieties around an MR unit enhances the ICT effect while maintaining SRCT dominance over LRCT.^123,124,127^ (ii) π-conjugation extension: Fusing D/A fragments into the MR-unit retains nonbonding characteristics.^100,105,154^ (iii) *Para* B-π-B/N-π-N modulation: Incorporating *para* B-π-B/N-π-N into a rigid backbone enables a sufficient redshift by substantially enhancing the electron-withdrawing and donating strength through delocalizing HOMO and LUMO wave functions.^12,27,160^

A notable breakthrough in enhancing the ICT character without compromising narrow FWHMs is the development of an *ortho*-positioned diboron compound (see Figure 16). For instance, the tetraazacyclophane-based architecture HBN (656), which incorporates *ortho*-positioned diboron atoms, demonstrates a remarkable ability to enhance ICT through the strategic positioning of boron and nitrogen atoms. This design resulted in a maximum emission at 572 nm with an extraordinarily narrow FWHM of 17 nm (0.064 eV). Notably, this represents a significant 165 nm redshift in the emission spectrum compared to its precursor, H-tetraazacyclophane (655), which exhibited a broader emission peak at 407 nm.^191^ Another approach employing multiple boron atoms enhancing ICT character is the proof-of-concept B-N “core–shell” strategy. In this design, Δ-DABNA-TB (298) incorporates a single boron atom into the center of deep-blue n-DABNA-O2-TB (297). This modification compresses the electron density, stabilizes the LUMO energy level, and induces LRCT between the B core and the electron-donating shell fragments. Consequently, a profound bathochromic shift was achieved, from 447 nm in DABNA-O2-TB (297) to 624 nm (∼0.8 eV) in Δ-DABNA-TB (298), while maintaining a narrow FWHM of 0.10 eV.^192^ These examples are reminiscent of the strategies employed in well-known diboron-based TADF compounds, CzDBA (657) and tBuCzDBA (658) (Figure 16c).^193^ The approaches used to modulate emission colors in state-of-the-art conventional TADF emitters may also apply to MR emitters.

**Figure 16:**
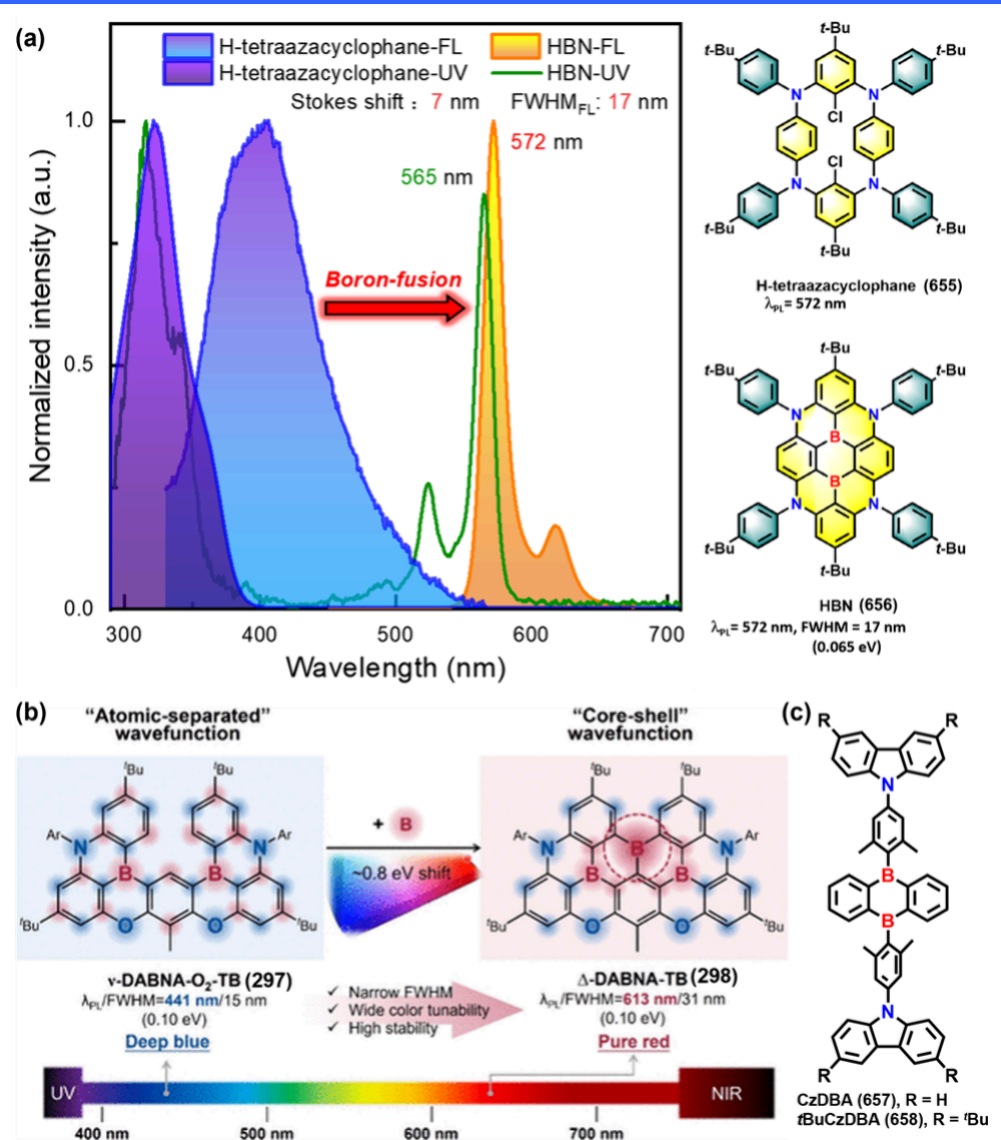
(a) UV/vis absorption and fluorescence spectra of H-tetraazacyclophane (655) and HBN (656) in toluene solutions.^191^ (b) Δ-DABNA-TB (298) achieving a wide range of wavelength red-shift via B-doping “core–shell” strategy.^192^ (c) Diboron-based TADF compounds CzDBA (657) and tBuCzDBA (658). Adapted from refs 191, 192 . Reproduced with permission from ref 191 (copyright, 2025, John Wiley and Sons) and ref 192 (copyright, 2024, American Chemical Society).

### 3.2 Modulation Emission Colors of Nitrogen/Carbonyl-Type MR Emitters

One fundamental principle among the strategies for modulating B/N MR emitter emission is to extend conjugation while preserving the desired MR-TADF characteristics. Furthermore, this strategy is also applicable to N/-CO-type MR emitters (Figure 17).

**Figure 17:**
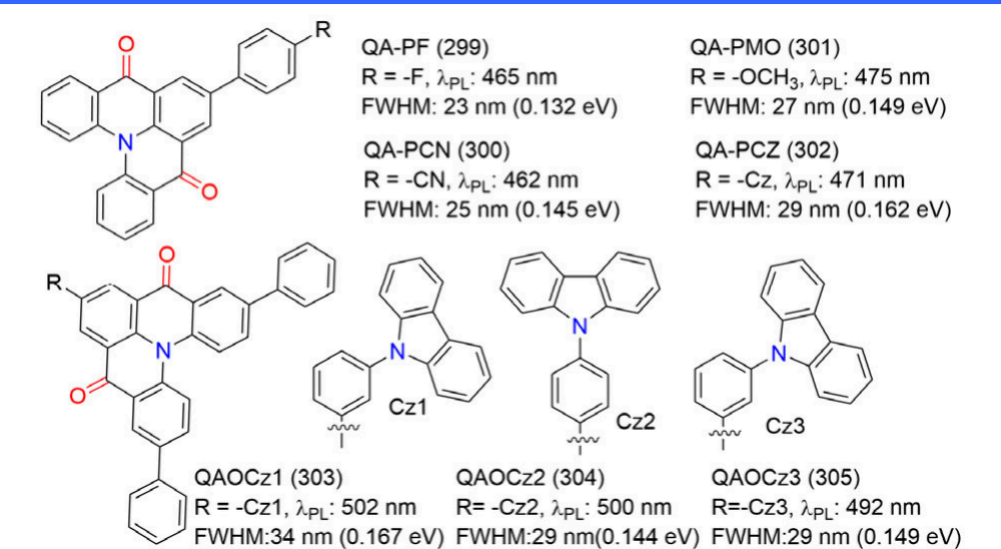
Modulating colors based on QAO (2) via single bond-linked phenyl moieties.

First, introducing single bond-linked phenyl moieties into the QAO (2) core effectively modulates the emission color of the resulting emitters. For example, QA-PF (299) and QA-PCN (300), incorporating fluorophenyl and benzonitrile as peripheral electron-withdrawing moieties, exhibited hypsochromically shifted emission compared to QAO (2). Conversely, QA-PMO (301) and QA-PCZ (302), featuring methoxyphenyl and phenylcarbazole as peripheral electron-donating groups, showed bathochromically shifted emission with narrow FWHMs.^194^ Moreover, weakening the D-A interaction between the substituents and the MR core via a spacer group is critical for maintaining a small FWHM. For instance, QAOCz1 (303), QAOCz2 (304), and QAOCz3 (305) demonstrated red-shifted emissions and narrow FWHMs by strategically adjusting the substitution site to systematically weaken the D-A interaction and hence enhance the molecular rigidity.^194,195^

Second, extending π-conjugation through strategies such as dimerization or fusing PAHs segments provides a feasible approach to emission color modulation (Figure 18). DdiKTa (306), synthesized by simply dimerizing the monomer emitter DiKTa (2), retained the MR-TADF photophysical properties of its monomeric counterpart DiKTa (2). The weak electronic coupling between dual DiKTa (2) fragments in the twisted configuration of DdiKTa (306) resulted in a red-shifted emission and reduced the OLEDs doping concentration needed to mitigate aggregation. OLEDs based on DdiKTa (306) exhibited a λ_max_ of 500 nm, achieving an EQE_max_ of 19% at a relatively high doping concentration of 9 wt % DdiKTa (306) in DPEPO.^196^ Similarly, DDiKTa-A (307), derived from the dimerization of the sky-blue emitter DiKTa (2) via a central aniline bridge, emitted with a λ_max_ of 562 nm and an FWHM of 75 nm in 2 wt % doped mCP films.^197^ By amalgamating distinct SRCT characteristics of MR-cores into a single molecular entity, an extended conjugation emitter can achieve both red-shifted and narrow emission simultaneously. For example, BOQAO (310), which integrates two MR-TADF cores (tBuBO (308) and tBuQAO (309)), exhibited a slightly red-shifted emission (7 nm) compared to the parent emitter tBuQAO (309). Identified as a typical D-A-type TADF emitter, BOQAO (310) maintained a narrow FWHM due to the weak LRCT from tBuBO (308) to tBuQAO (309), as supported by the TD-DFT simulation.^198^ In other cases, structural isomerism and rigid molecular frameworks play key roles in modulating emission color. Sym-DiDiKTa (311) and Asym-DiDiKTa (312), featuring *para*-positioned C=O-π-C=O and N-π-N frameworks with positional *t*-butyl isomerism, displayed green-yellow electroluminescence maxima at 543 and 544 nm, respectively.^199^ Similarly, SS-DAO (39), which incorporated two acridone units within a sterically protected 11-ring fused core skeleton, exhibited red-shifted green emission compared to 10-ring S-DAO (313) and their precursor 2AcPh (659) (also named pTIAO^200^).^48,201^ As shown in Figure 18, axially symmetric mTIAO (660), with the *meta*-oriented N-π-N framework, exhibited a further hypsochromic shift to deep blue emission. In contrast, centrosymmetric pTIQA (661) (also known as NCON-TB^202^) and NCON-Mes (662), featuring *para*-N-π-N and *para*-C=O-π-C=O frameworks, exhibited a red shift to green emission.^200,202^ Additionally, incorporating spiro structures into MR scaffolds provides an intramolecular locking mechanism that modulates emission color while suppressing undesirable vibrations of peripheral heterocycles. For instance, sulfur-based SpiroS-QAO (314) exhibited a relatively narrowband green emission peak at 494 nm with an FWHM of 43 nm, whereas the FWHM of the sulfone-based SpiroSO2-QAO (315) was further sharpened to 32 nm. By contrast, SpiroO-QAO (316) and SpiroOSO2-QAO (317) showed nearly identical photophysical properties, as the sulfur valence changes occurred in the nonconjugated spiro structure, far from the emission core.^203^

**Figure 18:**
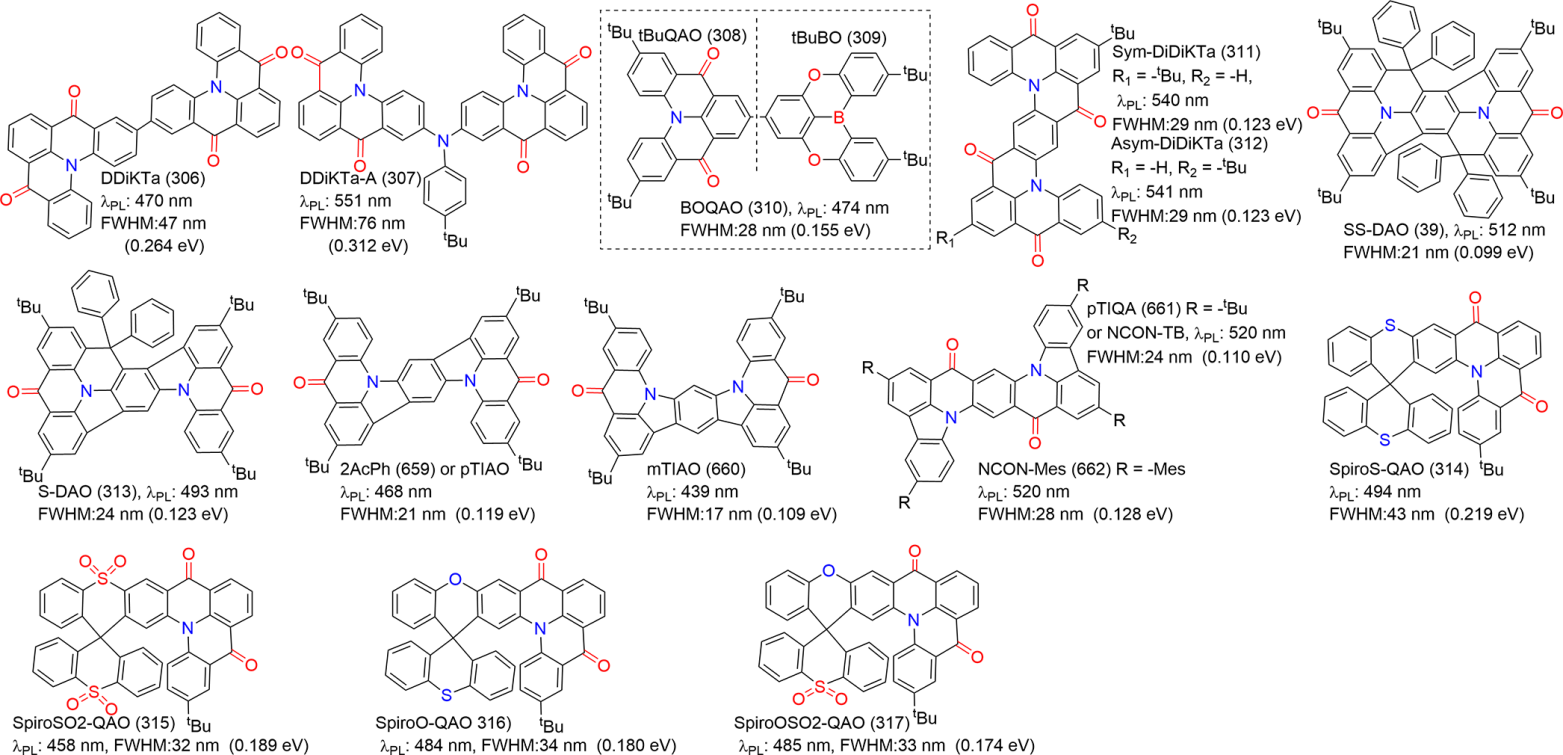
Modulating colors via dimerization or fusing PAHs segments.

Third, peripheral decoration of the MR-core with varying electron-donating abilities and numbers of donor segments represents another effective strategy for modulating emission colors. For instance, analogs such as Cz-Ph-DiKTa (318), Cz-DiKTa (319) (also known as QAD-Cz^204^), 3Cz-DiKTa (320), 3TPA-DiKTa (321), 3DPA-DiKTa (322), QAD-2Cz (323), and QAD-mTDPA (324) utilized the same prototypical MR core but incorporated donors with different electron-donating abilities. These emitters exhibited narrow emission bands spanning a wide color range from blue to red, due to their dominant SRCT states Conversely, counterparts like TMCzDiKTa (325), DMAC-DiKTa (326), 3TMCz-DiKTa (327), and 3DMAC-DiKTa (328), featuring LRCT states, displayed broader emissions (see Figure 19).^205,206^ These results demonstrate that manipulating the nature and numbers of donor groups on a central MR core is a promising strategy for color modulation.^205^ Emitters adorned with weak donors tend to retain SRCT characteristics, leading to red-shifted emissions with narrow FWHMs.

**Figure 19:**
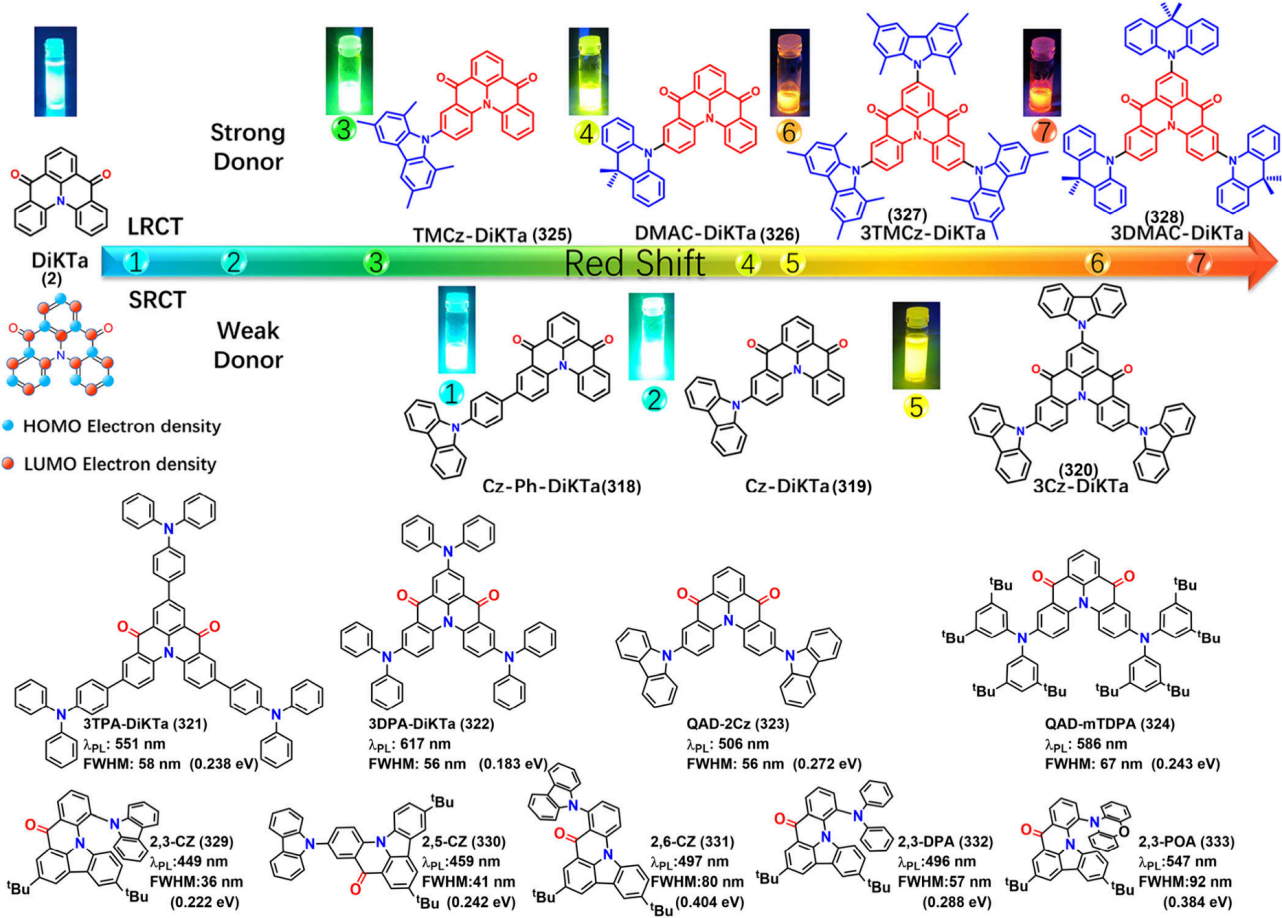
Modulating colors via peripheral decoration of MR-core with varying donor segments. Permission from ref 205. Reproduced with permission from ref 205. Copyright, 2022, American Chemical Society.

Conversely, strong donor influences are anticipated to transform the emitters into conventional D-A-type TADF, accompanied by structural relaxation and consequent spectral broadening. This tendency is evident in nitrogen/single carbonyl-based MR emitters. Molecules such as 2,3-CZ (329), 2,5-CZ (330), 2,6-CZ (331), 2,3-DPA (332), and 2,3-POA (333), which feature different ancillary donors within the fused carbazole/carbonyl skeletons, demonstrated emissions tunability from blue to yellow-green.^207^ Introducing donor segments to the MR core can impart the resultant emitters with hybridization of MR and intersegmental charge-transfer characteristics. However, careful design is required to balance emission color and bandwidth, especially for MR cores with strong electron-withdrawing properties, like TOAT (74) (see Figure 20). Specific emitters such as mBDPA-TOAT (334) and pBDPA-TOAT (335) were synthesized to examine whether MR or conventional TADF characteristics dominate. Mild electron-donating abilities lower the energy levels, enabling red emission while preserving MR-dominated FMOs transition. In contrast, DMACTOAT (336) is dominated by LRCT transitions in the excited state, as evident by the separated FMOs and large Stokes shifts due to the strong electron-donating effect.^208^ Notably, a series of TOAT (74) derivatives (TOAT-1 (337) to TOAT-5 (341)) highlighted the superiority of symmetrical substitutions over asymmetrical ones for controlling color bandwidth. Asymmetrical donor units in TOAT-1 (337) to TOAT-4 (340) disrupt molecular symmetry, resulting in green to greenish-yellow, or even yellow emission with broad spectral widths (70–95 nm). In contrast, the symmetrical TOAT-5 (341), modified with three diphenylamine units, retained distinct FMOs compared to its asymmetrical counterparts. This led to an orange emission with a narrow FWHM of 45 nm, indicating that the electronic transition primarily originated from the MR effect.^209^

**Figure 20:**
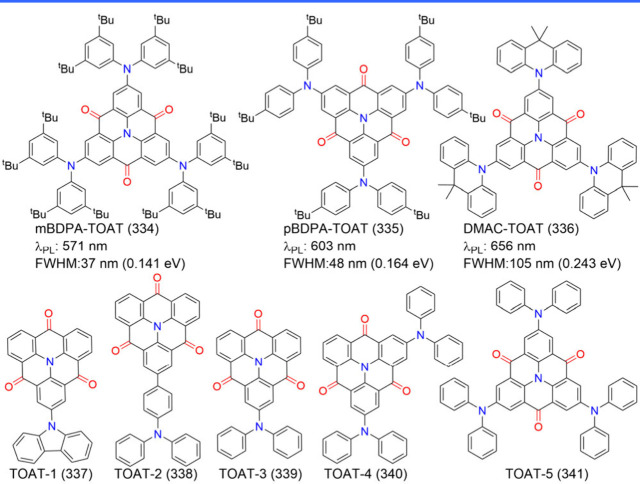
Modulating colors based on TOAT (74) backbone.

### 3.3 Modulation Emission Colors of N-PAHs-Type MR Emitters

N-PAHs incorporating indolocarbazole (ICZ) fusions have garnered significant attention for their narrowband emission, particularly efficient deep-blue emission with CIE_*y*_ < 0.1, owing to their rigid planar structure and unique SRCT characteristics. ICZ (342), a common core for N-PAHs-based MR emitters, exhibited a pure violet emission peak at 374 nm and an FWHM of 21 nm in a toluene solution.^210^ Various approaches have been explored to modulate the emission colors of such MR emitters into the desired visible spectrum (see Figure 21). One promising strategy involves introducing different donors into the unconventional N-PAHs acceptor without sacrificing narrowband emission. For instance, ICZAc (343) and ICZDAc (344), synthesized by attaching stronger donors such as acridine derivatives to ICZ (342) moieties, exhibited efficient TADF behavior with emissions at 454 and 462 nm, respectively. Conversely, adding a weaker donor, such as carbazolylcarbazole, resulted in ICZCz (345) displaying a purple emission at 416 nm, notably without exhibiting TADF properties.^211^ Fluorene-indolocarbazole hybrid chromophore IDCz (82) exhibited emissions at 431 nm with a vibronic sideband, attributed to an enlarged π-conjugation that emphasizes π-π* transition and delocalization.^77^ Decorating IDCz (82) with auxochromophores, such as mono- or di-DPA, i.e., IDCz-DPA (346) and IDCz-2DPA (347), revealed red-shifted emission and demonstrated high brightness with long device lifetime in OLEDs.^77^

**Figure 21:**
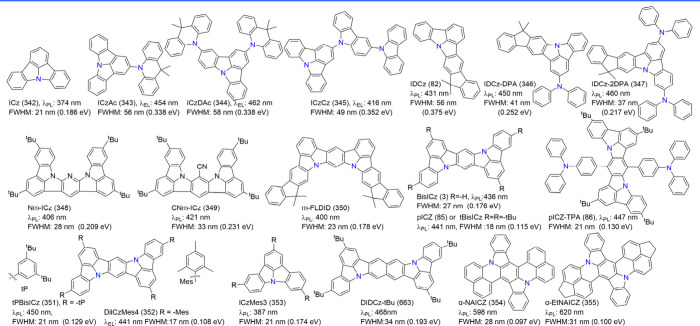
Modulating colors based on N-PAHs-type MR emitters.

Incorporating *meta*-oriented nitrogen distribution in tDIDCz (3) moiety triggers SRCT, effectively suppressing the vibrational reorganization energy while extending π-conjugation, hence resulting in a hypsochromic shift. By replacing the central phenyl unit of the nitrogen-based violet MR skeleton in tDIDCz (3) with pyridine or benzonitrile units to introduce weak ICT, derivatives like Nm-ICz (348) and CNm-ICz (349) achieved bathochromic-shifted fluorescence by ∼10 nm and ∼25 nm, respectively, while retaining narrow FWHMs.^212^ m-FLDID (350), a bis-fusion of two IDCz (82),^77^ exhibited emission at 404 nm with an FWHM of 22 nm in low-doped films.^213^

Deep blue emitters, namely pICz (85) and pICz-TPA (86), displayed red-shifted emission compared to ICZ (342), with peaks at 441 and 447 nm and FWHMs of only 18 and 21 nm, respectively, benefiting from enhanced electronic coupling and decreased emitting energy gap through *para*-positioned nitrogen atoms in bis-ICZ subunits.^79^ Although pICz (85) (also known as tBisICz) lacked TADF properties, its analog tPBisICz (351), featuring attached blocking groups, demonstrated efficient triplet-to-singlet crossover aided by resonant spin-vibronic coupling (SVC).^214^ Expanding π-conjugation in N-PAHs while retaining narrowband emission often induces a bathochromic shift. For instance, DiICzMes_4_ (352), incorporating mesityl groups and a *para*-positioned N-π-N configuration, exhibited a red-shifted emission at 441 nm with enhanced Φ_PL_ and reduced Δ*E*_ST_, allowing for optical detection of RISC. This is in sharp contrast to ICZ (342) (374 nm) and ICZMes_3_ (353) (387 nm), which lack TADF properties.^210^ DIDCz-tBu (663) (Figure 21) tuned the emission color toward the pure blue region while suppressing shoulder emission peaks by extending the π-conjugation of the N-π-N bridge.^215^ Furthermore, α-NAICZ (354) and α-EtNAICZ (355), incorporating homogeneous hexatomic rings into nitrogen-embedded MR skeletons, exhibited emissions at 598 and 620 nm with narrow FWHMs of 28 and 31 nm, respectively, due to the retention of nonbonding character.^216^

With significant advancements, both B/N-type and N/-CO- or even N-PAHs-based MR emitters have achieved full visible spectrum coverage. Some green and red emitters have met practical application standards, while deep-blue MR emitters show the potential to replace existing counterparts.

## 4 Suppression of Aggregation

Undoubtedly, MR-TADF emitters possess unique characteristics, such as ultrapure emission and high efficiency, particularly at low concentrations. However, a common challenge for most rigid MR emitters is their susceptibility to severe aggregation-caused quenching (ACQ). This issue arises from their highly planar configurations, which promote π-π stacking, leading to doping sensitivity and compromised device performance.

To address ACQ, various strategies have been developed to minimize undesired aggregation and excimer emissions while maintaining color purity, making MR-emitter-based OLEDs increasingly viable for practical applications. One effective method is the attachment of bulky substituents to the MR core, which increases intermolecular distances and inhibits interchromophore interactions (see Figure 22). Moreover, the incorporation of electron-rich substituents enables the resulting emitters to exhibit CT properties in higher triplet excited states, which can be thermally accessible. This promotes denser state mixing, particularly between CT and ππ* states, thereby facilitating reverse intersystem crossing (*k*_RISC_) in accordance with the El-Sayed rule. For instance, bulky groups like butyl, t-butyl triphenylamine and triptycene in t-DABNA (151),^114^ the t-DAB-DPA (154)^116^ (see Figure 10), and Tp-DABNA (356)^217^ effectively suppress ACQ and spectral broadening, even at higher concentrations, without compromising narrowband emission. Modifications of DABNA-1 (1), such as introducing m-xylene or *para*-phenyl groups, have localized triplet states in the vicinity of S_1_ and T_1_, simultaneously enhancing excitons harvesting and suppressing ACQ. As a result, twisted mBP-DABNA-Me (357)^218^ or pBP-DABNA-Me (358)^219^ exhibited more efficient deep-blue emission at high doping concentrations than the parent DABNA-1 (1) emitter.

**Figure 22:**
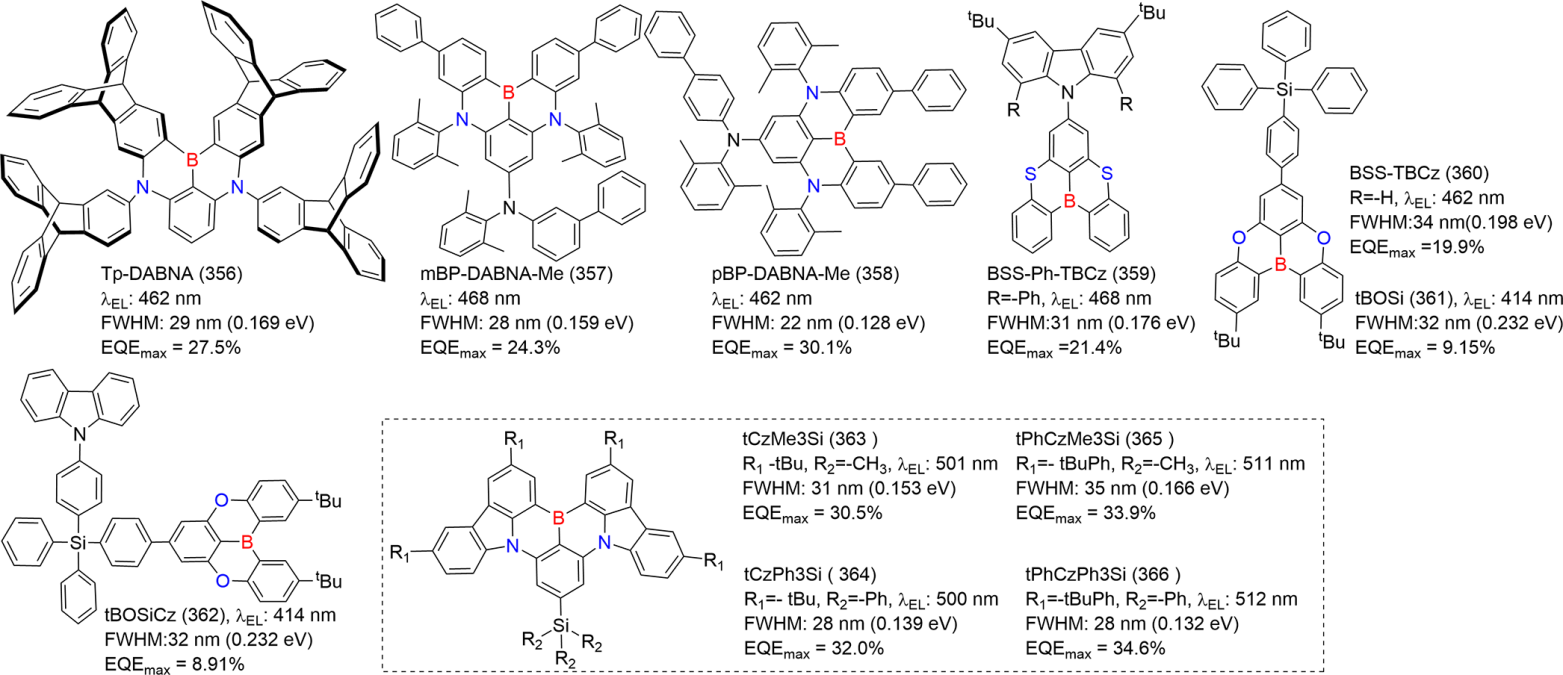
Some representative molecules of addressing ACQ for B/N-type MR emitters.

MR emitters based on the B/chalcogen frameworks have also undergone significant improvements in addressing ACQ. For instance, BSS-Ph-TBCz (359), featuring a 1,8-diphenyl-carbazole at the *para*-position of the B/S-doped MR emitter, maintained consistent emission profiles across doping concentrations (1–100 wt %) compared to the ACQ-prone BSS-TBCz (360).^220^ Additionally, introducing silyl units with sp^3^ hybridization has shown promise in mitigating ACQ due to their nonplanar geometry. Emitters like tBOSi (361) and tBOSiCz (362) achieved near-ultraviolet emission at 414 nm with narrow FWHMs (∼32 nm) in solution-processed OLEDs, irrespective of doping levels.^221^ Similarly, silyl-decorated emitters such as tCzMe3Si (363), tCzPh3Si(364), tPhCzMe3Si (365), and tPhCzPh3Si (366) exhibited promising OLED performances, among which tPhCzPh3Si (366) emitted a pure green light at 512 nm with a high EQE_max_ of 34.6%.^222^

For BCz-BN (5)-based emitters, introducing sterically hindered units, such as phenyl derivatives or spiro-bifluorene groups, has proven effective in mitigating ACQ (see Figure 23), certified anti-ACQ data at high doping concentrations, along with mild efficiency roll-offs, can be found in Table S4. For example, TW-BN (367) (also named TCzBN-TMPh^223^), TPh-BN (368), pCz-BN (369), mCz-BN (370), SPAC-tCzBN (371) (also named BNCz-SAF^148^), SPBAC-tCzBN (372), BNSi (373), o-SPAC-tCzBN (374), BN-N-TPA (375), TPA-Cz-BN (376), TPA-PCz-BN (377), BN-PCz-TPA (378), TCzBN-DPF (379), TCzBN-oPh (380), p-1-PCzBN (381), m-1-PCzBN (382), BNCz-pTPA (383), BNCz-mTPA (384), IDAD-BNCz (385), and TIDAD-BNCz (386) were designed with sterically hindered units connected via single bonds, which successfully weaken the intermolecular π-π stackings of neighboring molecules and inhibit the notorious bimolecular interactions of the rigid molecular structure, thus hindering ACQ as well as suppressing spectral broadening.^223–230^ Notably, sterical groups, e.g., triphenylamine, also promote aggregation-induced emission enhancement (AIEE);^228^ acridine moieties also induce high-lying charge transfer excited states that facilitate *k*_RISC_.^230^ Shield-like SF1BN (387) exhibited minimal spectral broadening at higher doping ratios, unlike its less hindered counterpart SF3BN (388).^231^

**Figure 23:**
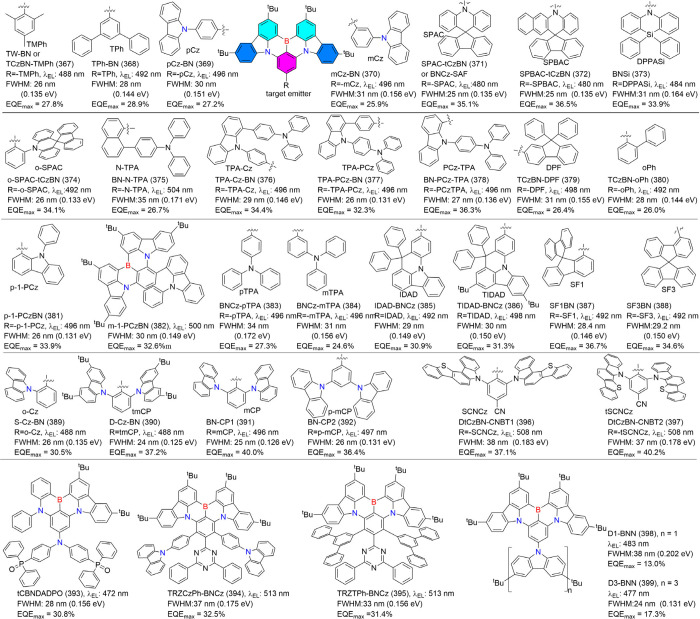
Representative molecules of addressing ACQ for MR emitters based on BCz-BN (5) framework.

Innovative strategies like the “self-host” approach, which has been previously adopted in aggregation-induced delayed fluorescence (AIDF),^232^ have also been applied to mitigate ACQ. Wrapping the MR core with host-like moieties at C1-substituted phenyl rings has proven particularly effective. This approach has been widely applied in the decoration of BCz-BN (5), achieving desired outcomes (Figure 23). For example, emitters such as S-Cz-BN (389) and D-Cz-BN (390), decorated with phenyl-9H-carbazole, achieved high PLQY of 90% and FWHMs of 25 nm over a doping range of 1–20 wt %.^135^ Similarly, BN-CP1 (391), with its unique three-dimensional geometry, exhibited superior photophysical properties compared to the less shielded chromophore BN-CP2 (392). Due to its relative inertness to doping concentration, BN-CP1 (391) maintained an EQE_max_ of 33.3% without changes in the EL spectrum, even at a doping level of 30 wt %, highlighting the effective elimination of performance-limiting factors such as excimers/aggregates.^137^ Furthermore, introducing a peripherally ambipolar segment to the MR-core proved highly effective in enhancing carrier recombination and exciton/energy transfer. As a result, tCBNDADPO (393) not only successfully mitigated ACQ but also accelerated singlet radiation, and alleviated collisional quenching in both ultrasimple trilayer and heavily doped tCBNDADPO (393) systems.^233^ Additionally, the partial introduction of acceptor units and steric moiety onto the MR skeleton has proven effective in mitigating ACQ. For example, TRZCzPh-BNCz (394) and TRZTPh-BNCz (395), employ a space-confined donor–acceptor (SCDA) molecular design strategy, where electron-rich 9-phenyl-carbazole (CzPh) or terphenyl (TPh) units are positioned adjacent to the TRZ acceptor in DtCzB-DPTRZ (185).^131^ Both emitters exhibited only a slight redshift and maintained high Φ_PL_ values of 83.7% and 84.2%, respectively, even with doping concentrations up to 50 wt %.^234^

Both DtCzBN-CNBT1 (396) and DtCzBN-CNBT2 (397) effectively mitigated ACQ; however, DtCzBN-CNBT2 (397), incorporating a D-A TADF moiety with appropriately higher energy levels, demonstrated a faster *k*_RISC_ compared to DtCzBN-CNBT1 (396), which features a non-TADF D-A moiety.^235^ Dendritic structures have also proven effective in suppressing intermolecular aggregation due to steric hindrance. For example, the third-generation dendritic emitter, namely D3-BNN (399), exhibited a high Φ_PL_ of 92% and an unchanged FWHM of 24 nm at high doping concentration, which was superior to the first-generation D1-BNN (398) counterpart.^236^

Another ingenious approach involves transforming a rigid planar MR framework into twisted geometries, which effectively increases the intermolecular distances between the MR-emitting cores and mitigates ACQ (Figure 24). For instance, DTBA-BN2 (400) and DTBA-B2N3 (401), featuring highly twisted conformations of donors, maintained narrow FWHM and high Φ_PL_ at high doping levels.^237^ Emitters like BNCz-aDMAC (402) and BNCz-PaDMAC (403) that utilize dual-conformational moieties of 10H-spiro­[acridine-9,2′-adamantane] achieved superior ACQ suppression through conformational isomerism. Specifically, the conformational isomerism of BNCz-aDMAC (402) resulted in a lower fraction of quasi-equatorial form compared to BNCz-PaDMAC (403) in amorphous doped film states, suppressing ACQ without sacrificing color purity.^238^ Similarly, adamantane-based emitters demonstrated efficient exciton utilization and ACQ mitigation. For example, BN-Ad (404) could effectively mitigate the intermolecular π-π stacking between the rigid planar skeletons and prevent ACQ compared to the counterpart without the adamantane group.^239^ A-BN (405), DA-BN (406), and A-DBN (407) effectively mitigated intermolecular interactions and suppressed exciton annihilation, thanks to the three-dimensional “umbrella-like” conformation of adamantane-containing spiro-fluorene units.^240^

**Figure 24:**
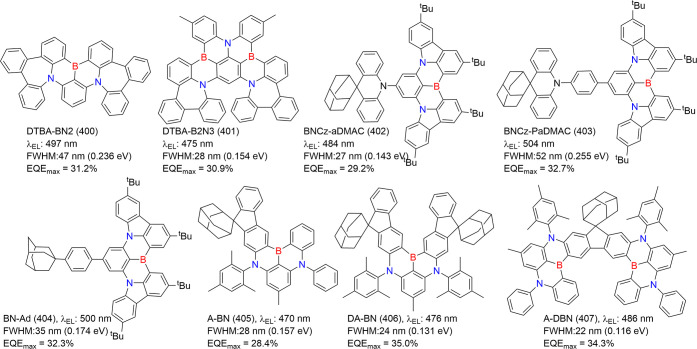
Representative strategies of addressing ACQ via twisted conformations, space-confined donor–acceptor (SCDA), and dendrimer.

Regarding N/-CO-type MR emitters, strategies to mitigate undesirable ACQ effects remain limited, although some progress has been made (see Figure 25). For example, incorporating sterically orthogonal mesityl groups, as seen in Mes3DiKTa (408), prevented ACQ without altering the multiresonance nature of the molecule, in contrast to its precursor DiKTa (2).^241^ Interestingly, the dumbbell-shaped dyad BOQAO (310), featuring a mixed parallel H-aggregate and J-aggregate packing mode, enforces strong intermolecular restrictions, significantly suppressing ACQ and achieving high color purity with FWHMs of less than 35 nm across a broad doping ratio range (1–40 wt %).^198^ SS-DAO (39) employed multiple intramolecular fusion and steric wrapping strategies to mitigate ACQ, achieving an EQE of up to 37.2% with a 10 wt % doping concentration.^48^ The blue emitter TPQAO (409) and TTPQAO (410), featuring ternary wrapping of QAO (2) core demonstrated remarkably quenching-resistant ability, e.g., from 2 to 20 wt %, EQE_max_ only decreases by 4.3% with a slight FWHM broadening of 1 nm.^242^ Moreover, DPQAO-M (411) and DPQAO-F (412), combined intramolecular locking and peripheral shielding in an N/-CO-based MR core to achieve ultrapure emission with a narrow FWHM approximately 24 nm, both in solution and heavy doping thin films.^243^

**Figure 25:**
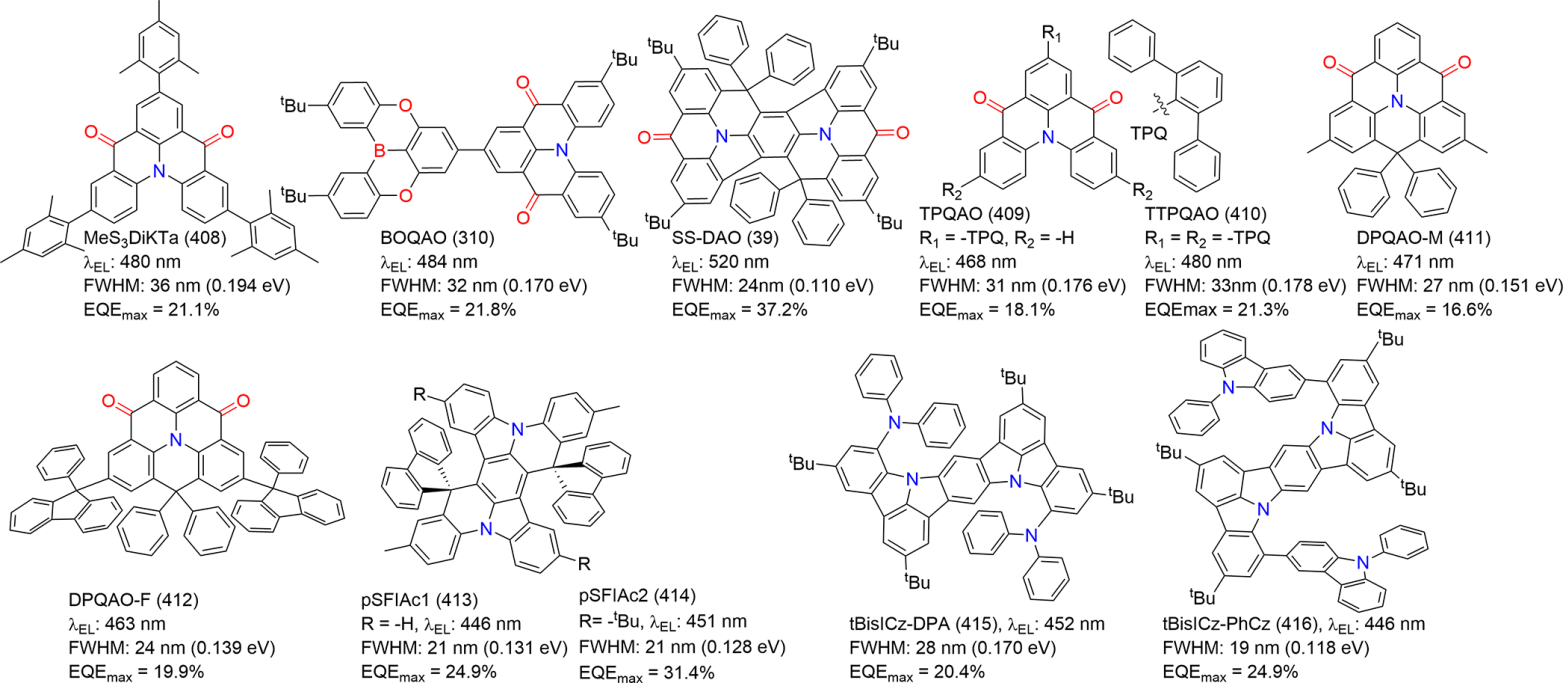
Representative molecules of addressing ACQ for N/CO and N-PAHs-type MR emitters.

Efforts to alleviate ACQ in N-PAHs-type MR emitters include designs such as pSFIAc1 (413) and pSFIAc2 (414), where an orthogonal spiro-structure fused with an indolo­[3,2,1-de]­acridine moiety enables high Φ_PL_ values across various doping concentrations (1–15 wt %) while preserving intrinsic FWHMs (see Figure 25).^244^ Additionally, tBisICz-DPA (415) and tBisICz-PhCz (416), featuring diphenylamine or 9-phenylcarbazole blocking groups on the tBisICz (85) core, effectively reduce intermolecular interactions, thereby enhancing efficiency and attaining narrow emission profiles.^245^

Although the dopant concentration of emitters is very low in device fabrication, it can lead to insufficient host–guest energy transfer and unbalanced charge carrier transport, posing a significant obstacle to overall device performance. Additionally, maintaining a low doping level requires precise control over the evaporation rate, which presents a challenge for industrial production. Therefore, mitigating ACQ is crucial to ensuring reproducibility in device fabrication. Moreover, a moderate molecular weight should be considered; as excessive molecular weight may create technological challenges for vacuum deposition.

## 5 Exciton Utilization

In this section, recently proposed emission mechanisms for MR emitters are reviewed. At the molecular level, a mutually staggered arrangement of FMOs in MR emitters is an intrinsic characteristic of their electronic states. Gaining a deeper understanding of the MR mechanism provides valuable insights to complement existing theoretical prediction methods. FMOs’ visualization and local density of states analysis via scanning tunneling microscopy/spectroscopy (STM/STS) clearly demonstrated that DABNA-1 (1) possesses well-separated FMOs according to the internal arrangement of heteroatoms.^246^ The effectively opposite resonance effect between the carbonyl and nitrogen atoms has been revitalized from the classic MR emitter, namely QAO (2), which was initially categorized as a conventional fluorescent emitter until 2019.^44^ N atom-embedded-PAHs, representing free-acceptor MR emitters, were originally regarded as hole-transporting segments until their unique properties were first reported in 2020.^49^ Additionally, the combination of a relatively large radiative decay rate (*k*_r_), a small nonradiative decay rate (*k*_nr_), and tolerance to oxygen interference explains why DABNA-1 (1) and its oxygen-based analogs like DOBNA (52) show negligible delayed fluorescence, often below the detection threshold of the state-of-the-art spectrometers.^7,247,248^ Therefore, it is important to revisit the emission mechanism of MR emitters. As we know, the pathway of triplet excitons to singlet states, or vice versa, fundamentally determines the nature of the emission—whether fluorescence, TADF, or phosphorescence. A deeper understanding of the mechanism of exciton utilization could unlock new potential in MR molecular design and expand their potential applications.

### 5.1 Revisit the Emission Mechanism of MR Emitters

Whether spin-flipping via RISC occurs, and the speed of this process, plays a crucial role in determining exciton utilization efficiency (EUE) and influences the operational stability and durability of devices. Classic MR emitters, such as DABNA-1 (1) and oxygen-substituted derivatives like BOOO (51)^249^ and BOO (52),^248^ show little or no delayed fluorescence in both solution and pure solid states.

The phenomenon, puzzling at first,^44,247^ has been explained by their slow forward and reverse intersystem crossing rates, which inhibit TADF unless a suitable host material induces an exciplex-like-host and emitter interaction, thus enabling TADF.^73^ Evidence like transient host interactions with DABNA-1 (1) and its time-resolved photoluminescence at low temperatures confirm the exciplex mechanism. This mechanism applies broadly to strong fluorescent dyes without TADF but with thermally accessible Δ*E*_ST_ (Figure 26a).^250^ Another proof of host influence on MR fluorophore exciton processes is further demonstrated in long-persistent luminescence (ΛΠΛ) applications. A glassy steroid-type host, namely gCLA (664), becomes both long-lived fluorescence and phosphorescence through a simple melt-cooling treatment, enabling exciton dissociation and recombination upon photoirradiation. Efficient Förster resonance energy transfer (FRET) from the host to MR-TADF emitters enhances LPL performance (Figure 26b), producing emissions with narrow FWHM of 33 nm, long persistent time over 10 s, and tunable colors ranging from deep blue to orange (414–600 nm).^251^ This mechanism, demonstrated by power-law decay behaviors linked to charge-separated states, contrasts with the multiexponential decay typical of short-lived TADF (e.g., BCzBN (5)). In such cases, efficient energy transfer converts BCzBN (5)’s short-lived TADF (33 μs) into LPL, following a power-law decay profile (Figure 26b).

**Figure 26:**
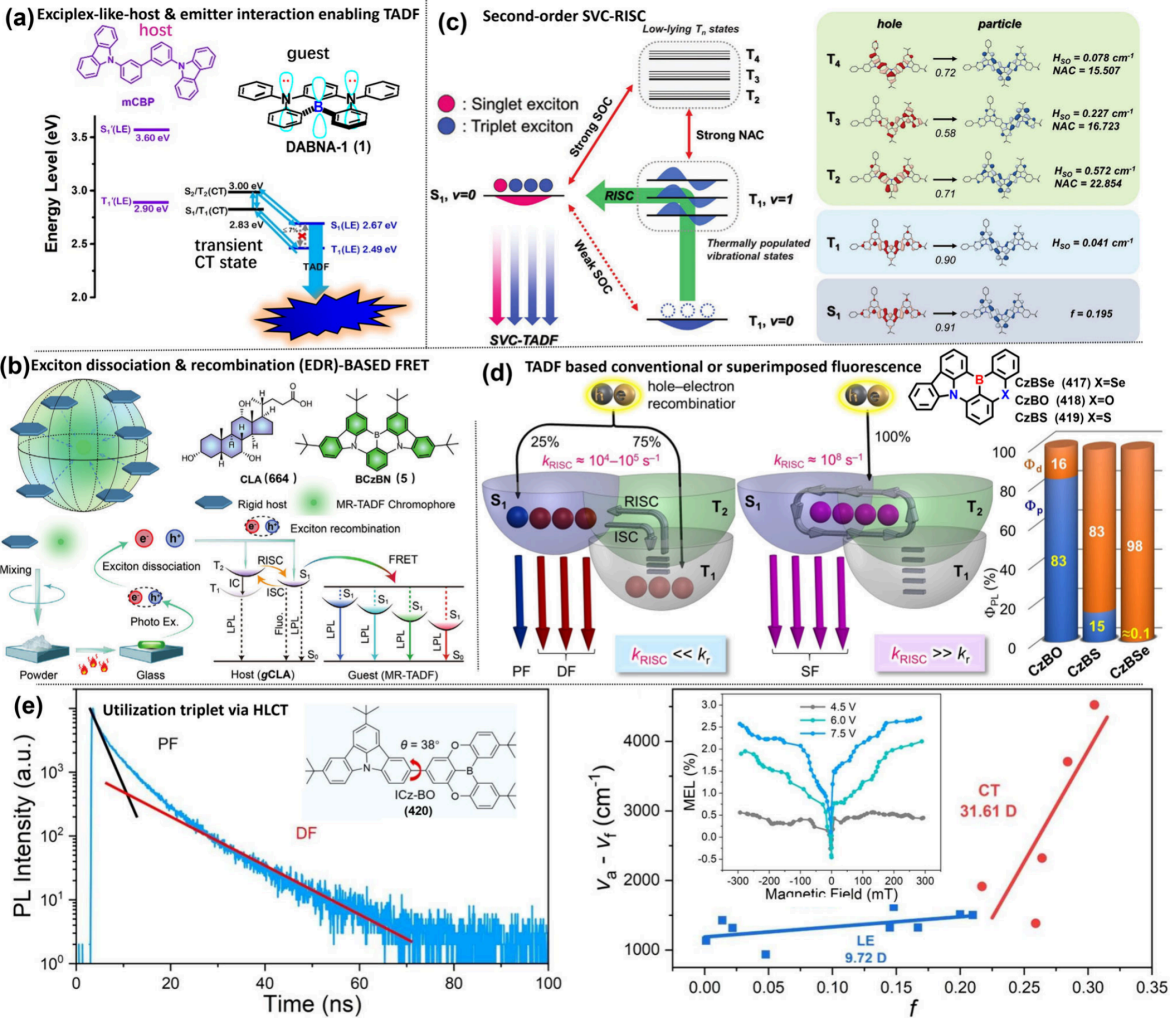
Exciton utilization of MR emitters. (a) The proposed mechanism for the exciplex formation enhancing ISC/RISC in mCBP/DABNA-1 (1) system.^73^ (b) Upper: schematic illustration and molecule structures of cholic acid (CLA) and BCzBN(5) for constructing narrowband organic long-persistent luminescence (LPL) in amorphous. Lower: the preparation of the LPL composite of BCzBN (5)/ gCLA and proposed strategy in realizing narrowband LPL through dissociation and recombination (EDR) and EDR-based Förster resonance energy transfer (FRET). The gCLA (664) is the glassy CLA.^251^ (c) Natural transition orbital calculation results and spin-vibronic-coupling-assisted TADF emission mechanism. NAC stands for nonadiabatic coupling.^80^ (d) Left: conventional TADF-RISC mechanism; Middle: novel ideal superimposed fluorescence (SF) mechanism; Right: CzBO (418) and CzBS (419) exhibit conventional TADF-RISC characteristics, while CzBSe (417) demonstrates an SF mechanism.^253^ (e) Left: transient PL decay spectra in 2.0 wt % ICz-BO (420) doped CBP film. Insert: magnetic field effect on the EL of ICz-BO (420)-doped device at different voltages. Right: linear correlation of the orientation polarization (*f*) of solvent media with Stokes shift (ν_a_–ν_f_).^254^ Reproduced with permission from refs 73, 251 (copyright, 2021 Springer Nature) and refs 80, 253, 254 (copyright, 2024, 2022, 2022, 2024 John Wiley and Sons).

In N-PAHs-type MR emitters, the small SOC between singlet and triplet excited states limits direct spin-crossover. However, higher-lying triplet states can mediate spin-crossover through second-order SVC, enhanced by vibrational energy matching. Natural transition orbital (NTO) analysis reveals that these higher triplet states (T_*n*_, *n* ≥ 2) are close in energy to S_1_, facilitating spin crossover.^252^ SOC analysis, consistent with the El-Sayed rule, shows significantly larger matrix elements between S_1_ and T_*n*_ (*n* ≥ 2) compared to S_1_ and T_1_ due to shared wave functions. Furthermore, the small geometric displacement between S_1_ and T_1_ creates an energetic resonance between the T_1_ states and the higher-lying vibrational states of S_0_. These intraring local π-π* excitations exhibit small energy gaps, large nonadiabatic coupling vectors, and overlap with vibrational modes, enabling second-order SVC-RISC for effective triplet harvesting (Figure 26c).^80^ This mechanism is evident in emitters like p3IDCz (41), where the synergistic between multiple-resonance extension and SVC leads to a record-breaking *k*_RISC_ among N-PAH-type MR emitters (vide infra). Second-order SVC-RISC for effective triplet harvesting not only presents in N-PAH-type MR emitters but also universally exists in B/N-type and N/-CO-type MR emitters.^72,93^

Once the RISC process initiates, triplet excitons can effectively contribute to TADF. Singlet and triplet excitons, denoted by blue and red spheres in Figure 26d, respectively, are formed through hole–electron recombination at a spin-statistical ratio of 1:3. Two scenarios arise depending on the relative rates of RISC and radiative decay (*k*_r_). In the first, where *k*_RISC_ is ≪*k*_r_ the triplet excitons are slowly upconverted into singlet excitons, emitting the delayed fluorescence (DF) after the prompt fluorescence (PF) of the singlet excitons. This necessitates strategies to accelerate spin-flipping during RISC, a critical research focus discussed in subsequent sections. In the second, when *k*_RISC_ is ≫*k*_r_, exciton spin conversion is ultrafast, so excitons (pink spheres, Figure 26d) are indistinguishable as either singlets or triplets due to ultrafast spin-flip conversion, producing quasi-single superimposed fluorescence (SF) without time delay. An exemplary material, CzBSe (417), achieved this behavior with the highest reported *k*_RISC_ value among the vast number of MR-TADF materials (see Table S1).^253^

Another promising strategy to achieve both high efficiency and narrow emission is leveraging the hot exciton mechanism based on the hybridized local and charge transfer (HLCT) model. For example, the near-ultraviolet emitter ICz-BO (420) achieved an EQE_max_ of 12.01% with an ultranarrow FWHM and a CIE_*y*_ coordinate of 0.031 in solution-processed OLEDs. Both experimental and calculated results attribute this remarkable performance to the HLCT mechanism. Specially, the slightly distorted combination of two simple MR subunits induces weak intramolecular charge transfer interaction, resulting in a high-lying reverse intersystem crossing channel. A large Δ*E*_ST_ of 0.48 eV and the absence of μs-scaled delayed fluorescence component indicate the unavailability of TADF mechanism. However, the larger spin–orbit coupling (SOC) matrix element values between the S_1_/S_2_ and T_6_ states suggest the occurrence of a high-lying RISC process, implying the presence of a hot exciton mechanism (Figure 26e). Nevertheless, the competition between the RISC rate and the T_*n*_→T_1_ internal conversion must be carefully considered. The latter is expected to be fast in the condensed phase. Therefore, the efficiency of the hot exciton mechanism should be carefully examined, which we believe remains to be rigorously explored. Furthermore, the Lippert–Mataga model highlights the coexistence of LE-featured and CT-featured radiative transitions. Additionally, magnetic-electroluminescence (MEL) values show an initial increase at low magnetic fields, followed by continuous saturation at higher magnetic fields, effectively excluding the triplet–triplet annihilation (TTA) process.^254–256^

Photoluminescence is often used as a reference for electroluminescence. However, the two phenomena are not equivalent. The proposed mechanism for electroluminescence is typically inferred retrospectively based on experimental results. In electroluminescence, electronic excitation leads to electron–hole recombination, predominantly occurring at the triplet manifold with a 3/4 probability. During the current pulse electroluminescence experiment, the observed decay time is influenced by the system response, which includes the electrical pulse width and the device thickness-dependent electron–hole recombination time. While MR compounds may lack TADF in photoluminescence, RISC can still occur in electroluminescence due to the 3/4 probability of direct population in the triplet state and its significantly longer lifespan. Regardless, solid progress in MR-based OLEDs is steadily moving toward practical applications.

### 5.2 Acceleration of Spin-Flipping RISC

Most MR-TADF materials present conventional TADF-RISC characteristics. Despite having thermally accessible Δ*E*_ST_, they present high *k*_r_ (*k*_r_ > 10^7^ s^–1^) but relatively slow *k*_RISC_ (*k*_RISC_ ≈ 10^4^ s^–1^) compared to traditional TADF emitters. The slower *k*_RISC_ is attributed to the combination of a large Δ*E*_ST_ and negligibly small spin–orbit coupling (SOC) matrix |<S_1_|ĤSOC|T_*n*_>| (see eq 1)^257^ as described by eq 1 (also illustrated in Figure 27).

**Figure 27:**
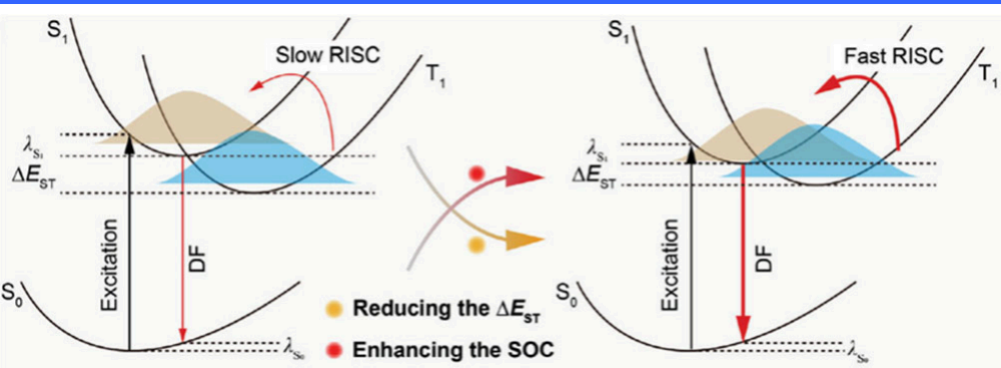
Schematic strategy to accelerate the RISC process of MR-TADF molecules by reducing the Δ*E*_ST_ and enhancing the SOC matrix elements.^24^ Reproduced with permission from ref 24. Copyright, 2023, John Wiley and Sons.

The potential consequences of slow *k*_RISC_ are severe, particularly under high excitation densities, where the buildup of triplets can trigger triplet–triplet annihilation (TTA) and singlet–triplet annihilation (STA). These processes result in detrimental exciton losses and significant roll-off in OLEDs. Therefore, it is essential for the MR emitters to minimize the decay lifetime of the delayed fluorescence (τ_DF_) and accelerate spin-flipping RISC from the perspective of the original molecular design. For instance, the seminal MR emitter DABNA-1 (1) demonstrates trace amounts of delayed fluorescence and long τ_DF_, leading to serious roll-off in OLEDs.^73^ This case highlights inefficient RISC processes as a pervasive issue that hampers the advancement of MR emitters. Accelerating RISC, and thereby enhancing the contributions of delayed fluorescence to the overall radiative process, is a crucial step toward improving the performance of MR-emitter-based OLEDs.^258,259^

Theoretically, exciton spin-flipping during RISC is commonly the rate-limiting step in the overall TADF process. Hereto, many strategies have been developed to accelerate the exciton spin-flipping.

#### 5.2.1 Boosting RISC through Peripheral Decoration with Lone-Pair (n) Electron Groups

Enhancing *k*_RISC_ in MR-TADF materials can be achieved by surrounding the MR-core with additional lone-pair (n) electron groups, which are not directly involved in the resonance but endow the advantages of a twisted D-A configuration (i.e., partial n-π* transition character following the El-Sayed rule).^260^ Actually, such superficial D-A-type MR emitters, characterized by both LRCT states akin to traditional TADF and SRCT states inherent to the MR framework, enable multichannel RISC pathways, thereby enhancing *k*_RISC_ rates without compromising emission efficiency or color fidelity (see Figure 28). Generally, incorporating weak electron-donating units, such as Cz or DPA derivatives, as auxiliary donors is preferred due to their weak donating ability, which avoids perturbation of the MR characteristics. In contrast, stronger donors may transform the emitters into twisted D-A-type TADF systems, leading to a broader FWHM.^261–263^ For example, m-Cz-BNCz (212), BBCz-Y (213), and BBCz-G (214) ingeniously integrated various amounts of tCz groups into the central phenyl ring of BCz-BN (5), significantly improving *k*_RISC_ to above10^5^ s^–1^.^27,145^ Similarly, CzBN3 (also named BNCz-PXZ^148^) (210), BNCz-SAF (371) and BNCz-DMAC (209) employed an orthogonal segment to the BCz-BN (5) framework. This modification “silently” induced intersegmental charge transfer in the triplet state without disrupting the MR character of S_1_ state, resulting in a 23-fold increase in *k*_RISC_ while retaining intrinsic MR-TADF properties compared to BCz-BN (5).^148,149^ Additionally, decorating boron-locked donor units enhances their donating ability and accelerates *k*_RISC_. For instance, BN1 (170) to BN3 (172) demonstrated both red-shifted emission and higher *k*_RISC_ compared to the parent molecule tBCz-BN (5)_._ ^125^

**Figure 28:**
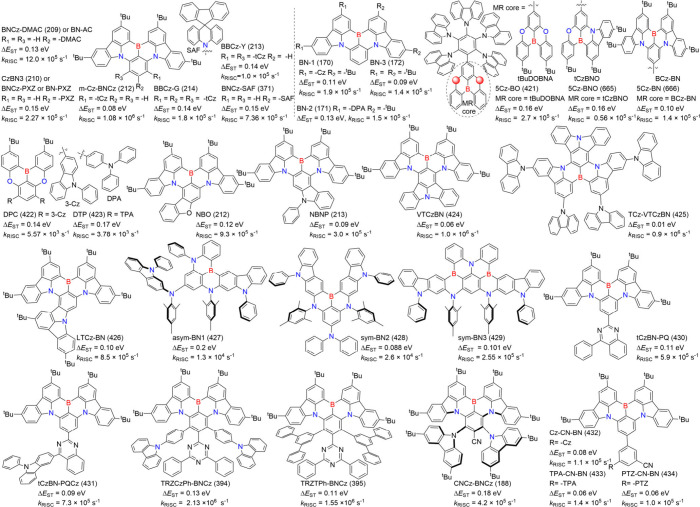
Some representative B/N type of MR emitters, where the MR-cores are surrounded by additional lone-pair (n) electron groups to accelerate spin-flipping RISC.

In contrast, B/O- or B/S-based MR cores are less tolerant of strong electron-donating units in preserving the MR characteristics compared to BCz-BN (5)-core. When electron-donating units are positioned at the *para*-carbon of the boron atom, the LRCT states typically dominate. Although this practice shortens exciton lifetimes to nanosecond scale and accelerates *k*_RISC_, the FMOs distributions often deviate from the MR-TADF scope.^264,265^ Therefore, further elaboration on this aspect will not be presented here. For precise control over SRCT rather than LRCT states, inserting a phenyl-spacer between the amino electron-donating unit and the B/O MR-core enhances emitters’ tolerance to auxiliary donor effects. For instance, DPC (422) and DTP (423) showed blue emissions with sharp peaks at 458 and 470 nm, with small FWHM of 44 and 50 nm, respectively. However, their *k*_RISC_ values remain at a moderate magnitude, below 10^–4^ s^–1^.^266^ Incorporation of multiple carbazole donors into the parent MR core can synergistically modulate excited states and introduce steric hindrances, thereby enhancing both the spin-flip process and quenching resistance. For example, 5Cz-BO (421), 5Cz-BNO (665), and 5Cz-BN (666) (see Figure 28), which employ a hybrid LRCT-SRCT approach with multiple donor moieties, exhibited deep-blue emission at 414 nm with an FWHM of 29 nm and achieved a thousand-fold increase in *k*_RISC_ compared to amino-decorated *meta*-positioned B/O MR-core emitters like DPC (422) and DTP (423).^266–268^ As tabulated in Table S1, emitters with electron-rich donors surrounding the MR-core typically demonstrate faster *k*_RISC_.

Extending the rigid π-conjugation of the B-N skeleton is another effective strategy, particularly when lone-pair (n) electrons are partially delocalized into the π-skeleton, blocking D-A interplay, and preserving MR characteristics (see Figure 28). For instance, high-triplet-energy units like carbazole and dibenzofuran in NBO (225) and NBNP (226), respectively, expanded the π-conjugation of the BBCz-SB (5) framework, enhancing charge transfer delocalization, minimizing Δ*E*_ST_ and accelerating *k*_RISC_.^155^ Emitters such as VTCzBN (424),^269^ TCz-VTCzBN (425),^269^ and LTCz-BN (426)^270^ utilized rational fusion of double resonance units (i.e., ICZ (342) and BBCz-SB (5)), broadening FMOs distributions, reducing Δ*E*_ST_ and enhancing SOC, resulted in *k*_*RISC*_ exceeding 10^5^ s^–1^. Moreover, gradually enlarging ring-fused structures with increasing rigidity, as in asym-BN1 (427), sym-BN2 (428), and sym-BN3 (429), improved transition oscillator strength *f*_osc_ and *k*_RISC_. Notably, sym-BN3 (429) exhibited a more than 10-fold increase in *k*_RISC_ compared to asym-BN1 (427).^29^

Electron-withdrawing units with lone pair (n) electrons can also accelerate *k*_RISC_ by providing dual LRCT channels (see Figure 28). For example, emitters such as tCzBN-PQ (430) and tCzBN-PQCz (431) incorporated quinazoline derivatives as secondary acceptor (PQ) or donor–acceptor moieties (PQCz). These designs generated intermediate locally excited triplet (^3^LE) states, enhancing SOC, accelerating high *k*_RISC_ and suppressing ACQ.^271^ Similarly, TRZCzPh-BNCz (394) and TRZTPh-BNCz (395) combined electron-rich units and a TRZ acceptor to extend FMOs distributions onto the peripheral space-confined donor–acceptor (SCDA) units, reducing ACQ effect (vide supra) while achieving ultrafast *k*_RISC_ of 2.13 × 10^6^ s^–1^ and 1.55 × 10^6^ s^–1^, respectively, with unprecedented τ_DF_ as short as 0.14 and 0.24 μs.^234^ Notably, cyano (CN) groups enhance the CT component of high-lying triplet states, effectively reducing Δ*E*_ST_ and promoting SOC for faster *k*_RISC_. For example, CNCz-BNCz (188), decorated with a *para*-cyano group on the B-substituted phenyl-ring, achieved a red-shifted emission and a superior *k*_RISC_ compared to BBCz-Y (213).^27,132^ Derivatives such as Cz-CN-BN (432), TPA-CN-BN (433), and PTZ-CN-BN (434), featuring CN groups alongside donors like Cz, DPA, and PTZ in the BCz-BN (5) framework, exhibited near-unity PLQY and rapid *k*_RISC_ exceeding 10^5^ s^–1^ while maintaining the MR properties.^272^

Increasing the number of electron-donating groups enlarges the delocalized chromophore, which is especially beneficial for accelerating *k*_RISC_ (see Figure 29). This strategy is crucial for developing deep-blue MR emitters. For instance, ν-DABNA (11), consisting of two auxiliary diphenylamine groups on the *para*-carbons of the B-substituted phenyl-ring, reduced boron’s electron-withdrawing ability, resulting in hypsochromic shifts (ref to Section 3.1.2, vide supra) and a *k*_RISC_ of 1.0 × 10^5^ s^–1^.^9^ Similarly, derivatives (related data cf. Table S1) such as m-ν-DABNA (264), 4F-ν-DABNA (265), and 4F-m-ν-DABNA (266), which involve minor modifications of ν-DABNA (11), also exhibited small Δ*E*_ST_ and rapid *k*_RISC_.^175^ Additionally, hybrid systems such as V-DABNA-Mes (19), V-DABNA (435), V-DABNA-F (436), f-DOABNA (437), DOB2-DABNA-A (438), DOB2-DABNA-A-NP (439), and DOB2-DABNA-B-NP (440), combing oxygen/boron and nitrogen/boron in large π-helical scaffolds, simultaneously facilitated *k*_RISC_ and reduced the molecular weight, making it suitable for device fabrication.^35,273–275^ Notably, *f*-DOABNA (437) achieved a remarkable *k*_RISC_ of up to 2.3 × 10^6^ s^–1^ in the doped film, representing one of the best values in *k*_RISC_ for deep-blue MR emitters.^274^ Additionally, two deep-blue MR-TADF emitters, namely TPD4PA (441) and tBu-TPD4PA (442), synthesized by merging PAB (144) and DOBNA (52) substructures to improve CT characteristics, exhibited very small Δ*E*_ST_ values (≤0.06 eV) and high *k*_*RISC*_ of 2.5 × 10^5^ s^–1^.^276^

**Figure 29:**
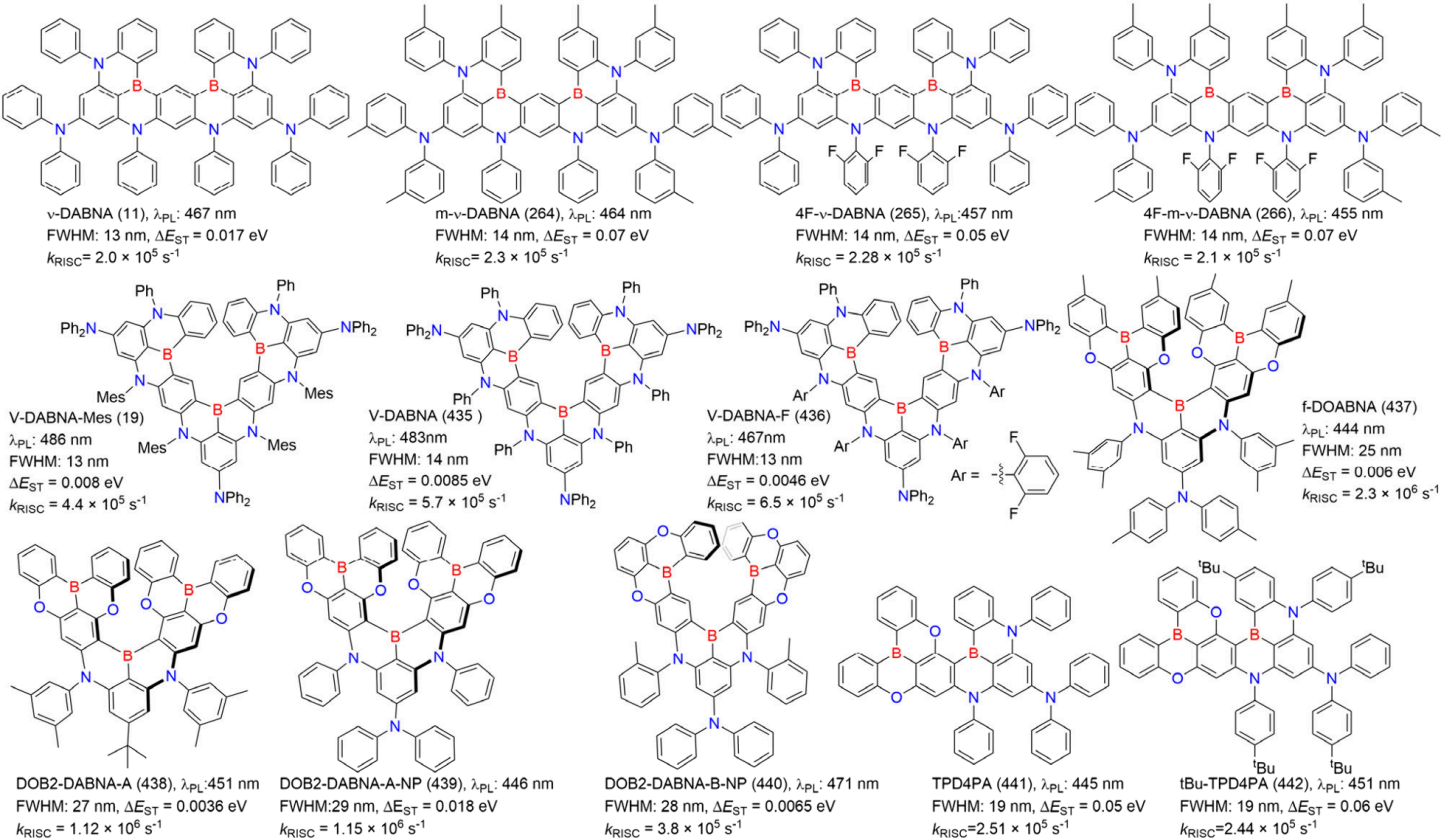
Some representative deep-blue MR emitters with high spin-flipping RISC.

#### 5.2.2 Heavy Atom Triggering RISC

An effective strategy to promote *k*_*RISC*_ is by strengthening SOC through the heavy atom effect, which induces direct spin-flipping channels between T_*n*_ and S_1_ (see Figure 30).^277^ Incorporating different chalcogens, especially those with large atomic numbers like sulfur (S, *Z*_*N*_ = 16) and selenium (Se, *Z*_*N*_ = 34), can strengthen SOC and accelerate *k*_*RISC*_. This is achieved by leveraging the heavy-atom effect, while the lone-pair (n) electrons of these elements contribute to electronic perturbation.^278^ For example, a periphery-locked MR skeleton with a sulfur atom in PTZ moiety or selenium in 10H-phenoselenazine induces distinct TADF characteristics such as shorter decay lifetimes, higher *k*_RISC_, and greater delayed fluorescence contributions compared to counterparts without heavy atoms. These improvements are contributed to larger SOC values and smaller Δ*E*_ST_, as indicated by theoretical calculations and photoluminescence studies. For example, emitters based on boron-locked PTZ units such as BN3 (106),^93^ BN4 (114),^93^ BN5 (115),^93^ Cz-PTZ-BN (443) (also known as BNCzPTZ^279^), and 2Cz-PTZ-BN (445),^280^ as well as boron-locked phenoselenazine moieties like BNSeSe (446),^281^ BNSSe (447),^281^ and BN-Se (448),^282^ as well as peripheral decoration with phenoselenazine group like PSeZBN1 (449) and PSeZBN2 (450),^283^ all exhibited near unity Φ_PL_ and a high *k*_RISC_ exceeding 10^5^ s^–1^. These characteristics, along with large *k*_r_ on the order of 10^7^ s^–1^, ensure excellent efficiencies and ultralow roll-off in OLEDs devices.

**Figure 30:**
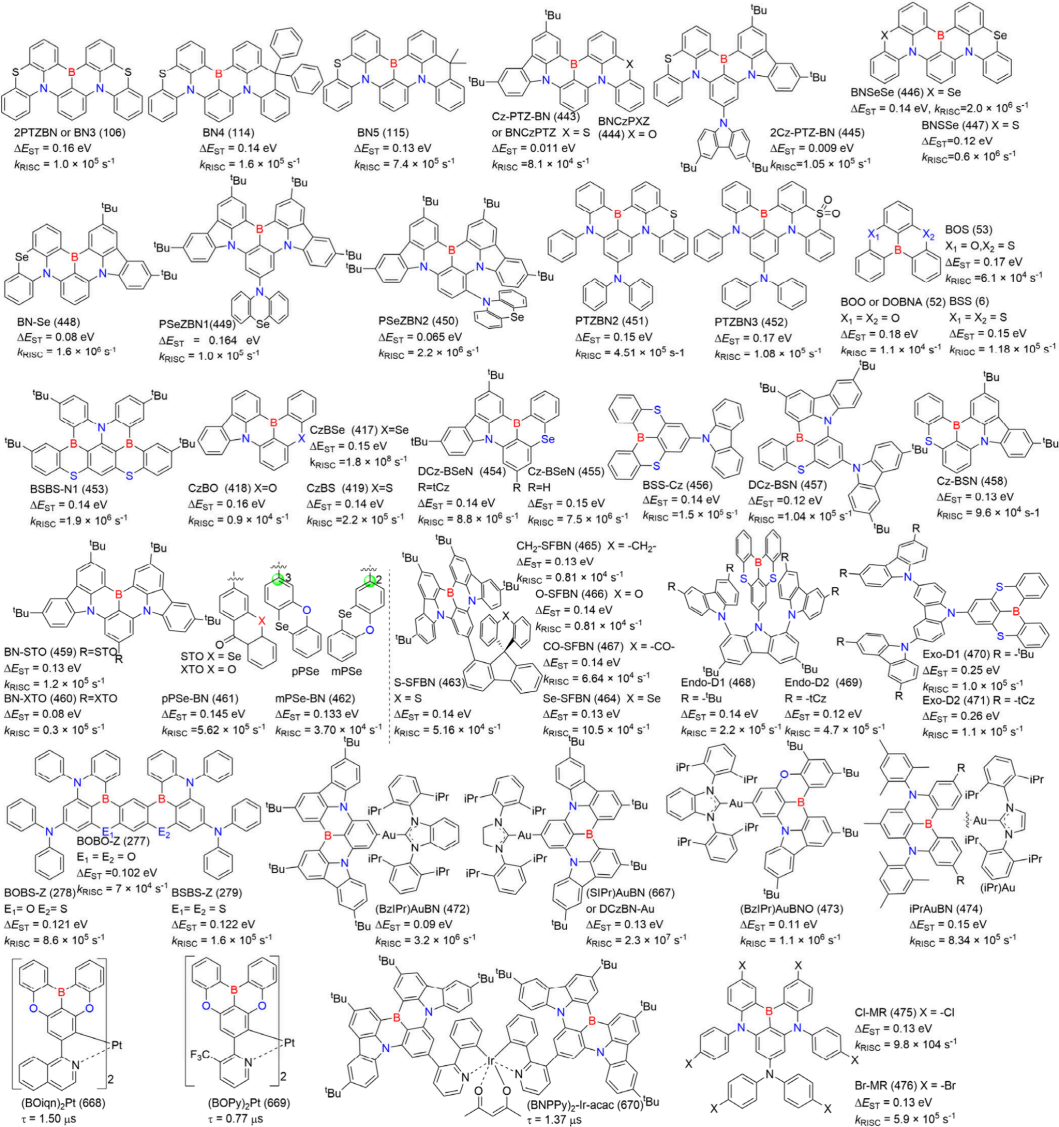
Some representative MR emitters of acceleration spin-flipping RISC by heavy atom effects.

However, the mismatch in atomic radius between chalcogens (e.g., sulfur and selenium) and *para*-positioned nitrogen atoms leads to conformational changes, which in turn can increase FWHM of the emission spectrum. For example, BNCzPTZ (443) exhibited inferior color purity compared to its oxygen counterpart, BNCzPXZ (444).^279^ To preserve narrow FWHM, one approach is to oxidize sulfur to sulfone, which reduces its donor ability and mitigates conformational changes in the excited state. This oxidation can result in a hypsochromically shifted emission with a narrower FWHM while maintaining SOC. For example, PTZBN3 (452) displayed an FWHM of 36 nm and a *k*_RISC_ of 1.08 × 10^5^ s^–1^ compared to the nonoxidative PTZBN2 (451) with corresponding parameters of 42 nm and 4.51 × 10^5^ s^–1^, respectively.^284^ Another strategy to simultaneously accelerate *k*_RISC_ and maintain a narrow FWHM is to create a direct resonant effect between boron and *para*-positioned chalcogens (S and Se). For instance, B/S-doped PAHs, like BOS (53) and BSS (6), have been developed as a novel class of MR emitters with a narrowband emission. The sulfur atom in these compounds lowers the energy bandgap, resulting in red-shifted emission and accelerated *k*_RISC_ relative to their oxygen analogs like BOO (52).^26^ Furthermore, incorporating boron-locked arylamine moieties introduces an additional resonance channel that not only enhances SOC, thereby promoting *k*_RISC_ but also shifts the excited-state characteristics from intersegmental CT to the MR state. Among these, BSBS-N1 (453) featured multiple resonance effects among B/N and B/S, facilitating desired characteristics such as a τ_DF_ of 5.6 μs, *k*_RISC_ of 1.9 × 10^6^ s^–1^, and overwhelming delayed fluorescence, which alleviates efficiency roll-off in OLEDs.^285^ Direct B/Se resonance induces a strong SOC effect and fast *k*_RISC_, with rates several orders of magnitude faster than those in B/N-type analogs due to the heavy-atom effect of Selenium. For example, CzBSe (417), with selenium as a heavy atom, set a new record *k*_RISC_ of 1.8 × 10^8^ s^–1^, which is hundreds of times greater than that of its oxygen and sulfur counterparts (e.g., CzBO (418) and CzBS (419)).^253^ Despite the rapid *k*_RISC_, *k*_r_ of CzBSe (417) dropped to 0.5 × 10^6^ s^–1^, necessitating additional multiple spin-flipping cycles. Consequently, its device performance, including EQE_max_ and roll-off characteristics, still requires improvement. A straightforward solution is to incorporate selenium into a more extended MR skeleton, which can achieve a fast spin-flipping process and improved oscillator strength. Newly reported B, Se, N-doped PAHs emitters with carbazole moieties incorporated at the *para*-position of the boron atom, namely DCz-BSeN (454), exhibited *k*_RISC_ close to 10^7^ s^–1^ and a blue-shifted emission without broadening the spectra, enhancing Φ_PL_ to 93% compared to the parent Cz-BSeN (455).^286^ Similarly, BSS-Cz (456), bearing a carbazole moiety, exhibited MR characteristics with an FWHM of 29 nm.^287^ DCz-BSN (457) exhibited outstanding *k*_RISC_ and device performance compared to the sulfur-only analog, Cz-BSN (458).^288^ However, a fly in the ointment for B/Se-type MR emitters is that the direct incorporation of selenium into the MR framework can result in extended FWHMs due to structural relaxation of phenoselenazine units with folded configurations, as observed in BNSSe (447), BNSeSe (446), and CzBSe (417) (see Table S1). The key to leveraging the heavy atom effect of selenium while preserving narrow FWHMs is through the peripheral linkage of selenoxanthone at the *para*-position of the B-substituted phenyl ring, as seen in BN-STO (459). The planar selenoxanthone not only enhanced the stability of the C-Se bond, improving device stability compared to BNSSe (447) and BNSeSe (446) but also induced red-shifted emission compared to DtBuCzB (5). Importantly, BN-STO (459) exhibited a significantly faster *k*_RISC_ process, four times faster than its oxygen analogous BN-XTO (460).^289^ pPSe-BN (461), with 3-substituted phenoxaselenine (PXSe), exhibited faster *k*_RISC_ and significantly reduced efficiency roll-off in comparison to the control molecule mPSe-BN (462), which is attributed to 3-substituted PXSe enhancing SOC by contribution from the Se orbitals to the high-lying triplets.^290^

In the history of exciton utilization, through-space charge transfer (TSCT) has proven to be an effective strategy for both room-temperature phosphorescence and thermally activated delayed fluorescence.^291–293^ Synergistically combining TSCT with heavy atom effects can further accelerate the reverse intersystem crossing process. For example, S-SFBN (463) and Se-SFBN (464), incorporating a through-space heavy-atom effect on the MR-TADF chromophore BCz-BN (5), exhibited faster *k*_RISC_ by 2 orders of magnitude and shorter τ_DF_ with an order of magnitude than those of their lighter atom analogs, such as CH_2_-SFBN (465), O-SFBN (466), and CO-SFBN (467).^294^ Compared to their exocounterparts (Exo-D1 (470) and Exo-D2 (471)), endoencapsulated luminescent dendrimers, specifically Endo-D1 (468) and Endo-D2 (469), not only demonstrated resistance to the ACQ effect but also exhibited reduced Δ*E*_ST_, thereby accelerating *k*_RISC_. This enhancement is attributed to the through-space interactions between the dendrons and the BSS (6) core via intramolecular π-stacking.^295^

Modification of the star-shape molecule ν-DABNA (11) through the exquisite combination of multiple boron, nitrogen, and chalcogens heteroatoms allows fine-tuning of the emission color while maintaining a narrow FWHM and high *k*_RISC_. As a result, emitters such as BOBO-Z (277), BOBS-Z (278), and BSBS-Z (279), exhibited excellent color saturation in the ultrapure blue gamut, meeting the Rec. 2020 requirement for high-performance displays. Notably, BOBS-Z (278) and BSBS-Z (279), with moderate-heavy atom effects from sulfur, showed more than an order of magnitude increase in SOC compared to BOBO-Z (277) and ν-DABNA (11), thereby accelerating spin-flipping ISC/RISC.^182^

In addition to the heavy-atom effects of chalcogens, the gold­(I) coordination strategy has been employed to enhance the RISC efficiency due to its large SOC constant. A panel of BN­(O)-based MR-TADF emitters, using an easily implementable gold­(I) coordination strategy, involves a luminogen covalently linked to the Au-NHC motif via an Au-Caryl bond at the *para*-B position. This approach enhances SOC, significantly accelerating both *k*_ISC_ and *k*_RISC_. For instance, (BzIPr)­AuBN (472), (SIPr)­AuBN (also named DCzBN-Au^296^) (667) (see Figure 30) and (BzIPr)­AuBNO (473) exhibited the highest *k*_ISC_ among all reported MR-TADF emitters, reaching up to 3 × 10^9^ s^–1^. Moreover, the *k*_RISC_ values of these complexes were greatly enhanced, ranging from 3.2 × 10^6^ s-^1^ to 5.0 × 10^6^ s^–1^, representing a 30- to 170-fold increase compared to their BN and BNO MR-core, respectively.^297^ iPrAuBN (474), which is coordinated to two Au atoms via the deprotonated *para*-C atoms at the B center, significantly reduced the τ_DF_ to 7.8 μs in comparison to 60.6 μs for its parent organic analog.^298^ Iridium­(III) and platinum­(II) complexes, such as (BOiqn)­2Pt (668), (BOPy)­2Pt (669), and (BNPPy)­2-Ir-acac (670) (Figure 30), incorporated into MR-core-based emitters can enhance SOC, enabling the resulting emitters to function as phosphors that exhibited a monoexponential decay lifetime of around 1 μs and faster decay of triplet excitons as the temperature increased.^299,300^ However, the coordinating effect also leads to a high molecular weight, limiting device fabrication options. Notably, the expectation for halogen-substituted MR emitters to enhance *k*_RISC_ was fulfilled in Cl-MR (475) and Br-MR (476). However, this enhancement did not translate into improved OLED performance due to the weak bond dissociation energies of the carbon–halogen bonds (see Figure 30).^301^ Furthermore, device engineering, particularly optimizing the emitter layer through a hyperfluorescence strategy, can significantly boost *k*_RISC_ and hence device improvements (vide infra).

#### 5.2.3 Enhancing RISC through Geometric Arrangement

Optimizing the geometric arrangement presents another effective strategy to enhance to boost *k*_RISC_ (see Figure 31). For instance, the ultrapure green emitter DBTN-2 (477) exhibited a fast *k*_RISC_ of up to 1.7 × 10^5^ s^–1^.^302^ This result highlights that highly distorted fused π-conjugated geometries generate distinct excitation characteristics for the S_1_ (ππ*) and T_1_/T_2_ (hybrid ππ* and πσ*) states, thereby enhancing the SOC between the singlet and triplet excited states. Additionally, introducing multiple carbazole moieties induces charge resonance-type excitation features of the T_1_ and T_2_ states, reducing the T_1_-T_2_ energy gap and opening the T_2_→S_1_ up-conversion channel. A similar structure, m-DBCz (478), demonstrated a small Δ*E*_ST_ of 0.04 eV and a fast *k*_RISC_ of up to 1.65 × 10^5^ s^–1^ in film (see Table S2), significantly outperforming p-TBNCz (479).^303^ Notably, under reaction conditions similar to DBTN-2 (477), the strong steric hindrance of the *tert*-butyl group led to a distinct B/N array pattern in m-DBCz (478). The twisted conformation of DTBA-BN2 (400) and DTBA-B2N3 (401), which are based on heptagonal tribenzo­[b,d,f]­azepine (TBA) donors, further validated this strategy. Their highly twisted geometries in DTBA-BN2 (400) and DTBA-B2N3 (401) enlarged the intermolecular distances between the MR-emitting cores, thereby suppressing the ACQ while enhancing SOC and fast spin-flipping (vide infra) in comparison with BCz-BN (5).^237^ Similarly, introducing an azepine donor with a twisted conformation in TAzBN (480) improved SOC, accelerating *k*_RISC_ to 8.50 × 10^5^ s^–1^ and mitigating ACQ.^304^ Isomeric quadruple-borylation PAHs, including QB-U (481), QB-J (482), and QB-I (483), demonstrated the critical dependence of molecular conformation and electronic topology. The planarized QB-I (442) showed multidimensional improvements in photophysical properties, achieving an ultranarrow emission spectrum with an FWHM of 13 nm and a large *k*_*RISC*_ of 2.7 × 10^6^ s^–1^, superior to the curved QB-U (481) and QB-J (482).^305^ Moreover, DPA-B4 (486), with its highly distorted skeleton in a linearly extended π-skeleton, demonstrated significantly enhanced properties compared to DPA-B2 (484)-based counterparts, such as DPA-B3 (485) and Cz-B4 (487). The substantial SOC arising from the twisted core structure, along with the minimized Δ*E*_ST_ from the higher-order fused-ring frameworks collectively contributed to a rapid *k*_RISC_ of up to 2.29 × 10^6^ s^–1^ (see Figure 31).^306^

**Figure 31:**
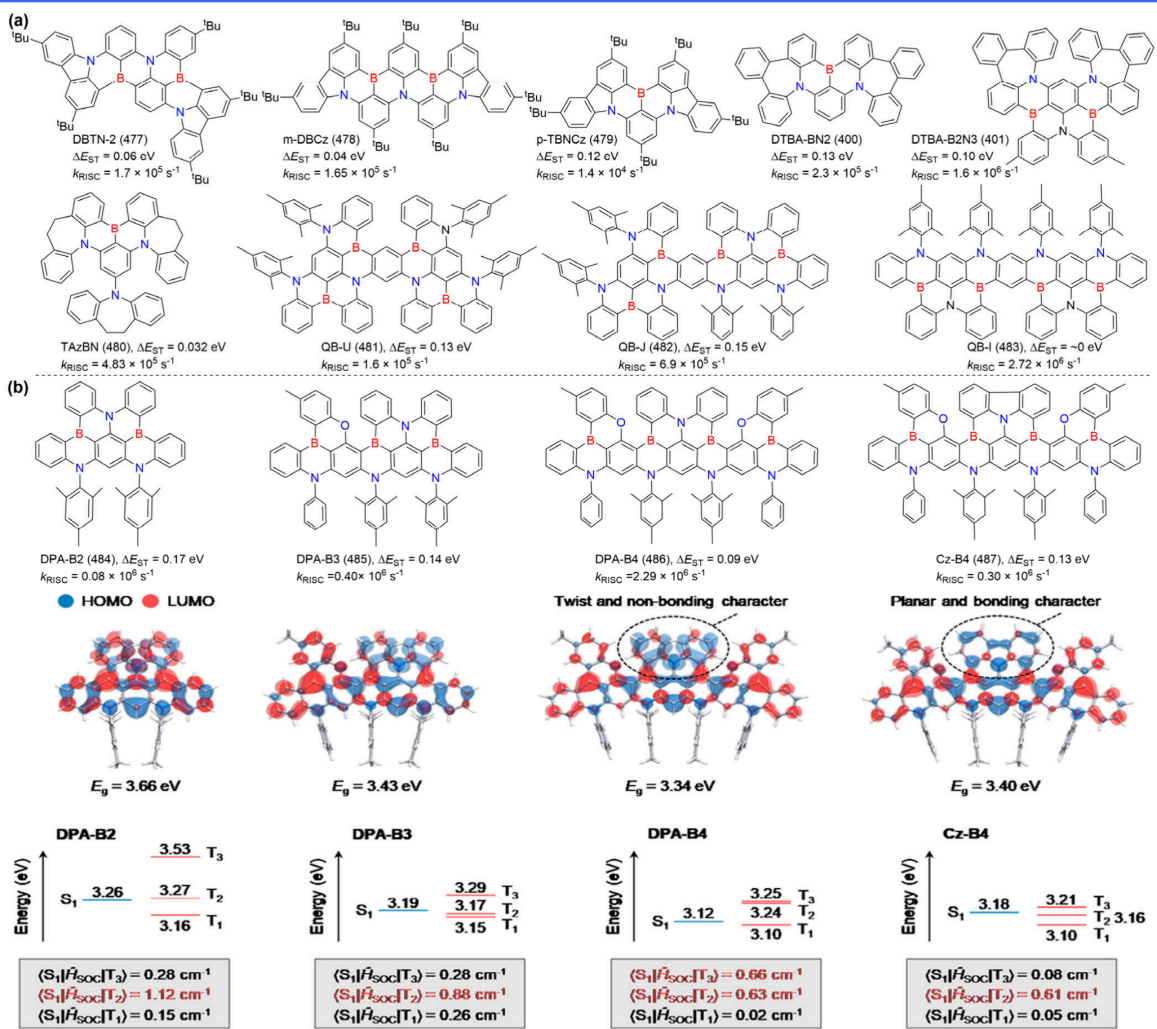
Some representative MR emitters accelerate spin-flipping RISC through fused distorted π-conjugated molecular design.^306^ Reproduced with permission from ref 306. Copyright, 2024, Springer Nature.

#### 5.2.4 Accelerating RISC for Boron-Free MR Emitters

For nitrogen/carbonyl-type MR emitters (see Figure 32, data referred to Table S2), the high *k*_RISC_ of 8.5 × 10^5^ s^–1^ observed in QA-2 (80)-doped films represents a significant advancement. This is attributed to its diverse short-range multisite charge transfer, enabled by the combination of two *meta*-linked electron-donating amino moieties and four peripheral electron-withdrawing carbonyl moieties, which collectively enhance large SOCs between the S_1_ and degenerate S_2_ states.^76^ Another notable example, hp-BQAO (488), showed *k*_RISC_ values up to 2.5 × 10^5^ s^–1^ while suppressing nonradiative decay rates. This improvement arises from the bridged phenyl group, which contributes to a highly twisted saddle molecular skeleton and separated FMOs, leading to increased PLQY and decreased Δ*E*_ST_.^307^ Additionally, MTDMQAO (489), featuring DQAO (71) decorated with a triazine acceptor, achieved efficient narrowband emission and accelerated *k*_RISC_ values of up to 1.93 × 10^5^ s^–1^, attributed to the enhanced SOCs.^308^

**Figure 32:**
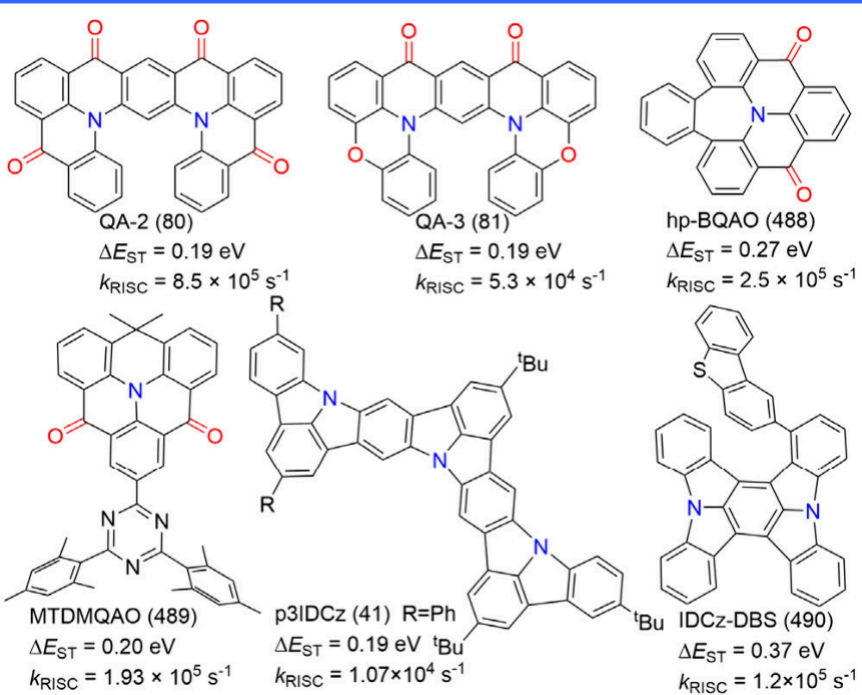
Some representative nitrogen/carbonyl or N-PAHs-type MR emitters with accelerated spin-flipping RISC.

Most N-PAHs-type MR emitters lack TADF properties. However, the extension of MR skeletons has led to significant improvements in optically detectable RISC. For instance, p3IDCz (41) achieved a *k*_RISC_ of 1.07 × 10^4^ s^–1^, a substantial improvement attributed to the small Δ*E*_ST_ and large spin-vibronic coupling (SVC) mediated by lower-lying triplet states, such as the T_2_ state.^80^ IDCz-DBS (490), incorporating a sulfur-containing dibenzo­[b,d]­thiophene moiety into DICz (4), leverages the heavy-atom effect to simultaneously enhance SOC and SVC. This strategy resulted in a record *k*_RISC_ of 1.2 × 10^5^ s^–1^, nearly an order of magnitude larger than previously reported values of N-PAHs-type MR emitters.^309^ However, this rate remains insufficient to compete with the higher *k*_RISC_ values achieved by the B/N-type counterparts (see Figures 28–31, refer to Table S3).

With ongoing advancements of MR-TADF, a new challenge arises: How can the delayed fluorescence occur within 1 μs, allowing MR emitters to compete with noble metal complexes such as iridium and platinum, and potentially replace them in the future? One possibility is that the S_1_ and T_1_ states are sufficiently close in energy to facilitate efficient RISC. This trend represents a key direction for researchers’ efforts. Representative MR emitters with excellent spin-flipping RISC are shown in Figures 28–32. The photophysical data for boron/amino- or nitrogen/carbonyl or N-PAHs-type MR emitters are summarized in Table S1, Table S2, and Table S3, respectively.

## 6 MR-TADF with Circularly Polarized Emission

Undoubtedly, MR emitters have made significant progress in the field of OLEDs, solving color purity issues without the need for color filters in practical applications. While MR emitters have already been employed in panchromatic displays, new-concept applications leveraging functional MR emitters have emerged to meet evolving demands, such as reducing the reflection of external light on the metallic cathode. From a material chemistry perspective, MR emitters with narrow emission profiles combined with circularly polarized luminescence (CPL), denoted as CP-MR-TADF, hold a prominent position in cutting-edge research. These materials aim to simplify device configuration such as without the accessories of the antiglare filter and color filer in practical OLEDs (see Figure 33).^310–313^

**Figure 33:**
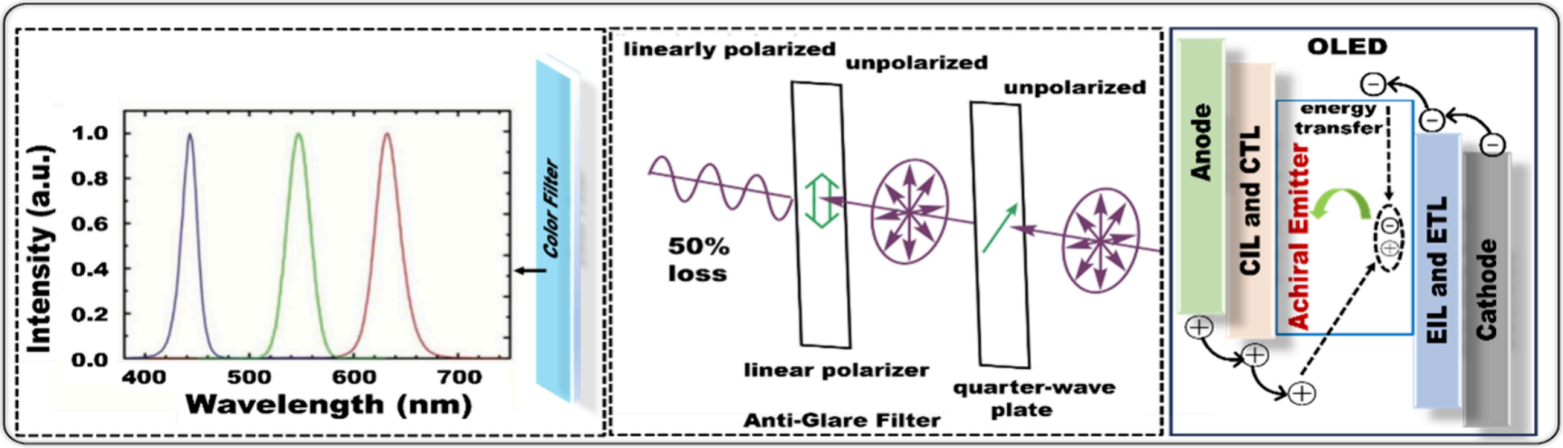
Schematic diagram of the significance of circularly polarized-organic light-emitting diodes (CP-OLED).^311^ Reproduced with permission from ref 311. Copyright, 2023, Elsevier.

The pivotal parameters such as dissymmetry factors *g*_abs_ (or *g*_CD_) and *g*_PL_ (*g*_PL_ and *g*_EL_ that specify the difference between photoluminescence and electroluminescence) are utilized to characterize the ground-state and excited-state chiral properties, respectively. Moreover, CPL provides a powerful and highly sensitive way to determine the conformation of dynamically correlated changes in the chiral structure and hence gives valuable insights into the chiral optical properties. Generally, the full potential of this burgeoning technology can only be realized in OLEDs when g_EL_ exceeds 0.1.^314^ The dissymmetry factor, expressed as

2 $$g_{\mathrm{PL}}=4\left|m\right|\left|\mu \right|\cos\theta /\left({\left|m\right|}^{2}+{\left|\mu \right|}^{2}\right)\approx 4\cos\theta \left|m\right|/\left|\mu \right|$$

where *m* and μ represent the magnetic and electric transition dipole moments, respectively, and θ denotes the angle between *m* and μ. If purely right- or left-CPL is obtained, *g*_PL_ values can theoretically reach −2 or +2.

Generally, m is much smaller than μ for most organic molecules, the denominator can thus be approximated as |μ|^2^. As a result, the *g*_PL_ can be simplified to

3 $$g_{\mathrm{PL}}\approx 4\cos\theta \left|m\right|/\left|\mu \right|$$

A promising emitter should simultaneously exhibit high Φ_PL_ and strong *g*_PL_. However, achieving this balance is challenging, as a high Φ_PL_ is typically associated with a large |μ|, given that the Einstein spontaneous decay rate constant is inherently proportional to |μ|^2^. This relationship poses a dilemma between Φ_PL_ and *g*_PL_. Therefore, CPL brightness (*B*_CPL_) serves as a balance parameter to evaluate both *g*_PL_ and Φ_PL_, providing a comprehensive measure for identifying promising emitters. *B*_CPL_ is expressed as

4 $$B_{\mathrm{CPL}}=\varepsilon \times \Phi \times g_{\mathrm{PL}}/2$$

where ε is the molar coefficient measured at the excitation wavelength.

Innovative strategies to enhance the interplay between *m* and μ are urgently needed to develop novel emitters with unique “opto-electromagnetic properties.” So far, there have been two principal strategies for constructing CP-MR-TADF materials: chiral perturbation and intrinsic chirality. In the former, euclidean chirality sources such as a stereogenic carbon center, axial chirality, or planar chirality, helical chirality is attached to the MR-skeleton without disturbing the FMOs. Conversely, the latter integrates chirality into the π-electron delocalization, enabling active participation in FMOs (see Figure 34). For instance, two pairs of highly efficient green CP-MR-TADF enantiomers, (R/S)-OBN-2CN-BN (491) and (R/S)-OBN-4CN-BN (492), incorporating (R/S)-octahydrobinaphthol units as a chiral perturbation, exhibited pure green emission with *g*_EL_ of +1.43 × 10^–3^/–1.27 × 10^–3^ and +4.60 × 10^–4^/–4.76 × 10^–4^, respectively.^315^ Notably, the electroluminescence simultaneously achieved high efficiency, narrow bandwidth and a CPL response, opening a chapter on the exploration of CP-MR-TADF emitters. Other chiral perturbation strategies, including planar chiral paracyclophane-based emitters such as Czp-tBuCzB (493) and Czp-POAB (494), as well as chiral donor [1,1′-binaphthalene]-2,2′-diamine (BA)-based emitters (R/S)-BA23CzBN (495) and (R/S)-BA34CzBN (496), yielded moderate *g*_EL_ values.^316,317^ Notably, due to the reduced spatial separation between the chiral donor and the electron-deficient boron atom, (R/S)-BA34CzBN (496) exhibited more competitive characteristics, including an accelerated *k*_RISC_ of 3.06 × 105 s^–1^, a decreased τ_d_ of 3.96 μs and a distinct *g*_PL_ value of ±7.7 × 10^–4^ compared to 1.60 × 10^5^ s^–1^, 9.78 μs, and ±3.5 × 10^–4^ for (R/S)-BA23CzBN (495) in dilute toluene (1 × 10^–5^ M), respectively.^317^

**Figure 34:**
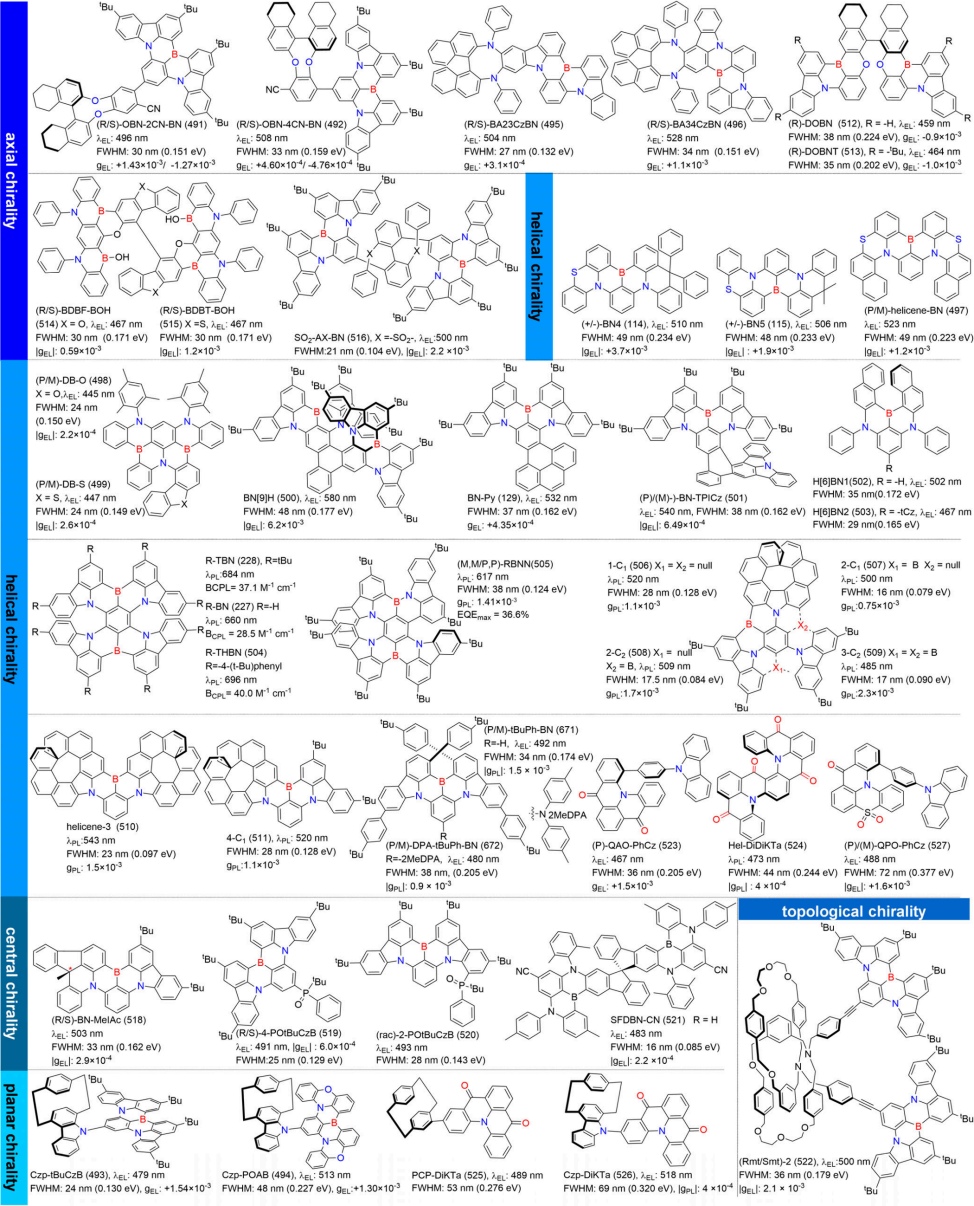
Representative molecules of CPMR-TADF.

However, chirality perturbation strategies primarily facilitate chirality transfer, as evidenced by the exceptionally weak Cotton effect observed for the lowest-lying electronic transition, resulting in a small *g*_PL_. In contrast to this approach, our group integrated intrinsic helical chirality directly into the MR framework. This design channels the chiral property into the π-electron delocalization, enabling it to actively participate in the FMOs. Consequently, following the asymmetrical peripheral-lock strategy, BN4 (114) and BN5 (115) demonstrated intrinsic chirality, achieving an enhanced *g*_EL_ of up to +3.7 × 10^–3^ in solution-processed OLEDs.^93^ Similarly, heterohelicene enantiomers (P/M)-helicene-BN (497), using 12H-benzo­[a]­phenothiazine (BPTZ) as symmetrical donors, displayed narrowband green emission with g_EL_ values of +1.2 × 10^–3^ and −2.2 × 10^–3^, respectively.^318^ Other examples also underscore the advantages of intrinsic helical chirality, such as (P/M)-DB-O (498) and (P/M)-DB-S (499) maintained deep-blue emission with distinct CPL signals,.^319^ B/N-embedded hetero[9]­helicenes, BN[9]H (500), achieved remarkable OLED performance with EQE_max_ of 35.5%, narrow FWHM of 48 nm, and high |g_EL_| of 6.2 × 10^–3^, ascribing to its inherited MR-TADF property and intrinsic helical skeleton.^320^ While less-helicity emitters, such as (P/M)-BN-Py (129) and (P/M)-BN-TPICz (501), displayed narrow green emissions with weak CPL signals.^104,321^ Azabora[6]­helicenes, H[6]­BN1 (502) and H[6]­BN2 (503), exhibited narrowband blue fluorescence and CPL, achieving |g_PL_| values of 4 ∼ 5 × 10^–4^ in hexane (1 × 10^–5^ M).^322^ Promising helical chirality emitters, such as R-BN (227), R-TBN (228), and R-THBN (504) showcased deep red emission with high *B*_CPL_ values up to 40.0 M^–1^ cm^–1^ in dichloromethane solution (1 × 10^–5^ M).^323^ Compared to R-TBN (228), (M,M/P,P)-RBNN (505), introducing a B-N covalent bond to reduce the electron-withdrawing ability of the *para*-positioned B-π-B motif, exhibited electroluminescence peaking at 617 nm with an impressive *g*_EL_ of +1.91/–1.77 × 10^–3^, EQE_max_ of 36.6%/34.4%, and NTSC standard CIE of (0.67, 0.33).^324^ Thanks to the significant steric interactions between the *tert*-butylphenyl groups, which not only induce helical chirality but also enhance the configurational stability of the enantiomers, the separated enantiomers tBuPh-BN (671) and DPA-tBuPh-BN (672) (Figure 34) exhibited |g_PL_| values of 1.5 × 10^–3^ and 0.9 × 10^–3^ in tetrahydrofuran (1 × 10^–5^ M), respectively. They also achieved EQE_max_ of 20.9% and 15.9% at emission wavelengths of 492 and 480 nm with FWHM values of 34 and 38 nm, respectively.^325^ While most 1,4-azaborine-embedded MR emitters exhibit narrowband fluorescence, their CPL spectra tend to be broader. This broadening likely stems from the 1,4-azaborine moiety being integrated into the helical backbone, where vibronic bands exhibit relatively high rotational strength in CPL. By rigidifying and extending the molecular core of 1-C1 (506) through variations in the number and positioning of boron and nitrogen atoms, a series of 1,4-azaborine-embedded helical nanographenes—denoted as 2-C1 (507), 2-C2 (508), and 3-C2 (509)—achieved ultra narrowband CPL with minimal Stokes shifts.^326^ Notably, double helicene helicene-3 (510) and single helicene 4-C1 (511) displayed a *B*_CPL_ of 65 and 36 M^–1^ cm^–1^ in dilute toluene (1 × 10^–6^ M), highlighting the advantage of double-helicenes over single-helicenes (see Figure 34).^327^

Axial chirality represents a facile and effective chiral resource for constructing CP-MR-TADF materials. For instance, (R/S)-DOBN (512) and (R/S)-DOBNT (513), achieved stable chiral configurations facilitated by an auxiliary steric hindrance from a cyclohexyl group, enabling the axial chirality to fully participate in the luminescence process. The dual-core tactics in these emitters increased the transition oscillator strength by 2-fold compared to their monocore counterparts, ultimately achieving obvious circularly polarized electroluminescence with |*g*_EL_| ≈ 10^–3^ and ultrapure blue emission through reduced reorganization energy.^328^ Intrinsically tetraborated axial emitters, namely (R/S)-BDBF-BOH (514) and (R/S)-BDBT-BOH (515), manifested ultrapure blue emission peaking at 458/459 nm and |*g*_PL_| of 6.8 × 10^–4^/8.5 × 10^–4^, respectively, in dilute toluene (5 × 10^–5^ M), due to the delocalization of FMOs across the chiral conjugation-extended bidibenzo­[*b,d*]­furan and bidibenzo­[*b,d*]­thiophene cores.^329^ Sulfur/sulfone-containing biphenyl skeletons fused with B/N-embedded PAHs, (R/S)-S-AX-BN (517) and (R/S)-SO2-AX-BN (516), displayed green electroluminescence with |*g*_EL_| values of 3.3 × 10^–3^ and 2.2 × 10^–3^, respectively.^330^

CP-MR-TADF materials based on central chirality demonstrate notable thermal stability but exhibit moderate CPL characteristics. For instance, enantiomers (R/S)-BN-MeIAc (518) were designed by integrating a quaternary carbon stereocenter-based acridan building block into an azaborine moiety skeleton. These enantiomers, benefiting from the rigid and quasi-planar MR framework, exhibited mirror image CPL spectra with *g*_EL_ values of +2.7 × 10^–4^ for the (*R*)-configuration and −2.9 × 10^–4^ for the (*S*)-form, while achieving excellent EQE_max_ values over 36% with minor low-efficiency roll-off.^331^ (R/S)-4-POtBuCzB (519) and (rac)-2-POtBuCzB (520) represent the first reported examples of phosphorus central chirality with a *tert*-butyl­(phenyl)­phosphine oxide group. Notably, (R/S)-4-POtBuCzB (517) exhibited an exceptionally narrow FWHM of 20 nm and a |*g*_PL_| of 0.54 × 10^–3^ in dilute toluene.^332^ SFDBN-CN (521), derived from the key intermediate 9,9′-spirobifluorene prepared through a simple recrystallization resolution technique and Cl-directed electrophilic borylation reaction, facilitated the development of narrowband blue CPL-OLEDs with an exceptionally narrow FWHM of 16 nm.^333^

Unlike Euclidean chirality, topologically chiral molecules require breaking covalent bonds during racemization, significantly enhancing their chiral configurations’ stability. Topologically chiral [2]­catenanes were thus emplored as key chiral skeletons for CP-MR-TADF emitters, specifically (Rmt/Smt)-2 (522). They displayed unique switchable properties such as in situ dynamic tuning of FWHM and CPL and a high |*g*_PL_| of up to 1.6 × 10^–2^. Moreover, solution-processed CP-OLEDs based on (Smt)-2 (522) demonstrated exceptional performance with a narrow FWHM of 36 nm, an EQE_max_ of 17.6%, and a |*g*_EL_| of 2.1 × 10^–3^, representing a breakthrough in chiral luminescent materials.^334^

Significant advancements have also been achieved in N/-CO-type CP-MR-TADF. For instance, (M/P)-QAO-PhCz (523) employed the QAO (2) framework and ortho-positioned 9-phenyl-9H-carbazole, induced helical chirality and achieved blue emission with EQE_max_ of 14.0%, an FWHM of 36 nm and *g*_EL_ of +1.5 × 10^–3^ simultaneously.^335^ Hel-DiDiKTa (524) in S-shaped triphenylamine diketone double [4]­helicene configuration displayed MR-TADF characteristics with an emission peak at 473 nm and FWHM of 44 nm but suffered from low Φ_PL_ precluding its application in OLEDs.^336^ Planar chiral [2.2]­paracyclophane-based PCP-DiKTa (525) and Czp-DiKTa (526) exhibited chiroptical properties in the ground state, whereas only Czp-DiKTa (526) displayed chiroptical activity in the excited state (|*g*_PL_| = 4 × 10^–4^ in 1 × 10^–4^ M toluene).^337^ Using a QP3O (34) framework, (P/M)-QPO-PhCz (527) achieved sky-blue CPL with *g*_EL_ of up to +1.6 × 10^–3^ (see Figure 34).^46^ To design cutting-edge emitters, theoretical analyses revealed that decorating QAO (2)-based CP-MR-TADF materials with moderate electron-donating/withdrawing groups is advantageous for optimizing θ_μ,m_ (where θ denotes the angle between m and μ), thereby boosting g values.^338^

Despite CP-MR-TADF endowing MR emitters with CPL, challenges such as the high cost of resolving enantiomers and dissymmetry factors far below practical thresholds must be addressed. Cost-effective chiral functionalization and assembly induced chirality enhancement deserve greater attention. Nevertheless, the burgeoning field of organic CP-MR-TADF research is well worth the joint efforts of chemists and materials scientists, along with further investment in the future.

## 7 Application in OLEDs

The aforementioned text emphasizes the diversity of MR emitters’ molecular structures, color modulation, acceleration of *k*_RISC_, suppression of ACQ, and the updated mechanistic studies of MR and MR-TADF. Apart from the intrinsic molecular design engineering of the MR emitters, optimizing device architecture is essential. Furthermore, an MR emitter can only be considered excellent by demonstrating satisfactory performance in practical applications. Therefore, device engineering plays a pivotal role in advancing the practical application of MR emitters. It is widely known that many MR emitters experience prolonged delayed fluorescence, leading to potential photochemical reactions that consume triplet excitons, such as TTA and/or triplet-polaron annihilation (TPA), during device operation. This often results in severe efficiency roll-off and shortening the device lifetimes, which has to be avoided but remains challenging. By optimizing device architectures, balancing charge injection, transport, and recombination, and addressing issues like exciton quenching and efficiency roll-off, device engineering helps maximize the performance of MR emitters, which will be discussed in subsequent sections. Notably, in-depth investigations into device efficiency and stability can provide valuable insights for the design of advanced MR emitters. For example, an analysis using in situ Raman spectroscopy and simulations for BN-PhOH (528), BN-Ph-OCH_3_ (529), and BN-PhN­(CH_3_)_2_ (530) revealed that larger torsion angle changes between the BN core and the peripheral phenyl group contribute to reduced stability and accelerated degradation of BN-TADF emitters (see Figure 35).^339^ The following application in OLEDs aims to provide comprehensive information to assist researchers in device engineering and enhance the understanding of MR-emitter design.

**Figure 35:**
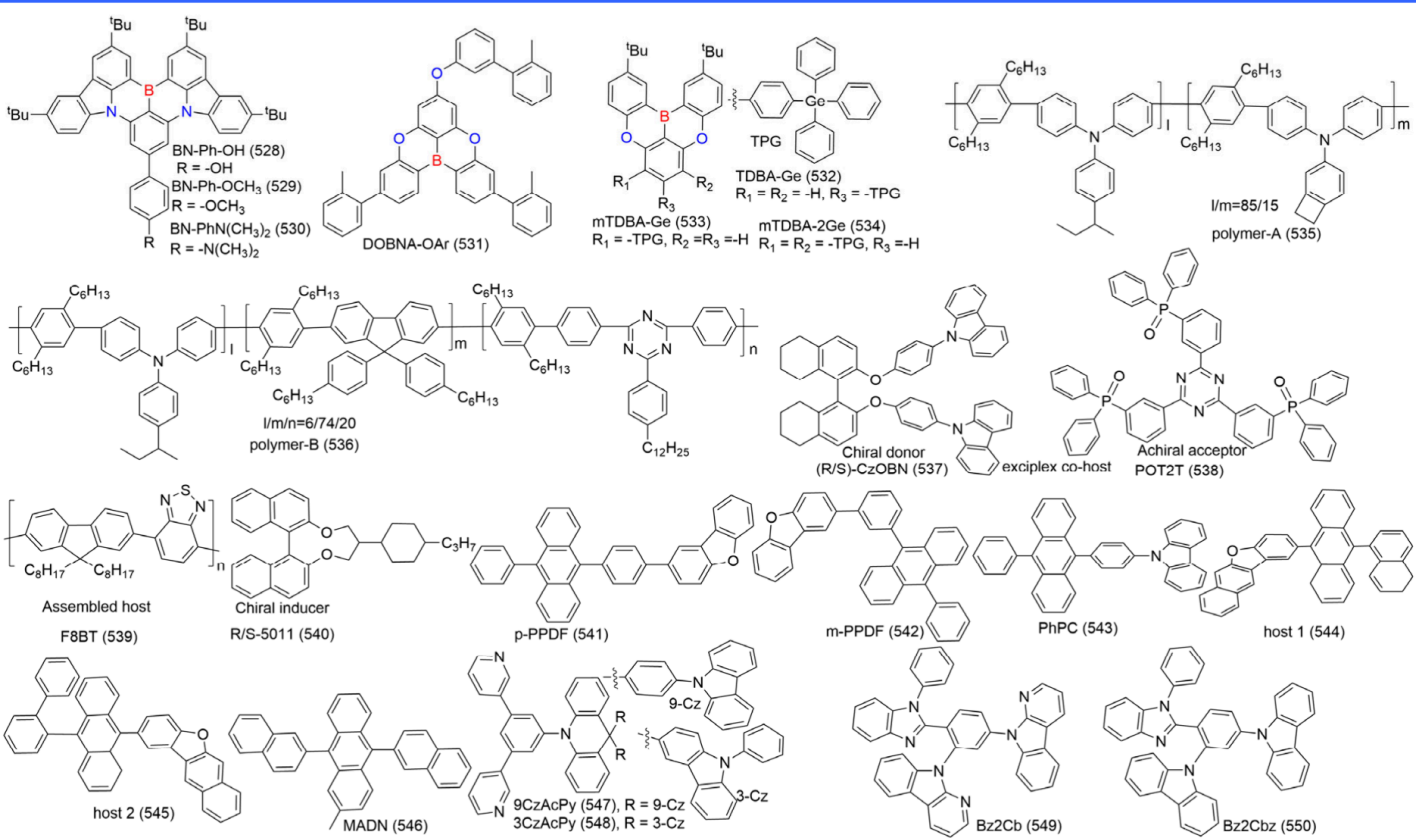
Some representative custom-designed hosts for MR-emitter-based OLEDs.

### 7.1 Custom-Designed Host for MR-Emitter-Based OLEDs

In the MR emitters-based OLEDs, a common approach involves doping these MR emitters into an organic matrix at low concentrations to suppress ACQ and enhance device efficiency and stability. Both the host materials and the MR emitters are critical in determining device performance. Traditionally, MR-emitter-based OLEDs have utilized classic hosts like mCBP (619), and DPEPO (631) (their molecular structure in Figure S1), which were originally developed for second-generation phosphorescent devices. It is worth noting that the characteristic of FMOs in MR emitters is SRCT, distinguishing them from LRCT-dominant emitters like TADF and noble-metal-based phosphors. Fortunately, recent studies have shifted focus toward developing tailor-designed host materials specifically optimized for MR emitters (Figure 35). A notable example is the use of 1 wt % ν-DABNA (11) doped into the custom-designed host DOBNA-Oar (531), which resulted in an impressive EQE_max_ of 34.4% (see Figure 36).^9^ In contrast, doping the same concentration of ν-DABNA (11) into the classic host mCBP (619), leads to a significantly lower EQE_max_ of just 3.7%, highlighting the importance of carefully tailored host materials in optimizing MR emitter efficiency.^340^ This phenomenon can be attributed to an exciplex-like host–emitter interaction that enhances the efficiency, as suggested by the energy levels of the doped film: −3.3 eV/–6.4 eV for DOBNA-Oar (531), −2.8 eV/–5.4 eV for ν-DABNA (11), and −2.55 eV/–6.1 eV for mCBP (619) (see Figure 36).^73^ Adopting hyperfluorescence (HF) technology based on ν-DABNA in the doped mCBP (619) film can enhance operational stability and boost efficiency, thanks to improved singlet-excited-state energy transfer and the horizontal orientation of the transition dipole moment.^340^ Additionally, DOBNA (52), known for its deep blue to ultraviolet emission and high triplet energy, has emerged as a core moiety in many host materials.^27,31,38,248^ Materials incorporating a tetraphenylgermanium (TPG) group into the main backbone of tBuDOBNA (90)—such as TDBA-Ge (532), mTDBA-Ge (533), and mTDBA-2Ge (534)—demonstrated improved performance. In particular, using mTDBA-2Ge (533) with ν-DABNA (11) in the emitting layer resulted in notable improvements in device efficiency and overall performance, emphasizing the advantages of integrating TPG groups into the molecular design.^341^ Additionally, tailor-designed polymers with ionization potentials similar to that of the OAB-ABP-1 (57) emitter guaranteed outstanding performance in solution-processed OLEDs. In this configuration, polymer-A (535) served as a hole-transporting layer, while polymer-B (536) acted as a bipolar host material, promoting efficient charge recombination in the emitting layer and enhancing device performance.^61^

**Figure 36:**
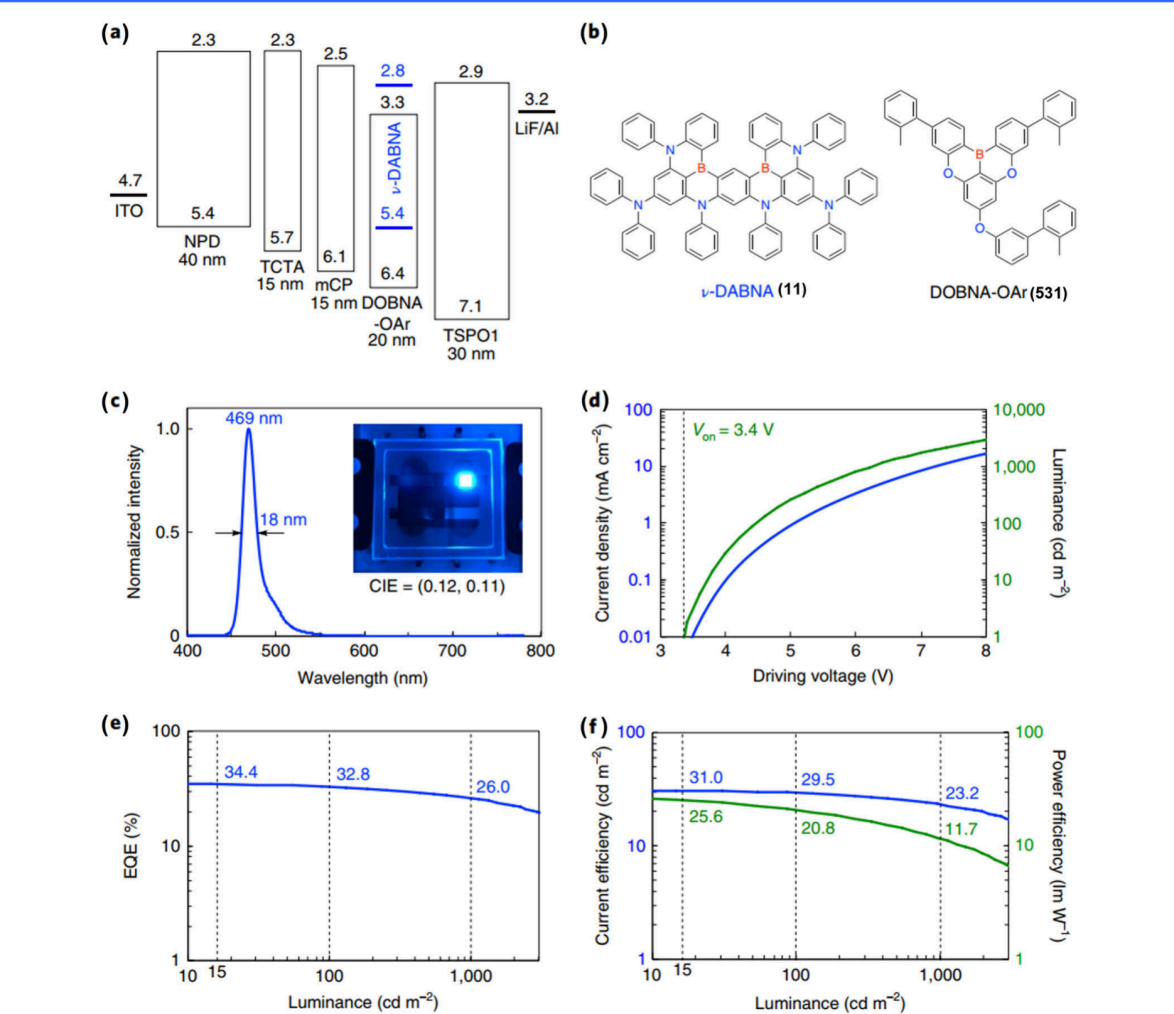
OLED performance. (a) Device structure and ionization potentials and electron affinities (in eV) for each material. (b) Molecular structures used in the emitting layer. (c) Normalized EL spectra and device in operation. Inset: electroluminescence of the device. (d) Current density and luminance versus driving voltage characteristics. (e) EQE versus luminance characteristics. (f) Current and power efficiency versus luminance characteristics.^9^ Reproduced with permission from ref 9. Copyright, 2019, Springer Nature.

By combining structural diversity with functional optimization, co-assemblies provide a versatile platform for advancing MR-emitters-based OLEDs. For example, a chiral exciplex-type co-host, composed of the chiral donor R-CzOBN (537) and the achiral acceptor PO-T2T (538), enabled the achiral green MR-emitter BN1 (170)^125^ to achieve narrowband emission with an FWHM of 42 nm, a high EQE_max_ of up to 33.2%, and a dissymmetry factor *g*_EL_ of 2.8 × 10^–3^.^342^ A promising class of ternary chiral co-assemblies, featuring high PLQY, large *g*_PL_, and narrowband MR characteristics, has also been developed through co-doped thermal annealing treatments. These co-assemblies, involving an achiral luminescent polymer (F8BT (539)), chiral inducers (R/S-5011 (540)), and an achiral FRET acceptor (DBN-ICZ (203)), exhibited strong CPL signals with a *g*_PL_ reaching 0.26. Notably, solution-processedCP-OLEDs fabricated using these ternary chiral co-assemblies as the emitting layer displayed yellow CPEL with an EQE_max_ of 4.6% and a *g*_EL_ of up to 0.16.^343^

Another effective strategy to extend the device’s lifetime while maintaining a high EUE is the triplet–triplet up-conversion (TTU) mechanism. Anthracene-based materials, owing to their low-lying triplet states, effectively facilitate the TTU process and can serve as both host and blocking layer materials. For instance, the electron transport-type host materials, m-PPDF (542) and p-PPDF (541), based on 9,10-diphenylanthracene and dibenzofuran moieties, were utilized with DABNA-NP-TB (154) as the emitting layer. The combination achieved an EQE_max_ of 7.03% with CIE coordinates of (0.136, 0.076) and an operational lifespan (LT95) of 85 h at an initial brightness of 600 cd/m^2^–1.7 times longer than devices with the bipolar host PhPC (543).^117^ Moreover, two TTU-specific hosts, host 1 (544) and host 2 (545), incorporating the benzo­(b)­naphtho­(2,3-d)­furan moiety into the anthracene backbone, balanced hole and electron currents in OLEDs and thereby improved operational stability. Consequently, OLEDs utilizing t-DABNA (151) doped in host 1(544) and host 2 (545) exhibited LT90 values of 249 and 192.3 h, respectively, showcasing 2.5 times greater operational stability compared to the commercially available MADN (546)-based OLED (see Table 1).^344^

**Table 1:** Device Performance of Organic Light-Emitting Diodes (OLEDs) with t-DABNA (151)-Doped EML

Sensitizer	*V*_on_ [v]^a^	EQE [%]^b^	CE [cd A^–1^]^c^	PE [lm W^–1^]^d^	λ_EL_/FWHM[nm]^e^	CIE _(*x*,*y*)_ ^f^	LT (h)^g^(brightness)	Ref.
DMAC-DPS[Table-fn t1fn8]	-	31.4/27.2/19.8%	32.6/28.9/20.9	33.6/21.0/10.9	464/31	0.13, 0.15	-	^114^
p4TCPhBN[Table-fn t1fn9]	-	32.5/26.4/23.2	-	-	466/29	0.13, 0.12	LT_80_ = 60 (1000 cd/m^2^)	^345^
Host 1[Table-fn t1fn10]	2.7	11.70/11.40/11.51	8.49	8.89	463	0.125, 0.098	LT_90_ = 249.0 (1000 cd/m^2^)	^344^
Host 2[Table-fn t1fn10]	2.7	10.40/10.15/9.87	7.11	7.52	463	0.124, 0.102	LT_90_ = 192.3 (1000 cd/m^2^)	
MADN[Table-fn t1fn10]	2.6	9.60/9.55/9.55	7.26	8.15	463	0.124, 0.102	LT_90_ = 100.4 (1000 cd/m^2^)	
m-tz2[Table-fn t1fn11]	3.7	19.7/–/–	20.0	17.5	468/31	0.12, 0.13		^346^
Ir(cb)3[Table-fn t1fn12]	-	24.8/-/18.4	22.6/-/	20.2/-/8.5	-	0.131, 0.107	LT_50_ = 293 (200 cd/m^2^)	^347^
*f*-tpb1[Table-fn t1fn13]	4.6	29.6/–/–	28.7/–/–	19.6/–/–	462/30	0.13, 0.11	-	^348^

a Voltage at 1 (turn-on voltage, *V*_on_), 1 cd m^–2^;

b EQE for maximum, and at 100 and 1000 cd m^–2^, respectively;

c Current Efficiency for maximum, and at 1000 cd m^–2^;

d Power Efficiency for maximum, and at 1000 cd m^–2^;

e λ_EL_: EL emission maximum, and FWHM: full width at half maximum;

f Commission Internationale de l’Éclairage color chromaticity coordinates;

g 90% of an initial luminance of 1000 cd m^–2^.

d1 ITO/PEDOT:PSS (60 nm)/ TAPC (20 nm)/ mCP (10 nm)/ DPEPO: 30 wt % DMAC-DPS: 1 wt % t-DABNA (25 nm)/ TSPO1 (5 nm)/ TPBi (20 nm)/ LiF (1.5 nm)/ Al (200 nm);

d2 ITO/HATCN (5 nm)/ NPB (30 nm)/ TCTA (10 nm)/ mCPCz: 40 wt % p4TCzPhBN: 2 wt % t-DABNA (30 nm)/ CzPhPy (10 nm)/ DPPyA:Liq (1:1, 30 nm)/ LiF (0.5 nm)/ Al (150 nm);

d3 ITO/3 wt % p-dopant: HTL1 (10 nm)/ HTL1­(50 nm)/ HTL2 (5 nm)/ 3 wt % t-DABNA: host (25 nm) (20 nm)/ ETL1 (5 nm)/ 50 wt % Liq:ETL2 (30 nm)/ Liq (2 nm)/ Al (120 nm);

d4 ITO/HATCN (10 nm)/ TAPC (35 nm)/ TCTA (10 nm)/ DPEPO:40 wt % m-tz2:1 wt % t-DABNA (25 nm)/ DPEPO (5 nm)/ TmPyPB (45 nm)/ LiF (2 nm)/ Al (120 nm);

d5 ITO/PEDOT:PSS (60 nm)/ TAPC (20 nm)/ mCP (10 nm)/ TSPO1:30 wt % Ir­(cb)_3_: 1 wt % t-DABNA (25 nm)/ TSPO1 (5 nm)/ TPBi (20 nm)/ LiF (1.5 nm)/ Al (200 nm);

d6 ITO/HATCN (10 nm)/ TAPC (25 nm)/ mCBP: 30% *f*-tpb1:1% t-DABNA (30 nm)/ TmPyPB (35 nm)/ LiF (1 nm)/ Al (100 nm).

The newly developed hosts, 9CzAcPy (547) and 3CzAcPy (548), possessing high triplet energy levels, high thermal stability, and excellent film morphology, allowed solution-processed OLEDs based on BN-CP1 (391)^137^ to achieve an EQE_max_ of 26.6%, marking one of the highest efficiencies reported for solution-processed MR-TADF OLEDs.^349^ Additionally, highly pure deep-blue OLEDs were realized through vapor-deposition doping of DABNA-O-Me (281) into films of asymmetric hosts Bz2Cb (549) and Bz2Cbz (550). Remarkably, the device with Bz2Cbz (550) exhibited electroluminescence at 464 nm with a narrow FWHM of 22 nm and an EQE_max_ of 28.2%, achieving a true-blue CIE of (0.13, 0.07) and an impressive blue index (defined as the ratio of current efficiency to CIE_*y*_) of 253.^350^

To enhance clarity and convenience for readers, we acknowledge that the same materials may be abbreviated differently across the literature. Therefore, we have summarized the molecular structures of commonly utilized materials in devices—including hosts, assistant dopants, and transporting layer materials—in Figure S1. This summary aims to provide a comprehensive reference for understanding their roles and designations in device architectures.

### 7.2 Binary-System Emitting Layer

Binary emitter layers simplify device fabrication by eliminating the need for assistant dopants, thereby reducing operational voltages and manufacturing costs. The pioneering MR emitters, DABNA-1 (1) and DABNA-2 (551) (molecular structure refer to Figure 37) exhibited pure blue emission with narrow FWHMs in binary emitter layers comparable to that of the Samsung Galaxy S5. Despite significant efficiency roll-off (see Table S4), devices based on DABNA-2 (551) showed a substantial improvement over DABNA-1 (1), attributable to the faster *k*_RISC_ of DABNA-2 (551) (see Table S1).^7^

**Figure 37:**
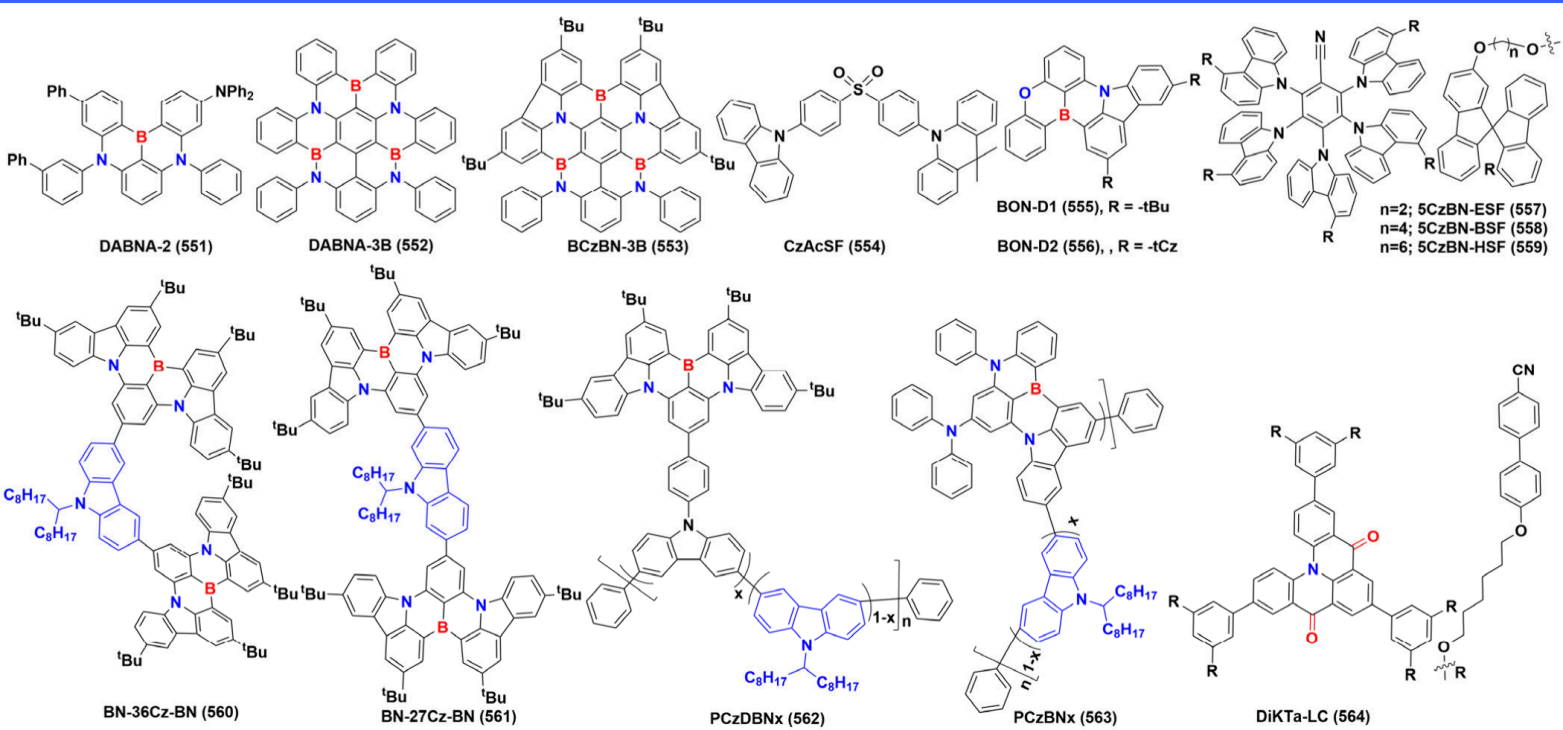
Molecular structures (No. 551–564) without reference in the above text.

Efforts to address the performance limitations of DABNA-1 have focused on intrinsic emitter improvements, targeting faster *k*_RISC_ and shorter τ_DF_ without compromising color purity. Substantial progress has been made in achieving panchromatic emission via vacuum-deposited binary systems. The adoption of the Rec.2020 color gamut—based on RGB primaries with chromaticity coordinates of (0.708, 0.292), (0.170, 0.797), and (0.131, 0.046)—has driven advancements in novel materials capable of simultaneously delivering monochromatic colors with narrow emission spectra and high efficiency. A milestone in this field is the blue emitter ν-DABNA (11), which has set a record performance in binary-system emitter layers with an emission peak at 469 nm, an EQE_max_ of up to 34.4%, and an FWHM of 18 nm. (see Figure 36).^9^ Unleashing blue could have a huge impact on the display and lighting industries, eventually making large-area, efficient OLED light sources a reality.^351^ Therefore, structural modifications of ν-DABNA (11) have further refined functional molecular FMOs distribution, enabling compliance with Rec.2020 color gamut while maintaining high efficiency and narrow FWHM. For example, NO-DBMR (280) achieved EL at 469 nm with an EQE_max_ of 33.7%;^177^ QB-I (483) delivered a high EQE of 30.4% at 1000 cd m^–2^ with CIE coordinates of (0.127, 0.078), without additional sensitizer achieved.^305^ ν-DABNA-CN-Me (267) showed a remarkable bathochromic shift to 504 nm due to the extension of the *para*-boron LUMO distribution via cyano groups, achieving an EQE_max_ of 31.9% and suppressed efficiency roll-off at high luminance.^42^ Deep blue emitters, TPD4PA (441) and tBu-TPD4PA (442)­exhibited narrowband deep blue emissions with EQE_max_ values of 30.7% and 32.5%, respectively, and CIE_*y*_ coordinates of 0.06 and 0.07, approaching the NTSC and BT.2020 standards.^276^

Emitters based on *meta*-carbon positioned BCz-BN (5), adorned with multifarious functional groups, not only induced bathochromic shifts with narrow emissions but also demonstrated exceptional performance in OLEDs. For instance, emitters like m-Cz-BNCz (212)^145^ and m-DPAcP-BNCz (218)^151^ simultaneously achieved narrow green emission and high EQE_max_ over 31% in binary-emitting layers. By leveraging the heavy atom effect, BN-STO (459) demonstrates state-of-the-art performance with an EQE_max_ of 40.1%, a well-suppressed efficiency roll-off, and a pure green gamut.^289^ Furthermore, DBTN-2 (477) enabled an ultrapure green OLED with a narrow FWHM of 29 nm, an EQE_max_ of 35.2%, and satisfying CIE coordinates of (0.19, 0.74).^302^ Thanks to the short τ_DF_ and rapid *k*_RISC_, TRZCzPh-BNCz (394) and TRZTPh-BNCz (395) in commonly used CBP host achieved high performance with EQE_max_ values as high as 32.5% and 31.4%, respectively, along with alleviated efficiency roll-off.^234^ Notably, TCz-VTCzBN (425) achieved an EQE_max_ of 32.2% with CIE_*y*_ meeting the green display standard of the NTSC requirements, owing to its exceptionally fast *k*_RISC_ exceeding 10^6^ s^–1^ and significant molecular planar orientation.^269^ The introduction of B-N covalent bonds for π-extension in DABNA-3B (552) and BCzBN-3B (553) not only enhanced molecular rigidity but also promoted *k*_RISC_ (see Figure 37). Consequently, single-host OLEDs based on BCzBN-3B (553) achieved an EQE_max_ of up to 42.6%, while effectively suppressing efficiency declines even at high brightness levels.^352^ Additionally, BBCz-R (7), a representative red B/N-type MR emitter, displayed excellent performance with an EQE_max_ of 22.0% and an FWHM of 21 nm at an emission peak of 616 nm in a conventional device configuration.^27^

Binary-system OLEDs compatible with solution processes also achieve state-of-the-art performance, offering an alternatively cost-effective approach comparable to vacuum-evaporated strategies.^353^ For instance, BCz-BN (5) and Cz-BN (102), based on the TADF host of CzAcSF (554) in solution-processed binary-system OLEDs, exhibited efficient bluish-green electroluminescence with EQE_max_ values of 16.3% and 14.7%, respectively.^354^ V-DABNA-Mes (19) in a polymer host exhibited narrowband emission at 480 nm with an EQE_max_ of 22.9%.^35^ BON-D1 (555) and BON-D2 (556) with steric carbazole dendrons emitted at 488 nm with an FWHM of 39 nm and an EQE_max_ of 13.4% in solution-processed OLEDs, effectively suppressing ACQ effect while maintaining narrowband emission characteristics.^355^ S-Cz-BN (389) achieved an EQE_max_ of up to 25.6% with a narrow FWHM of 29 nm in solution-processed OLEDs, attributed to the bipolar TADF hosts 5CzBN-ESF (557), 5CzBN-BSF (558), and 5CzBN-HSF (559) with high *Θ_∥_*.^356^ BN-36Cz-BN (560) and BN-27Cz-BN (561), which incorporated two MR units onto a carbazole bridge bearing long alkyl chains, showcased excellent film-forming capability and narrowband emissions in solution-processed electroluminescent devices.^357^ The blue TADF conjugated polymers PCzDBNx (562), which integrated MR moieties into the conjugated backbone, achieved an EQE_max_ of 17.9% with an emission peak at 479 nm and FWHM of 28 nm.^358^ Similarly, polymer PCzBNx (563) utilized as nondoped emissive layers successfully achieved both high EQE_max_ and narrow FWHM simultaneously (see Figure 37).^359^

The study of N/-CO-type MR emitters is gaining momentum, encompassing a wide range of emission colors across the visible spectrum. For instance, DiKTa-LC (564) (see Figure 37), a liquid crystalline MR-TADF emitter, achieved an EQE_max_ of 13.6% in solution-processed OLEDs due to its preferential *Θ_∥_*.^360^ However, most optimized OLEDs utilizing N/-CO-type MR emitters have employed conventional binary-system emitter layers, as shown in Table S5.

Regarding N-PAHs-type MR emitters like IDCz-DPA (346)^77^ and α-NAICZ (354)^216^ (see Figure 21), traditionally functioning as fluorescent emitters without TADF characteristics, their *k*_RIS_ rates are relatively slow, with the fastest represented by p3IDCz (41) at merely 1.07 × 10^4^ s^–1^.^80^ Therefore, binary-system-based N-PAHs emitters typically rely on TTA-type hosts such as α-AND for IDCz-DPA (346) and IDCz-2DPA (347),^77^ as well as α,β-AND for pSFIAc1 (413) and pSFIAc2 (414)^244^ (see Figure 25) to boost the exciton utilization efficiency.^361^

The binary-system emitting layer represents an attractive approach for commercial applications due to its cost-effectiveness, despite challenges such as efficiency roll-off and stability issues. To optimize MR-emitter-based binary-system OLEDs, rapid triplet exciton consumption is crucial, as discussed in Section 5.2 (“Acceleration of Spin-Flipping RISC”). From a device engineering perspective, selecting an appropriate host material—particularly a customized host compatible with both MR emitters and ACQ-resistant TADF materials—is essential for extending the device’s lifetime. Such hosts serve dual functions as both matrix and sensitizer, a concept that will be further elaborated in Section 7.3.4.

### 7.3 Ternary-System Emitter Layer

Currently, there are two primary configurations for MR-emitter devices: conventional binary-system and emerging ternary-system emitter layers. In binary-system OLEDs, a confined exciton recombination zone and low radiative decay lead to the accumulation of triplets, triggering exciton–exciton annihilation and triplet–polariton annihilation. Furthermore, electroluminescent MR-TADF compounds, influenced by the Faradaic yield of oxidation, undergo radical cationic formation.^362^ These processes result in detrimental exciton losses and significant efficiency roll-off in OLEDs. Beyond the boosting of the RISC process from the perspective of intrinsic emitter optimization, multicomponent systems, such as ternary-system emitter layers, offer a broader exciton recombination zone compared to the single-host systems. This results in superior performance in terms of high efficiency, minimal roll-off, and enhanced device durability, despite the potential complexity of the processes and the requirement for advanced vacuum deposition equipment. In ternary-system setups, co-hosts enable balanced charge transport and energy configuration modulation, outperforming their single-host counterparts. For instance, gold­(I)-coordinated MR emitters like (BzIPr)­AuBN (472), doped in co-hosts of DMIC-TRz (565) and DMIC-Cz (566) (see Figure S1 for the molecular structure), achieved a high luminance of up to 2.53 × 10^5^ cd m^–2^, an EQE_max_ of 30.3%, minimal roll-offs of 0.8%, and long device lifetimes (LT_60_) of 1210 h at an initial luminance of 1000 cd m^–2^.^297^ It is also worth noting that ternary systems can also facilitate white OLEDs through complementary hue emitters. For instance, DPMX-CzDABNA (567) as a blue-hazard-free component with blue narrow-band emission, and the conventional TADF emitter BPPZ-DPXZ (568) (see Figure 39 for the molecular structure) as an orange counterpart, can be combined to realize white OLEDs based on a single-emitting layer with human-eye-friendly emission spectra.^363^ Optimized devices based on MR emitters are detailed in Tables S4–S6.

#### 7.3.1 TADF Materials as Sensitizers

While progress has been made in achieving narrow emission bandwidth and high efficiency in OLEDs with binary-system emitter layers, challenges persist in suppressing ACQ, reducing efficiency roll-off, and increasing device stability. Ternary-system emitter layer-based device engineering, particularly utilizing hyperfluorescence (HF) technology (denoted as HF-OLEDs), presents a promising solution to these challenges.

HF technology leverages TADF-sensitized fluorescence (TSF) or phosphor-sensitized fluorescence (PSF) to harvest triplet excitons and transfer singlet excitons to the terminal fluorescent emitter via Förster energy transfer (FRET, illustrated in Figure 38).^365^ This mechanism has been significantly advanced due to its numerous attractive characteristics, including high efficiency, narrow emission bandwidth, and prolonged device lifetime. To be suitable for an HF system, several requirements must be met: (1) The host and TADF assistant dopant should have higher S_1_ and T_1_ energy to confine the high-energy excitons of the terminal MR emitters. (2) The emission spectrum of the sensitizer should overlap with the absorbance of the terminal MR emitters to induce FRET energy transfer, which is expected to have an exceptionally high FRET rate (*k*_FRET_). (3) The triplet exciton decay lifetime should be short to avoid Dexter energy transfer (DET) from the sensitizer to the terminal MR emitters. The long-range FRET from the sensitizer to the terminal emitter, governing both the efficiency and rate of exciton consumption, is crucial in the sensitization process.

**Figure 38:**
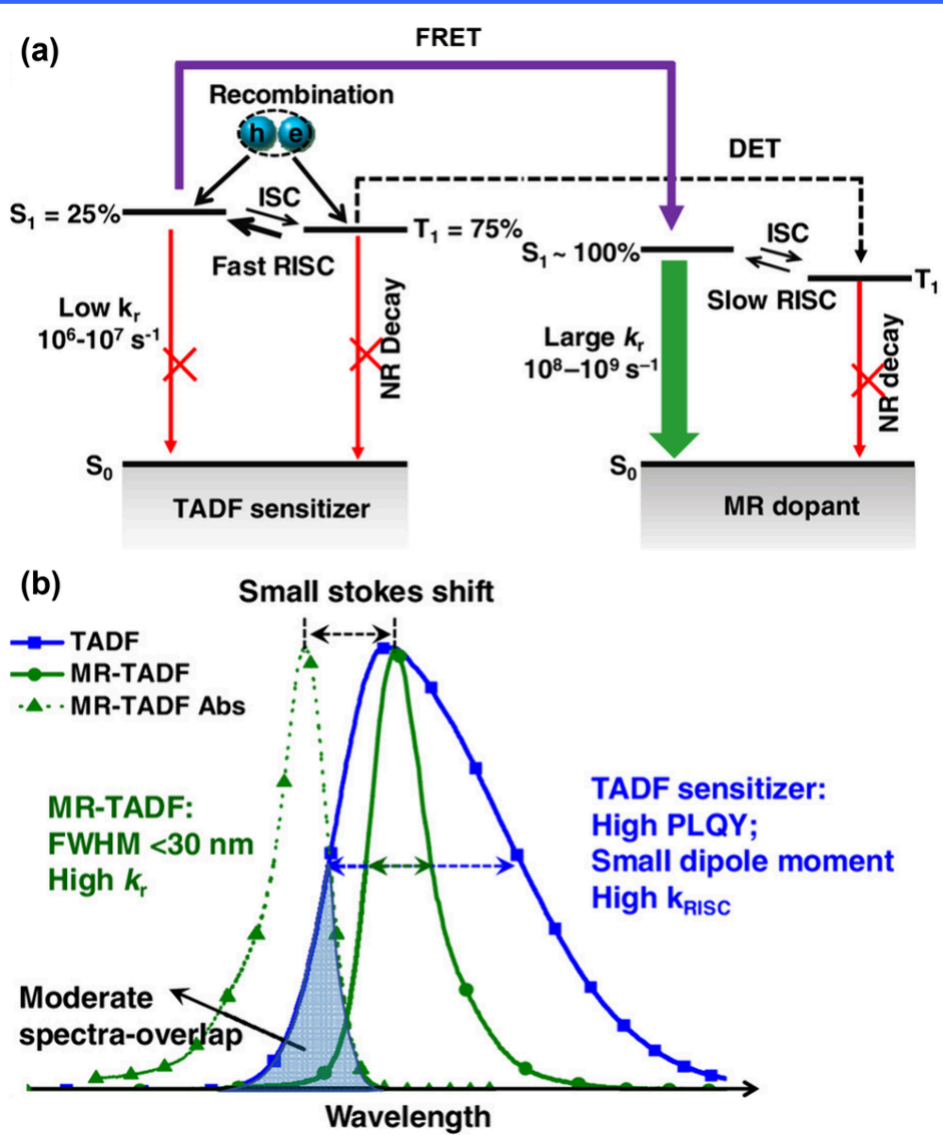
(a) The energy transfer process in (a) TADF-sensitized MR emitters (FRET: Förster energy transfer, DET: Dexter energy transfer). (b) The diagram of absorption (dashed green lines) and emission (solid green lines) of MR dopant and the emission spectra of sensitizer (blue). The shaded area indicates the spectral overlap of sensitizer emission and MR-dopant absorption.^364^ Reproduced with permission from ref 364. Copyright, 2020, Chinese Chemical Society.

In TSF devices, although TADF sensitizers generally possess larger *k*_r_ (≈10^6^–10^7^ s-^1^) and higher *k*_FRET_ values, the RISC process can still hinder overall energy transfer efficiency, as many excitons inevitably undergo multiple singlet–triplet spin-flip cycles before transferring energy to the terminal emitter. Conversely, in PSF devices, the FRET is typically constrained to the range of 10^5^–10^6^ s^–1^ due to the relatively low radiative decay rate (*k*_r_ ≈ 10^5^–10^6^ s^–1^) of phosphors. Ideally, FRET from the TADF sensitizer to the terminal MR emitters, without any DET, would harvest only singlet excitons of the MR emitters. This approach addresses efficiency roll-off issues while preserving color purity and high EQE. Bulky units are often incorporated to hinder DET, ensuring that triplet excitons on the sensitizer are rapidly upconverted to singlet excitons and then via long-range FRET to the MR emitter, resulting in narrowband emission.^366^ Optimized OLEDs utilizing TADF-sensitized MR emitters can reduce triplet accumulation, thereby improving device durability. This approach has yielded impressive performance, including EQE_max_ values exceeding 30% and narrow emission bands across various color regions such as blue,^340,367^ green,^364^ yellow,^143^ and red.^132^ Some custom-designed conventional TADF materials for MR emitters have achieved unprecedented advances in HF-OLEDs. For example, using TADF materials pMDBA-DI (569) or mMDBA-DI (570) as sensitizers and pure blue MR emitter *t*-Bu-ν-DABNA (571) as a terminal emitter, HF-OLEDs based on DBFPO (572) (also known as PPF) host exhibited EQE_max_ of 39.1% and narrow emission with an FWHW of 19 nm (CIE_*y*_ = 0.15) (the related molecular structures refer to Figure 39).^262^ A series of new sensitizers, including CTPCF3 (573), CNCTPCF3 (574), and TCTPCF3 (575), feature *ortho*-arranged donor–acceptors on a (trifluoromethyl) benzene linker, demonstrated small molecular dipole moments and fast *k*_RISC_ owing to through-bond and through-space charge transfers. Despite CTPCF3 (573) having inferior emission spectral overlap with the absorption of the green 2F-BN (179) dopant, devices based on these sensitizers achieved a high EQE_max_ of 33.1% with an FWHM of 28 nm, thanks to the contribution of fast *k*_*RISC*_.^364^ Notably, BN-DICz (202, vide supra) demonstrated an EQE_max_ of up to 37.4% and an FWHM of merely 23 nm, representing exceptional performance for yellow electroluminescence.^143^

**Figure 39:**
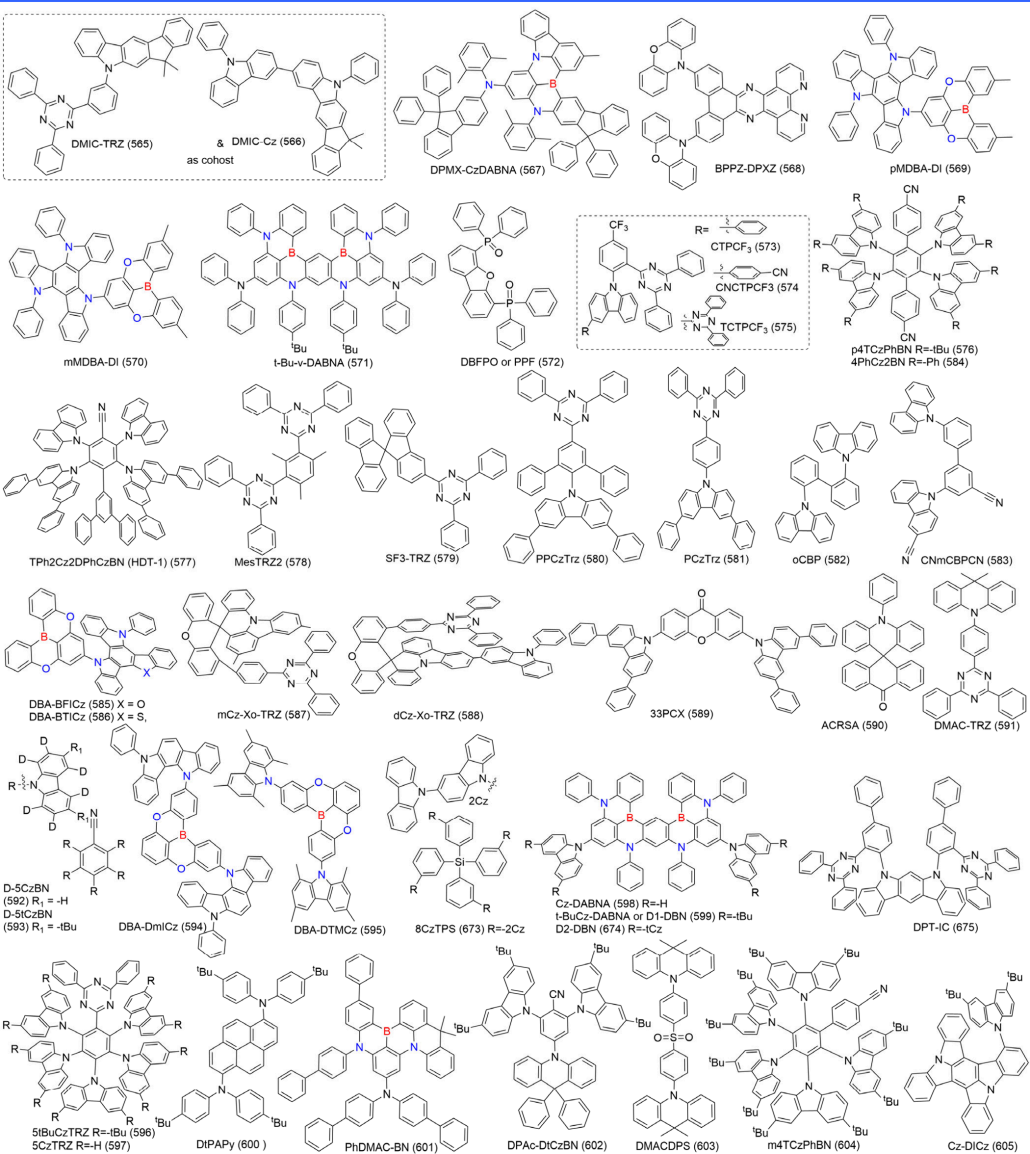
Molecular structures (No. 565–605) referred to in the above text.

The quest for highly efficient and long-lasting deep blue OLEDs has been a persistent challenge and a top priority. As a result, significant research efforts have focused on utilizing TADF sensitizers in combination with classic terminal MR emitters like t-DABNA (151) and ν-DABNA (11) to meet practical standards. One promising approach involves designing carbazole-benzonitrile derivatives, aimed at modulating delocalized excited states through a synergistic effect between charge-transfer and locally excited states.^369,370^ Among these derivatives, p4TCzPhBN (576), with linear D-π-D and A-π-A structures, stood out with a remarkably high *k*_RISC_ of 2.36 × 10^6^ s^–1^ and an emission peak at 456 nm, which perfectly aligns with the absorption spectrum of t-DABNA (151). Consequently, devices incorporating p4TCzPhBN (576) as a sensitizer for t-DABNA (151) achieved an EQE_max_ of 32.5% with a narrow FWHM of 29 nm. Moreover, these devices demonstrated prolonged operational stability, with an LT_80_ of 60 h (referred to Table 1 for HF-OLEDs data based on terminal emitter t-DABNA (151)).^345,387^

Given its exceptional performance, device engineering centered on ν-DABNA (11) as the terminal emitter has garnered significant attention and achieved remarkable advances in its operational lifetime (referred to Table 2 for HF-OLEDs data based on terminal emitter ν-DABNA (11)). For instance, HDT-1 (577), developed by Chan et al., featuring a bulky *m*-terphenyl unit at the *para*-position of the cyano group and heterodonor-type carbazole as donors, exhibited sky-blue light peaking at 485 nm with Φ_PL_ exceeding 90% and a fast *k*_RISC_ approaching 10^6^ s^–1^. Consequently, HF-OLED based on HDT-1 (577) achieved impressive results, including a FRET efficiency of 64%, an EQE_max_ of 27%, a narrow FWHM of 18 nm, and a device lifetime LT_95_ (an initial luminance of 1000 cd m^–2^) of up to 11 h.^340^ Further improvements in device performance based on HDT-1 (577): ν-DABNA (11) combination was achieved using MesTRZ2 (578), an electron transport material incorporating two triazine units nearby, as the hole-blocking and electron-transporting layer (ETL). As a result, the HF-OLED with MesTRZ2 (578) as the ETL exhibited a 4.5-fold increase in LT_50_ compared to the control device using SF3-TRZ (579).^371^ Furthermore, a quaternary system comprising a mixed host with a TADF material sensitizing the terminal MR emitter demonstrated the simultaneous realization of high efficiency and extended device stability. Jeon et al. introduced an innovative triplet-exciton-distributed (TED) device concept, wherein the T_1_ energy of the host is lower than that of the sensitizer to enhance device stability, yet higher than that of ν-DABNA (11) to maintain high efficiency. Specifically, TED devices with PPCzTrz (580) or PCzTrz (581) as the TADF sensitizer, and oCBP (582): CNmCBPCN (583) as mixed hosts achieved EQE_max_ values of 33.0 ± 0.3% and 33.5 ± 0.1%, with LT_50_ of 151 and 113 h at an initial luminance of 1000 cd m^–2^, respectively (Figure 40).^367^ Subsequently, Duan and colleagues further elaborated in detail on Chan and Jeon’s work.^368^

**Figure 40:**
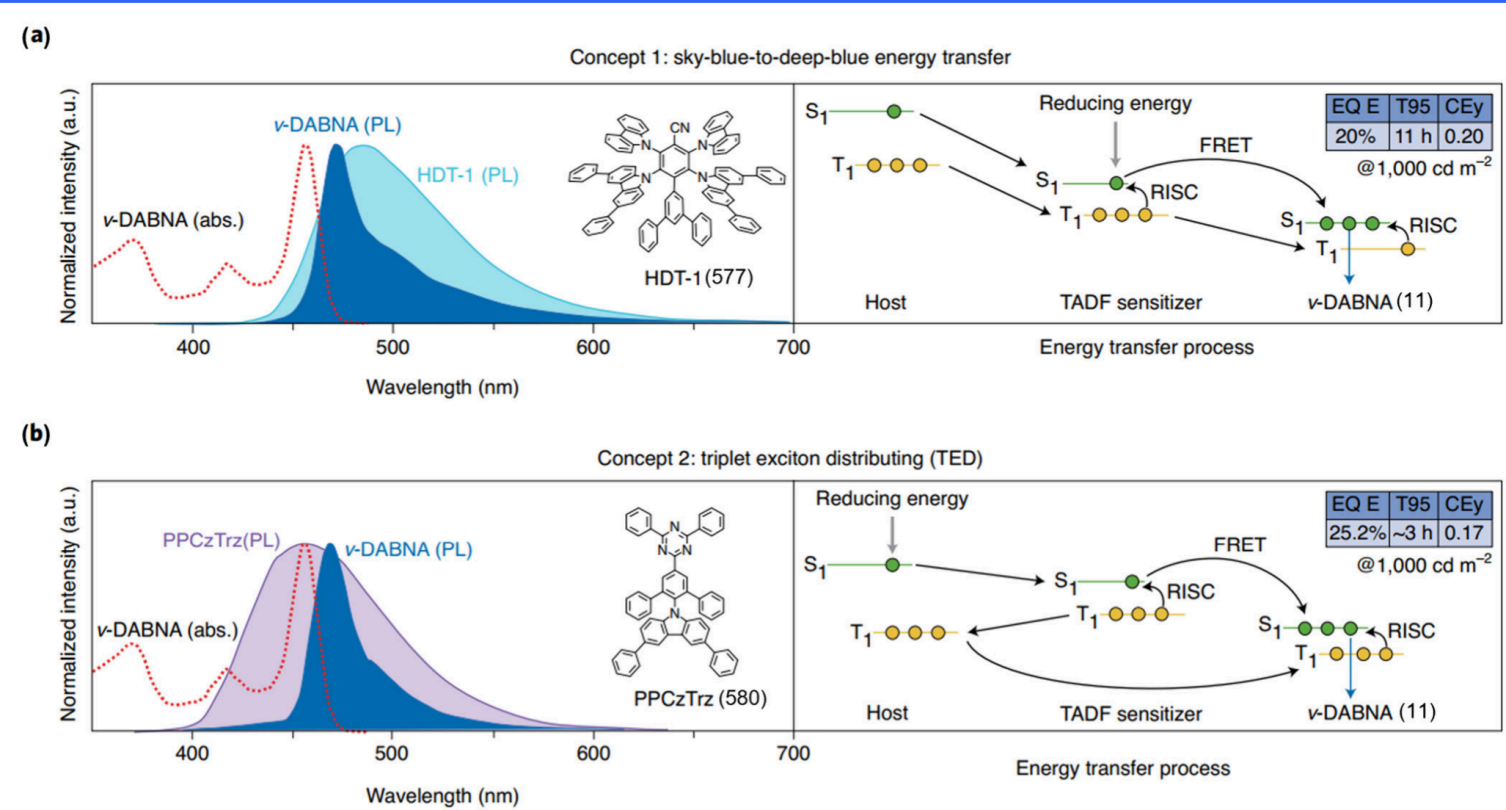
Comparison of photophysical characteristics and emission mechanisms. (a) Comparison for Chan et al.’s work.^340^ (b) Comparison for Jeon et al.’s work.^367^ Insets show the chemical structures of the sensitizers (HDT-1 (577) and PPCzTrz (580)).^368^ Reproduced with permission from ref 368. Copyright, 2021, Springer Nature.

**Table 2:** Device Performance of Organic Light-Emitting Diodes (OLEDs) with ν-DABNA (11)-Doped EML

Sensitizer	Voltage [v]^a^	EQE [%]^b^	CE [cd A^–1^]^c^	PE [lm W^–1^]^d^	λ_EL_/FWHM[nm]^e^	CIE_(*x*,*y*)_ ^f^	LT_90_ (h)^g^	Ref.
HDT-1[Table-fn t2fn11]	3.0	27/24/20	39/36/31	41/26/16	470/18	0.15, 0.20	11^h^	^340^
HDT-1[Table-fn t2fn12]	6.5	41/39/32	72/70/59	23/16/10	470/19	0.13, 0.16	18^h^	
HDT-1[Table-fn t2fn13]	2.8	28.1/24.6/18.8			471	0.14, 0.20	LT_50_ = 41 (1000 cd/m^2^)	^371^
HDT-1[Table-fn t2fn14]	2.8	26.1/24.9/20.9			471	0.15, 0.21	LT_50_ = 188 (1000 cd/m^2^)	
PPCzTrz[Table-fn t2fn15]		33.0/-/25.2	39.7/-/28.9	-		0.13, 0.20	151^i^	^367^
PCzTrz[Table-fn t2fn15]		33.5/-/23.8	35.5/-24.3	-		0.12, 0.18	113^i^	
4PhCz2BN[Table-fn t2fn16]	3.2	22.4/21.2/17.8	25.4/-/19.1	25.0/-/9.4	470/18	0.13, 0.15	7.5	^372^
dCz-Xo-TRZ[Table-fn t2fn17]	-	34.7/33.5/27.6	37.6	35.8	471/19	0.13, 0.15	-	^373^
mCz-Xo-TRZ[Table-fn t2fn17]	-	27.2/24.5/19.2	27.5	24.1	471/19	0.12, 0.14	-	
23PCX[Table-fn t2fn18]		25.1/21.6/23.4		40.4/38.9/29.9	471/24	0.149, 0.241	LT_95_ = 15 (1000 cd/m^2^)	^374^
33PCX[Table-fn t2fn18]		20.6/13.1/20.2		24.1/19.3/21.7	471/21	0.140, 0.195	LT_95_ = 13 (1000 cd/m^2^)	
TMDMAcTOX[Table-fn t2fn19]		22.2%						^375^
ACRSA[Table-fn t2fn20]	2.9	28.5/24.6/18.6	37/33/25	36/22/12	473/19	0.13, 0.19	LT_50_ = 69 min (2500 cd/m^2^)	^376^
DMAC-TRZ[Table-fn t2fn20]	2.9	23.5/22.7/18.3	49/47/38	41/31/18	474/23	0.17, 0.32	LT_50_ = 18.5 (1500 cd/m^2^)	
AZB-TRZ[Table-fn t2fn20]	3.3	19.6/16.3/12.5	30/26/20	27/16/9	473/19	0.14, 0.22		^261^
DBA-BFICz[Table-fn t2fn21]	3.1	38.8/-/29.1	30.0/-/22.5		473/19	0.12, 0.15		
DBA-BTICz[Table-fn t2fn21]	3.1	37.3/-/27.5	28.3/-/21.4		473/19	0.12, 0.15		
5Cz-BO[Table-fn t2fn22]	3.81	33.1	29.5	24.3	468/18	0.125, 0.112		^267^
D-5CzBN[Table-fn t2fn23]		29.2/-/24.1		34.3/-/17.1	468/19	0.14, 0.14	LT_80_ = 398 (1000 cd/m^2^)	^377^
D-5tCzBN[Table-fn t2fn23]		33.1/-/29.0		52.9/-/26.3	468/20	0.15, 0.20	LT_80_ = 1365 (1000 cd/m^2^)	
DBA-DmICz[Table-fn t2fn24]	3.2	36.5/-/31.7	42.5/-/38.3		473/21	0.13, 0.22		^378^
DBA-DTMCz[Table-fn t2fn24]	3.2	42.2/-/35.9	40.1/-/34.8		473/21	0.12, 0.16		
4TDTBN[Table-fn t2fn25]	3.0	25.4/-/22.2			467/17	0.13, 0.10	LT_80_ = 81.5 (500 cd/m^2^)	^379^
4TCzBN[Table-fn t2fn25]	3.1	21.5/-/16.9			467/18	0.13, 0.12	LT_80_ = 20.4 (500 cd/m^2^)	
PtON7-dtb[Table-fn t2fn26]		32.4/-/25.4	32.0/-/25.1		473/20	0.111, 0.141	LT_50_ = 156.3	^380^
CN-Ir[Table-fn t2fn27]	3.0	27.3/-/23.3	31.2/-/27.9				LT_50_ = 121 (1000 cd/m^2^)	^381^
CN-Ir[Table-fn t2fn28]			37.0/-/30.0				LT_50_ = 493 (1000 cd/m^2^)	
fct-6a[Table-fn t2fn29]	3.4	26.2/21.6/18.4	25.0/20.5/17.4	22.3/13.9/8.8	472/22	0.12, 0.13		^382^
fct-6b[Table-fn t2fn29]	3.3	25.1/20.7/17.7	23.3/19.2/16.1	20.7/13.8/8.7	472/22	0.12, 0.13		
fct-6c[Table-fn t2fn29]	3.3	25.8/21.5/17.3	25.0/20.9/17.3	23.3/15.8/9.6	472/22	0.12, 0.13		
f-ct9b[Table-fn t2fn30]	2.9	34.7/-/23.0	32.2	34.8	469/18	0.122, 0.131		^383^
Ce-2[Table-fn t2fn31]	3.1	30.0/-/25.6	30.8	27.5	471/20	0.13, 0.14		^384^
Ce-2[Table-fn t2fn32]	3.4	28.9/-/25.2					LT_50_ = 9.1 (1000 cd/m^2^)	
Cu-5[Table-fn t2fn33]	3.0	10.2/-/8.42	14.3	12.8	470/19	0.15, 0.20	LT_90_ = 12.21 (1000 cd/m^2^)	^385^
Pd-7[Table-fn t2fn34]		23.1/-/21.8	51.7		476/24	0.14, 0.24		^386^

a Voltage at 1 (turn-on voltage, *V*_on_), 1 cd m^–2^.

b EQE for maximum, and at 100 and 1000 cd m^–2^, respectively.

c Current efficiency for maximum, and at 1000 cd m^–2^.

d Power efficiency for maximum, and at 1000 cd m^–2^.

e λ_EL_: EL emission maximum, and FWHM: full width at half maximum.

f Commission Internationale de l’Éclairage color chromaticity coordinates.

g 90% of an initial luminance of 1000 cd m^–2^.

h 95% of an initial luminance of 1000 cd m^–2^.

i 50% of an initial luminance of 1000 cd m^–2^.

d1 ITO (50 nm)/ HAT-CN (10 nm)/ TAPC (20 nm)/ Tris-Pcz (10 nm)/ mCBP (5 nm)/ 0.5 wt % ν-DABNA (11):20 wt % HDT-1:mCBP (30 nm)/ SF3-TRZ (10 nm)/ SF3-TRZ: Liq (25 nm)/ Liq (2 nm)/ Al (100 nm).

d2 ITO (50 nm)/ HAT-CN (10 nm)/ TAPC (70 nm)/ Tris-Pcz (10 nm)/ mCBP (5 nm)/ 0.5 wt % ν-DABNA (11): 20 wt % HDT-1: mCBP (30 nm)/ SF3-TRZ (10 nm)/ SF3-TRZ: Liq (25 nm)/ Liq (2 nm)/ Al (1.5 nm)/ HAT-CN (10 nm)/ TAPC (20 nm)/ Tris-Pcz (10 nm)/ mCBP (5 nm)/ 0.5 wt % ν-DABNA (11): 20 wt % HDT-1:mCBP (30 nm)/ SF3-TRZ (10 nm)/ SF3-TRZ: Liq (25 nm)/ Liq (2 nm)/ Al (100 nm).

d3 ITO/HAT-CN (10 nm)/ Tris-PCz (30 nm)/ mCBP­(5 nm)/ 0.5 wt % *ν*-DABNA:20 wt % HDT-1:mCBP (30 nm)/ SF3-TRZ (10 nm)/ 30 wt % Liq: SF3-TRZ (30 nm)/ Liq (2 nm)/ Al (100 nm).

d4 ITO/HAT-CN (10 nm)/ Tris-PCz (30 nm)/ mCBP­(5 nm)/ 0.5 wt % ν-DABNA:20 wt % HDT-1:mCBP (30 nm)/ SF3-TRZ (10 nm)/ 30 wt % Liq: MesTRZ2 (30 nm)/ Liq (2 nm)/ Al (100 nm).

d5 ITO (50 nm)/ DNTPD (40 nm)/ BPBPA (10 nm)/ mCBP (10 nm)/ (10 wt % PPCzTrz or PCzTrz: 1 wt % ν-DABNA (11): 44.5 wt % oCBP and 44.5 wt % CNmCBPCN) (30 nm)/ DBFTrz (5 nm)/ ZADN (20 nm)/ LiF (1.5 nm)/ Al (200 nm).

d6 ITO/HAT-CN (10 nm)/ Tris-PCz (30 nm)/ mCBP (5 nm)/ 0.5 wt % ν-DABNA (11): 20-wt % 4PhCz2BN: mCBP (30 nm)/ SF3-TRZ (10 nm)/ 35-wt %-Liq: SF3-TRZ (30 nm)/ Liq (2 nm)/ Al (100 nm).

d7 ITO/HATCN (5 nm)/ TAPC (30 nm)/ TCTA (10 nm)/ mCP (10 nm)/ PPF: 30 wt % sensitizer: 1 wt % ν-DABNA (11) (24 nm)/ PPF (10 nm)/ BPhen (30 nm)/ LiF (0.5 nm)/ Al (150 nm).

d8 ITO/HATCN (5 nm)/ NPB (30 nm)/ TCTA (10 nm)/ mCPBC: 30 wt % sensitizers: 1 wt % ν-DABNA (30 nm)/ CzPhPy (10 nm)/ DPyPA:Liq (1:1, 30 nm)/ LiF (0.5 nm)/ Al (150 nm).

d9 ITO/NPB (40 nm)/ TSBPA­(10 nm)/ 64.5 wt % DPEPO:35 wt % TMDMAcTOX: 0.5 wt % ν-DABNA (11) (30 nm)/ TPBi (10 nm)/ LiF (0.8 nm)/ Al (150 nm).

d10 ITO/NPB (35 nm)/ NPB: mCBP­(1:1, 5 nm)/ mCBPCN: 10 wt % ACRSA: 1 wt % ν-DABNA or mCBPCN: 10 wt % DMAC-TRZ: 1 wt % v-DABNA or mCP: 10 wt % AZB-TRZ: 1 wt % v-DABNA (30 nm)/ T2T (10 nm)/ T2T:Liq (1:1, 30 nm)/ LiF (0.5 nm)/ Al (150 nm).

d11 ITO/TAPC (20 nm)/ DCDPA (10 nm)/ DBFPO: 25 wt % sensitizers: 1 wt % ν-DABNA (30 nm)/ DBFPO (10 nm)/ TPBi (120 nm)/ Al (100 nm).

d12 ITO/HAT-CN (10 nm)/ α-NPD (30 nm)/ Tris-PCz (15 nm)/ CzSi (6 nm)/ TSPO1:5Cz-BO: ν-DABNA (90 wt %: 10 wt %: 1 wt %, 20 nm)/ CF3-TRZ (10 nm)/ Liq: BPPB (25 nm, 50 wt %: 50 wt %)/ Liq (2 nm)/ Al (100 nm).

d13 ITO (50 nm)/ HAT-CN (5 nm)/ BCFN (30 nm)/ SiCzCz (5 nm)/ 55 wt % SiCzCz: 30 wt % SiTrzCz2:15 wt % sensitizer: 1 wt % ν-DABNA (11) (24 nm)/ mSiTrz (10 nm)/ DPPyA (25 nm):Liq (30 nm)/ LiF (0.5 nm)/ Al (100 nm).

d14 ITO (50 nm)/ HATCN (7 nm)/ TAPC (55 nm)/ DCDPA (10 nm)/ DBFPO: 30% of DBA-DmICz or DBA-DTMCz: 1 wt % ν-DABNA (25 nm)/ DBFPO (10 nm)/ TPBi (20 nm)/ LiF (1.5 nm)/ Al (100 nm).

d15 ITO/HATCN (5 nm)/ NPB (30 nm)/ SiCzCz (10 nm)/ SiCzCz: SiTrzCz2: TADF emitter (0.60:0.40:0.30): 1 wt % ν-DABNA (11) (40 nm)/ SiTrzCz2 (5 nm)/ DPPyA: Liq (1:1, 30 nm)/ LiF (0.5 nm)/ Al (150 nm).

d16 ITO (50 nm)/ 3 wt % NDP series doped BCFA (HIL, 10 nm)/BCFA (125 nm)/ HT series (10 nm)/ oCBP (5 nm)/ mCPD (5 nm)/ oCBP:mCBP- 2CN:PtON7-dtb: ν-DABNA (11) 50%:50%:10%:1.5%) (40 nm)/ mCP-2CN (10 nm)/ co-deposited NET series:NDN series (5:5,31 nm)/ Al (100 nm).

d17 ITO (50 nm)/ BPBPA:HAT-CN (40 nm:30 wt %)/ BPBPA (10 nm)/ mCBP (10 nm)/ 50 wt % mCBP: 50 wt % SiCz2Trz: 20 wt % CN-Ir: 0.5 wt % ν-DABNA (11) (30 nm)/ DBFTrz (5 nm)/ ZADN (20 nm)/ LiF (1.5 nm)/ Al (200 nm).

d18 ITO (50 nm)/ Ag (100 nm)/ ITO (5 nm)/ BCFN:NDP-2 (10 nm:2 wt %)/ BCFN (127 nm)/ mCBP (5 nm)/ 50 wt % mCBP: 50 wt % SiCz2Trz: 20 wt % CN-Ir: 0.5 wt % ν-DABNA (11) (30 nm)/ DBFTrz (5 nm)/ ZADN: Liq (30 nm_50:50)/ Yb (1 nm)/ Ag: Mg (13 nm).

d19 ITO/HAT-CN (10 nm)/ TAPC (40 nm)/ TCTA (10 nm)/ mCBP (10 nm)/ PPF: phosphors: ν-DABNA (11) (20 wt %, 1 wt %, 25 nm)/ TmPyPB (40 nm)/ Liq (2 nm)/ Al (100 nm).

d20 ITO/HATCN (5 nm)/ BCFN (30 nm)/ SiCzCz (10 nm)/ 65 wt % SiCzCz: 35 wt % SiTrzCz2:20 wt % fct9b: 2 wt % ν-DABNA (30 nm)/ mSiTrz (5 nm)/ DPPyA (30 nm)/ LiF (0.5 nm)/ Al (150 nm).

d21 ITO/HAT-CN (8 nm)/ HAT-CN (0.2 wt %):TAPC (40 nm)/ TAPC (10 nm)/ TCTA (5 nm)/ 1 wt % ν-DABNA (11): 10 wt % Ce-2:44.5 wt % TCTA: 44.5 wt % DPEPO (EML, 20 nm)/ Tm3PyP26PyB (60 nm)/ LiF (1 nm)/ Al (100 nm).

d22 ITO/HAT-CN (10 nm)/ BFCN (40 nm)/ SiCzCz (5 nm)/ 1 wt % ν-DABNA (11): 10 wt % Ce-2: SiCzCz (20 nm)/ mSiTrz (5 nm)/ DPPyA (30 nm)/ LiF (1 nm)/ Al (100 nm).

d23 ITO/HAT-CN (5 nm)/ TAPC (50 nm)/ TCTA (10 nm)/ mCBP: 8 wt % Cu-5 complex: 1 wt % ν-DABNA (11) (20 nm)/ HBL (10 nm)/ TPBi (40 nm)/ LiF (1 nm)/ Al (100 nm).

d24 ITO/HAT-CN (5 nm)/ TAPC (40 nm)/ CCP (10 nm)/ 10 wt % Pd-7 (678): 1 wt % ν-DABNA: PPF (10 nm)/ PPF (10 nm)/ TmPyPb (40 nm)/ LiF (1.2 nm)/ Al (100 nm).

Numerous effective strategies and advancements have been undertaken to optimize the performance of ν-DABNA (11) in HF-OLEDs. An HF-OLED utilizing 4PhCz2BN (584): ν-DABNA (11) exhibited an EQE_max_ of 22.4% with CIE coordinates (0.13, 0.15).^372^ The sensitizer 5Cz-BO (421), with its excellent TADF properties, enabled terminal emitter ν-DABNA (11) based-HF-OLED to reach an exceptional EQE_max_ of 33.1%.^267^ Two deep blue TADF materials, namely DBA-BFICz (585) and DBA-BTICz (586), incorporating B/O-triangulene acceptors with oxygen or sulfur-inserted donors, were used as TADF-sensitized hosts in bottom emission HF-OLEDs, exhibiting outstanding EQE and narrow FWHM.^261^ Blue emitters, mCz-Xo-TRZ (587) and dCz-Xo-TRZ (588) exhibited emission peaks around 460 nm with high *k*_RISC_ of nearly 10^7^ s^–1^, owing to electronic interactions facilitated by through-space charge transfer (TSCT) via space-confined xanthene bridges. Consequently, when sensitized with ν-DABNA (11), HF-OLEDs achieved EQE_max_ values of 27.8% and 34.7% with CIE_*y*_ coordinates of 0.29 and 0.15, respectively.^373^ 33PCX (589) served as an effective blue sensitizer for ν-DABNA (11), demonstrating comparable stability to champion counterparts.^374^ The molecular structure of the sensitizer is critical for the FRET efficiency. For instance, the spiro-linked TADF molecule ACRSA (590) optimized the FRET efficiency to nearly 100% by suppressing dihedral-angle inhomogeneity and any lower-energy conformers. While greenish in nature, ACRSA (590) and DMAC-TRZ (591) achieved remarkable EQEs in blue HF-OLEDs.^376^ Perdeuterated sensitizers, such as D-5CzBN (592) and D-5tCzBN (593), enhanced FRET due to their blue-shifted and narrowed spectra in the solid state. As a result, HF-OLEDs based on these perdeuterated sensitizers exhibited sharp blue emission (peaks at ∼468–469 nm) primarily from ν-DABNA (11) with reduced long-wavelength tails compared to protonated counterparts.^377^ Quadrupolar donor–acceptor–donor (D-A-D)-type TADF sensitizers, DBA-DmICz (594) and DBA-DTMCz (595), demonstrated superior performance over their bipolar D-A-type counterparts. Specifically, the HF device utilizing DBA-DTMCz (595) and ν-DABNA (11) achieved an impressive EQE_max_ of 43.9% with CIE coordinates of (0.12, 0.16). This remarkable efficiency resulted from a suppressed DET process, a high *k*_RISC_ rate, and shielding of the LUMO facilitated by the presence of two donor groups in the D-A-D molecular framework.^378^

Solution-processed OLEDs based on TADF-sensitized MR-TADF emitters have also been explored. A novel bulky TADF sensitizer, 5tBuCzTRZ (596), with its high *k*_RISC_ of 2.0 × 10^7^ s^–1^, effectively suppressed DET while facilitating long-range FRET to the DtBuCzB (5) emitter. Therefore, their solution-processed OLEDs achieved an EQE_max_ of 23.9% and maintained 21.5% at a practical luminance of 1000 cd m^–2^. Additionally, 5tBuCzTRZ (596) sensitized the green MR emitter m-Cz-BNCz (212), showcasing an improved EQE_max_ of 22.5% compared with nonsensitized devices.^388^ To tackle issues stemming from unbalanced charge transport and severe exciton quenching due to the trapped holes on higher-lying HOMO levels, Cz-DABNA (598) and *t*-BuCz-DABNA (599) (see Figure 39) were developed to mitigate hole-trapping effects. This optimization enabled a solution-processed pure-blue HF-OLED utilizing *t*-BuCz-DABNA (599) as the terminal emitter and 5CzTRZ (597) as the sensitizer to achieve a record EQE_max_ of 29.2% with an FWHM of 16.6 nm (Table S4).^389^ The solution-processed devices based on dendritic emitter D2-DBN (674) (Figure 39) doped in dendritic host 8CzTPS (673) achieved a record-breaking EQE_max_ of 35.3%, along with a narrow emission bandwidth of 17 nm and a pure blue color with CIE coordinates of (0.137, 0.176). These outstanding performances can be attributed to the high *Θ_∥_* of up to 83.0%, induced by the electrostatic interaction between 8CzTPS (673) and D2-DBN (674), as well as the exceptionally high PLQY of 98.6%, which is enhanced by the highly twisted structure of 8CzTPS (673) and the dendron encapsulation of D2-DBN (674).^390^

In general, conventional TADF-sensitized MR emitters can address the issue of slow *k*_RISC_, but research has also produced MR emitters rivaling TADF emitters in delayed fluorescence characteristics. Outstanding MR emitters have enabled HF-OLEDs to achieve high EUE, where one MR emitter sensitizes another MR terminal emitter. For instance, BNSeSe (446) exhibited short τ_DF_ and high Φ_PL_, boosting corresponding EQE_max_ to 40.5% and 39.6% for terminal emitter yellow BN3 (172)^125^ and green DtCzB-DPTRZ (185),^131^ respectively (ref Figure 10) with minimal roll-off efficiency.^281^ Deep-blue 3TPAB (154) was selected as a sensitizer for both blue traditional fluorescence DtPAPy (600) and MR emitter PhDMAC-BN (601), leading to an enhanced EQE_max_ of 14.4% and 33.9%, respectively.^391^

N-PAHs-type MR emitters, featuring exceptional color purity, have successfully addressed the challenge of recycling electrically formed triplet excitons in state-of-the-art HF-OLEDs. For instance, although pICz (85) and pICz-TPA (86) exhibit non-TADF characteristics with nanosecond-scale lifetimes, an unprecedentedly high EQE_max_ of over 30% with CIE_*y*_ ≤ 0.10 was achieved in HF-OLEDs based on deep blue sensitizer DPAc-DtCzBN (602).^79^ Similarly, p3IDCz (41), with DMACDPS (603) as a sensitizer, demonstrated improved efficiency roll-off compared to the pristine p3IDCz (41) device.^80^ DiICzMes4 (352), upon doped in conventional devices, suffered from low efficiency and severe roll-off; however, HF-OLEDs effectively resolved this issue, achieving an EQE_max_ of up to 16.5%, more than four times higher than the free-sensitizer counterpart.^210^ Spiro-configured pSFIAc1 (413) and pSFIAc2 (414) emitters exhibited impressive stability, with remarkably long LT_80_ (initial luminance of 100 cd m^–2^) of 18,900 and 43,470 h, respectively, in TTA mechanism-driven devices. Despite the boosted EQE_max_ in HF-OLEDs significant efficiency roll-off was observed, primarily due to the relatively shallow LUMO levels of the terminal emitters compared to the sensitizer m4TCzPhBN (604).^244^ Additionally, Cz-DICz (605), featuring DICz (also named mDICz) (4) as the core and tCz as the peripheral substitution, achieved balanced bathochromic-shift emission, spectral narrowing, and aggregation suppression, with EQE_max_ of 22.1%–25.6% and consistently narrow FWHMs of 18 nm across a practical mass-production concentration range (1–4 wt %) in m4TCzPhBN (604)-based HF OLEDs.^13^

#### 7.3.2 Metal Complexes as Sensitizers

TADF sensitizers have played a pivotal role in addressing the shortcomings of MR emitters, particularly their short operational lifespan in devices. Recently, HF technology based on phosphor-sensitized fluorescence (PSF) in OLEDs has emerged (see Figure 41 for the energy transfer mechanism), harnessing the shorter triplet exciton decay lifetime of phosphors to enhance the device’s performance.

**Figure 41:**
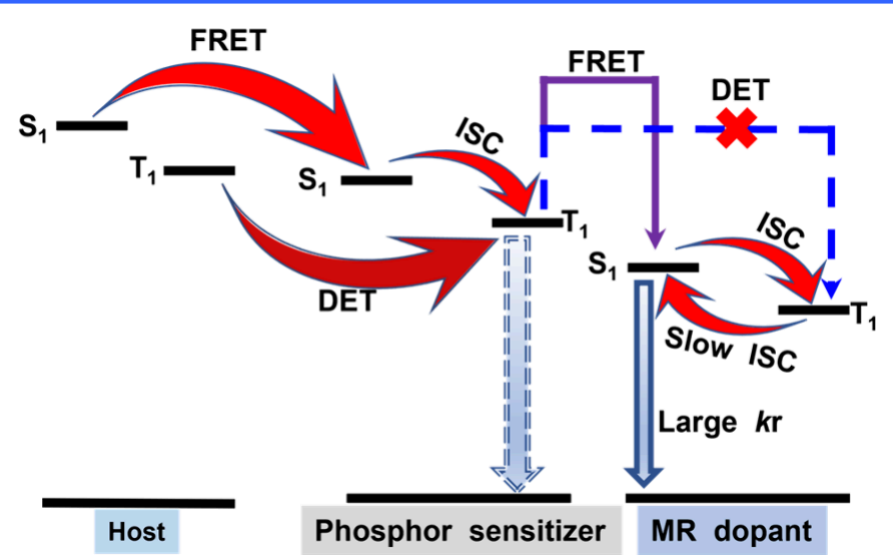
Energy transfer process in phosphor-sensitized MR emitters (FRET: Förster energy transfer, DET: Dexter energy transfer).

Phosphors, particularly noble iridium and platinum complexes, have shown significant promise in HF-OLEDs. Ir­(III) carbene complexes are especially effective as blue PSF, owing to their high stability, superior emission efficiency, and short radiative lifetime. For instance, the phosphor sensitizers like m-tz2 (606), Ir­(cb)_3_ (607), and f-tpb1 (608) could facilitate the terminal emitter t-DABNA (151) to exhibit high EQE_max_, alleviate efficiency roll-off and extend device lifetime compared to binary systems (see molecular structure in Figure 42; data of HF-OLEDs based on t-DABNA (151) are summarized in Table 1).^346–348^ A series of Ir­(III)-based carbene complexes with asymmetric chelates like fct-6a (609), fct-6b (610), and fct-6c (611) demonstrated effective energy transfer to ν-DABNA (11), resulting in a considerably high EQE_max_.^382,392,393^ Notably, PSF-OLED based on f-ct9b (612) and ν-DABNA (11) attained a blue emission of 469 nm and an EQE_max_ of up to 34.7% (see Table 2).^383^ Choi et al. demonstrated highly efficient and stable PSF-OLEDs using PtON7-dtb (613) as the sensitizer and ν-DABNA (11) as the terminal emitter, achieving an improved EQE_max_ of 32.2% and an extended device lifetime (LT_50_ = 156.3 h at an initial luminance of 1000 cd m^–2^) (see Table 2 for data of HF-OLEDs based on ν-DABNA (11)).^380^

**Figure 42:**
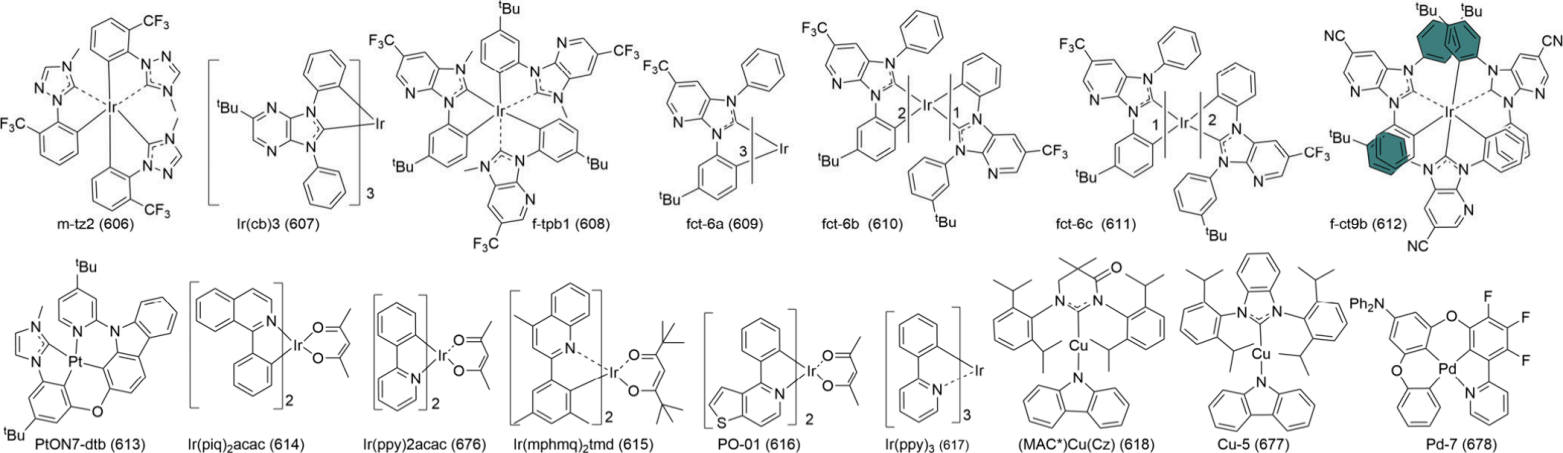
Molecular structures of organometallic sensitizer.

In addition to the aforementioned newly custom-designed phosphor sensitizers for deep-blue MR emitters, green and red phosphors also play a vital role in optimizing terminal MR emitters. For instance, BNNO (234), sensitized by Ir­(piq)_2_ acac (614) for triplet exciton recycling, achieved BT.2020 red electroluminescence for the first time, with an impressive EQE_max_ of 34.4% and an exceptionally long LT_95_ exceeding 10,000 h.^159^ A pure-red PSF-OLED, employing Ir­(mphmq)_2_ tmd (615) with CzIDBNO (236), achieved an EQE_max_ of 32.5% with CIE coordinates of (0.701, 0.298) approaching the BT.2020 standard.^160^ Terminal emitters BNO1 (231), BNO2 (232), and BNO3 (233) with PO-01 (616) as the sensitizer, achieved state-of-the-art performance metrics, including EQE_max_ exceeding 36%, ultrahigh brightness levels over 130,000 cd/m^2^, and significantly enhanced device stability.^136^ Thanks to singlet harvest through long-range FRET from Ir­(ppy)_3_ (617) to tCzphB-Ph (195) and tCzphB-Fl (196), the resultant PSF-OLEDs exhibited a sharp green emission with CIE_*y*_ > 0.71 and significantly improved EQE_max_ of 31.3% and 29.7%, respectively.^139^ Notably, a PSF-OLED, using Ir­(ppy)_3_ (617) phosphor to sensitize pure-green emitter AZA-BN (130), achieved an EQE_max_ of 28.2%, a record of PE_max_ of 121.7 lm W^–1^, and an excellent operational duration of 46.3 h (initial brightness of 2000 cd m^–2^).^103^

Multiple sensitizations through synergistic TSF and PSF, namely phosphor-assisted TADF-sensitized fluorescence (pTSF), can greatly accelerate the exciton consumption to achieve a 100% EUE with decay times in the submicrosecond regime, thus suppressing the EQE roll-off caused by exciton annihilation under high brightness. TADF sensitizing-host DPT-IC (675) possesses fast reverse intersystem crossing, an anti-ACQ character and excellent bipolar charge-transporting ability. Therefore, pTSF-OLEDs based on DPT-IC (675):Ir­(ppy)_2_ acac (676):tCzphB-Fl (196) achieved PE_max_ of 187.7 lm/W, and an exceptionally high critical maximum luminance exceeding 110,000 cd/m^2^. This breakthrough strategy demonstrates the potential of OLEDs to achieve high power efficiency even at high luminance.^394^

Overall, these advancements underscore the versatility and potential of phosphor-based sensitizers in pushing the boundaries of HF-OLED performance

#### 7.3.3 Exciplexes-Type TADF as Sensitizers

In the realm of TADF materials development, heterogeneous systems based on exciplex-type TADF materials have significantly advanced the exploration of highly efficient emitters.^395^ Donor–acceptor-based exciplex-type TADF materials have proven crucial for sensitization-type devices, serving as both exciplex hosts and sensitizers for MR emitters. They enhance device longevity by reducing triplet exciton concentration and improving charge balance through the opening of multiple reverse intersystem crossing (RISC) channels, thereby slowing the degradation and aging of MR emitters.^396,397^ Consequently, the resulting device exhibits lower driving voltages, balanced charge transport, and near-complete exciton harvesting.^398^ Interlays between mCBP (619) and PO-T2T (538) demonstrated clear TADF characteristics, making them preferred hosts for boosting MR terminals in OLEDs via efficient FRET (see Figure 43 for the molecular structures). For instance, MR emitter BN3 (172) doped in exciplex-hosts mCBP (619) and PO-T2T (538) displayed an emission peak at 568 nm with a narrow FWHM of 42 nm and an impressive EQE_max_ of up to 24.7%.^125^

**Figure 43:**
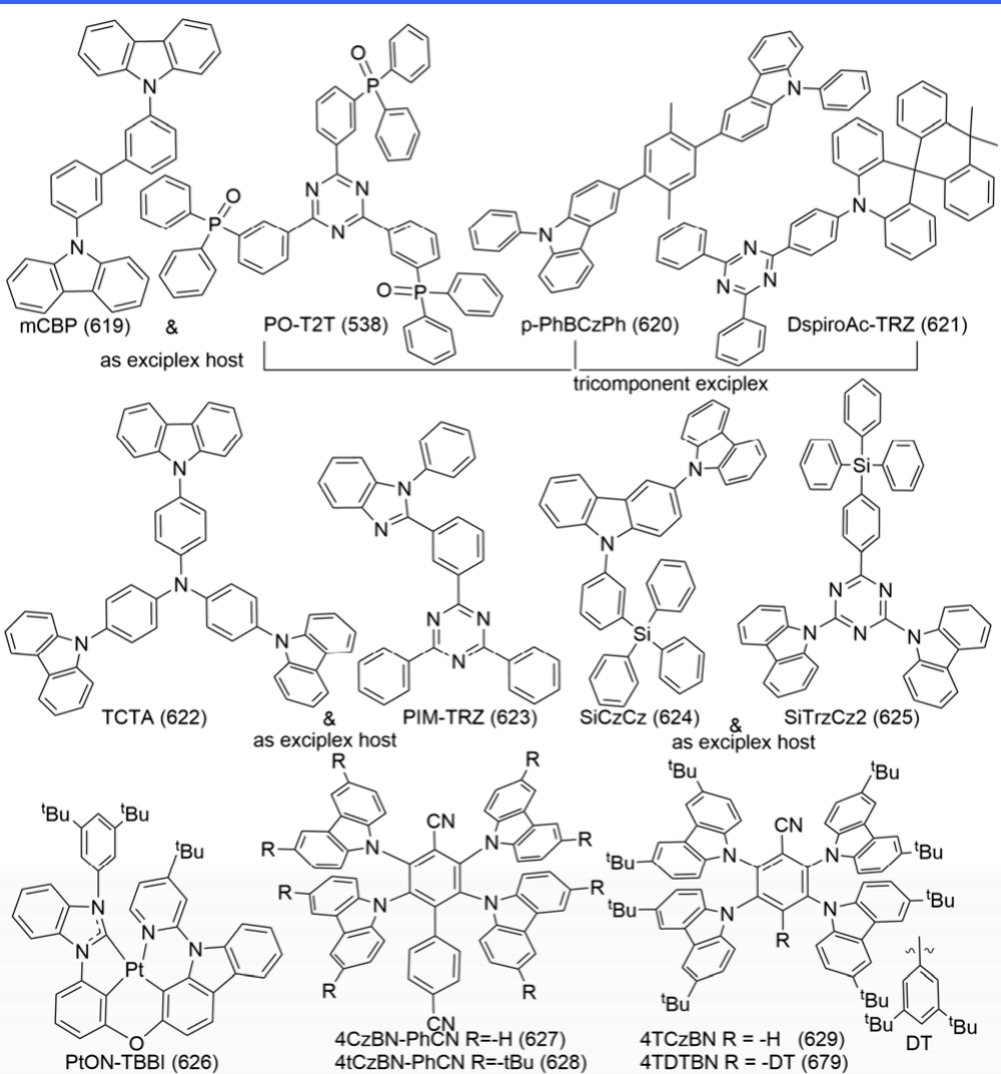
Some molecular structures in the above text.

Organometallic complexes, such as Pd­(II)-, Au­(III)-, and Cu­(I)-based emitters, typically exhibited TADF properties, and some of them performed well in HF-OLED applications.^399^ Luminescent carbene-Cu­(I)-amide complexes, with their high PLQYs and submicrosecond decay lifetimes, offered a promising alternative to noble metal-based phosphors in PSF-OLEDs. Employing (MAC*)­Cu­(Cz) (618) as a sensitizer for BN3 (172) achieved electroluminescent peak at 566 nm with an EQE_max_ of 26.5% and an FWHM of 46 nm, demonstrating superior efficiency, reduced roll-off, and enhanced operational stability over TSF-OLED.^400^ HF-OLED, utilizing robust Cu-5 (677) as a sensitizer and ν-DABNA (11) as an emitter, exhibited a moderate EQE and a narrow FWHM.^385^ A palladium­(II) complex, such as Pd-7 (678) sensitized ν-DABNA (11), achieved an EQE_max_ of 23.1% and an FWHM of 24 nm (Table 2).^386^ Although Au­(III) complexes incorporating an MR core, such as (BzIPr)­AuBN (472) and (BzIPr)­AuBNO (473), exhibit narrow emission bandwidths, other Au­(III) complexes are rarely used in HF-OLEDs.^297,399^ Green emitters like 2PXZBN (105) and 2PTZBN (106) doped in mCBP (619) and PO-T2T (538) films exhibited EQE_max_ values of 17.7% and 25.5%, respectively, benefiting from the exciplex-induced balanced carrier transport and lowered energy injecting barrier for both holes and electrons.^94^ Similarly, green emitters BN-DMAC (103) and BN-DPAC (104) demonstrated boosted device performance with EQE_max_ exceeding 30% and operational LT_80_ of up to 82 h under initial luminance of 500 cd m^–2^.^401^

Currently, exciplex-TADF hosts have been paying more attention to HF-OLEDs. For instance, the exciplex-TADF hosts TCTA (622) and PIM-TRZ (623) supported the terminal emitter DtBuPhCzB (120), achieving green electroluminescence with an EQE_max_ of up to 25.5% and a narrow FWHM of 33 nm.^99^ Moreover, a highly efficient tricomponent exciplex (p-PhBCzPh (620):PO-T2T (538):DspiroAc-TRZ (621)) with multiple RISC channels enabled sky-blue BCz-BN (5) and pure-green BN-TP (123)-based OLEDs to achieve remarkable EQEs of 36.2% and 40.3% with ultralow efficiency roll-off, respectively.^402^

Additionally, novel exciplex-TADF hosts combined with TADF or phosphor sensitizers have been actively explored in HF-OLEDs. To simultaneously achieve high efficiency and long device lifetime, a well-structured emitting layer combining stable exciplex hosts (SiCzCz (624):SiTrzCz2 (625)), phosphor sensitizer (PtON-TBBI (626)), and MR-terminal emitter (TBE01 (161) or TBE02 (162)) was realized (see Figure 10 for the molecular structures). This configuration achieved an EQ_max_ of 25.8% and LT_95_ of 72.9 h at an initial luminance of 1000 cd m^–2^ in a bottom-emissive device.^121^ A deep-blue HF-OLED using SiCzCz (624): SiTrzCz2 (625) as exciplex hosts, 4CzBN-PhCN (627) or 4tCzBN-PhCN (628) as sensitizers and *t*-BuCz-DABNA (599) as the terminal emitter achieved a remarkable LT_95_ of 221 and 454 h, respectively, at an initial luminance of 1,000 cd m^–2^ (molecular structure cf. Figure 43).^403^ An HF-OLED employing an emitting layer configuration comprising SiCzCz (624):SiTrzCz2 (625):4TCzBN (629):TB-PB (27) in ratio of 4 wt %:29 wt %:16 wt %:1 wt % achieved an EQE_max_ of 36.4%, an ultranarrow FWHM of 15 nm, a record-high luminescence of 9.0 × 10^4^ cd m^–2^, and a CIE_*y*_ coordinate of 0.20 (see Table S4).^39^ Owing to the higher C–N bond dissociation energy and enhanced molecular rigidity of 4TDTBN (679) compared to 4TCzBN (629), HF-OLEDs based on SiCzCz (624):SiTrzCz2 (625):4TCzBN (629):ν-DABNA (11) displayed higher efficiency, lower efficiency roll-off, and longer device lifetime than the corresponding 4TCzBN (629)-based devices (see Table 2).^379^ Sym-OBOICz (645) and asym-OBOICz (646), when doped in a film comprising the exciplex host SiCzCz (624):SiTrzCz_2_ (625) and the TADF assistant m4TCzPhBN (604), exhibited high LT_90_ values of 52.1 and 70.6 h at an initial luminance of 2,000 cd m^–2^, respectively.^85^

Both TSF- and PSF-based HF-technologies rely on closed-shell sensitizers, which are inevitably constrained by spin statistical limitations and forbidden transitions. To overcome the limitations of spin statistics in TSF and PSF systems, a new sensitization strategy, termed doublet-sensitized fluorescence (DSF), has been proposed. In this approach, a doublet-emitting cerium­(III) complex (Ce-2 (630)) serves as the sensitizer, while TCTA (622) and DPEPO (631) function as the exciplex-type cohosts and ν-DABNA (11) as the terminal emitter. The DSF mechanism demonstrates that holes and electrons predominantly recombine on Ce-2 (630), forming doublet excitons, which then transfer energy to the singlet state of ν-DABNA (11) via an exceptionally fast (>10^8^ s^–1^) and highly efficient (≈100%) FRET process (see Figure 44), and a notably short exciton residence time of 1.36 μs, circumventing the need for spin-flip processes.^384^

**Figure 44:**
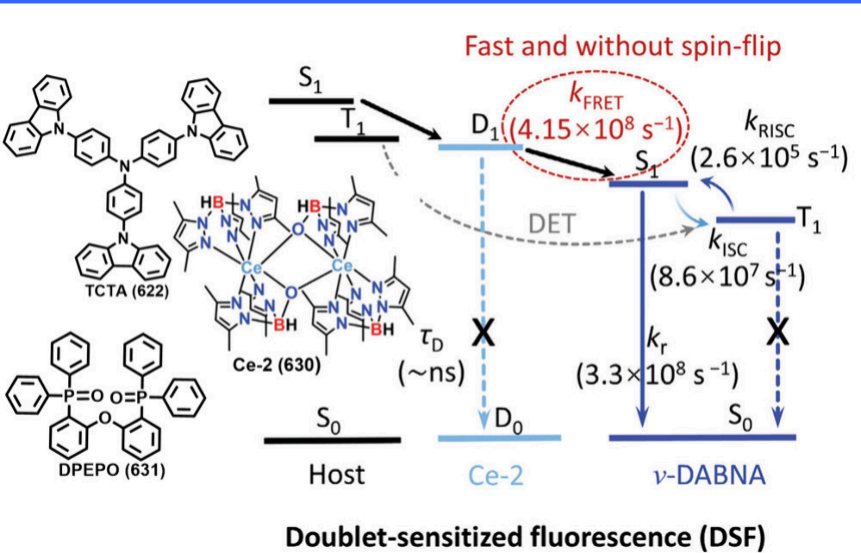
Doublet-sensitized fluorescence (DSF) mechanism (upon photoexcitation) with specific dynamic rates.^384^ Reproduced with permission from ref 384. Copyright, 2024, John Wiley and Sons.

#### 7.3.4 Exciton-Managing in HF Devices

HF technology has emerged as an effective strategy for mitigating device decay in OLEDs, particularly through the integration of triplet exciton managers (TEMs), which are pivotal for optimizing device engineering. The aforementioned TED, in a sense, is a pattern of TEMs.^367^ TEMs can be designed to lower the triplet energy of the host material below that of the MR emitters, enabling quenching of triplet excitons while harvesting singlet excitons. A notable implementation of this concept involves the host material DPBCz (632), which has a triplet energy lower than t-DABNA (151) but higher than the yellow phosphor PO-01 (616). In white organic light-emitting diodes (WOLEDs), this TEMs-based design extends device lifetimes by up to 5-fold compared to those using common hosts with high triplet energy.^404^ Additionally, TEMs can drive the triplet–triplet fusion (TTF) process, converting triplet excitons into singlet excitons and thus transforming MR emitters into fluorescent emitters to resolve issues linked to prolonged triplet excitons. Consequently, DABNA-1 (1) derivatives have been widely commercialized as blue emitters in triplet–triplet annihilation (TTA)-driven fluorescent devices.^405–407^ Specifically, anthracene-based hosts with MR-dopant t-DABNA-dtB (152) demonstrated this in deep blue TTF-OLEDs, achieving EQE_max_ values of up to 11.4% for the single electroluminescence-unit device and 30.1% for the tandem one. Notably, the tandem OLED also exhibited an LT_95_ of 502 h (initial luminance at 1000 cd m^–2^), representing the longest reported device lifetimes for deep blue OLEDs with a CIE_*y*_ coordinate under 0.12.^115^

From a material design standpoint, TEMs have been also utilized to design the proof-of-concept MR emitters. For instance, CzBNPyr (633), incorporating a low-triplet pyrene unit, achieved narrowband emission and rapid removal of triplets via non-TADF characteristics. A blue HF-OLED based on CzBNPyr (633) demonstrated an EQE_max_ of 20% and a 10-fold improvement in stability compared to standard MR-TADF emitters such as Cz-BN (102) and CzBNNa (634).^408^ Furthermore, bipolar host matrices like PIC-TRZ2 (635), acting as a nonbarrier functional spacer between the emissive layer and the electron transporting layer, enabled the distribution of the recombination zone away from interfaces. Consequently, the optimized OLED based on MR emitter h-BNCO-1 (94)^83^ and host PIC-TRZ2 (635) exhibited a low driving voltage, promising device stability (LT95 > 430 h at 1000 cd m^–2^) and a high CIE_*y*_ coordinate of 0.69.^409^

TEMs have also been applied in phosphor-sensitized HF-OLED. For example, in device employing CN-Ir (636) as a phosphor and ν-DABNA (11) as the terminal emitter, demonstrated simultaneous forward and backward energy transfer between the two components, subsequently minimizing exciton lifetimes and reducing the probability of TTA and TPA processes. In this configuration, the bottom-emitting device achieved a deep blue CIE_*y*_ coordinate of 0.16 and a narrow FWHM of 20 nm (see Figure 45 for the molecular structures). Meanwhile, the corresponding top-emitting device exhibited a record current efficiency of 37.0 cd/A and an impressive LT_50_ of 493 h at 1000 cd m^–2^.^381^

**Figure 45:**
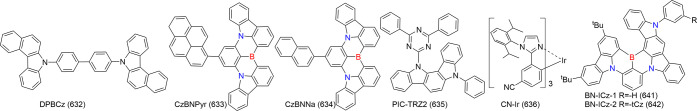
Molecular structures (No. 632–642) in the text.

Overall, OLEDs based on MR emitters have demonstrated remarkable performance. Currently, optimizing the emitting layers often involves the introduction of suitable TADF or phosphor sensitizers or exciplex-type co-hosts into high-gap matrices to prevent Dexter transfer. However, these approaches pose new challenges, including selecting appropriate sensitizers, controlling doping concentrations, and addressing increased fabrication costs. Binary systems, particularly TADF materials with short decay lifetimes, show potential as bifunctional host sensitizers that simplify device fabrication and accelerate exciton decay. For example, “matrix-free” HF-OLEDs utilizing pristine TADF hosts like DMAC-DPS (637) doped with alkylene straps encapsulated MR emitters, NB-1 (638) and NB-2 (639), achieved ultranarrowband electroluminescence (FWHM of 14.5 nm) and an EQE_max_ of 21.5% (see Figure 46).^366^ Another innovative approach employs a combination of an antiquenching TADF host SpiroAC-TRZ (621) and traditional concentration-sensitive MR-TADF emitter GBN (640), enabling a binary emissive layer to achieve pure-green emission with EQE_max_ exceeding 30%, offering a straightforward to achieve antiquenching matrix-free HF-OLEDs.^410^

**Figure 46:**
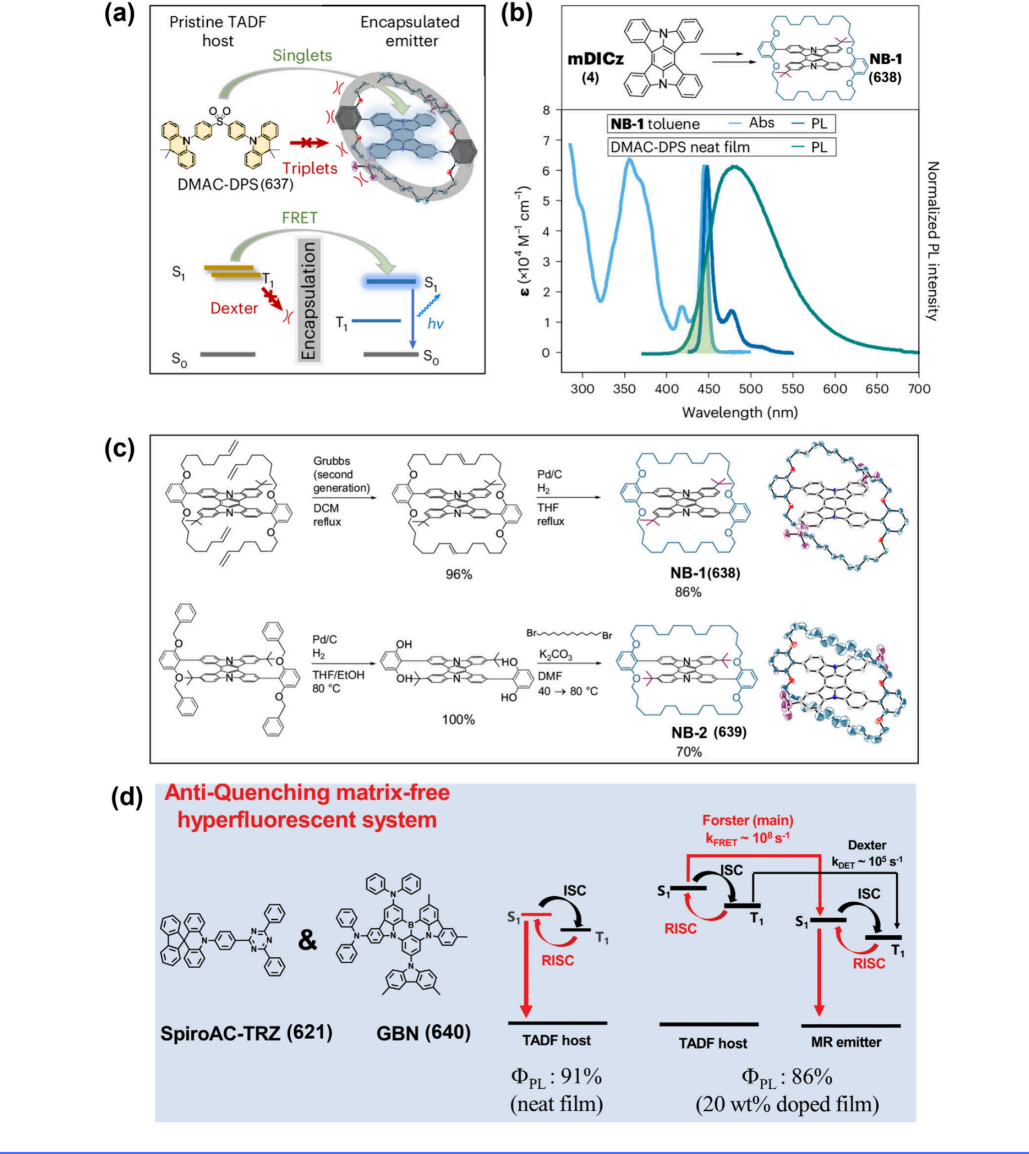
(a) Schematic and Jablonski diagram for the suppression of Dexter triplet transfer from a TADF host via emitter encapsulation. (b) Structures of the mDICz (4) and encapsulated NB-1 (638) luminophores; relevant absorption and PL spectra for NB-1 (563) and DMAC-DPS (637). (c) Synthesis schemes and structures of NB-1 (638) and NB-2 (639) with X-ray single-crystal structures (H atoms are omitted for clarity). (d) Matrix-free HF system based on antiquenching TADF host SpiroAC-TRZ (621) and traditional concentration-sensitive MR-TADF emitter.^366,410^ Reproduced with permission from ref 366 (copyright, 2024, Springer Nature) and ref 410 (copyright, 2024, John Wiley and Sons).

Existing practical device technologies hinder the co-vacuum deposition of complex multicomponent systems, unless supported by significant investments in advanced manufacturing technologies. Furthermore, most reported studies focus on bottom-emission OLEDs fabricated on glass substrates with simple encapsulation in small sizes, which deviate from practical application requirements. While these OLED architectures are well-suited to the glass panel display manufacturing chain, their integration into photonic devices can be challenging—and even unfeasible—for applications involving microtechnologies at the silicon wafer level, such as optical communication or high-definition displays.

From a scientific perspective, researchers never stop pursuing “faster, higher, stronger—together”. However, the ultimate does not denote the best, just like CzBSe (417), which achieved the highest reported *k*_RISC_ value but necessitated additional multiple spin-flipping cycles.^253^ Similarly, it is one-sided to only focus on the FWHM of the emission spectrum. We should also pay attention to the peak position of the emission spectrum. If the emission peak is far away from the three-primary color window, no matter how narrow the FWHM is, it may lack practical application significance. Herein, we hope to foster mutual encouragement among experts and colleagues to confront the current challenges in the scientific field. Specifically, there is a tendency to overestimate external quantum efficiency while overlooking power consumption, which is particularly critical for battery-powered mobile displays.

There is no doubt that key performance indicators such as high efficiency and long operational life are benchmarks for industrial applications. The broad prospects of HF-OLEDs lie in simplified architecture, narrowband emission, and high efficiency, which represent the main direction of scientific research and practical applications.

## 8 Summary and Outlook

MR emitters have emerged as a leading class of materials in OLEDs, renowned for their narrow emission bands (below 40 nm) and high efficiency (exceeding 30% EQE_max_) across panchromatic regions. However, these emitters still encounter challenges such as limited molecular diversity, prolonged τ_DF_, low *k*_RISC_ rates, undesired ACQ, and difficulties in achieving deep-red and NIR emissions. Recent advances, combining experimental and theoretic approaches, have provided valuable insights into the fundamental emission mechanisms of MR emitters.^73,375,411–418^ Comprehensive quantum-chemical calculations, such as Spin-Component Scaling second-order approximate Coupled-Cluster (SCS-CC2), have proven highly effective in accurately predicting energy gaps and excited state properties.^418^ Additionally, methods like delta self-consistent field (ΔSCF) and unrestricted Kohn–Sham (ΔUKS) have been instrumental in simulating singlet and triplet excited states.^416,419^

To some extent, considerable progress in MR emitters has been achieved through enhanced molecular diversity, emission modulation from pure violet to deep red, mitigation of undesired ACQ, and improved spin-flipping RISC. Further development of MR-TADF emitters may focus on addressing efficiency roll-off by resorting to the merits of TADF emitters with a negative singlet–triplet energy gap_._ ^420,421^ To overcome the complexities of three or four components co-evaporation processes in HF-OLEDs fabrication while maintaining high device efficiency and narrow bandwidth, future MR designs could explore metal coordination like gold­(I) and platinum­(II) complexes, a strategy that has proven effective in triplet exciton harvesting.^297,422^ Addressing color purity remains critical, particularly by eliminating unwanted shoulder peaks caused by delocalized π-bonding orbitals on the central phenyl ring of many MR-TADF emitters.^423^ Exemplary models, such as BN-TP (123),^102^ BN-ICz-1 (641), and BN-ICz-2 (642)^424^ (see Figure 45 for the molecular structures), incorporate PAHs segments into the MR-core, achieving ideal lasing-like Gaussian emission profiles. In device engineering, although HF-OLEDs have made remarkable strides, there is still an urgent need to streamline the fabrication processes to improve cost-effectiveness. Binary systems, composed solely of a TADF sensitizer and a “matrix-free” MR emitter, such as pristine TADF hosts combined with encapsulated NB-2 (639), offer promising avenues for future research.^366^ Furthermore, the development of CPMR-TADF emitters and advancements in the TEMs strategies could drive significant progress in OLED technology. HF technology also offers a valuable opportunity to revive traditional fluorescence with unique characteristics such as narrow FWHM and excited-state intramolecular proton transfer (ESIPT).^425,426^

Based on current device performance data, MR emitters employing HF-technology have demonstrated immense potential as strong candidates for future commercial applications. This undoubtedly strengthens OLEDs in their competition with mini-LEDs (mLEDs) and micro-LEDs (μLEDs).^427^ Consequently, we expect that MR-emitter-based OLEDs that meet commercial specifications will soon become a reality, especially with the help of generative artificial intelligence inverse design.^428–430^ In conclusion, this review has timely summarized the progress of MR-TADF emitters and highlighted the challenges that must be addressed to further advance this field. We hope this work will inspire the continued development of MR emitters and provide valuable insights for researchers in related disciplines.

## Supplementary Material



## Notes

Functional molecular structures, photophysical data, and optimized electroluminescent performance of the MR-emitters (PDF)
