# Supplementary material for: Comprehensive Review on the Structural Diversity and Versatility of Multi-Resonance Fluorescence Emitters: Advance, Challenges, and Prospects toward OLEDs
Source: Chem Rev. 2025 May 9;125(14):6685–752. doi: 10.1021/acs.chemrev.5c00021 (PMC12291210; doi:10.1021/acs.chemrev.5c00021)
Supplement: Supplementary file 1 [file cr5c00021_si_001.pdf]

# Supporting Information

## Comprehensive Review on the Structural Diversity and Versatility of Multi-Resonance Fluorescence Emitters: Advance, Challenges and Prospects toward OLEDs

Xiugang Wu*,[1], Songqian Ni[1], Chih-Hsing Wang[2], Weiguo Zhu*,[1], Pi-Tai Chou*,[2]

[1]*School of Materials Science and Engineering, Jiangsu Engineering Laboratory of Light-Electricity-Heat Energy-Converting Materials and Applications, Changzhou University, Changzhou, 213164, China.*

[2]National Taiwan University, Department of Chemistry, Taipei, 10617, Taiwan.

For the readers' convenience, we have also summarized the functional molecular structures, photophysical data, and optimized electroluminescent performance of the MR emitters (see the following Figure 1 and Table 1-6).

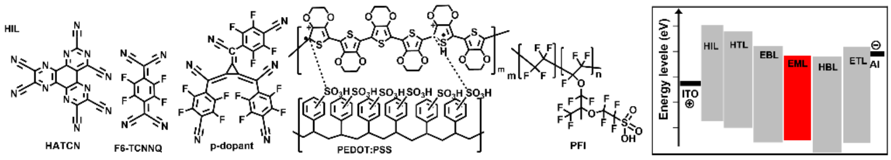
HIL
HATCN
F6-TCNNQ
p-dopant
PEDOT:PSS
PFI
Energy levele (eV)
ITO
HIL
HTL
EBL
EML
HBL
ETL
Al

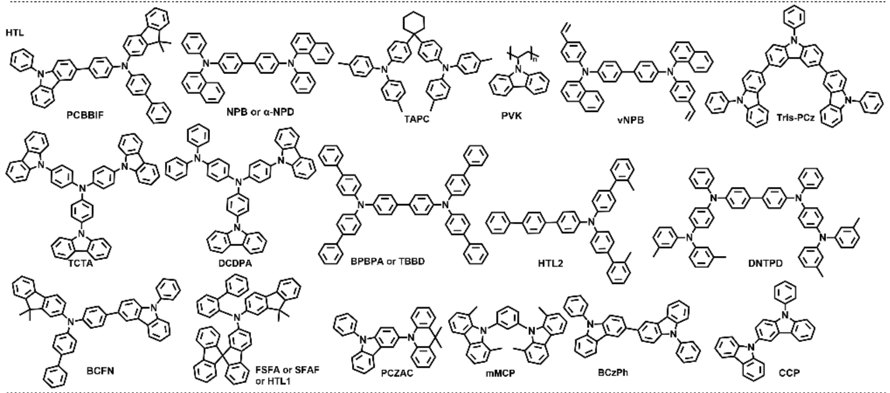
HTL
PCBBIF
NPB or α-NPD
TAPC
PVK
vNPB
Tris-PCz
TCTA
DCDPA
BPBPA or TBBD
HTL2
DNTPD
BCFN
FSFA or SFAF or HTL1
PCZAC
mMCP
BCzPh
CCP

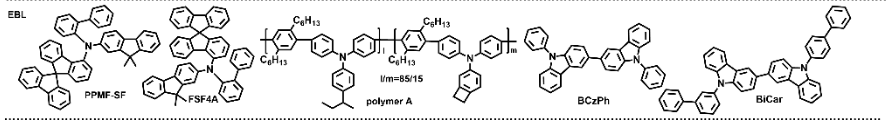
EBL
PPMF-SF
FSF4A
l/m=85/15
polymer A
BCzPh
BiCar

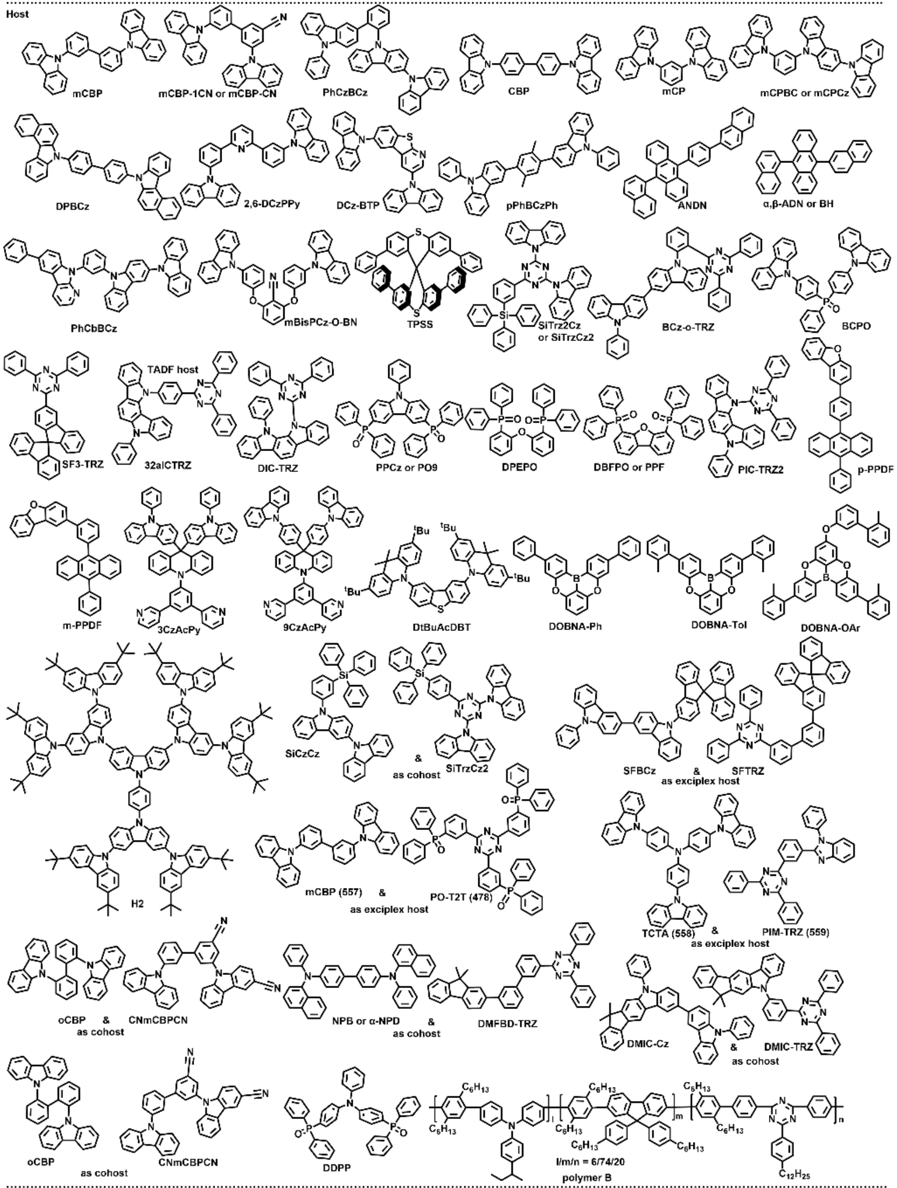
Host
mCBP
mCBP-1CN or mCBP-CN
PhCzBCz
CBP
mCP
mCPBC or mCPCz
DPBCz
2,6-DCzPPy
DCz-BTP
pPhBCzPh
ANDN
α,β-ADN or BH
PhCbBCz
mBisPCz-O-BN
TPSS
SiTrz2Cz or SiTrzCz2
BCz-o-TRZ
BCPO
SF3-TRZ
TADF host
32alCTRZ
DIC-TRZ
PPCz or PO9
DPEPO
DBFPO or PPF
PIC-TRZ2
p-PPDF
m-PPDF
3CzAcPy
9CzAcPy
DtBuAcDBT
DOBNA-Ph
DOBNA-Tol
DOBNA-OAr
SiCzCz
& as cohost
SiTrzCz2
SFBCz
& as exciplex host
SFTRZ
H2
mCBP (557)
& as exciplex host
PO-T2T (478)
TCTA (558)
& as exciplex host
PIM-TRZ (559)
oCBP
& as cohost
CNmCBPCN
NPB or α-NPD
& as cohost
DMFBD-TRZ
DMIC-Cz
& as cohost
DMIC-TRZ
oCBP
as cohost
CNmCBPCN
DDPP
l/m/n = 6/74/20
polymer B

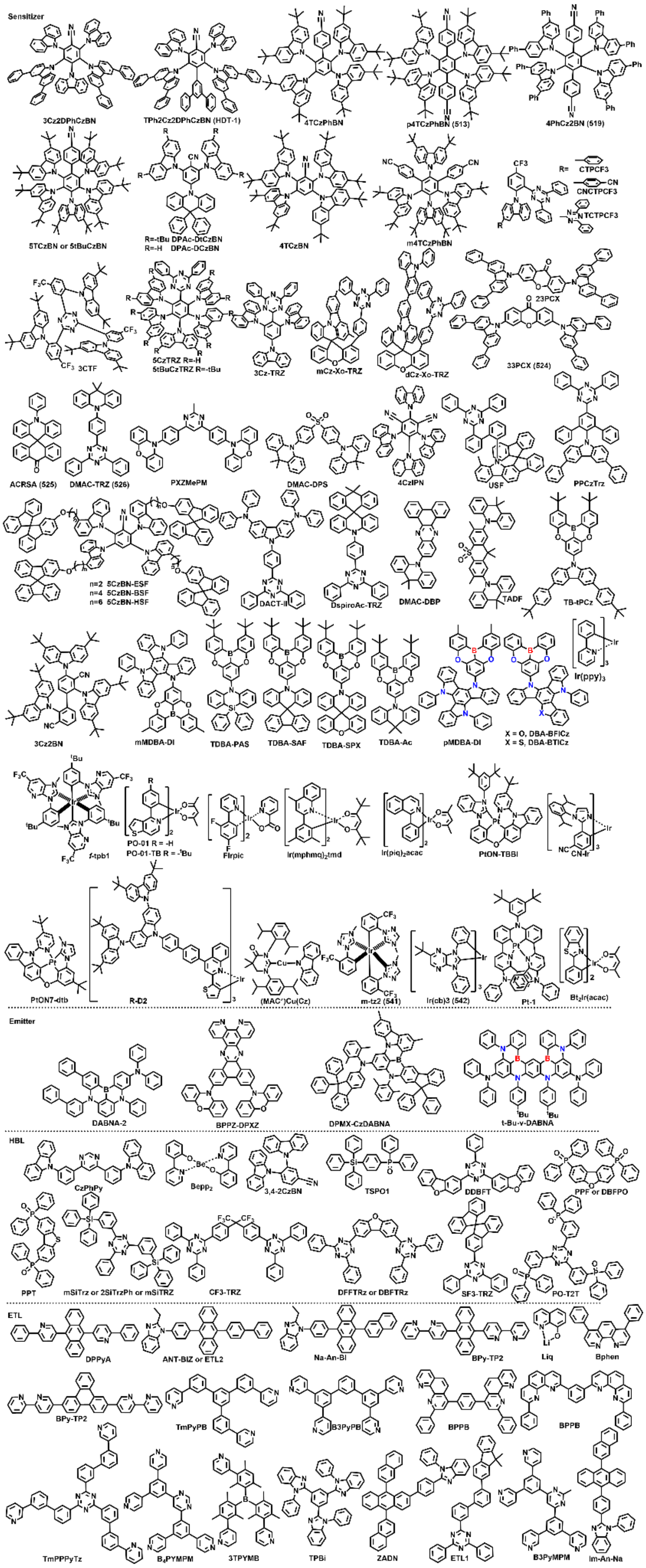


**Figure S1**. The common molecular structures of materials utilized in device fabrication.

## Table S1. Summary of reported B/N-type MR-TADF emitters.

| Emitter | $\lambda_{em}$ (nm) | FWHM (nm) | $\Delta E_{ST}$ (eV) | $\Phi_{PL}$ (%) | $\tau_p$ (ns) | $\tau_d$ (μs) | $k_r$ ($10^7$ $s^{-1}$) | $k_{IC}$ ($10^6$ $s^{-1}$) | $k_{ISC}$ ($10^7$ $s^{-1}$) | $k_{RISC}$ ($10^4$ $s^{-1}$) | test status | Ref. |
|---|---|---|---|---|---|---|---|---|---|---|---|---|
| DABNA-1 | 462 | 30 | 0.2 | 88 | 8.8 | 93.7 | 9.6 | 13 | 0.45 | 0.99 | 1 wt% in mCBP | 1 |
| DABNA-2 | 470 | 28 | 0.2 | 90 | 6 | 65.3 | 14.1 | 16 | 1.01 | 1.48 | | |
| 2a | 399 | 26 | 0.21 | 54 | - | - | - | - | - | - | 1 wt% in PMMA | 2 |
| B2 | 455 | 32/0.19 | 0.17 | 53 | 2.81 | 30.4 | 8 | 7.07 | 20.5 | 0.929 | 1 wt% in PMMA | 3 |
| B3 | 441 | 34/0.21 | 0.15 | 33 | - | - | - | - | - | - | | |
| B4 | 450 | 38/0.22 | 0.15 | 57 | - | - | - | - | - | - | | |
| TBN-TPA | 470 | 26 | 0.14 | 97 | 6.02 | 51.02 | - | - | - | - | 4 wt% in 2,6-DCzppy | 4 |
| ADBNA-Me-Mes | 482 | 33 | - | 89 | 6.9 | 165 | 10 | 13 | 2.9 | 0.76 | 1 wt% in DOBN-OAr | 5 |
| ADBNA-Me-Tip | 479 | 34 | - | 88 | 6 | 147 | 11 | 16 | 4.1 | 0.9 | | |
| t-DABNA | - | 31 | 0.17 | 85 | 6 | 83.3 | - | - | 3.03 | 0.244 | 1 wt% in DPEPO | 6 |
| 2F-BN | 499 | 32 | 0.16 | 89 | - | 25.9 | 5.65 | - | 1.49 | 2.2 | 6 wt% in mCPCB | 7 |
| 3F-BN | 499 | 33 | 0.08 | 83 | - | 16.7 | 4.66 | - | 2.01 | 3.9 | | |
| 4F-BN | 493 | 31 | 0.11 | 91 | - | 19 | 5.17 | - | 1.63 | 4.4 | | |
| ν-DABNA | 468 | 14 | - | 74 | 3 | - | 24 | - | - | - | 1×10$^{-5}$M in toluene | 8 |
| | 467 | 18 | 0.017 | 90 | 4.07 | 4.05 | 20 | 2.17 | 2.32 | 20 | 1 wt% in DOBNA-OAr | |
| OAB-ABP-1 | 506 | 34 | 0.12 | 90 | 9.9 | 32 | 7.2 | 7.9 | 21 | 4 | 1 wt% in PMMA | 9 |
| AZA-BN | 522 | 28 | 0.18 | 94 | 13.4 | 160 | 5.04 | - | 2.42 | 7.53 | 4 wt% in mCBP | 10 |
| m-Cz-BNCz | 525 | 38 | 0.08 | 97 | 8.8 | 10.6 | 9.7 | 0.4 | 1.25 | 108 | 10 wt% in PhCzBCz | 11 |
| DtBuCzB | 488 | 22 | 0.13 | 88 | 8.5 | 68.8 | - | - | - | - | 1 wt% in mCBP | 12 |
| DtBuPhCzB | 504 | 21 | 0.09 | 90 | 22.8 | 61.3 | - | - | - | - | | |
| BBCz-DB | 466 | 16 | 0.15 | 93 | 4.5 | 86 | 11 | - | 11 | 1.9 | 1×10$^{-5}$M in toluene | 13 |
| BBCz-SB | 489 | 23 | 0.15 | 98 | 4.7 | 102 | 14 | - | 7.4 | 1.4 | | |
| BBCz-G | 517 | 34 | 0.14 | 90 | 5 | 17 | 5.8 | - | 14 | 18 | | |
| BBCz-Y | 549 | 42 | 0.14 | 85 | 5.6 | 29 | 4.7 | - | 13 | 10 | | |
| BBCz-R | 615 | 21 | 0.19 | 89 | 6.1 | 89 | 11 | - | 5.7 | 1.2 | | |
| 3 | 482 | - | 0.18 | 84 | - | - | - | - | - | - | 1 wt% in PMMA | 14 |
| 4a | | - | 0.19 | 91 | - | - | - | - | - | - | | |
| 4b | 487 | - | 0.19 | 93 | - | - | - | - | - | - | | |
| 5a | | - | 0.18 | 91 | - | - | - | - | - | - | | |
| 5b | 486 | - | 0.17 | 86 | - | - | - | - | - | - | | |
| 6a | | - | 0.17 | 26 | - | - | - | - | - | - | | |
| 6b | 481, 601 | - | 0.13 | 24 | - | - | - | - | - | - | | |

# Table S1. Summary of reported B/N-type MR-TADF emitters.

| | | | | | | | | | | | | |
|---|---|---|---|---|---|---|---|---|---|---|---|---|
| α-3BNOH | 390 | 31 | 0.31 | 50 | 8.5 | 0.45 | - | - | - | - | $1×10^{-5}$M in THF | 15 |
| BN-DMAC | 512 | 29 | 0.14 | 63 | 5.1 | 13.9 | 9.2 | - | 4.86 | 9.6 | 1 wt% in mCBP | 16 |
| BN-DPAC | 516 | 30 | 0.11 | 86 | 4.4 | 11.6 | 13 | - | 7.21 | 12.6 | | |
| R-BN | 662 | 38 | 0.18 | 100 | 12 | 16.6 | 7.5 | - | 0.83 | 6.7 | $1×10^{-5}$M in toluene | 17 |
| R-TBN | 692 | 38 | 0.16 | 100 | 14.2 | 46.4 | 6.2 | - | 0.85 | 2.5 | | |
| CzDABNA-NP-M/TB | 468 | 28 | 0.18 | 86 | 8.7 | 106 | 8.4 | 14 | 1.7 | 1.1 | 1 wt% in PMMA | 18 |
| Cz2DABNA-NP-M/TB | 478 | 29 | 0.14 | 85 | 7.6 | 19 | 8.1 | 14 | 3.7 | 7.3 | | |
| CzB2-M/TB | 491 | 34 | 0.11 | 88 | 21 | 50 | 3.6 | 5 | 0.75 | 2.4 | | |
| Cz2B2-M/TB | 483 | 38 | 0.11 | 88 | 16 | 42 | 4.2 | 5.8 | 1.4 | 3.1 | | |
| CzDABNA-NP | 461 | 30 | 0.18 | 80 | 8.1 | 77 | 8 | 19 | 2.4 | 1.6 | | |
| CzDABNA-NP-TB/H | 465 | 30 | 0.18 | 82 | 8.3 | 93 | 8.1 | 18 | 2.1 | 1.3 | | |
| DABNA-NP-M | 460 | 29 | 0.17 | 88 | 9.7 | 89 | 7.5 | 9.7 | 1.9 | 1.4 | | |
| DABNA-NP-TB | 453 | 26 | 0.17 | 83 | 8.4 | 90 | 8.1 | 17 | 2.2 | 1.4 | | |
| CzB2-M/P | 504 | 39 | 0.06 | 87 | 28 | 24 | 2.6 | 3.8 | 0.64 | 5.1 | | |
| 2PXZBN | 504 | 34 | 0.19 | 78 | 4.9 | 11.6 | 20.4 | - | 10.3 | 10.3 | $1×10^{-5}$M in toluene | 19 |
| 2PTZBN | 510 | 39 | 0.15 | 58 | 4.2 | 5 | 23.8 | - | 27.8 | 27.6 | | |
| 2PXZBN | 515 | 40 | 0.18 | 84 | 6.8 | 25.3 | 14.7 | - | 5.6 | 5.6 | 1wt% in mCBP: PO-T2T | |
| 2PTZBN | 519 | 44 | 0.14 | 80 | 4.8 | 16.1 | 20.8 | - | 11.7 | 11.7 | | |
| 2PXZBN | 523 | - | 0.15 | 71 | 5.2 | 38.1 | 8.2 | | 7.5 | 4.3 | 1 wt% in DMIC-TRZ | 20 |
| 2PTZBN | 525 | - | 0.13 | 91 | 5.1 | 20.7 | 4.5 | | 15 | 19 | | |
| BNSSe | 520 | - | 0.12 | 99 | 3.0 | 12.7 | 4.3 | | 29 | 60 | | |
| BNSeSe | 514 | - | 0.14 | 100 | 1.9 | 9.9 | 2.6 | | 49 | 200 | | |
| OBN-2CN-BN | 493 | 22 | 0.12 | 99 | 9.8 | 95.3 | 8.92 | 4.7 | 0.82 | 0.95 | 3 wt% in PhCzBCz | 21 |
| OBN-4CN-BN | 500 | 27 | 0.13 | 96 | 10 | 97.4 | 7.92 | 8.8 | 1.2 | 0.83 | | |
| BOO | 396 | 30 | 0.18 | 70 | 9.2 | 101.1 | - | - | - | 1.1 | 1 wt% in PS | 22 |
| BOS | 434 | 29 | 0.17 | 63 | 10 | 90.6 | - | - | - | 6.1 | | |
| BSS | 457 | 27 | 0.15 | 58 | 11 | 85.1 | - | - | - | 11.8 | | |
| CzBN | 479 | 30 | 0.12 | 87.1 | 7 | 44.8 | 11.7 | - | 0.845 | 1.69 | 1 wt% in mCP | 23 |
| BCzBN | 497 | 28 | 0.11 | 86.3 | 8.4 | 50.4 | 9.8 | - | 0.498 | 1.48 | | |
| ν-DABNA-O-Me | 464 | 24 | 0.12 | 90 | 5.1 | 7.7 | 14 | 1.6 | 4 | 16 | 1 wt% in PMMA | 24 |
| BN1 | 496 | 23 | - | 99 | - | - | - | - | - | - | $1×10^{-5}$M in toluene | 25 |
| BN2 | 534 | 30 | - | 98 | - | - | - | - | - | - | | |
| BN3 | 562 | 30 | - | 98 | - | - | - | - | - | - | | |

## Table S1. Summary of reported B/N-type MR-TADF emitters.

| | | | | | | | | | | | | |
|---|---|---|---|---|---|---|---|---|---|---|---|---|
| BN1 | 499 | 38 | 0.11 | 93 | 6.2 | 68.6 | 11.8 | - | 3.5 | 19 | 1 wt% in mCBP | |
| BN2 | 538 | 41 | 0.13 | 89 | 7.1 | 107.6 | 7.9 | - | 5.2 | 15 | | |
| BN3 | 563 | 44 | 0.09 | 86 | 6.3 | 127.9 | 7.3 | - | 7.4 | 14 | | |
| DMAC-BN | 484 | 33 | 0.16 | 88 | 6.2 | 32.9 | 13.1 | 17.8 | 1.3 | 2.4 | 3 wt% in mCBP | 26 |
| PXZ-BN | 502 | 38 | 0.17 | 90 | 8.2 | 90.7 | 8.2 | 9.1 | 3.1 | 0.9 | | |
| CN-BCz-BN | 496 | 21 | | - | - | - | - | - | - | - | $1\times10^{-5}$M in toluene | 27 |
| CNCz-BNCz | 581 | 42 | 0.18 | 90 | 28.4 | 3.4 | 2.2 | | 1.1 | 42 | | |
| | 582 | 45 | | 96 | 17.7 | 60.4 | | | | | 3 wt% in mCBP | |
| PAB | 453 | 23 | 0.06 | 60.8 | 7.9 | 55.7 | 1.99 | 12.8 | 9.41 | 6.95 | 3 wt% in mCP | 28 |
| 2tPAB | 457 | 26 | 0.08 | 67.3 | 8.1 | 76.6 | 1.74 | 8.45 | 9.76 | 6.34 | | |
| 3tPAB | 458 | 23 | 0.1 | 74.7 | 7.2 | 74.2 | 2.43 | 8.23 | 10.63 | 5.74 | | |
| BSBS-N1 | 478 | 24 | 0.14 | 89 | 0.9 | 5.6 | 9 | - | 100 | 190 | 2 wt% in mCBP | 29 |
| y-Cb-B | 460 | 23 | 0.12 | 83 | 5 | 39 | 10 | - | 9.5 | 3.1 | $1\times10^{-5}$M in toluene | 30 |
| Cz-B | 477 | 25 | 0.14 | 98 | 5.2 | 106 | 13 | - | 4.5 | 1.2 | | |
| TCz-B | 512 | 27 | 0.09 | 100 | 5.4 | 60 | 16 | - | 3 | 2 | | |
| DACz-B | 572 | 34 | 0.14 | 87 | 7.9 | 316 | 9.8 | - | 3 | 1.8 | | |
| y-Cb-B | 461 | 30 | - | 89 | 6 | 32 | 7.3 | - | 9.4 | 5.8 | 1 wt% in oCBP | |
| Cz-B | 484 | 30 | - | 97 | 4.9 | 44 | 15 | - | 5.5 | 2.7 | 1 wt% in mCBP | |
| TCz-B | 517 | 31 | - | 89 | 5.4 | 71 | 13 | - | 5.7 | 1.3 | | |
| DACz-B | 576 | 44 | - | 87 | 7.8 | 118 | 7.5 | - | 5.3 | 1 | | |
| DtCzB-DPTRZ | 521 | 24 | 0.18 | 87 | 13.1 | 787.5 | 5.65 | 8.4 | 1.14 | 0.1 | 3 wt% in PhCzBCz | 31 |
| DtCzB-TPTRZ | 501 | 27 | 0.11 | 95 | 8.7 | 83.5 | 7.32 | 3.9 | 3.79 | 1.08 | | |
| DtCzB-PPm | 499 | 25 | 0.11 | 94 | 8.3 | 86.5 | 7.7 | 4.9 | 3.86 | 1.02 | | |
| DtCzB-CNPm | 515 | 36 | 0.15 | 87 | 14.2 | 524.3 | 5.08 | 7.6 | 1.2 | 0.14 | | |
| B-O-dpa | 433 | 28/- | 0.18 | 86 | 10 | 224 | 10.4 | - | 6.15 | 0.83 | 10 wt% in DPEPO | 32 |
| B-O-Cz | 441 | 27/- | 0.15 | 94 | 16 | 51 | 6.4 | - | 3.69 | 4.02 | | |
| B-O-dmAc | 461 | 38/- | 0.11 | 91 | 9 | 123 | 11.4 | - | 7.16 | 1.82 | | |
| B-O-dpAc | 463 | 38/- | 0.06 | 94 | 8 | 83 | 12.3 | - | 8.02 | 3.12 | | |
| DtBuCzB | 480 | 25/- | 0.04 | 92 | 6.7 | 56.7 | 12 | - | 0.21 | 3 | $1\times10^{-5}$M in toluene | 33 |
| S-Cz-BN | 490 | 23/- | 0.16 | 94 | 5.6 | 69.6 | 13 | - | 0.39 | 1.8 | | |
| D-Cz-BN | 490 | 22/- | 0.14 | 98 | 5.8 | 76 | 13 | - | 0.45 | 1.8 | | |
| BN-CP1 | 490 | 23 | 0.12 | 93 | 5.1 | 79.6 | 14.6 | - | 3.86 | 1.56 | 1 wt% in mCBP | 34 |
| BN-CP2 | 490 | 23 | 0.13 | 91 | 5 | 83.6 | 15.3 | - | 3.24 | 1.43 | | |
| BN1 | 502 | 41/0.196 | 0.2 | 61 | 6.19 | 0 | - | - | - | - | $1\times10^{-5}$M in toluene | 35 |
| BN2 | 475 | 34/0.184 | 0.21 | 88 | 6.39 | 0 | - | - | - | - | | |
| BN3 | 511 | 46/0.216 | 0.16 | 72 | 4.83 | 16.2 | 9.2 | 36 | 8 | 10 | | |
| BN4 | 500 | 43/0.207 | 0.14 | 88 | 5.1 | 8.2 | 13 | 15 | 5.2 | 16 | | |
| BN5 | 497 | 44/0.216 | 0.13 | 87 | 5.1 | 23.1 | 10 | 15 | 8 | 7.4 | | |
| BN4 | 522 | 50/0.231 | - | 96 | 4.71 | 24.72 | 4.9 | 2.1 | 16 | 3.7 | 3 wt% in mCPCN | |
| BN5 | 512 | 49/0.228 | - | 92 | 5.36 | 25.27 | 5.2 | 4.6 | 13 | 3.3 | 1 wt% mCPCN | |
| 1a | 660 | - | 0.22 | 100 | 13 | 16.4 | 6.5 | - | 1.23 | 7.3 | $1\times10^{-5}$M in toluene | 36 |

**Table S1. Summary of reported B/N-type MR-TADF emitters.**

| | | | | | | | | | | | | |
|---|---|---|---|---|---|---|---|---|---|---|---|---|
| 1b | 684 | - | 0.21 | 99 | 12.2 | 43.9 | 7.4 | - | 0.82 | 2.3 | | |
| 1c | 696 | - | 0.18 | 90 | 11 | 26.8 | 6.8 | - | 2.27 | 3 | | |
| CzBNCz | 470 | 22 | 0.18 | 95 | 6.8 | 92 | 1.28 | - | 1.9 | 0.76 | 1 wt% in mCBP | 37 |
| CzBN | 485 | 25 | 0.15 | 99 | 6 | 75 | 1.55 | - | 1.2 | 1.24 | | |
| CzBNO | 450 | 30 | 2.97 | | 7.1 | 48 | 8.08 | - | 5.61 | 3.47 | 3 wt% in 26DCzPPy | 38 |
| DMAcBNO | 470 | 41 | 2.89 | | 7.3 | 129.4 | 7.44 | - | 6.15 | 1.4 | | |
| DPAcBNO | 468 | 39 | 2.86 | | 7.5 | 100.1 | 7.46 | - | 5.74 | 1.75 | | |
| TW-BN | 485 | 25 | 0.12 | 92 | 2.9 | 112 | 2.44 | 2.12 | 7.95 | 8.21 | 3 wt% in mCBP | 39 |
| TPh-BN | 495 | 28 | 0.09 | 94 | 4.9 | 62 | 1.34 | 0.86 | 6.12 | 15.2 | | |
| pCz-BN | 496 | 30 | 0.15 | 95 | 4.1 | 89 | 1.48 | 0.78 | 8.81 | 8.5 | | |
| mCz-BN | 494 | 29 | 0.14 | 88 | 6.7 | 95 | 1.06 | 1.45 | 2.83 | 13.1 | | |
| DPACzBN1 | 479 | 31 | 0.11 | 98 | 4.9 | 116 | 1.5 | - | 0.5 | 1.2 | 3 wt % 26DCzPPy | 40 |
| DPACzBN2 | 470 | 29 | 0.12 | 92 | 4.1 | 54 | 1.4 | - | 0.9 | 2.9 | | |
| DPACzBN3 | 475 | 27 | 0.13 | 94 | 2.6 | 69 | 2.5 | - | 1.2 | 2.1 | | |
| BP-2DPA | 601 | 37 | | 91.3 | 12.5 | | 7.79 | | | | 1 wt% in DIC-TRz | 41 |
| DBP-4DPA | 612 | 35 | - | 96.4 | 18.9 | - | 5.03 | - | - | - | | |
| v-DABNA-Mes | 484 | 16 | 0.008 | 80 | 6.98 | 2.39 | 11 | 27 | 0.73 | 44 | 1 wt% in PMMA | 42 |
| BOBO-Z | 441 | 15 | 0.15 | 76 | 3.2 | 16 | 15 | - | 16 | 7 | $1\times10^{-5}$M in toluene | 43 |
| BOBS-Z | 453 | 21 | 0.159 | 94 | 1.3 | 18 | 9 | - | 68 | 43 | | |
| BSBS-Z | 460 | 20 | 0.144 | 93 | 1 | 15 | 7 | - | 93 | 88 | | |
| BOBO-Z | 445 | 18 | 0.102 | 64 | 2.3 | 7.7 | 22 | - | 22 | 7 | 3 wt% in mCBP | |
| BOBS-Z | 457 | 24 | 0.121 | 93 | 1.1 | 7.6 | 13 | - | 78 | 86 | | |
| BSBS-Z | 464 | 22 | 0.122 | 88 | 1 | 6.7 | 8 | - | 92 | 160 | | |
| m-v-DABNA | 464 | 14 | 0.07 | 90.5 | 8.43 | 3.09 | 9.31 | - | 2.55 | 23 | 3 wt% in DBFPO | 44 |
| 4F-v-DABNA | 457 | 14 | 0.05 | 90.2 | 8.72 | 3.12 | 8.94 | - | 2.52 | 22.8 | | |
| 4F-m-v-DABNA | 455 | 14 | 0.07 | 88.9 | 8.2 | 3.19 | 9.4 | - | 2.79 | 21 | | |
| TPXZBN | 502 | 33 | 0.16 | 91 | 7.4 | 27.1 | 13.51 | - | 2.53 | 4.8 | 5 wt% mCBP | 45 |
| DPXZCZBN | 500 | 32 | 0.13 | 90 | 10 | 15 | 10 | - | 2.06 | 11.1 | | |
| Cz-BSN | 476 | 24 | 0.13 | 82 | 4.8 | 40.8 | - | - | - | 9.6 | $1\times10^{-5}$M in toluene | 46 |
| DCz-BSN | 463 | 26 | 0.12 | 88 | 4.8 | 49.1 | - | - | - | 10.4 | | |
| BN1 | 492 | 82 | 0.2 | 92 | 4.7 | 6.9 | 0.95 | - | 1.24 | 2.9 | 2 wt% in mCBP | 47 |
| TCz-BN1 | 491 | 89 | 0.16 | 89 | 6.7 | 5.4 | 0.81 | - | 1.32 | 46.7 | | |
| BN2 | 559 | 97 | 0.19 | 64 | 1.2 | 33.2 | 0.13 | - | 1.04 | 21 | 5 wt% in mCBP | |
| TCz-BN2 | 560 | 108 | 0.17 | 61 | 1.2 | 18.1 | 0.15 | - | 0.87 | 24.4 | | |
| BN3 | 694 | 151 | - | 1 | 1.1 | - | - | - | - | - | 5 wt% in PMMA | |
| Cz-PTZ-BN | 510 | 37 | 0.11 | 84 | - | - | - | - | - | - | $1\times10^{-5}$M in toluene | 48 |
| 2Cz-PTZ-BN | 505 | 38 | 0.09 | 87 | - | - | - | - | - | - | | |
| Cz-PTZ-BN | 524 | 50 | - | 91 | 6.3 | 20.7 | 9.1 | - | 5.93 | 8.1 | 3 wt% in PhCzBCz | |
| 2Cz-PTZ-BN | 512 | 48 | - | 96 | 5 | 17.6 | 10.4 | - | 9.17 | 10.5 | | |
| BNCzPXZ | 508 | 36 | 0.13 | 94 | 10.76 | 102.41 | 3.55 | | 5.74 | 2.31 | 7 wt% in PhCzBCz | 49 |

## Table S1. Summary of reported B/N-type MR-TADF emitters.

| | | | | | | | | | | | | |
|---|---|---|---|---|---|---|---|---|---|---|---|---|
| BNCzPTZ | 509 | 42 | 0.09 | 91 | 39.43 | 33.35 | 0.439 | | 2.11 | 15.4 | 15 wt% PhCzBCz | |
| mBP-DABNA-Me | 467 | 28 | 0.12 | 97 | 7.64 | 64.5 | 10.1 | 3.12 | 2.68 | 1.95 | emitter:mCP:DPEPO=5:47.5:47.5 | 50 |
| NBO | 487 | 27 | 0.12 | 92 | 6.7 | 5.9 | 2.5 | - | 12 | 93 | 4 wt% mCBP | 51 |
| NBNP | 500 | 29 | 0.09 | 93 | 7.6 | 3.8 | 8.5 | - | 5 | 30 | | |
| tCBNDADPO | 466 | 26 | 0.04 | 92 | - | - | - | - | - | - | $10^{-6}$M in DCM - | 52 |
| | 472 | 38 | - | 79 | - | - | 12.9 | - | - | 1.75 | 10 wt% in DBFDPO | |
| | 472 | 41 | - | 93 | 1.2 | 2.4 | 20.6 | - | - | 2.27 | 20 wt% in DBFDPO | |
| | 472 | 44 | - | 99 | 0.6 | 0.04 | 26.4 | - | - | 2.43 | 30 wt% in DBFDPO | |
| | 473 | 45 | - | 82 | 0.9 | 0.5 | 31.7 | - | - | 1.85 | 40 wt% in DBFDPO | |
| | 474 | 47 | - | 45 | 0.2 | 1.1 | 20 | - | - | 0.97 | 100% tCBNDADPO | |
| BON-D0 | 450 | 23 | 0.18 | - | - | - | - | - | - | - | $1\times10^{-5}$M in toluene | 53 |
| BON-D1 | 476 | 28 | 0.14 | - | - | - | - | - | - | - | | |
| BON-D2 | 472 | 31 | 0.1 | - | - | - | - | - | - | - | | |
| BON-D0 | 464 | - | - | 85 | 14.5 | 157 | - | - | 6.73 | - | 5 wt% in mCP | |
| BON-D1 | 485 | - | - | 94 | 14.6 | 139.1 | - | - | 7.72 | - | | |
| BON-D2 | 478 | - | - | 98 | 13.8 | 107 | - | - | 9.81 | - | | |
| SF1BN | 493 | 23/0.12 | 0.13 | 90 | - | - | - | - | - | - | $1\times10^{-5}$ M in toluene | 54 |
| SF3BN | 493 | 25/0.13 | 0.15 | 86 | - | - | - | - | - | - | | |
| SF1BN | | - | - | 93 | 16.3 | 9.45 | 4.62 | - | 3.95 | 9.12 | 2 wt% in mCBP | |
| SF3BN | | - | - | 90 | 16.8 | 26 | 3.36 | - | 11.46 | 3.31 | | |
| DBNO | 500 | 19/0.09 | 0.17 | 96 | - | - | - | - | - | - | $1\times10^{-5}$M in toluene | 55 |
| | 508 | 28/- | - | 84 | 6 | 23.2 | 7.9 | 15 | 7.2 | 3 | 1 wt % DBNO: PhCzBCz | |
| | 506 | 29/- | - | 90 | 6.1 | 5.5 | 3.7 | 4 | 12.2 | 14.7 | 1 wt % DBNO:15 wt% 5TCzBN: PhCzBCz | |
| t-DABNA-dtB | 465 | 22 | 0.19 | - | - | - | - | - | - | - | $1\times10^{-5}$M in toluene | 56 |
| | 473 | 30 | - | 97 | 7.1 | 109.6 | 14.1 | - | 8.21 | 2.08 | 3 wt% mCBP | |
| (M)-helicene-BN | 520 | 46 | 0.18 | - | - | - | - | - | - | - | $1\times10^{-5}$M in toluene | 57 |
| | 525 | 48 | 0.15 | 98 | 5 | 71.8 | 5.9 | - | 14 | 4.6 | 1 wt% DMIC-TRZ | |
| BN-ICz-1 | 521 | 21/0.09 | 0.22 | 99.2 | 6.4 | 342 | 16 | - | 14 | 2.9 | $1\times10^{-5}$M in toluene | 58 |
| BN-ICz-2 | 521 | 22/0.09 | 0.18 | 98.3 | 6.7 | 48.6 | 15 | - | 10 | 6.4 | | |
| BN-ICz-1 | | -/0.1 | - | 95 | 6.8 | 239 | - | - | - | - | 3 wt% mCBP | |
| BN-ICz-2 | | -/0.1 | - | 93 | 7.1 | 160 | - | - | - | - | | |
| BN-MeIAc | 497 | 30 | 0.11 | 96 | 8.2 | 28.1 | 6.7 | - | 5.3 | 6.3 | 1 wt% DMIC-TRZ | 59 |
| asym-BN1 | 454 | 18/0.106 | 0.2 | 91 | 4.3 | 126.6 | 13 | - | 8.8 | 1.3 | 1 wt% DBFPO | 60 |
| sym-BN2 | 464 | 15/0.088 | 0.16 | 93 | 3.4 | 74.6 | 14 | - | 14 | 2.6 | | |
| sym-BN3 | 456 | 17/0.101 | 0.15 | 98 | 1.3 | 17.8 | 17 | - | 61.9 | 25.5 | | |
| BN-TP | 523 | 34 | 0.14 | 96 | 7.1 | 44 | 9.87 | 4.1 | 3.8 | 2.09 | 3 wt% PhCzBCz | 61 |
| PTZBN1 | 497 | 44 | 0.16 | 98 | 4.8 | 33.5 | 5.49 | - | 15.2 | 11.1 | 2 wt% 2,6-DCzppy | 62 |
| PTZBN2 | 487 | 42 | 0.15 | 95 | 4.5 | 22.4 | 2.09 | - | 20 | 45.1 | | |

## Table S1. Summary of reported B/N-type MR-TADF emitters.

| | | | | | | | | | | | | |
|---|---|---|---|---|---|---|---|---|---|---|---|---|
| PTZBN3 | 473 | 36 | 0.17 | 98 | 5.8 | 26.5 | 5.93 | - | 11.2 | 10.8 | | |
| BNO1 | 605 | 32 | - | 96 | - | - | - | - | - | - | 1×10$^{-5}$ M in toluene | 63 |
| BNO2 | 609 | 32 | - | 95 | - | - | - | - | - | - | | |
| BNO3 | 616 | 33 | - | 96 | - | - | - | - | - | - | | |
| BNO1 | 610 | 35 | 0.25 | - | 13.6 | - | 7.4 | - | - | - | 1 wt% DMIC-TRZ | |
| BNO2 | 618 | 37 | 0.27 | - | 16.4 | - | 6.1 | - | - | - | | |
| BNO3 | 624 | 38 | 0.26 | - | 15.9 | - | 6.3 | - | - | - | | |
| CzBO | 445 | 26/0.16 | 0.15 | 98 | 6.7 | - | 15 | - | - | - | 1×10$^{-5}$ M in toluene | 64 |
| CzBS | 471 | 28/0.16 | 0.11 | 99 | 4.5 | 36 | 1.5 | - | 21 | 40 | | |
| CzBSe | 477 | 33/0.18 | 0.12 | 98 | 0.7 | 17 | 0.05 | - | 128 | 15000 | | |
| CzBO | 448 | 29/0.18 | 0.16 | 99 | 6.7 | 120 | 13 | - | 2.5 | 0.9 | 1 wt% in mCBP | |
| CzBS | 472 | 30/0.17 | 0.14 | 98 | 4.6 | 30 | 33 | - | 19 | 22 | | |
| CzBSe | 479 | 34/0.18 | 0.15 | 98 | 0.8 | 14 | 0.05 | - | 110 | 18000 | | |
| tDPAC-BN | -- | 26 | 0.17 | 94.4 | 8.1 | 113.8 | 8.8 | 5.22 | 3.02 | 1.16 | 1 wt% in PMMA | 65 |
| tDMAC-BN | - | 34 | 0.15 | 89.7 | 6.5 | 64.2 | 4.51 | 5.18 | 10.4 | 4.8 | | |
| tDPAC-BN | 460 | 26 | - | 91.8 | - | - | - | - | - | - | 1 wt% emitter: 30 wt% DMAC-DPS: DPEPO film | |
| tDMAC-BN | 475 | 34 | - | 94.7 | - | - | - | - | - | - | | |
| SBON | 463 | 24 | 0.16 | 74 | 9.2 | 11.5 | 6.4 | - | 4.3 | 5 | 4 wt% mCBP | 66 |
| SBSN | 489 | 27 | 0.1 | 76 | 8.9 | 32.2 | 1.7 | - | 9.5 | 15 | | |
| DBON | 505 | 20 | 0.13 | 98 | 3.7 | 24.6 | 13.2 | - | 13.8 | 8 | | |
| DBSN | 553 | 28 | 0.13 | 98 | 4.3 | 25.7 | 4.6 | - | 18.7 | 19 | | |
| m[B-N]N1 | 483 | 28/0.15 | 0.15 | 91 | 5.0 | 128.1 | 10.2 | | 9.80 | 1.59 | 4 wt% in mCPBC | 67 |
| m[B-N]N2 | 491 | 33/0.17 | 0.13 | 90 | 5.6 | 136.2 | 8.8 | | 9.16 | 1.44 | | |
| BIC-mCz | 432 | 31 | 0.29 | 82 | 5.6 | 250 | 14.2 | 3.2 | 1.7 | 0.4 | 2 wt% in mCP | 68 |
| BIC-pCz | 471 | 41 | 0.15 | 96 | 12.5 | 328 | 7.7 | 0.3 | 0.8 | 0.31 | | |
| mDBIC | 431 | 33 | 0.31 | 68 | 6.4 | 202 | 10.6 | 4.9 | 3.0 | 0.5 | | |
| pDBIC | 539 | 30 | 0.35 | 85 | 11.3 | - | 7.5 | 1.3 | -- | - | | |
| v-DABNA-CN-Me | 496 | 17 | 0.1 | 86 | 4.9 | 10 | 17 | 28 | 0.8 | 10 | 1×10$^{-5}$M in toluene | 69 |
| TPD4PA | 445 | 19 | 0.05 | 88.1 | 7.82 | 4.69 | 7.65 | | 51.4 | 25.1 | 3 wt % emitter in mCBP-CN | 70 |
| tBu-TPD4PA | 451 | 19 | 0.06 | 90.3 | 8.07 | 5.55 | 7.09 | | 53.0 | 24.4 | | |
| tCzphB-Ph | 527 | 23 | 0.04 | 0.98 | 6.9 | | 12 | | | | 2 wt% in TPSS | 71 |
| tCzphB-Fl | 535 | 25 | 0.04 | 0.93 | 7.3 | | 11 | | | | | |
| DtBuCzB | 498 | 27 | 0.10 | 85.9 | 4.4 | 123 | 13.6 | | 9.1 | 0.88 | 5 wt% in $SF_3$-TRZ | 72 |
| TCzBN-DPF | 503 | 27 | 0.10 | 96.1 | 5.3 | 56 | 9.0 | | 9.8 | 3.45 | | |
| TCzBN-TMPh | 491 | 25 | 0.10 | 94.0 | 5.8 | 105 | 8.0 | | 9.3 | 1.83 | 1 wt% in $SF_3$-TRZ | |
| TCzBN-oPh | 495 | 24 | 0.10 | 96.1 | 5.9 | 112 | 8.6 | | 8.4 | 1.62 | | |
| t-Bu-v-DABNA | 467 | 14 | 0.04 | 91.9 | 5.79 | 1.55 | | | | | 1×10$^{-5}$M in toluene | 73 |
| | - | - | - | 91.9 | 5.79 | 2.93 | 13.8 | | 3.45 | 25.4 | 5 wt% of *t*-Bu-v-DABNA in DBFPO host film | |
| (IPr)AuBN | 511 | 30 | | 83 | 0.32 | 8.4 | 9.3 | 19 | 300 | 330 | 1×10$^{-5}$ M MeCN | 74 |
| (BzIPr)AuBN | 511 | 30 | | 85 | 0.317 | 6.5 | 8.2 | 15 | 310 | 500 | | |
| (PyIPr)AuBN | 511 | 30 | | 63 | 0.303 | 6.0 | 11 | 64 | 310 | 320 | | |
| (BzIPr)AuBNO | 471 | 30 | | 51 | 0.298 | 19.9 | 7.7 | 74 | 320 | 110 | | |

## Table S1. Summary of reported B/N-type MR-TADF emitters.

| | | | | | | | | | | | | |
|---|---|---|---|---|---|---|---|---|---|---|---|---|
| TBE01 | 459 | 21 | 0.16 | 91.1 | 6.5 | 240 | | | | 0.51 | in the SiCzCz: SiTrzCz2 host | 75 |
| TBE01 | 459 | 21 | 0.14 | 89.1 | 5.7 | 125 | | | | 1.03 | | |
| TRZCzPh-BNCz | 514 | 34 | 0.13 | 93.0 | 3.69 | 0.14 | 24.5 | | | 213 | $1\times10^{-5}$M in toluene | 76 |
| TRZTPh-BNCz | 513 | 29 | 0.11 | 94.7 | 3.80 | 0.24 | 24.2 | | | 155 | | |
| TRZCzPh-BNC | 516 | | | 97.6 | 3.43 | 5.66 | 5.7 | | | 88 | 3 wt% in CBP | |
| TRZTPh-BNCz | 516 | | | 99.0 | 3.39 | 6.37 | 6.1 | | | 75 | | |
| VTCzBN | 496 | 34 | 0.06 | 98 | 3.3 | 9.9 | 3.0 | | 27 | 100 | $5.0\times10^{-5}$ M in toluene | 77 |
| TCz-VTCzBN | 521 | 29 | <0.01 | 98 | 13.2 | 8.7 | 1.1 | | 6 | 90 | | |
| PCzBN3 | 421,487 | 42 | 0.13 | 51 | 27 | 28.8 | 0.76 | | | 8.6 | in thin film | 78 |
| PCzBN5 | 501 | 43 | 0.13 | 43 | 53 | 8.2 | 0.45 | | | 21.8 | | |
| BSS-Cz | 455 | 28 | 0.14 | 86 | 4.8 | 98.3 | 21 | | | 15 | 1 wt% in PS | 79 |
| m-Cz-BNCz | 500 | 33 | 0.15 | 98 | 6.1 | 74.6 | 13.7 | 0.28 | 2.43 | 1.29 | 3 wt% in PhCzBCz film | 80 |
| m-DPAcP-BNCz | 498 | 34 | 0.14 | 97 | 6.2 | 80.9 | 13.0 | 0.40 | 2.74 | 1.16 | | |
| m-SF-BNCz | 496 | 32 | 0.16 | 95 | 6.5 | 97.0 | 12.5 | 0.66 | 2.20 | 0.93 | | |
| *m*-BN-BNCz | 498 | 32 | 0.16 | 98 | 7.1 | 83.1 | 11.7 | 0.24 | 2.14 | 1.16 | | |
| Cl-MR | 474 | 36 | 0.13 | 85 | 4.1 | 17 | 12 | 22 | 9.8 | 9.8 | 10 wt% in DPEPO | 81 |
| Br-MR | 474 | 36 | 0.13 | 76 | 1.0 | 9.9 | 13 | 41 | 83 | 59 | | |
| TCZ-F-DABNA | 558 | 38 | 0.12 | 99 | 7.9 | 20.2 | 7.81 | | 4.85 | 7.80 | 8 wt% in PhCzBCz films | 82 |
| DBNS | 631 | 40 | 0.20 | 80 | 14.4 | 11.2 | | | | 21 | $1\times10^{-5}$M DCM | 83 |
| DBNS-*t*Bu | 641 | 39 | 0.19 | 85 | 19.1 | 10.2 | | | | 22 | | |
| mICz-DABNA | 465 | 26 | 0.20 | 92.6 | 7.1 | 17.5 | 12.3 | | 1.76 | 2.65 | 3% doped mCBP-CN film | 84 |
| BFCz-DABN | 462 | 26 | 0.20 | 93.1 | 7.9 | 18.5 | 11.1 | | 1.59 | 2.78 | | |
| C-BN | 450 | 19 | 0.21 | 100 | 5.1 | 64.8 | 20 | | 3.0 | 1.8 | $1\times10^{-5}$M toluene | 85 |
| Cz-BSeN | 479 | 30 | 0.15 | 87 | 0.9 | 29.8 | 0.44 | | 110 | 750 | 1 wt % emitters in PS | 86 |
| DCz-BSeN | 472 | 28 | 0.14 | 93 | 0.7 | 28.4 | 0.53 | | 140 | 880 | | |
| pBP-DABNA-Me | 462 | 22 | 0.176 | 98 | | 53 | | | | 6.85 | 5 wt% doped in the mCBP: DPEPO | 87 |
| BN-Se | 502 | 42 | 0.08 | 99 | 0.9 | 5.2 | 14 | - | 100 | 160 | $1\times10^{-5}$M in toluene | 88 |
| Czp-*t*BuCzB | 478 | 23 | 0.09 | 98 | 3.0 | 41.8 | | | 16.7 | 4.78 | 5 wt% in 26-DCzPPy | 89 |
| Czp-POAB | 498 | 36 | 0.13 | 96 | 5.3 | 62.4 | | | 11.8 | 1.54 | 8 wt% in 26-DCzPPy | |
| BN-ICz | 517 | 21 | 0.22 | 99.2 | | | 16 | | | | $1.0\times10^{-5}$ M in toluene | 90 |
| BN-DICz | 533 | 20 | 0.26 | 99.4 | 5.7 | 495.9 | 18 | | 8.2 | 7.8 | | |
| DBN-ICz | 542 | 18 | 0.20 | 98.6 | 5.6 | 23.7 | 17 | | 1.2 | 0.7 | | |
| BN-Y | 567 | 34 | 0.12 | 95 | 17.8 | | | | | | | 91 |
| BN-R | 624 | 46 | 0.11 | 94 | 17.8 | 71.8 | 3.8 | 4 | 1.33 | 1.13 | 1 wt % doped in NPB: DMFBD-TRZ=2: 1 | |
| PhDMAC-BN | 483 | 34 | 0.14 | 86.4 | 6.2 | 27.7 | 9.98 | 15.7 | 4.57 | 5.04 | 5wt% doped in PPF | 92 |
| LTCz-BN | 497 | 27 | 0.1 | 93 | 4.9 | 4.7 | 4.7 | | 16 | 83 | 4 wt% doped in mCPB | 93 |
| *ω*-DABNA | 509 | 22 | 0.013 | 87 | 5.9 | 8.9 | 14 | 21 | 1 | 12 | 4 wt% doped in PMMA | 94 |
| *ω*-DABNA-M | 514 | 23 | 0.01 | 87.4 | 5.60 | 5.04 | | | | 21 | 1 wt% doped in PMMA | 95 |
| *ω*-DABNA-PH | 512 | 28 | 0.018 | 86.5 | 4.62 | 4.70 | | | | 22 | | |
| p-1-PCzBN | 489 | 25 | 0.14 | 92 | 6.4 | 9.7 | 14 | / | 2.8 | 7.0 | 4 wt% doped in 2,6-DCzppy | 96 |
| m-1-PCzBN | 502 | 27 | 0.09 | 95 | 6.1 | 17.9 | 16 | / | 3.3 | 6.3 | | |
| DTBA-BN2 | 490 | 41 | 0.13 | 96 | 6.3 | 4.2 | 13 | | | 23 | $1.0\times10^{-5}$ M in toluene | 97 |

# Table S1. Summary of reported B/N-type MR-TADF emitters.

| | | | | | | | | | | | | |
|---|---|---|---|---|---|---|---|---|---|---|---|---|
| DTBA-B2N3 | 471 | 23 | 0.10 | 97 | 2.4 | 1.1 | 22 | | | 160 | | |
| BNCz-pTPA | 487 | 22 | 0.11 | 95 | 5.60 | 23.97 | 11.1 | 5.82 | 8.9 | 44.5 | 1 wt% in mCBP | 98 |
| BNCz-mTPA | 489 | 22 | 0.12 | 92 | 5.31 | 29.42 | 12.4 | 6.57 | 15.1 | 22.9 | | |
| DPMX-CzDABNA | 481 | 29 | 0.11 | 94.2 | 4.3 | 14.8 | 24 | | 5.4 | 6.6 | 15 wt% in SF3-TRZ doped film | 99 |
| BN-STO | 517 | 34 | 0.13 | 96 | 5.4 | 25.3 | 5.7 | | 12.5 | 12 | 5 wt% in DMIC-TRZ doped film | 100 |
| BN-XTO | 515 | 33 | 0.08 | 93 | 7.1 | 57.1 | 7.6 | | 5.9 | 3 | | |
| BNNO | 637 | 32 | 0.09 | 95 | 13.8 | 69.5 | 7.3 | | | 1.4 | $1.0\times10^{-5}$ M in toluene | 101 |
| [B-N]N | 442 | 19 | 0.28 | 84 | 4.4 | | 19.1 | | | | $1.0\times10^{-5}$ M in toluene | 102 |
| p[B-N]O | 488 | 19 | 0.43 | 93 | 4.3 | | 21.6 | | | | | |
| p[B-N]NO | 522 | 28 | 0.40 | 90 | 4.7 | | 19.1 | | | | | |
| p[B-N]N | 547 | 26 | 0.36 | 86 | 4.0 | | 21.5 | | | | | |
| DBTN-2 | 512 | 20 | 0.06 | 92 | 3.5 | 6.5 | 27 | | 2.2 | 17 | 2.5 wt% in SF3-TRZ film | 103 |
| $\alpha$-3BNMes | 442 | 30 | 0.28 | 63 | 10 | 9.08, 7060 | 5.25 | | 1.98 | 0.059 | 2.5 wt% in mCP film | 104 |
| NOBNacene | 410 | 38 | 0.30 | 71 | 2.9 | 1180 | 1.18 | | 26.1 | 0.374 | 1.5 wt % in TSPO1 film | 105 |
| p-CzB | 513 | 41 | 0.14 | 80 | 9.6 | 41 | 3.7 | | | 4.6 | 1 wt % in mCBP-CN film | 106 |
| m-CzB | 517 | 40 | 0.09 | 85 | 6.0 | 15 | 4.1 | | | 23 | | |
| BN-PhOH | 494 | 26 | 0.14 | 80 | 3.4 | 40.9 | 20 | 5.0 | 4.6 | 2.85 | 3 wt% in mCBP film | 107 |
| BNPhOCH$_3$ | 494 | 26 | 0.15 | 78 | 4.4 | 40.8 | 15 | 4.2 | 3.7 | 2.98 | | |
| BN-PhN(CH$_3$)$_2$ | 500 | 24 | 0.14 | 71 | 3.8 | 21.2 | 11 | 4.5 | 11 | 8.10 | | |
| TMlnBN | 475 | 23 | 0.39 | 66 | 5.5 | 14.7 | 8.9 | | 9.3 | 4.6 | $1.0\times10^{-5}$ M in toluene | 108 |
| MeS-TMlnBN | 475 | 23 | 0.40 | 70 | 6.9 | 24.5 | 6.1 | | 8.4 | 4.6 | | |
| Cz-TMlnBN | 470 | 22 | 0.41 | 66 | 5.5 | 15.8 | 7.8 | | 10.4 | 5.9 | | |
| TCz-TMlnBN | 470 | 22 | 0.42 | 69 | 5.7 | 17.9 | 7.9 | | 9.6 | 5.4 | | |
| BN1 | 401 | 36 | 0.40 | 98 | 7.03 | | 11.7 | | | | $1.0\times10^{-5}$ M in toluene | 109 |
| BN2 | 415 | 28 | 0.28 | 98 | 5.70 | 16240 | 12.4 | | | | | |
| BN3 | 420 | 29 | 0.37 | 86 | 5.54 | 13340 | 12.1 | | | | | |
| BN4 | 417 | 25 | 0.30 | 86 | 4.96 | 4190 | 14.0 | | | | | |
| NBO | 434 | 25 | 0.13 | 99 | 7.7 | 86.6 | 11.7 | | 1.4 | 1.2 | 3 wt % in mCBP | 110 |
| m-DiNBO | 450 | 17 | 0.06 | 94 | 3.4 | 31.4 | 22.7 | | 6.8 | 3.1 | | |
| p-DiNBO | 489 | 19 | 0.61 | 96 | 7.3 | 79.5 | 10.3 | | 3.4 | 1.4 | | |
| NO-DBMR | 458 | 14 | 0.04 | 83.4 | 6.64 | 5.46 | 15.1 | | 4.16 | 10.1 | 5wt% in DBFPO film | 111 |
| Cz-DBMR | 480 | 14 | 0.04 | 82.1 | 7.54 | 1.96 | 13.3 | | 4.55 | 37.2 | | |
| CzBN1 | 471/533 | 78 | 0.13 | 95 | 33.2 | 1.4 | 1.0 | | 2.0 | 206.5 | $1.0\times10^{-5}$ M in toluene | 112 |
| CzBN2 | 477/522 | 75 | 0.14 | 97 | 21.8 | 2.7 | 2.1 | | 2.4 | 78.2 | | |
| CzBN3 | 478 | 21 | 0.15 | 99 | 10.3 | 4.7 | 6.3 | | 3.5 | 32.7 | | |
| BN-TC | 464 | 22 | 0.14 | | | | | | | | $1.0\times10^{-5}$ M in toluene | 113 |
| BN-AC | 479 | 26 | 0.14 | 94 | 3.8 | 9.7 | 22.4 | | 2.7 | 11 | 15 wt% in mCBP | |
| BN-PXZ | 480 | 28 | 0.12 | 97 | 8.9 | 1.7 | 8.7 | | 2.3 | 75 | | |
| BN-PZ | 634 | 82 | 0.13 | 98 | 39.4 | 1.2 | 1.2 | | 1.3 | 185 | | |
| Tp-DABNA | 463 | 24 | 0.15 | 99 | 4.93 | 200.5 | 17.5 | | 2.62 | 0.57 | 2 wt% in mCBP:DPEPO | 114 |
| p-TBNCz | 490 | 29 | 0.12 | 92 | 7.2 | 92.0 | 10.1 | | 2.9 | 1.4 | 1 wt % in PhCzBCz | 115 |
| m-DBCz | 547 | 35 | 0.04 | 97 | 4.2 | 14.8 | 10.4 | | 13.1 | 16.5 | | |

# Table S1. Summary of reported B/N-type MR-TADF emitters.

| | | | | | | | | | | | | |
|---|---|---|---|---|---|---|---|---|---|---|---|---|
| (P)/(M)-BN-Py | 539 | 44 | 0.2 | 93 | 6.6 | 88.5 | 10 | 7.6 | 4.28 | 0.98 | 3 wt% in PhCbBCz | 116 |
| OBN | 425 | 30 | 0.20 | 50 | 7.29 | 11.1 | 3.4 | | 7.0 | 1.3 | 10 wt% in DBFDPO | 117 |
| NBN | 440 | 29 | 0.12 | 71 | 6.03 | 12.3 | 5.2 | | 9.2 | 2.9 | | |
| ODBN | 429 | 26 | 0.19 | 86 | 3.13 | 35.9 | 7.0 | | 24.0 | 2.1 | | |
| tPh[BN] | 460 | 20 | 0.63 | 92 | 2.2 | | 42 | | | | $1.0\times10^{-5}$ M in toluene | 118 |
| Cz[BN] | 470 | 27 | 0.57 | 90 | 2.2 | | 41 | | | | | |
| B4N6-Me | 580 | 19 | 0.19 | 98 | 14.6 | 138.3 | 6.4 | 1.3 | 0.34 | 0.76 | $1.0\times10^{-5}$ M in toluene | 119 |
| (M,M/P,P)-RBNN | 624 | 38 | 0.19 | 95 | 11.3 | 32.7 | 6.73 | | 2.12 | 3.19 | 1 wt% in mCPBC | 120 |
| D1-BNN | 474 | 21 | 0.15 | 68 | 13.27 | 100.47 | | | | 0.155 | 10 wt% in mCP | 121 |
| D2-BNN | 473 | 21 | 0.16 | 80 | 17.54 | 117.05 | | | | 0.235 | | |
| D3-BNN | 473 | 21 | 0.17 | 92 | 16.66 | 126.43 | | | | 0.394 | | |
| BNIP-tBuCz | 570 | 61 | 0.13 | 96 | 13.0 | 146.7 | 7.7 | | 2.0 | 0.9 | 1 wt% doped in a DMIC-TRZ host | 122 |
| BNIP-tBuDPAC | 557 | 53 | 0.11 | 98 | 13.9 | 116.4 | 7.1 | | 2.0 | 1.2 | | |
| BNIP-CzDPA | 585 | 57 | 0.12 | 95 | 12.0 | 132.8 | 8.3 | | 1.8 | 0.9 | | |
| BNDIP | 584 | 61 | 0.09 | 96 | 10.2 | 60.0 | 10 | | 2.8 | 2.3 | | |
| 1-$C_2$ | 543 | 23 | 0.35 | | | | | | | | $1.0\times10^{-5}$ M in toluene | 123 |
| 1-C1 | 520 | 28 | 0.32 | 85 | 4.5 | | | | | | | |
| Na-sBN | 516 | 31 | | 91 | 5.23 | | 17 | | | | | 124 |
| Na-dBN | 612 | 31 | | 93 | 4.76 | | 20 | | | | | |
| Cz-DABNA | 465 | 12 | 0.02 | 90.6 | 4.80 | 5.31 | 16.8 | | | 15 | $1.0\times10^{-5}$ M in toluene | 125 |
| t-BuCz-DABNA | 466 | 12 | 0.02 | 94.2 | 4.12 | 4.32 | 21.1 | | | 21 | | |
| BN-TP-N1 | 534 | 43 | 0.15 | 95 | 6.7 | 89.5 | 9.78 | 5.1 | 4.63 | 1.01 | 3 wt% in PhCzBCz | 126 |
| BN-TP-N2 | 535 | 43 | 0.13 | 91 | 6.3 | 99.6 | 10.0 | 9.8 | 4.88 | 0.83 | | |
| BN-TP-N3 | 526 | 39 | 0.17 | 97 | 6.5 | 55.4 | 9.55 | 3.0 | 5.54 | .70 | | |
| BN-TP-N4 | 530 | 39 | 0.15 | 97 | 6.1 | 49.5 | 11.6 | 3.6 | 4.44 | 1.90 | | |
| PPZ-BN | 613 | 48 | 0.16 | 88 | 12 | | | | 1.4 | 2.2 | $1.0\times10^{-5}$ M in toluene | 127 |
| Cz-DBCz | 489 | 20 | 0.16 | 93 | 4.6 | 28.4 | 14.3 | | 7.5 | 4.27 | 1.0 wt% in mCPBC | 128 |
| Cz-DBTPA | 484 | 19 | 0.23 | 90 | 5.8 | 30.7 | 12.6 | | 4.66 | 2.81 | | |
| PhO-DBCz | 477 | 27 | 0.15 | 94 | 5.1 | 28.8 | 12.5 | | 7.10 | 7.10 | | |
| TBA-BCz-BN | 472 | 28 | 0.14 | 86 | | 19 | | | | | 3 wt% in mCBP | 129 |
| PXZ-R-BN | 688 | 49 | 0.19 | 78 | 13.7 | 650 | 2.55 | | | 0.302 | 1 wt% in DMIC-TRZ | 130 |
| BCz-R-BN | 715 | 43 | 0.16 | 71 | 14.2 | 1490 | 2.69 | | | 0.129 | | |
| BSS-Ph-TBCz | 463 | 26 | 0.18 | 87 | 5.6 | 123.6 | | | | 12 | 1 wt% in PS | 131 |
| BSS-TBCz | 453 | 25 | 0.17 | 86 | 1.6 | 116.6 | | | | 14 | | |
| DB | 440 | 27 | 0.21 | 82 | 2.54 | 35.3 | 7.1 | | 31 | 13 | 2 wt% in DOBNA-OAr | 132 |
| DB-O | 443 | 24 | 0.18 | 91 | 2.47 | 35.5 | 5.9 | | 34 | 18 | | |
| DB-S | 444 | 23 | 0.18 | 95 | 2.43 | 35.7 | 5.9 | | 35 | 19 | | |
| BNB′-1 | 540 | 24.5 | 0.15 | 99 | 7.27 | 71.01 | 9.56 | | 4.21 | 1.96 | 1.5 wt% in PhCzBCz | 133 |
| TCZBAC | 492 | 35 | 0.15 | 84 | 6.0 | 38.8 | 17 | | 0.13 | 13 | 3 wt% in PhCzBCz | 134 |
| TCZBAO | 507 | 30 | 0.10 | 91 | 5.9 | 32.6 | 17 | | 0.13 | 16 | | |
| h-BNCO-1 | 516 | 28 | 0.03 | 75 | 6.3 | 3.6 | 9.35 | | 6.56 | 17.9 | 1 wt% in mCBP | 135 |
| Py-Cz-BN | 586 | 40 | 0.19 | 98.8 | 3.8 | 739 | 24 | | | 0.15 | 1 wt% in mCBP | 136 |

# Table S1. Summary of reported B/N-type MR-TADF emitters.

| | | | | | | | | | | | | |
|---|---|---|---|---|---|---|---|---|---|---|---|---|
| PSeZBN1 | 480 | 31 | 0.164 | 98 | 4.3 | 11.3 | 14.8 | | 8.1 | 10 | 1 wt% in DMIC-TRZ | 137 |
| PSeZBN2 | 519 | 53 | 0.065 | 99 | 3.0 | 3.1 | 4.9 | | 28.0 | 220 | | |
| BN[9]H | 578 | 47 | 0.15 | 98 | 33.3 | 51.1 | 1.4 | | 1.60 | 3.85 | 2 wt % in DMIC-TRZ | 138 |
| 6z | 516 | 22 | 0.10 | 98 | 13.5 | 43.7 | 3.2 | | | 5.2 | 1 wt% in DMIC-TRZ | 139 |
| 10b | 471 | 22 | 0.07 | 99 | 5.9 | 84.3 | 9.7 | | | 2.0 | 1 wt% in DOBNA-OAr | |
| BO-DICz | 507 | 18 | 0.32 | 90.7 | 8.0 | 302.1 | 7.4 | | | 0.59 | $1.0\times10^{-5}$ M in toluene | 140 |
| TPABO-DICz | 515 | 17 | 0.28 | 82.8 | 5.2 | 406.3 | 5.4 | | | 0.72 | | |
| B-N-1 | 455 | 32 | | 81 | 3.4 | 169 | 16 | | 9.7 | 0.88 | 3 wt % in mCBP | 141 |
| B-N-S-1 | 490 | 44 | | 85 | 4.4 | 66.7 | 10.9 | | 9.9 | 2.66 | | |
| B-N-S-2 | 462 | 38 | | 80 | 3.9 | 85.1 | 11.0 | | 11.8 | 2.08 | | |
| B-N-S-3 | 458 | 28 | | 90 | 3.8 | 0 | 24.4 | | 3.8 | 0 | | |
| BN-Cz | 561 | 52 | 0.17 | 91 | 9.4 | 340 | 5.95 | 0.59 | 4.10 | 0.27 | 3 wt % in PhCzBCz | 142 |
| BN-Cb | 559 | 47 | 0.22 | 82 | 10.1 | 1150 | 6.49 | 1.42 | 1.99 | 0.07 | | |
| PCzDBN1 | 476 | | 0.06 | 57 | 2.4 | 22.2 | 12.6 | | | 8.5 | Neat film | 143 |
| PCzDBN3 | 479 | | 0.06 | 54 | 2.4 | 16.1 | 10.8 | | | 12.9 | | |
| PCzDBN5 | 480 | | 0.04 | 28 | 1.9 | 11.4 | 5.6 | | | 23.1 | | |
| BN-36Cz-BN | 497 | 33 | 0.125 | 65 | 6.1 | 39.4 | 0.17 | | | 16.0 | 5 wt % emitters in DMIC-TRZ | 144 |
| BN-27Cz-BN | 499 | 32 | 0.124 | 66 | 5.7 | 32.8 | 0.44 | | | 8.0 | | |
| 2CB-BuDABNA | 472 | 26 | 0.14 | 93 | 9.30 | 69.1 | 7.28 | | 3.48 | 1.66 | 5 wt% in SiCzCz: SiTrzCz2 (70: 30 wt%) films | 145 |
| BuDABNA | 459 | 27 | 0.157 | 92 | 9.19 | 92.6 | 9.09 | | 1.79 | 0.66 | | |
| 3CB-BuDABNA | 495 | 32 | 0.17 | 26 | 5.59 | 56.4 | | | | | 5 wt.%-doped PMMA film | |
| IDAD-BNCz | 492 | 27 | 0.06 | 96 | 5.7 | 20.2 | 1.93 | | 15.5 | 43.2 | 1 wt.% in PhCzBCz | 146 |
| TIDAD-BNCz | 492 | 28 | 0.06 | 95 | 5.1 | 19.4 | 1.97 | | 17.5 | 48.9 | | |
| MFCzBN | 500 | 34 | 0.20 | 90 | 5.99 | 3233 | 15 | | | | 1 wt% doped PMMA film | 147 |
| SFCzBN | 500 | 35 | 0.19 | 91 | 6.066 | 810 | 15 | | | | | |
| PTZBNO | 612 | 47 | 0.27 | 97 | | 27100 | 5.5 | | | | $1.0\times10^{-5}$ M in toluene | 148 |
| PXZBNO | 627 | 45 | 0.30 | 94 | | 61500 | 6.5 | | | | | |
| CzBN-tDPA | 478 | 32 | 0.13 | 86 | 4.8 | 8.5 | 10.2 | | 1.81 | 38.7 | 1 wt% doped 26DCzPPy film | 149 |
| CzBN-mCP | 475 | 29 | 0.14 | 83 | 5.2 | 12.1 | 7.2 | | 6.12 | 2.52 | | |
| tBOSi | 414 | 28 | 0.26 | 81 | 6.36 | | | | | | 5 wt% doped mCPCN film | 150 |
| tBOSiCz | 414 | 28 | 0.21 | 85 | 6.05 | | | | | | | |
| tCzMe3Si | 495 | 24 | 0.14 | 94 | 2.4 | 88.6 | | | | 1.4 | $5.0\times10^{-5}$ M in toluene | 151 |
| tCzPh3Si | 496 | 23 | 0.10 | 95 | 3.4 | 104.9 | | | | 1.5 | | |
| tPhCzMe3Si | 506 | 25 | 0.15 | 96 | 4.1 | 98.4 | | | | 1.3 | | |
| tPhCzPh3Si | 508 | 25 | 0.12 | 96 | 3.2 | 100.4 | | | | 1.4 | | |
| tCzBN-PQ | 515 | 31 | 0.11 | 89 | 6.6 | 11.4 | 2.0 | | 13.1 | 5.9 | 3 wt% doped 26DCzPPy films | 152 |
| tCzBN-PQCz | 511 | 30 | 0.09 | 95 | 5.4 | 8.6 | 2.8 | | 15.4 | 7.3 | | |
| CzBN | 473 | 25 | 0.15 | 99 | 6.0 | 75 | 15.5 | | 1.2 | 1.24 | 1 wt% in mCBP | 153 |
| CzBNNa | 483 | 25 | 0.15 | 98 | 5.6 | 48 | 11.4 | | 6.43 | 3.07 | | |
| CzBNPyr | 480 | 25 | 0.61 | 90 | 5.1 | - | 17.6 | | 1.97 | - | | |
| CzCzB | 559 | 48 | 0.20 | 87 | 8.9 | 217 | 6.2 | | 5.0 | 0.59 | 1 wt% in mCBP | 154 |
| RBNO1 | 629 | 39 | 0.25 | 95 | | 26400 | 5.6 | | | | 1 wt% in the DMIC-TRZ | 155 |

## Table S1. Summary of reported B/N-type MR-TADF emitters.

| | | | | | | | | | | | | |
|---|---|---|---|---|---|---|---|---|---|---|---|---|
| RBNO2 | 645 | 39 | 0,26 | 98 | | 25300 | 6.2 | | | | | |
| DCzBNO | 468 | 36 | 0.08 | 95 | 9.8 | 129.0 | 6.88 | | 2.96 | 1.09 | 5 wt% in 26DCzPPy | 156 |
| TCzBNO | 497 | 48 | 0.16 | 90 | 7.2 | 74.5 | 4.38 | | 9.03 | 3.84 | | |
| SAC2MN1B | 468 | 30 | 0.12 | 75.5 | 5.2 | 44.8 | 10.2 | 3.31 | 5.71 | 1.27 | 1 wt% in CzSi | 157 |
| DPA2MN2B | 449 | 29 | 0.17 | 51.2 | 3.2 | 42.1 | 27.3 | 2.60 | 25.9 | 0.62 | | |
| Cz2MN2B | 481 | 23 | 0.12 | 85.2 | 17.9 | 31.9 | 3.3 | 0.57 | 1.71 | 2.27 | | |
| SAC2MN2B | 446 | 21 | 0.18 | 66.1 | 4.1 | 32.6 | 10.6 | 5.46 | 8.29 | 1.34 | | |
| IDIDBN | 529 | 28 | 0.31 | 76 | 15.6 | | 4.9 | | | | 1 wt% in DMIC-TRZ | 158 |
| tBuIDIDBN | 532 | 27 | 0.30 | 78 | 14.7 | | 5.3 | | | | | |
| OP-BN | 473 | 36 | 0.16 | 84.2 | 9.97 | 104 | 10.0 | | 3.21 | 0.716 | 8 wt% in mCBPCN | 159 |
| Cz-OP-BN | 474 | 36 | 0.18 | 99.2 | 10.1 | 122 | 9.90 | | 1.73 | 0.948 | | |
| 2Cz-OP-BN | 480 | 38 | 0.15 | 100 | 11.4 | 144 | 8.77 | | 2.45 | 0.963 | | |
| CFDBO | 458 | 21 | 0.27 | 94 | 3.25 | 60.79 | 28.5 | 1.82 | 0.458 | 1.67 | 2 wt% in mCBP | 160 |
| CFDBA | 472 | 18 | 0.19 | 96 | 3.01 | 70.51 | 30.3 | 1.26 | 1.696 | 1.49 | | |
| CFDBCz | 487 | 21 | 0.17 | 96 | 4.49 | 32.66 | 21.0 | 0.88 | 0.394 | 3.12 | | |
| CzBN4 | 483 | 14 | 0.0042 | 91.6 | 5.29 | 5.02 | 16 | 1.4 | 2.7 | 18 | 0.1 wt %-doped PS films | 161 |
| CzBN4-oPh | 478 | 16 | 0.087 | 92.5 | 5.24 | 16.0 | 15 | 1.2 | 3.7 | 6.4 | | |
| CzBN6 | 488 | 12 | 0.0031 | 94.0 | 3.34 | 2.93 | 26 | 1.7 | 3.4 | 30 | | |
| CzBN8 | 491 | 12 | 0.003 | 92.2 | 3.14 | 1.41 | 26 | 2.2 | 4.8 | 65 | | |
| Π-CzBN | 486 | 16 | 0.04 | 97.7 | 2.8 | 5.1 | 23 | | 13 | 29 | 1 wt% in mCP | 162 |
| BN-NAP | 511 | 26 | 0.47 | 81 | 9.5 | | 9 | | | | $1.0\times10^{-5}$ M in toluene | 163 |
| BN-ANAP | 518 | 20 | 0.47 | 93 | 9.3 | | 10 | | | | | |
| BNCz-aDMAC | 475 | 23 | 0.13 | | 2.6 | 21.6 | 9.5 | | 30 | 18.6 | 5 wt%in PhCzBCz film | 164 |
| BNCz-PaDMAC | 490 | 24 | 0.06 | | 5.6 | 26,7 | 7.0 | | 11 | 9.4 | 7 wt%in PhCzBCz film | |
| BO-N1 | 493 | 28 | 0.15 | 20.5 | 14.8 | 85 | 0.259 | | | 15.4 | 0.3% in CzAcSF | 165 |
| BO-N2 | 503 | 24 | 0.13 | 18.0 | 13.8 | 90 | 0.445 | | | 14.2 | 0.8wt% in CzAcSF | |
| BNCz-SAF | 484 | 25 | 0.15 | 85.5 | 5.3 | 1.74 | 12.6 | | 4.14 | 73.6 | 3wt% in mCBP | 166 |
| BNCz-DMAC | 484 | 26 | 0.13 | 82.7 | 4.69 | 1.05 | 14.0 | | 4.41 | 120 | | |
| BNCz-PXZ | 482 | 34 | 0.12 | 83.6 | 5.01 | 5.17 | 10.4 | | 7.54 | 31.1 | | |
| TTABN | 459 | 21 | 0.06 | 92 | 8.3 | 65.5 | 6.02 | | 5.50 | 3 | 2wt% in mCPCN | 167 |
| TAzBN | 464 | 35 | 0.032 | 94 | 7.4 | 7.9 | 1.89 | | 11.5 | 8.5 | 10wt% in mCPCN | |
| Me-PABO | 453 | 21 | 0.16 | 87.5 | 6.12 | 61.75 | 6.42 | 9.12 | 8.67 | 3.5 | 5 wt% doped in PPF | 168 |
| Me-PABS | 463 | 21 | 0.05 | 92.1 | 3.15 | 39.67 | 13.16 | 11.29 | 17.42 | 5.59 | | |
| DG7 | 552 | 20 | 0.19 | 68 | 6 | 1400 | 10.4 | | 0.85 | 0.0043 | 1 wt% doped in ZEONEX | 169 |
| BN-Ad | 486 | 21 | 0.09 | 98 | 6.1 | 35.0 | 13.0 | | 3.18 | 3.54 | 6 wt% doped in mCBP | 170 |
| BN-Ph | 487 | 24 | 0.09 | 96 | 5.1 | 47.6 | 14.9 | | 4.70 | 2.65 | | |
| DPC | 458 | 44 | 0.14 | 96.2 | 11.5 | 166 | 5.28 | 2.08 | 3.21 | 0.557 | 6 wt% doped in BCPO | 171 |
| DTP | 470 | 50 | 0.17 | 92.0 | 10.3 | 222 | 6.54 | 5.68 | 2.61 | 0.378 | | |
| CzCzBNO | 593 | 35 | 0.29 | 87 | 17.4 | | 5.0 | | | | $1.0\times10^{-5}$ M in toluene | 172 |
| CzIDBNO | 633 | 38 | 0.33 | 92 | 15.0 | | 6.1 | | | | | |
| IDIDBNO | 663 | 39 | - | 89 | 13.8 | | 6.4 | | | | | |
| A-BN | 455 | 22 | 0.09 | 97.2 | 6.76 | 18.36 | | | | 7.5 | $1.0\times10^{-5}$ M in toluene | 173 |

# Table S1. Summary of reported B/N-type MR-TADF emitters.

| | | | | | | | | | | | | |
|---|---|---|---|---|---|---|---|---|---|---|---|---|
| v-DABNA-Az1 | 458 | 19 | 0.007 | 87 | 4.8 | 5.0 | 16 | 23 | 3.0 | 23 | 1 wt% doped in PMMA | 174 |
| v-DABNA-Az2 | 458 | 19 | 0.016 | 82 | 4.4 | 3.8 | 16 | 35 | 3.5 | 31 | | |
| v-DABNA-Az3 | 458 | 19 | 0.012 | 86 | 4.5 | 3.4 | 16 | 28 | 3.2 | 34 | | |
| 3Cz-BO | 410 | 26.2 | 0.33 | 65 | 10.69 | - | 5.58 | | 6.9 | - | $1.0\times10^{-5}$ M in toluene | 175 |
| 5Cz-BO | 414 | 28.9 | 0.16 | 82 | 5.81 | 12.65 | 3.93 | | 13.3 | 27 | | |
| DABNA-3B | 470 | 19 | 0.13 | 94 | 1.79 | 6.10 | 9.98 | | 45.25 | 86.3 | 2 wt% in mCBP | 176 |
| BCzBN-3B | 482 | 16 | 0.10 | 99 | 7.04 | 25.4 | 3.80 | | 10.37 | 14.6 | | |
| pPSe-BN | 497 | 35 | 0.145 | 95 | 2.7 | 10.0 | 6.19 | | 30.9 | 56.2 | 3 wt% in pPhBCzPh | 177 |
| mPSe-BN | 494 | 33 | 0.133 | 97 | 6.9 | 30.5 | 11.1 | | 3.33 | 3.70 | | |
| R/S-BA23CzBN | 490 | 27 | 0.16 | 95 | 8.99 | 13.54 | 4.8 | | | 16 | $1.0\times10^{-5}$ M in toluene | 178 |
| R/S-BA34CzBN | 515 | 34 | 0.14 | 97 | 9.58 | 8.00 | 4.16 | | | 30.6 | | |
| V-DABNA | 481 | 17/0.093 | 0.0085 | 90 | 6.9 | 1.9 | 12 | 1.3 | 1.1 | 57 | 1 wt% in PMMA | 179 |
| V-DABNA-F | 464 | 16/0.097 | 0.0046 | 81 | 6.6 | 1.7 | 11 | 2.7 | 1.4 | 65 | | |
| V-DABNA-Mes | 484 | 16 | | 80 | 7.0 | 1.1 | 11 | 2.7 | 0.73 | 44 | | |
| BpIC-DPA | 527 | 25 | 0.17 | 87.9 | 44.9 | 31900 | 1.94 | | 0.29 | 2.49 | 2 wt% BpIC-DPA and 4 wt% BpIC-Cz in DIC-TRZ film | 180 |
| BpIC-Cz | 534 | 22 | 0.18 | 84.2 | 34.0 | 34400 | 2.44 | | 0.5 | 2.47 | | |
| BDBF-BOH | 458 | 27/0.16 | 0.12 | 90 | 7.2 | 20.2 | 14 | | | 6.9 | 10 wt% in 26-DCzPPy | 181 |
| BDBT-BOH | 459 | 27/0.16 | 0.11 | 91 | 8.2 | 14.8 | 12 | | | 13.0 | | |
| BN-N-TPA | 497 | 31 | 0.12 | 91 | 8.0 | 122.8 | 10.6 | 1.04 | 0.91 | 0.67 | 3 wt% in PhCbBCz | 182 |
| TPA-Cz-BN | 490 | 26 | 0.14 | 95 | 6.6 | 85.9 | 11.4 | 0.6 | 3.16 | 1.05 | | |
| TPA-PCz-BN | 489 | 23 | 0.14 | 92 | 5.0 | 44.3 | 13.8 | 1.2 | 5.02 | 1.91 | | |
| BN-PCz-TPA | 487 | 24 | 0.15 | 96 | 6.4 | 49.8 | 11.1 | 0.46 | 4.05 | 1.85 | | |
| f-DOABNA | 444 | 25 | 0.06 | 90 | 6.2 | 0.5 | 12.1 | | 2.8 | 230 | 3 wt% in DOBNA-Tol | 183 |
| 3tCzBN | 474 | 24/125.7 | 0.15 | 91 | 6.1 | 80.4 | 7 | | 8.9 | 2.72 | 1 wt% in DOBNA-OAr | 184 |
| Py-BN | 446 | 23/154.3 | 0.18 | 93 | 5.8 | 222.9 | 11 | | 5.1 | 0.64 | | |
| Pm-BN | 414 | 20/120.4 | 0.35 | 94 | 3.3 | 246.7 | 28 | | 0.9 | 0.42 | | |
| DOB2-DABNA-A | 451 | 27 | 0.0036 | 92 | 6.21 | 1.55 | 8.6 | 7.6 | 6.8 | 112 | 1 wt% in PMMA | 185 |
| DOB2-DABNA-A-NP | 446 | 29 | 0.018 | 76 | 2.39 | 4.41 | 8.2 | 26 | 19 | 115 | | |
| DOB2-DABNA-B-NP | 471 | 28 | 0.0065 | 87 | 7.54 | 2.99 | 10 | 16 | 1.4 | 38 | | |
| QB-U | 450 | 21/0.13 | 0.19 | 84 | 2.8 | 21.9 | 8.7 | - | 2.6 | 16 | 1 wt% DOBNA-OAr- film | 186 |
| QB-J | 454 | 15/0.09 | 0.15 | 95 | 2.0 | 4.5 | 15.0 | - | 3.4 | 69 | | |
| QB-I | 464 | 13/0.07 | ~0 | 99 | 2.0 | 0.8 | 23.8 | - | 2.7 | 272 | | |
| DPA-B2 | 444 | 31 | 0.17 | 91 | 3.1 | 40.4 | 8.95 | | 22.4 | 8 | 1 wt% SiTrzCz2 film | 187 |
| DPA-B3 | 451 | 16 | 0.14 | 97 | 2.6 | 9.2 | 10.1 | | 28.0 | 40 | | |
| DPA-B4 | 458 | 14 | 0.09 | 99 | 2.0 | 2.5 | 8.66 | | 41.3 | 229 | | |
| Cz-B4 | 457 | 28 | 0.13 | 94 | 3.9 | 11.7 | 6.85 | | 18.4 | 30 | | |
| BNCZ-DPAB | 625 | 51 | 0.10 | 99 | 7.97 | 79.4 | 2.52 | | 10 | 6.2 | 2 wt% CBP film | 188 |
| AN-BN | 601 | 48/0.16 | - | 94.2 | 15.2 | - | 6.2 | - | - | - | | 189 |
| (rac$_{mt}$)-2 | 496 | 37 | 0.13 | 1 | 9.29 | 38.38 | 8.7 | | 2.0 | 3.3 | $1.0\times10^{-5}$ M in toluene | 190 |
| M-2 | 494 | 22 | 0.16 | 0.99 | 5.09 | 44.56 | 1.7 | | 3.1 | 2.7 | | |
| 2-$C_2$ | 509 | 17.5 | 0.47 | 81 | 8.2 | | | | | | $1.0\times10^{-5}$ M in toluene | 191 |
| 2-$C_1$ | 500 | 16 | 0.39 | 85 | 4.5 | | | | | | | |

## Table S1. Summary of reported B/N-type MR-TADF emitters.

| | | | | | | | | | | | | |
|---|---|---|---|---|---|---|---|---|---|---|---|---|
| 3-$C_2$ | 485 | 17 | 0.57 | 59 | 8.5 | | | | | | | |
| TB-PB | 473 | 12 | 0.03 | 99 | 3.9 | 0.53 | 23 | | | 26 | 1.0×10-5 M in toluene | 192 |
| L-DABNA-1 | 534 | 33/0.14 | 0.088 | 95 | 8.50 | 9.68 | 8.02 | | 3.75 | 12.8 | 1 wt% SF3TRZ film | 193 |
| BNSi | 477 | 22 | 0.13 | 98 | 8.5 | 90 | 1.16 | | 11.5 | 10.1 | 15 wt% PhCzBCz | 194 |
| $CH_2$-SFBN | 492 | 25/0.12 | 0.13 | 85 | 5.65 | 89.07 | | | | 0.81 | $1.0\times10^{-5}$ M in toluene | 195 |
| O-SFBN | 488 | 23/0.12 | 0.14 | 98 | 5.03 | 97.90 | | | | 0.81 | | |
| S-SFBN | 488 | 24/0.12 | 0.14 | 99 | 5.08 | 18.99 | | | | 5.16 | | |
| Se-SFBN | 490 | 24/0.12 | 0.13 | 97 | 3.91 | 8.98 | | | | 10.50 | | |
| CO-SFBN | 489 | 24/0.12 | 0.14 | 76 | 3.99 | 8.70 | | | | 6.64 | | |
| FSBN | 621 | 55/0.18 | 0.05 | 98 | 12.5 | 51.6 | 7.11 | 1.5 | 0.74 | 1.86 | 1 wt% mCBP film | 196 |
| S-BN | 594 | 54 | 0.14 | 99.2 | 9.24 | 15.30 | 4.35 | | | 16.1 | $1.0\times10^{-5}$ M in toluene | 197 |
| 2S-BN | 671 | 54 | 0.01 | 98.4 | 10.68 | 10.68 | 3.30 | | | 36.6 | | |
| DtCzBN-CNBT1 | 502 | 29 | 0.10 | 90 | 3.8 | 75.5 | 18 | | | 2.0 | $1.0\times10^{-5}$ M in toluene | 198 |
| DtCzBN-CNBT2 | 500 | 28 | 0.12 | 97 | 5.1 | 15.3 | 16 | | | 10.6 | | |
| Exo-D1 | 452 | 29 | 0.25 | 68 | 1.9 | 111.0 | | | | 10 | $1.0\times10^{-5}$ M in toluene | 199 |
| Endo-D1 | 473 | 32 | 0.14 | 71 | 3.6 | 114.1 | | | | 22 | | |
| Exo-D2 | 450 | 28 | 0.26 | 83 | 1.8 | 146.7 | | | | 11 | | |
| Endo-D2 | 470 | 30 | 0.12 | 93 | 3.6 | 106.8 | | | | 47 | | |
| TBN3 | 566 | 31/12 | 0.11 | 91 | 8.39 | 26.68 | 3.58 | | 8.34 | 12.5 | $1.0\times10^{-5}$ M in toluene | 200 |
| BNTPA | 613 | 42/0.14 | 0.10 | 94 | 10.15 | 28.28 | 2.17 | | 1.61 | 16.1 | | |
| (rac)-S-AX-BN | 489 | 21 | 0127 | 93 | 4.9 | 39 | 0.90 | | 10.7 | 6.21 | 5 wt.% in 2,6-DCzPPy | 201 |
| (rac)-SO2-AX-BN | 495 | 20 | 0.140 | 92 | 4.5 | 73 | 1.80 | | 2.63 | 2.71 | | |
| BN-TP-ICz | 531 | 36 | 0.19 | 93 | 5.8 | 138.5 | 13.2 | 1.15 | 2.92 | 0.61 | 5 wt.% in 2,6-PhCzBCz | 202 |
| (R/S)-4-POtBuCzB | 493 | 20 | 0.16 | 97 | 8.4 | 62.8 | | | | | 10 wt% and 8 wt% in mCBP | 203 |
| (rac)-2-POtBuCzB | 498 | 27 | 0.04 | 99 | 7.5 | 37.4 | | | | | | |
| DBN-NaMe | 512 | 16/0.075 | | 91 | 7.5 | | 12 | | | | $1.0\times10^{-5}$ M in toluene | 204 |
| DBN-NaPh | 520 | 17/0.078 | | 97 | 6.9 | | 14 | | | | | |
| DBN-NaPh-d | 521 | 17/0.076 | | 96 | 5.8 | | 16 | | | | | |
| QB-DPA | 495 | 15/0.071 | 0.10 | 98 | 5.8 | 6.8 | 9.8 | | 7.2 | 25 | 1 wt% DMIC-TRZ | 205 |
| QB-PXZ | 508 | 15/0.067 | 0.09 | 97 | 6.3 | 9.7 | 7.9 | | 7.8 | 20 | | |
| NT-2B | 510 | 15 | 0.26 | 92.5 | 11.8 | 9900 | 7.9 | | 3.4 | $3.1\times10^{-4}$ | 1wt% doped in p-PhBCzPh | 206 |
| NT-3B | 511 | 14 | 0.25 | 80.0 | 12.4 | 3700 | 6.4 | | 0.6 | $6\times10^{-5}$ | | |
| Cz-CN-BN | 500 | 31/0.15 | 0.08 | 97 | 6.5 | 14.7 | 10 | | 5.1 | 11 | 5 wt% in PhCzBCz | 207 |
| TPA-CN-BN | 502 | 33/0.16 | 0.06 | 98 | 5.4 | 11.4 | 11 | | 7.2 | 14 | | |
| PTZ-CN-BN | 504 | 35/0.17 | 0.06 | 92 | 5.5 | 14.9 | 11 | | 6.1 | 10 | | |
| A-BN | 462 | 18 | 0.14 | 97.4 | 20.4 | 5.4 | 2.8 | | 2.1 | 31 | 1 wt.% in p-PhBCzPh | 208 |
| DA-BN | 473 | 16 | 0.13 | 99.1 | 24.8 | 11.3 | 1.5 | | 2.5 | 11 | | |
| A-DBN | 482 | 14 | 0.14 | 99.2 | 32.3 | 48.66 | 0.47 | | 2.6 | 13 | | |
| SFDBN-CN | 482 | 14 | 0.25 | 96 | 3.6 | 24 | 9.1 | | | 12 | $1.0\times10^{-5}$M in toluene | 209 |
| MeAuBN | 453 | 46/0.26 | 0.15 | 82 | 4.3 | 7.0 | 4.32 | 9.48 | 18.0 | 63.1 | 1 wt.% in mCP | 210 |
| iPrAuBN | 448 | 29/0.18 | 0.15 | 87 | 2.1 | 7.8 | 6.37 | 9.52 | 40.3 | 83.4 | | |
| ICz-BO | 413 | 34 | 0.48 | 53 | 3.32 | $10.10\times10^{-3}$ | 9.5 | | | $1.66\times10^{4}$ | 2 wt.% in CBP | 211 |

## Table S1. Summary of reported B/N-type MR-TADF emitters.

| | | | | | | | | | | | | |
|---|---|---|---|---|---|---|---|---|---|---|---|---|
| GBN | 541 | 43 | 0.07 | 100 | 8.7 | 6.2 | 11 | | | 17 | 1 wt.% in SpiroAC-TRZ | 212 |
| 2FPAB | 430 | 22 | 0.17 | 66 | 5.03 | 57.01 | 9.34 | 48.1 | 5.72 | 2.46 | 6 wt% in PPF film | 213 |
| MePAB | 446 | 22 | 0.14 | 69 | 6.74 | 59.32 | 7.27 | 32.7 | 4.30 | 2.37 | | |
| MePABF | 463 | 22 | 0.19 | 93 | 8.73 | 83.07 | 7.10 | 53.5 | 3.82 | 1.81 | | |
| H[6]BN1 | 474 | 30 | 0.26 | 83 | 9.0 | | | | | | $1.0\times10^{-5}$ M in toluene | 214 |
| H[6]BN2 | 465 | 27 | 0.29 | 54 | 6.5 | | | | | | | |
| HBN | 572 | 17 | 0.22 | 82 | 10.4 | 48.7 | 6.54 | 14.4 | 1.64 | 2.48 | 2 wt% in mCBP film | 215 |
| 2PO | 380 | 26 | 0.41 | 26 | 2.23 | 47.9 | 8.3 | 236 | | 0.29 | $1.0\times10^{-5}$ M in toluene | 216 |
| c3PO | 430 | 48 | 0.29 | 74 | 3.72 | 15.1 | 15.3 | 54 | | 0.86 | | |
| D2-DBN | 468 | 12 | 0.02 | 95.6 | 6.84 | 5.56 | 12 | | 26.6 | 16.7 | 1.0×10-5 M in toluene | 217 |
| CNBN | 501 | 14 | 0.12 | 99 | 4.2 | 30.2 | 17 | | | 4.7 | 3 wt% in DMIC-TRZ film | 218 |
| MCNBN | 510 | 15 | 0.12 | 97 | 4.8 | 43.1 | 15 | | | 3.2 | | |
| PCNBN | 503 | 18 | 0.08 | 96 | 5.0 | 15.5 | 13 | | | 9.4 | | |
| PMCNBN | 512 | 19 | 0.08 | 94 | 5.7 | 24.0 | 9 | | | 7.7 | | |
| DBNDS-TPh | 517 | 16 | 0.06 | 98.5 | 17.1 | 6,95 | 3.6 | | 2.3 | 22 | 1 wt% in PhCzDCz film | 219 |
| DBNDS-DFPh | 518 | 17 | 0.08 | 97.7 | 20.0 | 8.36 | 3.4 | | 1.6 | 17 | | |
| DBNDS-CNPh | 518 | 15 | 0.08 | 90.3 | 16.8 | 8.81 | 3.3 | | 2.6 | 16 | | |
| sym - OBOICz | 461 | 18 | 0.35 | 93 | 10.3 | | 9 | | | | $1.0\times10^{-5}$ M in toluene | 220 |
| sym - OBOICz | 468 | 20 | 0.25 | 96 | 8.2 | | 11.7 | | | | | |
| 5Cz-BNO | 460 | 23.6 | 0.16 | 90 | 8.98 | 12.07 | 8.63 | | 2.47 | 5.6 | $1.0\times10^{-5}$ M in toluene | 221 |
| 5Cz-BN | 496 | 23.4 | 0.10 | 92 | 7.83 | 13.41 | 5.82 | | 6.98 | 14 | | |
| [B-N]N1 | 445 | 19 | 0.26 | 86 | 4.8 | 956.9 | | | | 0.65 | $1.0\times10^{-5}$ M in toluene | 222 |
| [B-N]N2 | 438 | 16 | 0.30 | 93 | 4.1 | 956.6 | | | | 0.57 | | |
| [B-N]N3 | 458 | 37 | 0.26 | 93 | 5.5 | 978.0 | | | | 0.63 | | |
| [B-N]N4 | 463 | 27 | 0.27 | 92 | 5.0 | 973.1 | | | | 0.63 | | |
| DCzBN-Au | 508 | 39 | 0.13 | 95 | 1.6 | 4.3 | 0.59 | | 62 | 2300 | 1 wt% in mCP film | 223 |

## Table S2. Summary of reported carbonyl / amino-type MR-TADF emitters.

| Emitter | $\lambda_{em}$ (nm) | FWHM (nm) | $\Delta E_{ST}$ (eV) | $\Phi_{PL}$ (%) | $\tau_p$ (ns) | $\tau_d$ (μs) | $k_r$ ($10^7$ $s^{-1}$) | $k_{IC}$ ($10^6$ $s^{-1}$) | $k_{ISC}$ ($10^7$ $s^{-1}$) | $k_{RISC}$ ($10^4$ $s^{-1}$) | test status | Ref. |
|---|---|---|---|---|---|---|---|---|---|---|---|---|
| QAO | 466 | 32 | 0.18 | 72.4 | - | 93.3 | - | - | - | | 5 wt% in mCP | 224 |
| 3-PhQAD | 478 | 30 | 0.18 | 73 | 5.5 | 250 | 18 | - | 14 | 1.2 | 2 wt% mCP | 225 |
| 7-PhQAD | 472 | 22 | 0.19 | 68 | 5.5 | 474 | 18 | - | 15 | 0.64 | | |
| DiKTa | 453 | 27 | -- | 26 | 5.1 | 23 | 4.9 | 140 | 0.75 | 4.6 | $10^{-5}$ M in toluene | 226 |
| Me3DiKTa | 468 | 29 | - | 37 | 6.7 | 33 | 5.4 | 91 | 0.4 | 3.1 | | |
| DiKTa | 463 | 37 | 0.2 | 75 | 4.4 | 15 | - | - | - | - | 3.5 wt% in mCP | |
| Me3DiKTa | 477 | 37 | 0.21 | 80 | 5.9 | 20 | - | - | - | - | | |
| DDiKTa | 470 | 47 | 0.21 | 29 | - | - | - | - | - | - | $10^{-5}$ M in toluene | 227 |
| | 490 | 61 | 0.16 | 72 | 7.25 | 1.17 | 1.41 | - | 3 | 63 | 9 wt% in DPEPO | |
| QA-1 | 457 | 43 | 0.29 | 94 | 1.6 | 655 | 5.9 | - | 57 | 1.5 | 3 wt% in PPCz | 228 |
| QA-2 | 465 | 37 | 0.19 | 97 | 1.6 | 48 | 1.5 | - | 61 | 85 | | |
| QA-3 | 523 | 73 | 0.19 | 99 | 4.8 | 307 | 1.3 | - | 20 | 5.3 | | |
| QA-1 | 434 | 31 | 0.32 | 10 | 1.7 | - | 5.9 | - | - | - | $10^{-5}$ M toluene | |
| QA-2 | 444 | 22 | 0.26 | 24 | 0.8 | 4.3 | 6.3 | - | 120 | 93 | | |
| QA-3 | 485 | 64 | 0.29 | 16 | 3.4 | 2.5 | 4.5 | - | 25 | 2.2 | | |
| mBDPA-TOAT | 599 | 37 | 0.23 | 73.3 | 5.4 | 137 | 5.1 | 1.9 | 11.6 | 1.7 | 8 wt% in CBP | 229 |
| pBDPA-TOAT | 603 | 48 | 0.23 | 56.9 | 5.9 | 308 | 3 | 2 | 11.8 | 0.9 | 13.0 wt% in CBP | |
| DMAC-TOAT | 656 | 105 | 0.08 | 14.5 | 22 | 37 | 0.2 | 1.3 | 3.1 | 5.9 | 2.0 wt% in CBP | |
| TOAT-5 | - | 45 | 0.34 | 0.46 | 0.17 | 0.29 | 2.91 | - | 13.8 | 0.093 | 3 wt% in mCBP | 230 |
| QAO-PhCz | 460 | 29 | 0.11 | 46.6 | | | | | | | | 231 |
| Hel-DiDiKTa | 473 | 44/0.25 | 0.15 | 1.34 | - | - | - | - | - | - | $10^{-5}$ M toluene | 232 |
| | 478 | 50 | 0.15 | 4.1 | 134 | 5.4 | 2.3E-05 | - | 0.66 | 0.041 | 1 wt% in mCP | |
| QAOCz1 | 502 | 34 | 0.26 | - | - | - | - | - | - | - | $10^{-5}$ M in toluene | 233 |
| QAOCz2 | 500 | 29 | 0.18 | - | - | - | - | - | - | - | | |
| QAOCz3 | 492 | 29 | 0.16 | - | - | - | - | - | - | - | | |
| QAOCz1 | 501 | 45 | - | 86.1 | 2.63 | 116.73 | - | - | - | - | 5 wt% in CBP | |
| QAOCz2 | 500 | 41 | - | 86.8 | 5.51 | 16.37 | - | - | - | - | | |
| QAOCz3 | 495 | 42 | - | 98.9 | 5.55 | 26.45 | - | - | - | - | | |
| BOQAO | 487 | 33 | 0.22 | 98.7 | 6 | 214 | 17 | - | 13 | 2.4 | 5 wt% in CBP | 234 |
| DiKTa | 466 | 40 | 0.2 | 70 | 4.5 | 168 | - | - | - | - | 2 wt% in mCP | 235 |
| Cz-DiKTa | 502 | 54 | 0.14 | 90 | 8.6 | 196 | - | - | - | - | | |
| Cz-Ph-DiKTa | 486 | 47 | 0.1 | 77 | 6.2 | 153 | - | - | - | - | | |
| TMCz-DiKTa | 501 | 80 | 0.08 | 71 | 8.2 | 22 | - | - | - | - | | |
| DMAC-DiKTa | 534 | 94 | 0.04 | 76 | 26.4 | 6.6 | - | - | - | - | | |
| 3Cz-DiKTa | 539 | 53 | 0.16 | 78 | 11.2 | 286 | - | - | - | - | | |
| 3TMCz-DiKTa | 577 | 110 | 0.01 | 18 | 29.1 | 3 | - | - | - | - | | |
| 3DMAC-DiKTa | 599 | 116 | 0.01 | 23 | 29.9 | 3.5 | - | - | - | - | | |
| QA-PF | 478 | 30 | 0.24 | 89 | 4.91 | 346.9 | 9.6 | | 10 | 4.9 | 3 wt% in mCP | 236 |

**Table S2. Summary of reported carbonyl / amino-type MR-TADF emitters.**

| | | | | | | | | | | | | |
|---|---|---|---|---|---|---|---|---|---|---|---|---|
| QA-PCN | 477 | 34 | 0.18 | 68 | 3.97 | 223.9 | 4.3 | | 21 | 16.1 | | |
| QA-PMO | 485 | 33 | 0.25 | 66 | 4.80 | 484.1 | 8.6 | | 12 | 2.1 | | |
| QA-PCZ | 480 | 34 | 0.21 | 71 | 5.71 | 339.1 | 5.9 | | 12 | 5.0 | | |
| QAD-Cz | 488 | 47 | 0.20 | 99.6 | 9 | 205 | 1.18 | | 9.4 | 4.4 | 1 wt% in mCP | 237 |
| QAD-2Cz | 506 | 46 | 0.16 | 99.5 | 10 | 130 | 0.9 | | 9.0 | 8.4 | 12 wt% in mCP | |
| QAD-mTDPA | 586 | 55 | 0.17 | 97.2 | 17 | 269 | 0.48 | | 5.3 | 4.4 | 1.5 wt% in CBP | |
| DQAO | 465 | 33 | 0.19 | | | | | | | | $10^{-5}$ M toluene | 238 |
| OQAO | 520 | 36 | 0.16 | | | | | | | | | |
| SQAO | 552 | 54 | 0.16 | | | | | | | | | |
| DQAO | 472 | 34 | | 59.3 | 10.09 | 110.58 | | | | | 8 wt% in mCP | |
| OQAO | 534 | 45 | | 90.2 | 5.51 | 204.65 | | | | | 5 wt% in CBP | |
| SQAO | 560 | 60 | | 65.4 | 5.55 | 78.38 | | | | | 1 wt% in mCPCN | |
| CzAO | 431 | 36 | 0.40 | 49 | 3.68 | 1340 | | | | | 3 wt% emitters in mCPCN | 239 |
| MQAO | 447 | 61 | 0.31 | 61 | 9.90 | 1024 | | | | | | |
| QPXO | 485 | 76 | 0.29 | 35 | 14.73 | 2394 | | | | | | |
| QPO | 501 | 86 | 0.27 | 71 | 10.10 | 1436 | | | | | | |
| QPO-PhCz | 447 | 58 | 0.23 | 51 | | 536 | | | | | 18 wt% emitters in DPEPO | 240 |
| tP | 427 | 60 | 0.33 | | | | | | | | in n-hexane | 241 |
| tPT | 488 | 49 | 0.36 | | | | | | | | | |
| tCPD | 520 | 60 | 0.23 | 56 | | 1426 | | | | | 1 wt% in 26DCzPPy | |
| 2tCPD | 541 | 44 | 0.25 | 71 | | 927 | | | | | | |
| tPD | 491 | 28 | 0.24 | 88 | | 896 | | | | | | |
| CzCO | 430 | 32/0.21 | 0.33 | 75 | 2.93 | 392.8 | | | | | 3 wt% in mCP | 242 |
| Cz2CO | 440 | 16/0.10 | 0.37 | 84 | 3.28 | 431.8 | | | | | | |
| DOBDiKTa | 460 | 37 | 0.17 | 75 | 2.6 | 43 | 15.3 | | 31.5 | 8.39 | 1.5 wt% in mCP | 243 |
| | 461 | 38 | | 88 | 2.6 | 60 | 6.56 | | 32 | 8.39 | 1.5 wt% in mCP/PPT (1: 1) | |
| 2,3-CZ | 449 | 36 | 0.26 | 39.5 | 4.5 | 435.9 | | | | | $10^{-5}$ M toluene | 244 |
| 2,5-CZ | 459 | 41 | 0.29 | 81.1 | 6.1 | 619.0 | | | | | | |
| 2,6-CZ | 497 | 80 | - | 77.5 | - | 28.1 | | | | | | |
| 2,3-DPA | 496 | 57 | 0.19 | 51.1 | 21.0 | 373.1 | | | | | | |
| 2,3-POA | 547 | 92 | 0.01 | 82.5 | 32.8 | 6.2 | | | | | | |
| SFQ | 460 | 30 | 0.20 | 98.3 | 3.4 | 124 | 8.87 | | 2.04 | 2.63 | 3 wt% in mCBP | 245 |
| SOQ | 457 | 32 | 0.21 | 95.2 | 3.3 | 135 | 1.04 | | 1.94 | 2.06 | | |
| SSQ | 458 | 30 | 0.21 | 92 | 3.0 | 114 | 1.00 | | 2.25 | 2.69 | | |
| SSeQ | 456 | 34 | 0.22 | 96.2 | 2.5 | 131 | 0.65 | | 3.33 | 4.54 | | |
| Sym-DiDiKTa | 542 | 35 | 0.24 | 64 | 1.7 | 4600 | 2.7 | | 4.1 | 0.7 | 1 wt% in mCP | 246 |
| Asym-DiDiKTa | 547 | 35 | 0.23 | 57 | 8.3 | 3000 | 3.1 | | 2.7 | 0.56 | | |
| [5]he-BQAO | 477 | 40 | 0.46 | 78 | 2.3 | - | - | | | | 3 wt% in DCz-BTP | 247 |
| [6]he-BQAO | 494 | 44 | 0.42 | 82 | 2.8 | - | - | | | | | |
| hp-BQAO | 473 | 42 | 0.27 | 87 | 3.6 | 13.4 | 6.8 | | | 25 | | |
| CzP2PO | 379 | 34 | 0.06 | 35 | 2.20 | 5.44 | 8.23 | | | 6.00 | 10 wt% in mCP | 248 |
| tBCzP2PO | 385 | 32 | 0.04 | 62 | 2.20 | 5.47 | 14.3 | | | 10.9 | | |
| DMQAO | 471 | 29 | 0.21 | 84 | 5.1 | 145 | 19.6 | | 14.9 | 1.79 | 5 wt% DMQAO, | 249 |

## Table S2. Summary of reported carbonyl / amino-type MR-TADF emitters.

| | | | | | | | | | | | | |
|---|---|---|---|---|---|---|---|---|---|---|---|---|
| MTDMQAO | 470 | 37 | 0.20 | 99 | 3.7 | 32 | 27.0 | | 23.2 | 19.3 | 10 wt% MTDMQAO, 10 wt% MBDMQAO in mCP | |
| MBDMQAO | 480 | 28 | 0.24 | 94 | 4.3 | 540 | 23.3 | | 19.3 | 1.06 | | |
| SPACtCzBN | 481 | 20 | 0.13 | 82.8 | 5.1 | 44.6 | 3.8 | | 14.9 | 9 | 10 wt% in PhCzBCz | 250 |
| SPBAC-tCzBN | 480 | 20 | 0.13 | 88.8 | 4.4 | 35.9 | 4.6 | | 17.2 | 12 | | |
| o-SPAC-tCzBN | 494 | 21 | 0.11 | 85.6 | 6.5 | 40.7 | 2.8 | | 12.2 | 11 | | |
| IDID2BN | 524 | 25 | 0.16 | 96 | 6.4 | 89.9 | 14 | | | 1.2 | $10^{-5}$ M toluene | 251 |
| DiKTa-LC | 512 | 50 | 0.20 | 52 | 7 | 155.5 | | | | | 10 wt% in PMMA | 252 |
| S-DAO | 493 | 24 | 0.22 | 96 | 5.2 | 140 | 16 | | | 0.8 | $10^{-5}$ M toluene | 253 |
| SS-DAO | 512 | 21 | 0.18 | 98 | 7.3 | 91 | 10 | | | 1.5 | | |
| 3TPA-DiKTa | 551 | 58 | 0.13 | 93 | 14 | 131 | 1.98 | | | 2.49 | 2 wt% in mCP | 254 |
| 3DPA-DiKTa | 617 | 56 | 0.20 | 60 | 16 | 323 | 3.06 | | | 0.14 | | |
| PCP-DiKTa | 469 | 44 | 0.16 | 93 | 6.4 | 179 | 2.66 | | 13 | 3.01 | 2 wt% in 26DCzPPy | 255 |
| Czp-DiKTa | 505 | 66 | 0.13 | 99 | 11.7 | 140 | 1.37 | | 7.2 | 4.41 | | |
| TPQAO | 459 | 23 | 0.24 | 96.7 | 5.9 | 253.7 | 1.6 | | 15.3 | 4.1 | 2 wt% in mCBP | 256 |
| TTPQAO | 476 | 30 | 0.27 | 98.5 | 14.8 | 249.4 | 2.3 | | 4.4 | 1.1 | | |
| DDiKTa-A | 562 | 75 | 0.16 | 92 | 15.1 | 279 | | | | 1.91 | 2 wt% in mCP | 257 |
| SpiroS-QAO | 494 | 43/0.212 | 0.21 | 94 | 9.07 | 196.12 | 3.1 | | | 2.4 | 5 wt% in PhCzBCz | 258 |
| SpiroSO2-QAO | 458 | 32/0.186 | 0.21 | 88 | 9.17 | 216.29 | 2.8 | | | 1.4 | 1 wt% in PPF | |
| SpiroO-QAO | 484 | 34/0.177 | 0.23 | 88 | 9.44 | 584.97 | 2.7 | | | 0.61 | 5 wt% in PhCzBCz | |
| SpiroOSO2-QAO | 485 | 33/0.172 | 0.23 | 89 | 8.65 | 409.13 | 4.7 | | | 0.49 | 1 wt% in PhCzBC | |
| DPQAO-M | 472 | 32 | 0.22 | 77.7 | 4.16 | 163.64 | 24 | | 5.64 | 2.6 | 2 wt% in mCP | 259 |
| DPQAO-F | 472 | 24 | 0.21 | 84.2 | 3.37 | 125.44 | 29.7 | | 5.66 | 4.18 | | |
| 2AcPh | 468 | 21 | 0.30 | 83.1 | 4.5 | 2600 | 1.5 | | | 0.47 | 1 wt% in mCBP | 260 |
| TSFQ-TRZ | 468 | 19 | 0.23 | 91.2 | 7.9 | 385 | 0.945 | | 11.7 | 3.13 | 3 wt% in mCBP | 261 |
| TSFQ-Ph | 473 | 25 | 0.23 | 72.8 | 18.5 | 485 | 0.487 | | 4.9 | 1.61 | | |
| mTIAO | 439 | 17/0.11 | 0.28 | 92 | 3.4 | 179.2 | 10.6 | | 18.8 | 1.36 | 3 wt% in PPF | 262 |
| pTIAO | 465 | 18/0.10 | 0.22 | 90 | 5.8 | 190.7 | 4.5 | | 12.7 | 1.69 | | |
| pTIQA | 520 | 24/0.11 | 0.31 | 86 | 13.7 | - | 6.3 | | - | - | | |

# Table S3. Summary of reported indolocarbazole -type MR-TADF emitters.

| Emitter | $\lambda_{em}$ (nm) | FWHM (nm) | $\Delta E_{ST}$ (eV) | $\Phi_{PL}$ (%) | $\tau_p$ (ns) | $\tau_d$ (μs) | $k_r$ ($10^7$ $s^{-1}$) | $k_{IC}$ ($10^6$ $s^{-1}$) | $k_{ISC}$ ($10^7$ $s^{-1}$) | $k_{RISC}$ ($10^4$ $s^{-1}$) | test status | Ref. |
|---|---|---|---|---|---|---|---|---|---|---|---|---|
| ICzCz | 400 | | 0.38 | 40 | | - | | | | | 1 wt%-PS | 263 |
| ICzAc | 441 | | 0.20 | 32 | | 9.86 | | | | | | |
| ICzDAc | 446 | | 0.17 | 49 | | 8.46 | | | | | | |
| IDCz | 431 | - | - | 37 | 21.2 | - | | | | | 3 wt% in ADN film | 264 |
| IDCz-DPA | 450 | - | - | 48 | 10.4 | - | | | | | | |
| IDCz-2DPA | 460 | - | - | 56 | 12.2 | - | | | | | | |
| pICZ | 441 | 18 | | 88 | 8.37 | | 11 | | | | 1×$10^{-5}$ M in toluene | 265 |
| pICZ-TPA | 447 | 21 | | 90 | 6.70 | | 13 | | | | | |
| ICz | 374 | 21 | 0.47 | 58 | 15 | | | | | | 1×$10^{-5}$ M in toluene | 266 |
| lCzMes$_4$ | 387 | 21 | 0.39 | 66 | 22 | | | | | | | |
| DiICzMes$_4$ | 441 | 17 | 0.26 | 70 | 41 | - | - | - | - | - | | |
| | 457 | 22 | 0.26 | 82 | 14 | 433 | 7.4 | - | 1.4 | 0.018 | 3 wt% in mCP | |
| α-NAICZ | 598 | 28 | 0.38 | 97 | 8.9 | - | 10.9 | - | - | - | 1×$10^{-5}$M DCM | 267 |
| α-EtNAICZ | 620 | 31 | 0.31 | 90 | 8.6 | - | 10.5 | - | - | - | | |
| BisICz | 436 | 27 | 0.31 | 81 | 18.6 | - | 5.38 | | - | - | 1 wt% in mCP: TSPO1 film | 268 |
| tBisICz | 442 | 21 | 0.29 | 95 | 16.0 | 12.5 | 6.25 | | 3.25 | 0.015 | | |
| tPBisICz | 450 | 21 | 0.27 | 91 | 10.9 | 1.74 | 9.17 | | 5.96 | 0.141 | | |
| pSFIAc1 | 443 | 18/0.12 | 0.29 | 84 | 7.7 | - | 10.9 | - | - | - | 1×$10^{-5}$ M toluene | 269 |
| pSFIAc2 | 450 | 18/0.12 | 0.31 | 88 | 8 | - | 11 | - | - | - | | |
| t3IDCz | 470 | 27 | 0.21 | 92 | 12.9 | 377 | | | 5.58 | 0.84 | 1 wt% emitter in mCP: mCBP-1CN film | 270 |
| p3IDCz | 470 | 25 | 0.19 | 100 | 11.7 | 333 | | | 6.15 | 1.07 | | |
| CNICCz | 449 | 56 | 0.27 | 46 | 8.51 | 6.46 | | | | | 10 wt% in DPEPO | 271 |
| CNICtCz | 456 | 60 | 0.19 | 50 | 81.8 | 6.25 | | | | | | |
| tDIDCz | 393 | 20 | 0.44 | 60 | 11.4 | | | | | | 1×$10^{-5}$M in THF | 272 |
| m-FLDID | 404 | 22 | 0.36 | 71 | 15.5 | | | | | | 1 wt % in m-CP/TSPO1 | 273 |
| Cz-DICz | 457 | 14 | 0.34 | 98.6 | 5.5 | 405 | 18 | | | 0.26 | 1×$10^{-5}$ M toluene | 274 |
| tBisICz-DPA | 447 | 28 | 0.28 | 96 | 10.7 | 3390 | 9.35 | | 5.14 | 0.061 | 3 wt % in mCP: TSPO1 film | 275 |
| tBisICz-PhCz | 443 | 19 | 0.31 | 91 | 11.5 | 6270 | 8.70 | | 4.78 | 0.030 | | |
| Nm-ICz | 406 | 28 | 0.35 | 54.7 | 3.0 | | 18.2 | | | | 3 wt.% in mCBP | 276 |

## Table S3. Summary of reported indolocarbazole -type MR-TADF emitters.

| | | | | | | | | | | | | |
|---|---|---|---|---|---|---|---|---|---|---|---|---|
| CNm-ICz | 421 | 33 | 0.32 | 56.5 | 3.2 | | 17.7 | | | | 3 wt.% in PPF | |
| NB-1 | 447 | 11 | 0.5 | 81 | 5.8 | | 17 | | | | $1\times10^{-5}$ M toluene | 277 |
| NB-2 | 454 | 11 | | 75 | 6.2 | | 15 | | | | | |
| IDCz-DBS | 456 | 12/0.72 | 0.37 | 92 | 5.6 | 15.0 | 18 | | | 12 | $1\times10^{-5}$M toluene | 278 |
| DIDCz-tBu | 468 | 20 | 0.34 | 96.5 | 11.8 | | 8.2 | | | | 1 wt.% in BH | 279 |
| NCON-TB | 521 | 28/0.13 | 0.35 | 82 | 15.2 | | | | | | $1\times10^{-5}$ M toluene | 280 |
| NCON-Mes | 520 | 18/0.13 | 0.31 | 94 | 14.4 | | | | | | | |

**Table S4**. Performance of B/N type MR-emitters in optimized OLEDs

| Emitter | $\lambda_{EL}$ [nm] | FWHM [nm] | CIE (x,y) | $L_{max}$ [cd $m^{-2}$] | CE [cd $A^{-1}$] [a)] | PE [lm $W^{-2}$] [a)] | EQE [lm $W^{-2}$] [a)] | Efficiency Roll-off [%][b)] | Ref. |
|---|---|---|---|---|---|---|---|---|---|
| DABNA-1[1)] | 459 | 28 | 0.13,0.09 | ＜1000 | 10.6/-/- | 8.3/-/- | 13.5/6.3/- | 53 | 1 |
| DABNA-2[1)] | 467 | 28 | 0.12,0.13 | ＜1000 | 21.1/14.2/- | 15.1/7.9/- | 20.2/13.4/- | 34 | |
| B2[2)] | 460 | 37 | 0.13,0.11 | ＜1000 | 16.7/11.5/- | 13.8/7.1/- | 18.3/12.6/- | 31 | 3 |
| TBN-TPA[d)] | 474 | 27 | 0.13,0.19 | 16593 | 40.2/34.4/17.4 | 30.0/23.5/8.8 | 32.1/27.4/13.9 | 15 | 4 |
| t-DABNA[4)] | 466 | 31 | 0.13,0.15 | - | 32.6/28.9/20.9 | 33.6/21.0/10.9 | 31.4/27.2/19.8 | 13 | 6 |
| ν-DABNA[5)] | 469 | 18 | 0.12,0.11 | - | 31.0/29.5/23.2 | 25.6/20.8/23.2 | 34.4/32.8/26.0 | 5 | 8 |
| ADBNA-Me-MeS[6)] | 481 | 32 | 0.10,0.27 | ＜1000 | 25.5/16.6/- | 19.3/10.0/- | 16.2/11.1/- | 31 | 5 |
| ADBNA-Me-Tip[6)] | 480 | 33 | 0.11,0.29 | ＜1000 | 34.7/23.5/- | 28.7/14.9/- | 21.4/15.4/- | 28 | |
| TCz-BN[7)] | 474 | 34 | 0.13,0.20 | - | - | 24.0/17.0/10.0 | 18.9/15.2/10.5 | 20 | 7 |
| 2F-BN[7)] | 501 | 40 | 0.16,0.60 | - | - | 69.8/60.1/38.1 | 22.0/20.1/15.0 | 9 | |
| 3F-BN[7)] | 499 | 39 | 0.20,0.58 | - | - | 72.3/63.1/45.9 | 22.7/22.3/21.1 | 2 | |
| 4F-BN[7)] | 493 | 32 | 0.12,0.48 | - | - | 51.3/42.4/29.4 | 20.9/19.2/16.4 | 8 | |
| AZA-BN[8)] | 527 | 30 | 0.27,0.69 | > 2000 | - | 121.7/-/- | 28.2/26.5/19.1 | 6 | 281 |
| Cz-BN[9)] | 480 | 35 | - | > 1000 | 22.3/-/- | 14.0/-/- | 14.7/-/- | - | 23 |
| BCz-BN[9)] | 490 | 32 | - | > 1000 | 31.1/-/- | 19.5/-/- | 16.3/-/- | - | |
| m-Cz-BNCz[10)] | 528 | 45 | 0.26,0.68 | 26230 | 117.6/-/- | 127.4/-/- | 31.4/29.0/17.5 | 7.6 | 11 |
| DtBuPhCzB[11)] | 512 | 52 | 0.25,0.65 | 19950 | 90.3/-/- | 100.7/-/- | 26.5/14.3/7.3 | 46 | 12 |
| DtBuPhCzB[12)] | 504 | 34 | 0.15,0.61 | 13670 | 66.2/-/- | 54.7/-/- | 23.4/14.6/5.7 | 37 | |
| DtBuCzB[12)] | 488 | 29 | 0.10,0.42 | 6934 | 43.2/-/- | 37.7/-/- | 21.6/15.0/5.3 | 30.5 | |
| OAB-ABP-1[d13)] | 505 | 33 | 0.12,0.63 | > 1000 | 53.2/53.3/- | 43.5/34.9/- | 21.8/19.6/- | 10 | 9 |
| BN-DMAC[14)] | 502 | 48 | 0.14,0.54 | > 1000 | 55.2/-/- | 43.4/-/- | 21.1/19.7/12.5 | 6.6 | 282 |
| BN-DPAC[14)] | 504 | 48 | 0.14,0.56 | > 1000 | 77.3 | 56.5 | 28.2/27.7/19.2 | 1.8 | |
| BBCz-DB[15)] | 469 | 27 | 0.12,0.19 | - | - | - | 29.3/-/- | - | 13 |
| BCz-BN[15)] | 487 | 26 | | - | - | - | 27.8/-/- | - | |
| BBCz-G[15)] | 515 | 54 | 0.26,0.68 | - | - | - | 31.8/-/- | - | |
| BBCz-Y[15)] | 549 | 48 | | - | - | - | 29.3/-/- | - | |
| BBCz-R[15)] | 616 | 26 | 0.67,0.33 | - | - | - | 22.0/-/- | - | |
| R-BN[16)] | 664 | 48 | 0.719,0.280 | - | - | - | 28.4/-/- | - | 283 |
| R-TBN[16)] | 686 | 49 | 0.721,0.278 | - | - | - | 28.1/-/- | - | |
| m-ν-DABNA[17)] | 471 | 18 | 0.12,0.12 | | 32.1 | | 36.2 | | 44 |
| 4F-ν-DABNA[d17)] | 464 | 18 | 0.13,0.08 | | 26.8 | | 35.8 | | |
| 4F-m-ν-DABNA[17)] | 461 | 18 | 0.13,0.06 | | 24.9 | | 33.7 | | |
| ν-DABNA-Mes[18)] | 480 | 27 | 0.09, 0.21 | ＞1000 | 26.7/24.2/12.9 | 20.0/12.6/4.5 | 22.9/20.3/10.9 | 11 | 42 |
| ν-DABNA-O-Me[19)] | 465 | 23 | 0.13,0.10 | ＞ 10000 | 22.7/17.7/13.5 | 24.6/23.8/22.1 | 29.5/28.8/26.9 | 2.4 | 24 |
| DABNA-NP-TB[20)] | 457 | 33 | 0.14,0.11 | | | | 19.5/17.5/12.0 | 10.2 | 18 |
| Cz2DABNA-NP-M/TB[20)] | 477 | 27 | 0.11,0.23 | | | | 21.8/20.4/14.9 | 6.4 | |
| CzB2-M/P[20)] | 497 | 29 | 0.12,0.57 | | | | 26.7/24.4/18.0 | 8.6 | |
| S-Cz-BN[21)] | 488 | 26 | 0.12,0.43 | | | | 30.5/30.2/26.2 | 0.98 | 33 |
| D-Cz-BN[21)] | 488 | 24 | 0.11,0.43 | | | | 37.2/37.2/34.3 | 0 | |
| BN-CP1[22)] | 496 | 25 | 0.09,0.50 | 54690 | 83.8 | 109.7 | 40.0/34.0/18.5 | 15 | 34 |
| BN-CP2[22)] | 497 | 26 | 0.10,0.53 | 57582 | 82.6 | 108.1 | 36.4/32.6/19.2 | 10.4 | |
| BN1[23)] | 506 | 36 | 0.15,0.63 | 6636 | 72.8 | 65.3 | 24.3/18.4/12.9 | 24.3 | 25 |
| BN2[23)] | 545 | 46 | 0.38,0.61 | 6286 | 101.6 | 83.1 | 24.5/15.8/7.6 | 35.5 | |
| BN3[23)] | 568 | 43 | 0.47,0.52 | 5004 | 92.6 | 106.4 | 24.7/17.6/8.9 | 27.9 | |
| TW-BN[24)] | 488 | 26 | 0.14,0.36 | 9726 | 45.3, | 33.1 | 27.8/25.4/10.7 | 8.6 | 39 |
| TPh-BN[24)] | 492 | 28 | 0.10,0.46 | 13518 | 54.8 | 43.1 | 28.9/25.1/15.6 | 13.1 | |
| pCz-BN[24)] | 496 | 30 | 0.13,0.54 | 11483 | 64.6 | 50.8 | 27.2/25.6/12.2 | 5.9 | |
| mCz-BN[24)] | 496 | 31 | 0.15,0.55 | 13725 | 61.2 | 44.7 | 25.9/24.1/14.0 | 6.9 | |
| γ–Cb-B[25)] | 461 | 28 | 0.13,0.13 | | 17.8. | 19.0 | 19.0/16.2/7.7 | 14.7 | 30 |
| Cz-B[25)] | 482 | 30 | 0.11,0.31 | | 34.7 | 24.7 | 22.6/16.6/6.9 | 26.5 | |
| TCz-B[25)] | 515 | 30 | 0.16,0.71 | | 100.7 | 72.4 | 29.2/24.7/9.4 | 15.4 | |
| DACz-B[d25)] | 571 | 44 | 0.47,0.51 | | 81.6 | 58.2 | 19.6/12.0/4.8 | 38.8 | |
| BN-DMAC[26)] | 502 | 56 | 0.19,0.59 | | 76.6 | 68.8 | 25.5/21.5/16.0 | 15.7 | 16 |
| BN-DPAC[26)] | 504 | 49 | 0.16,0.61 | | 90.5 | 81.2 | 30.2/27.6/22.1 | 8.6 | |
| B-O-dpa[27)] | 443 | 32 | 0.15,0.05 | 182 | 8.3 | | 16.3/2.2/- | 86.5 | 32 |
| B-O-Cz[27)] | 481 | 63 | 0.13,0.22 | 665 | 20.3 | | 13.4/5.9/- | 55.9 | |
| B-O-dmAc[27)] | 475 | 44 | 0.12,0.21 | 653 | 23.1 | | 16.2/8.4/- | 48.1 | |
| B-O-dpAc[27)] | 473 | 42 | 0.12,0.20 | 593 | 22.8 | | 17.0/9.6/- | 43.5 | |
| CzBNO[28)] | 454 | 36 | 0.14,0.08 | | 14.7/10.3/4.0 | | 13.6/11.0/5.0 | 19.1 | 38 |
| DMAcBNO[28)] | 472 | 41 | 0.13,0.19 | | 31.2/25.6/11.6 | | 20.4/18.1/8.6 | 11.2 | |
| DPAcBNO[28)] | 468 | 37 | 0.13,0.14 | | 28.0/25.3/9.8 | | 23.0/22.3/9.1 | 3.0 | |
| CzBNO[29)] | 481 | 36 | - | - | - | - | 25.9 | - | |
| DMAcBNO[29)] | 470 | - | - | - | - | - | - | - | |
| DPAcBNO[d29)] | 470 | - | - | - | 53.7/50.9/40.4 | | 29.6/28.5/23.1 | 3.7 | |
| DPACBN1[30)] | 475 | 34 | 0.14,0.30 | 16234 | 36.0 | | 23.6/19.3/9.6 | 18.2 | 40 |
| DPACBN2[30)] | 469 | 28 | 0.13,0.16 | 15495 | 31.6 | | 24.0/23.5/14.3 | 2.1 | |
| DPACBN3[30)] | 472 | 24 | 0.12,0.18 | 14629 | 34.5 | | 27.7/19.3/6.7 | 30.3 | |
| BO3N[31)] | 466 | 66 | 0.14,0.24 | 3060 | 19.45 | 20.37 | 7.02/-/- | - | 284 |
| TRZ3DPA[31)] | 478 | 61 | 0.14,0.17 | 4669 | 12.60 | 13.20 | 14.37/-/- | - | |
| 2PXZBN[32)] | 522 | 60 | 0.28,0.64 | 11910 | 64.9 | 58.3 | 17.7/15.3/7.4 | 13.6 | 19 |
| 2PTZBN[32)] | 528 | 58 | 0.28,0.65 | 16970 | 96.5 | 86.6 | 25.5/21.7/17.2 | 14.9 | |
| BSBS-N1[33)] | 478 | 25 | 0.11,0.22 | - | - | - | 21.0/-/- | - | 29 |
| BOBO-Z[34)] | 445 | 18 | 0.15,0.04 | - | 7.2 | 5.0 | 13.6/9.8/3.3 | 27.9 | 43 |
| BOBS-Z[34)] | 456 | 23 | 0.14,0.06 | - | 16.7 | 12.9 | 26.9/24.0/15.0 | 10.8 | |
| BSBS-Z[34)] | 463 | 22 | 0.13,0.08 | - | 23.2 | 15.0 | 26.8/24.0/15.9 | 10.4 | |
| BN-ICz-1[35)] | 523 | 23 | 0.22,0.73 | - | 84.2/37.7.21.6 | | 30.5/17.2/12.8 | 43.6 | 58 |
| BN-ICz-2[35)] | 523 | 23 | 0.23,0.73 | - | 102.9/74.5/51.8 | | 29.8/26.1/22.0 | 12.4 | |
| BN-ICz-1[36)] | 525 | 19 | 0.17,0.78 | - | - | 220/183,155 | - | - | |
| BP-2DPA[37)] | 605 | 42 | 0.62,0.38 | ＞3000 | - | 14.0 | 11.3/8.7/5.3 | 23.0 | 41 |
| DBP-4DPA[37)] | 617 | 44 | 0.63,0.36 | ＞3000 | - | 13.8 | 15.1/11.2/7.0 | 25.8 | |
| tCBNDADPO[38)] | 472 | 28 | 0.14,0.22 | 7082 | 47.1/35.4,25.0 | 45.5/22.3/10.4 | 30.8/23.3/16.2 | 24.3 | 52 |
| DBNO[39)] | 504 | 24 | 0.18,0.60 | 7009 | 94.1 | 98.5 | 35.9/9.0/5.9 | 74.9 | 55 |
| DBNO[40)] | 504 | 27 | 0.14,0.53 | 22340 | 100.1 | 105.6 | 37.1/30.8/20.6 | 16.9 | |
| t-DABNA-dtB[41)] | 473 | 23 | 0.11,0.16 | | 28.1 | 26.0 | 25.4 | severe | 56 |
| t-DABNA-dtB[42)] | 471 | | 0.117,0.134 | | 11.4 | 9.7 | 11.4/-/10.9 | - | |
| t-DABNA-dtB[43)] | 470 | | 0.117,0.112 | | 25.6 | 6.8 | 30.1/-/28.8 | - | |
| SF1BN[44)] | 492 | 27.5 | 0.077,0.47 | | 70.0/53.0/27.2 | 56.7/38.8//15.7 | 35.9/26.9/14.0 | 25.1 | 54 |
| SF3BN[44)] | 496 | 29.9 | 0.092,0.515 | | 70.0/51.9/22.4 | 54.9/33.3/10.8 | 32.2/23.9/10.3 | 25.8 | |
| SF1BN[45)] | 492 | 28.4 | 0.088,0.432 | | 700/59.8/42.5 | 62.6/47.2/28.2 | 36.7/30.8/22. | 16.1 | |

## Table S4. Performance of B/N type MR-emitters in optimized OLEDs

| | | | | | | | | | |
|---|---|---|---|---|---|---|---|---|---|
| SF3BN [45] | 492 | 29.2 | 0.092,0.416 | | 65.0/57.0/42.6 | 55.0/42.9/25.9 | 34.6/30.3/22.7 | 12.4 | |
| Cz-PTZ-BN [46] | 520 | 53 | 0.26,0.65 | 29793 | 100.4 | 86.1 | 27.6/26.2/17.3 | 5.1 | 48 |
| 2Cz-PTZ-BN [46] | 516 | 56 | 0.24,0.63 | 26887 | 108.5 | 92.1 | 32.8/30.8/23.5 | 6.1 | |
| (*P*)-helicene-BN [47] g=+1.2 | 523 | 49 | 0.26,0.66 | 71483 | 117.5 | 153.8 | 31.5/29.6/18.7 | 6.0 | 57 |
| (*M*-helicene-BN [47] g=-2.2 | 524 | 50 | 0.26,0.66 | 77168 | 117.8 | 154.2 | 30.7/28.3/17.9 | 7.8 | |
| TPD4PA [48] | 455 | 29 | 0.14,0.06 | - | 15.7/-/9.2 | - | 30.7 / 30.6 / 17.8 | 0.33 | 70 |
| tBu-TPD4PA [48] | 460 | 29 | 0.14,0.07 | - | 19.5/-/12.9 | - | 32.5 / 30.9 / 20.5 | 49.3 | |
| (R)-OBN-2CN-BN [49] | 496 | 30 | 0.11,0.52 | 6617 | 67.3 | 70.5 | 29.4/19.8/8.1 | 32.6 | 21 |
| (R)-OBN-4CN-BN [49] | 508 | 33 | 0.14,0.64 | 2982 | 70.7 | 74.1 | 24.5/8.0/2.1 | 67.3 | |
| (R)-OBN-2CN-BN [50] | 496 | 33 | 0.13,0.53 | 27200 | 71.9 | 79.8 | 29.8/27.2/21.2 | 4.7 | |
| (R)-OBN-4CN-BN [50] | 508 | 34 | 0.16,0.66 | 9052 | 76.1 | 81.1 | 24.7/23.5/13.1 | 4.9 | |
| DMAC-BN [51] | 503 | 49 | 0.18, 0.60 | - | 57.0/49.7/34.2 | 49.7/26.6/20.1 | 20.3/17.6/12.0 | 13.3 | 26 |
| PXZ-BN [51] | 516 | 47 | 0.22,0.67 | - | 83.8/71.4/40.9 | 70.1/50.0/22.1 | 23.3/19.9/11.3 | 14.6 | |
| CNCz-BNCz [52] | 584 | 49 | 0.55,0.45 | | | 65.4/24.8/9.6 | 23.0/10.8/5.4 | 53.1 | 27 |
| CNCz-BNCz [53] | 584 | 50 | 0.53,0.45 | | | 67.4/38.3/11.7 | 24.7/17.7/7.8 | 28.3 | |
| CNCz-BNCz [54] | 583 | 49 | 0.54,0.46 | | | 117.8/69.1/29.6 | 33.7/27.7/16.4 | 17.8 | |
| PAB [55] | 456 | 31 | 0.145,0.076 | 782 | 10.4 | 7.4 | 14.7 | | 28 |
| 2tPAB [55] | 456 | 27 | 0.145,0.076 | 1241 | 11.8 | 8.0 | 16.8 | | |
| 3tPAB [55] | 460 | 26 | 0.141,0.076 | 1100 | 13.4 | 10.2 | 19.3 | | |
| y-Cb-B [56] | 461 | 28 | 0.13,0.13 | | 17.8 | 19.0 | 19.0/16.2/7.7 | 14.7 | 30 |
| Cz-B [56] | 482 | 30 | 0.11,0.31 | | 34.7 | 24.7 | 22.6/16.6/6.9 | 26.5 | |
| TCz-B [56] | 515 | 30 | 0.16,0.71 | | 100.7 | 72.4 | 29.2/24.7/9.4 | 15.4 | |
| DACz-B [56] | 571 | 44 | 0.47,0.51 | | 81.6 | 58.2 | 19.6/12.0/4.8 | 38.8 | |
| DtCzB-DPTRZ [57] | 532 | 39 | 0.33,0.63 | 16180 | 88.6 | 92.7 | 24.6/7.5/4.7 | 69.5 | 31 |
| DtCzB-TPTRZ [57] | 516 | 38 | 0.18,0.67 | 16550 | 93.2 | 98.8 | 29.8/26.4/12.4 | 11.4 | |
| DtCzB-PPm [57] | 508 | 33 | 0.16,0.66 | 13280 | 87.5 | 92.1 | 28.6/24.3/10.2 | 15.0 | |
| DtCzB-CNPm [57] | 540 | 44 | 0.35,0.63 | 13390 | 99.1 | 107.4 | 25.0/14.4/5.7 | 42.4 | |
| DtCzB-DPTRZ [58] | 528 | 36 | 0.27,0.60 | 19210 | 99.1 | 107.3 | 26.0/20.0/15.6 | 23.1 | |
| DtCzB-TPTRZ [58] | 520 | 41 | 0.23,0.68 | 25520 | 105.8 | 116.2 | 30.6/28.6/16.4 | 6.5 | |
| DtCzB-PPm [58] | 508 | 35 | 0.17,0.67 | 24310 | 93.3 | 103.3 | 29.8/29.4/21.7 | 1.3 | |
| DtCzB-CNPm [58] | 540 | 44 | 0.35,0.63 | 22520 | 102.1 | 114.5 | 28.1/23.4/10.7 | 16.7 | |
| (+)-BN4 [59] | 510 | 49/0.23 | 0.186,0.632 | 3334 | 66.3 | 38.58 | 20.6/20.5/10.7 | 0.5 | 35 |
| (-)-BN4 [59] | 512 | 49/0.23 | 0.206,0.635 | 3037 | 55.1 | 35.16 | 19.0/16.6/10.1 | 12.6 | |
| (+)-BN5 [59] | 506 | 48/0.23 | 0.173,0.590 | 2950 | 65.1 | 36.47 | 22.0/15.3/10.9 | 30.4 | |
| (-)-BN5 [59] | 506 | 48/0.23 | 0.167,0.603 | 3062 | 79.4 | 44.46 | 26.5/17.6/11.1 | 33.6 | |
| CzBNCz [60] | 471 | 50 | 0.16,0.305 | 63777 | 42.6 | 47.8 | 21.9/21.2/19.8 | 3.2 | 37 |
| CzBN [60] | 480 | 34 | 0.135,0.312 | 49933 | 38.3 | 40.1 | 20.6/19.4/17.0 | 5.8 | |
| TPXZBN [61] | 506 | 37 | 0.16,0.65 | 22723 | 64.8/60.6/53.2 | 37.0/35.9/24.9 | 21.3/19.6/17.4 | 7.98 | 45 |
| DPXZCZBN [61] | 505 | 36 | 0.15,0.64 | 37129 | 61.6/60.5/55.1 | 43.2/43.2/32.6 | 19.2/18.8/17.2 | 2.1 | |
| Cz-BSN [62] | 482 | 32 | 0.11,0.28 | 4034 | 29.5/21.0/10.6 | | 18.9/13.4/6.8 | 34.4 | 46 |
| DCz-BSN [62] | 473 | 29 | 0.11,0.17 | 4397 | 25.7/20.3/11.7 | | 22.0/18.6/10.0 | 15.4 | |
| BN1 [63] | 507 | - | 0.27,0.49 | 18180 | 26.6/14.8/19.1 [c] | 22.0/14.8/11.9 [c] | 9.9/8.0/7.0 [c] | 19.2 [c] | 47 |
| TCz-BN1 [63] | 507 | - | 0.28,0.48 | 20952 | 31.3/29.8/27.5 [c] | 22.2/18.7/16.0 [c] | 11.5/11.0/10.2 [c] | 4.3 [c] | |
| BN2 [64] | 547 | - | 0.40,0.57 | 21576 | 66.1/55.7/44.9 [c] | 59.0/39.7/28.2 [c] | 19.9/16.7/13.5 [c] | 16.1 [c] | |
| TCz-BN2 [64] | 554 | - | 0.41,0.56 | 30708 | 81.8/70.1/61.2 [c] | 79.7/52.4/41.8 [c] | 25.1/21.4/18.7 [c] | 14.7 [c] | |
| mBP-DABNA-Me [65] | 468 | 28 | 0.124,0.14 | | 24,4/19.8/16.2 | 21.3/10.5/7.6 | 24.3/19.5/16.0 | 19.8 | 50 |
| NBNP [66] | 502 | 33 | 0.12,0.62 | 34825 | 76.6/70.2/51.6 [d] | 45.4/37.4/20.5 [d] | 28.0/25.6/18.8 [d] | 8.6 | 51 |
| NBO [66] | 491 | 29 | 0.09,0.41 | 12396 | 48.3/37.1/17.9 [d] | 20.2/16.2/4.8 [d] | 26.1/20.0/9.7 [d] | 23.4 | |
| BON-D0 [67] | 460 | 39 | 0.14,0.12 | 1746 | 13.2 | 11.8 | 9.7 | - | 53 |
| BON-D1 [67] | 488 | 39 | 0.13,0.44 | 2415 | 29.2 | 22.9 | 13.4 | - | |
| BON-D2 [67] | 487/509 | 61 | 0.15,0.45 | 4387 | 35.7 | 26.7 | 14.9 | - | |
| (R)-BN-MeIAc [68] | 504 | 33 | 0.12, 0.63 | 76429 | 103.0 | 130.2 | 37.2/36.1/26.1 | 2.9 | 59 |
| (S)-BN-MeIAc [68] | 503 | 33 | 0.12, 0.62 | 71664 | 98.5 | 128.0 | 36.1/35.2/25.1 | 2.5 | |
| Asym-BN1 [69] | 457 | 28, 0.152 | 0.14, 0.08 | 8323 | 20.9 | 14.9 | 31.2/18.3/9.3 | 41.3 | 60 |
| Sym-BN2 [69] | 467 | 23, 0.129 | 0.13, 0.11 | 14064 | 29.7 | 20.3 | 33.2/25.5/15.5 | 23.2 | |
| Sym-BN3 [69] | 458 | 23, 0.131 | 0.14, 0.08 | 18438 | 27.5 | 19.0 | 37.6/34.0/26.2 | 9.6 | |
| BN-TP [70] | 528 | 36 | 0.26, 0.70 | 24030 | 139.3 | 139.3 | 35.1/32.4/20.8 | 7.7 | 61 |
| PTZBN1 [71] | 496 | 47 | 0.16, 0.51 | 31431 | 69.9 | 49.9 | 26.9/25.3/17.9 | 5.9 | 62 |
| PTZBN2 [71] | 483 | 43 | 0.13, 0.31 | 32662 | 59.6 | 46.8 | 30.5/29.7/23.0 | 2.6 | |
| PTZBN3 [71] | 476 | 36 | 0.13, 0.22 | 16322 | 30.6 | 24.0 | 19.9/15.8/12.1 | 20.6 | |
| PTZBN1 [72] | 489 | 47 | 0.14, 0.41 | 30798 | 73.3 | 82.2 | 32.7/25.9/16.5 | 20.8 | |
| PTZBN2 [72] | 478 | 48 | 0.15, 0.29 | 37497 | 67.4 | 75.6 | 34.8/28.8/19.5 | 17.2 | |
| PTZBN3 [72] | 468 | 46 | 0.15, 0.24 | 22417 | 55.7 | 62.5 | 32.0/30.3/25.0 | 5.3 | |
| BNO1 [73] | 610 | 39 | 0.64, 0.34 | 135 000 | 59.4 | 66.7 | 35.6/31.1/27.7 | 12.6 | 63 |
| BNO2 [73] | 618 | 39 | 0.65, 0.35 | 126 000 | 46.5 | 52.2 | 34.4/29.8/26.9 | 13.4 | |
| BNO3 [73] | 625 | 40 | 0.66, 0.34 | 125 000 | 43.4 | 48.7 | 36.1/32.1/28.6 | 11.1 | |
| CzBO [74] | 448 | 30/0.18 | 0.15, 0.05 | | 7.4 | 5.7 | 13.4/8.4/3.5 | 37.3 | 64 |
| CzBS [74] | 473 | 31/0.17 | 0.11, 0.16 | | 25.8 | 19.4 | 23.1/21.3/15.0 | 7.8 | |
| CzBSe [74] | 481 | 33/0.18 | 0.10, 0.24 | | 34.8 | 25.8 | 23.9/23.4/20.0 | 2.1 | |
| CzBO [75] | 448 | 30/0.18 | 0.15, 0.05 | | 9.0 | 6.7 | 16.3/10.7/4.7 | 34.4 | |
| CzBS [75] | 474 | 31/0.17 | 0.11, 0.16 | | 33.0 | 23.6 | 29.1/27.4/20.0 | 5.8 | |
| CzBSe [75] | 481 | 33/0.18 | 0.10, 0.24 | | 44.5 | 32.5 | 30.1/29.7/26.1 | 1.3 | |
| tDPAC-BN [76] | 460 | 28 | 0.135, 0.094 | 1126 | 17.6/12.9/5.0 | 15.4/6.5/1.5 | 21.6/15.3/5.4 | 29.6 | 65 |
| tDMAC-BN [76] | 472 | 34 | 0.116, 0.186 | 1536 | 28.1/23.9/13.2 | 23.7/12.9/4.6 | 22.3/19.0/10.4 | 14.8 | |
| SBON [77] | 466 | 28 | 0.13, 0.13 | 5627 | 16.1/15.0/8.0 | 11.4/8.1/3.0 | 13.7/12.6/6.7 | 8.0 | 66 |
| SBSN [77] | 492 | 31 | 0.10, 0.44 | 22817 | 40.4/40.4/27.5 | 25.4/25.4/13.9 | 17.6/17.6/12.0 | 0 | |
| DBON [77] | 510 | 29 | 0.17, 0.68 | 11719 | 94.1/70.9/42.0 | 75.8/48.4/22.8 | 26.7/20.2/12.0 | 24.3 | |
| DBSN [77] | 556 | 43 | 0.42, 0.57 | 15484 | 84.7/80.0/65.8 | 71.9/55.8/37.6 | 21.8/20.6/16.9 | 5.5 | |
| m[B-N]N1 [78] | 479 | 27/0.14 | 0.115, 0.272 | 21760 | 58.6/51.5/43.6 [d] | 61.9/25.3/14.9 [d] | 36.0/ 32.3/ 27.6 [d] | 10.3 | 67 |
| m[B-N]N2 [78] | 485 | 33/0.18 | 0.112, 0.319 | 26480 | 48.4/40.9/34.9 [d] | 53.5/21.6/11.8 [d] | 33.4/29.7/24.7 [d] | 11.1 | |
| BIC-mCz [79] | 432 | 42 | 0.16, 0.05 | - | - | 9.7 | 19.4 | - | 68 |
| BIC-pCz [79] | 466 | 48 | 0.14, 0.16 | - | - | 51.1 | 39.8 | - | |
| mDBIC [79] | 431 | 42 | 0.16, 0.05 | - | - | 9.7 | 13.5 | - | |
| pDBIC [79] | 535 | 30 | 0.33, 0.64 | - | - | 87 | 31.0 | - | |
| ν-DABNA-CN-Me [80] | 504 | 23 | 0.13,0.65 | > 10000 | 89.0/87.1/78.2 | 137.6/135.3/122.3 | 32.0/31.5/28.5 | 1.6 | 69 |
| BCzBN [81] | 490 | 32 | | | 31,1 | 19.5 | 16.3 | - | 23 |
| CzBN [81] | 480 | 35 | | | 22.3 | 14.0 | 14.7 | | |
| BN3 [82] | 566 | 46 | | | 99.6 | 104.3 | 26.5 | | 285 |
| BN3 [83] | 568 | 43 | | | 67.8 | 63.2 | 18.1 | | |

## **Table S4**. Performance of B/N type MR-emitters in optimized OLEDs

| | | | | | | | | | |
|---|---|---|---|---|---|---|---|---|---|
| BN1[84)] | 510 | 42 | | | 103.7, 62.7, 42.0 | 100.3, 46.3, 22.9 | 33.2/ 20.1/13.4 | 39.5 | 286 |
| BN1[85] | 510 | 42 | | | 102.8, 61.8, 41.6 | 99.3, 43.1, 20.1 | 32.9/19.8/13.3 | 39.8 | |
| DABNA-NP-TB[86)] | 458 | | 0.136,0.076 | | 4.28/-/4.27 | | 7.03/-7.00 | | 287 |
| DABNA-NP-TB[87)] | 458 | | 0.136,0.076 | | 3.64/-/3.63 | | 6.05/-/6.00 | | |
| (*R*)-DOBN[88)] | 459 | 38 | 0.14, 0.10 | 5140 | 21.4/11.3/7.7 | 18.8/-/- | 23.9/12.5/8.6 | 47.7 | 288 |
| (*R*)-DOBNT[88)] | 464 | 35 | 0.13, 0.12 | 6459 | 24.1/12.2/8.1 | 17.2/-/- | 25.6/13.9/8.6 | 45.7 | |
| 2PXZBN[89)] | 517 | 49 | 0.23, 0.67 | 120321 | 108.8/-/75.1 | 123.4/-/55.8 | 30.7/-/24.0 | | 20 |
| 2PTABN[89)] | 520 | 51 | 0.24,0.67 | 128576 | 124.9/-/94.4 | 157.7/-/79.1 | 34.6/-/29.5 | | |
| BNSSe[89)] | 515 | 50 | 0.22,0.66 | 113881 | 124.2/-/110.8 | 156.2/-/90.2 | 35.7/-/32.0 | | |
| BNSeSe[89)] | 512 | 48 | 0.19,0.66 | 108188 | 121.0/-/111.0 | 146.3/-/90.1 | 36.8/-/34.0 | | |
| BN3[90)] | 558 | 40 | 0.41,0.58 | 191023 | 164.5/-/131.3 | 205.8/-/112.2 | 40.5/-/32.4 | | |
| DtCzB-DPTRZ[90)] | 527 | 31 | 0.29,0.68 | 149376 | 161.6/-/139.9 | 181.3/-/114.8 | 39.6/-/34.5 | | |
| 2F-BN[91)] | 495 | 28 | | | | 76.165.3/43.9 | 33.1/31.2/27.0 | 5.7 | 289 |
| 2F-BN[92)] | 497 | 30 | | | | 62.4/50.3/22.7 | 25.6/22.6/14.2 | 11.7 | |
| 2F-BN[93)] | 495 | 28 | | | | 46.4/43.6/29.8 | 23.2/20.5/18.8 | 11.6 | |
| tCzphB-Ph[94)] | 527 | 24 | 0.21,0.75 | 32000 | - | - | 29.3 | - | 71 |
| tCzphB-Fl[94)] | 535 | 26 | 0.26,0.72 | 37000 | - | - | 26.2 | - | |
| tCzphB-Ph[95)] | 526 | 27 | 0.21,0.74 | 520000 | - | - | 31.3 | - | |
| tCzphB-Fl[95)] | | | | - | | - | 29.7 | | |
| DtBuCzB[96)] | 490 | 31 | 0.10,0.45 | | | | 26.3/21.1/9.0 | 19.8 | 72 |
| TCzBN-DPF[96)] | 498 | 31 | 0.10,0.56 | | | | 26.4/22.8/12.0 | 13.6 | |
| TCzBN-TMPh[96)] | 488 | 27 | 0.11,0.38 | | | | 25.1/18.4/6.5 | 26.7 | |
| TCzBN-oPh[96)] | 492 | 28 | 0.09,0.46 | | | | 26.0/22.9/10.4 | 11.9 | |
| t-Bu-ν-DABNA[97)] | 474 | 19 | 0.13,0.19 | | 39.4/-/36.0 | | 37.6/-/33.9 | - | 73 |
| t-Bu-ν-DABNA[98)] | 474 | 19 | 0.12,0.15 | | 31.6/-/27.7 | | 40.7/-/35.8 | - | |
| (SIPr)AuBN[99)] | 511 | 40 | 0.20.0.69 | 253000 | 86.1/-/84.4 | | 24.8/-/24.3 | - | 74 |
| (BzIPr)AuBN[99)] | 510 | 34 | 0.16,0.68 | 216000 | 97.1/-/89.9 | | 30.3/-/28.1 | - | |
| (PyIPr)AuBN[99)] | 512 | 37 | 0.18,0.69 | 151000 | 93.5/-/69.3 | | 27.6/-/20.5 | | |
| (PzIPr)AuBN[99)] | 515 | 39 | 0.22,0.67 | 192000 | 82.8/-/79.7 | | 24.0/-/23.1 | | |
| TBE01[100)] | | | -,0.165 | | 27.1/-/- | | 25.4/-/- | 12.4 | 75 |
| TBE02[100)] | | | -,0.165 | | 27.7/-/- | | 25.8/-/- | 11.4 | |
| TRZCzPh-BNCz[101)] | 513 | 37 | 0.17,0.68 | | | 101.4/83.9/55.4 | 32.5/30.5/22.9 | 6.1 | 76 |
| TRZTPh-BNCz[101)] | 513 | 33 | 0.16,0.70 | | | 99.5/84.1/57.7 | 31.4/29.5/23.1 | 6.1 | |
| VTCzBN[102)] | 499 | 38 | 0.14,0.56 | 31691 | 91.0/71.4/56.9 | 66.4/48.7/33.1 | 31.7/24.8/19.8 | 21.8 | 77 |
| TCz-VTCzBN[102)] | 524 | 37 | 0.22,0.71 | 38657 | 129.3/72.2/64.2 | 96.7/49.3/37.3 | 32.2/18.0/16.0 | 44.1 | |
| S-Cz-BN[103)] | 496 | 29 | 0.18,0.49 | 15246 | 59.9 | 47.1 | 25.6 | - | 290 |
| PCzBN3[104)] | 496 | | 0.12,0.54 | 2790 | 43.4 | 38.9 | 17.5 | - | 78 |
| PCzBN5[104)] | 496 | | 0.11,0.53 | 3139 | 31.1 | 32.5 | 13,3 | - | |
| BSS-Cz[105)] | 462 | 29 | 0.13,0.09 | 3257 | 18.8/12.8/- | | 21.8/15.6/- | 28.4 | 79 |
| m-Cz-BNCz[106)] | 504 | 29 | 0.11,0.61 | 20350 | 94.9 | 99.2 | 36.8/33.4/19.0 | 9.2 | 80 |
| m-DPAcP-BNCz[106)] | 496 | 28 | 0.09,0.54 | 13990 | 93.9 | 96.2 | 42.0/37.6/17.5 | 4.4 | |
| m-SF-BNCz[106)] | 492 | 28 | 0.09,0.48 | 10990 | 72.5 | 71.7 | 35.0/24.4/10.9 | 30.2 | |
| *m*-BN-BNCz[106)] | 496 | 28 | 0.09,0.53 | 16610 | 91.1 | 90.8 | 41.1/37.0/17.9 | 9.9 | |
| Cl-MR[107)] | 472 | 30 | 0.12,0.19 | 209 | | | | 17 | 81 |
| Br-MR[107)] | 476 | 39 | 0.14,0.25 | 10.3 | | | | 4.2 | |
| TCZ-F-DABNA[108)] | 588 | 61 | 0.54,0.44 | | 106.7 | 108.1 | 39.2/24.4/7.87 | 38 | 82 |
| DBNS[109)] | 613 | [83] | 0.63,0.35 | | 7.2 | | 5.8 | - | |
| DBNS-*t*Bu[109)] | 616 | | 0.65,0.34 | | 9.2 | | 7.8 | - | |
| mICz-DABNA[110)] | 466 | 26 | 0.13,0.11 | | 20.1 | | 26.4 | - | 84 |
| BFCz-DABN[110)] | 463 | 26 | 0.13,0.09 | | 23.1 | | 28.0 | - | |
| C-BN[111)] | 453 | 25 | 0.14,0.07 | | 20.1/13.8/6.0 | | 26.6/20.1/8.9 | - | 85 |
| Cz-BSeN[112)] | 490 | 36 | 0.13, 0.45 | | 44.0/36.7/- | | 20.3/16.9/- | 16.7 | 86 |
| DCz-BSeN[112)] | 481 | 32 | 0.11, 0.25 | | 32.3/280.4/- | | 22.3/19.6/- | 12.1 | |
| pBP-DABNA-Me[113)] | 464 | 23 | 0.13,0.092 | | 17.4 | | 23.4 | - | 87 |
| pBP-DABNA-Me[114)] | 462 | 22 | 0.13,0.109 | | 21.4 | | 30.1 | - | |
| BN-Se[115)] | 506 | 45 | 0.15,0.62 | > 100000 | 95.9 | 103.9 | 32.6 | 1.3% at 1000 cd $m^{-2}$ | 88 |
| Czp-*t*BuCzB[116)] | 479 | 24 | 0.11,0.21 | 13870 | 38.7/35.3/37.4 | 35.9 | 32.1/29.2/30.9 | 9.0 at 100cd $m^{-2}$ 3.7 at 1000cd $m^{-2}$ | 89 |
| Czp-POAB[116)] | 513 | 48 | 0.23,0.65 | 38643 | 96.7/94.5/68.9 | 98.0 | 28.7/28.1/20.4 | 2.1 | |
| BN-DICz[117)] | 541 | 27 | 0.30,0.56 | | 123.3/-/70 | 88.6/-/36.7 | 31.5/-/17.8 | | 90 |
| DBN-ICz[117)] | 551 | 23 | 0.36,0.59 | | 136.6/-/101 | 119.2/-/62 | 37.4/-/23.6 | - | |
| BN-R[118)] | 617 | 47 | 0.65,0.34 | 17083 | 30.2/-/- | 31.2/-/- | 22.0/15.4/7.1 | 30 | 91 |
| PhDMAC-BN[119)] | 480 | 35 | 0.108,0.223 | 6491 | 48.1/35.5/13.9 | 46.6/22.3/4.8 | 33.9/25.1/9.7 | 25.9 | 92 |
| LTCz-BN[120)] | 500 | 33 | 0.13,0.60 | 38099 | 71.0/-/- | 33.3/-/- | 27.2/-/- | | 93 |
| ***ω***-DABNA[121)] | 512 | 25 | 0.13,0.73 | > 10000 | 101.8 | 88.8 | 31.1/30.8/29.4 | 9.6 | 94 |
| p-1-PCzBN[122)] | 496 | 26 | 0.08,0.52 | | 71.4/-/56.0 | 51.0/-/38.2 | 33.9/-/26.5 | - | 96 |
| m-1-PCzBN[122)] | 500 | 30 | 0.10,0.56 | | 76.4/-/69.2 | 61.5/-/50.5 | 32.6/-/29.5 | - | |
| DTBA-BN2[123)] | 497 | 47 | | | | 58.8 | 31.2/28.2/25.6 | - | 97 |
| DTBA-B2N3[123)] | 475 | 28 | | | | 28.1 | 30.9/27.4/20.5 | - | |
| BNCz-pTPA[124)] | 496 | 34 | 0.12,0.54 | 8949 | 67.2/57.6/- | 58.7/41.2/- | 27.3/22.3/- | 18.3 | 98 |
| BNCz-mTPA[124)] | 496 | 31 | 0.11,0.53 | 5393 | 58.0/51.0/- | 48.0/39.4/- | 24.6/21.4/- | 13.0 | |
| DtBuCzB[125)] | 488 | 29 | 0.11,0.38 | 21233 | 40.4/-/30.5 | 39.6/-/17.7 | 21.7/-/16.4 | - | 291 |
| DtBuCzB[126)] | 488 | 28 | 0.12,0.38 | 30991 | 44.4/-40.0 | 40.6/-/22.4 | 23.9/-/21.5 | - | |
| *m*-Cz-BNCz[126)] | 504 | 43 | 0.20,067 | | 78.2/-/70.1 | 65.4/-/43.2 | 22.5/-/20.3 | - | |
| DPMX-CzDABNA[127)] | 484 | 29 | 0.10,0.32 | 7353 | 46.3/-/- | 45.5/-/- | 27.4/22.6/10.9 | 17.5 | 99 |
| DPMX-CzDABNA[128)] | | | 0.40,0.51 | 15890 | 99.1 | 94.3 | 35.8/34.4/26.0 | 3.9 | |
| BN-CP1[129)] | 494 | 28 | 0.14,0.47 | | 50.9 | 40.0 | 23.5/8.4/1.7 | 64.5 | 292 |
| BN-CP1[130)] | 492 | 28 | 0.14,0.46 | | 55.6 | 38.8 | 26.6/5.1/3.6 | 80.8 | |
| BN-STO[131)] | 517 | 34 | 0.19,0.70 | 93313 | 141.2 | 176.9 | 40.1/39.0/28.1 | 2.7 | 100 |
| BN-XTO[131)] | 516 | 34 | 0.19,0.70 | 87077 | 131.0 | 156.9 | 37.3/34.1/18.6 | 8.6 | |
| BN-STO[132)] | 511 | 32 | 0.15,0.66 | 109381 | 123.3 | 138.4 | 39.8/39.8/34.0 | 0 | |
| BNNO[133)] | 643 | 42 | 0.708,0.292 | | 25.2 | 24.1 | 34.4/-/31.4 | - | 101 |
| [B-N]N[134)] | 448 | 27.3 | 0.151,0.079 | 2002 | 14.9/-/7.1 | 12.8/-/3.4 | 16.7/-/7.6 | - | 102 |
| p[B-N]O[134)] | 493 | 24.6 | 0.163,0.514 | 112000 | 66.8/-/60.2 | 73.2/-/49.8 | 26.3/-/24.0 | - | |
| p[B-N]NO[134)] | 525 | 31.2 | 0.306,0.648 | 106000 | 102.0/-/96.6 | 101.0/-/75.9 | 27.6/-/26.3 | - | |
| p[B-N]N[134)] | 552 | 31.3 | 0.414,0.571 | 126900 | 83.0/-/79.6 | 71.9/-/56.9 | 24.6/-/23.6 | - | |
| DBTN-2[135)] | 520 | 29 | 0.19,0.74 | 42180 | 132.9 | 130.4 | 35.2/33.6/20.4 | 4.5 | 103 |
| *α*-3BNMes[136)] | 443 | 49 | 0.15,0.10 | | | | 14.6/10.2/- | 30.1 | 104 |
| NOBNacene[137)] | 412 | 41 | 0.176,0.068 | | | 3.51 | 11.2 | - | 105 |
| p-CzB[138)] | 511 | 41 | 0.16.0.66 | | 67.9 | 69.9 | 20.2/15.4/9.3 | 23.8 | 106 |
| m-CzB[138)] | 515 | 39 | 0.20,0.70 | | 85.1 | 94.7 | 23.5/21.0/15.0 | 10.6 | |
| BN-PhOH[139)] | 491 | 26 | 0.11,0.43 | 2337 | 34 | | 19.0/-/- | - | 107 |

**Table S4**. Performance of B/N type MR-emitters in optimized OLEDs

| | | | | | | | | | |
|---|---|---|---|---|---|---|---|---|---|
| BNPhOCH$_3$[139] | 493 | 29 | 0.10,0.47 | 4378 | 53 | | 25.6/-/- | - | |
| BN-PhN(CH$_3$)$_2$[139] | 492 | 28 | 0.10,0.46 | 3814 | 48 | | 24.1/19.6/- | 18.7 | |
| TMlnBN[140] | 480 | 31 | 0.13,0.26 | | 14.7 | 10.9 | 9.0 | - | 108 |
| MeS-TMlnBN[140] | 483 | 42 | 0.15,0.36 | | 15.1 | 11.1 | 7.3 | - | |
| Cz-TMlnBN[140] | 485 | 50 | 0.16,0.41 | | 14.2 | 10.1 | 6.1 | - | |
| TCz-TMlnBN[140] | 489 | 68 | 0.19,0.50 | | 13.3 | 9.4 | 4.9 | - | |
| BN4[141] | 423 | 31 | 0.17,0.04 | | | | 9.1 | - | 109 |
| NBO[142] | 459 | 45 | 0.137,0.142 | | 19.8/8.6/- | 17.6/5.0/- | 16.8/7.5/- | 55.4 | 110 |
| m-DiNBO[142] | 466 | 21 | 0.126,0.098 | | 20.0/14.1/- | 15.9/8.0/- | 24.2/17.3/- | 28.5 | |
| p-DiNBO[142] | 513 | 48 | 0.258,0.665 | | 79.5/71.7/33.3 | 60.4/48.5/17.5 | 21.6/19.5/9.2 | 9.7 | |
| NO-DBMR[143] | 469 | 26 | 0.12,0.12 | | 29.2 | | 33.7 | - | 111 |
| Cz-DBMR[143] | 491 | 22 | 0.09,0.34 | | 42.3 | | 29.8 | - | |
| CzBN1[144] | 523 | 75 | 0.28,0.57 | 182970 | 101.6 | 100.3 | 31.7/-/30.5 | - | 112 |
| CzBN2[144] | 481 | 75 | 0.28,0.49 | 113630 | 87.4 | 89.0 | 32.4/-/30.2 | - | |
| CzBN3[144] | 483 | 25 | 0.16,0.38 | 63753 | 72.0 | 77.8 | 36.4/-/30.3 | - | |
| CzBN3[145] | 487 | 27 | 0.17,0.34 | 71938 | 72.7 | 83.2 | 41.2/-/33.2 | - | |
| BN-TC[146] | 474 | 37 | 0.13, 0.20 | 4448 | 28.2 | 21.6 | 21.2/8.7/4.2 | 58.9 | 113 |
| BN-AC[146] | 484 | 27 | 0.10, 0.31 | 5089 | 35.1 | 31.5 | 23.4/15.9/5.5 | 36.3 | |
| BN-PXZ[146] | 482 | 25 | 0.23, 0.43 | 11710 | 51.2 | 48.7 | 24.4/15.5/8.2 | 36.5 | |
| BN-PZ[146] | 612 | 104 | 0.60, 0.40 | 44210 | 34.9 | 29.2 | 25.0/21.2/17.6 | 15.2 | |
| Tp-DABNA[147] | 462 | 25 | 0.14,0.11 | | 22.8/10.1/- | 19.9/6.1/- | 24.3/11.2/- | 53.9 | 114 |
| Tp-DABNA[148] | 462 | 29 | 0.14,0.13 | | 28.5/18.8/ | 29.8/13.1/- | 27.5/18.6/- | 33.4 | |
| p-TBNCz [149] | 488 | 26 | 0.09,0.35 | 12690 | 45.3 | 37.5 | 27.0/-/11.7 | - | 115 |
| m-DBCz [149] | 548 | 35 | 0.35,0.63 | 109087 | 145.8 | 134.2 | 34.9/-/31.3 | - | |
| m-DBCz [150] | 545 | 32 | 0.35,0.63 | 57600 | 153.4 | 151.8 | 37.1/-/34.4 | - | |
| m-DBCz [151] | 544 | 36 | 0.33,0.65 | 157800 | 155.3 | 168.2 | 36.7/-/32.2 | - | |
| m-DBCz [152] | 548 | 34 | 0.35,0.63 | 217700 | 151.5 | 162.5 | 36.3/-/35.1 | - | |
| (P)-BN-Py[153] $g_{EL}$=-4.4×10$^{-4}$ | 532 | 37 | 0.29,0.68 | 25910 | 119.6 | 118.9 | 30.6/23.7/10.0 | 22.5 | 116 |
| (M)-BN-Py[153] $g_{EL}$=4.4×10$^{-4}$ | 532 | 37 | 0.29,0.68 | 23760 | 110.6 | 110.1 | 29.2/23.7/10.1 | 18.8 | |
| OBN[154] | 437 | 44 | 0.15,0.09 | - | - | - | 23.02 | - | 117 |
| NBN[155] | 452 | 40 | 0.14,0.09 | - | - | - | 15.7 | - | |
| ODBN[155] | 446 | 54 | 0.15,0.10 | - | 9.71 | 8.02 | 24.5 | - | |
| tPh[BN] [154] | 463 | 48 | 0.14,0.20 | 38350 | 9.21 | 5.38 | 6.11 | - | 118 |
| Cz[BN] [154] | 474 | 50 | 0.14,0.29 | 52530 | 12.81 | 7.70 | 7.14 | - | |
| B4N6-Me [155] | 588 | 27 | 0.57,0.43 | - | 102.1 | 108.1 | 35.8/-/28.4 | - | 119 |
| (M,M)-RBNN [156] | 617 | 18 | 0.667,0.332 | 92690 | - | - | 36.6/26.7/21.3 | 27.1 | 120 |
| (P,P)-RBNN [156] | 617 | 48 | 0.667,0.331 | 92360 | - | - | 34.4/27.1/21.9 | 21.2 | |
| D1-BNN[157] | 483 | 38 | 0.11,0.36 | 1910 | 23.7 | - | 13.0 | - | 121 |
| D2-BNN[157] | 479 | 26 | 0.11,0.24 | 1970 | 20.5 | - | 14.8 | - | |
| D3-BNN[157] | 477 | 24 | 0.11,0.19 | 3050 | 20.5 | - | 17.3 | - | |
| BNIP-tBuCz[158] | 566 | 69 | 0.48,0.51 | 82521 | 104.2 | 116.9 | 32.8/29.6/15.5 | 9.7 | 122 |
| BNIP-tBuDPAC[158] | 554 | 58 | 0.41,0.56 | 119585 | 145.3 | 163.0 | 39.4/37.1/23.3 | 5.8 | |
| BNIP-CzDPA[158] | 584 | 62 | 0.54,0.45 | 72954 | 82.9 | 93.0 | 32.4/27.1/15.2 | 16.4 | |
| BNDIP[158] | 582 | 67 | 0.53,0.47 | 93864 | 97.9 | 108.1 | 34.1/33.5/25.5 | 1.8 | |
| Na-sBN[159] | 520 | 50 | 0.27,0.65 | 186923 | 103.1 | 115.7 | 28.8/28.7/28.2 | 0.35 | 124 |
| Na-dBN[159] | 621 | 38 | 0.66,0.34 | 71534 | 31.2 | 34.0 | 25.2/25.0/24.7 | 0.79 | |
| Cz-DABNA[160] | 472 | 16.6 | 0.138,0.190 | | 29.8/-/28.5 | 26.6/-/16.0 | 23.8/-/21.2 | - | 125 |
| t-BuCz-DABNA[160] | 472 | 16.6 | 0.139,0.189 | | 35.4/-/33.9 | 33.9/-/19.0 | 29.2/-/26.0 | - | |
| BN-TP-N1[161] | 532 | 34 | 0.28,0.69 | 19570 | 137.7 | 139.7 | 34.9/30.8/13.9 | 9.11 | 126 |
| BN-TP-N2[161] | 534 | 36 | 0.30,0.67 | 18070 | 129.5 | 139.8 | 31.9/28.5/11.1 | 10.6 | |
| BN-TP-N3[161] | 524 | 33 | 0.23,0.71 | 18670 | 135.3 | 139.9 | 37.3/34.7/19.8 | 6.9 | |
| BN-TP-N4[161] | 528 | 35 | 0.27,0.70 | 20840 | 136.3 | 143.2 | 36.5/34.1/21.2 | 6.6 | |
| PPZ-BN[162] | 613 | 55 | 0.66,0.34 | | 34.9/-/34.0 | 34.6/-/28.9 | 26.9/-/26.0 | - | 127 |
| Cz-DBCz[163] | 487 | 19 | 0.117, 0.367 | 64640 | 54.3/ 51.4/ 42.7 | 60.9/53.8, 33.5 | 31.9/ 29.8/ 25.8 | 6.6 | 128 |
| Cz-DBTPA[163] | 481 | 18 | 0.116, 0.264 | 43610 | 41.0/ 39.2/ 32.8 | 42.9/ 36.2/ 22.4 | 27.1/ 25.6/ 25.2 | 5.5 | |
| PhO-DBCz[163] | 473 | 27 | 0.126, 0.208 | 45090 | 40.9/ 40.2/ 36.2 | 45.9/ 43.6/ 33.5 | 29.5/ 29.1/ 26.2 | 1.3 | |
| TBA-BCz-BN[164] | 470 | 28 | 0.12, 0.15 | | | 31.3/14.8/7.0 | 30.8/19.0/11.7 | 38.3 | 129 |
| PXZ-R-BN[165] | 693 | 59 | - | - | - | - | 29.3/16.8/12.0 | 42.7 | 130 |
| BCz-R-BN[165] | 713 | 56 | - | - | - | - | 24.2/11.9/8.0 | 50.8 | |
| BSS-Ph-TBCz[166] | 468 | 31 | 0.12,0.11 | | 18.6 | | 21.4 | - | 131 |
| BSS-TBCz[166] | 462 | 34 | 0.03,0.09 | | 15.4 | | 19.9 | - | |
| DB[167] | 443 | 26 | 0.154,0.048 | 10486 | 10.6 | 8.3 | 23.4/17.4/9.0 | 25.6 | 132 |
| (M)-DB-O[167] | 445 | 24 | 0.150, 0.041 | 11610 | 11.9 | 10.4 | 27.5/21.3/11.4 | 22.5 | |
| (P)-DB-O[167] | 445 | 24 | 0.150, 0.041 | 12055 | 11.4 | 9.4 | 26.2/20.5/11.5 | 21.8 | |
| (M)-DB-S[167] | 447 | 24 | 0.148, 0.047 | 11153 | 14.7 | 11.5 | 29.3/23.6/12.2 | 19.5 | |
| (P)-DB-S[167] | 447 | 24 | 0.148, 0.048 | 10888 | 14.4 | 11.3 | 28.9/23.5/12.0 | 18.7 | |
| BNB′-1[168] | 539.9 | 30 | 0.33,0.64 | | 99.9 | 95.1 | 36.2/15.5/10.4 | 57.2 | 133 |
| BNB′-1[169] | 538.9 | 30.6 | 0.32,0.66 | | 120.2 | 121.8 | 40.3/27.2/24.3 | 32.5 | |
| TCZBAC[170] | 500 | 40 | 0.13,0.56 | 2045 | 45.4 | 46.0 | 17.3/14.2/6.6 | 17.9 | 134 |
| TCZBAO[170] | 520 | 36 | 0.20,0.70 | 15090 | 90.5 | 101.5 | 25.1/21.7/14.0 | 13.5 | |
| TCZBAC[171] | 502 | 46 | 0.16,0.56 | 13880 | 88.2 | 92.3 | 32.4/31.4/26.8 | 3.08 | |
| TCZBAO[171] | 522 | 39 | 0.22,0.69 | 27970 | 131.8 | 143.9 | 36.1/32.7/29.2 | 9.4 | |
| h-BNCO-1[172] | 528 | 39 | 0.24,0.71 | | 159.2 | 147.0 | 40.1/-/34.6 | - | 135 |
| Py-Cz-BN[173] | 587 | 54 | 0.55,0.45 | | | | 29.6/-/23.6 | - | 136 |
| PSeZBN1[174] | 475 | 27 | | 22900 | 52.9/-/31.5 | 59.0/-/27.0 | 34.8/-/22.8 | - | 137 |
| PSeZBN2[174] | 517 | 57 | | 159300 | 104.9/-/104.0 | 109.7/-/78.4 | 29.5/-/29.0 | - | |
| P-BN[9]H[175] | 580 | 48 | | 29950 | | | 35.4/33.1/23.9 | 6.5 | 138 |
| M-BN[9]H[175] | 580 | 48 | | 30390 | | | 35.5/33.4/18.8 | 5.9 | |
| 6z[176] | 513 | 27 | 0.18,0.70 | 110922 | 109.2 | 122.6 | 31.7 | - | 139 |
| 10b[177] | 476 | 35 | 0.13,0.26 | 62359 | 64.7 | 61.9 | 40.1 | - | |
| TPABO-DICz[178] | 522 | 22 | 0.24,0.74 | | 84.1/-/82.0 | 100.5/-/78.5 | 25.8/-/25.6 | - | 140 |
| B-N-1 [179] | 457 | 38 | 0.135,0.100 | | 4.3 | 3.4 | 4.9 | - | 141 |
| B-N-S-1 [179] | 491 | 40 | 0.109,0.426 | | 10.5 | 8.0 | 5.1 | - | |
| B-N-S-2 [179] | 465 | 41 | 0.127,0.128 | | 4.5 | 3.4 | 4.5 | - | |
| B-N-S-3 [179] | 461 | 35 | 0.128,0.119 | | 6.5 | 5.2 | 6.7 | - | |
| BN-Cz[180] | 561 | 52 | 0.44,0.55 | 74640 | 114.0 | 130.8 | 32.9/27.5/18.4 | 16.4 | 142 |
| BN-Cb[180] | 559 | 47 | 0.43,0.56 | 108900 | 113.1 | 131.6 | 29.7/22.9/17.1 | 22.9 | |
| SPACtCzBN[181] | 480 | 25 | | | | 44.8 | 35.1 | - | 250 |
| SPBAC-tCzBN[181] | 480 | 25 | | | | 49.7 | 36.5 | - | |
| o-SPAC-tCzBN[181] | 492 | 26 | | | | 64.8 | 34.1 | - | |
| PCzDBN1[182] | 477 | 28 | 0.12,0.21 | 1623 | 21.0 | 18.9 | 15.6/7.6/- | 51.3 | 143 |
| PCzDBN3[182] | 479 | 28 | 0.11,0.25 | 1831 | 26.5 | 23.8 | 17.9/8.2/- | 54.2 | |
| PCzDBN5[182] | 480 | 29 | 0.11,0.27 | 1472 | 22.1 | 19.8 | 15.0/8.7/- | 42 | |
| BN-36Cz-BN[183] | 495 | 31 | 0.11,0.50 | 4113 | 61.8 | 55.4 | 27.1/-/2.9 | - | 144 |
| BN-27Cz-BN[183] | 498 | 33 | 0.13,0.57 | 5104 | 53.5 | 42.0 | 20.9/-/3.3 | - | |
| 2CB-BuDABNA[184] | 472 | 27 | 0.128,0.187 | | 26.8/20.1/- | 31.1/17.6/- | 25.0/16.9/- | 32.4 | 145 |

**Table S4**. Performance of B/N type MR-emitters in optimized OLEDs

| | | | | | | | | | |
|---|---|---|---|---|---|---|---|---|---|
| BuDABNA[184] | 460 | 27 | 0.137,0.093 | | 19.3/10.0/- | 22.4/7.5/- | 25.1/12.7/- | 49.4 | |
| IDID2BN[185] | 534 | 29 | 0.31,0.65 | 188000 | 146.3 | 135.2 | 36.6/-/34.5 | - | 251 |
| IDAD-BNCz[186] | 492 | 29 | 0.088, 0.421 | | 66.5 | 55.0 | 30.9/24.8/10.3 | 19.7 | 146 |
| TIDAD-BNCz[186] | 498 | 30 | 0.095, 0.448 | | 68.8 | 59.8 | 31.3/24.7/11.4 | 21.1 | |
| BNCzPXZ[187] | 520 | 49 | 0.25,0.67 | 7262 | 93.7 | 91.9 | 25.7/21.1/10.4 | 17.9 | 49 |
| BNCzPTZ[187] | 524 | 57 | 0.27,0.65 | 13240 | 105.0 | 103.0 | 28.7/27.9/20.7 | 2.8 | |
| MFCzBN[188] | 508 | 34 | 0.152,0.615 | 156850 | 78.2 | 76.0 | 24.3/23.9/23.7 | 1.6 | 147 |
| SFCzBN[188] | 508 | 34 | 0.145,0.635 | 61747 | 74.1 | 72.2 | 24.7/18.7/12.6 | 24.3 | |
| PTZBNO[189] | 618 | 58 | 0.64,0.36 | 138000 | 50.1 | 62.6 | 34.5/-/29.7 | - | 148 |
| PXZBNO[189] | 632 | 54 | 0.67,0.33 | 91000 | 25.1 | 28.2 | 28.1/-/23.8 | - | |
| CzBN-tDPA[190] | 475 | 29 | 0.12,0.21 | 19659 | 26.6 | | 19.3/-/16.4 | - | 149 |
| CzBN-mCP[190] | 471 | 26 | 0.12,0.16 | 13234 | 23.6 | | 18.1/-/13.9 | - | |
| CzBN-tDPA[191] | 470 | 30 | 0.13,0.16 | 20760 | 37.8 | | 30.6/-/25.6 | - | |
| CzBN-mCP[191] | 469 | 28 | 0.13,0.14 | 15749 | 32.2 | | 30.6/-/23.8 | - | |
| tBOSi[192] | 414 | 32 | 0.165,0.034 | 224.4 | 1.67 | 0.94 | 9.15 | - | 150 |
| tBOSiCz[192] | 414 | 32 | 0.163,0.031 | 220.9 | 1.59 | 0.83 | 8.91 | - | |
| tCzMe3Si[193] | 501 | 31 | 0.13,0.60 | 11289 | 84.1/76.7/43.7 | 53.4/38.9/17.7 | 30.5/23.6/15.5 | 22.6 | 151 |
| tCzPh3Si[193] | 500 | 28 | 0.08,0.58 | 13148 | 64.8/57.5/29.8 | 42.4/36.1/16.5 | 32.0/28.4/16.3 | 11.3 | |
| tPhCzMe3Si[193] | 511 | 35 | 0.18,0.67 | 11914 | 113.8/73.8/43.3 | 85.1/50.4/23.1 | 33.9/22.2/13.0 | 34.5 | |
| tPhCzPh3Si[193] | 512 | 28 | 0.14,0.70 | 15368 | 112.3/84.6/52.2 | 80.1/56.8/28.3 | 34.6/26.1/16.1 | 24.6 | |
| tCzBN-PQ[194] | 524 | 37 | 0.25,0.67 | 22300 | 115.6 | 94.3 | 30.2/-/15.6 | - | 152 |
| tCzBN-PQCz[194] | 516 | 34 | 0.20,0.69 | 27000 | 131.0 | 106.9 | 35.1/-/22.6 | - | |
| CzBN[195] | 478 | 39 | 0.142, 0.287 | 48104 | 48.7 | 38.2 | 22.0/20.1/17.1 | 8.6 | 153 |
| CzBNNa[195] | 484 | 42 | 0.149, 0.360 | 56610 | 41.8 | 50.5 | 20.5/19.6/17.0 | 4.4 | |
| CzBNPyr[195] | 483 | 34 | 0.133, 0.318 | 34872 | 41.5 | 37.1 | 19.4/17.4/15.1 | 10.3 | |
| CzCzB[196] | 559 | 48 | 0.43,0.56 | | 74.8 | 36.3 | 19.0/9.7/6.7 | 43.7 | 154 |
| RBNO1[197] | 632 | 55 | 0.689,0.311 | 63800 | 26.6 | 34.8 | 33.1/-/22.5 | - | 155 |
| RBNO2[197] | 645 | 48 | 0.700,0.300 | 59000 | 18.5 | 20.7 | 34.7/-/28.8 | - | |
| DCzBNO[198] | 469 | 45 | 0.13,0.15 | 17990 | | | 31.7 | - | 156 |
| TCzBNO[198] | 482 | 52 | 0.13,0.25 | 31839 | | | 36.5 | - | |
| SAC2MN1B[199] | 470 | 36 | 0.125,0.163 | 1236 | 25.52 | 19.68 | 21.87 | - | 157 |
| DPA2MN2B[199] | 456 | 45 | 0.148,0.120 | 740 | 12.39 | 8.38 | 12.04 | - | |
| Cz2MN2B[199] | 486 | 35 | 0.113,0.401 | 1386 | 27.67 | 17.34 | 13.89 | - | |
| SAC2MN2B[199] | 448 | 25 | 0.147,0.061 | 770 | 12.04 | 8.79 | 19.80 | - | |
| IDIDBN[200] | 535 | 40 | 0.33,0.63 | 135320 | 68.8 | 68.6 | 16.3/9.9/8.5 | 39.3 | 158 |
| tBuIDIDBN[200] | 535 | 38 | 0.31,0.60 | 141644 | 73.2 | 81.6 | 18.3/12.6/11.1 | 31.1 | |
| OP-BN[201] | 480 | 32 | 0.11,0.23 | | 35.4/25.4/7.93 | 39.8/18.4/3.53 | 26.0/18.6/5.67 | 28.5 | 159 |
| Cz-OP-BN[201] | 480 | 32 | 0.11,0.28 | | 41.1/30.8/9.69 | 43.1/22.7/4.73 | 26.8/19.8/6.21 | 26.1 | |
| 2Cz-OP-BN[201] | 480 | 32 | 0.11,0.32 | | 49.7/38.2/14.5 | 51.4/29.6/7.82 | 29.6/22.5/8.66 | 23.98 | |
| CFDBO[202] | 460 | 24 | 0.14,0.12 | 8985 | 21.4 | 17.2 | 20.7 | - | 160 |
| CFDBA[202] | 473 | 21 | 0.13,0.18 | 12985 | 68.0 | 56.2 | 30.9 | - | |
| CFDBCz[202] | 488 | 22 | 0.12,0.43 | 20474 | 75.8 | 59.5 | 32.4 | - | |
| CzBN4-oPh[203] | 490 | 21 | 0.09,0.45 | | 57.0/55.0/50.8 | 55.9/44.0/33.8 | 28.7/27.8/25.8 | 3.1 | 161 |
| Π-CzBN[204] | 495 | 21 | 0.08,0.50 | | 73.4 | 54.9 | 38.0/-/18.4 | - | 162 |
| Π-CzBN[205] | 495 | 21 | 0.08,0.48 | | 79.3 | 65.6 | 39.3/-/31.4 | - | |
| BN-NAP[206] | 514 | 37 | 0.24,0.65 | | | | 19.2/16.6/14.1 | 13.5 | 163 |
| BN-ANAP[206] | 524 | 29 | 0.26,0.67 | | | | 21.0/17.7/15.7 | 12.9 | |
| BNCz-aDMAC[207] | 484 | 27 | 0.10,027 | | 42.9 | 40.8 | 29.1 | - | 164 |
| BNCz-PaDMAC[207] | 504 | 52 | 0.16,0.62 | | 99.4 | 94.6 | 32.7 | - | |
| BO-N1[208] | 494 | 45 | 0.16,0.38 | 6698 | 28.2 | 32.1 | 18.6 | - | 165 |
| BO-N2[208] | 502 | 29 | 0.17,0.50 | 10480 | 36.4 | 41.9 | 20.1 | - | |
| BNCz-SAF[209] | 480 | 24 | 0.10,0.24 | 4010 | 33.89 | 31.30 | 25.3/14.4/- | 43.1 | 166 |
| BNCz-DMAC[209] | 480 | 25 | 0.10,0.26 | 3839 | 33.09 | 31.97 | 23.8/13.4/- | 43.7 | |
| BNCz-PXZ[209] | 482 | 23 | 0.21,0.41 | 8385 | 44.84 | 44.00 | 20.9/17.5/- | 16.3 | |
| TTABN[210] | 464 | 31 | 0.14,0.13 | 1334 | 19.3 | 16.9 | 19.2/-/4.8 | - | 167 |
| TAzBN[210] | 482 | 45 | 0.13,029 | 2892 | 47.8 | 42.9 | 27.3/-/16.6 | - | |
| Me-PABO[211] | 460 | 26 | 0.134, 0.089 | 984 | 16.55/5.52/- | 12.99/2.03/- | 20.4/5.90/- | 71.1 | 168 |
| Me-PABS[211] | 468 | 32 | 0.121, 0.139 | 2300 | 24.00/9.86/3.70 | 22.21/4.99/1.04 | 23.0/9.84/3.01 | 57.2 | |
| DG7[212] | 559 | 23 | 0.41,0.55 | 9176 | | 7/-/2.9 | 2.8/-/1.8 | - | 169 |
| BN-Ad[213] | 500 | 35 | 0.14,0.57 | 9826 | 59.6 | 48.0 | 32.3/22.3/13.3 | 30.9 | 170 |
| BN-Ph[213] | 492 | 28 | 0.17,0.40 | 2836 | 9.9 | 7.9 | 6.1/5.1/2.5 | 16.4 | |
| DPC[214] | 464 | 47 | 0.14,0.15 | | | 59.6/-/28.5 | 38.6/-/29.3 | - | 171 |
| DTP[214] | 476 | 53 | 0.14,0.25 | | | 58.6/-/28.3 | 28.1/-/22.6 | - | |
| CzIDBNO[215] | 643 | 47 | 0.701,0.298 | 62137 | 20.2/-15.6 | 24.1/-/10.6 | 32,5/-/23.8 | - | |
| IDIDBNO[215] | 671 | 49 | 0.702,0.297 | 54191 | 8.4/-/8.4 | 8.8/-/4.6 | 27.2/-/21.1 | - | 171 |
| ω-DABNA[216] | 512 | 25 | 0.13,0.73 | - | 101.9 | 106.2 | 30.8/30.4/29.2 | 1.3 | 95 |
| ω-DABNA-M[216] | 515 | 25 | 0.15,0.74 | - | 114.1 | 114.5 | 32.7/32.0/27.4 | 2.2 | |
| ω-DABNA-PH[216] | 521 | 30 | 0.19,0.74 | - | 123.6 | 124.8 | 31.8/30.5/27.4 | 4.1 | |
| ω-DABNA-M[217] | 517 | 23 | 0.17,0.70 | 72622 | 94.2 | 98.7 | 28.2/25.3/22.6 | 10.3 | |
| ω-DABNA-PH[217] | 522 | 29 | 0.21,0.69 | 155964 | 94.2 | 98.6 | 26.5/24.9/23.1 | 6.03 | |
| A-BN[218] | 462 | 24 | 0.13,0.08 | | 31.9/17.4/7.58 | | 41.5/23.6/10.4 | 43.1 | 173 |
| A-BN[219] | 462 | 25 | 0.13,0.09 | | | | 38.5/32.7/25.1 | 15.1 | |
| ν-DABNA-Az1[220] | 459 | 19 | 0.136,0.083 | | 24.0 | 25.8 | 30.8/29.0/19.9 | 5.8 | 174 |
| ν-DABNA-Az2[220] | 458 | 17 | 0.140,0.060 | | 18.1 | 19.4 | 29.9/28.0/16.0 | 6.4 | |
| ν-DABNA-Az3[220] | 459 | 20 | 0.136,0.100 | | 29.9 | 32.0 | 33.0/29.2/22.4 | 11.5 | |
| 5Cz-BO[221] | 416 | 36 | 0.163,0.046 | | 8.85 | 7.72 | 22.8 | - | 175 |
| DABNA-3B[222] | 475 | 25/0.14 | - | 41634 | 78.7/76.6/57.0 | 37.2/32.3/21.7 | 33.8/30.6/25.1 | 9.5 | 176 |
| BCzBN-3B[222] | 493 | 22/0.11 | - | 64666 | 79.3/75.1/55.0 | 103.8/83.6/46.4 | 42.6/40.4/30.5 | 5.2 | |
| pPSe-BN[223] | 500 | 35 | 0.12,0.57 | 25020 | 90.7 | 95.0 | 33.2/-/27.1 | - | 177 |
| mPSe-BN[223] | 494 | 30 | 0.09,0.48 | 14722 | 68.9 | 67.6 | 31.8/-/15.1 | - | |
| R-BA23CzBN[224] | 504 | | 0.13, 0.52 | 55892 | 87.6/74.2/45.1 | 114.6/100.1/38.7 | 36.6/31.2/19.1 | 14.7 | 178 |
| S-BA23CzBN[224] | 503 | | 0.13, 0.52 | 47934 | 83.5/68.7/40.6 | 109.3/100.0/36.1 | 34.6/28.6/16.9 | 17.3 | |
| R-BA34CzBN[224] | 528 | | 0.19, 0.71 | 104773 | 122.7/113.4/84.8 | 160.6/130.8/77.6 | 36.0/32.9/25.0 | 8.61 | |
| S-BA34CzBN[224] | 526 | | 0.17, 0.70 | 105195 | 121.3/110.3/81.7 | 158.8/128.1/77.6 | 36.1/33.1/25.0 | 8.31 | |
| V-DABNA[225] | 483 | 17 | 0.09,0.27 | | 36.2/35.8/35.6 | 33.1/29.6/26.1 | 26.2/26.2/26.2 | 0 | 179 |
| V-DABNA-F[225] | 468 | 15 | 0.12,0.10 | | 22.3/21.7/21.3 | 21.5/18.6/15.7 | 26.6/26.1/25.8 | 1.88 | |
| BpIC-DPA[226] | 536 | 29 | 0.30,0.67 | | 81.8/-/68.9 | | 22.0/-/18.6 | - | 180 |
| BpIC-Cz[226] | 544 | 32 | 0.34,0.64 | | 94.0/-/88.6 | | 25.7/-/24.3 | - | |
| BDBF-BOH[227] | 467 | 30/0.17 | 0.13,0.12 | 8491 | 29.1/20.7/15.4 | 21.2/12.7/8.3 | 29.5/19.5/15.8 | 33.9 | 181 |
| BDBT-BOH[227] | 467 | 30/0.17 | 0.13,0.12 | 7544 | 29.9/25.1/19.6 | 23.1/17.5/11.6 | 30.1/25.2/19.7 | 16.3 | |
| BN-N-TPA[228] | 504 | 35 | 0.13,0.64 | 13680 | 83.5 | 87.4 | 26.7/9.2/3.4 | 65.5 | 182 |
| TPA-Cz-BN[228] | 496 | 29 | 0.08,0.52 | 17160 | 84.5 | 89.2 | 34.4/30.3/13.7 | 11.9 | |
| TPA-PCz-BN[228] | 496 | 26 | 0.08,0.47 | 10520 | 73.6 | 73.2 | 32.3/29.6/14.4 | 8.4 | |
| BN-PCz-TPA[228] | 496 | 27 | 0.08,0.51 | 14690 | 84.4 | 87.9 | 36.3/31.8/13.8 | 12.4 | |
| *f*-DOABNA[229] | 445 | 28 | 0.153,0.056 | 4858 | 11.4 | 8.4 | 19.9/12.9/6.7 | 35.2 | 183 |
| Py-BN[230] | 444 | 21 | 0.153,0.045 | 1967 | 6.7 | 5.8 | 15.8/7.4/3.9 | 54.5 | 184 |
| Pm-BN[230] | 415 | 24 | 0.161,0.045 | 1261 | 2.0 | 1.4 | 5.8/4.3/2.5 | 25.9 | |
| Py-BN[230] | 445 | 22 | 0.150,0.052 | 6467 | 17.0 | 16.2 | 27.7/25.0/16.1 | 9.7 | |

**Table S4**. Performance of B/N type MR-emitters in optimized OLEDs

| | | | | | | | | | |
|---|---|---|---|---|---|---|---|---|---|
| h-BNCO-1[231] | 525 | 39 | 0.23,0.71 | | | | 25.1/-/10.7 | - | 293 |
| | 529 | 39 | 0.26,0.70 | | | | 24.9/-/18.1 | - | |
| | 530 | 39 | 0.26,0.68 | | | | 16.3/-/15.7 | - | |
| | | | 0.27,0.69 | | | | 17.9/-/16.6 | - | |
| DOB2-DABNA-A[232] | 452 | 24 | 0.145,0.049 | | 12.1/12.1/11.1 | 11.3/8.6/6.1 | 24.1/23.3/21.6 | 3.3 | 185 |
| DOB2-DABNA-B-NP[232] | 471 | 23 | 0.117,0.127 | | 26.9/25.4/22.6 | 26.1/19.2/13.6 | 29.1/26.7/24.0 | 8.2 | |
| QB-U[233] | 453 | 23/0.14 | 0.142, 0.064 | 11853 | 15.5 | 12.2 | 24.7/-/11.5 | - | 186 |
| QB-J[233] | 457 | 17/0.10 | 0.142, 0.064 | 16281 | 18.7 | 14.7 | 30.8/-/20.9 | - | |
| QB-I[233] | 466 | 15/0.08 | 0.127, 0.078 | 29526 | 22.2 | 17.0 | 32.5/-/30.4 | - | |
| DPA-B2[234] | 444 | 31 | 0.153,0.055 | 21620 | 14.8 | 12.9 | 28.9/19.8/9.3 | 31.5 | 187 |
| DPA-B3[234] | 450 | 15 | 0.150,0.043 | 24637 | 17.3 | 15.1 | 37.7/33.7/21.0 | 10.6 | |
| DPA-B4[234] | 457 | 14 | 0.141,0.050 | 27911 | 21.8 | 18.2 | 39.2/36.4/28.7 | 7.1 | |
| Cz-B4[234] | 457 | 26 | 0.138,0.076 | 27428 | 22.4 | 19.5 | 32,1/26.9/16.1 | 16.2 | |
| DPA-B4(HF)[235] | 459 | 16 | 0.142,0.099 | 41607 | 40.7 | 34.8 | 44.6/44.3/38.8 | 0.7 | |
| DPA-B4(tandem)[236] | 458 | 17 | 0.142,0.116 | 56246 | 77.3 | 33.7 | 74.5/73.3/65.3 | 1.6 | |
| BNCZ-DPAB[237] | 636.9 | 53 | 0.697,0.305 | | 15.4 | 12.7 | 22.2/-/1.5 | | 188 |
| AN-BN[238] | 608 | 54 | 0.63,0.37 | 192000 | 62.6 | 56.1 | 27.3/-/- | | 189 |
| ($R_{mt}$)-2[239] | 500 | 36 | 0.18,0.59 | 1566 | 44.5 | 29.9 | 15.6 | | 190 |
| ($S_{mt}$)-2[239] | 500 | 36 | 0.18,0.59 | 1654 | 49.9 | 29.7 | 17.6 | | |
| TB-PB[240] | 479 | 15 | 0.10,0.20 | 91030 | 49.1/-/44.7 | 51.4/-/35.1 | 36.4/-/32.4 | | 192 |
| L-DABNA-1[241] | 544 | 41 | 0.33,0.64 | 19730 | 169.6 | 121.0 | 40.9/36.3/12.8 | 11.2 | 193 |
| BNSi[242] | 484 | 27 | | | 51.3 | | 33.9/23.6/6.5 | 30.4 | 194 |
| $CH_2$-SFBN[243] | 492 | 28 | | | 50.0/36.9/19.9 | 39.6/24.3/10.2 | 28.6/21.1/11.3 | 26.2 | 195 |
| O-SFBN[243] | 492 | 28 | | | 50.0/34.4/17.6 | 38.8/23.8/9.6 | 32.0/21.9/11.2 | 25.3 | |
| S-SFBN[243] | 488 | 26 | | | 50.0/34.4/17.6 | 48.0/24.8/14.0 | 36.6/26.0/14.7 | 28.9 | |
| Se-SFBN[243] | 488 | 27 | | | 60.0/51.0/37.2 | 47.7/36.3/21.8 | 35.6/30.3/22.1 | 14.9 | |
| CO-SFBN[243] | 488 | 25 | | | 30.0/23.6/11.6 | 23.1/14.8/5.5 | 20.0/15.6/7.8 | 22 | |
| FSBN[244] | 624 | 58/0.18 | 0.67,0.33 | 22080 | 43.3 | 50.1 | 37.5/29.6/21.3 | 21.1 | 196 |
| FSBN[245] | 624 | 59/0.18 | 0.66,0.33 | 89270 | 41.6 | 49.4 | 38.5/37.2/32.3 | 3.7 | |
| S-BN[246] | 600 | 58/0.20 | 0.61,0.39 | 45540 | 74.5 | 93.6 | 39.9/33.1/26.3 | 17.04 | 197 |
| 2S-BN[246] | 676 | 62/0.16 | 0.70,0.28 | 6811 | 4.2 | 4.4 | 29.3/21.1/16.7 | 27.9 | |
| DtCzBN-CNBT1[247] | 508 | 38/0.18 | 0.17,0.66 | 5400 | | | 37.1/23.4/11.9 | 36.9 | 198 |
| DtCzBN-CNBT2[247] | 508 | 37/0.18 | 0.17,0.67 | 7800 | | | 40.2/29.5/20.7 | 26.6 | |
| Exo-D1[248] | 458 | 41 | 0.15,0.16 | | | | 15.0/10.6/- | | 199 |
| Endo-D1[248] | 476 | 34 | 0.11,0.19 | | | | 15.4/10.2/- | | |
| Exo-D2[248] | 460 | 41 | 0.15,0.17 | | | | 17.7/10.8/- | | |
| Endo-D2[248] | 472 | 38 | 0.13,0.20 | | | | 22.4/16.3/- | | |
| BNTPA[249] | 617 | 48/0.15 | 0.657, 0.343 | | 59.6 | 64.5 | 35.2 | | 200 |
| BNTPA[250] | 619 | 48/0.15 | 0.663, 0.337 | | 70.8 | 76.7 | 43.3 | | |
| (rac)-S-AX-BN[251] | 495 | 22 | 0.066,0.481 | | 71.5 | 57.5 | 33.5/27.9/23.2 | 16.7 | 201 |
| (rac)-SO2-AX-BN[251] | 500 | 21 | 0.068,0.579 | | 66.2 | 56.2 | 31.5/23.7/20.3 | 24.8 | |
| BN-TP-ICz[252] | 540 | 38 | 0.33,0.65 | 20940 | 135.0 | 140.7 | 32.0/20.3/6.1 | 36.6 | 202 |
| (R/S)-4-POtBuCzB[253] | 491 | 25 | 0.08,0.43 | | 60.9 / 57.5 / 29.4 | 53.6 | 31.3 / 29.7 / 15.7 | 5.1 | 203 |
| (rac)-2-POtBuCzB[253] | 493 | 28 | 0.10,0.44 | | 72.4 / 72.0 / 56.6 | 67.1 | 37.0 / 36.9 / 29.1 | 0.3 | |
| DBN-NaMe[254] | 514 | 20/0.095 | 0.18, 0.70 | | 97.7 | 122.7 | 27.8/19.6/15.9 | 29.5 | 204 |
| DBN-NaPh[254] | 526 | 20/0.091 | 0.23, 0.72 | | 126.6 | 159.0 | 31.5/26.7/21.7 | 15.2 | |
| DBN-NaPh-d[254] | 526 | 19/0.087 | 0.21, 0.74 | | 142.8 | 187.0 | 35.2/26.4/21.4 | 25 | |
| QB-DPA[255] | 502 | 17/0.079 | 0.10,0.61 | 63282 | 78.6 | 89.1 | 36.0/-/30.4 | | 205 |
| QB-PXZ[255] | 516 | 20/0.093 | 0.18,0.74 | 110397 | 134.8 | 151.3 | 36.6/-/31.8 | | |
| NT-2B[256] | 516 | 23.5 | 0.18,0.70 | 65056 | | | 30.5/24.3/17.8 | 20.3 | 206 |
| NT-3B[256] | 514 | 43.0 | 0.25,0.66 | 63705 | | | 25.5/19.2/14.6 | 24.7 | |
| Cz-CN-BN[257] | 504 | 31/0.15 | | 37258 | 98.6/85.2/49.2 | 91.1/73.2/35.9 | 35.7/32.1/18.5 | 10.1 | 207 |
| TPA-CN-BN[257] | 504 | 31/0.15 | | 32132 | 102.5/94.1/57.3 | 94.7/80.4/40.3 | 37.9/34.8/21.8 | 8.2 | |
| PTZ-CN-BN[257] | 504 | 31/0.17 | | 17515 | 102.0/74.2/35.9 | 99.4/63.1/23.8 | 33.7/25.2/12.7 | 25.2 | |
| A-BN[258] | 470 | 28 | 0.120,0.142 | 5523 | | | 28.4/15.7/7.0 | 44.7 | 139 |
| DA-BN[258] | 476 | 24 | 0.118, 0.238 | 36717 | | | 35.0/27.0/19.5 | 22.9 | |
| A-DBN[258] | 486 | 22 | 0.117, 0.405 | 36836 | | | 34.3/21.7/13.0 | 36.7 | |
| (R)-SFDBN-CN[259] | 483 | 16 | 0.10, 0.33 | | | 55.8 | 30.0/20.1/15.0 | 33.0 | 209 |
| (S)-SFDBN-CN[259] | 483 | 16 | 0.10, 0.32 | | | 56.1 | 30.7/19.4/15.5 | 36.8 | |
| iPrAuBN[260] | 442 | 19 | 0.154,0.036 | | | | 14.8 | | 210 |
| ICz-BO[261] | 414 | 37 | 0.164,0.031 | 206 | 4.467 | 3.687 | 12.014 | | 211 |
| GBN[262] | 545 | 40 | 0.36,0.62 | 23400 | 122.7 | 120.6 | 31.4/20.9/8.3 | 33.4 | 212 |
| 2FPAB[263] | 436 | 35 | 0.157, 0.044 | 209 | 2.0 | 1.5 | 4.2 | | 213 |
| MePAB[263] | 448 | 29 | 0.146, 0.046 | 973 | 10.7 | 8.4 | 19.8 | | |
| MePABF[263] | 472 | 36 | 0.125, 0.246 | 1408 | 24.1 | 28.4 | 22.8 | | |
| H[6]BN1[264] | 502 | 35 | 0.14,0.55 | 7010 | 5.70 | 6.45 | 3.18 | | 214 |
| HBN[265] | 580 | 24 | 0.54,0.46 | 25946 | 82.63/-/11.47 | 66.53 | 26.7/-/3.7 | | 215 |
| HBN[265] | 581 | 25 | 0.52,0.48 | 40630 | 114.13/-/50.14 | 96.85 | 36.1/-/15.9 | | |
| 2PO[266] | 385 | 29 | | 808 | 6.2 | | 8.8 | | 216 |
| c3PO[266] | 438 | 44 | | 1346 | | | 10.3 | | |
| D2-DBN[267] | 471 | 17 | 0.137,0.176 | | 42.6/-/37.2 | 39.1/-/22.2 | 35.3/-/29.5 | | 217 |
| CNBN[268] | 506 | 16 | 0.12,0.68 | 51020 | 98.3 | 128.7 | 34.4/33.5/23.3 | 2.6 | 218 |
| MCNBN[268] | 517 | 17 | 0.17,0.74 | 64559 | 111.4 | 145.8 | 30.8/30.7/20.4 | 0.32 | |
| PCNBN[268] | 508 | 20 | 0.14,0.67 | 69410 | 85.2 | 95.6 | 29.1/29.0/24.4 | 0.34 | |
| PMCNBN[268] | 519 | 22 | 0.19,0.73 | 71907 | 99.6 | 111.8 | 27.2/27.1/20.3 | 0.36 | |
| DBNDS-TPh[269] | 524.6 | 19.3 | 0.17,0.75 | 36663 | 139 | | 35.2 | | 219 |
| DBNDS-DFPh[269] | 524.6 | 19.9 | 0.19,0.75 | 30115 | 144 | | 36.0 | | |
| DBNDS-CNPh[269] | 524.6 | 19.8 | 0.18,0.75 | 28436 | 120 | | 30.6 | | |
| sym -OBOICz[270] | 468 | 27 | 0.13,0.17 | 51700 | 30.2/-/22.5 | 31.4/-/13.0 | 23.0/-/17.6 | | 220 |
| asym -OBOICz[270] | 472 | 23 | 0.12,0.18 | 76500 | 32.4/-/27.2 | 29.5/-/14.9 | 25.0/-/21.4 | | |
| 5Cz-BNO[271] | 462 | 31 | 0.129,0.110 | | | | 36.3/30.7/21.4 | 15.4 | 221 |
| 5Cz-BN[271] | 499 | 26 | 0.102,0.561 | | | | 37.5/36.2/33.2 | 3.5 | |
| [B-N]N2[272] | 441 | 20 | 0.152,0.046 | 8714 | 13.2 | 13.8 | 20.3 | | 222 |
| [B-N]N3[272] | 459 | 40 | 0.141,0.105 | 19520 | 19.0 | 15.7 | 19.6 | | |
| [B-N]N4[272] | 466 | 30 | 0.134,0.133 | 29960 | 25.6 | 20.1 | 22.1 | | |
| DCzBN-Au[273] | 510 | 34 | 0.16,0.67 | 135310 | 112.5/-/111.8 | 106.8/-/74.0 | 35.8/-/35.7 | | 223 |
| | | | | | | | | | |

[a] current efficiency, power efficiency, external quantum efficiency for maximum, and at 100 cd $m^{-2}$ and 1000 cd $m^{-2}$ [b] Obtained using the equation ((EQEmax-EQE100)/ EQEmax) 100; [c] Maximum value, value at 500 and 1000 cd $m^{-2}$ [d] Maximum value, and value at 1000 and 5000 cd $m^{-2}$;

[1] ITO/NPD (40 nm)/ TCTA (15 nm)/ mCP(15 nm)/1 wt% emitter: mCBP (20 nm)/TSPO1(40 nm)/LiF(1nm)/Al(100 nm); [2] ITO/NPD (40 nm)/ TCTA (15 nm)/ mCP(15 nm)/1 wt% B2: mCBP (20 nm)/TSPO1(40 nm)/LiF(1nm)/Al(100 nm); [3] ITO/MoO3 (2.5 nm)/TAPC (30 nm)/2,6-DCzppy: 4 wt% TBN-TPA (10 nm)/TmPyPB (30 nm)/LiF(1 nm)/Al (100 nm); [4] ITO/PEDOT: PSS(60 nm)/TAPC (20 nm)/mCP (10 nm)/5 wt% t-DABNA: DPEPO: 30wt% DMAC-DPS (25 nm)/TSPO1 (5 nm)/TPBi (20 nm)/LiF (1.5 nm)/Al (200 nm); [5] ITO(50

## **Table S4**. Performance of B/N type MR-emitters in optimized OLEDs

nm)/NPD(40 nm)/TCTA(15 nm)/mCP (15 nm)/1 wt% ν-DABNA: 99 wt% DOBNA-OAr (20 nm)/TSPO1(30 nm)/LiF (1 nm)/Al (100 nm); [6] ITO/HAT-CN (5 nm)/NPD (35 nm)/TCTA (15 nm)/mCP (15 nm)/ 1 wt% ADBNA-Me-Mes or ADBNA-Me-Tip: 99 wt% DOBNA-OAr (20 nm)/TSPO1 (40 nm)/LiF (1 nm)/Al (100 nm); [7] ITO/HAT-CN (10 nm)/NPB (30 nm)/BCzPh (10 nm)/EML (20 nm)/9Cz46Pm (10 nm)/DPPyA: Liq(1: 1, 30 nm)/LiF (0.5 nm)/Al (150 nm), EML: (mCPCB: 35wt% 4TCzBN: 6 wt% TCz-BN), (mCPCB: 35wt% 5TCzBN: 9 wt% 2F-BN or 6 wt% 3F-BN or 6 wt% 4F-BN (20 nm)); [8] ITO/HAT-CN (10 nm)/NPB (30 nm)/BCzPh (10 nm)/ mCBP: 30wt% $Ir(ppy)_3$: 6 wt% AZA-BN (18 nm)/CzPhPy (10 nm)/DPPyA (30 nm)/LiF (0.5 nm)/Al (150 nm); [9] ITO/PEDOT: PSS (40 nm)/ CzAcSF: 2 wt% Cz-BN or BCz-BN (50 nm)/DPEPO (10 nm)/TmPyPB (35 nm)/Liq (1 nm)/Al; [10] ITO/TAPC (50 nm)/TCTA (5 nm)/PhCzBCz: 10 wt% m-Cz-BNCz (30 nm)/TmPyPB (30 nm)/LiF (0.8 nm)/Al (100 nm); [11] ITO/TAPC (50 nm)/TCTA (5 nm)/TCTA: PIM-TRZ (1: 2): 3 wt% DtBuPhCzB (30 nm)/TmPyPB (10 nm)/LiF (1 nm)/Al] (100 nm); [12] ITO)/TAPC (50 nm)/TCTA (5 nm)/mCBP: 1 wt% DtBuPhCzB or BCz-BN (30 nm)/TmPyPB (30 nm)/LiF (1 nm)/Al (100 nm); [13] ITO /Plexcore OC AQ-1200 (50 nm)/polymer A (30 nm)/2 wt% OAB-ABP-1: 98 wt% polymer B (70 nm)/NaF (4 nm)/Al (80 nm); [14] ITO/ HAT-CN (5 nm)/ TAPC (30 nm)/ mCP (10 nm)/1 wt% BN-DMAC or BN-DPAC: mCBP (20 nm)/ PO-T2T (10 nm)/ TmPyPB (40 nm)/ Liq (1.5 nm)/ Al (100 nm); [15] ITO/ HAT-CN (10 nm)/ TAPC (50 nm)/ mCBP (10 nm)/2 wt% BBCz-DB or BCz-BN or BBCz-G or BBCz-Y or BBCz-R: mCBP (20 nm)/ PPF (10 nm)/ B3PyPB (40 nm)/ Liq (1.5 nm)/ Al (100 nm); [16] ITO/HAT-CN (10 nm)/TAPC (60 nm)/TCTA (10 nm)/ CBP: 30wt%Ir(mphmq)2tmd: 3 wt% R-BN or R-TBN (30 nm)/CzPhPy (10 nm)/B4PyMPM(50 nm)/LiF (0.5 nm)/Al (150 nm); [17] ITO (50 nm)/HATCN (7 nm)/TAPC (50 nm)/DCDPA (10 nm)/DBFPO: 3 % emitter (25 nm)/DBFPO (5 nm)/TPBi (20 nm)/LiF (1.5 nm)/Al (100 nm). [18] ITO (45 nm)/Plexcore OC AQ-1200 (35 nm)/polymer A (20 nm)/1 wt % ν-DABNA-Mes: 99 wt % polymer C (70 nm)/NaF (3 nm)/Al (120 nm); [19] ITO (50 nm)/ NPD (40 nm)/TCTA (15 nm)/ mCP (15 nm)/ /1 wt% ν-DABNA-O-Me: 99 wt% DOBNA-Tol (20 nm)/ 3,4-2CzBN (10nm)/ BPy-TP2 (20 nm)/ LiF (1 nm)/Al (100 nm); [20] ITO (50 nm)/NPB (40 nm)/TCTA (15 nm)/mCP (15 nm)/ DOBNA-Tol: 1 % emitter (20 nm)/3,4-2CzBN (10 nm)/ BPyTP2 (20 nm)/LiF (1 nm)/Al (100 nm). [21] ITO/ HATCN (10 nm)/ NPB (30 nm)/ BCzPh (10 nm)/ mCBP: 20 wt% CTPCF3: 1 wt% MR-TADFs (15 nm)/ CzPhPy (10 nm) / DPPyA (30 nm) /LiF (0.5 nm)/Al (150 nm); [22] ITO/HAT-CN (5 nm)/TAPC (30 nm)/TCTA (15 nm)/mCBP (10 nm)/ DMIC-TRZ : 5 wt% emitter (40 nm)/PO-T2T (20 nm)/ANT-BIZ (30 nm)/Liq (2 nm)/Al (100 nm); [23] ITO/HAT-CN (5 nm)/TAPC (30 nm)/mCP (10 nm)/mCBP : 1wt % emitter (20 nm)/POT2T (10 nm)/TmPYPB (40 nm)/ Liq (1.5 nm)/Al (100 nm); [24] ITO/HATCN (6 nm)/TAPC (30 nm)/TCTA (5 nm)/mCP (5 nm)/mCBP: 3 wt% emitters (20 nm)/TmPyPB (40 nm)/LiF (1 nm)/Al; [25] ITO/HAT-CN (10 nm)/TAPC (40 nm)/mCBP (10 nm)/1 wt%-emitter: mCBP or oCBP, (20 nm, oCBP (for γ-Cb-B) or mCBP (for Cz-B, TCz-B, and DACz-B))/PPF (10 nm)/B3PyPB (40 nm)/Liq (1 nm)/Al (100 nm); [26] ITO/ HAT-CN (5 nm)/ TAPC (30 nm)/ mCP (10 nm)/1 wt% emitter: mCBP: PO-T2T (20 nm)/ PO-T2T (10 nm)/ TmPyPB (40 nm)/ Liq(1.5 nm)/ Al (100 nm); [27] ITO (50 nm)/PEDOT: PSS(40 nm) / TAPC (5 nm)/TCTA (5 nm)/PCZAC (5 nm)/ mCP (5 nm)/10 wt% emitter: 99 wt% DPEPO (25 nm)/ TSPO1 (5 nm)/ TPBI (20 nm)/ LiF (1 nm)/Al (200 nm). [28] ITO/HAT-CN (5 nm)/TAPC (30 nm)/TCTA (15 nm)/mCBP (10 nm)/ 3 wt% emitter: 26DCzPPy (25 nm)/POT2T (10 nm)/ANT-BIZ (30 nm)/ Liq (2 nm)/Al (100 nm); [29] ITO/HAT-CN (5 nm)/TAPC (30 nm)/TCTA (15 nm)/mCBP (10 nm)/ 1 wt% emitter: 20 wt% USF: 79 wt%CzSi (25 nm)/POT2T (10 nm)/ANT-BIZ (30 nm)/ Liq (2 nm)/Al (100 nm); 30) HATCN (5 nm)/ TAPC (30 nm)/ TCTA (15 nm)/ mCBP (10 nm)/ 3 wt% emitter: 26DCzPPy (20 nm)/ POT2T (20 nm)/ ANT-BIZ (30 nm)/ Liq (2 nm)/ Al (100 nm); [31] ITO/TAPC (30 nm)/mCP (10 nm)/PPF: 20 wt% BO3N, 30 nm)/PPF (10 nm)/TmPyPB (40 nm)/LiF/Al; [32] ITO /HAT-CN (5 nm)/ TAPC (30 nm)/ mCP (10 nm)/ mCBP: PO-T2T: emitter (49.5: 49.5: 1, 20 nm)/ PO-T2T (10 nm)/ TmPyPB (40 nm)/ Liq (1.5 nm)/ Al (100 nm); [33] ITO (50 nm)/HAT-CN (10 nm)/TAPC (50 nm)/mCBP (10 nm)/2 wt%-BSBS-N1 : mCBP (20 nm)/ PPF (10 nm)/B3PyPB (40 nm)/Liq (1 nm)/Al (100 nm); [34] ITO (50 nm)/HAT-CN (10 nm)/TAPC (40 nm)/mMCP (10 nm)/3 wt%-emitter: mCBP (30 nm)/PPF (10 nm)/B3PyPB (30 nm)/Liq (1 nm)/Al (100 nm); [35] ITO (50 nm)/HAT-CN (10 nm)/NPB (30 nm) TCTA (10 nm)/3 wt% emitter: 30 wt% 3CTF: mCBP (25 nm)/CzPhPy (10 nm)/DPPyA (30 nm)/LiF (0.5 nm)/Al (150 nm); [36] Glass/Ag (100 nm)/ITO (10 nm) HATCN (7 nm)/NPB: HAT-CN (0.3 wt.%) (30 nm)/NPB (120 nm)/TCTA (10 nm)/mCBP: 10 wt.% Ir(ppy)2acac: 3 wt.% BN-ICz-1 (35 nm)/CzPhPy (10 nm)/DPPyA: Liq (25 nm)/Ag (20 nm)/CPL (70 nm); [37] ITO/HATCN (7 nm)/PCBBiF (65 nm)/PCzAc (10 nm)/ DIC-TRz: 20 wt% DMAC-DBP: 0.7 wt% emitter (30 nm)/DDBFT (5 nm)/BPPB (55 nm)/LiF (1.5 nm)/Al (100 nm); [38] ITO/MoO3 (6 nm)/ mCP (50 nm)/30 wt% tCBNDADPO: DBFDPO (25 nm)/DBFDPO (40 nm)/LiF (1 nm)/Al (100 nm); [39] ITO/ TAPC (50 nm)/ TCTA (5 nm)/ 1 wt% DBNO: PhCzBCz (30 nm) / TmPyPB (30 nm)/LiF (1 nm)/Al (100 nm); [40] ITO/TAPC (50 nm)/TCTA (5 nm)/1 wt% DBNO: 15wt% 5TCzBN: PhCzBCz (30 nm)/TmPyPB (30 nm)/LiF (1 nm)/Al (100 nm); [41] ITO (50 nm)/BCFN: HATCN (40 nm, 30 wt%)/BCFN (10 nm)/mCBP (10 nm)/mCBP: mCBP-CN: emitters (30 nm, 50 wt%: 3 wt%)/DBFTRz (5 nm)/ZADN (20 nm)/LiF (1.5 nm)/Al (200 nm); [42] ITO (150 nm)/HTL (70 nm)/EBL (5 nm)/3 wt%t-DABNA-dtB: HOST (20 nm)/HBL (5 nm)/50 wt% ETL: Liq (30 nm)/Liq (1.5 nm)/Al (100 nm); [43] ITO (150 nm)/HTL (75 nm)/EBL (10 nm)/3 wt% t-DABNA-dtB: HOST (20 nm)/HBL (5 nm)/50 wt% ETL: Liq (10 nm)/ 3 wt% Li: nCGL (15 nm)/10 wt% p-doped HTL (10 nm)/HTL (45 nm)/ EBL (10 nm)/3 wt% t-DABNA-dtB: HOST (20 nm)/HBL (5 nm)/50 wt%ETL: Liq (15 nm)/3 wt% Li: nCGL (25 nm)/10 wt% p-doped HTL (10 nm)/ HTL (45 nm)/EBL (10 nm)/3 wt% t-DABNA-dtB: HOST (20 nm)/HBL (5 nm)/50 wt% ETL: Liq (30 nm)/Liq (1 nm)/Al (100 nm); [44] ITO/HAT-CN (10 nm)/TAPC (40 nm)/TCTA (10 nm)/mCBP (8 nm)/mCBP: 2 wt% for SF1BN or 5 wt% for SF3BN (20 nm)/PPF (8 nm)/TmPyPB (35 nm)/Liq (2.5 nm)/Al (60 nm); [45] ITO/HAT-CN (10 nm)/TAPC (40 nm)/TCTA (10 nm)/mCBP (8 nm)/ mCBP: 10 wt% 5CzBN: 2 wt% emitter (20 nm)/PPF (8 nm)/TmPyPB (35 nm)/Liq (2.5 nm)/Al (60 nm); [46] ITO/HATCN (6 nm)/ TAPC (30 nm)/TCTA (5 nm)/mCP (5 nm)/PhCzBCz: 3 wt% emitters (20 nm)/TmPyPB (40 nm)/LiF (1 nm)/Al; [47] HATCN (5 nm)/ TAPC (30 nm)/ TCTA (15 nm)/ mCBP (10 nm)/ DMIC-TRZ: 1 wt% emitters (50 nm)/ POT2T) (20 nm)/ ANT-BIZ (30 nm)/Liq (2 nm)/Al (100 nm); [48] ITO (50 nm)/HATCN (7 nm)/TAPC (50 nm)/DCDPA (10 nm)/mCBP-CN: 3 wt% MRTADF dopant (25 nm)/DBFPO (10 nm)/TPBi (15 nm)/LiF (1.5 nm)/Al (100 nm); [49] ITO/TAPC (50 nm)/TCTA (5 nm)/PhCzBCz: 3 wt% emitter (30 nm)/ TmPyPB (30 nm)/LiF (1 nm)/Al (100 nm); [50] ITO/TAPC (50 nm)/TCTA (5 nm)/ PhCzBCz: 15 wt% sensitizer: 3 wt% emitter (30 nm)/TmPyPB (30 nm)/LiF (1 nm)/Al (100 nm), where the sensitizer and emitter were respectively referred to as 5CzBN and (R)OBN-2CN-BN in device C, and 5tBuCzBN and (R)-OBN-4CNBN in device D; [51] ITO/polymer buffer(PPBI,20 nm)/TAPC (15 nm)/mCBP(5 nm)/ mCBP: 3 wt% emitter (20 nm)/ PO9 (10 nm)/B3PyPB (40 nm)/LiF (0.5 nm)/Al (100 nm); [52] ITO/TAPC (30 nm)/TCTA (10 nm)/CBP: 3 wt% CNCz-BNCz (20 nm)/TmPyPB (50 nm)/LiF (0.8 nm)/Al; [53] ITO/TAPC (30 nm)/TCTA (10 nm)/ CBP: 20 wt% DACT-II: 3 wt% CNCz-BNCz (20 nm)/TmPyPB (50 nm)/LiF (0.8 nm)/Al; [54] ITO/TAPC (30 nm)/TCTA (10 nm)/32alCTRZ: 20 wt% DACT-II: 1 wt% CNCz-BNCz (20 nm)/TmPyPB (50 nm)/LiF (0.8 nm)/Al; [55] ITO/MoO3 (10 nm)/TAPC (60 nm)/mCP (5 nm)/mCP: 3 wt% emitter (20 nm)/DPEPO (15 nm)/TmPyPB (30 nm)/LiF (1 nm)/Al (100 nm); [56] ITO)/HAT-CN (10 nm)/TAPC (40 nm)/mCBP (10 nm)/1 wt%-emitter (Cz-B, TCz-B, and DACz-B: mCBP) or 1-wt%γ-Cb-B: oCBP, 20 nm)/PPF (10 nm)/B3PyPB (40 nm)/Liq (1 nm)/Al (100 nm); [57] ITO/TAPC (50 nm)/TCTA (5 nm)/ PhCzBCz: 3 wt% CNCz-BNCz (20 nm)/TmPyPB (50 nm)/LiF (1 nm)/Al (100 nm); [58] ITO/TAPC (50 nm)/TCTA (5 nm)/PhCzBCz: 15 wt % 5tBuCzBN: 3 wt % emitter (DtCzB-DPTRZ or DtCzB-CNPm ) or 5 wt % emitter (DtCzB-TPTRZ or DtCzB-PPm ) (30 nm)/ TmPyPB (30 nm)/LiF (1 nm)/Al (100 nm); [59] ITO/PEDOT: PSS (35 nm)/ PVK (20 nm)/ mCPCN: emitter (x wt%, 30 nm)/DPEPO (9 nm)/TmPyPB (50 nm)/LiF (0.5 nm)/Al (120 nm), where 3 wt% for (+)-BN4 and (−)-BN4; 1 wt% for (+)-BN5; and 1 wt% (−)-BN5; [60] ITO)-coated glass/HAT-CN (10 nm)/Tris-PCz (30 nm)/ mCBP (5 nm)/mCBP: 20 wt% TPh2Cz2DPhCzBN: 0.5 wt% emitters (30 nm)/SF3-TRZ (10 nm)/SF3-TRZ: 30 wt% Liq (20 nm)/ Liq (2 nm)/Al (100 nm); [61] ITO/HATCN (10 nm)/TAPC (40 nm)/mCP (5 nm)/mCBP: 5 wt% TPXZBN or DPXZCZBN (20 nm)/TmPyPB (50 nm)/LiF (1 nm)/Al (100 nm); [62] ITO/TAPC (50 nm)/TCTA (5 nm)/ mCBP: 5 wt% emitter (30 nm)/TmPyPB (45 nm)/LiF (1 nm)/Al (100 nm); [63] ITO/NPB (30 nm)/TCTA (10 nm)/mCP(10 nm)/mCBP: 20 wt% TCTPCF3: 2 wt% BN1 or TCz-BN1 (20 nm)/ CzPhPy (10 nm)/ DPPyA(10 nm)/ LiF (1 nm)/Al(100 nm); [64] ITO/NPB (30 nm)/TCTA (10 nm)/mCP(10 nm)/mCBP: 20 wt% DACT-II: 5 wt% BN2 or TCz-BN2 (20 nm)/ CzPhPy (10 nm)/ DPPyA(10 nm)/ LiF (1 nm)/Al(100 nm); [65] ITO/ HATCN (1 nm)/TAPC (25 nm)/TCTA (10 nm)/ mCP (10 nm)/ mCP: DPEPO: 5 wt% emitter (25 nm)/TmPyPB (40 nm)/LiF (1 nm)/Al (100 nm); [66] ITO/ HAT-CN (5 nm)/ TAPC (40 nm)/ mCP (5 nm)/ mCBP: 4 wt.% emitter (20 nm)/ TmPyPB (45 nm)/ LiF (1 nm)/ Al (100 nm); [67] ITO/ PEDOT: PSS (40 nm)/ mCP: 5 wt.% emitter (30 nm)/ TSPO1 (8 nm) / TmPyPB (42 nm)/ LiF (1 nm)/ Al (100 nm); [68] ITO/ HAT-CN (5 nm)/ TAPC (30 nm)/ TCTA (15 nm)/ mCBP (10 nm)/ DMIC-TRZ: 1 wt % emitter (40 nm)/ POT2T (20 nm) /ANT-BIZ (30 nm)/ Liq (1 nm)/ Al (100 nm); [69] ITO/HATCN (5 nm)/TAPC (30 nm)/TCTA (15 nm)/mCBP (10 nm)/DBFPO: 25 wt% 3Cz2BN: 1 wt% emitter (25 nm)/DBFPO (15 nm)/ANT-BIZ (30 nm)/Liq/ Al; [70] ITO/TAPC (50 nm)/TCTA (5 nm)/PhCzBCz: 3 wt% BNTP (30 nm)/TmPyPB (30 nm)/LiF (1 nm)/Al (100 nm)]; [71] ITO (50 nm)/ HATCN (5 nm)/TAPC (30 nm)/TCTA (15 nm)/mCBP (10 nm)/ 2,6-DCzppy: 2 wt% emitter (15 nm)/PO-T2T (20 nm)/ANT-BIZ (30 nm)/Liq (2 nm)/ Al; [72] ITO (50 nm)/ HATCN (5 nm)/TAPC (30 nm)/TCTA (15 nm)/mCBP (10 nm)/ 79 wt% CzSi: 20 wt% USF: 1 wt% emitter (15 nm)/PO-T2T (20 nm)/ANT-BIZ (30 nm)/Liq (2 nm)/ Al; [73] ITO/HAT-CN (5 nm)/TAPC (30 nm)/ TCTA (15 nm)/mCBP (10 nm)/ 79 wt% DMIC-TRZ: 20 wt% PO-01: 1 wt% emitter (15 nm)/PO-T2T (20 nm)/ANTBIZ (30 nm)/Liq (2 nm)/Al (100 nm); [74] ITO/50 nm)/HAT-CN (10 nm)/TAPC (40 nm)/mMCP (5 nm)/ 1 wt%-emitter: mCBP (30 nm)/PPF (5 nm)/B3PyPB (40 nm)/Liq (1 nm)/Al (100 nm); [75] Device 74) equipped with a microlens array film to improve light out-coupling equipped with a microlens array film to improve light out-coupling; [76] ITO/ MoO3 (10 nm)/TAPC (60 nm)/mCP (5 nm)/DPEPO: 30 wt% DMACDPS: 1 wt% emitters (20 nm)/DPEPO (5 nm)/TPBi (40 nm)/LiF (1 nm)/Al (100 nm); [77] ITO/50 nm)/HAT-CN (5 nm)/TAPC (40 nm)/mCBP (5 nm)/ 4 wt%-emitter: mCBP (20 nm)/ / TmPyPB (45 nm)/Liq (1 nm)/Al (100 nm); [78] ITO/HATCN (5 nm)/NPB (30 nm)/TCTA (10 nm)/mCPBC: 4TCzBN: 2 wt% emitters (EML, 24 nm)/CzPhPy (10 nm)/DPPyA (20 nm)/LiF (0.5 nm)/Al (150 nm); [79] ITO/TAPC (30 nm)/TCTA (5 nm)/mCP (5 nm)/EMLs (4TCzPhBN, p4TCzPhBN and 3CTF were chosen as the sensitizer for BIC-mCz and mDBIC, BIC-pCz and pDBIC, respectively. 20 nm)/PPF (5 nm)/Bphen (30 nm) /LiF (0.5 nm)/Al (150 nm); [80] ITO(50 nm)/NPD(40 nm)/TCTA(15 nm)/mCP (15 nm)/0.5 wt% emitter: 99.5 wt% DOBNA-Ph (20 nm)/3,4-2CzBN (10 nm)/BPy-TP2(20 nm)/LiF (1 nm)/Al (100 nm); [81] ITO/PEDOT: PSS (40 nm)/ CzAcSF: 2 wt% emitter (EML, 50 nm)/DPEPO (10 nm)/TmPyPB (35 nm)/Liq (1 nm)/Al; [82] ITO/ HATCN (5 nm)/TAPC (60 nm)/ mCP (10 nm)/ mCBP: 20 wt% (MAC*)Cu(Cz): 1 wt% BN3 (25 nm)/ TPBi (10 nm)/ 3TPYMB (50 nm)/ Liq (1.5 nm)/Al (100 nm); [83] ITO/ HATCN (5 nm)/TAPC (60 nm)/ mCP (10 nm)/ mCBP: 20 wt% PXZMePM: 1 wt% BN3 (25 nm)/ TPBi (10 nm)/ 3TPYMB (50 nm)/Liq (1.5 nm)/Al (100 nm); [84] ITO/HATCN (5 nm)/TAPC (30 nm)/mCP (10 nm)/R-CzOBN: POT2T: BN1 (35 nm)/POT2T (10 nm)/TmPyPB(40 nm)/Liq (1.5 nm)/Al (100 nm); [85] ITO/HATCN (5 nm)/TAPC (30 nm)/mCP (10 nm)/S-CzOBN: POT2T: BN1 (35 nm)/POT2T (10 nm)/TmPyPB(40 nm)/Liq (1.5 nm)/Al (100 nm); [86] ITO (50 nm)/HATCN (7 nm)/PCBBiF (68 nm)/ p-PPDF: 5 wt% DABNA-NP-TB (25 nm)/BPPB (30 nm)/LiF (1.5 nm)/Al (100 nm); [87] ITO (50 nm)/HATCN (7 nm)/PCBBiF (68 nm)/ m-PPDF: 5 wt% DABNA-NP-TB (25 nm)/BPPB (30 nm)/LiF (1.5 nm)/Al (100 nm); [88] ITO)/HATCN (10 nm)/TAPC (45 nm)/chiral emitters: 2,6-DCzPPy (5 wt%, 20 nm)/TmPyPB (50 nm)/LiF (1 nm)/Al (100 nm); [89] ITO/ HAT-CN, (5 nm)/ TAPC (30 nm)/ (TCTA, 15 nm)/ mCBP (,10 nm)/ 1 wt% emitter: DMIC-TRZ(50 nm)/ POT2T (20 nm)/ ANT-BIZ (30 nm)/ Liq (2 nm)/ Al (100 nm); [90] ITO/HAT-CN (5 nm)/TAPC (30 nm)/ TCTA (15 nm)/mCBP (10 nm)/EML (1 wt% BN3 and 25 wt% BNSeSe inDMIC-TRZ, 50 nm)/POT2T (20 nm)/ANT-BIZ (30 nm)/Liq (2 nm)/Al (100 nm); [91] ITO/MoO3 (3 nm)/ NPB (30 nm)/ TCTA (10 nm)/ mCP (5 nm)/mCPBP: 10 wt % CTPCF3 as sensitizers: 1 wt % 2F-BN (15 nm)/BPBiPA (30 nm)/LiF (0.5 nm)/Al (150 nm); [92] ITO/MoO3 (3 nm)/ NPB (30 nm)/ TCTA (10 nm)/ mCP (5 nm)/mCPBP: 10 wt % CNCTPCF3 as sensitizers: 1 wt % 2F-BN (15 nm)/BPBiPA (30 nm)/LiF (0.5 nm)/Al (150 nm); [93] ITO/MoO3 (3 nm)/ NPB (30 nm)/ TCTA (10 nm)/ mCP (5 nm)/mCPBP: 10 wt % TCTPCF3 as sensitizers: 1 wt % 2F-BN (15 nm)/BPBiPA (30 nm)/LiF (0.5 nm)/Al (150 nm); [94] ITO/HAT-CN (15 nm)/TAPC (70 nm)/TPSS: emitter (2 wt%, 30 nm)/Bepp2 (5 nm)/Bepp2: Liq (50 wt%, 30 nm)/Liq (1 nm)/Al; [95] ITO/HAT-CN (15 nm)/TAPC (70 nm)/ BCz-o-TRZ: 5 wt% Ir(ppy)3: 2 wt% tCzphBs (30 nm)/Bepp2 (5 nm)/Bepp2: Liq (50 wt%, 30 nm)/Liq (1 nm)/Al; [96] ITO /TAPC (30 nm)/TCTA (10 nm)/emitting layer (5 wt% DtBuCzB or TCzBN-DPF in SF3-TRZ, or 1 wt% TCzBN-TMPh or TCzBN-oPh in SF3-TRZ, 20 nm)/TmPyPB (40 nm)/LiF (1 nm)/Al; [97] ITO (50 nm)/ HATCN (7 nm)/TAPC (50 nm)/DCDPA (10 nm)/DBFPO: 30% pMDBA-DI: 5% of t-Bu-ν-DABNA (25 nm)/ DBFPO (5 nm)/TPBi (20 nm)/LiF (1.5 nm)/Al (100 nm); [98] ITO (50 nm)/ HATCN (7 nm)/TAPC (50 nm)/DCDPA (10 nm)/DBFPO: 30% mMDBA-DI: 5% of t-Bu-ν-DABNA (25 nm)/ DBFPO (5 nm)/TPBi (20 nm)/LiF (1.5 nm)/Al (100 nm);[99] ITO/HAT-CN (10 nm)/ FSFA (120 nm)/FSF4A (5 nm)/ DMIC-Cz: DMIC-TRz: emitter (0.5wt% (SIPr)AuBN,or 1wt% (BzIPr)AuBN or 1wt% (PyIPr)AuBN or 4wt% (PzIPr)AuBN for emitter, 30 nm)/ANT-BIZ (5 nm)/ ANT-BIZ: Liq (1: 1, 25 nm)/Liq (2 nm)/Al (100 nm); [100] ITO/ HAT-CN (10 nm)/BCFN(60 nm)/SiCzCz (5 nm)/65% SiCzCz: 35%SiTrzCz2 + 13% PtON-TBBI +0.4% emitter (EML, 30 nm)/2SiTrzPh (5 nm)/2SiTrzPh: Liq (5: 5, 31 nm)/LiF (1.5 nm)/Al (80 nm); [101] ITO/ TAPC (30 nm)/ TCTA (10 nm)/ CBP: 3wt% emitter(EML, 20 nm)/ (TmPyPB, 50 nm)/LiF (0.8 nm)/Al (100 nm); [102] ITO/ HAT-CN (5 nm)/ TAPC (45 nm)/ mCP (5 nm)/ 2,6-Dczppy: 4 wt% emitters (20 nm)/ TmPyPB (50 nm)/ LiF (1 nm)/Al (100 nm); [103] ITO/ PEDOT: PSS (40 nm)/5CzBN-ESF sensitizer: 2 wt% S-Cz-BN/ POT2T (40 nm)/Cs2CO3 (1 nm)/Al (100 nm); [104] ITO/PEDOT: PSS/mCP: PCzbBNx/ DPEPO (10 nm)/TmPyPB (40 nm)/LiF(1 nm)/Al (100 nm); [105] ITO/ TAPC (50 nm)/ TCTA (5 nm)/ mCBP: 10 wt% emitter (30 nm)/ TSPO1 (5 nm)/ TmPyPB (45 nm)/LiF (1 nm)/Al (100 nm)； [106] ITO/TAPC (50 nm)/TCTA (5 nm)/PhCzBCz: 10 wt% emitter (30 nm)/TmPyPB (30 nm)/LiF (1 nm)/Al (100 nm); [107] ITO/ 70 nm/ NPB (80 nm)/ TCTA (20 nm)/ mCP (30 nm)/DPEPO: emitter (10 wt%, 20 nm)/ TPBi(30 nm)/LiF (1 nm)/Al (100 nm); [108] ITO /TAPC (30 nm)/ TCTA (10 nm)/ mCP (10 nm)/ PhCzBCz: 8 wt% TCZ-F-DABNA(20 nm)/ TmPyPB (40 nm)/LiF (1 nm)/Al (120 nm); [109] ITO)/ PEDOT: PSS (40 nm)/ H2: 10 wt% R-D2: 1 wt% emitter (30 nm)/ B3PyMPM (60 nm)/LiF (1nm)/Al (150 nm)； [110] ITO (50 nm)/HATCN (7 nm)/TAPC (50 nm)/DCDPA (10 nm)/mCBP-CN: 3% mICz-DABNA and BFCz-DABNA (25 nm)/DBFPO (10 nm)/TPBi (15 nm)/LiF (1.5 nm)/Al (100 nm); [111] ITO/ HATCN (10 nm)/ NPB (30 nm)/ BCzPh (10 nm) / mCBP: 20 wt % p4TzPhBN: 4 wt % C-BN (30 nm)/ CzPhPy (10 nm)/ DPPyA (30 nm)/LiF (0.5 nm)/Al (150 nm); [112] ITO /TAPC (50nm)/TCTA (5 nm)/mCBP: 5 wt % emitter (30 nm)/TSPO1 (5 nm)/TmPyPB (45 nm)/LiF (1nm)/Al (150 nm); [113] ITO ( 70 nm)/ MoO3 (1 nm)/ TAPC (25 nm)/ TCTA (10 nm)/mCBP (10 nm)/ mCBP: DPEPO: 5 wt% pBP-DABNA-Me (25 nm) /DPEPO (5 nm)/ TmPyPB (35 nm)/LiF (1 nm)/Al (100 nm); [114] ITO ( 70 nm)/ MoO3 (1 nm)/ TAPC (25 nm)/ TCTA (10 nm)/mCBP (10 nm)/ mCBP: DPEPO: TDBA-SAF: 5 wt% pBP-DABNA-Me (25 nm) /DPEPO (5 nm)/ TmPyPB (35 nm)/LiF (1 nm)/Al (100 nm); [115] ITO/ HAT-CN (5 nm)/TAPC (30 nm)/TCTA (15 nm)/mCBP (10 nm)/1 wt % BN-Se in DMIC-TRZ (40 nm)/PO-T2T (20 nm)/ANT-BIZ (30 nm)/Liq (2 nm)/Al (100 nm); [116] ITO/ HATCN (5 nm)/ TAPC (45 nm)/ (R)-Czp-tBuCzB (5 wt%) or (R)-Czp-POAB (8 wt%): 2,6DCzPPy (20 nm)/ TmPyPB (45 nm)/ LiF (1 nm)/ Al (100 nm); [117] ITO/ HATCN (10 nm)/ NPB (30 nm)/ TCTA (10 nm)/ mCBP: 30wt% 3CTF: 3 wt% emitter (EML. 25 nm)/ CzPhPy (10 nm)/ DPPyA (30 nm)/LiF (0. 5 nm)/Al ( 150 nm); [118] ITO/PEDOT: PSS: PFI (35 nm)/ vNPB (20 nm)/ 1wt% BN-R: NPB: DMFBD-TRZ (35 nm)/ DMFBD-TRZ (10 nm)/Liq (2 nm)/Al (100 nm); [119] ITO/ MoO3 (10 nm)/ TAPC (60 nm)/ mCP (10 nm)/ PPF: 20 wt% 3tPAB: 5 wt% PhDMAC-BN (20 nm)/ PPF (10 nm)/ TmPyPB (30 nm)/ LiF (1 nm)/ Al (100 nm); [120] ITO/ HATCN (5 nm)/ TAPC (45 nm)/ mCP (5 nm)/ mCBP: 5 wt% LTCz-BN (20 nm)/ TmPyPB (45 nm)/ LiF (1 nm)/ Al (100 nm); [121] ITO/ NPD (40 nm)/ TCTA (15 nm)/ mCP (15 nm)/ 0.5 wt % ω-DABNA emitter: 99.5 wt % DOBNA-Ph (20 nm)/ 3,4-2CzBN (10 nm)/ BPy-TP2 (20 nm), LiF (1 nm)/ Al (100 nm); [122] ITO/ HATCN (5 nm)/ TAPC (45 nm)/ mCP (5 nm)/ 2,6-DCzppy: 8 wt% p-1-PCzBN or 2 wt% m-1-PCzBN (x wt%, 20 nm)/ TmPyPB(45 nm)/LiF (1 nm)/Al (100 nm); [123] ITO ( 70 nm)/ TAPC (35 nm)/ TCTA (8 nm)/26DCzPPy: 3 wt% emitter (20 nm) / TmPyPB (50 nm)/LiF (0.8 nm)/Al (100 nm; [124] ITO/HATCN (10 nm)/TAPC (40 nm)/TCTA (10 nm)/ mCP (10 nm)/5 wt% emitter:

**Table S4**. Performance of B/N type MR-emitters in optimized OLEDs

mCBP (20 nm)/PPF (10 nm)/TmPyPB (40 nm)/LiF (1 nm)/Al (100 nm); [125] ITO/ PEDOT: PSS (50 nm)/ PVK (15 nm)/ mCP: 20 wt% 5CzTRZ: 2 wt% emitter (30 nm)/ mSiTRZ (12 nm)/ TmPPPyTz (55 nm)/LiF (1 nm)/Al (150 nm); [126] ITO/ PEDOT: PSS (50 nm)/ PVK (15 nm)/ mCP: 20 wt%5tBuCzTRZ: 2 wt% emitter (30 nm)/ mSiTRZ (12 nm)/ TmPPPyTz (55 nm)/LiF (1 nm)/Al (150 nm); [127] ITO/TAPC (40 nm)/TCTA (10 nm)/SF3-TRZ: 15 wt% DPMX-CzDABNA (20 nm)/TmPyPB(40 nm)/LiF (1 nm)/Al; [128] ITO/TAPC (35 nm)/TCTA (10 nm)/ SF3-TRZ: 15 wt% DPMX-CzDABNA: 0.4 wt%BPPZ-DPXZ (20 nm)/ TmPyPB(45 nm)/LiF(1 nm)/Al; [129] ITO/m-PEDOT: PSS (35 nm)/ 3CzAcPy: 1 wt% BN-CP1 (50 nm)/DPEPO (10 nm)/TmPyPB (50 nm)/Liq (1 nm)/Al (100 nm);[130] ITO/m-PEDOT: PSS (35 nm)/ 9CzAcPy: 1 wt% BN-CP1 (50 nm)/DPEPO (10 nm)/TmPyPB (50 nm)/Liq (1 nm)/Al (100 nm); [131] ITO/ HAT-CN (5 nm)/ TAPC (30 nm)/ TCTA (15 nm)/ mCBP (10 nm)/EML (5 wt% emitters in DMICTRZ, 50 nm)/ POT2T (20 nm)/ ANT-BIZ (30 nm)/ Liq (2 nm)/ Al (100 nm); [132] ITO/ HAT-CN (5 nm)/ TAPC (30 nm)/ TCTA (15 nm)/ mCBP (10 nm)/EML (1 wt% emitters : 25 wt% 5TBuCzBN: 74 wt% DMICTRZ, 50 nm)/ POT2T (20 nm)/ ANT-BIZ (30 nm)/ Liq (2 nm)/ Al (100 nm); [133] ITO/ HAT-CN (10 nm)/ NPB (30 nm)/ TCTA (10 nm)/ /EML (DMIC-TRZ: 8 wt% Ir(piq)2acac: 1.0 wt% BNNO, 24 nm)/ CzPhPy (10 nm)/ DPPyA (30 nm)/ LiF (0.7 nm)/ Al (150 nm); [134] ITO/HATCN (5 nm)/NPB (30 nm)/ TCTA (10 nm)/emitting layers (where the material mCPBC was selected as a host, meanwhile m4TCzPhBN, 5TCzBN, 3CTF, and DACTII were successively selected as sensitizers for [B-N]N, p[B-N]O, p[B-N]NO and p[B-N]N, respectively, 24 nm)/CzPhPy (15 nm)/DPPyA (30 nm)/LiF (0.5 nm)/Al (150 nm); [135] ITO/TAPC (30 nm)/ TCTA (10 nm)/SF3-TRZ: 2.5 wt% DBTN-2 (20 nm)/ TmPyPB (40 nm)/LiF (1 nm)/Al; [136] ITO (anode)/NPB (HTL, 40 nm)/TSBPA (EBL, 10 nm)/α-3BNMes: DtBuAcDBT: DPEPO 1: 25: 74% (EML, 30 nm)/DPEPO (HBL, 10 nm)/TBPi (ETL, 40 nm)/LiF (EIL, 1 nm) /Al (cathode, 100 nm); [137] ITO/HAT-CN (5 nm)/ NPB (40 nm)/TCTA (10 nm)/CzSi (10 nm)/NOBNacene (3 wt%): TSPO1 (20 nm)/TSPO1 (10 nm)/TmPyPB (20 nm)/LiF (0.8 nm)/Al (100 nm); [138] ITO/HAT-CN (10 nm)/TAPC (50 nm)/mMCP (5 nm)/1 wt%-emitter: mCBP-CN (30 nm)/PPF (5 nm)/B3PyPB (50 nm)/Liq (1 nm)/Al (100 nm); [139] ITO/HAT-CN (10 nm)/TAPC (40 nm)/TCTA (10 nm)/x wt%-emitter (BN-PhOH, BN-PhOCH3, and BNPhN(CH3)2 under the optimized doping concentration of 1 wt%, 3 wt% and 3 wt%, respectively): mCBP (30 nm)/TmPyPB (40 nm)/Liq (2 nm)/Al (120 nm); [140] ITO)/ HATCN (5 nm)/ NPB (30 nm)/ BCzPh (10 nm)/mCPBC: 4TCzBN: x wt% emitters (EML, 24 nm)/ CzPhPy (15 nm)/ DPPyA (30 nm)/LiF (0.5 nm)/Al (150 nm); [141] ITO)/ TAPC (35 nm)/ TCTA (10 nm)/ mCP (10 nm)/DPEPO: 1 wt% BN4 (30 nm)/DPEPO (10 nm)/ TPBi (45 nm)/LiF (0.8 nm)/Al (100 nm); [142] ITO/ polymer buffer (20 nm)/ TAPC (15 nm)/mCBP (5 nm)/ 3 wt% emitter: mCBP (20 nm)/ PO9 (10 nm)/ B3PyPB (40 nm)/LiF (0.5 nm)/Al (100 nm); [143] ITO (50 nm)/HATCN (7 nm)/TAPC (50 nm)/DCDPA (10 nm)/DBFPO: 5wt% emitter (25 nm)/DBFPO (5 nm)/TPBi (20 nm)/LiF (1.5 nm)/Al (100 nm); [144] ITO (50 nm)/HATCN (5 nm)/TAPC (30 nm)/TCTA (15 nm)/mCBP (10 nm)/ DMIC-TRZ: 5wt% emitter (25 nm)/ PO-T2T (15 nm)/ANT-BIZ(30 nm)/Liq (2 nm)/Al (100 nm); [145] ITO (50 nm)/HATCN (5 nm)/TAPC (30 nm)/TCTA (15 nm)/mCBP (10 nm)/74 wt% DBFPO: 25 wt% mMDBA-DI: 1wt% emitter (25 nm)/ DBFPO (15 nm)/ANT-BIZ(30 nm)/Liq (2 nm)/Al (100 nm); [146] ITO (50 nm)/HATCN (10 nm)/ TAPC ( 60 nm)/ mCBP (10 nm)/mCBP: x wt% emitters (5 wt% for BN-TC, and15 wt% forBN-AC, BN-PXZ and BN-PZ, 20 nm)/ TmPyPB (45 nm)/LiF (1 nm)/Al (100 nm); [147] ITO (70 nm)/ MoO3 ( 1 nm)/ TAPC (30 nm)/ TCTA (10 nm)/mCBP (10 nm)/mCBP: DPEPO: MR-emitter (50: 50: 2 wt %, 25 nm)/ DPEPO (5 nm)/ TmPyPB (40 nm)/LiF (1 nm)/Al (100 nm); [148] ITO (70 nm)/ MoO3 ( 1 nm)/ TAPC (30 nm)/ TCTA (10 nm)/mCBP (10 nm)/mCBP: DPEPO: TDBA-SAF: MR-emitter (50: 50: 20: 2wt %, 25 nm)/ DPEPO (5 nm)/ TmPyPB (40 nm)/LiF (1 nm)/Al (100 nm); [149] ITO (50 nm)/ TAPC (50 nm)/TCTA (5 nm)/ PhCzBCz: 1wt% m-DBCz (25 nm)/ TmPyPB (30 nm)//LiF (1 nm)/Al (100 nm); [150] emitter layer: (traditional PhCzBCz host is replaced by a reported TADF emitter DMIC-TRZ in device 149)); [151] emitter layer: (1 wt% m-DBCz: 20 wt% $Ir(ppy)_3$: PhCzBCz); [152] emitter layer: (1 wt% m-DBCz: 20 wt% Ir(ppy)3: DMIC-TRZ); [153] ITO/TAPC (50 nm)/TCTA (5 nm)/PhCbBCz: x wt% (P or M)-BN-Py (30 nm)/TmPyPB (30 nm)/LiF (1 nm)/Al (100 nm); [154] ITO/ MoO3 (6 nm)/mCP (50 nm)/DBFDPO: 10% emitters (25 nm)/DBFDPO (40 nm)/LiF (1 nm)/Al (100 nm); 154)ITO/HATCN (5 nm)/NPB (30 nm)/ BCzPh (10 nm)/α,β-ADN: 0.8 wt% emitters (30 nm)/CzPhPy (10 nm)/ DPPyA (20 nm)/LiF (0.5 nm)/Al (150 nm); [155] ITO/TAPC (30 nm)/TCTA (10 nm)/mCBP (10 nm)/ DMIC-TRZ: 10 wt% Bt2Ir(acac) 1 wt% B4N6-Me (20 nm)/PO-T2T (10 nm)/TmPyPB (30 nm)/ LiF (1 nm)/Al (150 nm); [156] ITO/ HATCN (5 nm)/NPB (30 nm)/ SFBCz (10 nm)/ SFBCz: SFTRZ: 15 wt% Ir(mphmq)2tmd: 1.0 wt% emitter (EML, 30 nm)/ SFTRZ (10 nm)/ DPPyA (30 nm)/ LiF (0.5 nm)/ Al (150 nm); [157] ITO/ PEDOT: PSS (40 nm)/polyvinylcarbazole (PVK)/mCP: 10 wt% 1-BNN, D2-BNN or 20 wt% D3-BNN/mSiTRZ (8 nm)/TmPPPyTz (42 nm)/LiF (1 nm)/Al (100 nm); [158] ITO/HATCN (5 nm)/TAPC (30 nm)/TCTA (15 nm)/mCBP (10 nm)/ DMIC-TRZ: 1 wt% (20 nm)/PO-T2T (20 nm)/ANT-BIZ (30 nm)/Liq(2 nm)/Al (100 nm); [159] ITO /HAT-CN (5 nm)/TAPC (30 nm)/TCTA (15 nm)/mCBP (10 nm)/ 1 wt% NasBN: 10 wt% Ir(tfmppy)2(mppy): 89 wt% DMIC-TRZ and 1 wt%Na-dBN: 10 wt% (mtfmptp)2Ir(mmND): 89 wt% DMIC-TRZ (30 nm)/POT2T (20nm)/ANT-BIZ (30 nm)/Liq (2 nm)/Al (100 nm); [160] ITO/ (PEDOT: PSS) (50 nm)/ PVK (15 nm)/mCP: 20 wt.% 5CzTRZ: 1.5 wt.% emitter (30 nm)/ mSiTRZ (12 nm)/ TmPPPyTz (55 nm)/LiF(1 nm)/Al (150 nm); [161] [ITO/TAPC (50 nm)/TCTA (5 nm)/PhCzBCz: 3 wt% emitter (30 nm)/TmPyPB (30 nm)/LiF (1 nm)/Al (100 nm); [162] ITO /HAT-CN (5 nm)/NPB (30 nm)/ BCzPh (10 nm)/ CBP: 20 wt% PO-01: 0.5 wt% PPZ-BN/CzPhPy (5 nm)/ DPPyA (30 nm)/ LiF (0.5 nm)/Al (150 nm); [163] ITO/ HATCN (5 nm)/ NPB (30 nm)/ TCTA (10 nm)/mCPBC: 30 wt.% 5TCzBN: 1 wt.% emitters (EML, 30 nm)/ CzPhPy (10 nm)/ DPPyA (20 nm)/LiF (0.5 nm)/Al (150 nm); [164] ITO/TAPC (35 nm)/ TCTA (10 nm)/mCBP: 25 wt% 4TCzBN: 3 wt% emitter (20 nm)/ TmPyPB (50 nm)/LiF (1 nm)/Al (100 nm); [165] ITO)/HAT-CN (5 nm)/TAPC (30 nm)/TCTA (15 nm)/mCBP (10 nm)/ 79 wt% DMIC-TRZ: 20 wt% Pt-1: 1 wt% emitters /PO-T2T (20 nm)/ANT-BIZ (30 nm)/ Liq (2 nm)/Al (100 nm); [166] ITO/TAPC (50 nm)/TCTA (5 nm)/mCBP: 5 wt% emitter (30 nm)/TSPO1 (5 nm)/TmPyPB (45 nm)/LiF (1 nm)/Al (100 nm); [167] ITO/ HAT-CN (5 nm)/ TAPC (30 nm)/ TCTA (15 nm)/ mCBP (10 nm)/DOBNA-OAr: 2 wt% DB or DB-O/DB-S enantiomers (15 nm)/DBFPO (20 nm)/ ANT-BIZ (30 nm)/ Liq (0.5 nm)/Al; [168] ITO/ PEDOT: PSS (40 nm) / PhCzBCz: 10 wt% 4CzIPN: 1.6 wt% BNB′-1 (EMLs, 20 nm)/ TmPyPB (40 nm)/LiF (1 nm) / Al (120 nm); [169] ITO / HAT-CN (10 nm)/ TAPC (30 nm)/ TCTA (10 nm)/ PhCzBCz: 10 wt% 4CzIPN: 1.6 wt% BNB′-1 (EML, 20 nm)/TmPyPB (40 nm)/LiF (1 nm)/Al (120 nm); [170] ITO/TAPC (30 nm)/TCTA (10 nm)/PhCzBCz: 3 wt.% emitters (30 nm)/TmPyPB (40 nm)/LiF (1 nm)/Al (120 nm); [171] ITO/TAPC (30 nm)/mCP (10 nm)/ DPEPO: 30 wt.% DspiroAc-TRZ: 1 wt.% emitters (30 nm)/DPEPO (10 nm)/TmPyPB (40 nm)/LiF (1 nm)/Al (150 nm); [172] ITO /HAT-CN (7 nm)/TAPC ( 30 nm)/TCTA (10 nm)/mCBP (10 nm)/ DMICTRZ: 1 wt% emitter (20 nm)/TmPyPB (40 nm)/LiF (1 nm)/Al; [173] ITO/HATCN (10 nm)/NPB (30 nm)/BCzPh (10 nm)/mCBP: 20 wt% PO-01: 1wt% Py-Cz-BN (30 nm)/CzPhPy (10 nm)/DPPyA (30 nm)/LiF (0.5 nm)/Al (150 nm); [174] ITO)/HAT-CN (5 nm)/ TAPC (30 nm)/TCTA (15 nm)/mCBP (10 nm)/ DMIC-TRZ: 5wt% emitter/POT2T (20 nm)/ANT-BIZ (30 nm)/Liq (2 nm)/Al (100 nm); [175] ITO /HAT-CN (5nm)/ TAPC (30nm)/ TCTA (15 nm)/ mCBP (10 nm)/ DMIC-TRZ: 2 wt.% emitter (15 nm)/ PO-T2T (20 nm)/ ANT-BIZ (20 nm)/ Liq (2 nm)/ Al (100 nm); [176] ITO)/HAT-CN (5 nm)/ TAPC (30 nm)/TCTA (15 nm)/mCBP (10 nm)/ DMIC-TRZ: $Ir(ppy)_3$: 5wt% emitter/POT2T (20 nm)/ANT-BIZ (30 nm)/Liq (2 nm)/Al (100 nm); [177] ITO)/HAT-CN (5 nm)/ TAPC (30 nm)/TCTA (15 nm)/mCBP (10 nm)/ DBFPO mMDBA-DI: 1wt% emitter/DBFPO (20 nm)/ANT-BIZ (30 nm)/Liq (2 nm)/Al (100 nm); [178] ITO/ HATCN (5 nm)/ NPB (30 nm)/ TCTA (10 nm)/ mCBP: 30 wt% 3CTF: 1 wt% TPABO-DICz (30 nm)/ CzPhPy (10 nm)/ DPPyA (30 nm)/ LiF (0.5 nm)/Al (150 nm); [179] ITO/HATCN(20 nm)/BPBPA(50 nm)/PPMF-SF(10 nm)/ANDN: emitter (20 nm, 3 wt%)/Na-An-Bi: Liq(35 nm)/ Yb(1 nm)/Ag(100 nm); 1[80] ITO /TAPC (50 nm)/TCTA (5 nm)/PhCzBCz: 25 wt % DACT-II: 3 wt % BN-Cz or BN-Cb (30 nm)/TmPyPB (30 nm)/LiF (1 nm)/Al (100 nm); [181] ITO/TAPC (50 nm)/TCTA (5 nm)/10 wt% emitter in PhCzBCz (30 nm)/TmPyPB (30 nm)/Liq (1 nm)/Al (100 nm); [182] ITO/PEDOT: PSS (40 nm)/ x3Cz2BN: emitter(3Cz2BN is 50 wt% for PCzDBN1, 70 wt% for PCzDBN3 and 30 wt% for PCzDBN5 40 nm)/PPF (10 nm)/TmPyPB (40 nm)/Liq (1 nm)/Al (100 nm); [183] ITO/PEDOT: PSS (40 nm)/DMIC-TRZ: emitters (40 nm)/PO-T2T (20 nm)/ ANT-BIZ (30 nm)/Liq (1 nm)/Al (100 nm); [184] ITO/ (70 nm)/MoO3(1 nm)/TAPC (30 nm)/ TCTA ( 10 nm)/ SiCzCz (10 nm)/SiCzCz: SiTrzCz2: emitter (70: 30: 5 wt.%, 25 nm)/SiTrzCz2 (5 nm)/ TmPyPB ( 40 nm)/LiF (1 nm)/Al (130 nm); [185] ITO)/ HATCN (5 nm)/ TAPC (30 nm)/ TCTA (10 nm)/ mCBP: 30 wt% 3CTF: 1.0 wt% IDID2BN (24 nm)/ CzPhPy (10 nm)/ DPPyA (30 nm)/LiF (0.5 nm)/Al (150 nm); [186] ITO/ HAT-CN (10 nm)/TAPC (50 nm)/ TCTA (10 nm)/PhCzBCz (2 nm)/PhCzBCz: 3 wt.% IDAD-BNCz or TIDAD-BNCz (20 nm)/TmPyPB (40 nm)/LiF (1 nm)/Al (100 nm); [187] ITO/TAPC (30 nm)/TCTA (10 nm)/mCP (10 nm)/PhCzBCz: 7 wt.% BNCzPXZ or 15 wt.% BNCzPTZ (20 nm)/TmTyPB (40 nm)/LiF (1 nm)/Al (120 nm); [188] HAT-CN (5 nm)/ TAPC (30 nm)/ TCTA (15 nm)/ mCBP (10 nm)/ DBFPO: 25 wt% mMDBA-DI: 1 wt% emitter (EML, 30 nm)/ DBFPO (2 nm)/ ANT-BIZ (30 nm)/ Liq (2 nm)/ (Al, 100 nm); [189] ITO/HAT-CN (5 nm)/ TAPC (30 nm)/TCTA (15 nm)/mCBP (10 nm)/ DMIC-TRZ: 20wt% PO-01 1 wt% emitter (EML, 30 nm)/ POT2T (20 nm)/ANT-BIZ (30 nm)/Liq (2 nm)/Al (100 nm); [190] ITO (50 nm)/ HATCN (5 nm)/ TAPC ( 30 nm)/ TCTA (15 nm)/ mCBP (10 nm)/2,6-DCzPPy: 2 wt% emitter (20 nm)/PPF (15 nm)/ANT-BIZ (30 nm)/Liq (2 nm)/Al (100 nm); [191] ITO (50 nm)/ HATCN (5 nm)/ TAPC ( 30 nm)/ TCTA (15 nm)/ mCBP (10 nm)/ 74 wt% PPF: 25 wt% 3Cz2BN: 1 wt% emitter (20 nm)/PPF (15 nm)/ANT-BIZ (30 nm)/Liq (2 nm)/Al (100 nm); [192] ITO/PEDOT: PSS (40 nm)/ /mCPCN: 5 wt% emitter (25 nm)/TmPyPB (45 nm)/LiF (0.5 nm)/Al (120 nm); [193] ITO/HAT-CN (5 nm)/ TAPC (45 nm)/ mCP (5 nm)/ 2,6-DCzPPy: 5 wt% emitter (20 nm)/ TmPyPB (50 nm)/ LiF (1 nm)/ Al (100 nm); [194] ITO/ TAPC (50 nm)/ TCTA (5 nm)/ 2,6-DCzPPy: 3 wt% emitter (30 nm)/ TmPyPB (30 nm)/ Liq (1 nm)/Al (100nm); [195] ITO (50 nm)/HAT-CN (10 nm)/NPD (40 nm)/TCTA (10 nm)/ mCBP (5 nm)/mCBP: 20 wt% HDT-1: 0.3 wt% emitters (30 nm)/ SF3-TRZ (5 nm)/SF3-TRZ: 50 wt% Liq (30 nm)/Liq (2 nm)/ Al (100 nm); [196] ITO (50 nm)/ HATCN (10 nm)/ TAPC (40 nm)/ mCBP (10 nm)/ mCBP: 2 wt% emitter (20 nm)/B3PyPB (30 nm)/Liq (2 nm)/Al (100 nm); [197] ITO/ HAT-CN (5 nm)/TAPC (30 nm)/TCTA (15 nm)/mCBP (10 nm)/ DMIC-TRZ: 20 wt% Ir(mphmq)2tmd: 1 wt% emitter (20 nm)/POT2T (20 nm)/ANT-BIZ (30 nm)/Liq (2 nm)/Al (100 nm); [198] ITO/HAT-CN (5 nm)/ TAPC (30 nm)/TCTA (15 nm)/mCBP (10 nm)/26DCzPPy: 20 wt% 3Cz2BN: 1 wt% DCzBNO or TCzBNO (25 nm)/PO-T2T (10 nm)/ANT-BIZ (30 nm)/Liq (2 nm)/Al (100 nm); [199] ITO/HATCN (5 nm)/ TAPC (50 nm)/TCTA (5 nm)/mCP (5 nm)/CzSi: 1 wt% emitters (20 nm)/DPEPO (5 nm)/TmPyPB (30 nm)/LiF (1 nm)/Al (120 nm); [200] ITO/HATCN (5 nm)/ TAPC (30 nm)/ TCTA (15 nm)/mCP (10 nm)/ DMIC-TRZ: 25 wt% 5TCzBN: 1 wt% emitters (30 nm)/ PO-T2T (20 nm)/ANT-BIZ (30 nm)/Liq (1 nm)/Al (120 nm); [201] ITO/ triphenylaminecontaining polymer [4-isopropyl-40-methyldiphenyl-iodonium tetrakis(pentafluorophenyl)borate] (20 nm)/TAPC (30 nm)/ mCP (10 nm)/8 wt% emitter-doped mCBPCN (20 nm)/3TPYMB (50 nm)/LiF (0.5 nm)/Al (100 nm); [202] ITO/ HATCN (10 nm)/ TAPC (30 nm)/ TCTA (10 nm)/ mCBP: 2 wt% emitters (20 nm)/ TmPyPB (50 nm)/ LiF (1 nm)/ Al (100 nm); [203] ITO/ NPD (40 nm)/ TCTA (15 nm)/ mCBP (15 nm)/DOBNA-Ph: 1 wt% emitters (20 nm)/3,4-2CzBN (10 nm)/ BPy-TP2(20 nm)/ LiF (1 nm)/ Al (100 nm); [204] ITO/TAPC (30 nm)/mCP (10 nm)/ mCP: 2wt% emitter (20 nm)/POT2T (10 nm)/TmPyPB (30 nm)/LiF (1 nm)/Al; [205] ITO/TAPC (30 nm)/mCP (10 nm)/ mCP: PO-T2T: 1wt% emitter (mCP: PO-T2T =1: 1, 20 nm)/POT2T (10 nm)/TmPyPB (30 nm)/LiF (1 nm)/Al; [206] ITO/HAT-CN (10 nm)/ TAPC (30 nm)/TCTA (10 nm)/ /DMIC-TRZ: 1.0 wt% (Ir(ppy)3: 1.0 wt% BN-NAP or BN-ANAP (20 nm)/ TmPyPB (50 nm)/ LiF (1 nm)/ Al (100 nm); [207] ITO/ TAPC (30 nm)/TCTA (10 nm)/ /5.0 wt% BNCz-aDMAC or 7.0 wt%BNCz-PaDMAC in PhCzBCz (20 nm) / TmPyPB (40 nm)/ LiF (1 nm)/ Al (100 nm); [208] ITO / MoO3 (6 nm) / NPB (30 nm)|CzAcSF: 0.3% BO-N1 or BO-N2 (25 nm) / DPEPO (5 nm) / TmPyPB (30 nm) / LiF (0.5 nm) / Al; [209] ITO/PEDOT: PSS (40 nm)/TAPC (20 nm)/TCTA (5 nm)/mCBP: 3 wt% emitter (20 nm)/TmPyPB (40 nm)/LiF (1 nm)/Al (200 nm); [210] ITO /MoO3 (1 nm)/ TAPC (50 nm)/ mCP (10 nm)/ mCPCN: 2 wt % TTABN or 10 wt % TAzBN (20 nm)/ 3TPYMB (50 nm)/ LiF (0.5 nm)/ Al (100 nm); [211] ITO/MoO3 (10 nm)/TAPC (60 nm)/mCP (10 nm)/PPF: 5 wt% emitter (20 nm)/PPF (10 nm) / TmPyPB (30 nm)/LiF (1 nm)/Al (100 nm); [212] ITO / MoO3 (2 nm)/ TCTA (64 nm)/mCP: 5wt% DG7 (10 nm)/ TPBi ( 10 nm)/ BPhen: Cs (50 nm)/ Al (100 nm); [213] ITO / HAT-CN (6 nm) / HAT-CN (0.2%): TAPC (50 nm) / 6 wt% BN-Ad or 2 wt% BN-Ph: 26DCzPPy (20 nm) / Tm3PyP26PyB (60 nm) / LiF (1 nm) / Al (100 nm); [214] ITO /HATCN (15 nm)/ TAPC (50 nm)/TCTA (5 nm)/BCPO: 6 wt% DPC or DTP (30 nm)/ TmPyPB (30 nm)/LiF (1 nm)/Al (100 nm); [215] ITO/HAT-CN (5 nm)/ TCTA (15 nm)/ mCBP (10 nm)/ DMIC-TRZ): 20 wt % Ir(mphmq)2tmd: 1 wt % emitter (EML, 40 nm)/ PO-T2T (20 nm)/ ANT-BIZ (30 nm)/ Liq (2 nm)/ Al (100 nm); [216] ITO/ (50 nm)/ NPD (40 nm)/ TCTA (15 nm)/ mCP (15 nm)/ DOBNA-Ph: 0.5 wt% MRE (20 nm)/ 3,4-2CzBN (10 nm)/ BPy-TP2 (20 nm)/ LiF (0.8 nm)/Al (100 nm); [217] ITO/ (50 nm)/ NPD (40 nm)/ TCTA (15 nm)/ mCP (15 nm)/ DOBNA-Ph: 1 wt% emitter: 20 wt% 3Cz2DPhCzBN (20 nm)/ 3,4-2CzBN (10 nm)/ BPy-TP2 (20 nm)/ LiF (0.8 nm)/Al (100 nm); [218] ITO/ HATCN (10 nm)/NPB (30 nm)/BCzPh (10 nm)/mCBP: 2 wt% A-BN (30 nm)/CzPhPy (10 nm)/DPPyA (30 nm)/LiF (0.5 nm)/Al (150 nm); [219] ITO/ HATCN (10 nm)/NPB (30 nm)/BCzPh (10 nm)/mCBP: p4TzPhBN: 2 wt% A-BN (30 nm)/CzPhPy (10 nm)/DPPyA (30 nm)/LiF (0.5 nm)/Al (150 nm); [220] ITO(50 nm)/NPD(40 nm)/TCTA(15 nm)/mCP (15 nm)/1 wt% emitter: 99 wt% DOBNA-OAr (20 nm)/mSiTrz (10 nm)/BPy-TP2 (20 nm)/ LiF (1 nm)/Al (100 nm); [221] ITO/ HAT-CN (10 nm)/ α-NPD (30 nm)/ Tris-PCz (15 nm)/ CzSi (6 nm)/ TSPO1: 5Cz-BO (20 nm, 90 wt%: 10 wt%)/ CF3-TRZ (10 nm)/ Liq: BPPB (25 nm, 50 wt%: 50 wt%)/ Liq (2 nm)/ Al (100 nm); [222] ITO/ (50 nm)/ HATCN (5 nm)/ TAPC (30 nm)/ TCTA (15 nm)/ mCBP (10 nm)/ SiTRZCz2: 2 wt% emitter (15 nm)/ SiTRZCz2 (20 nm)/ ANT-BIZ (30 nm)/ Liq (2 nm)/Al (100 nm); [223] ITO/HAT-CN (5 nm)/F6-TCNNQ (5 nm)/ TAPC (30 nm)/TCTA (10 nm)/mCBP (10 nm)/3 wt% emitter: pPhBCzPh (30 nm)/PPF (10 nm)/TmPyPB (40 nm)/LiF/Al; [224] ITO/HAT-CN (5 nm)/TAPC (30 nm)/TCTA (15 nm)/mCBP (10 nm)/chiral emitters: DMIC-TRZ (2 wt%, 45 nm)/PO-T2T (20 nm)/ANT-BIZ (30 nm)/Liq (2 nm)/Al (100 nm); [225] ITO (50 nm)/ NPD (40 nm)/TCTA (15 nm)/ mCP (15 nm)/ /1 wt% v-DABNA-O-Me: 99 wt% emitter (20 nm)/ 3,4-2CzBN (10nm)/ BPy-TP2 (20 nm)/ LiF (1 nm)/Al (100 nm); [226] ITO (50 nm)/HATCN (9 nm)/ PCBBiF (72 nm)/PCzAC (12 nm)/DIC-TRZ: 2 wt% of BpIC-DPA or 3 wt% of BpIC-Cz (30 nm)/DDBFT (10 nm)/BPPB (47 nm)/Liq (1.5 nm)/Al (90 nm); [227] ITO / HATCN (5 nm)/ TAPC (40 nm)/ 3 wt% emitters: 2,6-DCzPPy (20 nm)/ TmPyPB (45 nm)/ LiF (1 nm)/ Al (100 nm); [228] ITO/TAPC (50 nm)/TCTA (5 nm)/PhCbBCz: 10 wt%BN-N-TPA, 5 wt%TPA-Cz-BN, 5 wt%TPA-PCz-BN and 10 wt%BN-PCz-TPA (30 nm)/TmPyPB (30 nm)/LiF (1 nm)/Al (100 nm); [229] ITO /TAPC (55 nm)/mCP (15 nm)/1 wt% f-DOABNA in DOBNA-Tol (30 nm)/PPT (10 nm)/BPyTP2 (30 nm)/Liq (2 nm)/Al (100 nm); [230] ITO/HATCN (5 nm)/TAPC (30 nm)/TCTA (15 nm)/MCBP (10 nm)/ 1 wt% PyBN or 1 wt% Pm-BN or 1wt% Py-BN and 20 wt% TDBA-PAS: DOBNA-OAr host (EML, 20 nm)/DPEPO (15 nm)/ANT-BIZ (30 nm)/Liq (2 nm)/Al (100 nm); [231] ITO / HATCN (10 nm)/ Tris-PCz (30 nm)/ mCBP (5 nm)/ 1 wt% h-BNCO-1: mCBP (D1), 1 wt% h-BNCO-1: PICTRZ2 (D2), and 1 wt% h-BNCO-1: 8 wt% 4CzIPN: PIC-TRZ2 (D3), (EML, 30 nm), respectively, while 1 wt% h-BNCO-1: 8 wt% 4CzIPN: PIC-TRZ2 (25 nm) + spacer(1 wt% h-BNCO-1: PIC-TRZ2 as a functional spacer, 5nm, for 4,)/ SF3-TRZ (30 nm)/ Liq (2 nm)/ Al (100 nm); [232] ITO (50 nm)/ NPD (40 nm)/ TCTA (15 nm)/ mCP (15 nm)/ 1 wt% emitter (DOB2-DABNA-A or DOB2-DABNA-B-NP): 99 wt% DOBNA-Tol (20 nm)/ 3,4-2CzBN (10 nm)/BPy-TP2 (20 nm)/ LiF (1 nm)/ Al (100 nm); [233] ITO/ HAT-CN (5 nm)/ TAPC (30 nm)/ TCTA (15 nm)/ mCBP (10 nm)/99 wt% DOBNA-OAr: 1 wt% emitter (20 nm)/ PPF (15 nm)/ ANT-BIZ (30 nm)/ Liq (2 nm)/ Al (100 nm); [234] ITO/ HAT-CN (5 nm)/ TAPC (30 nm)/ TCTA (15 nm)/ mCBP (20 nm)/99 wt% SiTrzCz2: 1 wt% emitter (20 nm)/ SiTrzCz2 (10 nm)/ ANT-BIZ (30 nm)/ Liq (2 nm)/ Al (100 nm); [235] ITO/ HAT-CN (5 nm)/ TAPC (30 nm)/ TCTA (15 nm)/ mCBP (10 nm)/DBFPO: 25 wt% mMDBA-DI: 1 wt% emitter (25 nm)/ DBFPO (15 nm)/ ANT-BIZ (30 nm)/ Liq (2 nm)/ Al (100 nm); [236] ITO/ HAT-CN (5 nm)/ TAPC (30 nm)/ TCTA (15 nm)/ mCBP (10 nm)/DBFPO: 25 wt% mMDBA-DI: 1 wt% emitter (25 nm)/ DBFPO (15 nm)/ ANT-BIZ (30 nm)/ Liq (2 nm)/ Al (100 nm)/CGL(3 nm) HAT-CN (5 nm)/ TAPC (30 nm)/ TCTA (15 nm)/ mCBP (10 nm)/DBFPO: 25 wt% mMDBA-DI: 1 wt% emitter (25 nm)/ DBFPO (15 nm)/ ANT-BIZ (30 nm)/ Liq (2 nm)/ Al (100 nm); [237] ITO/ PEDOT: PSS (40 nm)/ CBP: 14 wt% emitter (20 nm)/ TmPyPB (45 nm)/ LiF (1 nm)/ Al (100 nm); [238] ITO/ HAT-CN (5 nm)/ NPB (30 nm)/ BCzPh (10 nm)/ mCPBC: 15 wt% PO-01: 0.5 wt% AN-BN (20 nm)/ CzPhPy (5 nm)/ DPPyA (30 nm)/ LiF(0.5 nm)/ Al (150 nm); [239] ITO/ (PEDOT: PSS: PFI, 40 nm)/ DMIC-TRZ: emitter (5 wt%, 30 nm)/ POT2T (20 nm)/ ANT-BIZ (30 nm)/ Liq (2

## **Table S4**. Performance of B/N type MR-emitters in optimized OLEDs

nm)/ Al (100 nm); [240] ITO/ HATCN (5 nm)/ NPB (30 nm)/ SiCzCz (10 nm) / SiCzCz: SiTrzCz2: 4tCzBN: TB-PB (54 wt%: 29 wt%: 16 wt%: 1 wt%, 30 nm)/ SiTrzCz2 (10 nm)/ DPPyA (30 nm)/LiF (0.7 nm)/Al(150 nm); [241] ITO/TAPC (30 nm)/TCTA (10 nm)/mCP (10 nm)/SF3TRZ: 1 wt% LDABNA-1 (15 nm)/TmTyPB (35 nm)/LiF (1 nm)/Al (120 nm); [242] ITO/HAT-CN (10 nm)/TAPC (40 nm)/TCTA(10 nm)/PhCzBCz: 15 wt% emitter (20 nm)/TmPyPB (40 nm)/Liq(2 nm)/Al (80 nm); [243] ITO/HAT-CN (10 nm)/TAPC (40 nm)/TcTa (10 nm)/mCBP (8 nm)/mCBP: 2 wt % emitters (20 nm)/PPF (8 nm)/TmPyPB (35 nm)/Liq (2.5 nm)/Al (60 nm); [244] ITO)/TAPC (50 nm)/TCTA (10 nm)/1 wt% FSBN: DMIC-TRZ (30nm)/TmPyPB (35 nm)/LiF (1 nm)/Al (100 nm); [245] ITO)/TAPC (50 nm)/TCTA (10 nm)/ 1 wt% FSBN: 20 wt% PO-01: DMIC-TRZ (30nm)/TmPyPB (35 nm)/LiF (1 nm)/Al (100 nm); [246] ITO)/TAPC (50 nm)/TCTA (10 nm)/0.5 wt% emitter: DMIC-TRZ (30nm)/TmPyPB (30 nm)/LiF (1 nm)/Al (100 nm); [247] ITO/HATCN (5 nm)/TAPC(30 nm)/TCTA (10 nm)/mCBP (10 nm)/ /15 wt% emitter: PhCzBCz (20 nm)/TmPyPB (40 nm)/LiF (1 nm)/Al (100 nm); [248] ITO/PEDOT: PSS (40 nm)/ PVK / mCP): 5 wt% emitters / mSiTRZ (8 nm)/ TmPPPyTz (42nm) / LiF (1 nm) / Al (100 nm); [249] ITO/ HAT-CN (5 nm)/ α-NPD (50 nm)/ TCTA (10 nm)/ 54.5 wt % mCBP: 45 wt % PPF: 0.5 wt% emitter (20 nm)/ PPF (10 nm)/ BPPB (55 nm)/ Liq (2 nm)/ Al (120 nm); [250] ITO/ HAT-CN (5 nm)/ α-NPD (50 nm)/ TCTA (10 nm)/ 5Cz-TRZ: 20 wt % PO-01-TB: 0.5 wt% emitter (20 nm)/ PPF (10 nm)/ BPPB (55 nm)/ Liq (2 nm)/ Al (120 nm); [251] ITO / HATCN (5 nm)/ TAPC (40 nm) / TCTA (5 nm) / 2,6-Dczppy: 5 wt.% (rac/R/S)-S-AX-BN or (rac/R/S)-SO2-AXBN (20 nm)/ TmPyPB (45 nm)/ LiF (1 nm)/ Al (100 nm); [252] ITO/TAPC (50 nm)/TCTA (5 nm)/ PhCzBCz: 5 wt% (P/M)-BN-TP-ICz (30 nm)/TmPyPB (30 nm)/ LiF (1 nm)/Al (100 nm); [253] ITO / HATCN (5 nm)/ TAPC (45 nm)/ (R/S)-4-POtBuCzB (10 wt%) or 2-POtBuCzB ( 8 wt%): mCBP (20 nm)/ TmPyPB (45 nm)/ LiF (1 nm)/ Al (100 nm); [254] ITO / HATCN (5 nm)/ BPBPA (50 nm)/ SFAF (15 nm)/ DMIC-TRZ (95 wt%): 3 wt%$Ir(ppy)_2$: 2 wt% emitter (45 nm)/ ANT-BIZ (40 nm)/ Liq (1 nm)/ Al (100 nm); [255] ITO / HATCN (5 nm)/ TBBD (30 nm)/ BiCar (15 nm)/ DMIC-TRZ (95 wt%): 1 wt% emitter (45 nm)/ PO-T2T (40 nm)/ ANT-BIZ (30 nm)/Liq (1 nm)/ Al (100 nm); [256] ITO/ TAPC (30 nm)/ TCTA (10 nm)/ mCP (10 nm)/ 1 wt.% NT-2B/NT-3B: 10wt.% Ir(ppy)3: PhCzBCz (30 nm) / TmPyPB (40 nm)/ LiF (1)/ Al (150 nm); [257] ITO/HATCN(5 nm)/ TAPC (30 nm)/ TCTA (10 nm)/5 wt% emitters inPhCzBCz (30 nm)/ TPBi (40 nm)/ LiF (1 nm)/ Al (120 nm); [258] ITO/ TAPC (30 nm)/ TCTA (10 nm)/ mCP (10 nm) /3Cz2BN: 1 wt.% A-BN or m-MDBA-DI: 1 wt.% DA-BN: or m-MDBA-DI: 1 wt.% A-DBN (20 nm)/ PPF (10 nm)/ TmPyPB (40 nm)/ LiF (1 nm)/ Al (100 nm); [259] ITO/HAT-CN (5 nm)/TAPC (30 nm)/SiCzCz (10 nm)/3% (R)-SFDBN-CN or (S)-SFDBNCN: 10% PtON-TBBI: SiTrzCz2 (30 nm)/ TmPyPB (30 nm)/ LiF (1 nm)/Al (100 nm); [260] ITO/PEDOT: PSS (35 nm)/mCP: 1 wt% emitter (35 nm)/ DBFPO(10 nm)/TmPyPB (50 nm)/ Liq (2 nm)/ Al (100 nm); [261] ITO/PEDOT: PSS (30 nm)/CBP: 2 wt % ICzBO (20 nm)/TmPyPB (50 nm)/LiF (1 nm)/Al (100 nm); [262] ITO (100 nm)/ /TAPC (40 nm)/TCTA (10 nm)/ SpiroAC-TRZ: 5wt% GBN (20 nm)/TmPyPB (50 nm)/ LiF(0.5 nm)/Al (100 nm); [263] ITO/MoO3 (10 nm)/TAPC (60 nm)/mCP (10 nm)/PPF: 6 wt% emitter (20 nm)/PPF (10 nm)/TmPyPB (30 nm)/LiF (1 nm)/Al (100 nm); [264] ITO/HATCN (4.3 nm)/TAPC (33.3 nm)/mCP: 5 wt% H[6]BN1 (20 nm)/TPBI (21 nm)/LiF (1 nm)/Al; [265] ITO/ HATCN (10 nm)/ TAPC (30 nm)/ 2 wt% HBN *: mCBP* or *2 wt%* HBN*:* 30 wt% Bt2Ir(acac): mCBP (20 nm)/ TmPyPB (50 nm)/ LiF (1 nm)/ Al (100 nm); [266] ITO/ $MoO_3$ (20 nm)/ mCP (20 nm)/ mCP: 10 wt% emitter (20 nm)/ (TmPyPB, 50 nm)/ LiF (1 nm)/ Al (100 nm); [267] ITO/ PEDOT: PSS (50 nm)/ PVK (15 nm)/ 8CzTPS: 20 wt% 5CzTRZ: 0.6 wt%D2-DBN (30 nm)/ mSiTRZ (12 nm)/ TmPPPyTz (55 nm)/ LiF (1 nm)/ Al (150 nm); [268] ITO/ HATCN (5 nm)/ TAPC (30 nm)/ TCTA (15 nm)/ mCBP (10 nm)/ 2 wt% emitter: DMIC-TRZ (45 nm)/ POT2T (20 nm)/ ANT-BIZ (30 nm)/ Liq (2 nm)/ Al (100 nm); [269] ITO/ HATCN (5 nm)/ TAPC (30 nm)/ TCTA (10 nm)/ 3 wt% emitter: PhCzBCz (45 nm)/ PPF (10 nm)/ TmPyPB (40 nm)/ LiF (2 nm)/ Al (100 nm); [270] ITO/ HATCN (5 nm)/ TAPC (30 nm)/ SiCzCz (10 nm)/ 2 wt% emitter: m4TCzPhBN: SiCzCz: SiTrCz2 (45 nm)/ SiTrCz2 (10 nm)/ DPPyA (30 nm)/ LiF (0.5 nm)/ Al; [271] ITO/ HATCN (10 nm)/ NPB (30 nm)/ TrisPCz (15 nm)/ mCBP (8 nm)/ mCBP: 18 wt% 3Cz-TRZ : 1.5 wt% 5Cz-BNO or mCBP: 18 wt% 5Cz-TRZ : 1.5 wt% 5Cz-BN (20 nm)/ CF3-TRZ (8 nm)/ 50 wt% Liq: 50 wt% BPPB (30 nm)/ Liq (2 nm)/ Al (120 nm); [272] ITO (135 nm)/ HATCN (5 nm)/ NPB (30 nm)/ SiCzCz (10 nm)/ 1 wt% emitter: TADF sensitizer (m4TCzPhBN or p4TCzPhBN) : SiCzCz: SiTrCz2 (45 nm)/ SiTrCz2 (10 nm)/ DPPyA (30 nm)/ LiF (0.5 nm)/ Al; [273] ITO/ HATCN (5 nm)/ TAPC (30 nm) /TCTA (15 nm)/mCBP (10 nm)/ DMIC-TRZ: 1 wt% emitter (35 nm)/ POT2T (10 nm)/ ANT-BIZ (30 nm)/ Liq (2 nm)/ Al (100 nm;

# Table S5. Performance of carbonyl / amino-type MR-emitters in optimized OLEDs

| Emitter | EL [nm] | FWHM [nm] | CIE (x,y) | $L_{max}$ [cd m$^{-2}$] | $CE_{max}/CE_{100}/CE_{1000}$ [cd m$^{-2}$] [a)] | $PE_{max}/PE_{100}/PE_{1000}$ [lm W$^{-2}$] [a)] | $EQE_{max}/EQE_{100}/EQE_{1000}$ [lm W$^{-2}$] [a)] | Efficiency Roll-off [%] [b)] | Ref. |
|---|---|---|---|---|---|---|---|---|---|
| QAO [1)] | 468 | 39 | 0.13,0.18 | ＜1000 | 26.2/-/- | 31.6/-/- | 19.4/9.2/- | 53 | 224 |
| 3-Ph-QAD [2)] | 480 | 44 | 0.13,0.32 | 4975 | 33.5/19.3/- | 32.9/16.0/- | 19.1/11.0/- | 42 | 225 |
| 7-Ph-QAD [2)] | 472 | 34 | 0.12,0.24 | 2944 | 28.8/8.8/- | 28.2/8.8/- | 18.7/5.8/- | 69 | |
| DDiKTa [3)] | 500 | 59 | 0.18,0.53 | ＜1000 | 52.4/-/- | 44.4/-/- | 19.0/-/- | - | 227 |
| QAO [4)] | 465 | 39 | 0.14,0.18 | 10385 | - | - | 14.7/8.3/3.3 | 44 | 226 |
| $Mes_3$DiKTa [4)] | 480 | 36 | 0.12,0.32 | 12949 | - | - | 21.1/14.5/4.5 | 31 | |
| QA-1 [5)] | 455 | 49 | 0.14,0.12 | - | 18.1 | 12.9 | 17.1/1.2/- | 92.9 | 228 |
| QA-2 [5)] | 463 | 37 | 0.13,0.14 | - | 20.8 | 14.2 | 19.0/11.0/- | 42.1 | |
| QA-3 [5)] | 515 | 67 | 0.26,0.62 | - | 64.1 | 45.8 | 18.6/11.1/- | 40.3 | |
| mBDPA-TOAT [6)] | 600 | 45 | 0.61,0.39 | - | 37.1 | 36.4 | 17.3 | -- | 229 |
| pBDPA-TOAT [6)] | 624 | 62 | 0.66,0.34 | - | 12.6 | 11.7 | 11.3 | | |
| DMAC-TOAT [6)] | 616 | 104 | 0.59,0.39 | - | 2.1 | 1.5 | 1.5 | - | |
| TOAT-5 [7)] | | | 0.57, 0.43 | | | | 2 | - | 230 |
| QAOCz1 [8)] | 516 | 44 | 0.23,0.66 | 11320 | 60 | 56.5 | 16.9 | - | 233 |
| QAOCz2 [8)] | 504 | 43 | 0.18,0.62 | 7679 | 60 | 57.0 | 19.4 | - | |
| QAOCz3 [8)] | 500 | 40 | 0.16,0.57 | 6217 | 60 | 56.7 | 21.1 | - | |
| BOQAO [9)] | 484 | 32 | 0.11,0.37 | | | | 21.8 | - | |
| (P)-QAO-PhCz [10)] | 467 | 36 | 0.13, 0.18 | - | 17.5 | 14.9 | 14.0 | - | 231 |
| Cz-DiKTa [11)] | 511 | 62 | 0.24,0.61 | 13260 | | 68.9 | 24.9/20.4/13.0 | 18.1 | 235 |
| Cz-Ph-DiKTa [11)] | 492 | 61 | 0.18,0.50 | 8529 | | 52.3 | 23.0/19.3/10.2 | 16.1 | |
| TMCz-DiKTa [11)] | 527 | 78 | 0.32,0.60 | 21758 | | 60.0 | 20.2/19.6/16.7 | 2.9 | |
| DMAC-DiKTa [11)] | 549 | 89 | 0.40,0.57 | 35506 | | 78.6 | 23.8/22.3/19.9 | 6.3 | |
| 3Cz-DiKTa [11)] | 547 | 54 | 0.39,0.60 | 10796 | | 83.1 | 24.4/17.3/6.2 | 29.1 | |
| QA-PF [12)] | 474 | 27 | 0.12,0.17 | 1740 | 21.1 | | 16.8/5.6/- | 66.7 | 236 |
| QA-PCN [12)] | 473 | 30 | 0.12,0.18 | 2760 | 22.6 | | 16.9/9.4/- | 44.4 | |
| QA-PMO [12)] | 484 | 27 | 0.11,0.30 | 3040 | 26.6 | | 15.0/3.5/- | 76.7 | |
| QA-PCz [12)] | 482 | 29 | 0.11,0.28 | 3600 | 30.7 | | 17.5/7.6/- | 56.6 | |
| QAD-Cz [13)] | 494 | 57 | 0.16,0.47 | | 49.0 | 44.0 | 20.3 | - | 237 |
| QAD-2Cz [13)] | 530 | 56 | 0.30,0.65 | | 103.1 | 104.4 | 27.3 | - | |
| QAD-mTDPA [14)] | 589 | 67 | 0.55,0.44 | | 66.7 | 65.4 | 26.3 | - | |
| DQAO [15)] | 472 | 34 | 0.12,0.18 | | 20 | 23.0/10.1/- | 15.2/8.5/- | 44.1 | 238 |
| OQAO [15)] | 532 | 45 | 0.32,0.65 | | 80 | 94.4/62.0/- | 20.3/15.1/- | 25.6 | |
| SQAO [16)] | 564 | 72 | 0.47,0.52 | | 60 | 53.4/31.5/- | 17.8/13.6/- | 23.6 | |
| CzAO [17)] | 444 | 43 | 0.148, 0.065 | 636 | 5.46 | 4.28 | 8.62 | - | 239 |
| MQAO [17)] | 460 | 62 | 0.142, 0.139 | 1368 | 11.02 | 9.60 | 10.28 | - | |
| QPXO [17)] | 486 | 67 | 0.166, 0.406 | 4044 | 16.20 | 14.11 | 7.05 | - | |
| QPO [17)] | 510 | 82 | 0.237, 0.529 | 3217 | 45.56 | 25.96 | 15.29 | - | |
| QPO-PhCz [18)] | 488 | 72 | 0.17, 0.34 | | 25.0 | 30.5 | 10.6 | - | 240 |
| tCPD [19)] | 530 | 77 | 0.36, 0.60 | 1640 | 23.5 | 17.7 | 6.5 | - | 241 |
| 2tCPD [19)] | 542 | 48 | 0.39, 0.59 | 1591 | 44.9 | 35.2 | 12.0 | - | |
| tPD [19)] | 492 | 30 | 0.13, 0.47 | 2258 | 50.4 | 37.7 | 22.0 | - | |
| 2tCPD [20)] | | | 0.15, 0.41 | 28353 | 78.8 | 88.4 | 22.0 | - | |
| tPD [20)] | | | 0.33, 0.62 | 11510 | 58.3 | 67.8 | 27.1 | - | |
| CzCO [21)] | 432 | 35/0.22 | 0.154,0.047 | | 8.6/4.2/- | | 15.6/6.0/5.9 | 61.5 | 242 |
| Cz2CO [21)] | 445 | 23/0.14 | 0.154,0.061 | | 9.4/7.6/- | | 13/9.9/3.9 | 23.8 | |
| CzCO [22)] | 432 | 44/0.28 | 0.154,0.051 | | 13.6/13.6/- | 7.9/7.9/- | 26.9/26.8/24.3 | 0.3 | |
| Cz2CO [22)] | 445 | 26/0.16 | 0.157,0.074 | | 16.4/7.0/- | 5.1/3.5/- | 25.6/10.1/22.4 | 60.5 | |
| DOBDiKTa [23)] | 458 | 38.1 | 0.14,0.12 | 4707 | | | 17.4/11.8/5.5 | 32.2 | 243 |
| 2,3-CZ [24)] | 465 | 55 | 0.13,0.15 | 2758 | 11.6 | 9.9 | 8.1 | - | 244 |
| 2,5-CZ [24)] | 463 | 48 | 0.13,0.13 | 4387 | 28.6 | 26.4 | 22.3 | - | |
| 2,6-CZ [25)] | 489 | 66 | 0.15,0.33 | 4437 | 47.0 | 33.5 | 21.2 | - | |
| 2,3-DPA [25)] | 503 | 54 | 0.17,0.54 | 8000 | 35.2 | 23.5 | 11.7 | - | |
| 2,3-POA [25)] | 528 | 68 | 0.30,0.62 | 38491 | 80.1 | 51.4 | 21.7 | - | |
| SFQ [26)] | 460 | 30 | 0.133,0.106 | | 20.0 | 17.3 | 21.7/12.5/3.6 | 42.4 | 245 |
| SOQ [26)] | 456 | 33 | 0.137,0.104 | | 20.0 | 17.6 | 24.3/14.9/4.2 | 38.7 | |
| SSQ [26)] | 456 | 31 | 0.137,0.105 | | 20.0 | 17.2 | 25.5/13.4/3.5 | 47.4 | |
| SSeQ [26)] | 460 | 35 | 0.144,0.120 | | 20.0 | 17.2 | 22.2/13.0/3.1 | 41.4 | |
| Sym-DiDiKTa [27)] | 543 | 36 | 0.359,0.624 | 4310 | | | 9.8/1.8/0.8 | 81.6 | 246 |
| Asym-DiDiKTa [27)] | 544 | 36 | 0.369,0.616 | 3989 | | | 10.5/1.3/0.8 | 87.6 | |
| Asym-DiDiKTa [28)] | 548 | 56 | 0.397,0.592 | 53625 | | | 19.9/9.9/6.4 | 50.3 | |
| [5]he-BQAO [29)] | 471 | 43 | | | | 4.9 | 3.3/1.6/1.2 | 51.5 | 247 |
| hp-BQAO [29)] | 471 | 41 | | | | 33.0 | 24.1/18.1/8.2 | 24.9 | |
| CzP2PO [30)] | 379 | 34 | 0.15,0.04 | 2936 | 1.29 | 1.12 | 8.01, 7.97, 8.00 | 0.5 | 248 |
| tBCzP2PO [30)] | 384 | 32 | 0.14,0.04 | 2696 | 2.46 | 1.70 | 15.1, 13.5, 14.7 | 10.6 | |
| MTDMQAO [31)] | 472 | 39 | 0.13,0.21 | | 38.3 | 40.1 | 29.4/21.1/- | 28.2 | 249 |
| MBDMQAO [31)] | 480 | 27 | 0.12,0.28 | | 26.3 | 29.1 | 18.9/4.7/- | 75.1 | |
| DMQAO [31)] | 472 | 33 | ,0.18 | | 20.0 | 23.0 | 15.2/8.5/- | 44.1 | |
| DiKTa-LC [32)] | 492 | 51 | 0.22,0.49 | 1000 | | | 13.6 | - | 252 |
| S-DAO [33)] | 502 | 26 | 0.12, 0.62 | | 80.1/54.9/18.9 | 62.4/35.8/9.2 | 29.9/19.2/7.1 | 35.8 | 253 |
| SS-DAO [33)] | 520 | 24 | 0.20,0.73 | | 136.0/88.0/25.1 | 115.5/57.9/12.1 | 37.2/24.8/7.1 | 33.3 | |
| 3TPA-DiKTa [34)] | 551 | 62 | 0.409,0.577 | 36400 | 117 | 108 | 30.8/18.1/7.3 | 41.2 | 254 |
| 3DPA-DiKTa [34)] | 613 | 60 | 0.633,0.365 | 8800 | 28 | 27 | 16.7/3.4/1.9 | 79.6 | |
| 3TPA-DiKTa [35)] | 556 | 70 | 0.424,0.551 | 112190 | 106 | 111 | 30.0/27.4/20.0 | 8.7 | |
| 3DPA-DiKTa [35)] | 615 | 61 | 0.585,0.396 | 46003 | 35 | 37 | 17.9/8.7/6.0 | 51.4 | |
| PCP-DiKTa [36)] | 489 | 53 | 0.162,0.450 | 5558 | | | 25.7/19.1/8.7 | 25.7 | 255 |
| Czp-DiKTa [36)] | 518 | 69 | 0.256,0.610 | 10689 | | | 29.2/25.9/17.1 | 11.9 | |
| TPQAO [3 7)] | 468 | 31 | 0.124, 0.146 | | 20.0 | 18.0 | 18.1 | - | 256 |
| TTPQAO [37)] | 480 | 33 | 0.117, 0.221 | | 30.0 | 25.4 | 21.3 | - | |
| DDiKTa-A [38)] | 574 | 75 | 0.48,0.50 | 12310 | | | 24.3/22.5/14.6 | 7.4 | 257 |
| SpiroO-QAO [39)] | 487 | 34/0.174 | 0.13,0.41 | 5949 | 38.1 | 41.3 | 18.0 | - | 258 |
| $SpiroOSO_2$-QAO [39)] | 492 | 39/0.196 | 0.16,0.49 | 7270 | 38.0 | 42.6 | 18.5 | - | |
| SpiroS-QAO [39)] | 507 | 44/0.208 | 0.19,0.63 | 11961 | 70.2 | 81.6 | 27.3 | - | |
| $SpiroSO_2$-QAO [39)] | 470 | 46/0.251 | 0.13,0.19 | 393 | 12.2 | 8.32 | 9.0 | - | |
| SpiroO-QAO [40)] | 492 | 45 | 0.17,0.46 | 48289 | | | 30.8 | - | |
| SpiroOSO2-QAO [40)] | 490 | 48 | 0.17,0.43 | 46796 | | | 30.3 | - | |
| SpiroS-QAO [40)] | 511 | 47 | 0.21,0.63 | 53616 | | | 29.2 | - | |
| DPQAO-M [41)] | 471 | 27 | 0.126,0.198 | | 18.1, 6.2, 2.9 | 15.0, 4.0, 1.2 | 16.6, 4.6, 2.2 | 72.3 | 259 |
| DPQAO-F [41)] | 463 | 24 | 0.134,0.118 | | 22.4, 8.8, 3.0 | 20.1, 7.0, 1.6 | 19.9, 8.3, 3.0 | 58.3 | |
| DPQAO-F [42)] | 461 | 36 | 0.146, 0.117 | | 32.03, 19.2, 9.5 | 28.0, 13.5, 4.0 | 32.7, 17.6, 9.9 | 46.2 | |

| | | | | | | | | | |
|---|---|---|---|---|---|---|---|---|---|
| 2AcPh [43] | 476 | 30 | 0.12,0.26 | 1126 | 39.17 | | 28.52 | | 260 |
| mTIAO [44] | 450 | 26/0.16 | 0.147, 0.063 | 2094 | 13.0 | 12.8 | 29.6/4.8/3.3 | 83.8 | 262 |
| pTIAO [44] | 472 | 26/0.14 | 0.128, 0.208 | 7075 | 47.4 | 49.7 | 34.4/9.3/6.1 | 72.9 | |
| pTIQA [44] | 475 | 25/0.14 | 0.299, 0.636 | 140100 | 66.1 | 59.5 | 21.1/20.6/20.6 | 2.4 | |
| NCON-TB [45] | 530 | 40/0.17 | 0.314,0.617 | 14480 | 73.2 | 46.9 | 20.0/19.8/10.8 | 1.0 | 280 |
| NCON-Mes [45] | 524 | 39/0.17 | 0.279,0.617 | 16340 | 97.2 | 61.0 | 27.7/26.6/13.8 | 3.98 | |

[1] ITO/HATCN, 10 nm)/TAPC, (45 nm)/TCTA, (10 nm)/5 wt% QAO: mCP (20 nm)/B3PYMPM (40 nm)/ Liq, (2 nm)/Al (120 nm); [2] ITO/TAPC (35 nm)/TCTA (10 nm)/mCP: 2 wt% 3-Ph-QAO or 3-Ph-QAO (20 nm)/TmPyPB (40 nm)/LiF (1 nm)/Al; [3] ITO/TAPC (35 nm)/TCTA (10 nm)/CzSi (10 nm)/9 wt% DDiKTa: DPEPO (20 nm)/TmPyPB (40 nm)/LiF (1 nm)/Al (100 nm); [4] ITO/HAT-CN (10 nm)/TAPC (40 nm)/TCTA (10 nm)/3.5 wt% QAO or Mes3DiKTa: mCP (20 nm)/TmPyPB (50 nm)/LiF (1 nm)/Al (100 nm); [5] TO/ HAT-CN(10 nm)/ TAPC (40 nm)/ CCP ( 10 nm)/3 wt%-emitter: PPCz (20 nm)/ PPF (10 nm)/ B3PYMPM ( 40 nm)/ (Liq, 1 nm)/Al (100 nm); [6] ITO /TAPC (40 nm)/TCTA (10 nm)/CBP: x wt% emitter (x =8.0, 13.0, and 2.0 wt% for mBDPA-TOAT, pBDPA-TOAT, and DMAC-TOAT 20 nm)/TmPyPB (45 nm)/LiF (1 nm)/Al; [7] ITO/HAT-CN (10 nm)/Tris-PCz (30 nm)/3 wt% TOAT-5: mCBP (30 nm)/T2T (10 nm)/ BPy-TP2 (40 nm)/ LiF (0.8 nm)/Al (100 nm); [8] ITO/ HAT-CN, (10 nm)/TAPC(40 nm)/ TCTA (10 nm)/ 5 wt%-emitter: CBP ( (20 nm)/ TmPyPB ( 50 nm)/ Liq (2 nm)/Al (100 nm); [9] ITO/HAT-CN (10 nm)/ TAPC (40 nm)/ TCTA (10 nm)/CBP: 5 wt% BOQAO (20 nm)/ TmPyPB (45 nm)/Liq (2 nm)/Al (100 nm); [10] ITO/HAT-CN (10 nm)/TAPC (40 nm)/TCTA (10 nm)/ 5 wt% (P)-QAO-PhCz: mCBP (20 nm)/ PPF (10 nm)/ TmPyPB (40 nm)/Liq (2 nm)/Al (120 nm); [11] ITO/ TAPC (40 nm)/mCP (10 nm)/7.5 wt % emitter: mCP (20 nm)/ PPT (10 nm)/ TmPyPB ( 50 nm)/LiF (1 nm)/Al (100 nm); [12] ITO/ HATCN (10 nm)/TAPC (45 nm)/TCTA (10 nm)/mCP: 3 wt% emitter (20 nm)/TmPyPB (40 nm)/LiF (1 nm)/Al; [13] ITO/TAPC (30 nm)/TCTA (10 nm)/ mCP (10 nm)/ 1.0 wt % QAD-Cz or 12.0 wt % QAD-2Cz: mCP(20 nm)/TmPyPB (45 nm)/LiF (1 nm)/Al for QAD-Cz and QAD-2Cz; [14] ITO/TAPC (30 nm)/TCTA (10 nm)/ 1.5 wt % QADmTDPA: CBP(20 nm)/TmPyPB (45 nm)/LiF (1 nm)/Al for QAD-Cz and QAD-2Cz; [15] ITO/ HATCN (10 nm)/TAPC (40 nm)/TCTA (10 nm)/mCP: 8 wt% DQAO or CBP: 5 wt%OQAO or mCPCN: 1 wt%SQAO (20 nm)/B4PYMPM for DQAO and OQAO or 3TPYMB (40 nm)/Liq (1 nm)/Al; [16] ITO/ HATCN (10 nm)/TAPC (40 nm)/TCTA (10 nm)/ mCPCN: 1 wt%SQAO (20 nm)/ 3TPYMB (50 nm)/Liq (1 nm)/Al; [17] ITO/PEDOT: PSS (35 nm)/mCP (30 nm)/mCPCN: 3 wt% enantiomers (30 nm)/TmPyPB (50 nm)/LiF (0.5 nm)/Al (120 nm); [18] ITO/ HATCN (10 nm)/ TAPC (45 nm)/ TCTA (10 nm)/mCP (8 nm)/DPEPO: x wt% emitters (20 nm)/DPEPO (8 nm)/ TmPyPB (40 nm)/Liq (2 nm)/Al (120 nm); [19] ITO/TAPC (30 nm)/TCTA (10 nm)/26DCzPPy: 1 wt% emitters (20 nm)/TmPyPB (40 nm)/LiF (1 nm)/Al (150 nm); [20] ITO/TAPC (30 nm)/TCTA (10 nm)/ CBP: 20 wt % 4CzIPN: 1 wt % 2tCPD or PhCzBCz: 15 wt % 5tCzBN: 1 wt% tPD (20 nm)/ TmPyPB (40 nm)/LiF (1 nm)/Al (150 nm); [21] ITO/TAPC (45 nm)/TCTA (5 nm)/ mCP (5 nm)/ mCP: 3 wt% emitters (20 nm)/PPF (5 nm)/TPBi (35 nm)/LiF (1 nm)/Al (100 nm); [22] ITO/TAPC (45 nm)/TCTA (5 nm)/ mCP (5 nm)/ PPF: 15 wt% TDBA-PAS: 3 wt% emitters (20 nm)/PPF (7 nm)/ Bphen (40 nm)/LiF (1 nm)/Al (100 nm); [23] ITO/HATCN (5 nm)/TAPC (40 nm)/TCTA (10 nm)/mCP (10 nm)/ 1.5 wt % DOBDiKTa in 1: 1 mCP: PPT (20 nm)/PPT (10 nm)/TmPyPB (50 nm)/LiF (0.8 nm)/Al (100 nm); [24] ITO/HAT-CN (10 nm)/TAPC (45 nm)/TCTA (5 nm)/ mCBP: 3.5 wt% emitter (25 nm)/TmPyPB (50 nm)/LiF (1 nm)/Al (100 nm); [25] ITO/HAT-CN (10 nm)/TAPC (45 nm)/TCTA (5 nm)/ 26DCzPPy: 10 wt% emitter (25 nm)/TmPyPB (50 nm)/LiF (1 nm)/Al (100 nm); [26] ITO/HAT-CN (10 nm)/ TAPC (40nm)/ TcTa (10 nm)/ mCBP: 3 wt% emitter (20 nm)/ TmPyPB (40 nm)/ Liq (2.5 nm)/ Al (100 nm); [27] ITO (100 nm)/TAPC (35 nm)/TCTA (10 nm)/mCP: 3 wt% of SymDiDiKTa or Asym-DiDiKTa (20 nm)/TmPyPB (30 nm)/Liq (2 nm)/Al (100 nm); [28] ITO (100 nm)/ HAT-CN (10 nm)/Tris-PCz (30 nm)/mCBP (5 nm)/mCBP: 20 wt% 4CzIPN: 3 wt% Asym-DiDiKTa (30 nm)/T2T (10 nm)/BPy-TP2 (40 nm)/Liq (2 nm)/Al (100 nm); [29] ITO/ TAPC (30 nm)/ TCTA (10 nm)/ DCz-BTP: 3 wt% emitter (20 nm)/ TmPyPB (45 nm)/LiF (0.8 nm)/Al (100 nm); [30] ITO/MoO3 (6 nm)/*m*CP (45 nm)/*m*CP: *x*% emitter (25 nm)/TmPyPB (45 nm)/LiF (1 nm)/Al (100 nm); [31] ITO/HATCN (10 nm)/TAPC (40 nm)/ TCTA (10 nm)/10 wt% MTDMQAO or 10 wt% MBDMQAO or 8 wt% DMQAO in mCP (20 nm)/ $B_4$PYMPM (40 nm)/Liq (2 nm)/ Al (120 nm); [32] ITO (120 nm)/PEDOT: PSS (35 nm)/ PVK (20 nm): 10 wt% emitter (30 nm)/DPEPO (9 nm)/TmPyPB (40 nm)/LiF (1 nm)/Al (100 nm); [33] ITO/HATCN (15 nm/ TAPC (60 nm)/ TCTA (5 nm)/ *m*CBP: 10 wt % SS-DAO or *m*CBP: 1 wt % S-DAO / TmPyPB (30 nm)/LiF (1 nm)/Al (100 nm); [34] ITO/HATCN (5 nm)/TAPC (40 nm)/TCTA (10 nm)/mCP (10 nm)/emissive layer (2 wt% emitter in mCP, 20 nm)/TmPyPB (50 nm)/LiF (0.6 nm)/Al (100 nm)； [35] ITO/HATCN (5 nm)/TAPC (40 nm)/TCTA (10 nm)/mCP (10 nm)/emissive layer (10 wt% 4CzIPN and 2 wt% emitter in mCP, 20 nm)/TmPyPB (50 nm)/LiF (0.6 nm)/Al (100 nm)； [36] ITO/ HATCN (5 nm)/ TAPC (45 nm)/ mCP (5 nm)/26DCzPPy: 2 wt% PCP-DiKTa, or 5 wt% CzpDiKTa (20 nm) / TmPyPB (45 nm)/ LiF (1 nm)/ Al (100 nm); [37] ITO/HAT-CN (10 nm)/TAPC (40 nm)/TCTA (10 nm)/7 wt % TPQAO or 2 wt % TTPQAO in mCBP (20 nm)/TmPyPB (40 nm)/Liq (2 nm)/Al (100 nm); [38] ITO/TAPC (35 nm)/TCTA (10 nm)/CzSi (10 nm)/ 5 wt % DDiKTa-A: 10 wt % 4CzIPN: 85 wt % mCP /(20 nm)/TmPyPB (40 nm)/LiF (1 nm)/Al (100 nm); [39] ITO/ TAPC (30 nm)/ mCP (10 nm)/ PPF: 1 wt%, 5 wt%, 5 wt% and 1 wt% for SpiroO-QAO, SpiroOSO2-QAO, SpiroS-QAO and SpiroSO2-QAO, respectively (20 nm)/ TmPyPB (40 nm)/ LiF (1 nm)/ Al (120 nm); [40] ITO/ HAT-CN (5 nm)/ F6-TCNNQ (1 nm)/ TAPC (30 nm)/ TCTA (10 nm)/ mCP (10 nm)/ pPhBCzPh: PO-T2T: DspiroAc-TRZ: Emitters, where the ratio of pPhBCzPh: PO-T2T: DspiroAc-TRZ is 42: 28: 30 and the doping concentrations of emitters are 1, 0.8 and 1 wt% for SpiroO-QAO, SpiroOSO2-QAO and SpiroS-QAO, respectively (30 nm)/ PPF (10 nm)/ TmPyPB (40 nm)/ LiF (1 nm)/ Al (150 nm); [41] ITO/ TAPC (40 nm)/ TCTA (10 nm)/ mCP (10 nm)/ 2 wt%: Mcp (20 nm)/ TmPyPB (40 nm)/ LiF (1 nm)/ Al (100 nm); [42] ITO/ TAPC (50 nm)/ TCTA (10 nm)/ mCP (10 nm)/ PPF: 30 wt.% TDBA-SPX: 1.5 wt.% emitters (25 nm)/ PPF (5 nm)/ 3TPYMB (50 nm)/ LiF (1 nm)/ Al (100 nm); [43] ITO/ HAT-CN (15 nm)/ TAPC (60 nm)/ TCTA (5 nm)/ 1 wt % 2AcPh: mCBP (30 nm)/ TmPyPB (30 nm)/ LiF (1 nm)/ Al (100 nm); [44] ITO/ HATCN (5 nm)/ NPB (30 nm)/ TCTA(5 nm)/ mCP (5 nm)/ 30 wt% TB-tPCz: 1 wt.% mTIAO: PPF or 30 wt% TDBA-Ac: 1 wt.% pTIAO: PPF or 30 wt% 3CTF: 1 wt.% pTIQA: PPF (24 nm)/ PPF (10 nm)/ BPhen (30 nm)/ LiF (0.5 nm)/ Al (150 nm); [45] ITO)/ PEDOT: PSS (40 nm)/ 5CzBN-ESF: 2 wt.% emitters (EML, 40 nm)/ POT2T (40 nm)/ Cs2CO3 (1 nm)/ Al (100 nm);

# Table S6. Performance of N-PAHs type MR-emitters in optimized OLEDs.

| Emitter | $\lambda_{EL}$ [nm] | FWHM [nm] | CIE (x,y) | $L_{max}$ [cd m$^{-2}$] | $CE_{max}/CE_{100}/CE_{1000}$ [cd m$^{-2}$] [a)] | $PE_{max}/PE_{100}/PE_{1000}$ [lm W$^{-2}$] [a)] | $EQE_{max}/EQE_{100}/EQE_{1000}$ [lm W$^{-2}$] [a)] | Efficiency Roll-off [%][b)] | Ref. |
|---|---|---|---|---|---|---|---|---|---|
| ICzCz [1)] | 416 | 49 | 0.17,0.04 | - | 0.7/-/- | 0.5/-/- | 2.3/-/- | | 263 |
| ICzAc [1)] | 454 | 56 | 0.15,0.09 | - | 11.8/-/- | 10.6/-/- | 13.7/-/- | | |
| ICzDAc [1)] | 462 | 58 | 0.15,0.16 | - | 24.7/-/- | 22.2/-/- | 19.5/-/- | | |
| CNICCz [2)] | 449 | 56 | 0.15,0.08 | - | 9.9/4.9/- | 8.8/2.6/- | 12.4/6.4/- | 48.4 | 271 |
| CNICtCz [2)] | 456 | 60 | 0.14,0.13 | - | 17.2/11.5/-- | 13.4/6.1/- | 16.0/10.7/- | 33.1 | |
| pICz [3)] | 393 | 20 | 0.164,0.018 | 200 | | | 3.3/-/- | | 272 |
| IDCz-DPA [4)] | 456 | 41 | 0.14,0.09 | 23700 | 4.4/-/- | | 5.3/-/- | | 264 |
| IDCz-2DPA[4)] | 466 | 35 | 0.13,0.15 | 47600 | 6.4/-/- | | 5.6/-/- | <0.3 | |
| pICz [5)] | 441 | 18 | 0.15,0.10 | | 30.1/9.4/- | 25.4/3.4/- | 32.0/6.7/- | 79.1 | .265 |
| pICz-TPA [5)] | 447 | 21 | 0.15,0.085 | | 31.5/6.7/- | 27.1/6.4/- | 34.7/11.2/ | 67.7 | |
| BisICz [6)] | 437 | 24 | 0.16,0.04 | | 2.9 | 2.7 | 6.5/-/- | | 268 |
| tBisICz [6)] | 445 | 22 | 0.16,0.05 | | 8.4 | 8.3 | 15.1/-/- | | |
| tPBisICz [6)] | 452 | 21 | 0.15,0.05 | | 13.5 | 13.3 | 23.1/-/- | | |
| t3IDCz [7)] | 472 | 25 | 0.119,0.161 | | 36.9 | | 30.0/12.4/5.0 | 58.7 | 270 |
| p3IDCz [7)] | 472 | 23 | 0.120,0.158 | | 37.9 | | 30.9/12.6/4.7 | 59.2 | |
| t3IDCz [8)] | 471 | 26 | 0.116,0.158 | | 35.4 | | 30.9/11.4/- | 63.1 | |
| p3IDCz [8)] | 473 | 28 | 0.111,0.188 | | 43.4 | | 33.8/14.8/- | 56.2 | |
| p3IDCz [9)] | 474 | 33 | 0.116,0.213 | | 42.6 | | 30.5/21.0/7.7 | 31.1 | |
| DiICzMes$_4$ [10)] | 446 | | 0.15,0.11 | 130 | | | 3.0/1.9/- | 36.7 | 266 |
| DiICzMes$_4$ [11)] | 446 | | 0.15,0.11 | 1500 | | | 16.5/15.5/12.9 | 6.1 | |
| α-NAICZ [12)] | 610 | 38 | 0.66,0.34 | | 23.7/-/23.3 | | 18.2/-/17.7 | low | 267 |
| α-EtNAICZ [12)] | 633 | 56 | 0.68,0.32 | | 11.5/-/11.4 | | 12.9/-12.7 | low | |
| pSFIAc1 [13)] | 448 | 19/0.12 | 0.147, 0.054 | 14040 | | 4.6/-/2.9 | 8.9/-/8.2 | | 269 |
| pSFIAc2 [13)] | 453 | 19/0.12 | 0.142, 0.066 | 16250 | | 5.2/-/4.0 | 9.1/-/8.7 | | |
| pSFIAc1 [14)] | 446 | 21/0.13 | 0.148, 0.058 | 2002 | | 14.9/1.2/0.6 | 24.9/4.1/3.2 | 85.3 | |
| pSFIAc2 [14)] | 451 | 21/0.13 | 0.146, 0.078 | 2253 | | 23.0/3.0/1.4 | 31.4/5.6/1.4 | 82.2 | |
| tDIDCz [15)] | 401 | 14/0.105 | 0.164, 0.018 | - | | | 2.75 | high | 272 |
| tDIDCz [15)] | 402 | 20/0.148 | 0.164, 0.019 | 200 | | | 3.3 | | |
| m-FLDID [16)] | 407 | 17 | 0.168,0.024 | 125 | | | 4.4/2.7/- | 38.6 | 273 |
| | 409 | 22 | 0.166,0.025 | 210 | | | 5.1/3.2/- | 37.3 | |
| | 411 | 27 | 0.163,0.027 | 290 | | | 5.2/3.2/- | 38.5 | |
| Cz-DICz [17)] | 460 | 18 | 0.14,0.10 | | 25.62 | 26.67 | 25.6 | | 274 |
| tBisICz-DPA [18)] | 452 | 28 | 0.15,0.05 | | 12.3 | 12.1 | 20.4 | | 275 |
| tBisICz-PhCz[18)] | 446 | 19 | 0.16,0.04 | | 11.6 | 11.4 | 24.9 | | |
| Nm-ICz [19)] | 412 | 42 | 0.164,0.025 | 1788 | | | 3.0/2.7/1.5 | | 276 |
| CNm-ICz [19)] | 427 | 42 | 0.161,0.027 | 196 | | | 3.7/2.7/- | | |
| NB-1 [20)] | 449 | 13.9 | 0.1168,0.180 | | | | 16.4/14.6/- | 10.9 | 277 |
| NB-2 [20)] | 458 | 15.4 | 0.161,0.186 | | | | 21.5/17.8/- | 17.2 | |
| IDCz-DBS[21)] | 461 | 21 | 0.135,0.160 | 43300 | | | 31.1/26.2/29.9 | 15.8 | 278 |
| DIDCz-tBu [22)] | 468 | 34 | 0.139,0.184 | | 31.6/-/5.98 | 27.7/-/23.8 | 22.3/-/17.4 | | 279 |
| TSFQ-TRZ[23)] | 475 | 25 | | | 35/22.4/12.2 | | 28.3/18.2/9.9 | 35.7 | 261 |
| TSFQ-Ph[23)] | 476 | 29 | | | 30/18.6/7.8 | | 20.2/12.6/5.3 | 37.6 | |

[1)] ITO (50 nm)/ PEDOT: PSS (60 nm)/ TAPC (20 nm)/ mCP (10 nm)/ DPEPO: 10 wt% emittert (25 nm)/TSPO1 (5 nm)/TPBI (30 nm)/LiF (1.5 nm)/Al (200 nm). [2)] ITO ( 50 nm)/ PEDOT: PSS (60 nm)/ TAPC (20 nm)/ mCP (10 nm)/DPEPO: emitter (25 nm, 10%)/ TSPO1 (5 nm)/ TPBi (20 nm)/LiF(1.5 nm)/Al(200 nm). [3)] ITO ( 50 nm)/ NPB (75 nm)/ TCTA (5 nm)/ PCzAc (5 nm)/ mCP (5 nm)/ mCBP: TSPO1: emitter (25 nm, 3 wt%)/ TSPO1 (5 nm)/ TPBi (20 nm)/LiF(1.5 nm)/Al(200 nm). [4)] ITO (50 nm)/ BPBPA: HATCN (40 nm: 30%)/BPBPA (10 nm)/TNPA (10 nm)/ a-ADN: 5 wt% emitter (30 nm)/TNPT (5 nm)/ZADN (20 nm)/LiF (1.5 nm)/Al (200 nm). [5)] ITO/ TAPC (30 nm)/ TCTA (5 nm)/ mCP (5 nm)/ PPF: 20 wt% DPAc-DtCzBN: 2 wt% emitter (20 nm)/ PPF (5 nm)/ Bphen (30 nm)/ LiF (0.5 nm)/ Al (150 nm); [6)] PEDOT: PSS (40 nm)/TAPC (5 nm)/TCTA (5 nm)/PCZAC (5 nm)/mCP (5 nm)/mCP: TSPO1: emitter (25 nm, 50%: 1%)/TSPO1 (25 nm)/LiF (1.5 nm)/Al (200 nm); [7)] ITO (50 nm)/PEDOT: PSS (40 nm)/TAPC (10 nm)/mCP (10 nm)/mCP: mCBP-1CN: t3IDCz or p3IDCz (25 nm, 50%, 1 wt%)/TSPO1 (25 nm)/LiF (1.5 nm)/Al (200 nm); [8)] ITO (50 nm)/PEDOT: PSS (40 nm)/TAPC (10 nm)/mCP (10 nm)/mBisPCz-OBN: t3IDCz or p3IDCz (25 nm, 1 wt%)/TSPO1 (25 nm)/LiF (1.5 nm)/Al (200 nm); [9)] TO (50 nm)/PEDOT: PSS (40 nm)/TAPC (10 nm)/mCP (10 nm)/mBisPCz-OBN: 20 wt%DMAC-DPS: 1 wt% p3IDCz (25 nm)/TSPO1 (25 nm)/LiF (1.5 nm)/Al (200 nm); [10)] ITO/NPB (40 nm)/TSBPA (10 nm)/ 10%DiICzMes4: DPEPO (30 nm)/DPEPO (10 nm)|TBPi (40 nm)/LiF (1 nm)|Al (100 nm); [11)] ITO/NPB (40 nm)/TSBPA (10 nm)/ 35 wt% TADF: 1 wt %DiICzMes4: DPEPO (30 nm)/DPEPO (10 nm)|TBPi (40 nm)/LiF (1 nm)|Al (100 nm); [12)] ITO/HATCN (5 nm)/NPB (30 nm)/SFBCz (10 nm)/emissive layer (exciplex (SFBCz: SFTRZ=1: 1): 5 wt% Ir(mphmq)2tmd: 0.3 wt% α-NAICZ and exciplex (SFBCz: SFTRZ=1: 1): 30 wt% Ir(mphmq)2tmd: 0.3 wt% α-EtNAICZ, 30 nm)/ SFTRZ (10 nm)/DPPyA (30 nm)/LiF (0.5 nm)/Al; [13)] ITO/HATCN (5 nm)/ NPB (30 nm)/ BCzPh ( 10 nm)/ α,β-ADN: 1 wt% emitter (30 nm)/ CzPhPy (10 nm)/ DPPyA (20 nm)/LiF (0.5 nm)/Al (150 nm); [14)] ITO/TAPC (30 nm)/TCTA (5 nm)/mCP (5 nm)/ mCPB: 30 wt% m4TCzPhBN: 1 wt% emitter (30 nm)/PPF (5 nm)/Bphen (30 nm)/LiF (0.5 nm)/Al (150 nm); [15)] ITO (50 nm)/ NPB (75 nm)/ TCTA (5 nm)/ PCzAc (5 nm)/ mCP (5 nm)/50 wt% mCBP: 50 wt%TSPO1: 1 wt% or 3 wt% emitter (25 nm)/TSPO1 (5 nm)/TPBi ( 20 nm)/LiF (1.5 nm)/Al (200 nm); [16)] ITO (50 nm)/ PEDOT: PSS (40 nm)/ TAPC (5 nm)/ TCTA (5 nm)/ PCZAC (5 nm)/ *m*-CP (5 nm)/ *m*-CP: TSPO1: *m*-FLDID (25: 50%: X%)/ TSPO1 (25 nm)/ LiF (1.5 nm)/ Al (200 nm); [17)] ITO(135 nm)/HATCN (10 nm)/TAPC (30 nm)/TCTA (5 nm)/mCP (5 nm)/mCBP: 30 wt.% m4TCzPhBN: 1 wt.% Cz-DICz (30 nm)/PPF (5 nm)/Bphen (30 nm)/LiF (0.5 nm)/Al(150 nm); [18)] ITO(50 nm)/PEDOT: PSS (40 nm) / TAPC (5 nm) / TCTA (5 nm) / PCZAC (5 nm) / mCP (5 nm) / mCP: TSPO1: tBisICz-DPA or tBisICz-PhCz (25 nm: 50 wt%: 3wt%) / TSPO1 (25 nm) / LiF (1.5 nm)/Al(200 nm); [19)] ITO/HATCN (10 nm)/TAPC (60 nm)/TCTA (5 nm)/mCBP: 3wt.% Nm-ICz or PPT: 3wt.% CNm-ICz (20 nm)/TPBi (40 nm)/LiF (1 nm)/Al (120 nm); [20)] ITO / TAPC (50 nm)/ oCBP (10 nm)/ DMAC-DPS: 1 wt% NB-1 or DPAc-DCzBN: 1 wt%NB-2 (20 nm)/ TSPO1 (45 nm)/ LiF (1.5 nm)/ Al (200 nm); [21)] ITO/ HATCN (5 nm)/ TAPC (30 nm)/ TCTA (10 nm)/ 49.25 wt% SiCzCz: 49.25 wt% SiTrzCz2: 1.5 wt% IDCz-DBS (24 nm)/ CzPhPy (10 nm)/ DPPyA (30 nm)/LiF (0.7 nm)/Al(150 nm); [22)] ITO (95 nm)/ 3% HI-9:BPBPA (60 nm)/ BPBPA (30 nm)/ BCzPh (10 nm)/ mCBP (15 nm)/ 0.5% DIDCz-tBu:20 wt.% mMDBA-DI:PPF (25 nm)/ PPF (10 nm)/ ImAn-Na (30 nm)/ Liq (2 nm)/ Al (100 nm); [23)] ITO/HAT-CN (10 nm)/TAPC (40 nm)/TCTA (8 nm)/mCBP: 15 wt.% Firpic： 3wt.% emitter (20 nm)/ TmPyPB (40 nm)/Liq (2 nm)/Al.

**Table S7 Relevant chiroptical properties reported for the CP-MR-TADF emitters implemented in CP-OLEDs. The molecular structures are referred to Figure 34 in main text.**

| Enantiomer emitter | $\lambda_{PL}$[a] (nm) | $FWHM_{PL}$[b] (nm) | $g_{PL}$[c] ($\times 10^{-3}$) | $\lambda_{EL}$[d] (nm) | $FWHM_{EL}$[e] (nm) | $EQE_{max}$[f] (%) | $g_{EL}$[g] ($\times 10^{-3}$) | Ref. |
|---|---|---|---|---|---|---|---|---|
| (R/S)-OBN-2CN-BN | 493 | 22 | 0.9/ -0.91 (Tol) | 496 | 30 | 29.4/28.8 | 1.43/ -1.27 | 21 |
| (R/S)-OBN-4CN-BN | 500 | 27 | 0.8/ -1.04 (Tol) | 508 | 33 | 24.5/24.3 | 0.46/ -0.47 | |
| (R/S)-Czp-tBuCzB | 478 | 23 | 0.54/ -0.51 | 479 | 24 | 32.1/31.9 | 1.54/ -1.48 | 89 |
| (R/S)-Czp-POAB | 498 | 36 | 0.48/ -0.46 | 513 | 48 | 28.7/28.5 | 1.30/ -1.25 | |
| (R/S)-BA23CzBN | 490 | 27 | ±0.35 | 504/503 | | 36.6/34.6 | +0.31/-0.22 | 178 |
| (R/S)-BA34CzBN | 515 | 34 | ±0.77 | 528/526 | | 36.0/36.1 | +1.1/-1.2 | |
| (+)/(−)-BN4 | 522 | 50 | 1.1/ -1.0 | 512 | 96 | 20.6/19.0 | 3.7/ -3.1 | 35 |
| (+)/(−)-BN5 | 512 | 49 | 1.3/ -1.0 | 506 | 92 | 22.0/26.5 | 1.9/ -1.6 | |
| (P/M)-helicene-BN | 525 | 48 | 1.3/ -2.0 | 524/523 | 49/50 | 31.5/30.7 | 1.2/ -2.2 | 57 |
| (P/M)-DB-O | 443 | 24 | -0.8/ +1.1 | 445 | 24 | 26.2/27.5 | -0.22/ +0.22 | 132 |
| (P/M)-DB-S | 444 | 23 | -0.8/ +1.1 | 447 | 24 | 28.9/29.3 | -0.26/ +0.26 | |
| (P/M)-BN[9]H | 578 | 47 | -5.5/ 5.8 | 580 | 48 | 35.4 | -6.2/4.8 | 138 |
| (P/M)-BN-Py | 527 | 35 | -0.381/ 0.519 | 532 | 38 | 31.0 | -0.274/ +0.367 | 116 |
| (P/M)-BN-TPICz | 531 | 36 | 0.573/ -0.580 | 540 | 38 | 32.0 | 0.649/ -0.774 | 202 |
| H[6]BN1 | 472 | 30 | 0.426/-0.508 | 502 | 35 | 3.18 | | 214 |
| (M,M/P,P)-RBNN | 617 | 38 | 1.40/ -1.41 | 617 | 48 | 36.6 | 1.91/ -1.77 | 120 |
| tBuPh-BN () | 490 | 25 | 1.5/ -1.3 | 492 | 34 | 20.9 | | 294 |
| DPA-tBuPh-BN () | 477 | 28 | 0.9/ -0.8 | 480 | 38 | 15.9 | | |
| R-DOBN | 453 | 21 | 1.0 | 459 | 38 | 23.9 | -0.9 | 288 |
| R-DOBNT | 459 | 21 | 0.9 | 464 | 35 | 25.6 | -1.0 | |
| (R/S)-BDBF-BOH | 458 | 27 | -0.6/ 0.69 | 467 | 30 | 29.5 | +0.59/ -0.59 | 181 |
| R/S)-BDBT-BOH | 459 | 27 | -0.9/+1.0 | 467 | 30 | 30.1 | +1.2/ -1.1 | |
| (R/S)-S-AX-BN | 489 | 21 | +3.5/ -3.3 | 495 | 22 | 33.5 | +3.3/ -3.2 | 201 |
| (R/S)-SO2-AX-BN | 495 | 20 | +2.3/ -2.2 | 500 | 21 | 31.5 | +2.2/ -2.1 | |
| (R/S)-BN-MeIAc | 497 | 30 | 0.25/ -0.25 | 504/503 | 33 | 37.2/36.1 | 0.27/ -0.29 | 59 |
| (R/S)-4-POtBuCzB | 493 | 20 | 0.40/ -0.36 | 491 | 25 | 31.3 | 0.60/ -0.61 | 203 |

| | | | | | | | | |
|---|---|---|---|---|---|---|---|---|
| (R/S)-SFDBN-CN | 482 | 14 | 0.66 | 483 | 16 | 30.0/30.7 | -0.26/ +0.22 | 209 |
| (Rmt)-2 | 496 | 37 | -16 | 500 | 36 | 15.6 | -2.0 | 190 |
| (Smt)-2 | 496 | 37 | +11 | 500 | 36 | 17.6 | +2.1 | |
| (P/M)-QAO-PhCz | 461 | 29 | 1.1/ -0.92 | 467 | 36 | 14.0 | 1.5/ - | 231 |
| (P/M)-QPO-PhCz | 446 | 58 | -1.1/ 1.6 | 488 | 56 | 10.6 | -1.1/ 1.6 | 240 |

[a] photoluminescence peak wavelength; [b] full width at half maximum (FWHM) of photoluminescence; [c] photoluminescence dissymmetry factors ($g_{PL}$); [d] electroluminescence (EL) peak; [e] full width at half maximum (FWHM) of electroluminescence (EL); [f] maximum external quantum efficiency (EQE) for enantiomer emitters; [g] electroluminescence dissymmetry factors ($g_{EL}$).

**Table S8** Summary of the representative **deep-blue** OLEDs performance employing MR-TADF emitters ($CIE_y \leq 0.10$).

| Emitter | $\lambda_{PL}$ (nm) | $FWHM_{PL}$ (nm) | $\lambda_{EL}$ [nm] | $FWHM_{EL}$ [nm] | EQE [%] Max/100/1000 | CIE (x, y) | Ref. |
|---|---|---|---|---|---|---|---|
| V-DABNA-F | 464 | 16 | 468 | 15 | 26.6/25.8/23.4 | 0.12, 0,10 | 179 |
| ν-DABNA-O-Me | 464 | 24 | 465 | 23 | 29.5/28.8/26.9 | 0.13, 0.10 | 24 |
| DABNA-1 | 462 | 30 | 459 | 28 | 13.5/6.0/- | 0.I3, 0.09 | 1 |
| PAB | 453 | 23 | 456 | 31 | 14.7 | 0.145,0.076 | 28 |
| 2tPAB | 457 | 26 | 456 | 27 | 16.8 | 0.145,0.076 | |
| 3tPAB | 458 | 23 | 460 | 26 | 19.3 | 0.141,0.076 | |
| BOBO-Z | 441 | 15 | 445 | l8 | 13.6/9.8/3.3 | 0.15, 0.04 | 43 |
| BOBS-Z | 453 | 21 | 456 | 23 | 26.9/24.0/15.0 | 0.14, 0.06 | |
| BSBS-Z | 460 | 20 | 463 | 22 | 26.8/24.0/15.9 | 0.13, 0.08 | |
| CzBO | 445 | 26 | 448 | 30 | 13.4/8.4/3.5 | 0.15, 0.05 | 64 |
| tDPAC-BN | 460 | 26 | 460 | 28 | 21.6/15.3/5.4 | 0.135, 0.094 | 65 |
| BIC-mCz | 432 | 42 | 432 | 42 | 19.4 | 0.16, 0.05 | 68 |
| mDBIC | 431 | 42 | 431 | 42 | 13.5 | 0.16, 0.05 | |
| asym-BN1 | 454 | 18 | 457 | 28 | 31.2/18.3/9.3 | 0.14, 0.08 | 60 |
| sym-BN3 | 456 | 17 | 458 | 23 | 37.6/34.0/26.2 | 0.14, 0.08 | |
| t-DABNA | 458 | 22 | 464 | 26 | 28.4/14.8/4.4 | 0.13, 0.10 | 295 |
| t-DAB-DPA | 446 | 18 | 459 | 26 | 27.6/21.8/9.2 | 0.14, 0.08 | |
| 4F-ν-DABNA | 457 | 14 | 464 | 18 | 35.8/-/- | 0.13, 0.08 | 44 |
| 4F-m-ν-DABNA | 455 | 14 | 461 | 18 | 33.7/-/- | 0.13, 0.06 | |
| DPACzBN2 | 470 | 29 | 469 | 28 | 24.0/23.5/14.3 | 0.13, 0.10 | 296 |
| BFCz-DABNA | 456 | 22 | 463 | 26 | 28.0/-/- | 0.13, 0.09 | 84 |
| CzCO | 430 | 32 | 432 | 35 | 15.6/-/- | 0.157, 0.071 | 242 |
| Cz2CO | 440 | 16 | 445 | 23 | 13.0/-/- | 0.154, 0.051 | |
| C-BN | 450 | 19 | 453 | 25 | 20.1/13.8/6.0 | 0.14, 0,07 | 85 |
| NOBNacene | 410 | 38 | 409 | 37 | 8.5/-/- | 0.173, 0.055 | 105 |
| ν-DABNA-O-xy | 446.7 | 13.8 | 460 | 19 | 27.5/-/16.2 | 0.14, 0.07 | 297 |
| TPD4PA | 445 | 19 | 455 | 29 | 30.7/30.6/17.8 | 0.14, 0.06 | 70 |
| tBu-TPD4PA | 451 | 19 | 460 | 29 | 32.5/30.9/25 | 0.14, 0.07 | |
| *f*-DOABNA | 444 | 25 | 445 | 24 | 19.5/15.6/7.5 | 0.150, 0.041 | 183 |
| DOB2-DABNA-A | 451 | 27 | 452 | 24 | 24.1/23.3/21.6 | 0.145, 0.049 | 185 |

| | | | | | | | |
|---|---|---|---|---|---|---|---|
| DPA-B2 | 444 | 31 | 444 | 31 | 28.9/19.8/9.3 | 0.153, 0.055 | 187 |
| DPA-B3 | 451 | 16 | 450 | 15 | 37.7/33.721.0 | 0.150, 0.043 | |
| DPA-B4 | 458 | 14 | 457 | 14 | 39.2/36.4/28.7 | 0.141, 0.050 | |
| Cz-B4 | 457 | 28 | 457 | 26 | 32.1/26.9/16.1 | 0.138, 0.076 | |
| DABNA-NP-TB | 453 | 26 | 458 | | 7.03/-7.00 | 0.136,0.076 | 287 |
| | | | 458 | | 6.05/-/6.00 | 0.136,0.076 | |
| BSS-Cz | 455 | 28 | 462 | 29 | 21.8/15.6/- | 0.13,0.09 | 79 |
| pBP-DABNA-Me | 462 | 22 | 464 | 23 | 23.4 | 0.13,0.092 | 87 |
| [B-N]N | 442 | 19 | 448 | 27.3 | 16.7/-/7.6 | 0.151,0.079 | 102 |
| $\alpha$-3BNMes | 442 | 30 | 443 | 49 | 14.6/10.2/- | 0.15,0.10 | 104 |
| m-DiNBO | 450 | 17 | 466 | 21 | 24.2/17.3/- | 0.126,0.098 | 110 |
| OBN | 425 | 30 | 437 | 44 | 23.02 | 0.15,0.09 | 117 |
| NBN | 440 | 29 | 452 | 40 | 15.7 | 0.14,0.09 | |
| ODBN | 429 | 26 | 446 | 54 | 24.5 | 0.15,0.10 | |
| B-N-1 | 455 | 32 | 457 | 38 | | 0.135,0.1004.9 | 141 |
| BuDABNA | 472 | 26 | 460 | 27 | 25.1/12.7/- | 0.137,0.093 | 145 |
| tBOSi | 414 | 28 | 414 | 32 | 9.15 | 0.165,0.034 | 150 |
| tBOSiCz | 414 | 28 | 414 | 32 | 8.91 | 0.163,0.031 | |
| A-BN | 455 | 22 | 462 | 24 | 41.5/23.6/10.4 | 0.13,0.08 | 173 |
| | | | 462 | 25 | 38.5/32.7/25.1 | 0.13,0.09 | |
| ν-DABNA-Az1 | 458 | 19 | 459 | 19 | 30.8/29.0/19.9 | 0.136,0.083 | 174 |
| ν-DABNA-Az2 | 458 | 19 | 458 | 17 | 29.9/28.0/16.0 | 0.140,0.060 | |
| ν-DABNA-Az3 | 458 | 19 | 459 | 20 | 33.0/29.2/22.4 | 0.136,0.100 | |
| 5Cz-BO | 414 | 28.9 | 416 | 36 | 22.8 | 0.163,0.046 | 175 |
| Py-BN | 446 | 23 | 444 | 21 | 15.8/7.4/3.9 | 0.153,0.045 | 184 |
| Pm-BN | 414 | 20 | 415 | 24 | 5.8/4.3/2.5 | 0.161,0.045 | |
| Py-BN | 446 | 23 | 445 | 22 | 27.7/25.0/16.1 | 0.150,0.052 | |
| QB-U | 450 | 21 | 453 | 23/0.14 | 24.7/-/11.5 | 0.142, 0.064 | 186 |
| QB-J | 454 | 15 | 457 | 17/0.10 | 30.8/-/20.9 | 0.142, 0.064 | |
| QB-I | 464 | 31 | 466 | 15/0.08 | 32.5/-/30.4 | 0.127, 0.078 | |
| iPrAuBN | 448 | 29 | 442 | 19 | 14.8 | 0.154,0.036 | 210 |
| ICz-BO | 413 | 34 | 414 | 37 | 12.014 | 0.164,0.031 | 211 |
| 2FPAB | 430 | 22 | 436 | 35 | 4.2 | 0.157, 0.044 | 213 |

| | | | | | | | |
|---|---|---|---|---|---|---|---|
| MePAB | 446 | 22 | 448 | 29 | 19.8 | 0.146, 0.046 | |
| [B-N]N2 | 438 | 16 | 441 | 20 | 20.3 | 0.152,0.046 | 222 |

**Table S9**. Summary of reported green MR-TADF OLED with CIEy ⩾ 0.65.

| Emitter | $\lambda_{PL}$ (nm) | $FWHM_{PL}$ (nm) | $\lambda_{EL}$ (nm) | $FWHM_{EL}$ (nm) | EQE (%) | CIE (x,y) | Ref. |
|---|---|---|---|---|---|---|---|
| CNBN | 501 | 14 | 506 | 16 | 34.4 | 0.12, 0.68 | 218 |
| MCNBN | 510 | 15 | 517 | 17 | 30.8 | 0.17, 0.74 | |
| PCNBN | 503 | 18 | 508 | 20 | 29.1 | 0.14, 0.67 | |
| PMCNBN | 512 | 19 | 519 | 22 | 27.2 | 0.19, 0.73 | |
| AZA-BN | 522 | 28 | 528 | 31 | 25.7 | 0.28, 0.69 | 10 |
| m-Cz-BNCz | 519 | 38 | 520 | 44 | 27.0 | 0.23, 0.69 | 11 |
| BBCz-G | 517 | 34 | 515 | 54 | 31.8 | 0.26, 0.68 | 13 |
| PXZ-BN | 502 | 38 | 516 | 47 | 23.2 | 0.22, 0.67 | 26 |
| TCz-B | 512 | 27 | 515 | 30 | 29.2 | 0.16, 0.71 | 30 |
| TCz-VTCzBN | 521 | 29 | 524 | 37 | 32.2 | 0.22, 0.71 | 77 |
| BN-ICz-1 | 521 | 21 | 523 | 23 | 30.5 | 0.22, 0.74 | 58 |
| BN-ICz-2 | 521 | 22 | 523 | 23 | 29.8 | 0.23, 0.73 | |
| DtCzB-TPTRZ | 521 | 24 | 520 | 41 | 30.6 | 0.23, 0.68 | 31 |
| (SIPr)AuBN | 511 | 30 | 511 | 40 | 24.8 | 0.20, 0.69 | 74 |
| (BzIPr)AuBN | 511 | 30 | 513 | 34 | 24.8 | 0.18, 0.70 | |
| (PyIPr)AuBN | 511 | 30 | 516 | 38 | 27.3 | 0.19, 0.70 | |
| (PzIPr)AuBN | 510 | 30 | 515 | 39 | 24.0 | 0.22, 0.67 | |
| TRZCzPh-BNCz | 514 | 34 | 513 | 37 | 32.5 | 0.17, 0.68 | 76 |
| TRZTPh-BNCz | 513 | 29 | 513 | 33 | 31.4 | 0.16, 0.70 | |
| BN-TP | 523 | 34 | 528 | 36 | 35.1 | 0.26, 0.70 | 61 |
| DBNO | 500 | 19 | 512 | 41 | 33.1 | 0.22, 0.67 | 55 |
| tCzphB-Ph | 523 | 21 | 527 | 24 | 29.3 | 0.21, 0.75 | 71 |
| tCzphB-Fl | 531 | 21 | 535 | 26 | 26.2 | 0.26, 0.72 | |
| ν-DABNA-CN-Me | 496 | 17 | 504 | 23 | 31.9 | 0.13, 0.65 | 69 |
| BN-STO | 517 | 34 | 517 | 34 | 40.1 | 0.19, 0.70 | 100 |
| BN-XTO | 515 | 33 | 516 | 34 | 37.3 | 0.19, 0.70 | |
| ω-DABNA | 509 | 22 | 512 | 25 | 31.1 | 0.13, 0.73 | 94 |
| DBNT-2 | 512 | 20 | 520 | 29 | 35.2 | 0.19, 0.74 | 103 |
| DCzBN-Au | 508 | 39 | 510 | 34 | 35.8 | 0.16, 0.67 | 223 |
| BN-TP-N1 | 526 | 33 | 532 | 34 | 34.9 | 0.28, 0.69 | 126 |

| | | | | | | | |
|---|---|---|---|---|---|---|---|
| BN-TP-N2 | 528 | 33 | 534 | 36 | 31.9 | 0.30, 0.67 | |
| BN-TP-N3 | 519 | 32 | 524 | 33 | 37.3 | 0.23, 0.71 | |
| BN-TP-N4 | 520 | 32 | 528 | 35 | 36.5 | 0.27, 0.70 | |
| DBF-DBN | 514 | 22 | 521 | 31 | 21.5 | 0.22, 0.71 | 298 |
| DBT-DBN | 516 | 19 | 520 | 24 | 31.3 | 0.22, 0.70 | |
| MesPXZ-tCzBN | 514 | 41 | 528 | 54 | 27.6 | 0.31, 0.65 | 299 |
| BNO | 504 | 36 | 520 | 55 | 19.5 | 0.26, 0.66 | 300 |
| SBNO | 514 | 30 | 524 | 40 | 25.7 | 0.26, 0.68 | |
| SBNOS | 514 | 30 | 522 | 40 | 32.7 | 0.25, 0.69 | |
| 2SBN | 503 | 29 | 514 | 45 | 27.1 | 0.20, 0.65 | 301 |
| 3SBN | 509 | 29 | 514 | 43 | 27.0 | 0.20, 0.66 | |
| 6z | 516 | 22 | 513 | 27 | 31.7 | 0.18, 0.70 | 139 |
| BpIC-DPA | 527 | 25 | 536 | 29 | 22.0 | 0.30, 0.67 | 180 |
| TCzBAO | 507 | 30 | 520 | 36 | 25.1 | 0.20, 0.70 | 134 |
| Spiro-BNCz | 528 | 42 | 533 | 48 | 34.2 | 0.29, 0.67 | 302 |
| BThPAc-1 | 508 | 30 | 517 | 34.5 | 22.0 | 0.181, 0.721 | 303 |
| BThPAc-2 | 509 | 30 | 517 | 32.9 | 20.3 | 0.176, 0.723 | |
| *m*-ICz-BNCz | 508 | 38 | 516 | 40 | 28.0 | 0.20, 0.70 | 304 |
| *dm*-ICz-BNCz | 526 | 41 | 534 | 46 | 32.9 | 0.30, 0.67 | |
| DPhCz-SFBN | 516 | 26 | 522 | 37 | 31.0 | 0.22, 0.70 | 305 |
| DCz-SFBN | 503 | 33 | 506 | 37 | 29.2 | 0.15, 0.65 | |
| DPhCz-SFBN | 516 | 26 | 522 | 37 | 31.0 | 0.22, 0.70 | |
| CzTCz-SFBN | 515 | 28 | 518 | 35 | 28.1 | 0.20, 0.71 | |
| QB-DPA | 495 | 15 | 502 | 17 | 36.0 | 0.10, 0.61 | 205 |
| QB-PXZ | 508 | 15 | 516 | 20 | 36.6 | 0.18, 0.74 | |

**Table S10.** Summary of reported MR-TADF with $\lambda_{PL}$ > 600 nm

| Emitter | $\lambda_{PL}$ (nm) | $FWHM_{PL}$ (nm) | $\lambda_{EL}$ (nm) | $FWHM_{EL}$ (nm) | EQE (%) | CIE (x,y) | Ref. |
|---|---|---|---|---|---|---|---|
| BBCz-R | 615 | 21 | 616 | 26 | 22.0/-/- | 0.67,0.33 | 13 |
| R-BN | 662 | 38 | 664 | 48 | 28.4/-/- | 0.719,0.280 | 17 |
| R-TBN | 692 | 41 | 686 | 49 | 28.1/-/- | 0.721,0.278 | |
| BP-2DPA | 601 | 37 | 605 | 34 | 11.3/8.7/5.3 | 0.62,0.38 | 41 |
| DBP-4DPA | 612 | 35 | 617 | 34 | 15.1/11.2/7.0 | 0.63,0.36 | |
| BNO1 | 605 | 32 | 610 | 39 | 35.6/31.1/27.7 | 0.64, 0.34 | 63 |
| BNO2 | 609 | 32 | 618 | 39 | 34.4/29.8/26.9 | 0.65, 0.35 | |
| BNO3 | 616 | 33 | 625 | 40 | 36.1/32.1/28.6 | 0.66, 0.34 | |
| PPZ-BN | 613 | 48 | 613 | 48 | 26.9/-/26.0 | 0.66,0.34 | 127 |
| BNNO | 637 | 32 | 643 | 42 | 34.4/-/31.4 | 0.708,0.292 | 101 |
| mBDPA-TOAT | 599 | 37 | 600 | 45 | 17.3 | 0.61,0.39 | 229 |
| pBDPA-TOAT | 603 | 48 | 624 | 62 | 11.3 | 0.66,0.34 | |
| 3DPA-DiKTa | 617 | 56 | 613 | 60 | 16.7/3.4/1.9 | 0.633,0.365 | 254 |
| RBNO1 | 629 | 39 | 632 | 55 | 33.1/-/22.5 | 0.689,0.311 | 155 |
| RBNO2 | 645 | 39 | 645 | 48 | 34.7/-/28.8 | 0.700,0.300 | |
| PTZBNO | 612 | 47 | 618 | 58 | 34.5/-/29.7 | 0.64,0.36 | 148 |
| PXZBNO | 627 | 45 | 632 | 54 | 28.1/-/23.8 | 0.67,0.33 | |
| CzIDBNO | 633 | 38 | 643 | 47 | 32,5/-/23.8 | 0.701,0.298 | 172 |
| IDIDBNO | 663 | 39 | 671 | 49 | 27.2/-/21.1 | 0.702,0.297 | |
| DMAC-TOAT | 656 | 105 | 616 | 104 | 1.5 | 0.59,0.39 | 229 |

**Reference**

1. Hatakeyama, T.; Shiren, K.; Nakajima, K.; Nomura, S.; Nakatsuka, S.; Kinoshita, K.; Ni, J.; Ono, Y.; Ikuta, T., Ultrapure blue thermally activated delayed fluorescence molecules: efficient HOMO-LUMO separation by the multiple resonance effect. *Adv. Mater.* **2016,** *28* (14), 2777-2781.
2. Nakatsuka, S.; Gotoh, H.; Kinoshita, K.; Yasuda, N.; Hatakeyama, T., Divergent synthesis of heteroatom-centered 4,8,12-triazatriangulenes. *Angew. Chem. Int. Ed. Engl.* **2017,** *56* (18), 5087-5090.
3. Matsui, K.; Oda, S.; Yoshiura, K.; Nakajima, K.; Yasuda, N.; Hatakeyama, T., One-shot multiple borylation toward BN-doped nanographenes. *J. Am. Chem. Soc.* **2018,** *140* (4), 1195-1198.
4. Liang, X.; Yan, Z. P.; Han, H. B.; Wu, Z. G.; Zheng, Y. X.; Meng, H.; Zuo, J. L.; Huang, W., Peripheral amplification of multi-resonance induced thermally activated delayed fluorescence for highly efficient OLEDs. *Angew. Chem. Int. Ed. Engl.* **2018,** *57* (35), 11316-11320.
5. Oda, S.; Kawakami, B.; Kawasumi, R.; Okita, R.; Hatakeyama, T., Multiple resonance effect-induced sky-blue thermally activated delayed fluorescence with a narrow emission band. *Org. Lett.* **2019,** *21* (23), 9311-9314.
6. Han, S. H.; Jeong, J. H.; Yoo, J. W.; Lee, J. Y., Ideal blue thermally activated delayed fluorescence emission assisted by a thermally activated delayed fluorescence assistant dopant through a fast reverse intersystem crossing mediated cascade energy transfer process. *J. Mater. Chem. C* **2019,** *7* (10), 3082-3089.
7. Zhang, Y.; Zhang, D.; Wei, J.; Liu, Z.; Lu, Y.; Duan, L., Multi-resonance induced thermally activated delayed fluorophores for narrowband green OLEDs. *Angew. Chem. Int. Ed. Engl* **2019,** *58* (47), 16912-16917.
8. Kondo, Y.; Yoshiura, K.; Kitera, S.; Nishi, H.; Oda, S.; Gotoh, H.; Sasada, Y.; Yanai, M.; Hatakeyama, T., Narrowband deep-blue organic light-emitting diode featuring an organoboron-based emitter. *Nat. Photonics* **2019,** *13* (10), 678-682.
9. Ikeda, N.; Oda, S.; Matsumoto, R.; Yoshioka, M.; Fukushima, D.; Yoshiura, K.; Yasuda, N.; Hatakeyama, T., Solution-processable pure green thermally activated delayed fluorescence emitter based on the multiple resonance effect. *Adv. Mater.* **2020,** *32* (40), 2004072.
10. Zhang, Y.; Zhang, D.; Wei, J.; Hong, X.; Lu, Y.; Hu, D.; Li, G.; Liu, Z.; Chen, Y.; Duan, L., Achieving pure green electroluminescence with ciey of 0.69 and eqe of 28.2% from an aza-fused multi-resonance emitter. *Angew. Chem. Int. Ed. Engl* **2020,** *59* (40), 17499-17503.
11. Wang, Y.; Xu, Y.; Li, C.; Li, Z.; Wang, Q.; Cai, X.; Wei, J., Constructing charge transfer excited state based on frontier molecular orbital engineering: narrowband green electroluminescence with high color purity and efficiency. *Angew. Chem. Int. Ed. Engl.* **2020,** *59*, 17442-17446.
12. Xu, Y.; Cheng, Z.; Li, Z.; Liang, B.; Wang, J.; Wei, J.; Zhang, Z.; Wang, Y., Molecular-structure and device - configuration optimizations toward highly efficient green electroluminescence with narrowband emission and high color purity. *Adv. Opt. Mater.* **2020,** *8* (9), e1902142.
13. Yang, M.; Park, I. S.; Yasuda, T., Full-color, narrowband, and high-efficiency electroluminescence from boron and carbazole embedded polycyclic heteroaromatics. *J. Am. Chem. Soc.* **2020,** *142* (46), 19468-19472.
14. Knoller, J. A.; Meng, G.; Wang, X.; Hall, D.; Pershin, A.; Beljonne, D.; Olivier, Y.; Laschat, S.; Zysman-Colman, E.; Wang, S., Intramolecular borylation via sequential b-mes bond cleavage for the divergent synthesis of B,N,B-doped benzo[4]helicenes. *Angew. Chem. Int. Ed. Engl.* **2020,** *59* (8), 3156-3160.
15. Suresh, S. M.; Duda, E.; Hall, D.; Yao, Z.; Bagnich, S.; Slawin, A. M. Z.; Bassler, H.; Beljonne, D.; Buck, M.; Olivier, Y.; Kohler, A.; Zysman-Colman, E., A deep blue B,N-doped heptacene emitter that shows both thermally activated delayed fluorescence and delayed fluorescence by triplet-triplet annihilation. *J. Am. Chem. Soc.* **2020,** *142* (14), 6588-6599.
16. Jiang, P.; Zhan, L.; Cao, X.; Lv, X.; Gong, S.; Chen, Z.; Zhou, C.; Huang, Z.; Ni, F.; Zou, Y.; Yang, C., Simple acridan - based multi - resonance structures enable highly efficient narrowband green TADF electroluminescence. *Adv. Opt. Mater.* **2021,** *9* (21), 2100825.
17. Zhang, Y.; Zhang, D.; Huang, T.; Gillett, A. J.; Liu, Y.; Hu, D.; Cui, L.; Bin, Z.; Li, G.; Wei, J.; Duan, L., Multi-resonance deep-red emitters with shallow potential-energy surfaces to surpass energy-gap law*. *Angew. Chem. Int. Ed. Engl.* **2021,** *60* (37), 20498-20503.
18. Oda, S.; Kumano, W.; Hama, T.; Kawasumi, R.; Yoshiura, K.; Hatakeyama, T., Carbazole-based DABNA analogues as highly efficient thermally activated delayed fluorescence materials for narrowband organic light-emitting diodes. *Angew. Chem. Int. Ed. Engl.* **2021,** *60* (6), 2882-2886.
19. Hua, T.; Zhan, L.; Li, N.; Huang, Z.; Cao, X.; Xiao, Z.; Gong, S.; Zhou, C.; Zhong, C.; Yang, C., Heavy-atom effect promotes multi-resonance thermally activated delayed fluorescence. *Chem. Eng. J.* **2021,** *426*, 131169.
20. Hu, Y. X.; Miao, J.; Hua, T.; Huang, Z.; Qi, Y.; Zou, Y.; Qiu, Y.; Xia, H.; Liu, H.; Cao, X.; Yang, C., Efficient selenium-integrated TADF OLEDs with reduced roll-off. *Nat. Photonics* **2022,** *16*, 803-810.
21. Xu, Y.; Wang, Q.; Cai, X.; Li, C.; Wang, Y., Highly efficient electroluminescence from narrowband green circularly polarized multiple resonance thermally activated delayed fluorescence enantiomers. *Adv. Mater.* **2021,** *33* (21), e2100652.
22. Chen, F.; Zhao, L.; Wang, X.; Yang, Q.; Li, W.; Tian, H.; Shao, S.; Wang, L.; Jing, X.; Wang, F., Novel boron- and sulfur-doped polycyclic aromatic hydrocarbon as multiple resonance emitter for ultrapure blue

thermally activated delayed fluorescence polymers. *Sci. China Chem.* **2021,** *64*, 547-551.
23. Xu, S.; Yang, Q.; Zhang, Y.; Li, H.; Xue, Q.; Xie, G.; Gu, M.; Jin, J.; Huang, L.; Chen, R., Solution-processed multi-resonance organic light-emitting diodes with high efficiency and narrowband emission. *Chin. Chem. Lett.* **2020,** *32* (4), 1372-1376.
24. Tanaka, H.; Oda, S.; Ricci, G.; Gotoh, H.; Tabata, K.; Kawasumi, R.; Beljonne, D.; Olivier, Y.; Hatakeyama, T., Hypsochromic shift of multiple-resonance-induced thermally activated delayed fluorescence by oxygen atom incorporation. *Angew. Chem. Int. Ed. Engl.* **2021,** *60* (33), 17910-17914.
25. Qi, Y.; Ning, W.; Zou, Y.; Cao, X.; Gong, S.; Yang, C., Peripheral decoration of multi-resonance molecules as a versatile approach for simultaneous long-wavelength and narrowband emission. *Adv. Funct. Mater.* **2021,** *31* (29), e2102017.
26. Liu, G.; Sasabe, H.; Kumada, K.; Matsunaga, A.; Katagiri, H.; Kido, J., Facile synthesis of multi-resonance ultra-pure-green TADF emitters based on bridged diarylamine derivatives for efficient OLEDs with narrow emission. *J. Mater. Chem. C* **2021,** *9* (26), 8308-8313.
27. Liu, Y.; Xiao, X.; Ran, Y.; Bin, Z.; You, J., Molecular design of thermally activated delayed fluorescent emitters for narrowband orange-red OLEDs boosted by a cyano-functionalization strategy. *Chem. Sci.* **2021,** *12* (27), 9408-9412.
28. Wang, Y.; Duan, Y.; Guo, R.; Ye, S.; Di, K.; Zhang, W.; Zhuang, S.; Wang, L., A periphery cladding strategy to improve the performance of narrowband emitters, achieving deep-blue OLEDs with CIEy < 0.08 and external quantum efficiency approaching 20%. *Org. Electron.* **2021,** *97*, 106275.
29. Nagata, M.; Min, H.; Watanabe, E.; Fukumoto, H.; Mizuhata, Y.; Tokitoh, N.; Agou, T.; Yasuda, T., Fused-nonacyclic multi-resonance delayed fluorescence emitter based on ladder-thiaborin exhibiting narrowband sky-blue emission with accelerated reverse intersystem crossing. *Angew. Chem. In. Ed. Engl.* **2021,** *60* (37), 20280-20285.
30. Yang, M.; Shikita, S.; Min, H.; Park, I. S.; Shibata, H.; Amanokura, N.; Yasuda, T., Wide-range color tuning of narrowband emission in multi-resonance organoboron delayed fluorescence materials through rational imine/amine functionalization. *Angew. Chem. Int. Ed. Engl.* **2021,** *60* (43), 23142-23147.
31. Xu, Y.; Li, C.; Li, Z.; Wang, J.; Xue, J.; Wang, Q.; Cai, X.; Wang, Y., Highly efficient electroluminescent materials with high color purity based on strong acceptor attachment onto B-N-containing multiple resonance frameworks. *CCS Chem.* **2022,** *4* (6), 2065-2079.
32. Park, J.; Lim, J.; Lee, J. H.; Jang, B.; Han, J. H.; Yoon, S. S.; Lee, J. Y., Asymmetric blue multiresonance TADF emitters with a narrow emission band. *ACS Appl. Mater. Interfaces* **2021,** *13* (38), 45798-45805.
33. Zhang, Y.; Wei, J.; Zhang, D.; Yin, C.; Li, G.; Liu, Z.; Jia, X.; Qiao, J.; Duan, L., Sterically wrapped multiple resonance fluorophors for suppression of concentration quenching and spectrum broadening. *Angew. Chem. Int. Ed.* **2021,** *61* (2), e202113206.
34. Jiang, P.; Miao, J.; Cao, X.; Xia, H.; Pan, K.; Hua, T.; Lv, X.; Huang, Z.; Zou, Y.; Yang, C., Quenching-resistant multiresonance TADF emitter realizes 40% external quantum efficiency in narrowband electroluminescence at high doping level. *Adv. Mater.* **2021,** *34* (3), e2106954.
35. Wu, X.; Huang, J. W.; Su, B. K.; Wang, S.; Yuan, L.; Zheng, W. Q.; Zhang, H.; Zheng, Y. X.; Zhu, W.; Chou, P. T., Fabrication of circularly polarized mr-tadf emitters with asymmetrical peripheral-lock enhancing helical B/N-doped nanographenes. *Adv. Mater.* **2021,** *34* (1), e2105080.
36. Li, J. K.; Chen, X. Y.; Guo, Y. L.; Wang, X. C.; Sue, A. C.; Cao, X. Y.; Wang, X. Y., B,N-embedded double hetero[7]helicenes with strong chiroptical responses in the visible light region. *J. Am. Chem. Soc.* **2021,** *143* (43), 17958-17963.
37. Lee, Y. T.; Chan, C. Y.; Tanaka, M.; Mamada, M.; Balijapalli, U.; Tsuchiya, Y.; Nakanotani, H.; Hatakeyama, T.; Adachi, C., Investigating HOMO energy levels of terminal emitters for realizing high-brightness and stable TADF-assisted fluorescence organic light-emitting diodes. *Adv. Electron. Mater.* **2021,** *7* (4), e2001090.
38. Han, J.; Huang, Z.; Lv, X.; Miao, J.; Qiu, Y.; Cao, X.; Yang, C., Simple molecular design strategy for multiresonance induced TADF emitter: highly efficient deep blue to blue electroluminescence with high color purity. *Adv. Opt. Mater.* **2021,** *10* (4), e2102092.
39. Liu, F.; Cheng, Z.; Wan, L.; Feng, Z.; Liu, H.; Jin, H.; Gao, L.; Lu, P.; Yang, W., Highly efficient multi-resonance thermally activated delayed fluorescence material with a narrow full width at half-maximum of 0.14 eV. *Small* **2021,** *18* (4), e2106462.
40. Qiu, Y.; H. X.; Miao, J.; Huang, Z.; Li, N.; Cao, X.; Han, J.; Zhou, C.; Zhong, C.; Yang, C., Narrowing the electroluminescence spectra of the multi-resonance emitters for high-performance blue OLEDs by a peripheral-decoration strategy. *ACS Appl. Mater. Interfaces* **2021,** *13* (49), 59035-59042.
41. Naveen, K. R.; Hwang, S. J.; Lee, H.; Kwon, J. H., Narrow band red emission fluorophore with reasonable multiple resonance effect. *Adv. Electron. Mater.* **2021,** *8* (3), e2101114.
42. Oda, S.; Kawakami, B.; Yamasaki, Y.; Matsumoto, R.; Yoshioka, M.; Fukushima, D.; Nakatsuka, S.; Hatakeyama, T., One-shot synthesis of expanded heterohelicene exhibiting narrowband thermally activated delayed fluorescence. *J. Am. Chem. Soc.* **2021,** *144* (1), 106-112
43. Park, I. S.; Yang, M.; Shibata, H.; Amanokura, N.; Yasuda, T., Achieving ultimate narrowband and

ultrapure blue organic light-emitting diodes based on polycyclo-heteraborin multi-resonance delayed fluorescence emitters. *Adv. Mater.* **2022,** *34* (9), e2107951.
44. Rayappa Naveen, K.; Lee, H.; Braveenth, R.; Joon Yang, K.; Jae Hwang, S.; Hyuk Kwon, J., Deep blue diboron embedded multi-resonance thermally activated delayed fluorescence emitters for narrowband organic light emitting diodes. *Chem. Eng. J.* **2022,** *432*, 134381.
45. Hu, J.-J.; Luo, X.-F.; Zhang, Y.-P.; Mao, M.-X.; Ni, H.-X.; Liang, X.; Zheng, Y.-X., Green multi-resonance thermally activated delayed fluorescence emitters containing phenoxazine units with highly efficient electroluminescence. *J. Mater. Chem. C* **2022,** *10* (2), 768-773.
46. Li, Q.; Wu, Y.; Wang, X.; Yang, Q.; Hu, J.; Zhong, R.; Shao, S.; Wang, L., Boron-, sulfur- and nitrogen-doped polycyclic aromatic hydrocarbon multiple resonance emitters for narrow-band blue emission. *Chem.* **2022,** *28* (12), e202104214.
47. Meng, G.; Liu, L.; He, Z.; Hall, D.; Wang, X.; Peng, T.; Yin, X.; Chen, P.; Beljonne, D.; Olivier, Y.; Zysman-Colman, E.; Wang, N.; Wang, S., Multi-resonant thermally activated delayed fluorescence emitters based on tetracoordinate boron-containing PAHs: colour tuning based on the nature of chelates. *Chem. Sci.* **2022,** *13* (6), 1665-1674.
48. Liu, F.; Cheng, Z.; Jiang, Y.; Gao, L.; Liu, H.; Liu, H.; Feng, Z.; Lu, P.; Yang, W., Highly Efficient asymmetric multiple resonance thermally activated delayed fluorescence emitter with eqe of 32.8 % and extremely low efficiency roll-off. *Angew. Chem. Int. Ed.* **2022,** *61* (14), e202116927.
49. Xiong, X.; Cheng, Y.-C.; Wang, K.; Yu, J.; Zhang, X.-H., A comparative study of two multi-resonance TADF analogous materials integrating chalcogen atoms of different periods. *Mater. Chem. Front.* **2023,** *7* (5), 929-936.
50. Cheon, H. J.; Shin, Y. S.; Park, N. H.; Lee, J. H.; Kim, Y. H., Boron-based multi-resonance TADF emitter with suppressed intermolecular interaction and isomer formation for efficient pure blue OLEDs. *Small* **2022,** *18* (19), e2107574.
51. Luo, X. F.; Ni, H. X.; Ma, H. L.; Qu, Z. Z.; Wang, J.; Zheng, Y. X.; Zuo, J. L., Fused π-extended multiple-resonance induced thermally activated delayed fluorescence materials for high-efficiency and narrowband OLEDs with low efficiency roll-off. *Adv. Opt. Mater.* **2022,** *10* (9), e2102513.
52. Bian, J.; Chen, S.; Qiu, L.; Tian, R.; Man, Y.; Wang, Y.; Chen, S.; Zhang, J.; Duan, C.; Han, C.; Xu, H., Ambipolar self-host functionalization accelerates blue multi-resonance thermally activated delayed fluorescence with internal quantum efficiency of 100. *Adv. Mater.* **2022,** *34* (17), e2110547.
53. Liu, J.; Chen, L.; Wang, X.; Yang, Q.; Zhao, L.; Tong, C.; Wang, S.; Shao, S.; Wang, L., Multiple resonance dendrimers containing boron, oxygen, nitrogen-doped polycyclic aromatic emitters for narrowband blue-emitting solution-processed OLEDs. *Macromol. Rapid. Commun.* **2022,** *43* (16), e2200079.
54. Qu, Y. K.; Zhou, D. Y.; Kong, F. C.; Zheng, Q.; Tang, X.; Zhu, Y. H.; Huang, C. C.; Feng, Z. Q.; Fan, J.; Adachi, C.; Liao, L. S.; Jiang, Z. Q., Steric modulation of spiro structure for highly efficient multiple resonance emitters. *Angew. Chem. Int. Ed.* **2022,** *61* (22), e202201886.
55. Cai, X.; Xue, J.; Li, C.; Liang, B.; Ying, A.; Tan, Y.; Gong, S.; Wang, Y., Achieving 37.1% green electroluminescent efficiency and 0.09 ev full width at half maximum based on a ternary boron-oxygen-nitrogen embedded polycyclic aromatic system. *Angew. Chem. Int. Ed.* **2022,** *61* (23), e202200337.
56. Park, J.; Kim, K. J.; Lim, J.; Kim, T.; Lee, J. Y., High efficiency of over 25% and long device lifetime of over 500 h at 1000 nit in blue fluorescent organic light-emitting diodes. *Adv. Mater.* **2022,** *34* (21), e2108581.
57. Yang, W.; Li, N.; Miao, J.; Zhan, L.; Gong, S.; Huang, Z.; Yang, C., Simple double hetero[5]helicenes realize highly efficient and narrowband circularly polarized organic light-emitting diodes. *CCS Chem.* **2022,** *4* (11), 3463-3471.
58. Zhang, Y.; Li, G.; Wang, L.; Huang, T.; Wei, J.; Meng, G.; Wang, X.; Zeng, X.; Zhang, D.; Duan, L., Fusion of multi-resonance fragment with conventional polycyclic aromatic hydrocarbon for nearly BT2020 green emission. *Angew. Chem. Int. Ed.* **2022,** *61* (24), e202202380.
59. Yang, Y. ; Li, N.; Miao, J. ; Cao, X.; Ying, A. ; Pan, K. ; Lv, X.; Ni, F.; Huang, Z. ; Gong, S. ; Yang, C., Chiral multi-resonance TADF emitters exhibiting narrowband circularly polarized electroluminescence with an EQE of 37.2%. *Angew. Chem. Int. Ed.* **2022,** *61* (30), e202202227.
60. Lv, X.; Miao, J.; Liu, M.; Peng, Q.; Zhong, C.; Hu, Y.; Cao, X.; Wu, H.; Yang, Y.; Zhou, C.; Ma, J.; Zou, Y.; Yang, C., Extending the π-skeleton of multi-resonance TADF materials towards high-efficiency narrowband deep-blue emission. *Angew. Chem. Int. Ed.* **2022,** *61* (29), e202201588.
61. Xu, Y.; W, Q.; Wei, J.; Peng, X.; Xue,J.; Wang,Z.; Su,S.; Wang,Y., Constructing organic electroluminescent material with very high color purity and efficiency based on polycyclization of multiple resonance parent core. *Angew. Chem. In. Ed.* **2022,** *61* (30), e202204652.
62. Hua, T.; Miao, J.; Xia, H.; Huang, Z.; Cao, X.; Li, N.; Yang, C., Sulfone-incorporated multi-resonance TADF emitter for high-performance narrowband blue OLEDs with EQE of 32%. *Adv. Funct. Mater.* **2022,** *32* (31), 2201032.
63. Zou, Y.; Hu, J.; Yu, M.; Miao, J.; Xie, Z.; Qiu, Y.; Cao, X.; Yang, C., High-performance narrowband pure-red OLEDs with external quantum efficiencies up to 36.1% and ultralow efficiency roll-off. *Adv. Mater.* **2022,** *34* (29), e2201442.

64. Park, I. S.; Min, H.; Yasuda, T., Ultrafast triplet-singlet exciton interconversion in narrowband blue organoboron emitters doped with heavy chalcogens. *Angew. Chem. Int. Ed. Engl* **2022,** *61* (31), e202205684.
65. Wang, Y.; Di, K.; Duan, Y.; Guo, R.; Lian, L.; Zhang, W.; Wang, L., The selective regulation of borylation site based on one-shot electrophilic C–H borylation reaction, achieving highly efficient narrowband organic light-emitting diodes. *Chem. Eng. J.* **2022,** *431*, 133221.
66. Luo, X. F.; Ni, H. X.; Lv, A. Q.; Yao, X. K.; Ma, H. L.; Zheng, Y. X., High-efficiency and narrowband OLEDs from blue to yellow with ternary boron/nitrogen-based polycyclic heteroaromatic emitters. *Adv. Opt. Mater.* **2022,** *10* (16), e2200504.
67. Meng, G.; Dai, H.; Huang, T.; Wei, J.; Zhou, J.; Li, X.; Wang, X.; Hong, X.; Yin, C.; Zeng, X.; Zhang, Y.; Yang, D.; Ma, D.; Li, G.; Zhang, D.; Duan, L., Amine-directed formation of B-N bonds for BN-fused polycyclic aromatic multiple resonance emitters with narrowband emission. *Angew. Chem. Int. Ed.* **2022,** *61* (40), e202207293.
68. Wang, X.; Zhang, Y.; Dai, H.; Li, G.; Liu, M.; Meng, G.; Zeng, X.; Huang, T.; Wang, L.; Peng, Q.; Yang, D.; Ma, D.; Zhang, D.; Duan, L., Mesityl-functionalized multi-resonance organoboron delayed fluorescent frameworks with wide-range color tunability for narrowband OLEDs. *Angew. Chem. Int. Ed.* **2022,** *61* (38), e202206916.
69. Oda, S.; Sugitani, T.; Tanaka, H.; Tabata, K.; Kawasumi, R.; Hatakeyama, T., Development of pure green thermally activated delayed fluorescence material by cyano substitution. *Adv. Mater.* **2022,** *34* (32), e2201778.
70. Naveen, K. R.; Lee, H.; Seung, L. H.; Jung, Y. H.; Keshavananda Prabhu, C. P.; Muruganantham, S.; Kwon, J. H., Modular design for constructing narrowband deep-blue multiresonant thermally activated delayed fluorescent emitters for efficient organic light emitting diodes. *Chem. Eng. J.* **2023,** *451*, 138498.
71. Liu, J.; Zhu, Y.; Tsuboi, T.; Deng, C.; Lou, W.; Wang, D.; Liu, T.; Zhang, Q., Toward a BT.2020 green emitter through a combined multiple resonance effect and multi-lock strategy. *Nat. Commun.* **2022,** *13* (1), 4876.
72. Huang, F.; Fan, X.-C.; Cheng, Y.-C.; Wu, H.; Shi, Y.-Z.; Yu, J.; Wang, K.; Lee, C.-S.; Zhang, X.-H., Distinguishing the respective determining factors for spectral broadening and concentration quenching in multiple resonance type TADF emitter systems. *Mater. Horiz.* **2022,** *9* (8), 2226-2232.
73. Naveen, K. R.; Lee, H.; Braveenth, R.; Karthik, D.; Yang, K. J.; Hwang, S. J.; Kwon, J. H., Achieving high efficiency and pure blue color in hyperfluorescence organic light emitting diodes using organo-boron based emitters. *Adv. Funct. Mater.* **2021,** *32* (12), e2110356.
74. Cai, S.; Tong, G. S. M.; Du, L.; So, G. K.; Hung, F. F.; Lam, T. L.; Cheng, G.; Xiao, H.; Chang, X.; Xu, Z. X.; Che, C. M., Gold(I) multi-resonance thermally activated delayed fluorescent emitters for highly efficient ultrapure-green organic light-emitting diodes. *Angew. Chem. Int. Ed.* **2022,** *61* (52), e202213392.
75. Eungdo Kim, J. P., Mieun Jun, Hyosup Shin, Jangyeol Baek, Taeil Kim, Seran Kim, Jiyoung Lee, Heechoon Ahn, Jinwon Sun, Soo-Byung Ko, Seok-Hwan Hwang, Jun Yeob Lee, Changwoong Chu, Sunghan Kim, Highly efficient and stable deep-blue organic light-emitting diode using phosphor-sensitized thermally activated delayed fluorescence. *Sci. Adv.* **2022,** *8*, eabq1641.
76. Liu, Y.; Xiao, X.; Huang, Z.; Yang, D.; Ma, D.; Liu, J.; Lei, B.; Bin, Z.; You, J., Space-confined donor-acceptor strategy enables fast spin-flip of multiple resonance emitters for suppressing efficiency roll-off. *Angew. Chem. Int. Ed.* **2022,** *61* (40), e202210210.
77. Luo, X. F.; Song, S. Q.; Ni, H. X.; Ma, H.; Yang, D.; Ma, D.; Zheng, Y. X.; Zuo, J. L., Multiple-resonance-induced thermally activated delayed fluorescence materials based on indolo[3,2,1-jk]carbazole with an efficient narrowband pure-green electroluminescence. *Angew. Chem. Int. Ed.* **2022,** *61* (41), e202209984.
78. Wang, T.; Zou, Y.; Huang, Z.; Li, N.; Miao, J.; Yang, C., Narrowband emissive TADF conjugated polymers towards highly efficient solution-processible OLEDs. *Angew. Chem. Int. Ed.* **2022,** *61* (46), e202211172.
79. Chang, Y.; Wu, Y.; Wang, X.; Li, W.; Yang, Q.; Wang, S.; Shao, S.; Wang, L., Boron, sulfur-doped polycyclic aromatic hydrocarbon emitters with multiple-resonance-dominated lowest excited states for efficient narrowband deep-blue emission. *Chem. Eng. J.* **2023,** *451*, 138545.
80. Wang, Q.; Xu, Y.; Yang, T.; Xue, J.; Wang, Y., Precise functionalization of multiple resonance framework: constructing narrowband organic electroluminescent materials with external quantum efficiency over 40. *Adv. Mater.* **2022,** *35* (3), e2205166.
81. Lee, Y.; Hong, J.-I., Multiple resonance thermally activated delayed fluorescence enhanced by halogen atoms. *J. Mater. Chem. C* **2022,** *10* (33), 11855-11861.
82. Cheng, Y. C.; Fan, X. C.; Huang, F.; Xiong, X.; Yu, J.; Wang, K.; Lee, C. S.; Zhang, X. H., A highly twisted carbazole-fused DABNA derivative as an orange-red TADF emitter for OLEDs with nearly 40 % EQE. *Angew. Chem. Int. Ed.* **2022,** *61* (47), e202212575.
83. Wang, Y.; Zhang, K.; Chen, F.; Wang, X.; Yang, Q.; Wang, S.; Shao, S.; Wang, L., Boron-, sulfur- and nitrogen-doped tridecacyclic aromatic emitters with multiple resonance effect for narrowband red emission. *Chin. J. Chem.* **2022,** *40* (22), 2671-2677.
84. Lee, H.; Braveenth, R.; Park, J. D.; Jeon, C. Y.; Lee, H. S.; Kwon, J. H., Manipulating spectral width

and emission wavelength towards highly efficient blue asymmetric carbazole fused multi-resonance emitters. *ACS Appl. Mater. Interfaces* **2022,** *14* (32), 36927-36935.
85. Fan, T.; Zhang, Y.; Wang, L.; Wang, Q.; Yin, C.; Du, M.; Jia, X.; Li, G.; Zhang, D.; Duan, L., One-shot synthesis of B/N-doped calix[4]arene exhibiting narrowband multiple resonance fluorescence. *Angew. Chem. Int. Ed.* **2022,** *61* (52), e202213585.
86. Li, Q.; Wu, Y.; Yang, Q.; Wang, S.; Shao, S.; Wang, L., Selenium-doped polycyclic aromatic hydrocarbon multiresonance emitters with fast reverse intersystem crossing for narrowband blue emission. *ACS Appl. Mater. Interfaces* **2022,** *14* (44), 49995-50003 .
87. Cheon, H. J.; Woo, S. J.; Baek, S. H.; Lee, J. H.; Kim, Y. H., Dense local triplet states and steric shielding of a multi-resonance TADF emitter enable high-performance deep-blue OLEDs. *Adv. Mater.* **2022,** *34* (50), e2207416.
88. Cao, X.; Pan, K.; Miao, J.; Lv, X.; Huang, Z.; Ni, F.; Yin, X.; Wei, Y.; Yang, C., Manipulating exciton dynamics toward simultaneous high-efficiency narrowband electroluminescence and photon upconversion by a selenium-incorporated multiresonance delayed fluorescence emitter. *J. Am. Chem. Soc.* **2022,** *144* (50), 22976-22984.
89. Liao, X. J.; Pu, D.; Yuan, L.; Tong, J.; Xing, S.; Tu, Z. L.; Zuo, J. L.; Zheng, W. H.; Zheng, Y. X., Planar chiral multiple resonance thermally activated delayed fluorescence materials for efficient circularly polarized electroluminescence. *Angew. Chem. Int. Ed.* **2022,** *62* (6), e202217045.
90. Zhang, Y.; Wei, J.; Wang, L.; Huang, T.; Meng, G.; Wang, X.; Zeng, X.; Du, M.; Fan, T.; Yin, C.; Zhang, D.; Duan, L., Multiple fusion strategy for high-performance yellow OLEDs with full width at half maximums down to 23 nm and external quantum efficiencies up to 37.4%. *Adv. Mater.* **2022,** *35* (7), e2209396.
91. Cai, X.; Xu, Y.; Pan, Y.; Li, L.; Pu, Y.; Zhuang, X.; Li, C.; Wang, Y., Solution-processable pure-red multiple resonance-induced thermally activated delayed fluorescence emitter for organic light-emitting diode with external quantum efficiency over 20%. *Angew. Chem. Int. Ed.* **2023,** *62* (7), e202216473.
92. Wang, Y.; Guo, R.; Ying, A.; Di, K.; Chen, L.; Gu, H.; Liu, S.; Duan, Y.; Su, H.; Gong, S.; Wang, L., Multiple-resonance-type TADF emitter as sensitizer improving the performance of blue fluorescent organic light-emitting diodes. *Adv. Opt. Mater.* **2022,** *11* (1), e2202034.
93. Luo, X. F.; Ni, H. X.; Shen, L.; Wang, L.; Xiao, X.; Zheng, Y. X., An indolo[3,2,1-jk]carbazole-fused multiple resonance-induced thermally activated delayed fluorescence emitter for an efficient narrowband OLED. *Chem. Commun.* **2023,** *59* (17), 2489-2492.
94. Uemura, S.; Oda, S.; Hayakawa, M.; Kawasumi, R.; Ikeda, N.; Lee, Y. T.; Chan, C. Y.; Tsuchiya, Y.; Adachi, C.; Hatakeyama, T., Sequential multiple borylation toward an ultrapure green thermally activated delayed fluorescence material. *J. Am. Chem. Soc.* **2023,** *145* (3), 1505-1511.
95. Lee, Y. T.; Chan, C. Y.; Matsuno, N.; Uemura, S.; Oda, S.; Kondo, M.; Weerasinghe, R. W.; Hu, Y.; Lestanto, G. N. I.; Tsuchiya, Y.; Li, Y.; Hatakeyama, T.; Adachi, C., Bright, efficient, and stable pure-green hyperfluorescent organic light-emitting diodes by judicious molecular design. *Nat. Commun.* **2024,** *15* (1), 3174.
96. Luo, X. F.; Ni, H. X.; Liang, X.; Yang, D.; Ma, D.; Zheng, Y. X.; Zuo, J. L., Face-to-face steric modulation to achieve high performance multiple-resonance thermally activated delayed fluorescence emitters with quenching resistant effect. *Adv. Opt. Mater.* **2023,** *11* (8), e2203002.
97. Lei, B.; Huang, Z.; Li, S.; Liu, J.; Bin, Z.; You, J., Medium-ring strategy enables multiple resonance emitters with twisted geometry and fast spin-flip to suppress efficiency roll-off. *Angew. Chem. In. Ed.* **2023,** *62* (12), e202218405.
98. Chen, G.; Wang, J.; Chen, W. C.; Gong, Y.; Zhuang, N.; Liang, H.; Xing, L.; Liu, Y.; Ji, S.; Zhang, H. L.; Zhao, Z.; Huo, Y.; Tang, B. Z., Triphenylamine-functionalized multiple-resonance TADF emitters with accelerated reverse intersystem crossing and aggregation-induced emission enhancement for narrowband OLEDs. *Adv. Funct. Mater.* **2023,** **33** (12), e2211893.
99. Hu, Y. N.; Fan, X. C.; Huang, F.; Shi, Y. Z.; Wang, H.; Cheng, Y. C.; Chen, M. Y.; Wang, K.; Yu, J.; Zhang, X. H., Novel multiple resonance type TADF emitter as blue component for highly efficient blue-hazard-free white organic light-emitting diodes.*Adv. Opt. Mater.* **2022,** *11* (3), e2202267.
100. Hu, Y.; Miao, J.; Zhong, C.; Zeng, Y.; Gong, S.; Cao, X.; Zhou, X.; Gu, Y.; Yang, C., Peripherally heavy-atom-decorated strategy towards high-performance pure green electroluminescence with external quantum efficiency over 40%. *Angew. Chem. Int. Ed.* **2023,** *62* (19), e202302478.
101. Fan, T.; Du, M.; Jia, X.; Wang, L.; Yin, Z.; Shu, Y.; Zhang, Y.; Wei, J.; Zhang, D.; Duan, L., High-efficiency narrowband multi-resonance emitter fusing indolocarbazole donors for BT. 2020 red electroluminescence and ultra-long operation lifetime. *Adv. Mater.* **2023,** *35* (30), e2301018.
102. Meng, G.; Dai, H.; Zhou, J.; Huang, T.; Zeng, X.; Wang, Q.; Wang, X.; Zhang, Y.; Fan, T.; Yang, D.; Ma, D.; Zhang, D.; Duan, L., Wide-range color-tunable polycyclo-heteraborin multi-resonance emitters containing B-N covalent bonds. *Chem. Sci.* **2023,** *14* (4), 979-986.
103. Fan, X.-C.; Wang, K.; Shi, Y.-Z.; Cheng, Y.-C.; Lee, Y.-T.; Yu, J.; Chen, X.-K.; Adachi, C.; Zhang, X.-H., Ultrapure green organic light-emitting diodes based on highly distorted fused π-conjugated molecular design. *Nat. Photonics* **2023,** *17* (3), 280-285.

104. Stavrou, K.; Madayanad Suresh, S.; Hall, D.; Danos, A.; Kukhta, N. A.; Slawin, A. M. Z.; Warriner, S.; Beljonne, D.; Olivier, Y.; Monkman, A.; Zysman-Colman, E., Emission and absorption tuning in TADF B,N-doped heptacenes: toward ideal-blue hyperfluorescent OLEDs. *Adv. Opt. Mater.* **2022,** *10* (17), e2200688.
105. Madayanad Suresh, S.; Zhang, L.; Hall, D.; Si, C.; Ricci, G.; Matulaitis, T.; Slawin, A. M. Z.; Warriner, S.; Olivier, Y.; Samuel, I. D. W.; Zysman-Colman, E., A deep-blue-emitting heteroatom-doped MR-TADF nonacene for high-performance organic light-emitting diodes. *Angew. Chem. Int. Ed.* **2023,** *62* (8), e202215522.
106. Yang, M.; Konidena, R. K.; Shikita, S.; Yasuda, T., Facile dimerization strategy for producing narrowband green multi-resonance delayed fluorescence emitters. *J. Mater. Chem. C.* **2023,** *11* (3), 917-922.
107. Xue, W.; Yan, H.; He, Y.; Wu, L.; Zhang, X.; Wu, Y.; Xu, J.; He, J.; Yan, C.; Meng, H., Identifying the molecular origins of green BN-TADF material degradation and device stability via in situ Raman spectroscopy. *Chem. Eur. J.* **2022,** *28* (36), e202201006.
108. Du, C.-Z.; Lv, Y.; Dai, H.; Hong, X.; Zhou, J.; Li, J.-K.; Gao, R.-R.; Zhang, D.; Duan, L.; Wang, X.-Y., Indole-fused BN-heteroarenes as narrowband blue emitters for organic light-emitting diodes. *J. Mater. Chem. C* **2023,** *11* (7), 2469-2474.
109. Bae, J.; Sakai, M.; Tsuchiya, Y.; Ando, N.; Chen, X. K.; Nguyen, T. B.; Chan, C. Y.; Lee, Y. T.; Auffray, M.; Nakanotani, H.; Yamaguchi, S.; Adachi, C., Multiple resonance type thermally activated delayed fluorescence by dibenzo [1,4] azaborine derivatives. *Front. Chem.* **2022,** *10*, 990918.
110. Liu, G.; Sasabe, H.; Kumada, K.; Arai, H.; Kido, J., Nonbonding/bonding molecular orbital regulation of nitrogen-boron-oxygen-embedded blue/green multiresonant TADF emitters with high efficiency and color purity. *Chem. Eur. J.* **2022,** *28* (48), e202201605.
111. Naveen, K. R.; Oh, J. H.; Lee, H.; Kwon, J. H., Tailoring extremely narrow FWHM in hypsochromic and bathochromic shift of polycyclo-heteraborin MR-TADF materials for high-performance OLEDs. *Angew. Chem. In. Ed.* **2023,** *62* (32), e202306768.
112. Huang, Z.; Xie, H.; Miao, J.; Wei, Y.; Zou, Y.; Hua, T.; Cao, X.; Yang, C., Charge transfer excited state promoted multiple resonance delayed fluorescence emitter for high-performance narrowband electroluminescence. *J. Am. Chem. Soc.* **2023,** *145* (23), 12550-12560.
113. He, Y.-H.; Xie, F.-M.; Li, H.-Z.; Zhang, K.; Shen, Y.; Ding, F.; Wang, C.-Y.; Li, Y.-Q.; Tang, J.-X., Red-shift emission and rapid up-conversion of B,N-containing electroluminescent materials via tuning intramolecular charge transfer. *Mater. Chem. Front.* **2023,** *7* (12), 2454-2463.
114. Mubarok, H.; Amin, A.; Lee, T.; Jung, J.; Lee, J. H.; Lee, M. H., Triptycene-fused sterically shielded multi-resonance TADF emitter enables high-efficiency deep blue OLEDs with reduced dexter energy transfer. *Angew. Chem. Int. Ed.* **2023,** *62* (32), e202306879.
115. Cai, X.; Pu, Y.; Li, C.; Wang, Z.; Wang, Y., Multi-resonance building-block-based electroluminescent material: lengthening emission maximum and shortening delayed fluorescence lifetime. *Angew. Chem. Int. Ed.* **2023,** *62* (27), e202304104.
116. Wang, Q.; Yuan, L.; Qu, C.; Huang, T.; Song, X.; Xu, Y.; Zheng, Y. X.; Wang, Y., Constructing highly efficient circularly polarized multiple resonance thermally activated delayed fluorescence materials with intrinsically helical chirality. *Adv. Mater.* **2023,** *35* (42), e2305125.
117. Jin, J.; Duan, C.; Jiang, H.; Tao, P.; Xu, H.; Wong, W. Y., Integrating asymmetric O-B-N unit in multi-resonance thermally activated delayed fluorescence emitters towards high-performance deep-blue organic light-emitting diodes. *Angew. Chem. In. Ed.* **2023,** *62* (18), e202218947.
118. Dai, H.; Zhou, J.; Meng, G.; Wang, L.; Duan, L.; Zhang, D., Highly efficient and stable blue OLEDs based on B-N bonds embedded 6,12-Diphenyl-5,11-dihydroindolo[3,2-b]carbazole with narrowband emission and extended lifetime. *Chin. J. Chem.* **2023,** *41* (6), 657-664.
119. Fan, X. C.; Huang, F.; Wu, H.; Wang, H.; Cheng, Y. C.; Yu, J.; Wang, K.; Zhang, X. H., A quadruple-borylated multiple-resonance emitter with para/meta heteroatomic patterns for narrowband orange-red emission. *Angew. Chem. Int. Ed.* **2023,** *62* (35), e202305580.
120. Meng, G.; Zhou, J.; Han, X. S.; Zhao, W.; Zhang, Y.; Li, M.; Chen, C. F.; Zhang, D.; Duan, L., B-N covalent bond embedded double hetero-[n]helicenes for pure red narrowband circularly polarized electroluminescence with high efficiency and stability. *Adv. Mater.* **2023,** *36* (5), e2307420.
121. Liu, J.-P.; Chen, L.; Zhao, L.; Tong, C.-Y.; Wang, S.-M.; Shao, S.-Y.; Wang, L.-X., Carbazole-based multiple resonance dendrimers with narrowband blue emission for solution-processed OLEDs. *Chin. J. Polym. Sci.* **2023,** *41* (5), 802-810.
122. Yang, W.; Miao, J.; Hu, F.; Zou, Y.; Zhong, C.; Gong, S.; Yang, C., An effective approach toward yellow-to-orange multi-resonance TADF emitters by integrating strong electron donor into B/N-based polycyclic architecture: high performance OLEDs with nearly 40% EQE. *Adv. Funct. Mater.* **2023,** *33* (23), e2213056.
123. Zhang, F.; Rauch, F.; Swain, A.; Marder, T. B.; Ravat, P., Efficient narrowband circularly polarized light emitters based on 1,4-B,N-embedded rigid donor-acceptor helicenes. *Angew. Chem. In. Ed.* **2023,** *62* (16), e202218965.
124. Hu, Y.; Huang, M.; Liu, H.; Miao, J.; Yang, C., Narrowband fluorescent emitters based on BN-doped

polycyclic aromatic hydrocarbons for efficient and stable organic light-emitting diodes. *Angew. Chem. Int. Ed.* **2023,** *62* (46), e202312666.
125. Zhang, K.; Wang, X.; Chang, Y.; Wu, Y.; Wang, S.; Wang, L., Carbazole-decorated organoboron emitters with low-lying HOMO levels for solution-processed narrowband blue hyperfluorescence OLED devices. *Angew. Chem. Int. Ed.* **2023,** *135* (47), e202313084.
126. Wang, Q.; Xu, Y.; Huang, T.; Qu, Y.; Xue, J.; Liang, B.; Wang, Y., Precise regulation of emission maxima and construction of highly efficient electroluminescent materials with high color purity. *Angew. Chem. In. Ed.* **2023,** *62* (19), e202301930.
127. Chen, H.; Fan, T.; Zhao, G.; Zhang, D.; Li, G.; Jiang, W.; Duan, L.; Zhang, Y., A simple molecular design strategy for pure - red multiple resonance emitters. *Angew. Chem. In. Ed.* **2023,** *62* (20), e202300934.
128. Meng, G.; Zhou, J.; Huang, T.; Dai, H.; Li, X.; Jia, X.; Wang, L.; Zhang, D.; Duan, L., B−N/B−O contained heterocycles as fusion locker in multi - resonance frameworks towards highly - efficient and stable ultra - narrowband emission. *Angew. Chem. In. Ed.* **2023,** *62* (45), e202309923.
129. Xiao, X.; Lei, B.; Wu, D.; Bin, Z., "Medium-ring" strategy enables high-performance narrowband pure-blue multi-resonance emitters: boost provided by a unique perpendicular geometry. *Chem. Commun.* **2023,** *59* (43), 6556-6559.
130. Hua, T.; Li, N.; Huang, Z.; Zhang, Y.; Wang, L.; Chen, Z.; Miao, J.; Cao, X.; Wang, X.; Yang, C., Narrowband near-infrared multiple-resonance thermally activated delayed fluorescence emitters towards high-performance and stable organic light-emitting diodes. *Angew. Chem. Int. Ed.* **2023,** *63* (7), e202318433.
131. Chang, Y.; Wu, Y.; Zhang, K.; Wang, S.; Wang, X.; Shao, S.; Wang, L., 1,8-diphenyl-carbazole-based boron, sulfur-containing multi-resonance emitters with suppressed aggregation emission for narrowband OLEDs. *Dyes Pigm.* **2023,** *220*, 111678.
132. Ye, Z.; Wu, H.; Xu, Y.; Hua, T.; Chen, G.; Chen, Z.; Yin, X.; Huang, M.; Xu, K.; Song, X.; Huang, Z.; Lv, X.; Miao, J.; Cao, X.; Yang, C., Deep-blue narrowband hetero[6]helicenes showing circularly polarized thermally activated delayed fluorescence toward high-performance OLEDs. *Adv. Mater.* **2023,** *36* (1) , e2308314.
133. Wang, H.; Fan, X. C.; Chen, J. X.; Cheng, Y. C.; Zhang, X.; Wu, H.; Xiong, X.; Yu, J.; Wang, K.; Zhang, X. H., A multiple resonance emitter integrating para-B-π-B′/meta-N-π-N pattern via an unembedded organoboron decoration for both high-efficiency solution-and vacuum-processed OLEDs. *Adv. Funct. Mater.* **2023,** *33* (47), e2306394.
134. Yang, Z.; Yang, G. X.; Jiang, S.; Li, M.; Qiu, W.; Peng, X.; Shen, C.; Gan, Y.; Liu, K.; Li, D.; Su, S. J., Carbonyl fused organoboron polycyclic aromatic hydrocarbon for bathochromic - shifted narrowband OLED. *Adv. Opt. Mater.* **2023,** *12* (9), e2301711.
135. Cheng, Y.-C.; Tang, X.; Wang, K.; Xiong, X.; Fan, X.-C.; Luo, S.; Walia, R.; Xie, Y.; Zhang, T.; Zhang, D.; Yu, J.; Chen, X.-K.; Adachi, C.; Zhang, X.-H., Efficient, narrow-band, and stable electroluminescence from organoboron-nitrogen-carbonyl emitter. *Nat. Commun.* **2024,** *15* (1), 731.
136. Du, M.; Mai, M.; Zhang, D.; Duan, L.; Zhang, Y., Stereo effects for efficient synthesis of orange-red multiple resonance emitters centered on a pyridine ring. *Chem. Sci.* **2024,** *15*, 3148-3154.
137. Zou, Y.; Yu, M.; Xu, Y.; Xiao, Z.; Song, X.; Hu, Y.; Xu, Z.; Zhong, C.; He, J.; Cao, X.; Li, K.; Miao, J.; Yang, C., Acceleration of reverse intersystem crossing in multi-resonance TADF emitter. *Chem* **2024,** *10* (5), 1485-1501.
138. Guo, W.-C.; Zhao, W.-L.; Tan, K.-K.; Li, M.; Chen, C.-F., B,N-embedded hetero[9]helicene toward highly efficient circularly polarized electroluminescence. *Angew. Chem. In. Ed.* **2024,** *63* (18), e202401835.
139. Wu, L.; Huang, Z.; Miao, J.; Wang, S.; Li, X.; Li, N.; Cao, X.; Yang, C., Orienting group directed cascade borylation for efficient one - shot synthesis of 1,4 - BN-doped polycyclic aromatic hydrocarbons as narrowband organic emitters. *Angew. Chem. In. Ed.* **2024,** *63* (18), e202402020.
140. Wu, Z. G.; Xin, Y.; Lu, C.; Huang, W.; Xu, H.; Liang, X.; Cao, X.; Li, C.; Zhang, D.; Zhang, Y.; Duan, L., Precise regulation of multiple resonance distribution regions of a b,n - embedded polycyclic aromatic hydrocarbon to customize its BT2020 green emission. *Angew. Chem. In. Ed.* **2024,** *63* (7), e202318742.
141. Bai, K.; Li, M.; Tan, X.; Dai, L.; Liang, K.; Li, H.; Su, S.-J., Reducing intersystem crossing rates of boron emitters for high-efficiency and long-lifetime deep-blue OLEDs. *J. Mater. Chem. C* **2023,** *11* (46), 16159-16167.
142. Wang, Q.; Huang, T.; Qu, Y.; Song, X.; Xu, Y.; Wang, Y., Frontier molecular orbital engineering of aromatic donor fusion: modularly constructing highly efficient narrowband yellow electroluminescence. *ACS Appl. Mater. Interfaces* **2024,** *16* (4), 4948-4957.
143. Luo, W.; Wang, T.; Huang, Z.; Huang, H.; Li, N.; Yang, C., Blue TADF conjugated polymers with multi-resonance feature toward solution-processable narrowband blue OLEDs. *Adv. Funct. Mater.* **2023,** *34* (6), e2310042.
144. Wang, T.; Yin, X.; Cao, X.; Yang, C., A simple approach to solution-processible small-molecule multi-resonance TADF emitters for high-performance narrowband OLEDs. *Angew. Chem. Int. Ed.* **2023,** *62* (24), e202301988.
145. Lee, T.; Jang, J. H.; Nguyen, N. N. T.; Jung, J.; Lee, J. H.; Lee, M. H., Ortho-carborane decorated multi-resonance TADF emitters: preserving local excited state and high efficiency in OLEDs. *Adv. Sci.* **2024,** *11*

(11), e2309016.
146. Jin, J. M.; Liu, D.; Chen, W. C.; Shi, C.; Chen, G.; Wang, X.; Xing, L.; Ying, W.; Ji, S.; Huo, Y.; Su, S. J., Synergetic modulation of steric hindrance and excited state for anti-quenching and fast spin-flip multi-resonance thermally activated delayed fluorophore. *Angew. Chem. Int. Ed.* **2024,** *63* (16). e202401120.
147. Jing, Y.-Y.; Wu, H.; Li, N.; Luo, S.; Ye, Z.; Wang, X.; Cao, X.; Yang, C., Steric groups fusion strategy for green multi-resonance emitters toward efficient OLEDs with narrowband emission. *Dyes Pigm.* **2023,** *219*, 111520.
148. He, J.; Xu, Y.; Luo, S.; Miao, J.; Cao, X.; Zou, Y., Phenoxazine and phenothiazine embedded multi-resonance emitters for highly efficient pure-red OLEDs with improved color purity. *Chem. Eng. J.* **2023,** *471*, 144565.
149. Wang, S.; Xu, Y.; Miao, J.; Hua, T.; Cao, X.; Li, N.; Huang, Z.; Yang, C., Peripheral decoration of multi-resonance TADF emitter for narrowband blue OLEDs. *Chem. Eng. J.* **2023,** *471*, 144664.
150. Gan, X. Q.; Ding, Z. M.; Liu, D. H.; Zheng, W. Q.; Ma, B.; Zhang, H.; Chang, X.; Wang, L.; Liu, Y.; Wu, X.; Su, S. J.; Zhu, W., High-efficiency and narrow‐band near‐ultraviolet emitters with low CIEy of 0.03 by incorporating extra weak charge transfer channel into multi‐resonance skeleton. *Adv. Opt. Mater.* **2023,** *11* (15), 2300195.
151. Ni, H.-X.; Sun, W.; Luo, X.-F.; Yuan, L.; Liang, X.; Liao, X.-J.; Zhou, L.; Zheng, Y.-X., Peripherally non-planar multiple resonance induced thermally activated delayed fluorescence materials containing silyl units. *The Innovation Mater.* **2023,** *1* (3), 100041.
152. Song, X.; Shen, S.; Zou, S.; Wang, Y.; Guo, F.; Gao, S.; Zhang, Y., Secondary donor-acceptor group enable efficient pure green organic light-emitting devices based on multi-resonance TADF emitters. *Chem. Eng. J.* **2024,** *481*, 148794.
153. Lee, Y. T.; Chan, C. Y.; Tanaka, M.; Mamada, M.; Goushi, K.; Tang, X.; Tsuchiya, Y.; Nakanotani, H.; Adachi, C., Tailor-made multi-resonance terminal emitters toward narrowband, high-efficiency, and stable hyperfluorescence organic light-emitting diodes. *Adv. Opt. Mater.* **2022,** *10* (17), e2200682.
154. Konidena, R. K.; Yang, M.; Yasuda, T., A pi-extended tercarbazole-core multi-resonance delayed fluorescence emitter exhibiting efficient narrowband yellow electroluminescence. *Chem. Commun.* **2023,** *59* (68), 10251-10254.
155. Zou, Y.; He, J.; Li, N.; Hu, Y.; Luo, S.; Cao, X.; Yang, C., Precisely regulating the double-boron-based multi-resonance framework towards pure-red emitters: high-performance OLEDs with CIE coordinates fully satisfying the BT.2020 standard. *Mater. Horiz.* **2023,** *10* (9), 3712-3718.
156. Xu, Y.; Han, J.; Li, N.; Huang, Z.; Miao, J.; Yang, C., B/N/O-participated multi-resonance TADF emitters by a simple peripheral decoration strategy enable high-efficiency electroluminescence with EQEs up to 36.5%. *J. Mater. Chem. C* **2023,** *11* (40), 13733-13739.
157. Li, B.; Lou, J.; Zhang, B.; Liu, L.; He, X.; Xu, H.; Feng, X.; Zhang, H.; Wang, Z.; Tang, B. Z., Modulating electronic confinement and structural distortion of multiple resonance emitters enables high-performance ultrapure blue OLED. *Chem. Eng. J.* **2024,** *482*, 148876.
158. Jing, Y. Y.; Yang, Y.; Li, N.; Ye, Z.; Wang, X.; Cao, X.; Yang, C., Indolo[3,2-b]indole-based multi-resonance emitters for efficient narrowband pure-green organic light-emitting diodes. *Luminescence* **2024,** *39* (1), e4624.
159. Kumada, K.; Sasabe, H.; Matsuya, M.; Yoshida, N.; Hoshi, K.; Nakamura, T.; Nemma, H.; Kido, J., Phenylene-bridged cyclic multi-resonance TADF emitters for high-efficiency and high-color-purity sky-blue OLEDs with EQE of 30%. *J. Mater. Chem. C* **2023,** *11* (40), 13782-13787.
160. Hu, J. J.; Wei, Y.; Wang, X. Z.; Liang, X.; Liao, X. J.; Yuan, L.; Ni, H. X.; Zheng, Y. X., Efficient ultra-narrowband OLEDs based on carbazole-fused dual-boron embedded multi-resonance thermally activated delayed fluorescence materials. *Adv. Opt. Mater.* **2024,** *12* (15), e2302987.
161. Sano, Y.; Shintani, T.; Hayakawa, M.; Oda, S.; Kondo, M.; Matsushita, T.; Hatakeyama, T., One-shot construction of BN-embedded heptadecacene framework exhibiting ultra-narrowband green thermally activated delayed fluorescence. *J. Am. Chem. Soc.* **2023,** *145* (21), 11504-11511.
162. Huang, F.; Fan, X. C.; Cheng, Y. C.; Wu, H.; Xiong, X.; Yu, J.; Wang, K.; Zhang, X. H., Combining carbazole building blocks and nu-DABNA heteroatom alignment for a double boron-embedded MR-TADF emitter with improved performance. *Angew. Chem. Int. Ed.* **2023,** *62* (32), e202306413.
163. Li, Z.; L, Z.; Zhang, S.; Liu, M.; Gao, G.; You, J.; Bin, Z., Narrowband pure-green emitters based on naphthalene-fused metapositioned double boron framework. *Sci. China Mater.* **2024,** *67,* 1581-1587.
164. Wu, H.; Shi, Y.-Z.; Li, M.-Y.; Fan, X.-C.; Huang, F.; Wang, K.; Yu, J.; Zhang, X.-H., Conformational isomerization imparts low concentration dependence to multiple resonance thermally activated delayed fluorescence (MR-TADF) emitters. *Chem. Eng. J.* **2024,** *480*, 147977.
165. Wang, C.; Hu, N.; Chen, Z.; Chen, Y.; Chang, P.; Han, C.; Cao, X.; Xu, H., Efficient narrowband organic light-emitting diodes based on B,O embedded multi-resonance emitters containing B-N covalent bonds. *Chem. Eng. J.* **2024,** *488*, 150785.
166. Wu, Q.; Li, J.; Liu, D.; Mei, Y.; Liu, B.; Wang, J.; Xu, M.; Li, Y., Dual emission from donor-modified MR-TADF emitter: Evidence for coexistence of TICT and MR excited states. *Dyes Pigm.* **2023,** *217*, 111421.
167. Yi-Kuan Chen, J. L., Tien-Lin Wu, Elevating upconversion performance of a multiple resonance

thermally activated delayed fluorescence emitter via an embedded azepine approach. *Chem. Sci.* **2024,** *15* (26)*,* 10146-10154.
168. Di, K.; Guo, R.; Wang, Y.; Lv, Y.; Su, H.; Zhang, Q.; Yang, B.; Wang, L., Achieving high-performance narrowband blue MR-TADF emitters by suppressing isomer formation and extending π-conjugate skeletons. *J. Mater. Chem. C* **2023,** *11* (19), 6429-6437.
169. Keruckiene, R.; Vaitusionak, A. A.; Hulnik, M. I.; Berezianko, I. A.; Gudeika, D.; Macionis, S.; Mahmoudi, M.; Volyniuk, D.; Valverde, D.; Olivier, Y.; Woon, K. L.; Kostjuk, S. V.; Reineke, S.; Grazulevicius, J. V.; Sini, G., Is a small singlet–triplet energy gap a guarantee of TADF performance in MR-TADF compounds? Impact of the triplet manifold energy splitting. *J. Mater. Chem. C* **2024,** *12* (10), 3450-3464.
170. Qi, Y.; Zhang, Z.; Sun, W.; Wu, S.; Liu, J.; Lin, Z.; Jiang, P.; Yu, H.; Zhou, L.; Lu, G., High-efficiency narrowband multi-resonance TADF emitters via the introduction of bulky adamantane units. *J. Mater. Chem. C* **2024,** *12* (17)*,* 6319-6325.
171. Wang, Z.; Qu, C.; Liang, J.; Zhuang, X.; Liu, Y.; Wang, Y., Optimizing high-efficiency multiple resonance blue delayed fluorescent emitters through charge transfer excited state tuning. *J. Mater. Chem. C* **2024,** *12* (16), 5985-5990.
172. Jing, Y.; L. N.; Cao, X.; Wu, H.; Miao, J.; Chen, Z.; Huang, M.; Wang, X.; Hu, Y.; Zou, Y.; Yang, C., Precise modulation of multiple resonance emitters toward efficient electroluminescence with pure-red gamut for high-definition displays. *Sci. Adv.* **2023,** *9* (30), eadh8296.
173. Li, G.; Du, M.; Fan, T.; Luo, X.; Duan, L.; Zhang, Y. Asymmetric structural design of a highly oriented multi-resonance emitter enables a record 41.5% external quantum efficiency in deep-blue OLED. *Mater. Today* **2024,** *73*, 30-37.
174. Mamada, M.; Aoyama, A.; Uchida, R.; Ochi, J.; Oda, S.; Kondo, Y.; Kondo, M.; Hatakeyama, T., Efficient deep-blue multiple-resonance emitters based on azepine-decorated Nu-DABNA for CIE (y) below 0.06. *Adv. Mater.* **2024,** *36* (30) e2402905.
175. An, R. Z.; Sun, Y.; Chen, H. Y.; Liu, Y.; Privitera, A.; Myers, W. K.; Ronson, T. K.; Gillett, A. J.; Greenham, N. C.; Cui, L. S., Excited-state engineering enables efficient deep-blue light-emitting diodes exhibiting BT.2020 color gamut. *Adv. Mater.* **2024,** *36* (31), e2313602.
176. Huang, X.; Liu, J.; Xu, Y.; Chen, G.; Huang, M.; Yu, M.; Lv, X.; Yin, X.; Zou, Y.; Miao, J.; Cao, X.; Yang, C., B–N covalent bond-involved pi-extension of multiple resonance emitters enables high-performance narrowband electroluminescence. *Nat. Sci. Rev.* **2024,** *11* (6), nwae115.
177. Chen, Z.; Liu, D.; Li, M.; Jiao, Y.; Yang, Z.; Liu, K.; Su, S. J., Advancing triplet exciton harvesting through heavy atom selenium manipulation in multiple resonance thermally activated delayed fluorescent emitters. *Adv. Funct. Mater.* **2024,** *34* (41), e2404278.
178. Huang, H.; Li, N.; Li, W.; Mo, X.; Cao, X.; Miao, J.; Yin, X.; Yang, C., Synergistic modulation of excited state ingredients and chiroptical activity for high-performance pure-green circularly polarized electroluminescence. *Adv. Funct. Mater.* **2024,** *34* (39), e202403191.
179. Oda, S.; Kawakami, B.; Horiuchi, M.; Yamasaki, Y.; Kawasumi, R.; Hatakeyama, T., Ultra-narrowband blue multi-resonance thermally activated delayed fluorescence materials. *Adv. Sci.* **2022,** *10* (1), e2205070.
180. Palanisamy, P.; Kumar, O. P.; Kim, H. U.; Naveen, K. R.; Kim, J.-Y.; Baek, J.-H.; Chae, M. Y.; Kwon, J. H., Rigidification with indolocarbazole and molecular orbitals regulation by peripheral donation towards pure green polycyclo-heteraborin MR-TADF scaffolds for stable narrowband OLEDs. *Chem. Eng. J.* **2024,** *481*, 148781.
181. Yuan, L.; Xu, J. W.; Yan, Z. P.; Yang, Y. F.; Mao, D.; Hu, J. J.; Ni, H. X.; Li, C. H.; Zuo, J. L.; Zheng, Y. X., Tetraborated intrinsically axial chiral multi-resonance thermally activated delayed fluorescence materials. *Angew. Chem. Int. Ed.* **2024,** *63* (32), e202407277.
182. Feng, Y.; Xu, Y.; Qu, C.; Wang, Q.; Ye, K.; Liu, Y.; Wang, Y., Structurally tunable donor-bridge-fluorophore architecture enables highly efficient and concentration-independent narrowband electroluminescence. *Adv. Mater.* **2024,** *36* (31), e2403061.
183. Weerasinghe, R. W.; Madayanad Suresh, S.; Hall, D.; Matulaitis, T.; Slawin, A. M. Z.; Warriner, S.; Lee, Y. T.; Chan, C. Y.; Tsuchiya, Y.; Zysman-Colman, E.; Adachi, C., A boron, nitrogen, and oxygen doped pi-extended helical pure blue multiresonant thermally activated delayed fluorescent emitter for organic light emitting diodes that shows fast $k_{RISC}$ without the use of heavy atoms. *Adv. Mater.* **2024,** *36* (26), e2402289.
184. Cai, X.; Pan, Y.; Li, C.; Li, L.; Pu, Y.; Wu, Y.; Wang, Y., Nitrogen-embedding strategy for short-range charge transfer excited states and efficient narrowband deep-blue organic light emitting diodes. *Angew. Chem. Int. Ed.* **2024**, *63* (35), e202408522.
185. Ochi, J.; Yamasaki, Y.; Tanaka, K.; Kondo, Y.; Isayama, K.; Oda, S.; Kondo, M.; Hatakeyama, T., Highly efficient multi-resonance thermally activated delayed fluorescence material toward a BT.2020 deep-blue emitter. *Nat. Commun.* **2024,** *15* (1), 2361.
186. Cao, X.; X. H., Miao, J.; Sun, H.; Su, C.; Sun, L.; Liao, Y.; Chen, Z.; Zhong, C.; Lin, H.; Lv, X.; Li, N.; Huang, Z.; Chen, Z.; Hua, T.; Yin, X.; Zou, Y.; Yang, C., Topological structure optimization of B,N-doped nanographenes for deep-blue emitters. *Research Square* **2024**, DOI: 10.21203/rs.3.rs-4346848/v1.
187. Hua, T.; Cao, X.; Miao, J.; Yin, X.; Chen, Z.; Huang, Z.; Yang, C., Deep-blue organic light-emitting

diodes for ultrahigh-definition displays. *Nat. Photonics* **2024,** *18*, 1161-1169.
188. Wang, H.; Cheng, Y. C.; Fan, X. C.; Chen, D. Y.; Xiong, X.; Hao, X. Y.; Shi, Y. Z.; Yu, J.; Huang, D.; Chen, J. X.; Wang, K.; Zhang, X. H., Efficient solution-processable OLEDs near BT.2020 red standard enabled by a multiresonant emitter. *Sci. Bull.* **2024**, *69* (19), 2983-2986.
189. Chen, H.; Du, M.; Qu, C.; Jin, Q.; Tao, Z.; Ji, R.; Zhao, G.; Zhou, T.; Lou, Y.; Sun, Y.; Jiang, W.; Duan, L.; Zhang, Y., Clar's Aromatic pi-sextet rule for the construction of red multiple resonance emitter. *Angew. Chem. Int. Ed.* **2024**, *64* (3), e202415400.
190. Wang, Y.; Zhao, W. L.; Gao, Z.; Qu, C.; Li, X.; Jiang, Y.; Hu, L.; Wang, X. Q.; Li, M.; Wang, W.; Chen, C. F.; Yang, H. B., Switchable topologically chiral [2]catenane as multiple resonance thermally activated delayed fluorescence emitter for efficient circularly polarized electroluminescence. *Angew. Chem. Int. Ed.* **2024,** *64* (5), e202417458.
191. Zhang, F.; Brancaccio, V.; Saal, F.; Deori, U.; Radacki, K.; Braunschweig, H.; Rajamalli, P.; Ravat, P., Ultra-narrowband circularly polarized luminescence from multiple 1,4-azaborine-embedded helical nanographenes. *J. Am. Chem. Soc.* **2024,** *146* (43), 29782-29791.
192. Yuan, W.; Jin, Q.; Du, M.; Duan, L.; Zhang, Y., Tailoring ultra-narrowband tetraborylated multiple resonance emitter for high-performance blue OLED. *Adv. Mater.* **2024,** *36* (48), e2410096.
193. Xiong, X.; Chen, T. F.; Walia, R.; Fan, X. C.; Cheng, Y. C.; Wang, H.; Wu, H.; Chen, X. K.; Yu, J.; Wang, K.; Zhang, X. H., Stepwise one-shot borylation reactions for intersecting DABNA substructures exhibiting bright yellow-green electroluminescence with EQE beyond 40 % and mild roll-off. *Angew. Chem. Int. Ed.* **2024**, *64* (2), e202414882.
194. Yuan, H.-T.; Yang, Y.-J.; Yu, Z.-H.; Zheng, Q.; Yan, H.-Y.; Wang, Y.; Zhou, D.-Y.; Liao, L.-S.; Jiang, Z.-Q., Silicon-based peripheral steric donor modifications for a high-efficiency multi-resonance thermally activated delayed fluorescence emitter. *J. Mater. Chem. C* **2024,** *12* (46), 18725-18731.
195. Zheng, Q.; Qu, Y.-K.; Zuo, P.; Yuan, H.-T.; Yang, Y.-J.; Qiu, Y.-C.; Liao, L.-S.; Zhou, D.-Y.; Jiang, Z.-Q., Enhancing multi-resonance thermally activated delayed fluorescence emission via through-space heavy-atom effect. *Chem* **2024**, e102353.
196. Pu, Y.; Cai, X.; Qu, Y.; Cui, W.; Li, L.; Li, C.; Zhang, Y.; Wang, Y., Spiro-carbon-locking and sulfur-embedding strategy for constructing deep-red organic electroluminescent emitter with high efficiency. *Angew. Chem. Int. Ed.* **2024,** *64* (8), e202420253.
197. Pu, Y.; Jin, Q.; Zhang, Y.; Li, C.; Duan, L.; Wang, Y. Sulfur-locked multiple resonance emitters for high performance orange-red/deep-red OLEDs. *Nat. Commun.* **2025,** *16* (1), e332.
198. Chen, D.; Wang, H.; Sun, D.; Wu, S.; Wang, K.; Zhang, X. H.; Zysman-Colman, E., The combination of a donor-acceptor TADF and a MR-TADF emitting core results in outstanding electroluminescence performance. *Adv. Mater.* **2024,** *36* (50), e2412761.
199. Chang, Y.; Zhang, K.; Zhao, L.; Wang, X.; Wang, S.; Shao, S.; Wang, L., Endo-encapsulated multi-resonance dendrimers with through-space interactions for efficient narrowband blue-emitting solution-processed OLEDs. *Angew. Chem. Int. Ed.* **2024,** *64* (3), e202415607.
200. Ge, L.; Zhang, W.; Hao, Y. H.; Li, M.; Liu, Y.; Zhou, M.; Cui, L. S., Efficient and stable narrowband pure-red light-emitting diodes with electroluminescence efficiencies exceeding 43. *J. Am. Chem. Soc.* **2024**, *146* (47), 32826-32836.
201. Wang, X. Z.; Xing, S.; Xiao, X.; Yuan, L.; Hou, Z. Y.; Zheng, Y. X., Axial chiral biphenyl mr-tadf enantiomers for efficient narrowband circularly polarized electroluminescence. *Adv. Funct. Mater.* **2024**, *35* (1), e202412044.
202. Huang, T.; Yuan, L.; Lu, X.; Qu, Y.; Qu, C.; Xu, Y.; Zheng, Y. X.; Wang, Y., Efficient circularly polarized multiple resonance thermally activated delayed fluorescence from B,N-embedded hetero[8]helicene enantiomers. *Chem. Sci.* **2024,** *15* (37), 15170-7.
203. Liao, X.-J.; Xing, S.; Hu, J.-J.; Wang, X.-Z.; Zheng, Y.-X. Phosphorus central chiral multiresonance thermally activated delayed fluorescence emitter towards narrowband and efficient circularly polarized electroluminescence. *CCS Chem.*e202404691.
204. Liu, J.; Yin, X.; Huang, M.; Miao, J.; Li, N.; Huang, Z.; Yang, C., High-performance narrowband pure-green oleds with gamut approaching bt.2020 standard: deuteration promotes device efficiency and lifetime simultaneously. *Adv. Mater.* **2024,** *37* (3), e202411610.
205. Xiao, S.; Cao, X.; Chen, G.; Yin, X.; Chen, Z.; Miao, J.; Yang, C., Synergistic pi-extension and peripheral-locking of b/n-based multi-resonance framework enables high-performance pure-green organic light-emitting diodes. *Angew. Chem. Int. Ed.* **2024,** *64* (6), e202418348.
206. Feng, T.; Nie, X.; Liu, D.; Wu, L.; Liu, C. Y.; Mu, X.; Xin, Z.; Liu, B.; Qi, H.; Zhang, J.; Li, W.; Su, S. J.; Ge, Z., Multiple resonance quasi-fluorescence from bn-doped aromatic compounds modified with "naphthalene" units approaches the bt.2020 green light standard. *Angew. Chem. Int. Ed.* **2024,** *64* (3), e202415113.
207. Liu, F.; Cheng, Z.; Dong, W.; Yan, Y.; Xu, Y.; Su, Z.; Hu, Y.; Wan, L.; Lu, P., Precise regulation of the reverse intersystem crossing pathway by hybridized long-short axis strategy for high-performance multi-resonance TADF emitters. *Angew. Chem. Int. Ed.* **2024,** *64* (14), e202416154.
208. Wu, L.; Mu, X.; Liu, D.; Li, W.; Li, D.; Zhang, J.; Liu, C.; Feng, T.; Wu, Y.; Li, J.; Su, S. J.; Ge, Z.,

Regional functionalization molecular design strategy: a key to enhancing the efficiency of multi-resonance OLEDs. *Angew. Chem. Int. Ed.* **2024,** *63* (38), e202409580.
209. Cheng, H.; Guo, C.-H.; Li, M.; Guo, Y.; Fu, Z.; Liu, J.; Yang, Y.; Lan, J.; Bin, Z., Facile access to spirobifluorene-fused chiral multi-resonance materials with ultra-narrowband blue emission. *Sci. Bull.***2024,** *69* (11), 1674-1685.
210. Song, X. F.; Luo, S.; Li, N.; Wan, X.; Miao, J.; Zou, Y.; Li, K.; Yang, C., Gold coordination-accelerated multi-resonance TADF emission for efficient solution-processible ultrapure deep-blue OLEDs. *Angew. Chem. Int. Ed.* **2024,** *64* (1), e202413536.
211. Jiang, K.; Chang, X.; Zhu, J.; Zhu, T.; Yu, J.; Wang, Y.; Zhang, Y.; Ma, D.; Zhu, W., High-performance solution-processable organic light-emitting diode based on a narrowband near-ultraviolet emitter and a hot exciton strategy. *Angew. Chem. Int. Ed.* **2024,** *64* (6), e202421520.
212. Jiang, D.; Sasabe, H.; Chen, Y.; Sagae, Y.; Sato, H.; Yokoyama, D.; Katagiri, H.; Kido, J., A novel matrix-free hyperfluorescent system based on anti-quenching TADF host for high color purity MR-TADF emitters. *Adv. Mater.* **2024,** *37* (5), e2409746.
213. Su, H.; Wang, Y.; Di, K.; Yue, H.; Huang, S.; Tian, Y.; Zhang, Q.; Shao, H.; Guo, R.; Wang, L., High color purity deep-blue multi-resonance tadf material with narrowband emission toward bt.2020 standard. *Adv. Funct. Mater.* **2024**, e2419679.
214. Liu, M.; Li, C.; Liao, G.; Zhao, F.; Yao, C.; Wang, N.; Yin, X., Narrowband blue circularly polarized luminescence emitter based on bn-doped benzo[6]helicene with stimuli-responsive properties. *Chem. Eur. J.* **2024,** *30* (52), e202402257.
215. Hu, J. J.; Liang, X.; Yan, Z. P.; Liang, J. Q.; Ni, H. X.; Yuan, L.; Zuo, J. L.; Zheng, Y. X., An efficient ultra-narrowband yellow emitter based on a double-boron-embedded tetraazacyclophane. *Angew. Chem. Int. Ed.* **2025,** *64*(10), e202421102.
216. Liu, Z.; Meng, L.; Jiang, Y.; Li, C.; Gu, H.; Zhao, K.; Zhang, J.; Meng, H.; Ren, Y., Hyperconjugation engineering of π-Extended azaphosphinines for designing tunable thermally activated delayed fluorescence emitters. *J. Am. Chem. Soc.* **2025,** *147* (4), 3650-3661.
217. Zhang, K.; Wang, X.; Wang, M.; Wang, S.; Wang, L., Solution-processed blue narrowband oled devices with external quantum efficiency beyond 35% through horizontal dipole orientation induced by electrostatic interaction. *Angew. Chem. Int. Ed.* **2025,** *64* (13), e202423812.
218. Xue, Z.; Y, H.; Xiao, S.; Liu, J.; Miao, J.; Yang, C. Cyano-modified multi-resonance thermally activated delayed fluorescent emitters towards pure-green oleds with a cie y value of 0.74. *Angew. Chem. Int. Ed.* **2025**, e202500108.
219. Wu, L.; Xin, Z.; Liu, D.; Li, D.; Zhang, J.; Zhou, Y.; Wu, S.; Wang, T.; Su, S. J.; Li, W.; Ge, Z., Bifunctional group modulation strategy enables mr-tadf electroluminescence toward bt.2020 green light standard. *Adv. Mater.* **2025**, *37* (9), e2416224.
220. Zeng, X.; Luo, X.; Meng, G.; Wang, X.; Zhang, D.; Duan, L., Sym- and Asym-expanded heterohelicene isomers featuring extended multi-resonance skeleton for narrowband deep-blue fluorescence. *Angew. Chem. Int. Ed.* **2025**, *64* (13), e202423670.
221. An, R. Z.; Zhao, F. M.; Shang, C.; Zhou, M.; Cui, L., Excited-state and steric hindrances engineering enable fast spin-flip narrowband thermally activated delayed fluorescence emitters with enhanced quenching resistance. *Angew. Chem. Int. Ed.* **2025,** *137* (11), e202420489.
222. Wan, D.; Zhou, J.; Yang, Y.; Meng, G.; Zhang, D.; Duan, L.; Ding, J., Peripheral substitution engineering of MR-TADF emitters embedded with b–n covalent bond towards efficient BT.2020 blue electroluminescence. *Adv. Mater.* **2024,** *36* (49), e2409706.
223. Wang, J.; Li, N.; Zhong, C.; Miao, J.; Huang, Z.; Yu, M.; Hu, Y. X.; Luo, S.; Zou, Y.; Li, K.; Yang, C., Metal-perturbed multiresonance TADF emitter enables high-efficiency and ultralow efficiency roll-off nonsensitized OLEDs with pure green gamut. *Adv. Mater.* **2023,** *35* (6), e2208378.
224. Yuan, Y.; Tang, X.; Du, X. Y.; Hu, Y.; Yu, Y. J.; Jiang, Z. Q.; Liao, L. S.; Lee, S. T., The design of fused amine/carbonyl system for efficient thermally activated delayed fluorescence: novel multiple resonance core and electron acceptor. *Adv. Opt. Mater.* **2019,** *7* (7), e1801536.
225. Li, X.; Shi, Y. Z.; Wang, K.; Zhang, M.; Zheng, C. J.; Sun, D. M.; Dai, G. L.; Fan, X. C.; Wang, D. Q.; Liu, W.; Li, Y. Q.; Yu, J.; Ou, X. M.; Adachi, C.; Zhang, X. H., Thermally activated delayed fluorescence carbonyl derivatives for organic light-emitting diodes with extremely narrow full width at half-maximum. *ACS Appl. Mater. Interfaces* **2019,** *11* (14), 13472-13480.
226. Hall, D.; Suresh, S. M.; dos Santos, P. L.; Duda, E.; Bagnich, S.; Pershin, A.; Rajamalli, P.; Cordes, D. B.; Slawin, A. M. Z.; Beljonne, D.; Köhler, A.; Samuel, I. D. W.; Olivier, Y.; Zysman-Colman, E., Improving processability and efficiency of resonant TADF emitters: a design strategy. *Adv. Opt. Mater.* **2019,** *8* (2), e1901627.
227. Sun, D.; Suresh, S. M.; Hall, D.; Zhang, M.; Si, C.; Cordes, D. B.; Slawin, A. M. Z.; Olivier, Y.; Zhang, X.; Zysman-Colman, E., The design of an extended multiple resonance TADF emitter based on a polycyclic amine/carbonyl system. *Mater. Chem. Front.* **2020,** *4* (7), 2018-2022.
228. Min, H.; Park, I. S.; Yasuda, T., Cis-quinacridone-based delayed fluorescence emitters: seemingly old but renewed functional luminogens. *Angew. Chem. Int. Ed. Engl* **2021,** *60* (14), 7643-7648.

229. Fan, X. C.; Wang, K.; Shi, Y. Z.; Chen, J. X.; Huang, F.; Wang, H.; Hu, Y. N.; Tsuchiya, Y.; Ou, X. M.; Yu, J.; Adachi, C.; Zhang, X. H., Managing intersegmental charge‐transfer and multiple resonance alignments of $D_3$-A typed TADF emitters for red OLEDs with improved efficiency and color purity. *Adv. Opt. Mater.* **2021,** *10* (3), e2101789.
230. Tsuchiya, Y.; Ishikawa, Y.; Lee, S. H.; Chen, X. K.; Brédas, J. L.; Nakanotani, H.; Adachi, C., Thermally activated delayed fluorescence properties of trioxoazatriangulene derivatives modified with electron donating groups. *Adv. Opt. Mater.* **2021,** *9* (14), e2002174.
231. Yang, S. Y.; Zou, S. N.; Kong, F. C.; Liao, X. J.; Qu, Y. K.; Feng, Z. Q.; Zheng, Y. X.; Jiang, Z. Q.; Liao, L. S., A narrowband blue circularly polarized thermally activated delayed fluorescence emitter with a hetero-helicene structure. *Chem. Commun.* **2021,** *57* (84), 11041-11044.
232. Dos Santos, J. M.; Sun, D.; Moreno-Naranjo, J. M.; Hall, D.; Zinna, F.; Ryan, S. T. J.; Shi, W.; Matulaitis, T.; Cordes, D. B.; Slawin, A. M. Z.; Beljonne, D.; Warriner, S. L.; Olivier, Y.; Fuchter, M. J.; Zysman-Colman, E., An S-shaped double helicene showing both multi-resonance thermally activated delayed fluorescence and circularly polarized luminescence. *J. Mater. Chem. C* **2022,** *10* (12), 4861-4870.
233. Liu, J.-F.; Zou, S.-N.; Chen, X.; Yang, S.-Y.; Yu, Y.-J.; Fung, M.-K.; Jiang, Z.-Q.; Liao, L.-S., Isomeric thermally activated delayed fluorescence emitters based on a quinolino[3,2,1-de]acridine-5,9-dione multiple resonance core and carbazole substituent. *Mater. Chem. Front.* **2022,** *6* (7), 966-972.
234. Yu, Y.-J.; Zou, S.-N.; Peng, C.-C.; Feng, Z.-Q.; Qu, Y.-K.; Yang, S.-Y.; Jiang, Z.-Q.; Liao, L.-S., Efficient narrowband electroluminescence based on a hetero-bichromophore thermally activated delayed fluorescence dyad. *J. Mater. Chem. C* **2022,** *10* (12), 4941-4946.
235. Wu, S.; Li, W.; Yoshida, K.; Hall, D.; Madayanad Suresh, S.; Sayner, T.; Gong, J.; Beljonne, D.; Olivier, Y.; Samuel, I. D. W.; Zysman-Colman, E., Excited-state modulation in donor-substituted multiresonant thermally activated delayed fluorescence emitters. *ACS Appl. Mater. Interfaces* **2022,** *14* (19), 22341-22352.
236. Qiu, X.; Tian, G.; Lin, C.; Pan, Y.; Ye, X.; Wang, B.; Ma, D.; Hu, D.; Luo, Y.; Ma, Y., Narrowband emission from organic fluorescent emitters with dominant low‐frequency vibronic coupling. *Adv. Opt. Mater.* **2020,** *9* (4), e2001845.
237. Huang, F.; Wang, K.; Shi, Y. Z.; Fan, X. C.; Zhang, X.; Yu, J.; Lee, C. S.; Zhang, X. H., Approaching efficient and narrow RGB electroluminescence from D-A-type TADF emitters containing an identical multiple resonance backbone as the acceptor. *ACS Appl. Mater. Interfaces* **2021,** *13* (30), 36089-36097.
238. Zou, S. N.; Peng, C. C.; Yang, S. Y.; Qu, Y. K.; Yu, Y. J.; Chen, X.; Jiang, Z. Q.; Liao, L. S., Fully bridged triphenylamine derivatives as color-tunable thermally activated delayed fluorescence emitters. *Org. Lett.* **2021,** *23* (3), 958-962.
239. Huang, J.; Hsu, Y.; Wu, X.; Wang, S.; Gan, X.; Zheng, W.; Zhang, H.; Gong, Y.; Hung, W.; Chou, P.-T.; Zhu, W., Influence of emission bandwidth by charge transfer strength for the multiple-resonance emitters via systematically tuning the electron acceptor-donor assembly. *J. Mater. Chem. C* **2022**, *10* (20), 7866-7874.
240. Yang, S.-Y.; Tian, Q.-S.; Liao, X.-J.; Wu, Z.-G.; Shen, W.-S.; Yu, Y.-J.; Feng, Z.-Q.; Zheng, Y.-X.; Jiang, Z.-Q.; Liao, L.-S., Efficient circularly polarized thermally activated delayed fluorescence hetero-[4]helicene with carbonyl-/sulfone-bridged triarylamine structures. *J. Mater. Chem. C* **2022,** *10* (11), 4393-4401.
241. Jiang, S.; Yu, Y.; Li, D.; Chen, Z.; He, Y.; Li, M.; Yang, G. X.; Qiu, W.; Yang, Z.; Gan, Y.; Lin, J.; Ma, Y.; Su, S. J., Sulfone-embedded heterocyclic narrowband emitters with strengthened molecular rigidity and suppressed high-frequency vibronic coupling. *Angew. Chem. Int. Ed.* **2023,** *62* (16), e202218892.
242. Cao, C.; Tan, J. H.; Zhu, Z. L.; Lin, J. D.; Tan, H. J.; Chen, H.; Yuan, Y.; Tse, M. K.; Chen, W. C.; Lee, C. S., Intramolecular cyclization: a convenient strategy to realize efficient BT.2020 blue multi-resonance emitter for organic light-emitting diodes. *Angew. Chem. Int. Ed.* **2023,** *62* (10), e202215226.
243. Wu, S.; Zhang, L.; Wang, J.; Kumar Gupta, A.; Samuel, I. D. W.; Zysman-Colman, E., Merging boron and carbonyl based MR-TADF emitter designs to achieve high performance pure blue OLEDs. *Angew. Chem. Int. Ed.* **2023,** *62* (28), e202305182.
244. Luo, X. F.; Li, F. L.; Zou, J. W.; Zou, Q.; Su, J.; Mao, M. X.; Zheng, Y. X., A series of fused carbazole/carbonyl based blue to yellow‐green thermally activated delayed fluorescence materials for efficient organic light‐emitting diodes. *Adv. Opt. Mater.* **2021,** *9* (21), 2100784.
245. Yu, Y. J.; Feng, Z. Q.; Meng, X. Y.; Chen, L.; Liu, F. M.; Yang, S. Y.; Zhou, D. Y.; Liao, L. S.; Jiang, Z. Q., Introducing spiro-ocks into the nitrogen/carbonyl system towards efficient narrowband deep-blue multi-resonance TADF emitters. *Angew. Chem. Int. Ed.* **2023,** *62* (40), e202310047.
246. Dos Santos, J. M.; Chan, C.-Y.; Tang, S.; Hall, D.; Matulaitis, T.; Cordes, D. B.; Slawin, A. M. Z.; Tsuchiya, Y.; Edman, L.; Adachi, C.; Olivier, Y.; Zysman-Colman, E., Color tuning of multi-resonant thermally activated delayed fluorescence emitters based on fully fused polycyclic amine/carbonyl frameworks. *J. Mater. Chem. C* **2023,** *11* (24), 8263-8273.
247. Wu, Y.; Liu, X.; Liu, J.; Yang, G.; Han, S.; Yang, D.; Cao, X.; Ma, D.; Bin, Z.; You, J., Geometry engineering of a multiple resonance core via a phenyl-embedded strategy toward highly efficient narrowband blue OLEDs. *Mater. Horiz.* **2023,** *10* (9), 3785-3790.
248. Ma, P.; Chen, Y.; Man, Y.; Qi, Q.; Guo, Y.; Wang, H.; Li, Z.; Chang, P.; Qu, C.; Han, C.; Xu, H., High-efficiency ultraviolet electroluminescence from multi-resonance phosphine oxide polycyclic aromatics. *Angew. Chem. Int. Ed.* **2023,** *63* (5), e202316479.

249. Chen, L.; Cai, J.-H.; Yu, Y.-J.; Qu, Y.-K.; Yang, S.-Y.; Zou, S.-N.; Liu, R.-H.; Zhou, D.-Y.; Liao, L.-S.; Jiang, Z.-Q., Narrowband blue emitter based on fused nitrogen/carbonyl combination with external quantum efficiency approaching 30%. *Sci. China Chem.* **2023,** *67* (1), 351-359.
250. Song, X.; Shen, S.; Zou, S.; Guo, F.; Wang, Y.; Gao, S.; Zhang, Y., Efficient narrowband organic light-emitting devices based on multi-resonance TADF emitters with secondary donor. *Chem. Eng. J.* **2023,** *467*, 143557.
251. Fan, T.; Zhu, S.; Cao, X.; Liang, X.; Du, M.; Zhang, Y.; Liu, R.; Zhang, D.; Duan, L., Tailored design of π-extended multi-resonance organoboron using indolo[3,2-b]Indole as a multi-nitrogen bridge. *Angew. Chem. Int. Ed.* **2023,** *62* (48), e202313254.
252. Chen, D.; Tenopala-Carmona, F.; Knöller, J. A.; Mischok, A.; Hall, D.; Madayanad Suresh, S.; Matulaitis, T.; Olivier, Y.; Nacke, P.; Gießelmann, F.; Laschat, S.; Gather, M. C.; Zysman-Colman, E., Mesogenic groups control the emitter orientation in multi-resonance TADF emitter films. *Angew. Chem. Int. Ed.* **2023,** *62* (16), e202218911.
253. Liang, L.; Qu, C.; Fan, X.; Ye, K.; Zhang, Y.; Zhang, Z.; Duan, L.; Wang, Y., Carbonyl-and nitrogen-embedded multi-resonance emitter with ultra-pure green emission and high electroluminescence efficiencies. *Angew. Chem. Int. Ed.* **2023**, *63* (4), e202316710.
254. Wu, S.; Kumar Gupta, A.; Yoshida, K.; Gong, J.; Hall, D.; Cordes, D. B.; Slawin, A. M. Z.; Samuel, I. D. W.; Zysman-Colman, E., Highly efficient green and red narrowband emissive organic light - emitting diodes employing multi - resonant thermally activated delayed fluorescence emitters. *Angew. Chem. In. Ed.* **2022,** *61* (52), e202213697.
255. Xu, Y.; Hafeez, H.; Seibert, J.; Wu, S.; Ortiz, J. S. O.; Crassous, J.; Bräse, S.; Samuel, I. D. W.; Zysman-Colman, E., [2.2]Paracyclophane-substituted chiral multiresonant thermally activated delayed fluorescence emitters for efficient organic light-emitting diodes. *Adv. Funct. Mater.* **2024,** *34* (47), e2402036.
256. Liu, F.-M.; Qu, Z.-H.; Zuo, P.; Yu, Y.-J.; Li, M.-T.; Liao, L.-S.; Zhou, D.-Y.; Jiang, Z.-Q., Ternary wrapped nitrogen/carbonyl multiresonance TADF emitters with quenching-resistant abilities. *ACS Mater. Lett.* **2024,** *6* (4), 1380-1387.
257. Sen, W.; Hu, Y.-N.; Wang, J.; Sun, D.; Wang, K.; Zhang, X.-H.; Zysman-Colman, E., Efficient orange organic light-emitting diodes employing a central aniline bridged multiresonant thermally activated delayed fluorescence emitter. *J. Mater. Chem. C* **2024**, *12* (17), 6177-6184.
258. Jiang, S.; Liu, D.; Chen, Z.; Yang, Z.; He, Y.; Yang, G. X.; Li, D.; Su, S. J., Carbonyl-based narrowband emitters peripherally decorated by sulfone-containing spiro structures. *Adv. Funct. Mater.* **2024,** *34* (32), e2316355.
259. Yu, J. R.; Tan, H. J.; Gao, X. Q.; Wang, B.; Long, Z. Q.; Liu, J. L.; Lin, Z. Z.; Li, X. Y.; Zhu, Z. L.; Jian, J. X.; Tong, Q. X.; Lee, C. S., Stepwise toward pure blue organic light-emitting diodes by synergetically locking and shielding carbonyl/nitrogen-based MR-TADF emitters. *Adv. Sci.* **2024,** *11* (28), e2401664.
260. Yu, Y.; Xu, L.; Tan, W.; Pan, Y.; Xiao, J.; Wang, B.; Tian, G.; Ma, Y.; Ying, L., Design high-performance narrowband emitters for blue light-emitting diodes through manipulating resonance structure of aromatic heterocycles. *Sci. China Chem.* **2025,** doi.org/10.1007/s11426-024-2478-x.
261. Jiang, Z. Q.; Liu, R. H.; Feng, Z. Q.; Ge, S. J.; Wang, Y.; Yu, Z. H.; Wu, J. R.; Yan, H. Y.; Zhou, D. Y.; Liao, L. S., Integration of through-space conjugation of adjacent arene with nitrogen/carbonyl framework for narrowband emission. *Angew. Chem. Int. Ed.* **2025**, e202424950.
262. Meng, G.; Z, J.; Wang, Q.; Huang, Y.; Zhang, G.; Duan, L.; Zhang, D. Isomeric pentagonal fusion and π-expanding of nitrogen/carbonyl-containing multi-resonant emitters for high-performance and narrowband organic electroluminescence. *Adv. Funct. Mater.* 2025**,** e2422973.
263. Seo, J. A.; Im, Y.; Han, S. H.; Lee, C. W.; Lee, J. Y., Unconventional molecular design approach of high-efficiency deep blue thermally activated delayed fluorescent emitters using indolocarbazole as an acceptor. *ACS Appl. Mater. Interfaces* **2017,** *9* (43), 37864-37872.
264. Patil, V. V.; Lee, K. H.; Lee, J. Y., A novel fluorene–indolocarbazole hybrid chromophore to assemble high efficiency deep-blue fluorescent emitters with extended device lifetime. *J. Mater. Chem. C* **2020,** *8* (9), 3051-3057.
265. Wei, J.; Zhang, C.; Zhang, D.; Zhang, Y.; Liu, Z.; Li, Z.; Yu, G.; Duan, L., Indolo[3,2,1-jk]carbazole embedded multiple-resonance fluorophors for narrowband deep-blue electroluminescence with EQE approximately 34.7% and CIEy approximately 0.085. *Angew. Chem. Int. Ed. Engl* **2021,** *60* (22), 12269-12273.
266. Hall, D.; Stavrou, K.; Duda, E.; Danos, A.; Bagnich, S.; Warriner, S.; Slawin, A. M. Z.; Beljonne, D.; Köhler, A.; Monkman, A.; Olivier, Y.; Zysman-Colma, E., Diindolocarbazole-achieving multiresonant thermally activated delayed fluorescence without the need for acceptor units. *Mater. Horiz.* **2022,** *9* (3), 1068-1080.
267. Zeng, X.; Wang, X.; Zhang, Y.; Meng, G.; Wei, J.; Liu, Z.; Jia, X.; Li, G.; Duan, L.; Zhang, D., Nitrogen-embedded multi-resonance heteroaromatics with prolonged homogeneous hexatomic rings. *Angew. Chem. Int. Ed.* **2022,** *61* (14), e202117181.
268. Patil, V. V.; Lee, H. L.; Kim, I.; Lee, K. H.; Chung, W. J.; Kim, J.; Park, S.; Choi, H.; Son, W. J.; Jeon, S. O.; Lee, J. Y., Purely spin-vibronic coupling assisted triplet to singlet up-conversion for real deep blue

organic light-emitting diodes with over 20% efficiency and y color coordinate of 0.05. *Adv. Sci.* **2021,** *8* (20), e2101137.
269. Meng, G.; Zhang, D.; Wei, J.; Zhang, Y.; Huang, T.; Liu, Z.; Yin, C.; Hong, X.; Wang, X.; Zeng, X.; Yang, D.; Ma, D.; Li, G.; Duan, L., Highly efficient and stable deep-blue OLEDs based on narrowband emitters featuring an orthogonal spiro-configured indolo[3,2,1-de]acridine structure. *Chem. Sci.* **2022,** *13* (19), 5622-5630.
270. Lee, H. L.; Jeon, S. O.; Kim, I.; Kim, S. C.; Lim, J.; Kim, J.; Park, S.; Chwae, J.; Son, W.-J.; Choi, H.; Lee, J. Y., Multiple-resonance extension and spin-vibronic-coupling-based narrowband blue organic fluorescence emitters with over 30% quantum efficiency. *Adv. Mater.* **2022,** *34* (33), e2202464.
271. Im, Y.; Han, S. H.; Lee, J. Y., Deep blue thermally activated delayed fluorescent emitters using CN-modified indolocarbazole as an acceptor and carbazole-derived donors. *J. Mater. Chem. C* **2018,** *6* (18), 5012-5017.
272. Lee, H. L.; Chung, W. J.; Lee, J. Y., Narrowband and pure violet organic emitter with a full width at half maximum of 14 nm and y color coordinate of below 0.02. *Small* **2020,** *16* (14), e1907569.
273. Patil, V. V.; Lim, J.; Lee, J. Y., Strategic Synchronization of 7,7-Dimethyl-5,7-dihydroindeno[2,1-b]carbazole for Narrow-Band, Pure Violet Organic Light-Emitting Diodes with an Efficiency of > 5% and a CIE y Coordinate of < 0.03. *ACS Appl. Mater. Interfaces* **2021,** *13* (12), 14440-14446.
274. Luo, X.; Jin, Q.; Du, M.; Wang, D.; Duan, L.; Zhang, Y., An ideal molecular construction strategy for ultra-narrow-band deep-blue emitters: balancing bathochromic-shift emission, spectral narrowing, and aggregation suppression. *Adv. Sci.* **2023**, *11* (11), e2307675.
275. Kang, J.; Jeon, S. O.; Kim, I.; Lee, H. L.; Lim, J.; Lee, J. Y., Kang, J.; Jeon, S. O.; Kim, I.; Lee, H. L.; Lim, J.; Lee, J. Y., Color stable deep blue multi-resonance organic emitters with narrow emission and high efficiency. *Adv. Sci.* **2023,** *10* (26), e2302619.
276. Luo, M.; Li, W.; Du, S.; Zhang, J.; Wang, Z.; Zhang, X.; Li, Y.; Ge, Z., Purely nitrogen‐based multi‐resonance deep‐blue emitter with an ultralow y color coordinate of < 0.03 via rationally intramolecular charge transfer. *Adv. Opt. Mater.* **2023,** *11* (16), 2300491.
277. Cho, H. H.; Congrave, D. G.; Gillett, A. J.; Montanaro, S.; Francis, H. E.; Riesgo-Gonzalez, V.; Ye, J.; Chowdury, R.; Zeng, W.; Etherington, M. K.et al. Suppression of Dexter transfer by covalent encapsulation for efficient matrix-free narrowband deep blue hyperfluorescent OLEDs. *Nat. Mater.* **2024,** *23*, 519-526.
278. Fan, T.; Liu, Q.; Zhang, H.; Wang, X.; Zhang, D.; Duan, L., Enhancing spin-orbit coupling in an indolocarbazole multiresonance emitter by a sulfur-containing peripheral substituent for a fast reverse intersystem crossing. *Adv. Mater.* **2024,** *36* (45), e2408816.
279. Wang, Z.; Hu, X.; Yan, Z.; Liang, J.; Song, X.; Chen, Q.; Bi, H.; Wang, Y., Achieving narrowband and stable pure blue organic light-emitting diodes by employing molecular vibration limited strategies in the extended pi-conjugated indolocarbazole skeleton. *Adv. Sci.* **2025,** *12* (9), e2410479.
280. Shi, A.; Zhao, G.; Yang, R.; Liu, X.; Huang, F.; Chen, Z.; Jiang, W.; Wang, Y.; Ai, X.; Ma, Z.; Li, Y.; Shao, S., Fused nonacyclic carbonyl/nitrogen-containing multi-resonance emitters with simultaneously redshifted and narrowed emission spectra for high-efficiency solution-processed OLEDs. *Chem. Eng. J.* **2025,** *507*, 160102.
281. Zhang, Y.; Zhang, D.; Wei, J.; Hong, X.; Lu, Y.; Hu, D.; Li, G.; Liu, Z.; Chen, Y.; Duan, L., Achieving pure green electroluminescence with CIEy of 0.69 and EQE of 28.2% from an aza‐fused multi‐resonance emitter. *Angew. Chem. In. Ed.* **2020,** *59* (40), 17499-17503.
282. Jiang, P.; Zhan, L.; Cao, X.; Lv, X.; Gong, S.; Chen, Z.; Zhou, C.; Huang, Z.; Ni, F.; Zou, Y.; Yang, C., Simple acridan-based multi-resonance structures enable highly-efficient narrowband green TADF electroluminescence. *Adv. Opt. Mater.* **2021,** 9 *(21)*, e2100825.
283. Zhang Y, Zhang D, Huang T, Li G, Zhang C, Lu Y, et al. Beating the limitation of energy gap law utilizing deep red MR-TADF emitter with narrow energy-bandwidth. *ChemRxiv*. **2020,** doi:10.26434/chemrxiv.13202975.v1.
284. Gan, Y.; Peng, X.; Qiu, W.; Wang, L.; Li, D.; Xie, W.; Liu, D.; Li, M.; Lin, J.; Su, S.-J., Multiple charge transfer disk-like emitters with fast fluorescence radiation rate and high horizontal dipole orientation for pure blue organic light-emitting diodes. *Chem. Eng. J.* **2022,** *430*, 133030.
285. Zhan, L.; Ying, A.; Qi, Y.; Wu, K.; Tang, Y.; Tan, Y.; Zou, Y.; Xie, G.; Gong, S.; Yang, C., Copper(I) complex as sensitizer enables high‐performance organic light‐emitting diodes with very low efficiency roll‐off. *Adv. Funct. Mater.* **2021,** *31* (48), 2106345.
286. Chen, Z.; Zhong, C.; Han, J.; Miao, J.; Qi, Y.; Zou, Y.; Xie, G.; Gong, S.; Yang, C., High-performance circularly polarized electroluminescence with simultaneous narrowband emission, high efficiency, and large dissymmetry factor. *Adv. Mater.* **2022,** *34* (17), 2109147.
287. Cho, S. M.; Youn, K. M.; Yang, H. I.; Lee, S. H.; Naveen, K. R.; Karthik, D.; Jeong, H.; Kwon, J. H., Anthracene-dibenzofuran based electron transport type hosts for long lifetime multiple resonance pure blue OLEDs. *Org. Electron.* **2022,** *105*, 106501.
288. Yan, Z. P.; Yuan, L.; Zhang, Y.; Mao, M. X.; Liao, X. J.; Ni, H. X.; Wang, Z. H.; An, Z.; Zheng, Y. X.; Zuo, J. L., A chiral dual-core organoboron structure realizes dual-channel enhanced ultrapure blue emission and highly efficient circularly polarized electroluminescence. *Adv. Mater.* **2022,** *34* (36), e2204253.

289. Yin, C.; Zhang, D.; Zhang, Y.; Lu, Y.; Wang, R.; Li, G.; Duan, L., High-efficiency narrow-band electro-fluorescent devices with thermally activated delayed fluorescence sensitizers combined through-bond and through-space charge transfers. *CCS Chem.* **2020,** *2* (4), 1268-1277.
290. Zhao, G.; Liu, D.; Wang, P.; Huang, X.; Chen, H.; Zhang, Y.; Zhang, D.; Jiang, W.; Sun, Y.; Duan, L., Exceeding 30 % external quantum efficiency in non-doped OLEDs utilizing solution processable tadf emitters with high horizontal dipole orientation via anchoring strategy. *Angew. Chem. Int. Ed. Engl* **2022,** *61* (45), e202212861.
291. Chen, L.; Chang, Y.; Shu, H.; Li, Q.; Shi, S.; Wang, S.; Wang, L., Achieving efficient solution ‐ processed blue narrowband emitting oleds with small efficiency roll ‐ off by using a bulky TADF sensitizer with high reverse intersystem crossing rate. *Adv. Opt. Mater.* **2022,** *11* (2), e2201898.
292. Li, N.; Chen, Z.; Zhou, C.; Ni, F.; Huang, Z.; Cao, X.; Yang, C., Versatile host materials for both D-A type and multi-resonance TADF emitters towards solution-processed OLEDs with nearly 30% EQE. *Adv. Mater.* **2023**, *35* (28), e2300510.
293. Tang, X.; Tsagaantsooj, T.; Rajakaruna, T. P. B.; Wang, K.; Chen, X. K.; Zhang, X. H.; Hatakeyama, T.; Adachi, C., Stable pure-green organic light-emitting diodes toward Rec.2020 standard. *Nat. Commun.* **2024,** *15* (1), 4394.
294. Wang, J.; Chen, D.; Moreno-Naranjo, J. M.; Zinna, F.; Frederic, L.; Cordes, D. B.; McKay, A. P.; Fuchter, M. J.; Zhang, X.; Zysman-Colman, E., Helically chiral multiresonant thermally activated delayed fluorescent emitters and their use in hyperfluorescent organic light-emitting diodes. *Chem. Sci.* **2024,** *15* (41), 16917-16927.
295. Kim, J. H.; Chung, W. J.; Kim, J.; Lee, J. Y., Concentration quenching-resistant multiresonance thermally activated delayed fluorescence emitters. *Mater. Today Energy* **2021,** *21*, 100792.
296. Qiu, Y.; H. X.; Miao, J.; Huang, Z.; Li, N.; Cao, X.; Han, J.; Zhou, C.; Zhong, C.; Yang, C., Narrowing the electroluminescence spectra of multi-resonance emitters for high-performance blue OLEDs by a peripheral decoration strategy. *ACS Appl. Mater. Interfaces* **2021,** *13* (49), 59035-59042.
297. Hyung Suk Kim, H. J. C., Sang Hoon Lee, Junho Kim, Seunghyup Yoo, Yun- Hi Kim, Chihaya Adachi. Advancing efficiency in deep-blue OLEDs: Exploring a machine learning–driven multiresonance TADF molecular design. *Sci. Adv.* **2025,** *11* (4), eadr1326.
298. Wang, M.; Fu, Z.; Cheng, R.; Du, J.; Wu, T.; Bin, Z.; Wu, D.; Yang, Y.; Lan, J., Dibenzo[b,d]furan/thiophene-fused double boron-based multiresonance emitters with narrowband ultrapure green electroluminescence. *Chem. Commun.* **2023,** *59* (34), 5126-5129.
299. Nemma, H.; Kori, Y.; Meguro, N.; Mimura, R.; Chiba, Y.; Kido, J.; Sasabe, H., π-extended phenoxazine ‐ based asymmetric mr ‐ tadf emitters for narrow emission band green oleds with a power efficiency of 150 lm $w^{-1}$, an EQE of 27%, and an $LT_{95}$ of over 3000 h at 1000 $cdm^{-2}$. *Adv. Opt. Mater.* **2024,** *13* (2), e2402131.
300. Li, H. Z.; Xie, F. M.; Bai, J. Y.; Zhang, K.; Shi, H. N.; Liu, J. Y.; Li, X.; Tang, J. X.; Li, Y. Q., Spiral ‐ locking strategy for efficient narrowband multiple resonance thermally activated delayed fluorescence emitters. *Small* **2024,** *20* (52), e2407220.
301. Xiao, X.; Hu, J. J.; Huo, Z. Z.; Liang, J. Q.; Yang, B.; Hong, X. F.; Chen, Z. J.; Wang, Y.; Li, C. H.; Zheng, Y. X., Construction of concentration quenching ‐ resistant multi ‐ resonance TADF emitters via positional isomerization for OLEDs. *Adv. Opt. Mater.* **2024,** *12* (35), e2401754.
302. Luo, X. F.; Song, S. Q.; Wu, X.; Yip, C. F.; Cai, S.; Zheng, Y. X., A chiral spirofluorene-embedded multiple-resonance thermally activated delayed fluorescence emitter for efficient pure-green circularly polarized electroluminescence. *Aggregate* **2023,** *5* (2), e445.
303. Zhong, R.; Wang, M.; Wang, X.; Wang, S.; Shao, S.; Wang, L., 10-Dibenzothiophenyl-9,9-diphenylacridane-based multiple resonance emitters for high-efficiency narrowband green OLEDs with CIE y > 0.7 at high doping concentrations. *Chem. Sci.* **2024,** *15* (33), 13290-13298.
304. Song, S. Q.; Yip, C. F.; Liu, Q. M.; Zhong, X. S.; Wang, Y.; Zheng, Y. X., Efficient OLEDs with alleviated efficiency roll ‐ off based on MR-TADF materials containing indolo[3,2,1 ‐ jk]carbazole. *Adv. Opt. Mater.* **2024,** *12* (19), e2400200.
305. Cheng, H.; Lan, J.; Yang, Y.; Bin, Z., Spirobifluorene-fused strategy enables pure-green multiple resonance emitters with low efficiency roll-off. *Mater. Horiz.* **2024,** *11* (19), 4674-4680.
